# Evaluation of Different Anthropometric Indicators for Screening for Nonalcoholic Fatty Liver Disease in Elderly Individuals

**DOI:** 10.1155/2021/6678755

**Published:** 2021-01-28

**Authors:** Yan Zhang, Bo Li, Nan Liu, Peixi Wang, Jinghua He

**Affiliations:** ^1^Zhujiang Hospital, Southern Medical University, 253 Gongye Road, Guangzhou, Guangdong 510280, China; ^2^Institute of Chronic Disease Risks Assessment, Henan University, Jinming Campus, Kaifeng, Henan 457004, China; ^3^Pingshan District People's Hospital of Shenzhen, Pingshan General Hospital of Southern Medical University, Shenzhen, Guangdong 518118, China

## Abstract

**Objective:**

To explore the anthropometric indicators suitable for screening for nonalcoholic fatty liver disease (NAFLD) in the elderly population.

**Methods:**

This cross-sectional study screened subjects over 65 years, who had undergone a physical examination in 2019. Their height, weight, waist circumference, and fasting blood glucose and triglyceride levels were measured. Body mass index (BMI), waist circumstance (WC), waist-to-height ratio (WHtR), relative fat mass (RFM), ponderal index (PI), conicity index (CI), lipid accumulation product (LAP), and body shape index (ABSI) were calculated. Statistical analyses were performed using the Chi-square test, logistic regression, and receiver operating characteristic (ROC) curve.

**Subjects:**

Of a total of 4985 subjects, 1173 diagnosed with NAFLD and 3812 without NAFLD were included.

**Results:**

The NAFLD group had increased BMI, WC, WHtR, RFM, PI, CI, and LAP. ABSI was only significantly different in males between the groups. Logistic regression analysis showed that RFM was an effective prognostic factor for males with NAFLD, and LAP, BMI, and WC were effective prognostic factors for females. ROC curve analysis showed that LAP played a significant role in the prediction of NAFLD.

**Conclusion:**

LAP is closely related to the occurrence of NAFLD and could be an efficient screening and treatment tool for NAFLD in the elderly people. *Lay Summary*. We conducted a screening and study of nonalcoholic fatty liver disease in the elderly population by determining the association between obesity indexes and nonalcoholic fatty liver disease. We found that LAP is practical, easy-to-measure tool for screening and studying NAFLD in the high-risk community elderly population, making it a valuable indicator in research.

## 1. Introduction

Nonalcoholic fatty liver disease (NAFLD) is liver damage caused by the accumulation of triglycerides (hepatic steatosis) in liver cells, similar to that caused by alcohol, but it occurs in people who do not abuse alcohol [1]. The occurrence and development of this disease is related to type 2 diabetes mellitus (T2DM), obesity, and metabolic syndrome [2, 3]. Because of the obesity epidemic, NAFLD has become a major cause of liver lesions in adults and children worldwide, with a global prevalence of 24%; its prevalence is highest in South America and the Middle East, followed by Asia, the United States, and Europe [4]. In the United States, NAFLD and especially nonalcoholic steatohepatitis (NASH) can lead to advanced liver disease: cirrhosis and liver cancer [5]. NASH is considered the second leading indicator of liver transplantation [6] in the United States after chronic hepatitis [7]. NAFLD has gradually replaced hepatitis B virus infection as the highest risk factor for liver cirrhosis and even liver cancer [8]. At the same time, there is growing evidence that NAFLD, a multisystem disease, may lead to an increased incidence of diabetes, metabolic syndrome, and cardiovascular disease [9–12]. If the prevalence of obesity and T2DM levels off in the future, we project a modest growth in total NAFLD cases (0%–30%), between 2016 and 2030, with the highest growth in China as a result of urbanization and the lowest growth in Japan as a result of a shrinking population [13].

The gold standard for the diagnosis of NAFLD is the histological findings of a liver biopsy, which are consistent with the pathological diagnostic criteria for fatty liver disease [14–17]. However, this approach is often not feasible in population screening and clinical practice. Although there are other methods, ultrasonography (US) is the most frequently used modality to diagnose fatty liver [18] and has been endorsed by the guidelines for the Asia-Pacific region [19].

The diagnostic criteria for fatty liver on abdominal US are two of three abnormalities: a diffusely increased liver echogenicity near the field of the ultrasound echo (i.e., “bright liver); the echo of the liver being greater than that of the kidney or spleen; and the vascular blurring and gradual attenuation of the fat field of the ultrasound echo [19]. It was previously reported that patients with NAFLD had no history of an alcohol drinking habit or the ethanol intake per week was less than 140 g in men (70 g in women) in the past 12 months, and there is no specific disease that can cause steatosis, such as viral hepatitis, drug-induced liver disease, autoimmune liver disease, and so on [16].

Despite the huge economic and clinical burden of NAFLD, liver US has become a safe and effective test method, but as a screening method, it is still cumbersome and expensive for screening for NAFLD [20, 21], especially in the elderly population. Older people often suffer from various chronic diseases and have a greater need for screening and intervention for NAFLD than younger people. Because of the relationship between obesity and NAFLD, it is necessary to find simple and effective anthropometric indexes to help screen and treat NAFLD in the elderly population.

Body mass index (BMI) and waist circumference (WC) are the most common indicators of obesity and have been shown to have a certain correlation with NAFLD [22]. There are new anthropometric indicators that are more sensitive and specific in screening for NAFLD. Traditional obesity-related indicators include BMI, WC, ponderal index (PI) [23], and conicity index (CI) [24]. New anthropometric indicators introduced in recent years include the waist-to-height ratio (WHtR) [25], relative fat mass (RFM) [26], lipid accumulation product (LAP) [27], and a body shape index (ABSI) [28]. The associations between different anthropometric indicators and NAFLD are different. Currently, the indicators with the strongest correlation with NAFLD are still controversial, and these indicators are related to demographic characteristics, such as sex, age, and ethnicity. In this study, we aimed to explore different anthropometric indicators for screening for NAFLD in the elderly population.

## 2. Materials and Methods

### 2.1. Ethics Statements

The Henan University approved the study protocol. This study was conducted in conformance with the principles of ethical standards. Written informed consent was obtained from all participants.

### 2.2. Study Design, Data Collection, and Measurements

In 2019, the community health service center in the Pearl River Delta region in Southern China organized a free physical examination for persons older than 65 years of age within the scope of management. This project was part of the national basic public health service. The physical examination included but was not limited to a general examination, routine blood test, routine urine test, liver function analysis (alanine transaminase, aspartate transaminase, and total bilirubin levels), renal function analysis (serum creatinine and urea level), fasting blood glucose (FBG) analysis, blood lipid analysis, electrocardiography, and abdominal US. Then, doctors provided health advice and guidance to the patients based on the results of the physical examination. Subjects with a positive hepatitis virus test result or those who were diagnosed with hepatitis were excluded. A total of 4985 subjects were included in this cross-sectional study based on data from this physical examination.

Trained investigators administered a face-to-face structured questionnaire to document specified demographic data (age and sex) and information about health-related behavior (smoking, alcohol use, physical intensity, diet, history of hepatitis, and so on). Blood pressure of both subject's arms was measured by trained observers using an automatic electronic sphygmomanometer. The mean reading of all replicate measurements was recorded for further analysis. Hypertension was defined as systolic blood pressure ≥140 mmHg and/or diastolic blood pressure ≥90 mmHg, or if the subjects were diagnosed with hypertension.

The anthropometric indexes, including weight and height, were measured when the subjects wore light clothes without shoes. WC was gauged with an inelastic soft ruler at 1 cm above the navel, measured, and recorded at the end of the subjects' natural breath.

The subjects were reminded to fast overnight for at least 8 hours, and the subjects' venous blood samples were collected by professional medical personnel for the evaluation of FBG, triglyceride (TG), and other levels.

### 2.3. Ultrasonography

An experienced physician scanned the subjects using real-time US by an experienced physician, and the operating physician was blinded to the subject's biochemical test results and personal clinical diagnostic information.

### 2.4. Diagnostic Criteria

According to the Chinese Diagnostic Criteria for Nonalcoholic and Alcoholic Fatty Liver Disease, a diagnosis of NAFLD was based on subjects' test results [29].The calculations of the obesity indexes are as follows:

  BMI (kg/m^2^) = weight (kg)/height (m^2^),
  WC (cm): obesity, men: WC ≥ 85 cm, women: WC ≥ 80 cm,
  WHtR (cm/cm) = WC (cm)/height (cm); central obesity, WHtR ≥ 0.5,
  RFM = 64 − (20 × height)/WC (cm) + (12 × sex), male = 0, female = 1,
  PI (kg/m^3^) = weight (kg)/height (m^3^),
  CI = (m^1/2^·kg^−1/2^) = WC (cm)/[0.109 × weight (kg)/height (m)^1/2^],
  LAP = men: (WC (cm)–65) × TG, women: (WC (cm)–58) × TG,
  ABSI (kg^−2/3^·m^11/6^) = WC (cm)/[BMI (kg/m^2^)^2/3^ × height (m)^1/2^].

Smoker was defined as current smoker or had been smoking for more than 6 months, and alcohol use was defined as drinking more than 50 ml per day or had been drinking alcohol continuously or accumulatively for more than 1 year. Regarding the physical intensity level, we created a variable that added all activities for each individual and then categorized the activities into three levels: lower (zero or one activity in the past week), middle (two or more activities per week in the past month), and high (more than one activity per day in the past month).

### 2.5. Statistical Analysis

Statistical analyses were performed using SPSS 20.0 for Windows (IBM Corp.). Subjects' total data were classified by the presence or absence of NAFLD and stratified by sex. All subjects with missing data were eliminated. Continuous data are expressed as mean ± standard deviation (SD) and compared using the *t*-test. Categorical data were analyzed using the Chi-square test. Comparisons between different anthropometric indexes were assessed using Pearson's test. Logistic regression analysis was used to analyze the relationship between different anthropometric indicators and NAFLD, and the odds ratio (OR) and 95% confidence interval (CI) were used to reflect the correlations. BMI, WC, WHtR, RFM, PI, CI, LAP, and ABSI, adiposity phenotypes, were modeled as continuous variables and divided into quartile segments to evaluate their impact on the prevalence of NAFLD. Since the calculations of LAP and RFM are different in men and women, the relationship between different anthropometric indicators and NAFLD were analyzed separately for men and women. The area under the curve (AUC) in the receiver operating characteristic (ROC) analysis was calculated to assess the diagnostic ability of all the anthropometric indexes for screening for NAFLD and determine the optimal cut-off point with Youden's index (sensitivity + specificity − 1 was the highest). All statistical comparisons were two-tailed, and *P*values < 0.05 were considered significant.

## 3. Result

This study included 4985 Chinese elderly individuals (1971 men and 3014 women; age: 73.8 ± 6.4 years, range: 66–115 years). Among these subjects, 505 (25.6%) men and 1068 (35.4%) women were diagnosed with NAFLD by us.  Table 1 shows the characteristics of the individuals included in the current analysis. In both sexes, the number of cases of NAFLD tended to increase with age (*P*< 0.05). Furthermore, we found that participants with hypertension had a higher risk of NAFLD than nonhypertensive participants (*P* < 0.05). Comparison of the NAFLD and control groups revealed that the groups were significantly different in terms of alcohol use in men, but this difference was not observed with respect to alcohol use in women probably because very few women in our cohort used alcohol. Physical activity made sense for women but not for men. In both sexes, participants in the NAFLD group exhibited significantly higher values of FBG, BMI, WC, WHtR, RFM, PI, CI, and LAP than those in the control group. The NAFLD and control groups were significantly different in terms of ABSI in men but not in women, and the ROC curve demonstrated that ABSI may not be a good predictor of NAFLD.

Table 2 lists the anthropometrics criteria for determining obesity; the RFM, PI, CI, LAP, and ABSI decision criteria indicate no obesity, while the universal cut-off points for BMI and waist circumference are not appropriate for use worldwide [30].

Tables 3 and 4 show the results of the association of the anthropometric quartile with NAFLD; model 1 was unadjusted. Model 2 was adjusted for age, alcohol use, physical activity, hypertension, and FBG level. When all anthropometric indexes were classified by quartile range, which we found before and after adjustment of the model, the OR value corresponding to each anthropometric index was significantly higher than that of the reference group. Additionally, with the increase of the quartile level of each anthropometric index, the OR value corresponding to each anthropometric index also gradually increased (*P* < 0.001), except for ABSI in women, for which we found no difference in increased ABSI in women after adjusting the model.

**Figure 1:**
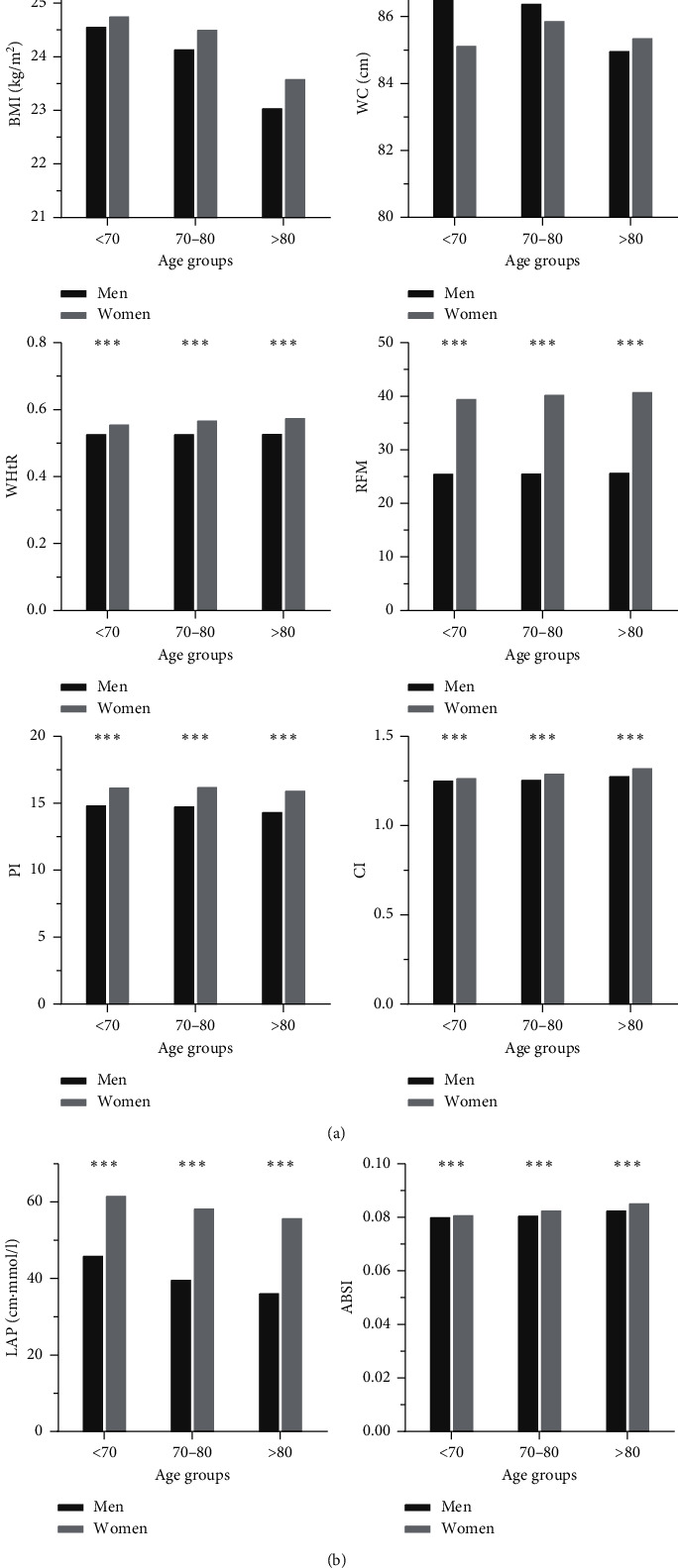
The difference in anthropometric indicators between genders and ages. ^*∗*^*P* < 0.05, ^*∗∗*^*P* < 0.01, and ^*∗∗∗*^*P* < 0.001.

As shown in Figure 1, the difference in anthropometric indicators between ages for men and women, WHtR, RFM, CI, and ABSI increased with age, but BMI, WC, PI, and LAP decreased with age in the Chinese elderly population. However, for each age-specific group, men consistently had significantly greater WHtR, RFM, PI, CI, LAP, and ABSI than women did (all, *P* < 0.001).

For each *Z*-score standardization, as shown in Table 5, after adjusting for confounding factors, a 1-SD increment change for most adiposity indexes was associated with a higher risk of NAFLD in the sex-specific and age-specific groups. RFM had the highest OR in men in all age groups (≤70 years: 4.53, 70–80 years: 3.48, and >80 years: 4.28). However, in women, LAP had the highest OR in the ≤70-year-old group (3.59), followed by BMI (2.63) in the 70–80-year-old group, and WC had the highest OR in women aged 80 years or older (2.76).

**Figure 2:**
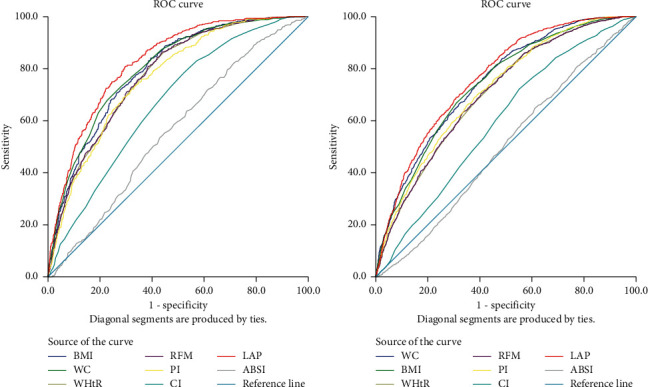
ROC curve showing the performance of different anthropometric indexes in predicting NAFLD.

Table 6 presents the optional cut-off values and corresponding sensitivity, specificity, and AUC of each adiposity index for identifying NAFLD by sex. LAP had the highest AUC and highest cut-off value. When the cut-off values were 0.827 in men and 0.765 in women, Youden's indexes (men: sum of sensitivity, 79.8%; specificity, 70.4%; women: 71.0% and 67.0%, respectively) were the largest (Figure 2).

**Figure 3:**
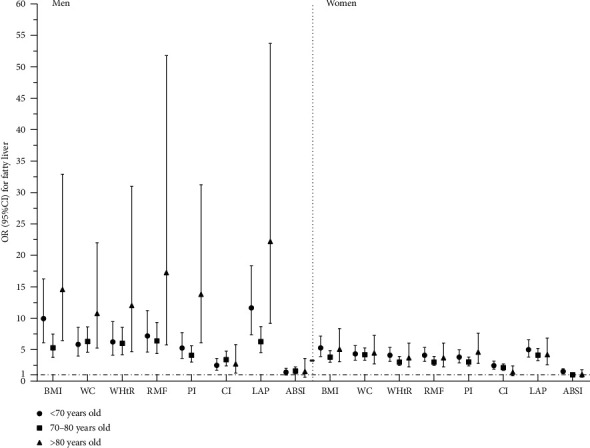
After adjusting for confounding factors, the optimal cut-off value was taken as the demarcation point for the correlation between anthropometric indexes and NAFLD. Confounding variables: age, drinking, physical activity, hypertension, and FBG.

Figure 3 shows the relationship between NAFLD and the conversion of anthropometric indicators into dichotomous variables, as determined by the optimal cut-off point for age and sex stratification after adjusting for confounding variables. In addition to ABSI, we found that the other anthropometric indexes had a low optimal critical value as a reference, and the OR (95% CI) corresponding to the high critical value was greater than 1.

## 4. Discussion

The increase in the prevalence and severity of NAFLD has been associated with rising trends in obesity [31], particularly in morbidly obese patients, and it can reach 90% [32]. Obesity can lead to the development of metabolic syndrome and complications, including NAFLD, T2DM, hypertension, hyperlipidemia, and cardiovascular diseases (CVDs) [33]; thus, NAFLD is likely to represent the hepatic manifestation of the metabolic syndrome [34]. In common obesity or conditions lacking adipose tissue such as lipodystrophies [35], hepatocytes store extra lipids, resulting in intrahepatic fat accumulation (simple steatosis [SS]), which is farther amplified by the high dietary fat and carbohydrates commonly observed in obesity. The latter increases de nova lipogenesis. Uncontrolled obesity leads to the development and progression of inflammatory processes in the liver. Cirrhosis and liver cancer are the results of the continuous and vigorous response to these processes [36]. There is also so-called metabolically “obese” normal-weight (MONW) people who are lean with metabolic dysfunction [37]. Because of their body size, they are classified as lean people according to their BMI, but they have metabolic syndrome risks. For groups such as MONW, conventional obesity indicators cannot be used for discrimination of NAFLD, so more appropriate obesity indicators should be used to screen the population. Therefore, this study used different anthropometric indicators and developed simple and sensitive indicators for NAFLD screening in China.

In this cross-sectional study, BMI, WC, WHtR, RFM, PI, CI, LAP, and ABSI were measured, and in the logistic regression, after adjusting confounding factors, RFM had the highest OR values in different age groups of men (<70 years: 4.856, 70–80 years: 3.485, and >80 years: 4.192). The result was different in women. In different age groups, different ORs indicated the maximum value. For instance, LAP had the highest value in the ≤70-year-old group (3.585); in the 70–80-year-old group and >80-year-old group, BMI (2.634) and WC (2.758) had the highest ORs. In the analysis of NAFLD with the ROC curve, we found that LAP was significantly associated with NAFLD in men and women. LAP had the largest AUC in both sexes and thus had the best diagnostic value. When the cut-off of LAP was 35.97 in men, the highest sensitivity and specificity were 80.7% and 70.4%, respectively, and when the cut-off of LAP was 49.17 in women, the sensitivity and specificity were 71.0% and 67.0%, respectively.

NAFLD has become a global problem with high morbidity and mortality, but there is no medication specifically approved for its treatment [38]. Lifestyle modification remains the cornerstone of NAFLD management. Based on these observations and given that steatosis is a prerequisite, followed by hepatic inflammation and fibrosis, this steatosis may remain unchanged for years [38]. We have proposed that the prevention or solution to SS may be an effective way to intervene and prevent the sustainable development of NAFLD [39]. Weight loss interventions (diet and exercise) have been shown to reduce all-cause mortality in obese adults [40]. In a recent review of NAFLD in older women, lifestyle modifications with weight loss and exercise were regarded as first-line treatment [41], and data from studies on animal models indicate that exercise training effectively reduces liver fat accumulation [42, 43].

Using obesity indicators to prevent obesity should be a major goal for policymakers and healthcare systems [36]. BMI as a traditional obesity index is not fully applicable to populations with metabolic dysfunction. WC is better than BMI, an index of obesity boundary, is closely related to abdominal fat content, and is considered to represent visceral stored fat [44]. However, the cut-off proposed by WC varies between diverse races and countries [45], which makes it more difficult for WC to become the universal obesity screening tool. Compared with WC, TG was included in LAP, and it is reasonable to speculate that the two LAP components—that is, enlarged abdominal fat depots and increased TG concentration—are each an indication that available lipid fuels have exceeded the individual's capacity to buffer and safely store this major form of acquired energy [27]. Therefore, LAP can replace various clinical indicators of lipid accumulation and become a prerequisite for the diagnosis of metabolic syndrome [46, 47]. LAP has been shown to be able to identify various diseases such as insulin resistance, CVDs [48], polycystic ovary syndrome [46], and hypertension and to assist in defining the concept of systemic fat abdominal visceral adipose tissue separation [49].

In conclusion, LAP is simple and easy to measure, and it is very suitable for research and as a practical tool for screening and studying NAFLD in the high-risk community elderly population. Its diagnostic accuracy of LAP is higher in men than in women. Therefore, elderly men with high levels of LAP need to be vigilant, and diet and exercise intervention should be carried out as soon as possible for weight loss. In this study, the causal relationship between anthropometric indicators and NAFLD could not be assessed because of the cross-sectional study design; therefore, a prospective cohort study may yield better analysis and prediction of NAFLD indicators. The lack of hip measurements in our data collection prevents other obesity-related anthropometric indicators (such as waist-to-hip ratio) from being applied to statistical analysis. Hip measurement is an important indicator of body shape, but further studies may be needed to confirm the correlation between hip measurements and NAFLD. Participants in this study were all residents of southern China, so our findings may not be applicable to other countries, regions, or ethnic groups. Large prospective multicenter cohort studies are needed to demonstrate the reliability of these results in a population.

## Figures and Tables

**Table 1:** Characteristics of subjects with NAFLD stratified by sex.

Variables	Male (*n* = 1971)			Female (*n* = 3014)		
	Non-NAFLD	NAFLD	*P* value	Non-NAFLD	NAFLD	*P* value
Age (years) [*n* (%)]			<0.001			<0.001
−70	426 (68.6)	195 (31.4)		642 (59.8)	432 (40.2)	
70–80	774 (74.9)	260 (25.1)		945 (64.3)	525 (35.7)	
−80	266 (84.2)	50 (15.8)		359 (76.4)	111 (23.6)	
Smoking [*n* (%)]	624 (75.5)	202 (24.5)	>0.05	15 (71.4)	6 (28.6)	>0.05
Drinking [*n* (%)]	120 (66.3)	61 (33.7)	<0.05	3 (60.0)	2 (40.0)	>0.05
Physical activity [*n* (%)]			>0.05			<0.05
Lower	458 (77.9)	130 (22.1)		577 (69.0)	259 (31.0)	
Middle	114 (73.5)	41 (26.5)		154 (63.9)	87 (36.1)	
High	894 (72.8)	334 (27.2)		1210 (62.7)	720 (37.3)	
Hypertension [*n* (%)]	881 (70.2)	374 (29.8)	<0.001	1224 (61.5)	766 (38.5)	<0.001
Fasting glucose (mmol/l)	5.6 ± 1.8	6.1 ± 2.0	<0.001	5.6 ± 1.7	6.3 ± 2.4	<0.001
BMI (kg/m^2^)	23.2 ± 3.2	26.7 ± 2.9	<0.001	23.4 ± 3.4	26.6 ± 3.3	<0.001
WC (cm)	83.8 ± 9.0	93.6 ± 7.6	<0.001	83.0 ± 9.0	90.7 ± 7.9	<0.001
WHtR	0.52 ± 0.06	0.57 ± 0.05	<0.001	0.55 ± 0.06	0.60 ± 0.06	<0.001
RFM	24.8 ± 4.3	28.7 ± 2.8	<0.001	39.4 ± 4.3	42.3 ± 3.1	<0.001
PI (kg/m^3^)	14.3 ± 2.1	16.3 ± 1.9	<0.001	15.6 ± 2.4	17.6 ± 2.4	<0.001
CI	1.26 ± 0.08	1.30 ± 0.06	<0.001	1.29 ± 0.85	1.31 ± 0.07	<0.001
LAP (cm·mmol/l)	31.2 ± 31.3	72.1 ± 50.4	<0.001	45.5 ± 42.7	83.6 ± 66.4	<0.001
ABSI	0.082 ± 0.005	0.082 ± 0.004	<0.001	0.083 ± 0.005	0.083 ± 0.005	>0.05

Data are expressed as mean ± standard deviation and numbers (percentage) as appropriate.

**Table 2:** Normal range of anthropometric indexes.

	Normal
Male	
BMI (kg/m^2^)	18.5–24.9
WC (cm)	≤102 (≤44 in)
WHtR	<0.5

Female	
BMI (kg/m^2^)	18.5–24.9
WC (cm)	≤88 (≤35 in)
WHtR	<0.5

**Table 3:** Multivariate logistic of anthropology indexes for NAFLD in men.

Variables	Model 1	*P*	Model 2	*P*
BMI		<0.001		<0.001
Q1 (≤21.75)	1.00		1.00	
Q2 (21.75–24.06)	5.36 (3.03–9.50)		5.22 (2.93–9.31)	
Q3 (24.06–26.26)	15.13 (8.75–26.15)		14.75 (8.47–25.67)	
Q4 (>26.26)	35.87 (20.83–61.77)		34.01 (19.60–58.99)	

WC		<0.001		<0.001
Q1 (≤80.0)	1.00		1.00	
Q2 (80.0–87.0)	5.13 (3.06–8.58)		4.94 (2.94–8.28)	
Q3 (87.0–93.0)	12.39 (7.54–20.36)		11.65 (7.06–19.22)	
Q4 (>93.0)	34.07 (20.78–55.86)		30.96 (18.78–51.06)	

WHtR		<0.001		<0.001
Q1 (≤0.49)	1.00		1.00	
Q2 (0.49–0.53)	4.44 (2.73–7.24)		4.25 (2.60–6.94)	
Q3 (0.53–0.57)	10.65 (6.70–16.92)		9.90 (6.20–15.80)	
Q4 (>0.57)	26.62 (16.65–42.55)		24.14 (15.01–38.82)	

RFM		<0.001		<0.001
Q1 (≤23.49)	1.00		1.00	
Q2 (23.49–26.44)	4.66 (2.78–7.83)		4.48 (2.66–7.54)	
Q3 (26.44–28.78)	12.03 (7.33–19.76)		11.31 (6.85–18.65)	
Q4 (>28.78)	25.99 (15.90–42.50)		23.73 (14.42–39.03)	

PI		<0.001		<0.001
Q1 (≤13.34)	1.00		1.00	
Q2 (13.34–14.74)	5.76 (3.46–9.60)		5.54 (3.30–9.30)	
Q3 (14.74–16.16)	11.24 (6.84–18.47)		11.09 (6.69–18.38)	
Q4 (>16.16)	24.71 (15.12–40.38)		23.49 (14.24–38.72)	

CI		<0.001		<0.001
Q1 (≤1.22)	1.00		1.00	
Q2 (1.22–1.27)	2.67 (1.89–3.76)		2.44 (1.72–3.45)	
Q3 (1.27–1.32)	4.19 (3.02–5.83)		3.70 (2.65–5.16)	
Q4 (>1.32)	5.44 (3.90–7.60)		4.55 (3.24–6.41)	

LAP		<0.001		<0.001
Q1 (≤16.66)	1.00		1.00	
Q2 (16.66–31.08)	8.89 (4.21–18.77)		8.44 (3.99–17.83)	
Q3 (31.08–53.20)	26.42 (12.81–54.52)		23.92 (11.57–49.49)	
Q4 (>53.20)	84.64 (41.16–174.05)		75.62 (36.69–155.87)	

ABSI		<0.05		<0.05
Q1 (≤0.0784)	1.00		1.00	
Q2 (0.0784–0.0813)	1.66 (1.23–2.26)		1.53 (1.12–2.09)	
Q3 (0.0813–0.0842)	2.01 (1.56–2.82)		1.87 (1.38–2.53)	
Q4 (>0.0842)	1.73 (1.28–2.35)		1.46 (1.07–1.99)	

Model 1: unadjusted. Model 2: adjusted for age, drinking, physical activity, FGE, and hypertension.

**Table 4:** Multivariate logistic of anthropology indexes for NAFLD in women.

Variables	Model 1	*P*	Model 2	*P*
BMI		<0.001		<0.001
Q1 (≤22.07)	1.00		1.00	
Q2 (22.07–24.53)	3.29 (2.49–4.35)		3.16 (2.38–4.20)	
Q3 (24.53–26.84)	5.84 (4.46–7.66)		5.36 (4.07–7.07)	
Q4 (>26.84)	13.23 (10.07–17.37)		11.99 (9.09–15.83)	

WC		<0.001		<0.001
Q1 (≤80.0)	1.00		1.00	
Q2 (80.0–86.0)	2.77 (2.13–3.60)		2.58 (1.97–3.37)	
Q3 (86.0–92.0)	6.01 (4.68–7.71)		5.55 (4.30–7.16)	
Q4 (>92.0)	10.72 (8.27–13.89)		9.90 (7.60–12.90)	

WHtR		<0.001		<0.001
Q1 (≤0.53)	1.00		1.00	
Q2 (0.53–0.57)	2.94 (2.30–3.76)		2.79 (2.17–3.59)	
Q3 (0.57–0.61)	4.55 (3.58–5.77)		4.34 (3.40–5.54)	
Q4 (>0.61)	7.86 (6.15–10.05)		8.07 (6.26–10.40)	

RFM		<0.001		<0.001
Q1 (≤38.11)	1.00		1.00	
Q2 (38.11–41.00)	2.64 (2.04–3.41)		2.45 (1.89–3.18)	
Q3 (41.00–43.23)	4.42 (3.44–5.68)		4.09 (3.17–5.29)	
Q4 (>43.23)	7.87 (6.12–10.12)		7.84 (6.05–10.14)	

PI		<0.001		<0.001
Q1 (≤14.52)	1.00		1.00	
Q2 (14.52–16.23)	3.04 (2.34–3.96)		2.92 (2.24–3.82)	
Q3 (16.23–17.87)	4.83 (3.73–6.25)		4.59 (3.53–5.97)	
Q4 (>17.87 V)	9.58 (7.41–12.39)		8.90 (6.84–11.57)	

CI		<0.001		<0.001
Q1 (≤1.24)	1.00		1.00	
Q2 (1.24–1.30)	2.17 (1.75–2.70)		1.97 (1.58–2.47)	
Q3 (1.30–1.35)	2.46 (1.96–3.09)		2.37 (1.88–3.00)	
Q4 (>1.35)	2.57 (2.04–3.23)		2.69 (2.12–3.41)	

LAP		<0.001		<0.001
Q1 (≤27.60)	1.00		1.00	
Q2 (27.60–46.08)	5.09 (3.72–6.98)		4.90 (3.56–6.73)	
Q3 (46.08–72.21)	9.01 (6.62–12.26)		8.32 (6.09–11.37)	
Q4 (>72.21)	21.85 (16.03–29.80)		19.75 (14.42–27.05)	

ABSI		<0.05		>0.05
Q1 (≤0.080)	1.00		1.00	
Q2 (0.080–0.0828)	1.16 (0.94–1.43)		1.11 (0.90–1.38)	
Q3 (0.0828–0.0863)	1.28 (1.04–1.58)		1.24 (1.00–1.53)	
Q4 (>0.0863)	0.94 (0.76–1.17)		1.03 (0.82–1.28)	

Model 1: unadjusted. Model 2: adjusted for age, physical activity, FGE, and hypertension.

**Table 5:** Standardized odds ratios and 95% confidence interval of different anthropometric indexes for NAFLD under gender and age stratification.

		Age groups (years)		
		≤70	70–80	>80
Male				
BMI *Z*-score	Crude OR	4.30 (3.27–5.64)^a^	3.01 (2.50–3.62)^a^	3.51 (2.36–5.22)^a^
	Adjusted OR	4.17 (3.17–5.50)^a^	2.97 (2.46–3.59)^a^	3.76 (2.47–5.74)^a^
WC *Z*-score	Crude OR	4.56 (3.42–6.07)^a^	3.52 (2.88–4.32)^a^	3.83 (2.53–5.80)^a^
	Adjusted OR	4.40 (3.28–5.91)^a^	3.46 (2.81–4.26)^a^	3.74 (2.44–5.73)^a^
WHtR *Z*-score	Crude OR	3.84 (3.02–5.15)^a^	3.01 (2.50–3.65)^a^	3.42 (2.29–5.11)^a^
	Adjusted OR	3.68 (2.81–4.81)^a^	2.97 (2.44–3.60)^a^	3.33 (2.20–5.04)^a^
RFM *Z*-score	Crude OR	4.77 (3.49–6.53)^a^	3.55 (2.85–4.42)^a^	4.41 (2.70–7.22)^a^
	Adjusted OR	4.53 (3.29–6.24)^a^	3.48 (2.78–4.36)^a^	4.28 (2.58–7.11)^a^
PI *Z*-score	Crude OR	3.39 (2.66–4.31)^a^	2.50 (2.11–2.97)^a^	2.84 (1.98–4.06)^a^
	Adjusted OR	3.29 (2.58–4.21)^a^	2.47 (2.08–2.94)^a^	2.99 (2.06–4.41)^a^
CI *Z*-score	Crude OR	2.01 (1.64–2.46)^a^	2.02 (1.71–2.38)^a^	2.01 (1.42–2.85)^a^
	Adjusted OR	1.87 (1.52–2.30)^a^	1.92 (1.62–2.27)^a^	1.86 (1.32–2.71)^c^
LAP *Z*-score	Crude OR	4.46 (3.29–6.05)^a^	2.93 (2.41–3.56)^a^	3.95 (2.68–5.83)^b^
	Adjusted OR	4.35 (3.17–6.05)^a^	2.82 (2.32–3.44)^a^	4.04 (2.68–6.09)^a^
ABSI *Z*-score	Crude OR	1.25 (1.05–1.48)^a^	1.30 (1.13–1.50)^a^	1.26 (0.93–1.71)^b^
	Adjusted OR	1.16 (0.97–1.39)^c^	1.23 (1.07–1.43)^c^	1.19 (0.86–1.64)^c^

Female				
BMI *Z*-score	Crude OR	2.87 (2.42–3.39)^a^	2.67 (2.33–3.07)^a^	2.69 (2.07–3.49)^a^
	Adjusted OR	2.75 (2.32–3.27)^a^	2.63 (2.29–3.03)^a^	2.63 (2.00–3.46)^a^
WC *Z*-score	Crude OR	3.11 (2.61–3.70)^a^	2.55 (2.21–2.93)^a^	2.89 (2.18–3.82)^a^
	Adjusted OR	3.02 (2.52–3.61)^a^	2.48 (2.15–2.86)^a^	2.76 (2.06–3.69)^a^
WHtR *Z*-score	Crude OR	2.86 (2.42–3.39)^a^	2.14 (1.88–2.44)^a^	2.16 (1.68–2.78)^a^
	Adjusted OR	2.76 (2.33–3.28)^a^	2.11 (1.85–2.41)^a^	2.12 (1.64–2.78)^a^
RFM *Z*-score	Crude OR	3.24 (2.68–3.92)^a^	2.30 (1.99–2.64)^a^	2.38 (1.79–3.16)^a^
	Adjusted OR	3.13 (2.58–3.80)^a^	2.26 (1.95–2.61)^a^	2.32 (1.72–3.14)^a^
PI *Z*-score	Crude OR	2.54 (2.17–2.98)^a^	2.27 (1.99–2.58)^a^	2.19 (1.72–2.78)^a^
	Adjusted OR	2.44 (2.08–2.86)^a^	2.24 (1.97–2.56)^a^	2.15 (1.67–2.78)^a^
CI *Z*-score	Crude OR	1.83 (1.59–2.10)^a^	1.35 (1.21–1.51)^a^	1.32 (1.06–1.65)^b^
	Adjusted OR	1.76 (1.53–2.03)^a^	1.32 (1.19–1.48)^a^	1.32 (1.05–1.67)^b^
LAP *Z*-score	Crude OR	3.95 (3.07–5.07)^a^	2.70 (2.23–3.06)^a^	2.03 (1.61–2.55)^a^
	Adjusted OR	3.59 (2.79-4.61)^a^	2.50 (2.13–2.93)^a^	1.82 (1.43–2.32)^a^
ABSI *Z*-score	Crude OR	1.23 (1.08–1.39)^b^	0.95 (0.86–1.06)^c^	0.94 (0.76–1.17)^c^
	Adjusted OR	1.20 (1.05–1.36)^b^	0.94 (0.84–1.05)^c^	0.96 (0.76–1.20)^c^

a: *P* < 0.001, b: *P* < 0.05, and c: *P* > 0.05; OR: odds ratio. Adjusted OR: adjusted for age, drinking, sedentary behavior, physical activity, FGE, and hypertension.

**Table 6:** The cut-off, sensitivities, specificities, Youden's index, and area under the curve of different variable for the screening of NAFLD in men and women.

	Cut-off	Sensitivity (%)	Specificity (%)	Youden's index	AUC (95% CI)
Male (1971/505)					
BMI	24.01	83.4	61.3	0.45	0.795 (0.774–0.816)
WC	90.00	70.4	74.5	0.45	0.801 (0.781–0.822)
WHtR	0.53	86.3	55.5	0.42	0.776 (0.754–0.798)
RFM	26.04	85.5	56.1	0.42	0.778 (0.756–0.800)
PI	15.09	72.3	66.7	0.40	0.766 (0.744–0.788)
CI	1.26	78.2	48.0	0.26	0.670 (0.644–0.695)
LAP	36.15	79.8	70.4	0.51	0.827 (0.808–0.846)
ABSI	0.079	77.8	28.4	0.11	0.561 (0.533–0.589)

Female (3014/1068)					
BMI	23.99	78.7	56.7	0.35	0.747 (0.729–0.764)
WC	87.75	67.0	68.9	0.36	0.739 (0.721–0.757)
WHtR	0.57	70.0	59.1	0.29	0.704 (0.686–0.723)
RFM	40.89	70.4	59.6	0.30	0.705 (0.687–0.724)
PI	16.20	70.2	60.8	0.31	0.717 (0.699–0.736)
CI	1.28	72.2	44.9	0.17	0.594 (0.574–0.615)
LAP	49.17	71.0	67.0	0.38	0.765 (0.748–0.782)
ABSI	0.081	64.8	38.9	0.04	0.500 (0.479–0.521)

AUC: area under the curve; 95% CI: 95% confidence interval.

## Data Availability

The data used to support the findings of this study are provided in Supplementary Materials.

## Supplementary Materials

Supplementary Materials The data in supplementary file are additional datasets provided for the help of readers.
