# Supplementary material for: Evaluation of Different Anthropometric Indicators for Screening for Nonalcoholic Fatty Liver Disease in Elderly Individuals
Source: Int J Endocrinol. 2021 Jan 28;2021:6678755. doi: 10.1155/2021/6678755 (PMC7861948; doi:10.1155/2021/6678755)
Supplement: Supplementary Materials — The data in supplementary file are additional datasets provided for the help of readers. [file 6678755.f1.pdf]

年龄	是否高血	是否糖尿	是否老年	性别	血压左侧	血压左侧	血压右侧	血压右侧
70	是	否	是	女	130.00	70.00	135.00	80.00
73	否	否	是	女	135.00	85.00	140.00	90.00
67	否	否	是	女	118.00	70.00	126.00	73.00
68	否	否	是	女	120.00	82.00	130.00	90.00
71	是	是	是	女	130.00	80.00	135.00	82.00
71	否	否	是	女	118.00	73.00	121.00	68.00
74	是	否	是	女	125.00	80.00	130.00	80.00
72	是	否	是	女	140.00	86.00	159.00	96.00
66	是	否	是	女	130.00	80.00	120.00	80.00
68	是	是	是	女	120.00	72.00	132.00	80.00
68	否	否	是	女	108.00	72.00	112.00	78.00
75	否	否	是	女	124.00	82.00	130.00	76.00
77	否	否	是	女	130.00	70.00	138.00	78.00
77	是	否	是	女	134.00	80.00	139.00	85.00
66	否	否	是	女	110.00	70.00	120.00	72.00
70	否	否	是	女	110.00	60.00	105.00	60.00
69	否	否	是	女	138.00	80.00	135.00	80.00
68	否	否	是	女	151.00	80.00	148.00	75.00
68	是	否	是	女	126.00	78.00	130.00	80.00
69	否	否	是	女	106.00	70.00	110.00	70.00
76	否	否	是	女	112.00	80.00	116.00	60.00
69	否	否	是	女	133.00	73.00	135.00	80.00
73	是	是	是	女	140.00	88.00	132.00	82.00
68	是	是	是	女	145.00	86.00	142.00	78.00
67	否	否	是	女	135.00	79.00	136.00	73.00
69	否	否	是	女	138.00	86.00	144.00	93.00
70	是	是	是	女	107.00	78.00	121.00	85.00
73	是	否	是	女	135.00	75.00	131.00	79.00
68	是	否	是	女	150.00	82.00	157.00	88.00
78	是	是	是	女	146.00	87.00	144.00	85.00
89	是	否	是	女	158.00	76.00	159.00	72.00
87	否	否	是	女	138.00	86.00	143.00	83.00
70	是	否	是	女	110.00	70.00	108.00	68.00
66	是	否	是	女	176.00	90.00	182.00	92.00
69	是	是	是	女	120.00	82.00	124.00	80.00
66	否	否	是	女	110.00	68.00	112.00	70.00
68	是	是	是	女	140.00	75.00	142.00	80.00
66	否	否	是	女	150.00	82.00	162.00	74.00
71	是	否	是	女	130.00	80.00	135.00	78.00
68	是	否	是	女	122.00	81.00	125.00	83.00
76	否	否	是	女	155.00	78.00	160.00	78.00
68	否	否	是	女	150.00	86.00	158.00	90.00
72	否	否	是	女	123.00	68.00	122.00	65.00
71	是	否	是	女	140.00	70.00	138.00	74.00
71	是	是	是	女	110.00	82.00	113.00	82.00
75	否	否	是	女	110.00	75.00	114.00	79.00
75	否	否	是	女	105.00	76.00	110.00	70.00
71	否	否	是	女	158.00	99.00	167.00	89.00
66	是	否	是	女	135.00	80.00	138.00	84.00
71	否	否	是	女	130.00	74.00	130.00	76.00

87	是	否	是	女	116.00	60.00	127.00	61.00
91	否	否	是	女	110.00	70.00	108.00	68.00
70	是	否	是	女	128.00	82.00	130.00	85.00
77	是	否	是	女	142.00	81.00	129.00	73.00
74	是	是	是	女	136.00	60.00	138.00	60.00
70	是	是	是	女	150.00	80.00	145.00	80.00
74	是	否	是	女	142.00	76.00	140.00	76.00
69	是	是	是	女	148.00	84.00	146.00	82.00
73	是	是	是	女	158.00	78.00	148.00	75.00
70	否	否	是	女	131.00	71.00	132.00	75.00
67	是	否	是	女	144.00	98.00	145.00	99.00
87	是	否	是	女	100.00	70.00	106.00	77.00
73	是	否	是	女	147.00	86.00	147.00	77.00
75	是	是	是	女	145.00	107.00	163.00	109.00
80	否	否	是	女	138.00	88.00	145.00	64.00
72	是	否	是	女	120.00	80.00	128.00	85.00
69	否	否	是	女	111.00	62.00	116.00	68.00
69	是	否	是	女	148.00	86.00	145.00	88.00
76	是	否	是	女	121.00	71.00	120.00	70.00
75	是	否	是	女	124.00	80.00	130.00	76.00
75	是	是	是	女	139.00	78.00	141.00	89.00
68	否	是	是	女	130.00	78.00	132.00	80.00
68	否	否	是	女	120.00	76.00	128.00	78.00
69	否	否	是	女	129.00	68.00	135.00	72.00
75	是	否	是	女	138.00	60.00	146.00	64.00
71	是	否	是	女	120.00	73.00	134.00	84.00
90	是	是	是	女	150.00	71.00	151.00	75.00
66	否	否	是	女	124.00	86.00	121.00	79.00
69	是	否	是	女	131.00	89.00	129.00	90.00
77	否	否	是	女	125.00	75.00	130.00	80.00
70	否	否	是	女	116.00	56.00	112.00	58.00
72	是	否	是	女	118.00	68.00	121.00	72.00
78	是	否	是	女	127.00	68.00	124.00	68.00
83	是	否	是	女	150.00	85.00	154.00	90.00
74	是	否	是	女	125.00	77.00	126.00	86.00
66	否	否	是	女	110.00	80.00	118.00	74.00
74	是	是	是	女	126.00	78.00	130.00	80.00
68	否	否	是	女	122.00	77.00	121.00	75.00
70	是	否	是	女	133.00	79.00	139.00	80.00
68	否	否	是	女	105.00	65.00	110.00	70.00
66	否	否	是	女	137.00	89.00	139.00	88.00
82	是	否	是	女	155.00	80.00	157.00	80.00
73	是	是	是	女	130.00	70.00	148.00	81.00
77	是	是	是	女	181.00	106.00	189.00	100.00
68	否	否	是	女	146.00	84.00	155.00	88.00
72	是	否	是	女	130.00	82.00	134.00	80.00
66	否	否	是	女	180.00	100.00	182.00	110.00
72	否	否	是	女	130.00	80.00	125.00	74.00
77	是	否	是	女	132.00	80.00	130.00	80.00
78	否	否	是	女	150.00	90.00	160.00	70.00
66	否	否	是	女	90.00	60.00	92.00	62.00

66	否	否	是	女	106.00	72.00	104.00	70.00
79	否	否	是	女	106.00	62.00	110.00	68.00
69	否	否	是	女	118.00	74.00	122.00	76.00
74	是	否	是	女	130.00	80.00	138.00	88.00
66	否	否	是	女	150.00	89.00	152.00	90.00
79	是	否	是	女	144.00	78.00	139.00	79.00
67	是	是	是	女	126.00	70.00	131.00	78.00
77	否	否	是	女	133.00	79.00	135.00	82.00
71	否	否	是	女	135.00	82.00	137.00	84.00
66	否	否	是	女	135.00	70.00	135.00	80.00
66	否	否	是	女	190.00	100.00	201.00	110.00
69	否	否	是	女	120.00	70.00	126.00	78.00
67	否	否	是	女	148.00	78.00	150.00	80.00
70	是	否	是	女	136.00	65.00	139.00	72.00
71	否	否	是	女	126.00	70.00	125.00	68.00
69	否	否	是	女	120.00	70.00	122.00	70.00
67	否	否	是	女	130.00	75.00	136.00	76.00
72	是	否	是	女	190.00	106.00	185.00	107.00
76	否	否	是	女	110.00	68.00	112.00	70.00
68	是	否	是	女	120.00	75.00	130.00	78.00
72	否	否	是	女	126.00	68.00	130.00	70.00
68	否	否	是	女	123.00	84.00	120.00	74.00
66	否	否	是	女	128.00	82.00	133.00	85.00
74	否	否	是	女	128.00	76.00	130.00	77.00
74	否	否	是	女	135.00	85.00	135.00	82.00
72	否	否	是	女	128.00	72.00	126.00	70.00
83	是	否	是	女	130.00	80.00	140.00	78.00
76	是	否	是	女	144.00	90.00	153.00	89.00
70	否	否	是	女	128.00	79.00	130.00	88.00
70	否	是	是	女	125.00	75.00	130.00	80.00
76	是	是	是	女	151.00	83.00	154.00	74.00
69	是	是	是	女	140.00	84.00	142.00	86.00
73	是	否	是	女	135.00	78.00	138.00	82.00
74	否	否	是	女	150.00	89.00	160.00	90.00
77	是	否	是	女	134.00	84.00	139.00	89.00
75	是	否	是	女	132.00	82.00	130.00	84.00
71	是	否	是	女	138.00	80.00	135.00	80.00
67	是	否	是	女	142.00	91.00	142.00	92.00
66	否	否	是	女	118.00	62.00	110.00	60.00
71	否	否	是	女	130.00	82.00	132.00	84.00
75	是	否	是	女	166.00	99.00	143.00	95.00
75	否	是	是	女	125.00	70.00	128.00	78.00
67	是	是	是	女	148.00	83.00	132.00	77.00
67	否	否	是	女	101.00	68.00	100.00	70.00
75	是	否	是	女	143.00	83.00	150.00	87.00
71	否	否	是	女	136.00	68.00	139.00	70.00
72	是	否	是	女	200.00	85.00	208.00	94.00
66	否	否	是	女	116.00	81.00	129.00	87.00
71	否	否	是	女	130.00	68.00	137.00	70.00
74	否	是	是	女	139.00	80.00	126.00	78.00
80	是	否	是	女	135.00	78.00	140.00	80.00

69	否	否	是	女	110.00	68.00	112.00	68.00
69	是	否	是	女	127.00	70.00	131.00	74.00
69	是	否	是	女	138.00	78.00	139.00	80.00
76	是	否	是	女	110.00	70.00	120.00	78.00
66	否	否	是	女	135.00	86.00	137.00	85.00
69	是	否	是	女	136.00	78.00	139.00	80.00
66	是	否	是	女	123.00	70.00	132.00	73.00
72	否	否	是	女	110.00	70.00	118.00	70.00
81	是	是	是	女	130.00	60.00	125.00	70.00
69	是	否	是	女	147.00	91.00	159.00	94.00
73	是	否	是	女	152.00	97.00	144.00	91.00
66	是	是	是	女	120.00	78.00	130.00	80.00
86	否	否	是	女	114.00	68.00	116.00	70.00
77	否	否	是	女	140.00	75.00	143.00	72.00
66	否	否	是	女	108.00	64.00	118.00	64.00
82	是	否	是	女	150.00	62.00	150.00	80.00
68	是	是	是	女	143.00	86.00	142.00	84.00
67	否	否	是	女	105.00	70.00	108.00	68.00
73	否	否	是	女	126.00	70.00	130.00	70.00
72	是	否	是	女	139.00	85.00	139.00	89.00
78	否	否	是	女	138.00	66.00	141.00	68.00
76	是	否	是	女	170.00	75.00	172.00	73.00
67	否	否	是	女	140.00	88.00	140.00	84.00
68	是	否	是	女	124.00	82.00	126.00	82.00
74	是	否	是	女	167.00	97.00	166.00	89.00
72	是	否	是	女	150.00	80.00	151.00	83.00
74	是	是	是	女	138.00	76.00	140.00	70.00
81	是	是	是	女	130.00	70.00	131.00	68.00
78	否	否	是	女	126.00	79.00	128.00	80.00
71	否	否	是	女	115.00	84.00	120.00	84.00
79	是	否	是	女	138.00	68.00	140.00	70.00
70	是	否	是	女	116.00	70.00	118.00	70.00
70	是	否	是	女	120.00	78.00	120.00	80.00
71	否	否	是	女	126.00	80.00	130.00	84.00
68	否	否	是	女	100.00	66.00	112.00	70.00
71	否	否	是	女	116.00	67.00	120.00	70.00
86	否	否	是	女	120.00	70.00	122.00	74.00
75	否	是	是	女	138.00	82.00	120.00	70.00
71	是	否	是	女	160.00	74.00	161.00	84.00
81	是	是	是	女	152.00	70.00	150.00	70.00
84	是	否	是	女	130.00	80.00	140.00	80.00
75	否	否	是	女	115.00	81.00	110.00	80.00
68	是	是	是	女	142.00	88.00	148.00	90.00
79	否	否	是	女	136.00	58.00	138.00	60.00
83	是	否	是	女	102.00	60.00	100.00	60.00
89	否	否	是	女	120.00	70.00	122.00	72.00
80	否	否	是	女	140.00	70.00	140.00	60.00
80	否	否	是	女	110.00	74.00	120.00	80.00
67	否	否	是	女	128.00	80.00	130.00	82.00
73	否	否	是	女	110.00	61.00	112.00	62.00
68	是	否	是	女	142.00	90.00	144.00	92.00

80	是	是	是	女	123.00	70.00	126.00	78.00
66	是	否	是	女	142.00	72.00	152.00	84.00
69	否	否	是	女	170.00	100.00	170.00	90.00
78	否	否	是	女	129.00	90.00	125.00	94.00
69	否	否	是	女	120.00	70.00	124.00	74.00
67	是	是	是	女	139.00	72.00	144.00	71.00
78	是	否	是	女	120.00	76.00	120.00	70.00
75	是	否	是	女	168.00	69.00	165.00	80.00
79	否	是	是	女	121.00	73.00	124.00	76.00
81	是	是	是	女	130.00	71.00	130.00	70.00
87	否	否	是	女	160.00	70.00	166.00	70.00
75	是	否	是	女	110.00	74.00	109.00	73.00
68	是	否	是	女	124.00	88.00	122.00	78.00
78	否	否	是	女	145.00	81.00	142.00	80.00
76	否	否	是	女	136.00	70.00	139.00	72.00
72	是	否	是	女	144.00	96.00	146.00	98.00
71	否	否	是	女	136.00	80.00	140.00	80.00
72	否	否	是	女	120.00	64.00	116.00	72.00
70	是	否	是	女	131.00	94.00	133.00	95.00
71	否	否	是	女	120.00	70.00	126.00	74.00
89	是	否	是	女	130.00	70.00	132.00	70.00
85	否	否	是	女	126.00	68.00	130.00	70.00
73	是	否	是	女	134.00	80.00	139.00	85.00
74	是	否	是	女	145.00	67.00	141.00	81.00
80	否	否	是	女	140.00	76.00	144.00	80.00
67	否	否	是	女	121.00	60.00	135.00	69.00
67	否	否	是	女	125.00	88.00	120.00	85.00
75	否	否	是	女	107.00	70.00	100.00	70.00
68	是	否	是	女	130.00	80.00	123.00	78.00
68	是	否	是	女	130.00	75.00	132.00	80.00
73	否	否	是	女	130.00	74.00	130.00	75.00
68	否	否	是	女	124.00	76.00	126.00	78.00
71	否	否	是	女	135.00	81.00	132.00	80.00
70	否	否	是	女	109.00	65.00	115.00	80.00
77	是	否	是	女	104.00	63.00	105.00	64.00
70	否	否	是	女	140.00	72.00	130.00	79.00
78	是	否	是	女	156.00	84.00	152.00	86.00
71	否	否	是	女	140.00	93.00	145.00	84.00
80	否	否	是	女	126.00	78.00	130.00	78.00
87	否	否	是	女	140.00	80.00	136.00	78.00
79	是	否	是	女	116.00	76.00	119.00	74.00
71	否	否	是	女	145.00	79.00	158.00	88.00
72	否	否	是	女	136.00	70.00	138.00	75.00
70	是	是	是	女	152.00	70.00	150.00	74.00
68	否	否	是	女	110.00	76.00	115.00	75.00
75	否	否	是	女	168.00	72.00	164.00	82.00
67	否	否	是	女	136.00	70.00	138.00	78.00
79	否	否	是	女	145.00	95.00	150.00	100.00
66	否	否	是	女	108.00	68.00	110.00	70.00
77	否	否	是	女	110.00	85.00	113.00	89.00
71	否	否	是	女	147.00	79.00	153.00	83.00

71	否	否	是	女	129.00	76.00	139.00	79.00
74	否	否	是	女	134.00	76.00	136.00	78.00
87	是	是	是	女	130.00	65.00	133.00	73.00
69	否	否	是	女	120.00	76.00	115.00	77.00
75	是	否	是	女	132.00	75.00	130.00	75.00
72	是	是	是	女	155.00	72.00	132.00	78.00
73	否	是	是	女	98.00	66.00	100.00	70.00
80	是	否	是	女	114.00	62.00	116.00	65.00
71	否	否	是	女	125.00	74.00	128.00	76.00
69	否	否	是	女	135.00	80.00	132.00	78.00
67	否	否	是	女	120.00	70.00	127.00	80.00
68	是	否	是	女	142.00	89.00	139.00	88.00
75	是	是	是	女	136.00	64.00	130.00	60.00
69	否	否	是	女	108.00	66.00	109.00	67.00
74	是	是	是	女	152.00	71.00	154.00	75.00
77	是	是	是	女	155.00	97.00	156.00	92.00
76	否	否	是	女	100.00	75.00	116.00	84.00
70	是	否	是	女	148.00	89.00	144.00	88.00
70	是	否	是	女	140.00	85.00	135.00	80.00
80	是	否	是	女	159.00	81.00	166.00	72.00
76	是	否	是	女	115.00	67.00	110.00	74.00
70	是	否	是	女	129.00	84.00	128.00	76.00
67	否	否	是	女	144.00	82.00	141.00	79.00
82	是	是	是	女	155.00	98.00	144.00	94.00
67	否	否	是	女	96.00	70.00	94.00	72.00
77	是	否	是	女	132.00	78.00	134.00	80.00
92	是	否	是	女	116.00	60.00	118.00	60.00
66	否	否	是	女	125.00	73.00	128.00	70.00
89	是	否	是	女	135.00	60.00	132.00	60.00
72	否	否	是	女	128.00	76.00	130.00	78.00
66	否	否	否	女	110.00	68.00	105.00	67.00
67	否	否	是	女	120.00	70.00	122.00	72.00
67	否	否	是	女	118.00	76.00	122.00	80.00
96	是	否	是	女	197.00	78.00	178.00	80.00
67	是	否	是	女	130.00	68.00	136.00	70.00
70	否	否	是	女	122.00	80.00	120.00	80.00
69	是	否	是	女	124.00	89.00	138.00	92.00
76	是	否	是	女	112.00	78.00	120.00	80.00
73	否	否	是	女	122.00	70.00	120.00	80.00
70	否	否	是	女	120.00	78.00	124.00	80.00
73	是	是	是	女	145.00	80.00	140.00	80.00
78	是	否	是	女	145.00	70.00	140.00	70.00
74	否	是	是	女	114.00	70.00	110.00	70.00
71	否	否	是	女	120.00	70.00	130.00	80.00
78	否	否	是	女	109.00	62.00	124.00	67.00
68	是	否	是	女	122.00	67.00	122.00	69.00
70	否	否	是	女	166.00	80.00	168.00	80.00
66	否	否	是	女	134.00	88.00	126.00	85.00
83	是	否	是	女	120.00	62.00	122.00	68.00
70	是	否	是	女	154.00	80.00	150.00	80.00
75	是	否	是	女	130.00	75.00	130.00	77.00

94	是	否	是	女	130.00	70.00	130.00	68.00
77	是	是	是	女	130.00	80.00	133.00	81.00
67	是	是	是	女	128.00	79.00	125.00	75.00
70	是	是	是	女	164.00	80.00	156.00	70.00
78	否	否	是	女	160.00	80.00	160.00	80.00
72	是	否	是	女	118.00	60.00	120.00	60.00
78	是	否	是	女	145.00	77.00	148.00	80.00
76	否	否	是	女	115.00	78.00	120.00	70.00
66	否	否	是	女	130.00	69.00	124.00	74.00
66	是	是	是	女	139.00	72.00	130.00	67.00
72	否	否	是	女	158.00	95.00	160.00	98.00
73	否	是	是	女	145.00	60.00	140.00	60.00
81	是	否	是	女	130.00	75.00	140.00	80.00
66	是	否	是	女	142.00	94.00	150.00	96.00
74	是	否	是	女	135.00	78.00	130.00	75.00
67	否	是	是	女	152.00	90.00	150.00	84.00
68	否	否	是	女	136.00	82.00	138.00	84.00
67	是	是	是	女	140.00	76.00	148.00	80.00
85	是	否	是	女	140.00	80.00	142.00	80.00
66	否	否	是	女	110.00	75.00	107.00	77.00
69	是	否	是	女	124.00	64.00	125.00	72.00
67	是	否	是	女	128.00	80.00	130.00	80.00
69	否	否	是	女	105.00	65.00	110.00	70.00
69	是	否	是	女	138.00	84.00	138.00	90.00
85	否	否	是	女	131.00	87.00	126.00	82.00
67	是	否	是	女	125.00	84.00	121.00	79.00
76	是	否	是	女	152.00	78.00	158.00	80.00
66	是	否	是	女	128.00	80.00	130.00	80.00
67	否	否	是	女	139.00	70.00	138.00	68.00
81	是	否	是	女	140.00	82.00	144.00	86.00
67	是	否	是	女	145.00	90.00	151.00	96.00
99	否	否	是	女	95.00	60.00	93.00	60.00
71	否	否	是	女	130.00	70.00	132.00	72.00
73	是	否	是	女	106.00	77.00	110.00	76.00
82	是	否	是	女	140.00	76.00	142.00	80.00
69	否	否	是	女	142.00	86.00	156.00	98.00
73	是	否	是	女	120.00	72.00	116.00	74.00
79	是	是	是	女	148.00	72.00	152.00	67.00
72	是	否	是	女	116.00	70.00	120.00	74.00
74	否	否	是	女	150.00	86.00	144.00	84.00
83	否	否	是	女	136.00	68.00	138.00	70.00
77	是	否	是	女	143.00	81.00	144.00	82.00
67	是	否	是	女	159.00	81.00	160.00	83.00
77	否	否	是	女	111.00	68.00	110.00	70.00
66	是	否	是	女	108.00	65.00	110.00	70.00
80	否	是	是	女	161.00	89.00	145.00	81.00
71	是	否	是	女	138.00	72.00	135.00	70.00
77	否	否	是	女	138.00	71.00	154.00	87.00
68	是	否	是	女	132.00	80.00	136.00	80.00
73	否	否	是	女	130.00	70.00	128.00	78.00
68	是	否	是	女	121.00	76.00	120.00	70.00

75	否	否	是	女	127.00	86.00	128.00	77.00
67	否	否	是	女	139.00	80.00	140.00	88.00
88	否	否	是	女	166.00	89.00	168.00	89.00
73	否	否	是	女	130.00	70.00	135.00	80.00
67	否	是	是	女	134.00	80.00	135.00	83.00
67	是	是	是	女	130.00	84.00	135.00	89.00
72	是	否	是	女	140.00	82.00	142.00	80.00
71	是	否	是	女	130.00	80.00	132.00	82.00
68	否	否	是	女	123.00	71.00	134.00	73.00
85	否	否	是	女	137.00	78.00	138.00	80.00
90	是	否	是	女	141.00	78.00	159.00	73.00
74	是	否	是	女	110.00	74.00	120.00	82.00
76	否	否	是	女	158.00	94.00	156.00	96.00
74	是	否	是	女	130.00	70.00	135.00	74.00
81	否	否	是	女	134.00	84.00	133.00	86.00
68	否	否	是	女	130.00	92.00	134.00	94.00
67	是	否	是	女	132.00	78.00	138.00	80.00
74	否	否	是	女	137.00	85.00	139.00	89.00
68	否	否	是	女	137.00	90.00	150.00	85.00
75	否	否	是	女	128.00	72.00	134.00	68.00
77	否	否	是	女	122.00	68.00	124.00	70.00
78	是	否	是	女	130.00	70.00	133.00	80.00
69	否	否	是	女	158.00	102.00	163.00	100.00
70	否	是	是	女	118.00	80.00	131.00	84.00
77	是	是	是	女	163.00	97.00	155.00	87.00
67	否	否	是	女	150.00	80.00	154.00	80.00
70	否	否	是	女	129.00	85.00	132.00	78.00
84	否	否	是	女	141.00	84.00	132.00	80.00
68	否	否	是	女	135.00	70.00	130.00	74.00
70	否	否	是	女	140.00	84.00	142.00	78.00
82	否	是	是	女	135.00	71.00	138.00	85.00
81	是	否	是	女	134.00	68.00	138.00	64.00
70	否	否	是	女	142.00	88.00	139.00	85.00
67	是	否	是	女	146.00	88.00	150.00	92.00
74	否	否	是	女	160.00	90.00	150.00	80.00
77	否	否	是	女	134.00	75.00	139.00	80.00
68	否	否	是	女	160.00	90.00	190.00	90.00
79	否	是	是	女	130.00	61.00	132.00	64.00
75	否	否	是	女	130.00	80.00	123.00	70.00
73	是	否	是	女	140.00	76.00	144.00	78.00
84	否	否	是	女	170.00	70.00	172.00	70.00
69	是	否	是	女	138.00	78.00	135.00	88.00
71	是	否	是	女	112.00	70.00	120.00	81.00
67	否	否	是	女	122.00	70.00	110.00	65.00
67	否	否	是	女	115.00	60.00	121.00	72.00
70	是	是	是	女	120.00	80.00	128.00	82.00
88	否	否	是	女	118.00	60.00	120.00	60.00
77	否	否	是	女	140.00	60.00	148.00	60.00
66	否	否	是	女	126.00	89.00	120.00	80.00
73	是	是	是	女	154.00	76.00	150.00	70.00
68	否	否	是	女	114.00	68.00	126.00	80.00

68	是	否	是	女	136.00	88.00	140.00	80.00
66	否	否	是	女	140.00	80.00	136.00	78.00
81	否	否	是	女	120.00	58.00	122.00	60.00
88	否	否	是	女	123.00	69.00	125.00	70.00
78	否	否	是	女	118.00	70.00	120.00	72.00
71	否	否	是	女	147.00	75.00	148.00	70.00
68	否	否	是	女	150.00	80.00	166.00	76.00
69	是	否	是	女	129.00	80.00	134.00	84.00
67	是	是	是	女	130.00	76.00	132.00	79.00
73	否	否	是	女	109.00	65.00	110.00	70.00
79	是	否	是	女	132.00	76.00	134.00	80.00
71	否	否	是	女	120.00	80.00	124.00	82.00
71	否	否	是	女	130.00	80.00	138.00	86.00
70	是	否	是	女	125.00	74.00	130.00	78.00
84	是	否	是	女	146.00	82.00	150.00	80.00
67	否	否	是	女	119.00	70.00	118.00	82.00
70	是	否	是	女	110.00	80.00	112.00	81.00
75	是	是	是	女	130.00	80.00	134.00	84.00
78	是	是	是	女	148.00	86.00	150.00	80.00
70	否	是	是	女	177.00	101.00	152.00	95.00
95	是	是	是	女	128.00	80.00	130.00	82.00
67	否	否	是	女	140.00	91.00	139.00	89.00
90	是	否	是	女	142.00	75.00	145.00	76.00
69	是	否	是	女	140.00	80.00	154.00	79.00
71	是	否	是	女	130.00	80.00	120.00	80.00
84	否	否	是	女	118.00	66.00	119.00	71.00
68	是	否	是	女	122.00	70.00	120.00	70.00
67	是	是	是	女	147.00	70.00	144.00	72.00
68	否	否	是	女	125.00	75.00	130.00	80.00
71	否	否	是	女	127.00	85.00	130.00	90.00
68	是	否	是	女	138.00	82.00	140.00	85.00
68	否	否	是	女	136.00	76.00	138.00	78.00
67	是	否	是	女	150.00	100.00	153.00	100.00
72	是	否	是	女	147.00	68.00	141.00	70.00
77	是	否	是	女	120.00	70.00	120.00	80.00
68	是	否	是	女	128.00	70.00	133.00	72.00
74	是	是	是	女	138.00	78.00	140.00	80.00
71	是	是	是	女	120.00	68.00	125.00	70.00
70	是	否	是	女	154.00	80.00	152.00	80.00
75	是	否	是	女	177.00	76.00	181.00	132.00
66	是	否	是	女	140.00	87.00	138.00	80.00
81	是	否	是	女	119.00	74.00	116.00	70.00
71	否	否	是	女	128.00	78.00	132.00	76.00
74	是	否	是	女	152.00	74.00	150.00	72.00
75	是	否	是	女	136.00	87.00	144.00	88.00
71	是	否	是	女	146.00	93.00	144.00	88.00
73	否	是	是	女	135.00	70.00	148.00	75.00
75	否	否	是	女	138.00	62.00	142.00	70.00
74	否	否	是	女	126.00	74.00	127.00	77.00
76	否	否	是	女	135.00	83.00	138.00	86.00
90	否	否	是	女	140.00	80.00	148.00	86.00

71	否	否	是	女	150.00	100.00	160.00	99.00
73	是	否	是	女	125.00	82.00	127.00	83.00
69	是	否	是	女	151.00	78.00	148.00	80.00
75	否	否	是	女	115.00	67.00	119.00	72.00
76	是	是	是	女	150.00	70.00	157.00	66.00
70	否	否	是	女	136.00	70.00	138.00	70.00
71	否	否	是	女	160.00	88.00	165.00	88.00
67	否	否	是	女	120.00	78.00	122.00	80.00
81	是	否	是	女	143.00	85.00	137.00	81.00
78	否	否	是	女	126.00	80.00	130.00	85.00
68	是	否	是	女	128.00	68.00	130.00	70.00
73	是	否	是	女	163.00	79.00	145.00	85.00
88	否	否	是	女	115.00	72.00	110.00	78.00
67	否	否	是	女	130.00	88.00	140.00	90.00
67	是	否	是	女	150.00	96.00	146.00	90.00
67	否	否	是	女	110.00	70.00	112.00	72.00
67	是	否	是	女	130.00	80.00	133.00	85.00
71	是	是	是	女	158.00	78.00	160.00	80.00
73	是	否	是	女	130.00	70.00	136.00	83.00
77	是	是	是	女	120.00	75.00	122.00	73.00
75	否	否	是	女	135.00	74.00	130.00	72.00
71	否	否	是	女	130.00	80.00	123.00	72.00
66	否	否	是	女	102.00	70.00	106.00	73.00
78	否	否	是	女	156.00	86.00	160.00	90.00
75	是	否	是	女	146.00	88.00	166.00	94.00
85	是	否	是	女	149.00	82.00	148.00	80.00
71	否	否	是	女	138.00	78.00	136.00	80.00
74	是	是	是	女	150.00	72.00	154.00	78.00
81	是	否	是	女	152.00	75.00	170.00	82.00
84	是	否	是	女	120.00	78.00	130.00	76.00
74	是	否	是	女	129.00	78.00	130.00	80.00
70	否	否	是	女	134.00	70.00	140.00	75.00
77	是	否	是	女	128.00	76.00	130.00	78.00
77	是	否	是	女	136.00	67.00	138.00	70.00
67	是	否	是	女	145.00	87.00	148.00	89.00
72	是	否	是	女	136.00	76.00	142.00	80.00
73	否	否	是	女	136.00	78.00	138.00	80.00
84	是	否	是	女	148.00	82.00	145.00	78.00
71	是	否	是	女	138.00	80.00	130.00	80.00
66	是	否	是	女	120.00	70.00	118.00	66.00
66	是	否	是	女	154.00	84.00	142.00	76.00
69	否	否	是	女	120.00	86.00	122.00	85.00
74	是	是	是	女	147.00	82.00	145.00	80.00
66	是	是	是	女	135.00	75.00	138.00	80.00
76	是	是	是	女	128.00	76.00	130.00	79.00
73	是	否	是	女	120.00	70.00	126.00	79.00
87	是	否	是	女	105.00	62.00	112.00	69.00
73	否	否	是	女	110.00	60.00	115.00	65.00
78	是	否	是	女	140.00	80.00	148.00	86.00
69	是	否	是	女	150.00	90.00	155.00	92.00
84	是	是	是	女	126.00	70.00	132.00	74.00

82	是	是	是	女	117.00	67.00	120.00	70.00
78	否	否	是	女	170.00	80.00	170.00	85.00
81	是	是	是	女	128.00	70.00	130.00	70.00
69	是	否	是	女	150.00	70.00	152.00	68.00
68	是	否	是	女	145.00	90.00	140.00	90.00
70	否	否	是	女	130.00	78.00	128.00	76.00
72	是	否	是	女	130.00	70.00	138.00	76.00
66	否	否	否	女	114.00	77.00	115.00	77.00
68	是	是	是	女	118.00	70.00	120.00	80.00
66	否	否	是	女	145.00	93.00	164.00	93.00
66	是	否	是	女	139.00	90.00	143.00	93.00
67	否	否	是	女	130.00	82.00	132.00	86.00
67	否	否	是	女	120.00	75.00	125.00	80.00
68	是	否	是	女	150.00	90.00	148.00	90.00
79	是	否	是	女	124.00	76.00	126.00	78.00
82	是	是	是	女	133.00	85.00	129.00	74.00
74	是	否	是	女	108.00	68.00	110.00	70.00
69	是	否	是	女	119.00	69.00	122.00	82.00
86	否	否	是	女	132.00	72.00	136.00	75.00
76	否	否	是	女	127.00	78.00	120.00	80.00
74	是	否	是	女	140.00	80.00	150.00	81.00
94	是	否	是	女	145.00	78.00	154.00	79.00
72	是	否	是	女	134.00	89.00	136.00	90.00
73	是	否	是	女	121.00	77.00	118.00	74.00
66	是	否	是	女	140.00	78.00	142.00	85.00
78	否	否	是	女	138.00	68.00	135.00	63.00
77	是	否	是	女	138.00	75.00	147.00	79.00
80	是	否	是	女	128.00	72.00	130.00	70.00
92	否	否	是	女	96.00	60.00	110.00	60.00
96	是	否	是	女	144.00	60.00	140.00	60.00
73	否	否	是	女	116.00	71.00	120.00	80.00
67	否	否	是	女	178.00	97.00	180.00	100.00
76	是	是	是	女	152.00	78.00	158.00	80.00
70	否	否	是	女	139.00	78.00	139.00	82.00
75	否	否	是	女	136.00	80.00	130.00	80.00
70	是	是	是	女	125.00	60.00	120.00	60.00
67	否	否	是	女	124.00	67.00	126.00	69.00
74	是	是	是	女	142.00	81.00	140.00	80.00
78	是	否	是	女	156.00	87.00	163.00	85.00
73	否	是	是	女	98.00	60.00	100.00	61.00
77	是	否	是	女	159.00	94.00	160.00	97.00
75	否	否	是	女	145.00	65.00	150.00	70.00
83	是	是	是	女	153.00	76.00	154.00	72.00
76	是	是	是	女	138.00	79.00	139.00	80.00
66	否	否	是	女	126.00	70.00	124.00	70.00
71	是	否	是	女	110.00	62.00	114.00	60.00
72	是	否	是	女	152.00	82.00	156.00	84.00
70	否	否	是	女	108.00	62.00	100.00	70.00
83	是	否	是	女	140.00	80.00	145.00	82.00
73	否	否	是	女	110.00	70.00	105.00	74.00
68	否	否	是	女	138.00	70.00	136.00	70.00

68	是	否	是	女	141.00	89.00	140.00	90.00
66	是	是	是	女	135.00	85.00	130.00	80.00
68	否	否	是	女	140.00	80.00	140.00	85.00
72	否	否	是	女	128.00	69.00	130.00	70.00
79	是	否	是	女	119.00	75.00	127.00	87.00
75	否	否	是	女	130.00	60.00	125.00	60.00
68	否	否	是	女	120.00	80.00	117.00	74.00
72	是	否	是	女	168.00	100.00	170.00	110.00
77	是	否	是	女	135.00	80.00	138.00	85.00
66	是	是	是	女	115.00	70.00	117.00	72.00
75	否	否	是	女	130.00	70.00	128.00	68.00
75	是	否	是	女	157.00	82.00	166.00	79.00
71	是	是	是	女	120.00	64.00	124.00	70.00
73	是	否	是	女	110.00	72.00	110.00	75.00
81	否	否	是	女	135.00	80.00	130.00	80.00
71	否	否	是	女	162.00	98.00	165.00	98.00
70	是	否	是	女	130.00	80.00	132.00	82.00
68	否	否	是	女	121.00	58.00	115.00	63.00
73	是	否	是	女	152.00	80.00	138.00	80.00
76	是	否	是	女	135.00	85.00	138.00	85.00
74	是	否	是	女	139.00	89.00	135.00	80.00
76	是	否	是	女	128.00	62.00	130.00	64.00
80	是	否	是	女	150.00	90.00	151.00	85.00
73	否	否	是	女	139.00	80.00	136.00	76.00
67	否	否	是	女	148.00	90.00	150.00	92.00
77	是	否	是	女	122.00	80.00	124.00	88.00
75	否	否	是	女	125.00	73.00	127.00	76.00
75	是	否	是	女	114.00	80.00	137.00	82.00
74	是	否	是	女	139.00	84.00	136.00	85.00
82	否	是	是	女	130.00	80.00	135.00	86.00
75	否	否	是	女	112.00	62.00	116.00	66.00
66	否	否	是	女	130.00	74.00	128.00	72.00
76	是	是	是	女	149.00	69.00	149.00	65.00
74	是	否	是	女	139.00	68.00	148.00	71.00
70	否	否	是	女	146.00	80.00	147.00	89.00
72	是	否	是	女	129.00	76.00	133.00	79.00
73	否	是	是	女	160.00	105.00	155.00	103.00
66	否	否	是	女	112.00	70.00	115.00	79.00
67	是	否	是	女	134.00	82.00	123.00	80.00
72	否	否	是	女	128.00	77.00	145.00	86.00
67	是	否	是	女	130.00	80.00	130.00	84.00
66	否	否	是	女	136.00	76.00	124.00	68.00
70	是	是	是	女	146.00	74.00	148.00	82.00
79	否	否	是	女	130.00	80.00	120.00	80.00
71	是	否	是	女	133.00	87.00	140.00	89.00
80	是	否	是	女	140.00	82.00	145.00	77.00
66	否	否	否	女	110.00	70.00	112.00	72.00
74	否	否	是	女	180.00	95.00	180.00	100.00
71	否	否	是	女	152.00	76.00	157.00	77.00
76	是	否	是	女	145.00	70.00	140.00	70.00
66	否	否	是	女	126.00	86.00	122.00	84.00

69	否	否	是	女	117.00	75.00	125.00	71.00
72	否	是	是	女	130.00	90.00	132.00	92.00
72	否	否	是	女	132.00	78.00	138.00	80.00
66	是	是	是	女	106.00	65.00	110.00	70.00
97	是	否	是	女	132.00	61.00	135.00	65.00
69	是	否	是	女	140.00	70.00	130.00	68.00
71	否	否	是	女	96.00	60.00	98.00	60.00
72	是	是	是	女	127.00	78.00	139.00	80.00
72	是	否	是	女	134.00	76.00	136.00	78.00
87	否	否	是	女	137.00	67.00	130.00	67.00
77	是	否	是	女	125.00	71.00	129.00	76.00
67	是	是	是	女	144.00	85.00	143.00	81.00
79	否	否	是	女	124.00	76.00	136.00	79.00
71	否	否	是	女	120.00	75.00	124.00	75.00
82	是	是	是	女	120.00	76.00	124.00	70.00
75	否	否	是	女	152.00	93.00	150.00	89.00
72	是	是	是	女	150.00	80.00	154.00	90.00
67	是	否	是	女	130.00	80.00	135.00	82.00
75	否	否	是	女	120.00	70.00	116.00	75.00
71	是	是	是	女	130.00	84.00	132.00	82.00
76	否	否	是	女	122.00	62.00	128.00	64.00
75	否	否	是	女	128.00	70.00	139.00	78.00
70	否	否	是	女	94.00	62.00	96.00	66.00
70	是	否	是	女	140.00	70.00	135.00	70.00
73	否	否	是	女	120.00	80.00	130.00	70.00
67	否	是	是	女	110.00	60.00	114.00	62.00
69	是	否	是	女	220.00	116.00	244.00	120.00
73	否	否	是	女	140.00	90.00	140.00	80.00
68	是	是	是	女	130.00	70.00	136.00	73.00
67	否	否	是	女	110.00	70.00	100.00	60.00
74	是	是	是	女	120.00	70.00	110.00	70.00
66	否	否	是	女	162.00	94.00	166.00	96.00
88	否	否	是	女	130.00	65.00	128.00	65.00
70	否	否	是	女	110.00	70.00	120.00	60.00
70	否	是	是	女	138.00	68.00	140.00	70.00
73	否	是	是	女	112.00	77.00	124.00	84.00
66	否	否	是	女	109.00	77.00	112.00	80.00
69	是	否	是	女	149.00	83.00	147.00	93.00
77	是	否	是	女	150.00	80.00	158.00	92.00
76	是	否	是	女	140.00	75.00	142.00	81.00
76	是	是	是	女	155.00	90.00	160.00	94.00
77	否	否	是	女	120.00	70.00	120.00	76.00
76	是	是	是	女	118.00	70.00	120.00	72.00
69	否	否	是	女	95.00	60.00	94.00	54.00
76	否	否	是	女	136.00	72.00	138.00	70.00
68	否	否	是	女	120.00	70.00	115.00	70.00
67	否	否	是	女	124.00	70.00	124.00	84.00
68	是	是	是	女	146.00	80.00	148.00	84.00
70	否	否	是	女	120.00	80.00	115.00	80.00
72	是	否	是	女	138.00	70.00	140.00	78.00
76	是	否	是	女	138.00	78.00	139.00	80.00

66	否	否	是	女	126.00	74.00	124.00	74.00
74	是	否	是	女	145.00	98.00	155.00	91.00
75	是	是	是	女	121.00	89.00	128.00	82.00
67	是	否	是	女	130.00	78.00	130.00	80.00
84	否	否	是	女	110.00	70.00	120.00	82.00
71	是	否	是	女	114.00	70.00	120.00	76.00
74	否	否	是	女	108.00	68.00	110.00	70.00
73	是	否	是	女	115.00	84.00	120.00	88.00
67	是	是	是	女	130.00	70.00	134.00	79.00
76	是	否	是	女	202.00	96.00	193.00	93.00
83	是	否	是	女	150.00	70.00	152.00	72.00
69	是	是	是	女	110.00	70.00	115.00	70.00
66	是	否	是	女	140.00	94.00	154.00	98.00
78	是	否	是	女	116.00	78.00	120.00	80.00
70	是	否	是	女	120.00	70.00	128.00	76.00
68	是	否	是	女	138.00	78.00	134.00	76.00
70	否	否	是	女	118.00	70.00	120.00	78.00
66	是	否	是	女	125.00	72.00	130.00	80.00
71	是	否	是	女	150.00	80.00	158.00	86.00
83	是	否	是	女	146.00	76.00	144.00	73.00
66	是	否	否	女	130.00	86.00	133.00	89.00
75	是	否	是	女	158.00	82.00	164.00	80.00
68	是	否	是	女	131.00	80.00	135.00	83.00
73	否	否	是	女	138.00	80.00	140.00	80.00
66	否	否	是	女	130.00	80.00	137.00	81.00
69	否	否	是	女	125.00	65.00	130.00	70.00
69	是	否	是	女	132.00	82.00	130.00	86.00
66	是	否	是	女	130.00	80.00	147.00	94.00
70	否	否	是	女	115.00	75.00	120.00	80.00
87	是	否	是	女	120.00	74.00	130.00	76.00
75	是	否	是	女	115.00	62.00	118.00	61.00
69	否	否	是	女	118.00	78.00	120.00	74.00
68	是	否	是	女	120.00	72.00	118.00	70.00
76	是	否	是	女	128.00	68.00	130.00	70.00
70	是	否	是	女	160.00	80.00	160.00	70.00
74	否	是	是	女	149.00	81.00	156.00	90.00
66	是	否	是	女	141.00	88.00	138.00	86.00
70	是	是	是	女	130.00	82.00	138.00	86.00
66	否	否	是	女	120.00	60.00	115.00	60.00
73	否	否	是	女	120.00	80.00	110.00	76.00
72	否	否	是	女	130.00	66.00	139.00	71.00
67	否	否	是	女	130.00	69.00	131.00	70.00
94	否	否	是	女	136.00	88.00	134.00	82.00
71	是	否	是	女	133.00	71.00	134.00	70.00
79	是	是	是	女	130.00	82.00	140.00	90.00
70	是	否	是	女	126.00	78.00	124.00	80.00
67	否	否	是	女	145.00	90.00	147.00	89.00
67	否	否	是	女	130.00	81.00	126.00	76.00
72	是	是	是	女	160.00	90.00	162.00	92.00
68	否	否	是	女	120.00	70.00	130.00	68.00
87	否	否	是	女	116.00	70.00	120.00	70.00

76	否	否	是	女	140.00	80.00	135.00	76.00
73	否	是	是	女	106.00	74.00	119.00	72.00
67	是	是	是	女	126.00	80.00	128.00	80.00
70	否	否	是	女	120.00	60.00	114.00	67.00
73	是	否	是	女	128.00	78.00	131.00	86.00
73	否	否	是	女	138.00	76.00	140.00	80.00
68	是	否	是	女	130.00	70.00	130.00	80.00
71	否	否	是	女	137.00	82.00	132.00	76.00
77	是	是	是	女	162.00	84.00	163.00	81.00
66	是	否	是	女	136.00	78.00	138.00	80.00
66	是	是	否	女	210.00	90.00	220.00	110.00
67	是	否	是	女	130.00	86.00	128.00	70.00
69	否	否	是	女	125.00	80.00	120.00	75.00
75	是	否	是	女	140.00	80.00	145.00	86.00
82	是	否	是	女	128.00	78.00	130.00	80.00
68	是	否	是	女	140.00	76.00	140.00	79.00
68	否	否	是	女	125.00	99.00	120.00	90.00
70	是	是	是	女	130.00	80.00	132.00	79.00
67	否	是	是	女	110.00	68.00	118.00	78.00
68	是	否	是	女	142.00	100.00	150.00	100.00
68	否	否	是	女	115.00	72.00	118.00	76.00
76	是	是	是	女	105.00	65.00	110.00	70.00
70	否	否	是	女	117.00	70.00	110.00	68.00
66	是	是	是	女	120.00	78.00	130.00	86.00
76	是	是	是	女	132.00	62.00	139.00	65.00
70	否	否	是	女	108.00	73.00	110.00	80.00
71	是	否	是	女	130.00	70.00	135.00	80.00
71	否	否	是	女	128.00	76.00	129.00	79.00
78	是	否	是	女	80.00	40.00	82.00	46.00
69	是	否	是	女	128.00	78.00	130.00	80.00
76	否	是	是	女	120.00	80.00	125.00	75.00
68	是	否	是	女	130.00	80.00	129.00	78.00
67	是	是	是	女	116.00	75.00	117.00	76.00
76	是	是	是	女	155.00	80.00	160.00	85.00
72	是	否	是	女	145.00	82.00	158.00	88.00
86	否	否	是	女	125.00	80.00	131.00	70.00
82	否	否	是	女	106.00	66.00	110.00	70.00
67	否	否	是	女	120.00	80.00	117.00	80.00
69	否	否	是	女	118.00	84.00	120.00	87.00
70	否	否	是	女	135.00	80.00	149.00	89.00
71	是	否	是	女	135.00	78.00	136.00	79.00
77	是	是	是	女	135.00	74.00	139.00	77.00
76	否	否	是	女	134.00	78.00	136.00	70.00
66	是	否	是	女	110.00	70.00	112.00	72.00
67	否	否	是	女	138.00	90.00	135.00	89.00
72	否	否	是	女	116.00	68.00	120.00	70.00
69	否	否	是	女	138.00	86.00	146.00	89.00
69	否	否	是	女	102.00	69.00	124.00	80.00
81	是	否	是	女	120.00	70.00	125.00	78.00
76	是	是	是	女	160.00	78.00	158.00	80.00
66	是	是	是	女	148.00	83.00	145.00	80.00

91	是	否	是	女	135.00	80.00	138.00	82.00
81	否	是	是	女	118.00	70.00	120.00	72.00
70	是	否	是	女	148.00	90.00	150.00	92.00
66	否	否	是	女	144.00	80.00	140.00	74.00
67	否	否	是	女	120.00	70.00	126.00	69.00
74	是	否	是	女	116.00	85.00	118.00	86.00
94	是	是	是	女	150.00	90.00	152.00	92.00
78	否	否	是	女	134.00	66.00	138.00	70.00
69	是	否	是	女	138.00	80.00	140.00	80.00
74	是	是	是	女	157.00	84.00	159.00	88.00
72	是	否	是	女	129.00	86.00	122.00	78.00
72	否	否	是	女	130.00	80.00	135.00	85.00
91	否	否	是	女	126.00	70.00	122.00	76.00
70	否	否	是	女	118.00	85.00	119.00	87.00
73	是	是	是	女	120.00	80.00	122.00	82.00
68	是	否	是	女	120.00	70.00	120.00	80.00
66	否	否	否	女	118.00	62.00	116.00	60.00
73	是	否	是	女	130.00	80.00	134.00	78.00
69	否	否	是	女	136.00	90.00	133.00	84.00
71	是	否	是	女	114.00	70.00	120.00	75.00
66	否	否	是	女	105.00	75.00	110.00	80.00
76	否	否	是	女	138.00	90.00	136.00	82.00
85	否	是	是	女	146.00	80.00	145.00	79.00
86	否	否	是	女	130.00	70.00	128.00	78.00
69	是	否	是	女	112.00	66.00	110.00	68.00
77	否	否	是	女	110.00	88.00	130.00	85.00
68	是	否	是	女	110.00	68.00	112.00	66.00
72	是	否	是	女	138.00	86.00	139.00	87.00
70	是	是	是	女	136.00	70.00	138.00	60.00
76	是	否	是	女	100.00	60.00	102.00	64.00
67	否	否	是	女	120.00	75.00	122.00	78.00
80	是	否	是	女	130.00	76.00	130.00	78.00
75	是	是	是	女	169.00	78.00	164.00	67.00
97	是	否	是	女	130.00	80.00	132.00	82.00
79	是	否	是	女	150.00	80.00	169.00	84.00
75	是	否	是	女	110.00	70.00	120.00	73.00
85	是	否	是	女	140.00	80.00	140.00	90.00
85	是	是	是	女	112.00	70.00	120.00	78.00
78	是	否	是	女	140.00	80.00	152.00	92.00
67	是	是	是	女	135.00	78.00	124.00	79.00
69	否	否	是	女	108.00	81.00	105.00	75.00
76	是	否	是	女	146.00	80.00	138.00	78.00
67	否	否	是	女	130.00	76.00	136.00	88.00
77	是	否	是	女	152.00	90.00	155.00	98.00
74	否	否	是	女	175.00	95.00	169.00	89.00
95	是	否	是	女	160.00	90.00	162.00	90.00
71	是	否	是	女	167.00	99.00	170.00	102.00
72	是	否	是	女	136.00	86.00	138.00	88.00
72	否	否	是	女	154.00	84.00	152.00	83.00
66	是	否	是	女	138.00	70.00	140.00	78.00
66	是	否	是	女	170.00	100.00	161.00	94.00

75	是	否	是	女	112.00	70.00	120.00	80.00
76	是	否	是	女	170.00	80.00	168.00	82.00
68	是	是	是	女	130.00	80.00	130.00	82.00
75	是	是	是	女	130.00	78.00	132.00	80.00
68	是	否	是	女	160.00	80.00	160.00	80.00
86	是	否	是	女	101.00	63.00	99.00	61.00
69	是	否	是	女	137.00	77.00	137.00	79.00
67	否	否	是	女	145.00	70.00	147.00	77.00
68	否	否	是	女	134.00	68.00	135.00	70.00
69	否	是	是	女	130.00	76.00	124.00	76.00
66	是	否	是	女	120.00	80.00	124.00	80.00
67	否	否	是	女	122.00	80.00	128.00	82.00
69	否	否	是	女	110.00	70.00	120.00	80.00
76	是	否	是	女	138.00	60.00	139.00	88.00
76	否	否	是	女	176.00	76.00	172.00	83.00
78	否	否	是	女	125.00	80.00	130.00	80.00
86	是	是	是	女	128.00	66.00	130.00	69.00
72	否	否	是	女	140.00	65.00	146.00	70.00
72	是	否	是	女	150.00	88.00	152.00	90.00
89	是	否	是	女	160.00	98.00	162.00	100.00
70	否	否	是	女	122.00	70.00	126.00	72.00
66	是	是	是	女	154.00	93.00	176.00	100.00
67	是	否	是	女	142.00	83.00	130.00	85.00
69	否	是	是	女	130.00	80.00	135.00	85.00
79	是	否	是	女	155.00	86.00	150.00	80.00
71	否	是	是	女	120.00	70.00	110.00	70.00
71	否	否	是	女	150.00	90.00	152.00	90.00
69	否	是	是	女	145.00	78.00	146.00	80.00
74	否	否	是	女	120.00	60.00	123.00	60.00
73	是	否	是	女	140.00	76.00	138.00	80.00
67	是	否	是	女	133.00	93.00	140.00	91.00
67	否	否	是	女	136.00	80.00	138.00	82.00
87	是	否	是	女	150.00	70.00	170.00	74.00
70	否	否	是	女	135.00	79.00	133.00	78.00
66	否	否	是	女	136.00	88.00	130.00	85.00
67	是	否	是	女	160.00	90.00	176.00	92.00
66	是	否	否	女	140.00	82.00	140.00	84.00
67	否	否	是	女	164.00	90.00	170.00	90.00
85	否	否	是	女	156.00	90.00	161.00	90.00
80	是	否	是	女	145.00	85.00	149.00	88.00
72	是	否	是	女	140.00	80.00	130.00	70.00
71	是	是	是	女	136.00	82.00	138.00	88.00
76	否	否	是	女	139.00	86.00	140.00	90.00
67	否	否	是	女	110.00	80.00	126.00	75.00
79	否	否	是	女	153.00	83.00	160.00	99.00
80	是	否	是	女	110.00	72.00	118.00	74.00
67	否	否	是	女	108.00	76.00	110.00	80.00
78	否	否	是	女	163.00	88.00	165.00	89.00
73	是	否	是	女	148.00	80.00	144.00	78.00
83	否	否	是	女	128.00	72.00	130.00	74.00
77	是	否	是	女	160.00	95.00	159.00	96.00

76	否	否	是	女	128.00	86.00	130.00	88.00
71	是	否	是	女	129.00	78.00	134.00	82.00
70	否	否	是	女	110.00	68.00	128.00	66.00
85	是	是	是	女	124.00	82.00	130.00	80.00
76	是	是	是	女	116.00	74.00	119.00	77.00
69	否	否	是	女	170.00	94.00	174.00	96.00
77	是	否	是	女	120.00	66.00	126.00	68.00
72	是	否	是	女	115.00	76.00	118.00	80.00
70	否	否	是	女	110.00	78.00	110.00	80.00
71	是	否	是	女	150.00	66.00	152.00	66.00
79	是	是	是	女	131.00	88.00	143.00	88.00
66	否	否	是	女	134.00	80.00	140.00	86.00
74	否	是	是	女	154.00	92.00	160.00	100.00
79	是	否	是	女	115.00	70.00	120.00	76.00
66	否	否	是	女	138.00	70.00	140.00	65.00
71	是	否	是	女	162.00	101.00	170.00	105.00
80	是	否	是	女	128.00	80.00	138.00	83.00
79	否	否	是	女	110.00	70.00	108.00	68.00
77	是	否	是	女	132.00	60.00	126.00	64.00
80	否	否	是	女	104.00	70.00	106.00	70.00
71	否	否	是	女	136.00	70.00	138.00	72.00
77	是	否	是	女	108.00	72.00	110.00	70.00
76	是	否	是	女	136.00	82.00	136.00	80.00
69	是	否	是	女	168.00	88.00	170.00	80.00
74	是	是	是	女	160.00	80.00	165.00	90.00
85	否	否	是	女	139.00	89.00	140.00	90.00
75	是	否	是	女	128.00	84.00	130.00	80.00
66	是	否	是	女	132.00	78.00	136.00	80.00
70	是	否	是	女	143.00	88.00	141.00	88.00
85	否	否	是	女	117.00	76.00	126.00	82.00
71	是	否	是	女	158.00	96.00	158.00	94.00
75	是	否	是	女	155.00	65.00	160.00	70.00
75	是	否	是	女	135.00	84.00	138.00	88.00
72	否	否	是	女	136.00	80.00	140.00	80.00
75	是	否	是	女	154.00	89.00	158.00	88.00
76	否	否	是	女	140.00	80.00	145.00	86.00
71	是	是	是	女	133.00	84.00	136.00	86.00
77	否	否	是	女	136.00	70.00	137.00	86.00
79	是	否	是	女	135.00	70.00	145.00	80.00
80	是	否	是	女	146.00	89.00	138.00	88.00
67	是	否	是	女	130.00	86.00	137.00	79.00
72	是	否	是	女	130.00	80.00	138.00	86.00
83	是	否	是	女	130.00	65.00	136.00	70.00
74	否	否	是	女	148.00	78.00	144.00	76.00
73	是	否	是	女	125.00	90.00	127.00	93.00
69	是	否	是	女	115.00	79.00	110.00	75.00
81	否	否	是	女	100.00	60.00	102.00	60.00
67	是	否	是	女	136.00	80.00	140.00	88.00
70	否	是	是	女	100.00	60.00	94.00	65.00
79	否	否	是	女	144.00	86.00	146.00	88.00
68	是	否	是	女	141.00	66.00	158.00	65.00

68	是	否	是	女	120.00	74.00	124.00	74.00
69	是	否	是	女	130.00	70.00	139.00	80.00
81	是	否	是	女	130.00	70.00	133.00	68.00
69	是	是	是	女	156.00	77.00	147.00	78.00
69	是	否	是	女	138.00	80.00	138.00	82.00
69	是	是	是	女	134.00	80.00	140.00	86.00
69	是	是	是	女	150.00	80.00	154.00	80.00
94	否	是	是	女	110.00	70.00	120.00	80.00
72	是	是	是	女	139.00	85.00	137.00	81.00
66	否	否	是	女	90.00	60.00	92.00	61.00
67	否	否	是	女	150.00	80.00	155.00	85.00
73	是	否	是	女	135.00	70.00	140.00	70.00
79	是	否	是	女	176.00	91.00	152.00	80.00
74	否	否	是	女	132.00	80.00	130.00	80.00
85	是	否	是	女	130.00	76.00	126.00	70.00
70	否	否	是	女	130.00	80.00	125.00	70.00
68	是	是	是	女	128.00	88.00	130.00	90.00
69	否	否	是	女	130.00	80.00	122.00	78.00
76	否	否	是	女	180.00	94.00	182.00	96.00
84	是	是	是	女	120.00	90.00	118.00	88.00
69	是	否	是	女	132.00	80.00	135.00	85.00
68	否	否	是	女	112.00	72.00	110.00	70.00
67	否	否	是	女	130.00	88.00	138.00	89.00
67	否	否	是	女	120.00	70.00	124.00	70.00
80	是	是	是	女	135.00	82.00	137.00	83.00
66	是	否	是	女	163.00	88.00	175.00	97.00
71	是	是	是	女	138.00	80.00	136.00	80.00
69	是	否	是	女	125.00	75.00	126.00	77.00
82	是	是	是	女	136.00	70.00	140.00	70.00
68	是	否	是	女	130.00	80.00	120.00	75.00
73	是	否	是	女	122.00	68.00	130.00	70.00
72	是	否	是	女	148.00	91.00	157.00	91.00
69	是	否	是	女	105.00	71.00	100.00	70.00
72	否	否	是	女	130.00	68.00	133.00	72.00
86	是	否	是	女	170.00	100.00	180.00	100.00
73	是	是	是	女	152.00	92.00	154.00	98.00
69	是	否	是	女	136.00	70.00	140.00	72.00
71	是	是	是	女	126.00	70.00	128.00	72.00
75	否	否	是	女	120.00	80.00	125.00	76.00
77	是	否	是	女	130.00	78.00	132.00	76.00
98	否	否	是	女	136.00	86.00	132.00	80.00
66	否	否	是	女	180.00	80.00	185.00	79.00
68	是	否	是	女	132.00	86.00	130.00	80.00
70	是	否	是	女	150.00	92.00	152.00	90.00
76	否	否	是	女	138.00	80.00	130.00	75.00
75	是	否	是	女	130.00	70.00	128.00	66.00
75	是	否	是	女	135.00	82.00	138.00	79.00
67	否	否	是	女	150.00	74.00	155.00	80.00
71	否	否	是	女	138.00	80.00	160.00	100.00
70	是	否	是	女	110.00	65.00	118.00	66.00
90	是	否	是	女	150.00	90.00	155.00	96.00

69	是	否	是	女	135.00	75.00	139.00	88.00
67	否	否	是	女	122.00	80.00	120.00	78.00
74	否	否	是	女	106.00	60.00	107.00	57.00
70	是	是	是	女	138.00	80.00	140.00	76.00
66	否	否	是	女	132.00	75.00	137.00	79.00
80	是	否	是	女	170.00	90.00	180.00	100.00
78	是	否	是	女	150.00	80.00	152.00	81.00
67	是	否	是	女	132.00	84.00	142.00	88.00
77	否	否	是	女	100.00	60.00	104.00	62.00
70	是	否	是	女	136.00	90.00	140.00	90.00
71	否	否	是	女	142.00	85.00	148.00	90.00
95	是	是	是	女	132.00	70.00	136.00	74.00
73	是	是	是	女	140.00	98.00	150.00	100.00
88	是	是	是	女	134.00	63.00	129.00	85.00
86	是	否	是	女	159.00	86.00	160.00	86.00
88	是	是	是	女	129.00	80.00	130.00	85.00
66	是	否	是	女	118.00	78.00	120.00	80.00
73	否	否	是	女	123.00	69.00	131.00	75.00
76	是	否	是	女	110.00	66.00	108.00	64.00
72	是	否	是	女	170.00	98.00	172.00	100.00
74	是	否	是	女	130.00	80.00	125.00	80.00
69	否	否	是	女	123.00	80.00	123.00	82.00
74	否	否	是	女	137.00	91.00	142.00	94.00
71	是	否	是	女	140.00	90.00	150.00	96.00
68	是	否	是	女	142.00	80.00	140.00	80.00
71	否	否	是	女	138.00	82.00	138.00	86.00
83	是	否	是	女	120.00	70.00	128.00	74.00
81	是	否	是	女	135.00	75.00	130.00	70.00
73	否	是	是	女	130.00	82.00	129.00	80.00
78	否	否	是	女	100.00	68.00	108.00	68.00
68	否	否	是	女	108.00	72.00	110.00	74.00
72	是	否	是	女	162.00	78.00	170.00	80.00
86	是	是	是	女	162.00	88.00	163.00	89.00
86	是	否	是	女	130.00	90.00	138.00	90.00
84	是	否	是	女	136.00	80.00	130.00	82.00
72	否	否	是	女	118.00	76.00	126.00	80.00
78	否	否	是	女	128.00	70.00	131.00	76.00
66	是	否	是	女	149.00	88.00	150.00	89.00
75	是	是	是	女	126.00	72.00	126.00	74.00
68	是	否	是	女	139.00	90.00	135.00	88.00
70	是	是	是	女	138.00	77.00	139.00	87.00
75	是	是	是	女	131.00	80.00	120.00	78.00
70	否	否	是	女	118.00	64.00	120.00	70.00
72	是	否	是	女	130.00	72.00	132.00	74.00
70	是	否	是	女	134.00	78.00	140.00	80.00
73	否	否	是	女	121.00	78.00	121.00	81.00
70	否	否	是	女	118.00	70.00	120.00	70.00
72	是	是	是	女	124.00	74.00	130.00	78.00
68	否	否	是	女	140.00	80.00	120.00	70.00
76	是	否	是	女	112.00	80.00	116.00	81.00
93	否	否	是	女	134.00	82.00	134.00	84.00

72	是	是	是	女	139.00	70.00	140.00	76.00
77	否	是	是	女	122.00	70.00	126.00	70.00
74	是	否	是	女	164.00	100.00	130.00	90.00
71	是	否	是	女	150.00	88.00	158.00	90.00
89	否	否	是	女	132.00	60.00	128.00	66.00
75	否	是	是	女	136.00	70.00	140.00	72.00
69	是	否	是	女	139.00	82.00	132.00	80.00
74	是	是	是	女	126.00	60.00	129.00	72.00
66	是	否	是	女	139.00	99.00	135.00	89.00
68	否	否	是	女	139.00	85.00	136.00	84.00
66	否	否	是	女	164.00	97.00	143.00	87.00
68	是	否	是	女	120.00	78.00	124.00	80.00
66	是	是	是	女	112.00	72.00	116.00	74.00
67	是	否	是	女	140.00	80.00	144.00	88.00
77	是	否	是	女	138.00	70.00	140.00	78.00
85	是	否	是	女	139.00	75.00	138.00	89.00
79	否	否	是	女	139.00	86.00	137.00	85.00
71	是	否	是	女	151.00	85.00	162.00	89.00
66	否	否	是	女	124.00	70.00	128.00	70.00
69	是	否	是	女	139.00	76.00	146.00	69.00
83	否	否	是	女	124.00	74.00	126.00	76.00
80	是	否	是	女	138.00	80.00	140.00	82.00
72	是	是	是	女	130.00	74.00	132.00	78.00
77	否	是	是	女	130.00	70.00	130.00	74.00
72	否	否	是	女	110.00	80.00	112.00	85.00
67	否	否	是	女	139.00	89.00	130.00	80.00
73	否	否	是	女	134.00	82.00	139.00	89.00
73	是	否	是	女	118.00	67.00	122.00	74.00
70	是	是	是	女	135.00	76.00	140.00	78.00
73	是	否	是	女	138.00	88.00	138.00	89.00
90	是	否	是	女	150.00	80.00	158.00	89.00
72	否	否	是	女	134.00	86.00	135.00	85.00
76	是	否	是	女	135.00	86.00	137.00	91.00
68	是	否	是	女	125.00	82.00	125.00	74.00
71	是	否	是	女	138.00	80.00	140.00	82.00
67	否	否	是	女	130.00	70.00	120.00	80.00
85	是	否	是	女	134.00	80.00	138.00	80.00
66	是	是	否	女	131.00	86.00	140.00	90.00
72	否	否	是	女	164.00	89.00	170.00	98.00
87	是	否	是	女	159.00	90.00	174.00	85.00
70	是	否	是	女	133.00	80.00	136.00	83.00
70	否	否	是	女	138.00	86.00	139.00	89.00
80	是	否	是	女	150.00	80.00	146.00	76.00
89	是	否	是	女	140.00	82.00	142.00	82.00
77	是	否	是	女	139.00	89.00	135.00	88.00
98	是	否	是	女	108.00	90.00	110.00	86.00
82	是	否	是	女	145.00	89.00	140.00	85.00
70	是	是	是	女	118.00	70.00	120.00	79.00
97	是	否	是	女	132.00	70.00	134.00	74.00
74	是	是	是	女	128.00	65.00	130.00	68.00
67	否	否	是	女	136.00	78.00	139.00	80.00

68	是	否	是	女	136.00	86.00	139.00	89.00
74	否	否	是	女	120.00	70.00	130.00	75.00
87	是	否	是	女	120.00	64.00	124.00	64.00
83	是	否	是	女	170.00	86.00	172.00	85.00
74	是	否	是	女	124.00	65.00	126.00	70.00
74	是	否	是	女	130.00	72.00	132.00	74.00
83	否	否	是	女	120.00	70.00	116.00	65.00
70	是	否	是	女	142.00	94.00	144.00	94.00
67	是	否	是	女	110.00	80.00	114.00	82.00
85	是	否	是	女	109.00	67.00	108.00	68.00
69	否	否	是	女	126.00	78.00	120.00	80.00
75	否	否	是	女	130.00	80.00	120.00	70.00
70	否	否	是	女	145.00	95.00	150.00	90.00
76	是	否	是	女	126.00	83.00	125.00	80.00
73	是	否	是	女	150.00	78.00	155.00	80.00
68	否	否	是	女	139.00	83.00	135.00	82.00
69	是	否	是	女	125.00	75.00	130.00	80.00
77	否	否	是	女	130.00	80.00	134.00	82.00
66	是	否	是	女	134.00	80.00	135.00	82.00
75	否	否	是	女	130.00	80.00	128.00	82.00
75	否	否	是	女	137.00	96.00	138.00	97.00
69	是	否	是	女	132.00	88.00	130.00	78.00
77	是	是	是	女	138.00	78.00	140.00	80.00
74	是	否	是	女	144.00	82.00	133.00	82.00
73	否	否	是	女	140.00	82.00	142.00	80.00
75	是	是	是	女	110.00	60.00	112.00	76.00
95	是	否	是	女	120.00	60.00	122.00	68.00
69	是	是	是	女	135.00	85.00	139.00	89.00
79	否	否	是	女	110.00	78.00	108.00	76.00
76	是	否	是	女	160.00	78.00	162.00	78.00
73	是	否	是	女	138.00	88.00	139.00	85.00
72	是	否	是	女	138.00	80.00	134.00	82.00
87	是	否	是	女	140.00	88.00	142.00	88.00
72	否	否	是	女	142.00	80.00	146.00	90.00
69	否	是	是	女	140.00	89.00	138.00	90.00
76	否	否	是	女	150.00	84.00	150.00	82.00
92	是	是	是	女	110.00	60.00	117.00	63.00
66	是	否	是	女	116.00	70.00	120.00	76.00
68	否	否	是	女	132.00	75.00	135.00	80.00
82	是	否	是	女	128.00	72.00	130.00	80.00
68	否	否	是	女	134.00	93.00	139.00	96.00
72	否	否	是	女	124.00	70.00	126.00	74.00
74	是	否	是	女	145.00	85.00	140.00	82.00
73	是	否	是	女	128.00	76.00	130.00	78.00
71	否	否	是	女	153.00	89.00	163.00	92.00
70	否	否	是	女	124.00	78.00	126.00	80.00
91	是	否	是	女	120.00	70.00	120.00	74.00
71	是	否	是	女	139.00	84.00	136.00	86.00
67	否	否	是	女	110.00	65.00	100.00	65.00
75	是	否	是	女	130.00	70.00	132.00	76.00
74	否	否	是	女	120.00	75.00	125.00	78.00

68	是	否	是	女	140.00	90.00	137.00	89.00
84	否	否	是	女	138.00	58.00	136.00	70.00
78	否	否	是	女	125.00	67.00	130.00	80.00
68	是	否	是	女	110.00	69.00	115.00	72.00
71	否	是	是	女	100.00	61.00	98.00	62.00
73	是	否	是	女	116.00	72.00	114.00	69.00
68	是	否	是	女	156.00	94.00	154.00	94.00
68	是	否	是	女	180.00	100.00	175.00	95.00
70	否	否	是	女	133.00	74.00	133.00	76.00
67	是	否	是	女	118.00	72.00	120.00	74.00
75	是	否	是	女	178.00	88.00	172.00	78.00
67	是	否	是	女	118.00	78.00	120.00	80.00
74	否	否	是	女	115.00	70.00	115.00	76.00
74	否	否	是	女	122.00	74.00	120.00	70.00
70	是	否	是	女	156.00	82.00	160.00	87.00
77	是	是	是	女	136.00	89.00	155.00	85.00
75	否	否	是	女	138.00	90.00	130.00	80.00
71	是	否	是	女	138.00	75.00	140.00	79.00
69	是	否	是	女	150.00	82.00	158.00	80.00
84	是	否	是	女	144.00	79.00	144.00	85.00
84	否	否	是	女	148.00	77.00	168.00	77.00
71	否	否	是	女	110.00	64.00	108.00	70.00
67	是	否	是	女	142.00	92.00	148.00	98.00
74	否	否	是	女	140.00	89.00	145.00	90.00
82	是	否	是	女	110.00	60.00	112.00	68.00
66	是	否	是	女	140.00	88.00	145.00	90.00
67	否	否	是	女	130.00	84.00	133.00	86.00
67	否	是	是	女	132.00	84.00	130.00	80.00
77	否	是	是	女	138.00	80.00	136.00	82.00
66	否	否	是	女	135.00	64.00	138.00	79.00
92	是	否	是	女	148.00	88.00	159.00	89.00
80	否	否	是	女	108.00	60.00	110.00	62.00
90	否	否	是	女	134.00	86.00	136.00	88.00
71	是	否	是	女	149.00	89.00	147.00	87.00
68	是	是	是	女	138.00	63.00	140.00	79.00
70	是	否	是	女	120.00	80.00	121.00	82.00
82	是	是	是	女	133.00	78.00	135.00	80.00
71	否	否	是	女	133.00	85.00	136.00	88.00
72	否	否	是	女	120.00	70.00	122.00	72.00
72	否	否	是	女	135.00	83.00	138.00	86.00
95	是	否	是	女	135.00	85.00	140.00	90.00
67	否	否	是	女	137.00	80.00	140.00	82.00
93	是	否	是	女	112.00	74.00	116.00	78.00
81	否	否	是	女	118.00	78.00	120.00	80.00
74	是	否	是	女	120.00	80.00	118.00	78.00
67	是	否	是	女	142.00	83.00	145.00	85.00
66	否	否	是	女	135.00	78.00	130.00	70.00
92	是	否	是	女	122.00	78.00	120.00	80.00
77	否	是	是	女	120.00	70.00	122.00	84.00
69	是	否	是	女	152.00	70.00	155.00	77.00
77	否	否	是	女	128.00	70.00	130.00	70.00

69	否	否	是	女	102.00	68.00	105.00	70.00
86	否	是	是	女	120.00	70.00	126.00	76.00
71	是	否	是	女	155.00	89.00	160.00	93.00
71	否	否	是	女	117.00	47.00	98.00	46.00
73	是	否	是	女	136.00	86.00	138.00	90.00
84	是	否	是	女	138.00	90.00	160.00	90.00
71	是	否	是	女	120.00	78.00	124.00	80.00
70	是	否	是	女	145.00	89.00	140.00	85.00
80	是	是	是	女	140.00	78.00	144.00	80.00
66	否	否	是	女	150.00	80.00	150.00	90.00
74	是	是	是	女	148.00	80.00	148.00	86.00
67	是	是	是	女	139.00	89.00	138.00	87.00
69	是	否	是	女	140.00	90.00	150.00	90.00
87	否	否	是	女	124.00	70.00	126.00	78.00
76	是	否	是	女	145.00	80.00	150.00	83.00
70	是	否	是	女	134.00	74.00	136.00	74.00
80	否	是	是	女	140.00	60.00	148.00	62.00
68	是	否	是	女	140.00	80.00	145.00	80.00
70	是	是	是	女	120.00	60.00	118.00	70.00
87	是	是	是	女	120.00	70.00	122.00	74.00
87	是	否	是	女	140.00	70.00	142.00	70.00
85	是	否	是	女	138.00	79.00	140.00	80.00
84	是	是	是	女	124.00	64.00	126.00	68.00
87	是	否	是	女	142.00	80.00	146.00	80.00
74	否	否	是	女	136.00	88.00	140.00	88.00
67	否	是	是	女	120.00	70.00	122.00	74.00
69	否	否	是	女	130.00	70.00	135.00	72.00
69	是	否	是	女	130.00	72.00	134.00	76.00
71	是	否	是	女	120.00	78.00	118.00	80.00
71	是	否	是	女	139.00	81.00	148.00	80.00
69	是	否	是	女	130.00	72.00	135.00	75.00
86	是	否	是	女	146.00	72.00	148.00	77.00
67	是	否	是	女	110.00	80.00	111.00	80.00
76	是	否	是	女	125.00	67.00	128.00	70.00
76	否	否	是	女	138.00	83.00	130.00	80.00
77	否	否	是	女	130.00	80.00	135.00	87.00
70	否	否	是	女	130.00	80.00	132.00	82.00
74	是	否	是	女	120.00	70.00	128.00	70.00
69	否	是	是	女	130.00	80.00	138.00	80.00
88	否	否	是	女	138.00	72.00	136.00	74.00
72	否	否	是	女	116.00	82.00	133.00	84.00
78	否	否	是	女	90.00	64.00	94.00	60.00
69	否	否	是	女	110.00	70.00	116.00	74.00
67	否	否	是	女	154.00	87.00	146.00	76.00
90	是	否	是	女	160.00	89.00	159.00	86.00
68	否	是	是	女	120.00	78.00	124.00	80.00
77	是	否	是	女	135.00	76.00	138.00	80.00
81	是	否	是	女	140.00	86.00	130.00	88.00
85	否	否	是	女	140.00	85.00	150.00	89.00
72	是	否	是	女	152.00	94.00	150.00	92.00
69	否	否	是	女	110.00	68.00	112.00	70.00

69	否	否	是	女	148.00	80.00	150.00	80.00
86	是	否	是	女	120.00	70.00	115.00	70.00
71	是	是	是	女	138.00	88.00	145.00	85.00
74	否	否	是	女	139.00	88.00	135.00	80.00
77	是	否	是	女	139.00	86.00	135.00	82.00
76	是	否	是	女	140.00	70.00	148.00	70.00
67	否	否	是	女	138.00	76.00	139.00	80.00
90	是	是	是	女	120.00	60.00	124.00	62.00
68	是	否	是	女	139.00	90.00	140.00	85.00
70	是	是	是	女	101.00	69.00	106.00	70.00
76	是	否	是	女	140.00	95.00	141.00	93.00
68	否	否	是	女	112.00	66.00	112.00	68.00
70	是	是	是	女	136.00	70.00	136.00	70.00
67	是	否	是	女	130.00	80.00	138.00	82.00
76	是	是	是	女	180.00	88.00	165.00	86.00
76	是	否	是	女	158.00	78.00	160.00	80.00
67	否	否	是	女	134.00	80.00	130.00	82.00
85	是	否	是	女	140.00	86.00	145.00	88.00
80	是	否	是	女	135.00	82.00	138.00	86.00
71	否	否	是	女	130.00	80.00	139.00	90.00
66	否	否	是	女	139.00	69.00	142.00	72.00
73	否	否	是	女	108.00	76.00	110.00	78.00
71	是	否	是	女	150.00	80.00	156.00	82.00
70	是	是	是	女	124.00	78.00	124.00	76.00
69	否	是	是	女	144.00	80.00	140.00	78.00
75	否	否	是	女	136.00	88.00	140.00	88.00
69	是	否	是	女	136.00	92.00	139.00	94.00
73	否	否	是	女	130.00	100.00	130.00	90.00
97	是	否	是	女	146.00	70.00	150.00	70.00
78	是	否	是	女	138.00	82.00	130.00	70.00
72	否	否	是	女	151.00	76.00	140.00	89.00
66	是	否	是	女	137.00	88.00	136.00	84.00
67	是	否	是	女	126.00	80.00	130.00	80.00
72	否	否	是	女	138.00	85.00	140.00	92.00
76	是	否	是	女	154.00	90.00	160.00	90.00
69	是	否	是	女	108.00	73.00	118.00	70.00
89	是	否	是	女	135.00	65.00	138.00	69.00
69	是	否	是	女	158.00	78.00	161.00	81.00
67	否	否	是	女	110.00	72.00	113.00	75.00
73	是	否	是	女	138.00	89.00	138.00	87.00
73	是	是	是	女	110.00	80.00	116.00	78.00
68	否	否	是	女	160.00	87.00	162.00	89.00
75	是	否	是	女	134.00	93.00	143.00	99.00
71	是	是	是	女	160.00	90.00	160.00	95.00
68	是	否	是	女	138.00	71.00	139.00	82.00
87	否	否	是	女	110.00	80.00	100.00	70.00
67	否	否	是	女	138.00	82.00	132.00	87.00
81	是	否	是	女	140.00	82.00	140.00	80.00
74	是	是	是	女	138.00	87.00	142.00	88.00
69	是	否	是	女	128.00	80.00	130.00	82.00
74	是	是	是	女	130.00	70.00	136.00	76.00

70	是	否	是	女	140.00	70.00	148.00	70.00
89	是	是	是	女	117.00	92.00	120.00	89.00
70	否	否	是	女	138.00	71.00	135.00	80.00
72	是	是	是	女	139.00	82.00	139.00	85.00
74	是	是	是	女	140.00	83.00	146.00	84.00
68	否	否	是	女	140.00	90.00	150.00	90.00
71	否	否	是	女	125.00	77.00	121.00	90.00
66	是	是	是	女	157.00	100.00	151.00	88.00
75	否	否	是	女	118.00	70.00	120.00	78.00
83	是	否	是	女	148.00	100.00	131.00	93.00
70	否	是	是	女	120.00	80.00	120.00	90.00
80	是	否	是	女	135.00	89.00	130.00	80.00
66	否	否	是	女	122.00	79.00	120.00	80.00
66	否	否	是	女	90.00	60.00	90.00	60.00
74	是	否	是	女	108.00	65.00	110.00	70.00
69	否	否	是	女	142.00	98.00	140.00	95.00
86	是	否	是	女	130.00	76.00	138.00	78.00
71	否	否	是	女	150.00	90.00	140.00	85.00
68	否	否	是	女	128.00	77.00	120.00	78.00
76	是	是	是	女	191.00	98.00	207.00	100.00
81	是	是	是	女	130.00	75.00	136.00	77.00
66	是	否	是	女	130.00	78.00	146.00	88.00
71	是	否	是	女	134.00	78.00	130.00	72.00
71	是	否	是	女	139.00	69.00	156.00	82.00
66	是	否	是	女	172.00	90.00	178.00	90.00
69	否	否	是	女	145.00	90.00	140.00	85.00
71	是	否	是	女	134.00	80.00	140.00	80.00
79	是	否	是	女	141.00	84.00	128.00	81.00
73	是	是	是	女	145.00	90.00	154.00	92.00
66	否	否	是	女	126.00	64.00	130.00	70.00
74	否	否	是	女	120.00	70.00	124.00	77.00
80	是	否	是	女	129.00	66.00	131.00	69.00
72	是	是	是	女	130.00	70.00	122.00	72.00
66	否	否	是	女	136.00	88.00	140.00	88.00
73	否	否	是	女	114.00	68.00	118.00	68.00
82	是	否	是	女	165.00	91.00	168.00	99.00
66	否	否	是	女	112.00	78.00	120.00	80.00
80	是	否	是	女	144.00	80.00	146.00	82.00
72	是	否	是	女	115.00	75.00	118.00	70.00
71	是	否	是	女	135.00	75.00	139.00	79.00
77	否	否	是	女	120.00	80.00	132.00	82.00
67	否	否	是	女	160.00	83.00	161.00	85.00
67	是	否	是	女	110.00	68.00	114.00	70.00
74	是	否	是	女	138.00	88.00	140.00	88.00
67	是	否	是	女	141.00	89.00	128.00	82.00
69	是	是	是	女	135.00	82.00	139.00	84.00
74	是	否	是	女	122.00	68.00	122.00	69.00
77	是	否	是	女	149.00	69.00	158.00	78.00
74	否	否	是	女	150.00	82.00	152.00	90.00
78	是	是	是	女	128.00	69.00	126.00	66.00
73	是	否	是	女	140.00	84.00	142.00	88.00

71	是	否	是	女	130.00	70.00	135.00	70.00
69	是	是	是	女	118.00	66.00	120.00	70.00
85	是	否	是	女	126.00	80.00	130.00	81.00
69	否	否	是	女	130.00	78.00	136.00	90.00
80	否	否	是	女	120.00	80.00	118.00	72.00
91	是	是	是	女	159.00	100.00	143.00	89.00
71	是	否	是	女	130.00	81.00	113.00	74.00
67	否	是	是	女	130.00	72.00	138.00	80.00
68	否	否	是	女	105.00	70.00	100.00	65.00
68	否	否	是	女	122.00	72.00	124.00	74.00
75	否	否	是	女	130.00	80.00	125.00	75.00
74	否	否	是	女	120.00	90.00	138.00	98.00
69	否	否	是	女	113.00	72.00	115.00	64.00
71	否	否	是	女	122.00	78.00	125.00	80.00
73	是	否	是	女	130.00	80.00	128.00	76.00
70	是	否	是	女	150.00	80.00	154.00	87.00
79	否	否	是	女	139.00	81.00	138.00	88.00
73	是	是	是	女	133.00	89.00	138.00	89.00
72	是	否	是	女	130.00	80.00	130.00	86.00
74	是	是	是	女	133.00	82.00	132.00	80.00
77	否	否	是	女	216.00	95.00	207.00	93.00
66	否	否	是	女	116.00	75.00	120.00	76.00
68	是	否	是	女	120.00	76.00	118.00	70.00
83	否	否	是	女	180.00	100.00	182.00	106.00
73	否	否	是	女	125.00	73.00	120.00	70.00
93	否	是	是	女	128.00	70.00	136.00	78.00
81	是	是	是	女	129.00	67.00	129.00	68.00
77	是	否	是	女	140.00	60.00	138.00	66.00
73	否	否	是	女	124.00	70.00	120.00	67.00
71	是	否	是	女	136.00	90.00	138.00	90.00
82	是	否	是	女	130.00	85.00	132.00	89.00
93	是	否	是	女	179.00	71.00	186.00	76.00
88	否	否	是	女	136.00	80.00	139.00	84.00
69	是	否	是	女	128.00	72.00	96.00	75.00
74	否	否	是	女	145.00	85.00	150.00	90.00
68	否	否	是	女	110.00	70.00	108.00	69.00
67	是	否	是	女	132.00	92.00	130.00	90.00
72	是	否	是	女	129.00	77.00	130.00	80.00
72	否	否	是	女	138.00	80.00	140.00	86.00
80	是	否	是	女	96.00	62.00	98.00	64.00
68	否	否	是	女	139.00	70.00	135.00	65.00
74	否	否	是	女	142.00	60.00	138.00	65.00
81	是	否	是	女	138.00	78.00	142.00	80.00
82	否	否	是	女	134.00	78.00	138.00	82.00
70	否	是	是	女	132.00	85.00	130.00	80.00
68	是	是	是	女	170.00	100.00	180.00	102.00
72	否	否	是	女	135.00	82.00	138.00	85.00
84	是	否	是	女	140.00	89.00	139.00	79.00
80	是	否	是	女	150.00	95.00	147.00	100.00
85	是	否	是	女	151.00	87.00	145.00	90.00
68	否	否	是	女	120.00	70.00	118.00	75.00

69	是	是	是	女	150.00	80.00	158.00	82.00
70	否	否	是	女	150.00	88.00	152.00	90.00
89	是	否	是	女	145.00	78.00	138.00	76.00
81	否	否	是	女	164.00	89.00	175.00	89.00
68	否	否	是	女	130.00	70.00	138.00	76.00
73	是	否	是	女	110.00	70.00	118.00	70.00
94	否	否	是	女	124.00	60.00	129.00	62.00
82	否	否	是	女	135.00	72.00	138.00	75.00
74	否	否	是	女	107.00	68.00	106.00	65.00
73	是	否	是	女	130.00	80.00	131.00	76.00
92	是	否	是	女	100.00	70.00	110.00	70.00
82	否	否	是	女	120.00	70.00	140.00	80.00
81	否	否	是	女	120.00	62.00	122.00	64.00
67	是	否	是	女	138.00	80.00	132.00	78.00
67	否	否	是	女	112.00	68.00	116.00	72.00
68	是	否	是	女	168.00	104.00	164.00	100.00
92	是	否	是	女	206.00	98.00	200.00	90.00
98	是	否	是	女	154.00	83.00	150.00	85.00
79	是	是	是	女	139.00	88.00	135.00	78.00
74	是	否	是	女	118.00	74.00	120.00	76.00
80	是	否	是	女	140.00	75.00	135.00	75.00
71	是	否	是	女	180.00	110.00	185.00	110.00
75	是	是	是	女	110.00	75.00	105.00	70.00
71	否	否	是	女	150.00	84.00	146.00	76.00
66	否	否	是	女	110.00	70.00	115.00	76.00
80	是	否	是	女	180.00	90.00	175.00	90.00
80	是	否	是	女	130.00	60.00	128.00	65.00
68	否	否	是	女	120.00	81.00	124.00	81.00
87	是	否	是	女	135.00	81.00	138.00	84.00
77	是	是	是	女	155.00	79.00	158.00	82.00
73	是	否	是	女	134.00	82.00	138.00	86.00
88	是	否	是	女	149.00	89.00	142.00	78.00
75	否	是	是	女	164.00	84.00	170.00	92.00
73	是	否	是	女	120.00	60.00	118.00	68.00
79	是	否	是	女	120.00	70.00	122.00	70.00
68	否	否	是	女	120.00	78.00	127.00	76.00
85	是	否	是	女	143.00	83.00	145.00	86.00
79	是	否	是	女	120.00	80.00	130.00	86.00
68	否	否	是	女	134.00	90.00	130.00	85.00
76	是	否	是	女	130.00	76.00	132.00	76.00
66	否	否	是	女	158.00	90.00	150.00	90.00
84	否	否	是	女	138.00	100.00	139.00	99.00
80	是	否	是	女	148.00	82.00	145.00	80.00
67	是	是	是	女	150.00	80.00	150.00	83.00
74	否	否	是	女	118.00	68.00	120.00	70.00
78	否	否	是	女	134.00	72.00	139.00	78.00
67	否	否	是	女	150.00	90.00	153.00	95.00
72	是	否	是	女	139.00	70.00	134.00	65.00
75	是	否	是	女	120.00	68.00	130.00	78.00
69	是	否	是	女	145.00	78.00	148.00	82.00
69	否	否	是	女	94.00	60.00	98.00	60.00

80	是	否	是	女	128.00	70.00	130.00	80.00
69	是	否	是	女	146.00	82.00	148.00	88.00
81	否	否	是	女	139.00	80.00	135.00	82.00
69	是	否	是	女	146.00	76.00	150.00	80.00
70	是	否	是	女	156.00	96.00	158.00	100.00
67	否	否	是	女	168.00	96.00	155.00	86.00
75	是	否	是	女	160.00	90.00	150.00	80.00
68	否	否	是	女	135.00	82.00	139.00	84.00
78	是	否	是	女	120.00	80.00	132.00	81.00
83	是	否	是	女	126.00	60.00	128.00	60.00
89	是	否	是	女	102.00	72.00	100.00	74.00
71	否	否	是	女	129.00	77.00	132.00	80.00
75	是	否	是	女	110.00	62.00	112.00	60.00
74	是	是	是	女	136.00	83.00	144.00	86.00
81	是	否	是	女	128.00	70.00	132.00	74.00
70	否	否	是	女	138.00	88.00	140.00	88.00
87	否	是	是	女	130.00	76.00	130.00	80.00
72	是	否	是	女	140.00	82.00	150.00	96.00
77	否	否	是	女	110.00	60.00	112.00	65.00
73	否	否	是	女	136.00	82.00	139.00	84.00
78	否	否	是	女	138.00	64.00	138.00	64.00
72	是	否	是	女	122.00	68.00	124.00	69.00
72	是	否	是	女	154.00	90.00	148.00	96.00
85	是	是	是	女	165.00	74.00	159.00	89.00
86	是	否	是	女	128.00	67.00	130.00	70.00
74	是	否	是	女	124.00	78.00	128.00	80.00
74	否	否	是	女	128.00	78.00	128.00	76.00
71	否	否	是	女	150.00	90.00	145.00	85.00
70	是	否	是	女	135.00	74.00	138.00	76.00
66	否	否	是	女	120.00	70.00	125.00	75.00
74	是	否	是	女	124.00	64.00	126.00	68.00
66	是	否	是	女	148.00	92.00	153.00	95.00
85	否	否	是	女	120.00	62.00	122.00	62.00
72	是	否	是	女	110.00	70.00	108.00	68.00
84	是	否	是	女	170.00	100.00	180.00	110.00
85	是	否	是	女	135.00	80.00	137.00	83.00
66	是	否	是	女	140.00	100.00	135.00	100.00
95	否	否	是	女	146.00	90.00	140.00	87.00
82	是	否	是	女	130.00	81.00	132.00	79.00
69	否	否	是	女	136.00	86.00	138.00	84.00
74	是	否	是	女	155.00	78.00	156.00	85.00
69	否	否	是	女	135.00	68.00	139.00	72.00
88	是	是	是	女	122.00	86.00	120.00	84.00
76	否	否	是	女	130.00	68.00	139.00	74.00
76	否	是	是	女	118.00	70.00	122.00	76.00
71	否	否	是	女	150.00	80.00	154.00	88.00
77	否	否	是	女	142.00	80.00	140.00	80.00
92	是	是	是	女	147.00	92.00	148.00	89.00
67	是	否	是	女	140.00	70.00	160.00	70.00
66	是	否	是	女	118.00	70.00	120.00	75.00
79	否	否	是	女	170.00	90.00	178.00	90.00

75	是	否	是	女	158.00	100.00	160.00	100.00
71	是	否	是	女	160.00	100.00	170.00	95.00
67	是	否	是	女	147.00	82.00	157.00	73.00
79	否	否	是	女	138.00	70.00	140.00	70.00
86	否	否	是	女	130.00	70.00	135.00	80.00
92	是	否	是	女	186.00	92.00	194.00	85.00
71	是	是	是	女	146.00	76.00	148.00	80.00
88	是	否	是	女	120.00	64.00	124.00	69.00
69	否	否	是	女	125.00	75.00	130.00	80.00
74	是	否	是	女	122.00	68.00	126.00	72.00
92	是	否	是	女	155.00	87.00	158.00	90.00
70	是	否	是	女	126.00	80.00	128.00	80.00
75	否	否	是	女	118.00	64.00	120.00	70.00
75	否	否	是	女	127.00	67.00	139.00	70.00
73	是	否	是	女	139.00	89.00	140.00	87.00
67	是	否	是	女	135.00	89.00	130.00	84.00
84	否	否	是	女	110.00	70.00	105.00	65.00
92	否	否	是	女	138.00	74.00	139.00	72.00
71	是	否	是	女	115.00	78.00	125.00	80.00
70	否	否	是	女	138.00	68.00	140.00	70.00
84	否	是	是	女	112.00	73.00	115.00	75.00
66	是	否	是	女	130.00	80.00	136.00	82.00
72	否	否	是	女	122.00	68.00	120.00	68.00
66	否	否	是	女	142.00	75.00	148.00	76.00
73	是	否	是	女	130.00	80.00	148.00	89.00
75	否	否	是	女	142.00	80.00	140.00	80.00
75	否	否	是	女	120.00	66.00	118.00	70.00
73	是	否	是	女	162.00	90.00	160.00	94.00
71	是	否	是	女	170.00	90.00	168.00	88.00
81	是	否	是	女	136.00	82.00	138.00	84.00
73	否	否	是	女	146.00	95.00	142.00	84.00
67	否	否	是	女	136.00	80.00	138.00	80.00
66	否	否	是	女	115.00	78.00	120.00	80.00
77	是	否	是	女	140.00	80.00	142.00	82.00
69	否	否	是	女	132.00	81.00	135.00	84.00
68	否	否	是	女	110.00	80.00	112.00	78.00
76	否	否	是	女	122.00	82.00	120.00	80.00
83	是	否	是	女	140.00	90.00	170.00	82.00
71	是	否	是	女	130.00	80.00	130.00	85.00
82	否	否	是	女	135.00	75.00	140.00	88.00
66	否	否	是	女	125.00	60.00	122.00	65.00
68	否	否	是	女	100.00	70.00	102.00	71.00
67	是	否	是	女	150.00	91.00	151.00	92.00
76	否	否	是	女	139.00	70.00	135.00	72.00
80	是	是	是	女	136.00	86.00	130.00	80.00
67	否	否	是	女	147.00	63.00	140.00	88.00
70	是	否	是	女	122.00	70.00	126.00	72.00
68	否	否	是	女	170.00	102.00	172.00	104.00
76	是	是	是	女	138.00	89.00	137.00	88.00
74	是	否	是	女	113.00	70.00	116.00	72.00
76	是	否	是	女	130.00	77.00	140.00	78.00

72	否	否	是	女	118.00	76.00	120.00	80.00
73	否	否	是	女	120.00	77.00	135.00	81.00
86	否	否	是	女	155.00	91.00	160.00	94.00
67	是	否	是	女	148.00	82.00	151.00	84.00
72	否	否	是	女	110.00	76.00	110.00	72.00
67	否	否	是	女	140.00	80.00	135.00	85.00
71	是	是	是	女	180.00	70.00	190.00	80.00
76	是	否	是	女	148.00	74.00	146.00	70.00
70	是	否	是	女	135.00	71.00	138.00	80.00
68	是	否	是	女	170.00	90.00	168.00	88.00
71	是	否	是	女	156.00	96.00	160.00	98.00
66	否	否	是	女	140.00	88.00	142.00	90.00
68	是	否	是	女	160.00	80.00	164.00	88.00
71	是	否	是	女	128.00	75.00	125.00	72.00
69	是	否	是	女	160.00	72.00	164.00	76.00
77	是	否	是	女	125.00	71.00	128.00	74.00
79	是	否	是	女	128.00	76.00	132.00	80.00
69	否	否	是	女	136.00	84.00	138.00	86.00
67	否	否	是	女	142.00	80.00	146.00	80.00
67	是	否	是	女	139.00	89.00	135.00	85.00
66	是	是	是	女	150.00	80.00	150.00	90.00
77	否	是	是	女	126.00	72.00	132.00	70.00
89	是	是	是	女	147.00	78.00	137.00	78.00
68	否	否	是	女	135.00	80.00	130.00	80.00
72	是	否	是	女	120.00	76.00	120.00	80.00
71	是	否	是	女	131.00	69.00	133.00	80.00
69	是	是	是	女	130.00	81.00	128.00	78.00
72	是	是	是	女	127.00	85.00	125.00	80.00
67	是	否	是	女	130.00	88.00	132.00	90.00
71	是	否	是	女	153.00	80.00	151.00	82.00
71	否	否	是	女	120.00	66.00	120.00	70.00
69	是	是	是	女	145.00	75.00	140.00	70.00
85	否	否	是	女	162.00	80.00	164.00	82.00
81	否	否	是	女	180.00	100.00	175.00	100.00
78	是	否	是	女	110.00	70.00	120.00	80.00
69	否	否	是	女	160.00	96.00	168.00	98.00
80	是	是	是	女	138.00	80.00	140.00	80.00
72	否	否	是	女	130.00	70.00	125.00	65.00
75	是	否	是	女	142.00	70.00	144.00	71.00
94	是	否	是	女	130.00	62.00	132.00	64.00
81	是	否	是	女	139.00	89.00	138.00	88.00
71	否	是	是	女	110.00	70.00	105.00	65.00
66	是	否	是	女	135.00	80.00	130.00	75.00
69	否	否	是	女	122.00	64.00	116.00	75.00
76	是	否	是	女	135.00	85.00	138.00	88.00
68	是	是	是	女	150.00	80.00	140.00	80.00
81	是	是	是	女	109.00	60.00	110.00	70.00
73	否	否	是	女	135.00	85.00	138.00	88.00
80	是	否	是	女	160.00	90.00	165.00	85.00
70	否	否	是	女	130.00	76.00	134.00	82.00
68	是	否	是	女	150.00	90.00	145.00	93.00

76	是	否	是	女	110.00	60.00	120.00	60.00
69	否	否	是	女	140.00	70.00	142.00	70.00
78	否	否	是	女	125.00	70.00	130.00	70.00
74	是	否	是	女	139.00	78.00	140.00	87.00
88	是	否	是	女	130.00	60.00	126.00	62.00
86	是	是	是	女	140.00	70.00	133.00	68.00
66	是	否	否	女	139.00	76.00	130.00	85.00
72	是	否	是	女	174.00	90.00	170.00	90.00
80	是	否	是	女	152.00	78.00	158.00	81.00
71	是	是	是	女	138.00	88.00	140.00	89.00
73	否	否	是	女	128.00	79.00	130.00	80.00
76	否	否	是	女	118.00	69.00	118.00	64.00
75	是	否	是	女	120.00	80.00	135.00	80.00
68	否	否	是	女	133.00	78.00	130.00	80.00
70	是	是	是	女	168.00	88.00	170.00	90.00
74	是	否	是	女	130.00	73.00	138.00	76.00
92	否	否	是	女	138.00	70.00	139.00	76.00
82	否	否	是	女	148.00	80.00	158.00	90.00
66	是	否	是	女	124.00	70.00	130.00	72.00
73	是	否	是	女	130.00	80.00	130.00	85.00
72	是	否	是	女	142.00	82.00	142.00	84.00
68	否	否	是	女	128.00	74.00	126.00	70.00
66	否	否	是	女	122.00	80.00	114.00	76.00
80	是	否	是	女	120.00	72.00	122.00	75.00
71	是	是	是	女	120.00	72.00	126.00	79.00
73	是	否	是	女	134.00	80.00	138.00	80.00
67	否	否	是	女	139.00	70.00	136.00	76.00
81	是	否	是	女	124.00	72.00	130.00	80.00
69	否	否	是	女	190.00	116.00	194.00	114.00
72	是	否	是	女	140.00	80.00	146.00	82.00
70	是	否	是	女	156.00	80.00	152.00	80.00
67	是	否	是	女	135.00	80.00	130.00	80.00
68	否	否	是	女	144.00	82.00	152.00	87.00
76	是	否	是	女	135.00	80.00	139.00	82.00
68	否	否	是	女	110.00	70.00	105.00	65.00
84	否	否	是	女	130.00	67.00	138.00	70.00
71	是	否	是	女	139.00	77.00	140.00	72.00
74	否	否	是	女	139.00	77.00	123.00	80.00
74	是	是	是	女	131.00	76.00	135.00	80.00
89	是	否	是	女	141.00	95.00	149.00	83.00
89	是	否	是	女	142.00	76.00	131.00	82.00
70	否	否	是	女	139.00	80.00	140.00	79.00
71	否	否	是	女	139.00	82.00	134.00	84.00
76	否	否	是	女	119.00	60.00	120.00	60.00
77	否	否	是	女	92.00	60.00	90.00	60.00
66	是	否	是	女	130.00	72.00	128.00	70.00
73	是	否	是	女	155.00	100.00	160.00	100.00
81	是	否	是	女	110.00	70.00	112.00	70.00
70	是	否	是	女	200.00	100.00	200.00	106.00
74	否	否	是	女	136.00	71.00	138.00	70.00
74	是	否	是	女	126.00	80.00	130.00	80.00

72	是	否	是	女	178.00	100.00	180.00	108.00
72	是	否	是	女	139.00	79.00	140.00	80.00
73	否	是	是	女	110.00	70.00	105.00	65.00
70	是	否	是	女	135.00	86.00	139.00	90.00
68	否	否	是	女	100.00	62.00	102.00	66.00
82	是	否	是	女	120.00	60.00	128.00	68.00
68	否	是	是	女	122.00	64.00	126.00	70.00
70	是	否	是	女	125.00	75.00	130.00	80.00
67	是	否	是	女	127.00	79.00	138.00	88.00
80	是	否	是	女	145.00	86.00	150.00	90.00
71	否	否	是	女	210.00	112.00	206.00	104.00
69	否	否	是	女	135.00	78.00	135.00	79.00
71	是	否	是	女	130.00	67.00	132.00	69.00
75	是	否	是	女	134.00	62.00	136.00	68.00
67	是	是	是	女	170.00	100.00	170.00	105.00
73	否	否	是	女	120.00	60.00	124.00	68.00
74	是	否	是	女	106.00	70.00	108.00	70.00
73	否	否	是	女	118.00	70.00	120.00	70.00
72	是	否	是	女	139.00	89.00	135.00	85.00
68	是	否	是	女	138.00	78.00	142.00	88.00
67	否	否	是	女	168.00	98.00	170.00	100.00
79	否	否	是	女	110.00	80.00	115.00	80.00
71	是	否	是	女	140.00	80.00	135.00	75.00
71	是	是	是	女	138.00	80.00	142.00	82.00
81	是	是	是	女	140.00	70.00	135.00	78.00
85	是	否	是	女	112.00	69.00	115.00	71.00
70	否	否	是	女	128.00	72.00	134.00	82.00
71	是	否	是	女	139.00	76.00	136.00	70.00
75	是	否	是	女	130.00	70.00	132.00	70.00
69	是	是	是	女	174.00	90.00	189.00	96.00
70	否	否	是	女	123.00	76.00	118.00	67.00
68	是	否	是	女	140.00	78.00	130.00	70.00
78	是	否	是	女	130.00	78.00	132.00	83.00
67	是	否	是	女	145.00	86.00	146.00	88.00
68	是	否	是	女	118.00	74.00	120.00	75.00
66	否	否	是	女	110.00	60.00	108.00	65.00
70	否	否	是	女	120.00	70.00	125.00	78.00
70	是	否	是	女	132.00	68.00	137.00	72.00
76	否	否	是	女	150.00	80.00	148.00	82.00
81	是	否	是	女	138.00	87.00	104.00	67.00
75	是	否	是	女	165.00	88.00	170.00	90.00
79	是	否	是	女	130.00	78.00	133.00	80.00
68	是	否	是	女	136.00	85.00	139.00	89.00
70	是	否	是	女	150.00	89.00	151.00	90.00
79	是	是	是	女	140.00	74.00	143.00	84.00
72	否	否	是	女	124.00	73.00	130.00	75.00
73	否	否	是	女	145.00	75.00	139.00	72.00
67	否	否	是	女	110.00	68.00	120.00	65.00
66	否	否	是	女	116.00	78.00	120.00	78.00
74	否	否	是	女	130.00	80.00	130.00	86.00
71	否	否	是	女	130.00	74.00	130.00	86.00

89	否	否	是	女	138.00	86.00	146.00	90.00
70	是	否	是	女	128.00	70.00	130.00	74.00
72	否	否	是	女	150.00	80.00	148.00	80.00
72	否	否	是	女	180.00	100.00	181.00	100.00
68	否	否	是	女	130.00	96.00	124.00	90.00
66	是	否	是	女	120.00	80.00	128.00	88.00
71	是	否	是	女	134.00	74.00	136.00	76.00
67	是	否	是	女	150.00	90.00	149.00	85.00
82	否	否	是	女	108.00	73.00	110.00	75.00
74	否	否	是	女	150.00	90.00	160.00	90.00
73	否	否	是	女	112.00	78.00	110.00	80.00
73	否	是	是	女	120.00	67.00	125.00	70.00
94	是	否	是	女	150.00	82.00	152.00	85.00
79	是	是	是	女	160.00	82.00	166.00	90.00
70	是	是	是	女	130.00	68.00	138.00	70.00
74	否	否	是	女	130.00	88.00	136.00	90.00
77	是	是	是	女	143.00	71.00	156.00	72.00
75	否	否	是	女	132.00	80.00	135.00	82.00
67	是	否	是	女	138.00	89.00	135.00	85.00
76	是	否	是	女	185.00	98.00	188.00	99.00
89	否	否	是	女	120.00	80.00	119.00	73.00
74	是	是	是	女	140.00	100.00	140.00	90.00
77	否	否	是	女	152.00	104.00	145.00	97.00
83	是	否	是	女	150.00	80.00	153.00	80.00
79	是	是	是	女	177.00	71.00	176.00	85.00
68	否	是	是	女	113.00	63.00	110.00	61.00
75	否	否	是	女	132.00	80.00	135.00	82.00
67	否	否	是	女	130.00	86.00	120.00	85.00
66	是	是	是	女	135.00	82.00	136.00	85.00
91	否	否	是	女	140.00	80.00	132.00	77.00
69	是	是	是	女	134.00	82.00	139.00	82.00
66	是	否	否	女	130.00	80.00	135.00	82.00
87	否	否	是	女	130.00	85.00	133.00	87.00
75	是	否	是	女	138.00	78.00	137.00	76.00
73	否	否	是	女	138.00	87.00	135.00	82.00
80	否	否	是	女	128.00	84.00	128.00	88.00
71	是	否	是	女	135.00	85.00	138.00	89.00
71	是	否	是	女	163.00	95.00	165.00	86.00
86	否	否	是	女	138.00	66.00	139.00	68.00
76	是	否	是	女	130.00	70.00	126.00	88.00
70	否	否	是	女	120.00	80.00	131.00	88.00
74	是	否	是	女	130.00	78.00	135.00	80.00
74	是	否	是	女	122.00	66.00	128.00	70.00
85	是	否	是	女	130.00	78.00	134.00	80.00
66	否	否	否	女	152.00	70.00	146.00	74.00
74	是	否	是	女	138.00	72.00	139.00	65.00
69	是	否	是	女	140.00	86.00	140.00	90.00
77	是	否	是	女	163.00	105.00	166.00	108.00
72	是	是	是	女	140.00	70.00	138.00	76.00
73	是	否	是	女	120.00	70.00	124.00	72.00
73	是	否	是	女	118.00	70.00	120.00	70.00

68	否	否	是	女	136.00	83.00	135.00	80.00
82	是	否	是	女	205.00	99.00	210.00	114.00
70	否	否	是	女	160.00	100.00	166.00	100.00
73	否	否	是	女	140.00	90.00	134.00	89.00
72	是	是	是	女	128.00	84.00	130.00	86.00
67	否	否	是	女	138.00	79.00	140.00	80.00
73	否	否	是	女	120.00	80.00	130.00	70.00
82	是	否	是	女	140.00	66.00	140.00	70.00
71	是	是	是	女	120.00	70.00	128.00	80.00
73	是	否	是	女	109.00	76.00	112.00	78.00
70	是	否	是	女	158.00	90.00	169.00	96.00
77	是	否	是	女	116.00	72.00	124.00	61.00
69	是	否	是	女	132.00	82.00	130.00	80.00
74	否	否	是	女	124.00	74.00	120.00	76.00
73	是	是	是	女	132.00	82.00	136.00	84.00
72	否	否	是	女	120.00	64.00	116.00	62.00
88	否	否	是	女	130.00	70.00	132.00	76.00
72	否	否	是	女	136.00	76.00	139.00	79.00
73	否	是	是	女	115.00	66.00	110.00	66.00
69	否	否	是	女	137.00	72.00	147.00	70.00
70	否	是	是	女	140.00	90.00	145.00	96.00
74	否	否	是	女	139.00	80.00	140.00	80.00
73	是	否	是	女	121.00	60.00	120.00	62.00
71	是	否	是	女	136.00	78.00	142.00	80.00
69	否	否	是	女	118.00	70.00	116.00	72.00
69	否	否	是	女	129.00	90.00	139.00	96.00
85	是	否	是	女	135.00	71.00	123.00	66.00
71	是	否	是	女	138.00	80.00	140.00	80.00
71	是	否	是	女	128.00	78.00	132.00	80.00
71	否	否	是	女	112.00	68.00	114.00	69.00
67	否	否	是	女	150.00	80.00	154.00	80.00
87	否	否	是	女	118.00	75.00	120.00	78.00
76	是	是	是	女	130.00	66.00	137.00	68.00
73	否	否	是	女	163.00	98.00	164.00	101.00
69	否	是	是	女	134.00	84.00	139.00	89.00
73	否	否	是	女	139.00	79.00	138.00	78.00
72	否	否	是	女	112.00	62.00	120.00	70.00
66	是	否	是	女	160.00	97.00	162.00	98.00
70	是	是	是	女	160.00	90.00	155.00	85.00
70	是	否	是	女	150.00	85.00	148.00	79.00
69	否	否	是	女	135.00	91.00	131.00	78.00
74	是	是	是	女	138.00	85.00	140.00	90.00
70	否	否	是	女	142.00	90.00	150.00	90.00
75	否	否	是	女	120.00	78.00	123.00	87.00
81	否	否	是	女	126.00	74.00	128.00	76.00
69	否	否	是	女	130.00	85.00	138.00	86.00
73	是	否	是	女	156.00	84.00	152.00	84.00
71	是	否	是	女	135.00	80.00	138.00	80.00
76	是	是	是	女	139.00	88.00	135.00	78.00
77	否	否	是	女	120.00	74.00	120.00	74.00
86	是	是	是	女	154.00	81.00	160.00	80.00

69	是	否	是	女	130.00	80.00	136.00	82.00
74	否	否	是	女	120.00	80.00	120.00	80.00
70	否	否	是	女	148.00	93.00	152.00	88.00
66	否	否	是	女	152.00	83.00	155.00	93.00
67	是	否	是	女	142.00	88.00	144.00	89.00
81	是	否	是	女	120.00	65.00	119.00	63.00
67	是	是	是	女	140.00	72.00	136.00	68.00
68	否	否	是	女	100.00	60.00	102.00	62.00
69	否	否	是	女	106.00	66.00	110.00	70.00
69	是	是	是	女	120.00	70.00	126.00	76.00
73	是	否	是	女	128.00	68.00	130.00	70.00
72	是	否	是	女	135.00	85.00	139.00	89.00
79	是	否	是	女	131.00	63.00	135.00	67.00
71	是	否	是	女	166.00	100.00	160.00	105.00
70	是	是	是	女	105.00	65.00	100.00	60.00
71	是	否	是	女	134.00	78.00	138.00	76.00
66	否	否	是	女	138.00	89.00	128.00	89.00
88	否	否	是	女	141.00	85.00	145.00	89.00
67	是	是	是	女	144.00	80.00	139.00	85.00
82	是	否	是	女	129.00	80.00	139.00	82.00
69	是	是	是	女	155.00	90.00	164.00	95.00
67	否	是	是	女	120.00	75.00	122.00	78.00
80	是	否	是	女	138.00	70.00	140.00	70.00
67	否	否	是	女	116.00	70.00	120.00	70.00
78	否	否	是	女	120.00	70.00	125.00	80.00
69	否	是	是	女	100.00	60.00	101.00	61.00
67	是	是	是	女	110.00	64.00	116.00	68.00
90	是	否	是	女	160.00	60.00	160.00	70.00
72	是	否	是	女	170.00	90.00	190.00	95.00
67	否	否	是	女	122.00	82.00	126.00	86.00
68	否	否	是	女	118.00	70.00	120.00	72.00
72	是	否	是	女	130.00	80.00	135.00	85.00
75	是	否	是	女	133.00	79.00	136.00	82.00
73	是	否	是	女	159.00	81.00	160.00	80.00
71	是	否	是	女	162.00	91.00	169.00	92.00
72	否	否	是	女	135.00	89.00	140.00	90.00
87	是	否	是	女	110.00	60.00	114.00	62.00
78	是	否	是	女	119.00	66.00	122.00	68.00
81	是	是	是	女	139.00	72.00	144.00	68.00
72	否	是	是	女	125.00	78.00	124.00	80.00
71	否	是	是	女	169.00	70.00	159.00	72.00
72	是	否	是	女	120.00	89.00	115.00	84.00
74	是	否	是	女	169.00	86.00	172.00	82.00
72	是	否	是	女	118.00	62.00	114.00	60.00
75	是	否	是	女	139.00	90.00	136.00	84.00
68	是	是	是	女	132.00	78.00	135.00	80.00
80	否	否	是	女	108.00	76.00	110.00	80.00
71	否	否	是	女	170.00	80.00	180.00	80.00
73	是	否	是	女	130.00	70.00	136.00	76.00
80	否	否	是	女	140.00	60.00	136.00	66.00
68	否	否	是	女	119.00	91.00	133.00	97.00

73	否	否	是	女	126.00	72.00	118.00	70.00
66	是	否	否	女	128.00	68.00	132.00	70.00
83	是	否	是	女	135.00	70.00	137.00	78.00
78	是	否	是	女	132.00	84.00	136.00	88.00
76	是	否	是	女	138.00	80.00	140.00	80.00
71	否	否	是	女	135.00	70.00	130.00	70.00
68	否	否	是	女	106.00	68.00	108.00	72.00
89	是	否	是	女	110.00	70.00	115.00	64.00
69	否	否	是	女	111.00	72.00	115.00	76.00
82	是	是	是	女	140.00	70.00	148.00	66.00
83	是	否	是	女	194.00	90.00	171.00	93.00
70	是	是	是	女	130.00	70.00	125.00	65.00
76	否	否	是	女	128.00	60.00	125.00	65.00
79	是	否	是	女	138.00	72.00	146.00	89.00
68	否	否	是	女	139.00	72.00	138.00	74.00
74	是	否	是	女	186.00	76.00	190.00	80.00
70	否	否	是	女	120.00	80.00	120.00	85.00
68	否	否	是	女	130.00	78.00	130.00	86.00
67	否	否	是	女	118.00	68.00	120.00	64.00
78	否	否	是	女	146.00	82.00	150.00	86.00
70	否	是	是	女	120.00	76.00	124.00	80.00
67	否	否	是	女	100.00	60.00	109.00	67.00
70	否	否	是	女	112.00	76.00	120.00	78.00
80	是	否	是	女	140.00	89.00	145.00	90.00
74	是	否	是	女	140.00	70.00	135.00	70.00
68	是	否	是	女	128.00	76.00	130.00	78.00
66	否	否	是	女	137.00	87.00	139.00	89.00
75	否	否	是	女	134.00	88.00	136.00	74.00
83	否	否	是	女	98.00	60.00	96.00	60.00
68	否	是	是	女	107.00	71.00	110.00	74.00
86	否	否	是	女	122.00	74.00	125.00	77.00
80	是	否	是	女	160.00	100.00	166.00	106.00
73	否	否	是	女	126.00	78.00	130.00	82.00
69	是	否	是	女	136.00	86.00	140.00	86.00
75	否	否	是	女	112.00	77.00	110.00	80.00
66	是	是	是	女	140.00	80.00	143.00	80.00
73	是	是	是	女	139.00	84.00	138.00	82.00
67	否	否	是	女	139.00	85.00	140.00	88.00
77	是	是	是	女	142.00	91.00	146.00	92.00
73	否	否	是	女	138.00	90.00	140.00	90.00
70	否	否	是	女	137.00	87.00	134.00	88.00
81	是	否	是	女	150.00	90.00	148.00	90.00
74	否	是	是	女	140.00	75.00	122.00	70.00
74	否	否	是	女	120.00	80.00	122.00	80.00
76	是	否	是	女	150.00	87.00	151.00	89.00
84	否	是	是	女	150.00	78.00	156.00	82.00
75	是	是	是	女	120.00	80.00	123.00	88.00
88	是	否	是	女	148.00	86.00	152.00	90.00
66	是	否	是	女	105.00	65.00	100.00	60.00
72	否	否	是	女	110.00	80.00	105.00	75.00
67	否	否	是	女	120.00	78.00	110.00	70.00

67	否	否	是	女	160.00	92.00	162.00	93.00
79	是	否	是	女	140.00	70.00	150.00	80.00
70	否	否	是	女	122.00	68.00	119.00	68.00
71	是	否	是	女	127.00	70.00	139.00	68.00
69	是	否	是	女	134.00	73.00	139.00	78.00
68	是	是	是	女	139.00	78.00	139.00	80.00
66	是	否	是	女	140.00	78.00	148.00	90.00
71	是	是	是	女	142.00	86.00	145.00	90.00
68	是	否	是	女	116.00	78.00	118.00	80.00
77	否	否	是	女	127.00	86.00	130.00	84.00
75	是	否	是	女	139.00	89.00	138.00	88.00
84	是	否	是	女	120.00	60.00	122.00	61.00
71	是	否	是	女	117.00	75.00	120.00	78.00
83	是	否	是	女	138.00	78.00	130.00	77.00
68	否	否	是	女	102.00	75.00	100.00	78.00
78	是	否	是	女	118.00	65.00	108.00	68.00
70	否	否	是	女	138.00	70.00	142.00	72.00
80	是	否	是	女	135.00	62.00	130.00	60.00
70	是	否	是	女	112.00	70.00	117.00	78.00
70	是	否	是	女	118.00	76.00	120.00	78.00
76	是	否	是	女	140.00	80.00	135.00	79.00
77	是	是	是	女	138.00	88.00	140.00	90.00
70	是	否	是	女	150.00	80.00	153.00	78.00
76	是	否	是	女	135.00	80.00	138.00	80.00
67	是	是	是	女	118.00	76.00	124.00	80.00
73	是	否	是	女	150.00	100.00	150.00	90.00
67	否	否	是	女	130.00	76.00	128.00	70.00
93	是	否	是	女	139.00	80.00	136.00	82.00
70	是	否	是	女	134.00	77.00	140.00	70.00
70	否	否	是	女	123.00	60.00	98.00	62.00
89	是	否	是	女	134.00	84.00	140.00	90.00
75	否	否	是	女	136.00	72.00	138.00	89.00
69	否	否	是	女	129.00	74.00	158.00	81.00
69	是	是	是	女	140.00	73.00	124.00	66.00
70	是	否	是	女	122.00	82.00	120.00	78.00
79	是	否	是	女	130.00	70.00	138.00	78.00
72	是	是	是	女	130.00	75.00	120.00	70.00
70	是	否	是	女	115.00	75.00	120.00	70.00
72	是	否	是	女	139.00	73.00	137.00	74.00
69	是	否	是	女	158.00	96.00	160.00	100.00
76	否	否	是	女	118.00	75.00	120.00	80.00
87	否	否	是	女	136.00	86.00	140.00	88.00
67	是	否	是	女	146.00	80.00	144.00	78.00
66	否	否	是	女	110.00	76.00	110.00	78.00
71	是	否	是	女	146.00	86.00	150.00	90.00
70	是	否	是	女	135.00	86.00	140.00	90.00
77	是	是	是	女	161.00	78.00	165.00	80.00
90	是	否	是	女	190.00	100.00	150.00	70.00
83	是	否	是	女	146.00	80.00	150.00	82.00
78	是	否	是	女	140.00	70.00	140.00	80.00
71	是	是	是	女	130.00	70.00	131.00	77.00

76	否	否	是	女	120.00	80.00	122.00	84.00
76	是	是	是	女	120.00	76.00	123.00	84.00
66	是	否	是	女	138.00	88.00	135.00	85.00
69	是	否	是	女	135.00	78.00	120.00	80.00
66	是	否	是	女	130.00	80.00	132.00	80.00
70	是	否	是	女	126.00	76.00	130.00	80.00
69	是	否	是	女	140.00	75.00	145.00	76.00
76	是	是	是	女	128.00	72.00	132.00	76.00
79	否	否	是	女	98.00	60.00	100.00	60.00
68	否	否	是	女	120.00	80.00	120.00	80.00
66	否	否	是	女	150.00	90.00	140.00	80.00
67	否	否	是	女	120.00	70.00	125.00	80.00
81	是	否	是	女	148.00	89.00	150.00	90.00
67	否	否	是	女	127.00	76.00	128.00	78.00
69	是	否	是	女	134.00	78.00	138.00	78.00
75	是	否	是	女	170.00	98.00	170.00	100.00
77	是	是	是	女	114.00	64.00	118.00	68.00
74	是	否	是	女	140.00	80.00	140.00	90.00
73	否	否	是	女	128.00	72.00	130.00	80.00
68	否	否	是	女	130.00	82.00	132.00	82.00
68	是	否	是	女	175.00	85.00	180.00	90.00
76	是	否	是	女	128.00	84.00	136.00	88.00
67	否	否	是	女	150.00	80.00	162.00	84.00
75	是	否	是	女	167.00	89.00	162.00	90.00
71	否	否	是	女	138.00	88.00	140.00	90.00
71	是	否	是	女	110.00	60.00	120.00	60.00
67	是	是	是	女	124.00	80.00	120.00	84.00
71	是	否	是	女	138.00	94.00	142.00	98.00
66	是	否	否	女	130.00	70.00	150.00	80.00
67	否	否	是	女	118.00	64.00	122.00	68.00
70	否	否	是	女	133.00	75.00	136.00	78.00
69	是	否	是	女	148.00	98.00	150.00	100.00
76	否	否	是	女	135.00	77.00	138.00	80.00
67	否	否	是	女	136.00	88.00	138.00	88.00
66	是	否	否	女	158.00	86.00	162.00	90.00
77	否	是	是	女	133.00	85.00	136.00	88.00
66	否	否	是	女	136.00	84.00	138.00	88.00
71	否	否	是	女	136.00	88.00	138.00	89.00
71	是	否	是	女	178.00	70.00	180.00	70.00
67	是	否	是	女	110.00	62.00	110.00	60.00
67	是	否	是	女	133.00	69.00	136.00	72.00
76	是	否	是	女	110.00	59.00	114.00	63.00
74	是	否	是	女	129.00	81.00	132.00	84.00
81	否	否	是	女	133.00	71.00	130.00	70.00
73	是	否	是	女	144.00	80.00	146.00	80.00
72	是	否	是	女	135.00	80.00	139.00	88.00
79	否	否	是	女	133.00	65.00	135.00	70.00
77	是	否	是	女	180.00	82.00	150.00	80.00
69	是	否	是	女	135.00	80.00	138.00	84.00
68	是	否	是	女	160.00	100.00	150.00	100.00
77	是	是	是	女	142.00	79.00	148.00	80.00

80	否	是	是	女	130.00	70.00	132.00	76.00
72	是	否	是	女	132.00	76.00	135.00	78.00
88	否	否	是	女	122.00	80.00	130.00	77.00
79	是	否	是	女	135.00	80.00	134.00	83.00
87	是	否	是	女	166.00	86.00	174.00	82.00
76	是	否	是	女	130.00	70.00	135.00	70.00
66	否	否	否	女	130.00	75.00	135.00	80.00
98	是	否	是	女	135.00	70.00	138.00	70.00
75	是	否	是	女	120.00	80.00	125.00	83.00
67	否	否	是	女	138.00	89.00	141.00	90.00
70	是	否	是	女	129.00	82.00	114.00	70.00
68	否	否	是	女	120.00	64.00	122.00	64.00
83	是	否	是	女	128.00	72.00	132.00	76.00
67	是	是	是	女	167.00	99.00	164.00	94.00
74	否	否	是	女	136.00	89.00	139.00	84.00
76	是	是	是	女	120.00	82.00	130.00	80.00
73	否	否	是	女	100.00	80.00	102.00	80.00
69	是	否	是	女	137.00	83.00	139.00	84.00
71	否	否	是	女	142.00	100.00	134.00	98.00
77	是	否	是	女	100.00	70.00	105.00	70.00
74	否	否	是	女	134.00	80.00	136.00	81.00
66	是	否	是	女	122.00	68.00	124.00	70.00
70	否	否	是	女	151.00	79.00	140.00	83.00
67	是	是	是	女	134.00	78.00	138.00	80.00
75	是	否	是	女	158.00	81.00	152.00	79.00
82	是	否	是	女	132.00	64.00	134.00	66.00
73	是	否	是	女	144.00	90.00	144.00	92.00
68	是	否	是	女	130.00	86.00	135.00	88.00
74	是	否	是	女	156.00	78.00	158.00	80.00
66	否	否	是	女	128.00	69.00	134.00	77.00
74	否	否	是	女	126.00	64.00	128.00	66.00
73	是	否	是	女	135.00	80.00	137.00	68.00
66	否	否	是	女	138.00	88.00	140.00	90.00
79	是	否	是	女	130.00	78.00	139.00	82.00
69	否	否	是	女	135.00	90.00	138.00	90.00
76	是	否	是	女	121.00	70.00	110.00	72.00
72	否	否	是	女	148.00	98.00	150.00	100.00
68	是	否	是	女	132.00	80.00	138.00	80.00
69	是	否	是	女	164.00	86.00	156.00	83.00
73	否	否	是	女	124.00	64.00	131.00	64.00
83	是	否	是	女	140.00	72.00	138.00	76.00
90	否	否	是	女	131.00	90.00	138.00	88.00
74	是	是	是	女	150.00	70.00	150.00	80.00
78	否	否	是	女	110.00	68.00	122.00	72.00
74	否	否	是	女	134.00	84.00	137.00	81.00
77	否	否	是	女	100.00	60.00	104.00	62.00
67	是	否	是	女	140.00	88.00	138.00	88.00
76	是	否	是	女	135.00	79.00	138.00	80.00
84	是	否	是	女	116.00	68.00	120.00	72.00
88	是	否	是	女	139.00	67.00	138.00	72.00
73	是	否	是	女	142.00	84.00	144.00	89.00

68	是	否	是	女	132.00	81.00	130.00	80.00
75	否	否	是	女	110.00	70.00	108.00	68.00
78	否	否	是	女	138.00	66.00	140.00	65.00
83	是	否	是	女	144.00	89.00	150.00	90.00
78	是	否	是	女	158.00	87.00	162.00	92.00
77	否	否	是	女	110.00	70.00	106.00	68.00
68	是	否	是	女	133.00	71.00	136.00	74.00
75	是	否	是	女	120.00	68.00	126.00	70.00
72	否	否	是	女	140.00	75.00	140.00	80.00
80	是	否	是	女	134.00	86.00	138.00	88.00
76	是	否	是	女	124.00	70.00	128.00	74.00
73	是	否	是	女	126.00	78.00	130.00	80.00
75	是	否	是	女	195.00	100.00	200.00	120.00
76	是	是	是	女	138.00	98.00	140.00	96.00
68	否	否	是	女	139.00	90.00	124.00	90.00
75	是	否	是	女	160.00	90.00	155.00	90.00
69	否	否	是	女	142.00	85.00	150.00	90.00
72	是	否	是	女	154.00	92.00	142.00	92.00
76	是	是	是	女	138.00	80.00	140.00	84.00
84	是	否	是	女	140.00	82.00	152.00	85.00
82	否	否	是	女	118.00	72.00	122.00	70.00
67	是	是	是	女	136.00	78.00	138.00	80.00
70	否	是	是	女	123.00	75.00	138.00	73.00
81	是	否	是	女	139.00	85.00	142.00	83.00
67	是	否	是	女	148.00	78.00	150.00	80.00
72	否	否	是	女	137.00	73.00	144.00	92.00
88	是	否	是	女	167.00	88.00	168.00	89.00
67	是	否	是	女	150.00	70.00	153.00	72.00
72	是	否	是	女	131.00	83.00	134.00	84.00
77	是	否	是	女	162.00	78.00	161.00	81.00
68	否	否	是	女	127.00	85.00	130.00	88.00
87	是	否	是	女	121.00	61.00	124.00	64.00
69	是	否	是	女	136.00	53.00	138.00	63.00
68	是	否	是	女	130.00	80.00	139.00	82.00
66	否	否	是	女	124.00	82.00	125.00	84.00
96	是	否	是	女	150.00	90.00	149.00	88.00
70	是	否	是	女	148.00	86.00	144.00	82.00
66	否	否	是	女	145.00	90.00	140.00	88.00
74	否	否	是	女	130.00	74.00	136.00	76.00
72	是	否	是	女	124.00	66.00	130.00	70.00
89	否	否	是	女	130.00	70.00	135.00	75.00
73	是	是	是	女	135.00	70.00	130.00	70.00
74	否	是	是	女	136.00	70.00	138.00	71.00
77	是	否	是	女	142.00	80.00	150.00	86.00
73	否	否	是	女	139.00	68.00	145.00	70.00
68	否	否	是	女	154.00	96.00	156.00	98.00
69	否	否	是	女	114.00	74.00	126.00	75.00
67	是	否	是	女	130.00	65.00	136.00	66.00
77	是	否	是	女	139.00	81.00	118.00	90.00
76	是	否	是	女	137.00	80.00	136.00	84.00
72	是	是	是	女	136.00	77.00	135.00	70.00

77	是	否	是	女	130.00	80.00	125.00	75.00
75	是	否	是	女	138.00	78.00	135.00	75.00
73	是	是	是	女	140.00	76.00	152.00	80.00
69	否	否	是	女	134.00	86.00	138.00	80.00
66	是	是	是	女	130.00	70.00	120.00	80.00
74	否	否	是	女	120.00	80.00	122.00	83.00
70	是	否	是	女	125.00	84.00	120.00	80.00
83	是	否	是	女	130.00	82.00	130.00	80.00
76	是	否	是	女	190.00	98.00	200.00	100.00
67	是	否	是	女	142.00	78.00	146.00	80.00
73	是	是	是	女	135.00	80.00	140.00	80.00
84	是	否	是	女	170.00	88.00	160.00	88.00
74	否	否	是	女	106.00	62.00	108.00	62.00
72	否	否	是	女	140.00	90.00	158.00	99.00
67	否	否	是	女	138.00	70.00	135.00	67.00
88	否	否	是	女	130.00	80.00	136.00	78.00
67	否	否	是	女	107.00	72.00	110.00	75.00
70	否	否	是	女	126.00	70.00	130.00	70.00
68	否	否	是	女	114.00	74.00	116.00	76.00
67	是	是	是	女	100.00	68.00	110.00	70.00
70	否	否	是	女	126.00	84.00	132.00	84.00
79	是	否	是	女	132.00	81.00	138.00	91.00
85	是	否	是	女	136.00	80.00	134.00	80.00
70	是	是	是	女	140.00	68.00	138.00	74.00
67	是	是	是	女	120.00	68.00	115.00	60.00
74	是	否	是	女	140.00	62.00	138.00	60.00
78	是	否	是	女	150.00	80.00	150.00	82.00
71	否	否	是	女	139.00	70.00	135.00	70.00
72	是	否	是	女	156.00	94.00	160.00	98.00
70	否	否	是	女	129.00	80.00	130.00	82.00
94	是	否	是	女	90.00	60.00	92.00	70.00
69	否	否	是	女	96.00	60.00	94.00	62.00
85	是	否	是	女	119.00	89.00	120.00	86.00
67	否	否	是	女	139.00	80.00	138.00	78.00
70	否	否	是	女	130.00	70.00	130.00	76.00
77	否	否	是	女	93.00	74.00	95.00	68.00
70	是	否	是	女	135.00	78.00	134.00	81.00
66	否	否	是	女	140.00	89.00	139.00	86.00
81	是	否	是	女	138.00	85.00	139.00	87.00
77	是	否	是	女	122.00	90.00	124.00	96.00
67	是	否	是	女	110.00	68.00	115.00	70.00
73	是	否	是	女	130.00	80.00	130.00	70.00
68	否	否	是	女	127.00	80.00	125.00	78.00
70	否	否	是	女	155.00	90.00	160.00	95.00
67	否	否	是	女	130.00	80.00	136.00	80.00
74	否	否	是	女	150.00	92.00	148.00	93.00
90	否	是	是	女	139.00	83.00	138.00	80.00
78	是	是	是	女	130.00	89.00	125.00	84.00
79	是	是	是	女	139.00	89.00	135.00	85.00
70	是	否	是	女	125.00	80.00	120.00	75.00
68	是	否	是	女	110.00	73.00	115.00	78.00

69	是	否	是	女	135.00	83.00	139.00	87.00
85	否	否	是	女	111.00	79.00	114.00	82.00
73	是	否	是	女	122.00	62.00	120.00	60.00
70	否	否	是	女	149.00	88.00	146.00	82.00
84	是	是	是	女	150.00	78.00	146.00	80.00
70	是	否	是	女	117.00	86.00	120.00	78.00
71	是	否	是	女	175.00	115.00	170.00	110.00
67	是	否	是	女	163.00	91.00	158.00	90.00
67	否	否	是	女	110.00	70.00	115.00	70.00
79	是	否	是	女	130.00	70.00	125.00	65.00
87	是	否	是	女	120.00	80.00	122.00	80.00
67	否	否	是	女	139.00	76.00	136.00	74.00
80	否	是	是	女	158.00	79.00	171.00	80.00
68	否	否	是	女	100.00	60.00	104.00	60.00
97	否	否	是	女	128.00	74.00	130.00	75.00
76	是	否	是	女	152.00	88.00	156.00	88.00
76	是	是	是	女	130.00	80.00	135.00	79.00
72	是	否	是	女	146.00	96.00	150.00	100.00
66	是	否	否	女	150.00	89.00	158.00	90.00
72	是	否	是	女	120.00	76.00	130.00	80.00
83	是	否	是	女	122.00	75.00	120.00	70.00
79	否	否	是	女	138.00	62.00	134.00	68.00
70	是	否	是	女	143.00	88.00	145.00	89.00
70	是	是	是	女	140.00	78.00	146.00	80.00
73	是	是	是	女	142.00	90.00	144.00	100.00
67	否	否	是	女	99.00	71.00	98.00	66.00
70	是	否	是	女	125.00	83.00	129.00	81.00
68	否	否	是	女	140.00	82.00	138.00	84.00
75	否	否	是	女	130.00	70.00	126.00	78.00
68	是	否	是	女	120.00	80.00	123.00	68.00
69	是	否	是	女	176.00	96.00	180.00	100.00
82	否	否	是	女	145.00	86.00	150.00	90.00
71	否	是	是	女	139.00	77.00	140.00	78.00
72	否	否	是	女	116.00	70.00	113.00	71.00
68	否	否	是	女	121.00	62.00	134.00	72.00
77	是	是	是	女	148.00	80.00	156.00	82.00
80	是	否	是	女	142.00	82.00	150.00	86.00
80	是	否	是	女	139.00	89.00	138.00	90.00
77	否	否	是	女	130.00	70.00	140.00	80.00
69	否	否	是	女	135.00	86.00	138.00	89.00
79	否	否	是	女	110.00	70.00	120.00	80.00
69	是	否	是	女	149.00	90.00	150.00	89.00
75	是	是	是	女	131.00	83.00	130.00	80.00
71	否	是	是	女	108.00	70.00	110.00	70.00
67	否	否	是	女	138.00	80.00	136.00	78.00
67	是	否	是	女	120.00	75.00	120.00	72.00
81	否	是	是	女	131.00	81.00	135.00	85.00
67	否	否	是	女	139.00	87.00	139.00	88.00
68	否	否	是	女	110.00	60.00	110.00	66.00
69	是	否	是	女	165.00	85.00	170.00	90.00
74	是	是	是	女	135.00	75.00	140.00	70.00

68	是	否	是	女	135.00	85.00	140.00	96.00
67	是	否	是	女	132.00	86.00	130.00	85.00
71	是	否	是	女	138.00	78.00	158.00	80.00
67	否	否	是	女	130.00	80.00	139.00	85.00
69	是	是	是	女	172.00	100.00	172.00	97.00
73	否	否	是	女	130.00	75.00	134.00	78.00
74	否	否	是	女	132.00	66.00	136.00	70.00
66	是	是	是	女	118.00	78.00	122.00	80.00
84	是	否	是	女	152.00	80.00	154.00	94.00
86	否	否	是	女	139.00	89.00	140.00	90.00
71	否	否	是	女	120.00	66.00	125.00	70.00
77	是	否	是	女	130.00	82.00	135.00	85.00
73	是	否	是	女	125.00	84.00	130.00	70.00
76	否	否	是	女	153.00	98.00	141.00	92.00
69	否	是	是	女	118.00	78.00	122.00	80.00
70	否	否	是	女	126.00	76.00	130.00	78.00
68	是	否	是	女	145.00	80.00	150.00	82.00
67	是	否	是	女	100.00	60.00	104.00	60.00
69	否	否	是	女	120.00	68.00	128.00	68.00
72	是	否	是	女	120.00	70.00	128.00	70.00
78	是	否	是	女	138.00	90.00	140.00	90.00
75	是	否	是	女	125.00	70.00	136.00	70.00
79	否	否	是	女	130.00	89.00	132.00	92.00
85	否	否	是	女	136.00	80.00	140.00	86.00
84	否	否	是	女	143.00	78.00	143.00	79.00
70	否	否	是	女	138.00	70.00	146.00	74.00
66	否	否	是	女	100.00	60.00	110.00	70.00
67	是	否	是	女	132.00	80.00	135.00	83.00
74	否	是	是	女	121.00	70.00	128.00	71.00
69	否	否	是	女	120.00	72.00	126.00	80.00
69	是	是	是	女	140.00	80.00	144.00	82.00
77	否	否	是	女	136.00	80.00	130.00	82.00
77	否	否	是	女	170.00	90.00	162.00	90.00
88	是	是	是	女	155.00	90.00	168.00	96.00
81	否	否	是	女	104.00	68.00	108.00	68.00
75	否	否	是	女	140.00	84.00	150.00	88.00
70	否	否	是	女	150.00	98.00	163.00	106.00
68	是	否	是	女	120.00	80.00	124.00	86.00
66	是	是	是	女	130.00	80.00	136.00	72.00
70	否	否	是	女	132.00	98.00	130.00	100.00
75	否	否	是	女	136.00	88.00	140.00	88.00
88	是	否	是	女	150.00	79.00	134.00	77.00
75	否	否	是	女	139.00	85.00	140.00	81.00
68	否	否	是	女	116.00	78.00	120.00	80.00
66	否	否	否	女	130.00	82.00	136.00	88.00
68	是	否	是	女	125.00	70.00	130.00	75.00
69	否	否	是	女	90.00	60.00	90.00	60.00
72	是	是	是	女	150.00	80.00	153.00	82.00
74	是	否	是	女	130.00	82.00	135.00	73.00
79	否	否	是	女	90.00	60.00	92.00	65.00
67	否	否	是	女	138.00	86.00	142.00	88.00

69	是	否	是	女	120.00	68.00	127.00	70.00
67	否	是	是	女	118.00	78.00	120.00	82.00
73	是	是	是	女	128.00	70.00	130.00	70.00
68	是	否	是	女	143.00	78.00	165.00	82.00
71	否	是	是	女	158.00	98.00	160.00	100.00
78	是	是	是	女	152.00	70.00	150.00	70.00
67	是	否	是	女	152.00	98.00	153.00	96.00
91	是	否	是	女	209.00	114.00	200.00	100.00
73	否	是	是	女	120.00	80.00	118.00	78.00
70	是	否	是	女	128.00	74.00	130.00	76.00
69	否	否	是	女	150.00	110.00	145.00	100.00
72	是	是	是	女	160.00	100.00	160.00	100.00
92	是	否	是	女	134.00	72.00	130.00	68.00
76	是	否	是	女	130.00	78.00	136.00	80.00
73	是	否	是	女	142.00	80.00	144.00	82.00
69	否	否	是	女	130.00	68.00	132.00	68.00
68	否	否	是	女	125.00	66.00	138.00	84.00
69	否	否	是	女	136.00	78.00	139.00	89.00
68	否	否	是	女	116.00	70.00	120.00	72.00
70	是	否	是	女	130.00	80.00	128.00	70.00
66	是	是	是	女	140.00	60.00	135.00	70.00
72	是	是	是	女	146.00	88.00	149.00	90.00
78	是	否	是	女	164.00	92.00	168.00	93.00
69	是	否	是	女	110.00	66.00	112.00	70.00
70	否	否	是	女	108.00	76.00	110.00	80.00
71	否	否	是	女	120.00	70.00	124.00	70.00
67	是	否	是	女	138.00	70.00	142.00	76.00
71	否	否	是	女	130.00	80.00	138.00	82.00
77	否	否	是	女	130.00	82.00	135.00	80.00
76	是	否	是	女	135.00	75.00	138.00	80.00
99	是	否	是	女	103.00	72.00	105.00	70.00
79	是	否	是	女	144.00	84.00	148.00	88.00
70	是	否	是	女	137.00	70.00	136.00	74.00
77	否	否	是	女	135.00	60.00	130.00	60.00
71	是	是	是	女	130.00	86.00	120.00	80.00
69	是	否	是	女	145.00	90.00	148.00	98.00
67	否	否	是	女	118.00	60.00	120.00	62.00
68	否	否	是	女	100.00	68.00	104.00	68.00
79	是	否	是	女	132.00	83.00	132.00	80.00
69	否	否	是	女	146.00	78.00	153.00	81.00
73	否	否	是	女	136.00	79.00	139.00	83.00
67	否	否	是	女	128.00	79.00	119.00	80.00
72	是	是	是	女	130.00	84.00	132.00	85.00
75	是	是	是	女	140.00	85.00	145.00	80.00
69	否	否	是	女	135.00	80.00	138.00	84.00
73	否	否	是	女	140.00	88.00	141.00	90.00
67	否	否	是	女	110.00	70.00	128.00	82.00
66	是	否	是	女	140.00	82.00	148.00	82.00
72	是	否	是	女	142.00	85.00	140.00	76.00
84	是	否	是	女	146.00	84.00	148.00	89.00
71	否	否	是	女	136.00	86.00	138.00	86.00

76	否	否	是	女	110.00	70.00	118.00	76.00
68	否	否	是	女	136.00	86.00	138.00	88.00
82	否	否	是	女	136.00	80.00	140.00	80.00
70	否	否	是	女	132.00	70.00	134.00	74.00
79	是	否	是	女	128.00	89.00	130.00	90.00
79	是	否	是	女	130.00	76.00	135.00	78.00
67	是	是	是	女	140.00	80.00	140.00	80.00
67	否	是	是	女	118.00	82.00	120.00	83.00
85	是	否	是	女	128.00	68.00	130.00	68.00
67	否	否	是	女	128.00	69.00	139.00	72.00
89	否	否	是	女	145.00	78.00	142.00	70.00
86	是	否	是	女	141.00	61.00	138.00	70.00
66	是	是	是	女	130.00	70.00	132.00	71.00
77	是	否	是	女	150.00	92.00	154.00	94.00
84	否	否	是	女	120.00	72.00	130.00	80.00
72	否	否	是	女	130.00	70.00	135.00	80.00
69	否	否	是	女	120.00	70.00	125.00	72.00
70	否	否	是	女	130.00	70.00	134.00	74.00
80	否	否	是	女	140.00	90.00	140.00	90.00
99	是	是	是	女	151.00	84.00	149.00	85.00
90	是	否	是	女	140.00	80.00	139.00	78.00
71	是	否	是	女	130.00	80.00	144.00	82.00
69	是	否	是	女	140.00	90.00	140.00	90.00
68	否	否	是	女	150.00	80.00	140.00	84.00
73	否	否	是	女	100.00	60.00	105.00	62.00
75	是	否	是	女	120.00	87.00	130.00	88.00
93	是	否	是	女	122.00	86.00	130.00	80.00
72	是	否	是	女	140.00	80.00	130.00	82.00
69	是	否	是	女	135.00	72.00	138.00	75.00
71	是	是	是	女	142.00	88.00	141.00	80.00
68	是	否	是	女	130.00	80.00	135.00	88.00
68	否	否	是	女	136.00	90.00	140.00	90.00
97	是	否	是	女	146.00	82.00	140.00	80.00
74	否	否	是	女	137.00	71.00	139.00	83.00
96	否	否	是	女	127.00	67.00	130.00	70.00
72	是	否	是	女	155.00	90.00	160.00	100.00
67	是	否	是	女	152.00	92.00	150.00	89.00
83	是	是	是	女	100.00	62.00	102.00	64.00
73	否	否	是	女	130.00	80.00	135.00	82.00
66	否	否	否	女	130.00	85.00	132.00	87.00
73	否	否	是	女	130.00	80.00	135.00	86.00
89	是	否	是	女	135.00	82.00	138.00	88.00
68	否	否	是	女	120.00	70.00	121.00	75.00
66	否	否	是	女	154.00	89.00	160.00	92.00
85	是	否	是	女	131.00	78.00	129.00	79.00
71	是	是	是	女	150.00	90.00	144.00	80.00
70	否	否	是	女	137.00	84.00	139.00	88.00
77	是	否	是	女	120.00	76.00	123.00	79.00
68	是	否	是	女	135.00	82.00	140.00	88.00
89	是	否	是	女	140.00	75.00	140.00	80.00
69	否	否	是	女	110.00	70.00	117.00	74.00

75	否	否	是	女	145.00	98.00	148.00	98.00
71	是	否	是	女	138.00	70.00	140.00	72.00
90	是	否	是	女	120.00	60.00	122.00	63.00
69	是	否	是	女	130.00	80.00	133.00	98.00
70	否	否	是	女	140.00	80.00	136.00	78.00
95	否	是	是	女	126.00	70.00	132.00	74.00
89	是	否	是	女	140.00	89.00	142.00	86.00
67	否	否	是	女	160.00	92.00	162.00	92.00
72	是	否	是	女	134.00	80.00	139.00	74.00
69	是	否	是	女	137.00	84.00	139.00	80.00
74	是	是	是	女	142.00	82.00	140.00	80.00
73	否	否	是	女	130.00	70.00	138.00	76.00
70	是	否	是	女	138.00	88.00	127.00	86.00
66	是	是	是	女	154.00	86.00	160.00	90.00
70	否	是	是	女	135.00	86.00	139.00	89.00
70	否	否	是	女	125.00	76.00	130.00	82.00
77	是	否	是	女	145.00	72.00	128.00	75.00
71	是	否	是	女	132.00	70.00	130.00	68.00
82	是	否	是	女	130.00	80.00	135.00	85.00
68	否	否	是	女	120.00	70.00	122.00	74.00
67	是	否	是	女	156.00	88.00	160.00	90.00
70	是	否	是	女	130.00	60.00	135.00	70.00
74	是	是	是	女	120.00	70.00	120.00	77.00
83	是	否	是	女	140.00	81.00	139.00	74.00
72	是	否	是	女	120.00	80.00	118.00	60.00
66	是	否	是	女	135.00	92.00	139.00	90.00
69	否	否	是	女	130.00	70.00	139.00	85.00
77	是	否	是	女	130.00	71.00	132.00	72.00
66	是	否	否	女	139.00	89.00	135.00	85.00
72	是	是	是	女	144.00	78.00	150.00	80.00
75	否	是	是	女	120.00	60.00	115.00	70.00
68	是	否	是	女	135.00	86.00	139.00	89.00
92	是	否	是	女	100.00	68.00	102.00	70.00
81	是	否	是	女	120.00	70.00	126.00	86.00
70	否	否	是	女	120.00	80.00	125.00	81.00
67	是	否	是	女	142.00	82.00	142.00	83.00
71	否	否	是	女	139.00	87.00	137.00	88.00
70	否	否	是	女	110.00	70.00	115.00	75.00
66	是	否	是	女	128.00	65.00	130.00	70.00
68	是	否	是	女	120.00	80.00	122.00	82.00
80	是	否	是	女	138.00	62.00	138.00	64.00
80	是	否	是	女	125.00	80.00	113.00	77.00
69	是	是	是	女	129.00	80.00	137.00	84.00
73	否	否	是	女	140.00	90.00	140.00	96.00
68	否	否	是	女	170.00	100.00	180.00	90.00
72	是	否	是	女	160.00	89.00	162.00	90.00
71	否	是	是	女	123.00	74.00	130.00	80.00
68	是	否	是	女	120.00	88.00	125.00	89.00
68	否	否	是	女	164.00	104.00	163.00	106.00
67	是	否	是	女	127.00	64.00	139.00	66.00
69	是	是	是	女	120.00	80.00	121.00	81.00

73	是	否	是	女	138.00	86.00	148.00	97.00
66	是	否	是	女	125.00	70.00	130.00	70.00
67	否	否	是	女	161.00	105.00	176.00	102.00
86	是	是	是	女	140.00	70.00	138.00	70.00
71	是	否	是	女	180.00	110.00	182.00	114.00
67	是	是	是	女	145.00	80.00	145.00	83.00
90	是	否	是	女	126.00	78.00	130.00	82.00
71	是	否	是	女	164.00	80.00	168.00	82.00
83	是	否	是	女	135.00	78.00	139.00	82.00
89	是	否	是	女	160.00	80.00	150.00	70.00
73	否	否	是	女	150.00	89.00	150.00	89.00
77	是	是	是	女	138.00	70.00	136.00	74.00
73	是	是	是	女	128.00	70.00	130.00	70.00
82	是	是	是	女	140.00	80.00	143.00	81.00
76	否	否	是	女	148.00	80.00	150.00	82.00
81	是	否	是	女	160.00	92.00	162.00	94.00
66	否	否	是	女	146.00	78.00	144.00	76.00
72	是	否	是	女	134.00	80.00	130.00	78.00
78	否	否	是	女	124.00	74.00	130.00	74.00
81	是	否	是	女	136.00	84.00	138.00	86.00
81	是	否	是	女	138.00	90.00	140.00	77.00
74	否	否	是	女	136.00	84.00	138.00	86.00
75	否	否	是	女	116.00	70.00	120.00	74.00
71	是	否	是	女	130.00	80.00	136.00	84.00
73	否	否	是	女	167.00	92.00	177.00	90.00
87	是	否	是	女	138.00	60.00	144.00	65.00
74	否	否	是	女	130.00	80.00	132.00	82.00
70	否	否	是	女	130.00	80.00	135.00	82.00
72	是	否	是	女	170.00	90.00	170.00	100.00
70	是	是	是	女	138.00	80.00	140.00	82.00
72	否	否	是	女	136.00	79.00	130.00	77.00
74	是	否	是	女	160.00	80.00	162.00	80.00
82	是	否	是	女	135.00	80.00	142.00	82.00
68	是	否	是	女	136.00	80.00	140.00	84.00
72	否	否	是	女	110.00	69.00	133.00	60.00
68	否	否	是	女	109.00	70.00	114.00	75.00
66	否	否	是	女	138.00	80.00	140.00	80.00
71	否	否	是	女	120.00	60.00	125.00	65.00
75	否	否	是	女	132.00	84.00	130.00	82.00
69	是	是	是	女	138.00	85.00	136.00	82.00
78	是	否	是	女	140.00	80.00	142.00	86.00
77	否	否	是	女	132.00	90.00	130.00	90.00
68	是	否	是	女	178.00	99.00	180.00	98.00
69	否	否	是	女	106.00	70.00	118.00	70.00
67	是	否	是	女	168.00	100.00	162.00	100.00
70	否	否	是	女	130.00	90.00	135.00	75.00
86	是	否	是	女	145.00	75.00	150.00	80.00
73	是	否	是	女	150.00	90.00	146.00	88.00
70	否	是	是	女	160.00	90.00	155.00	89.00
66	是	否	是	女	126.00	72.00	128.00	75.00
66	否	否	是	女	138.00	89.00	128.00	66.00

86	否	否	是	女	130.00	80.00	134.00	82.00
72	是	是	是	女	120.00	70.00	116.00	72.00
74	是	是	是	女	142.00	78.00	145.00	80.00
73	否	否	是	女	137.00	83.00	130.00	79.00
68	否	否	是	女	120.00	80.00	127.00	76.00
74	是	是	是	女	130.00	79.00	138.00	90.00
71	否	否	是	女	127.00	68.00	139.00	79.00
71	是	否	是	女	125.00	60.00	137.00	63.00
70	否	否	是	女	120.00	85.00	118.00	70.00
68	否	否	是	女	130.00	80.00	132.00	80.00
68	否	否	是	女	126.00	70.00	130.00	72.00
76	否	是	是	女	115.00	71.00	118.00	70.00
73	是	否	是	女	132.00	85.00	135.00	86.00
70	否	否	是	女	100.00	72.00	104.00	74.00
69	否	否	是	女	118.00	70.00	120.00	80.00
79	否	否	是	女	106.00	70.00	110.00	72.00
72	否	否	是	女	130.00	76.00	138.00	80.00
69	否	否	是	女	108.00	70.00	110.00	78.00
71	否	否	是	女	108.00	66.00	110.00	66.00
91	否	否	是	女	155.00	82.00	156.00	84.00
81	是	否	是	女	136.00	74.00	134.00	72.00
68	否	否	是	女	120.00	68.00	123.00	74.00
71	是	否	是	女	135.00	70.00	139.00	74.00
69	是	是	是	女	158.00	78.00	160.00	80.00
74	是	否	是	女	140.00	76.00	147.00	78.00
71	否	否	是	女	140.00	90.00	138.00	88.00
83	是	否	是	女	130.00	70.00	130.00	75.00
85	否	否	是	女	140.00	80.00	140.00	88.00
72	否	否	是	女	130.00	70.00	138.00	72.00
66	是	否	是	女	173.00	105.00	186.00	112.00
72	否	否	是	女	106.00	70.00	108.00	73.00
88	是	否	是	女	148.00	90.00	150.00	88.00
68	是	是	是	女	159.00	81.00	158.00	78.00
88	是	是	是	女	136.00	80.00	142.00	86.00
68	否	否	是	女	132.00	89.00	139.00	74.00
66	否	否	是	女	143.00	105.00	139.00	96.00
73	是	否	是	女	120.00	70.00	124.00	75.00
69	是	否	是	女	114.00	75.00	120.00	76.00
86	是	否	是	女	139.00	89.00	138.00	85.00
83	否	否	是	女	146.00	70.00	150.00	75.00
92	是	是	是	女	109.00	60.00	110.00	62.00
66	否	否	是	女	142.00	102.00	145.00	97.00
69	是	是	是	女	142.00	90.00	140.00	90.00
70	否	否	是	女	130.00	89.00	135.00	89.00
76	否	否	是	女	138.00	69.00	136.00	69.00
70	是	否	是	女	119.00	79.00	138.00	86.00
70	是	否	是	女	120.00	72.00	126.00	74.00
66	否	否	是	女	140.00	80.00	140.00	80.00
75	是	否	是	女	128.00	86.00	132.00	88.00
72	是	否	是	女	136.00	80.00	139.00	83.00
69	否	否	是	女	137.00	87.00	139.00	86.00

69	否	否	是	女	118.00	68.00	124.00	70.00
71	是	是	是	女	117.00	81.00	120.00	84.00
77	是	是	是	女	145.00	87.00	148.00	89.00
68	是	是	是	女	160.00	78.00	160.00	80.00
69	否	否	是	女	140.00	68.00	140.00	69.00
75	是	否	是	女	134.00	65.00	144.00	68.00
70	是	是	是	女	135.00	88.00	140.00	89.00
67	否	否	是	女	136.00	87.00	139.00	80.00
69	是	否	是	女	130.00	80.00	135.00	85.00
68	否	否	是	女	139.00	80.00	150.00	81.00
68	是	否	是	女	144.00	72.00	142.00	74.00
71	否	否	是	女	145.00	88.00	154.00	93.00
88	否	否	是	女	110.00	71.00	112.00	72.00
79	否	否	是	女	162.00	90.00	162.00	87.00
67	否	否	是	女	138.00	78.00	142.00	80.00
71	是	否	是	女	118.00	86.00	120.00	88.00
79	是	否	是	女	110.00	66.00	118.00	68.00
80	是	否	是	女	138.00	80.00	139.00	82.00
70	是	否	是	女	139.00	70.00	136.00	76.00
79	否	否	是	女	114.00	74.00	118.00	80.00
68	是	否	是	女	136.00	90.00	140.00	90.00
68	否	否	是	女	120.00	70.00	125.00	72.00
72	否	否	是	女	135.00	80.00	142.00	84.00
75	是	是	是	女	137.00	89.00	140.00	90.00
69	是	否	是	女	148.00	90.00	147.00	88.00
71	否	否	是	女	168.00	92.00	165.00	97.00
71	否	否	是	女	159.00	91.00	157.00	84.00
68	是	否	是	女	138.00	80.00	135.00	70.00
69	否	否	是	女	135.00	75.00	138.00	78.00
75	是	否	是	女	120.00	70.00	122.00	72.00
69	是	否	是	女	148.00	80.00	144.00	80.00
71	是	否	是	女	120.00	80.00	122.00	82.00
66	是	否	是	女	135.00	80.00	142.00	82.00
72	是	是	是	女	140.00	90.00	142.00	91.00
89	否	否	是	女	134.00	70.00	136.00	72.00
66	否	是	是	女	106.00	73.00	88.00	56.00
72	否	否	是	女	118.00	89.00	110.00	88.00
66	否	否	否	女	130.00	82.00	130.00	90.00
67	否	否	是	女	130.00	90.00	150.00	110.00
73	否	否	是	女	112.00	60.00	118.00	64.00
78	是	否	是	女	139.00	90.00	135.00	88.00
67	是	否	是	女	130.00	80.00	135.00	82.00
71	否	否	是	女	132.00	70.00	130.00	72.00
67	是	否	是	女	139.00	72.00	138.00	74.00
88	是	否	是	女	130.00	87.00	132.00	89.00
71	是	否	是	女	139.00	76.00	142.00	77.00
66	是	否	是	女	132.00	80.00	136.00	84.00
70	否	否	是	女	130.00	60.00	135.00	62.00
74	是	否	是	女	114.00	76.00	118.00	77.00
77	否	否	是	女	148.00	80.00	160.00	90.00
76	是	否	是	女	123.00	88.00	120.00	90.00

69	否	否	是	女	122.00	78.00	120.00	80.00
84	是	是	是	女	132.00	84.00	125.00	79.00
73	否	是	是	女	160.00	90.00	155.00	80.00
67	否	否	是	女	144.00	80.00	140.00	80.00
76	是	是	是	女	164.00	90.00	170.00	86.00
72	是	否	是	女	138.00	78.00	140.00	85.00
76	否	否	是	女	128.00	79.00	139.00	80.00
72	是	否	是	女	140.00	78.00	140.00	80.00
72	否	是	是	女	127.00	78.00	130.00	82.00
68	否	否	是	女	130.00	80.00	125.00	76.00
70	否	否	是	女	139.00	70.00	133.00	77.00
77	是	否	是	女	130.00	80.00	135.00	82.00
77	是	否	是	女	130.00	77.00	146.00	80.00
70	否	否	是	女	109.00	72.00	112.00	74.00
67	否	否	是	女	145.00	99.00	140.00	89.00
67	是	是	是	女	135.00	70.00	137.00	73.00
71	否	否	是	女	116.00	78.00	120.00	78.00
73	是	否	是	女	150.00	80.00	160.00	80.00
79	是	否	是	女	134.00	88.00	140.00	88.00
81	否	是	是	女	140.00	79.00	145.00	85.00
70	否	否	是	女	135.00	80.00	139.00	84.00
93	是	否	是	女	118.00	62.00	120.00	62.00
69	是	是	是	女	133.00	80.00	147.00	85.00
76	否	否	是	女	140.00	86.00	145.00	90.00
77	是	是	是	女	127.00	70.00	137.00	78.00
71	否	否	是	女	116.00	70.00	128.00	76.00
88	是	否	是	女	132.00	78.00	138.00	98.00
71	是	否	是	女	116.00	64.00	118.00	69.00
86	是	否	是	女	142.00	64.00	148.00	67.00
73	是	否	是	女	163.00	76.00	166.00	79.00
67	否	否	是	女	130.00	78.00	138.00	82.00
76	是	否	是	女	151.00	85.00	148.00	86.00
70	否	否	是	女	138.00	86.00	130.00	80.00
72	否	否	是	女	120.00	80.00	125.00	85.00
71	是	否	是	女	136.00	80.00	140.00	84.00
72	是	否	是	女	128.00	70.00	130.00	70.00
72	是	是	是	女	128.00	80.00	132.00	80.00
76	是	否	是	女	122.00	69.00	124.00	69.00
70	否	否	是	女	133.00	87.00	136.00	89.00
82	否	否	是	女	120.00	70.00	122.00	72.00
72	是	否	是	女	132.00	83.00	130.00	80.00
72	否	否	是	女	165.00	85.00	170.00	90.00
66	否	否	是	女	158.00	98.00	160.00	100.00
67	否	否	是	女	108.00	68.00	110.00	70.00
72	否	否	是	女	110.00	60.00	105.00	60.00
70	否	否	是	女	130.00	70.00	138.00	78.00
67	是	否	是	女	130.00	82.00	135.00	85.00
90	是	是	是	女	156.00	83.00	139.00	89.00
72	否	否	是	女	140.00	80.00	144.00	86.00
72	是	否	是	女	123.00	82.00	120.00	80.00
67	是	否	是	女	138.00	96.00	142.00	98.00

66	否	否	是	女	120.00	70.00	125.00	70.00
72	是	否	是	女	112.00	60.00	110.00	62.00
66	是	否	是	女	134.00	76.00	138.00	80.00
69	是	否	是	女	136.00	82.00	139.00	87.00
68	否	是	是	女	136.00	82.00	138.00	83.00
74	是	否	是	女	120.00	80.00	122.00	78.00
68	是	否	是	女	129.00	76.00	127.00	76.00
67	否	否	是	女	138.00	79.00	120.00	80.00
82	是	是	是	女	170.00	92.00	150.00	90.00
66	是	否	是	女	139.00	79.00	137.00	79.00
83	否	否	是	女	134.00	85.00	136.00	86.00
77	是	否	是	女	128.00	70.00	130.00	70.00
67	否	否	是	女	135.00	92.00	100.00	60.00
92	是	否	是	女	198.00	108.00	187.00	111.00
78	是	是	是	女	134.00	70.00	136.00	78.00
71	否	否	是	女	130.00	81.00	121.00	88.00
71	否	否	是	女	110.00	65.00	115.00	70.00
70	否	否	是	女	120.00	78.00	125.00	85.00
66	是	是	是	女	138.00	84.00	140.00	84.00
77	是	是	是	女	135.00	69.00	138.00	72.00
68	是	否	是	女	134.00	84.00	139.00	89.00
67	是	否	是	女	147.00	72.00	152.00	75.00
69	否	否	是	女	130.00	80.00	138.00	79.00
69	否	是	是	女	140.00	80.00	135.00	80.00
90	否	否	是	女	90.00	60.00	95.00	68.00
71	是	是	是	女	118.00	85.00	123.00	87.00
74	是	是	是	女	150.00	76.00	156.00	83.00
69	否	否	是	女	120.00	76.00	111.00	78.00
84	否	否	是	女	135.00	85.00	139.00	85.00
89	是	是	是	女	112.00	71.00	110.00	68.00
70	否	否	是	女	138.00	78.00	138.00	80.00
70	否	否	是	女	125.00	78.00	126.00	79.00
79	否	否	是	女	160.00	100.00	160.00	90.00
71	是	否	是	女	164.00	82.00	169.00	84.00
67	否	否	是	女	119.00	73.00	120.00	80.00
80	是	否	是	女	156.00	100.00	160.00	100.00
74	否	否	是	女	155.00	90.00	160.00	90.00
67	是	是	是	女	125.00	78.00	130.00	80.00
73	是	否	是	女	120.00	62.00	118.00	60.00
73	是	是	是	女	139.00	75.00	134.00	70.00
81	否	否	是	女	122.00	64.00	124.00	65.00
77	是	否	是	女	139.00	71.00	130.00	89.00
67	否	否	是	女	116.00	69.00	120.00	70.00
92	是	否	是	女	143.00	67.00	139.00	89.00
72	是	否	是	女	130.00	70.00	139.00	80.00
73	是	否	是	女	132.00	82.00	130.00	80.00
78	是	否	是	女	140.00	80.00	144.00	82.00
70	是	否	是	女	122.00	78.00	124.00	80.00
76	是	是	是	女	139.00	68.00	136.00	64.00
87	是	否	是	女	148.00	80.00	144.00	68.00
74	是	否	是	女	150.00	90.00	156.00	90.00

67	否	否	是	女	115.00	70.00	118.00	72.00
74	是	否	是	女	140.00	80.00	138.00	86.00
72	是	否	是	女	138.00	78.00	139.00	79.00
85	是	否	是	女	162.00	108.00	160.00	106.00
88	是	否	是	女	132.00	80.00	139.00	69.00
78	是	否	是	女	118.00	72.00	120.00	76.00
69	否	否	是	女	138.00	84.00	139.00	85.00
72	否	否	是	女	112.00	76.00	110.00	74.00
73	是	否	是	女	130.00	80.00	125.00	80.00
73	否	否	是	女	124.00	73.00	127.00	76.00
69	否	否	是	女	128.00	72.00	128.00	74.00
77	是	否	是	女	140.00	90.00	141.00	92.00
72	是	否	是	女	147.00	82.00	148.00	85.00
73	否	否	是	女	127.00	83.00	139.00	88.00
76	否	否	是	女	139.00	80.00	140.00	82.00
66	是	是	是	女	136.00	80.00	139.00	80.00
79	是	否	是	女	119.00	73.00	117.00	76.00
76	是	是	是	女	138.00	88.00	139.00	89.00
76	否	否	是	女	152.00	90.00	144.00	88.00
81	是	是	是	女	130.00	78.00	138.00	80.00
77	是	否	是	女	135.00	72.00	138.00	70.00
74	是	否	是	女	150.00	68.00	150.00	70.00
72	否	否	是	女	158.00	90.00	158.00	92.00
72	否	否	是	女	120.00	80.00	122.00	82.00
74	是	否	是	女	130.00	80.00	138.00	80.00
78	是	是	是	女	130.00	78.00	130.00	80.00
76	否	否	是	女	134.00	90.00	138.00	90.00
72	否	否	是	女	135.00	85.00	138.00	88.00
74	是	是	是	女	125.00	80.00	127.00	85.00
70	是	否	是	女	130.00	60.00	135.00	60.00
76	是	否	是	女	147.00	81.00	145.00	79.00
67	是	否	是	女	132.00	70.00	138.00	74.00
75	是	否	是	女	130.00	80.00	140.00	82.00
71	否	否	是	女	130.00	70.00	135.00	80.00
72	是	否	是	女	138.00	78.00	140.00	80.00
80	是	否	是	女	136.00	82.00	134.00	84.00
77	否	否	是	女	135.00	88.00	139.00	90.00
66	否	否	是	女	105.00	85.00	110.00	85.00
76	是	否	是	女	140.00	85.00	148.00	80.00
69	否	否	是	女	116.00	66.00	120.00	70.00
72	是	否	是	女	136.00	77.00	139.00	80.00
75	是	否	是	女	130.00	78.00	130.00	80.00
73	是	否	是	女	153.00	87.00	152.00	86.00
71	否	否	是	女	140.00	90.00	145.00	80.00
76	是	否	是	女	135.00	80.00	139.00	88.00
80	是	否	是	女	118.00	72.00	120.00	70.00
72	否	否	是	女	120.00	84.00	126.00	90.00
69	是	否	是	女	150.00	90.00	155.00	92.00
71	否	否	是	女	116.00	80.00	122.00	80.00
81	否	否	是	女	153.00	81.00	151.00	85.00
68	否	否	是	女	120.00	80.00	138.00	86.00

73	否	否	是	女	130.00	72.00	134.00	76.00
69	是	是	是	女	130.00	70.00	142.00	80.00
82	是	否	是	女	140.00	83.00	145.00	85.00
84	是	否	是	女	178.00	73.00	170.00	78.00
66	是	否	是	女	135.00	89.00	130.00	86.00
78	否	否	是	女	126.00	89.00	125.00	85.00
68	否	否	是	女	120.00	80.00	128.00	82.00
70	是	否	是	女	120.00	70.00	118.00	66.00
66	否	否	是	女	150.00	100.00	160.00	95.00
66	是	否	是	女	139.00	80.00	136.00	82.00
68	否	否	是	女	148.00	70.00	146.00	72.00
66	否	否	是	女	153.00	104.00	154.00	99.00
73	是	是	是	女	130.00	82.00	135.00	85.00
87	是	是	是	女	126.00	74.00	130.00	80.00
73	否	否	是	女	138.00	78.00	140.00	88.00
80	是	否	是	女	165.00	83.00	166.00	90.00
66	否	否	是	女	100.00	60.00	110.00	70.00
66	否	否	是	女	130.00	76.00	132.00	79.00
95	是	是	是	女	130.00	85.00	130.00	80.00
69	否	否	是	女	126.00	78.00	130.00	80.00
71	是	否	是	女	120.00	73.00	122.00	76.00
70	否	否	是	女	130.00	80.00	125.00	88.00
71	否	否	是	女	116.00	70.00	120.00	70.00
74	否	否	是	女	138.00	90.00	140.00	90.00
72	是	否	是	女	135.00	70.00	138.00	74.00
72	是	是	是	女	140.00	80.00	135.00	78.00
82	是	否	是	女	171.00	75.00	170.00	73.00
71	否	是	是	女	105.00	70.00	110.00	70.00
69	是	否	是	女	142.00	80.00	140.00	80.00
73	是	否	是	女	127.00	76.00	136.00	81.00
86	否	否	是	女	130.00	70.00	120.00	80.00
70	否	是	是	女	148.00	80.00	150.00	80.00
88	否	否	是	女	110.00	79.00	125.00	85.00
68	是	否	是	女	126.00	75.00	132.00	74.00
74	否	否	是	女	124.00	80.00	126.00	84.00
69	是	否	是	女	132.00	83.00	130.00	80.00
68	否	是	是	女	139.00	71.00	139.00	84.00
72	否	否	是	女	172.00	91.00	173.00	85.00
71	否	否	是	女	136.00	84.00	134.00	88.00
77	是	否	是	女	146.00	66.00	148.00	70.00
77	是	否	是	女	170.00	89.00	175.00	86.00
67	是	否	是	女	136.00	86.00	139.00	80.00
82	是	否	是	女	144.00	68.00	138.00	72.00
69	否	否	是	女	135.00	64.00	136.00	65.00
78	否	否	是	女	120.00	80.00	130.00	70.00
66	是	否	是	女	144.00	92.00	146.00	92.00
67	否	否	是	女	96.00	60.00	100.00	60.00
66	否	否	是	女	120.00	73.00	125.00	70.00
74	是	否	是	女	150.00	90.00	152.00	92.00
68	否	否	是	女	110.00	60.00	115.00	69.00
70	否	是	是	女	130.00	70.00	132.00	71.00

73	否	否	是	女	140.00	78.00	142.00	80.00
68	是	否	是	女	167.00	90.00	153.00	94.00
68	是	否	是	女	128.00	70.00	135.00	74.00
74	是	是	是	女	138.00	76.00	128.00	70.00
70	是	否	是	女	140.00	80.00	142.00	84.00
71	否	否	是	女	110.00	70.00	115.00	76.00
67	是	否	是	女	130.00	90.00	135.00	85.00
71	否	是	是	女	135.00	70.00	138.00	65.00
70	否	否	是	女	132.00	63.00	138.00	67.00
80	否	否	是	女	120.00	76.00	117.00	73.00
66	是	是	是	女	157.00	78.00	160.00	85.00
73	是	否	是	女	136.00	84.00	139.00	87.00
75	否	否	是	女	136.00	74.00	139.00	76.00
67	否	是	是	女	110.00	65.00	100.00	60.00
67	否	否	是	女	140.00	90.00	139.00	89.00
73	是	是	是	女	140.00	86.00	140.00	80.00
76	是	否	是	女	120.00	88.00	126.00	90.00
88	是	否	是	女	124.00	62.00	126.00	66.00
68	是	否	是	女	130.00	80.00	134.00	82.00
67	否	否	是	女	122.00	74.00	120.00	70.00
67	否	是	是	女	139.00	89.00	140.00	87.00
73	是	否	是	女	130.00	72.00	134.00	76.00
66	是	否	是	女	139.00	92.00	158.00	93.00
79	否	否	是	女	127.00	77.00	130.00	80.00
72	否	否	是	女	128.00	84.00	136.00	90.00
72	是	否	是	女	142.00	80.00	146.00	82.00
67	否	否	是	女	151.00	89.00	150.00	88.00
78	否	否	是	女	178.00	98.00	180.00	100.00
73	否	否	是	女	120.00	72.00	122.00	72.00
75	是	是	是	女	95.00	61.00	105.00	65.00
70	否	否	是	女	135.00	75.00	139.00	80.00
76	是	是	是	女	130.00	91.00	134.00	92.00
73	否	否	是	女	116.00	78.00	120.00	80.00
79	是	否	是	女	156.00	90.00	158.00	92.00
83	是	否	是	女	140.00	89.00	135.00	86.00
76	是	否	是	女	160.00	88.00	162.00	90.00
66	否	否	是	女	125.00	80.00	130.00	82.00
89	否	否	是	女	141.00	73.00	140.00	87.00
87	否	否	是	女	150.00	95.00	145.00	90.00
70	是	否	是	女	130.00	65.00	120.00	60.00
74	否	否	是	女	118.00	60.00	120.00	64.00
69	是	否	是	女	160.00	92.00	155.00	90.00
66	否	否	否	女	118.00	70.00	120.00	72.00
70	否	否	是	女	130.00	75.00	128.00	80.00
67	是	否	是	女	135.00	86.00	138.00	87.00
82	是	否	是	女	134.00	80.00	138.00	84.00
68	否	否	是	女	115.00	63.00	110.00	60.00
86	是	否	是	女	124.00	68.00	128.00	68.00
75	是	否	是	女	120.00	78.00	120.00	80.00
77	是	否	是	女	132.00	74.00	133.00	75.00
89	否	否	是	女	150.00	66.00	140.00	62.00

70	是	否	是	女	149.00	89.00	150.00	90.00
87	否	否	是	女	129.00	84.00	130.00	80.00
75	否	否	是	女	120.00	70.00	125.00	68.00
70	否	是	是	女	110.00	70.00	112.00	75.00
68	否	是	是	女	127.00	78.00	139.00	83.00
78	是	是	是	女	136.00	70.00	140.00	70.00
75	否	是	是	女	120.00	75.00	125.00	80.00
69	否	否	是	女	138.00	88.00	140.00	90.00
67	否	否	是	女	128.00	78.00	130.00	80.00
75	否	否	是	女	145.00	80.00	140.00	90.00
70	否	否	是	女	106.00	60.00	110.00	62.00
67	否	否	是	女	136.00	88.00	136.00	84.00
75	否	否	是	女	108.00	60.00	112.00	75.00
84	否	否	是	女	127.00	70.00	129.00	67.00
71	否	否	是	女	138.00	72.00	140.00	75.00
75	是	否	是	女	137.00	80.00	130.00	78.00
76	否	否	是	女	90.00	60.00	91.00	65.00
72	是	否	是	女	116.00	62.00	118.00	62.00
75	是	否	是	女	120.00	70.00	128.00	70.00
69	否	否	是	女	136.00	72.00	139.00	80.00
73	是	否	是	女	139.00	89.00	136.00	80.00
73	是	否	是	女	128.00	70.00	130.00	70.00
79	是	否	是	女	140.00	90.00	140.00	85.00
72	否	否	是	女	146.00	98.00	150.00	100.00
78	否	否	是	女	115.00	82.00	120.00	85.00
70	是	是	是	女	167.00	92.00	171.00	90.00
75	否	否	是	女	127.00	85.00	140.00	86.00
72	是	是	是	女	135.00	82.00	139.00	85.00
66	否	否	否	女	110.00	78.00	112.00	78.00
72	是	是	是	女	140.00	88.00	153.00	90.00
77	否	否	是	女	162.00	90.00	162.00	86.00
73	是	否	是	女	139.00	75.00	138.00	72.00
71	是	是	是	女	106.00	80.00	120.00	80.00
67	否	否	是	女	127.00	88.00	130.00	84.00
87	是	否	是	女	140.00	67.00	160.00	84.00
71	是	是	是	女	154.00	81.00	156.00	92.00
83	否	否	是	女	126.00	80.00	124.00	82.00
74	否	否	是	女	155.00	87.00	160.00	90.00
72	是	否	是	女	138.00	79.00	139.00	80.00
67	否	否	是	女	154.00	90.00	158.00	96.00
89	否	否	是	女	140.00	80.00	142.00	85.00
68	否	否	是	女	118.00	68.00	122.00	70.00
66	是	是	是	女	110.00	68.00	118.00	74.00
69	是	是	是	女	100.00	60.00	115.00	70.00
70	否	否	是	女	136.00	90.00	147.00	93.00
71	否	否	是	女	127.00	68.00	125.00	70.00
70	否	是	是	女	100.00	62.00	100.00	68.00
80	是	否	是	女	124.00	76.00	126.00	74.00
67	否	否	是	女	114.00	70.00	120.00	80.00
84	是	否	是	女	160.00	95.00	170.00	94.00
78	是	否	是	女	145.00	75.00	151.00	78.00

74	是	是	是	女	162.00	98.00	159.00	92.00
70	是	否	是	女	116.00	74.00	120.00	80.00
78	是	否	是	女	136.00	60.00	138.00	60.00
85	否	否	是	女	105.00	74.00	100.00	71.00
68	否	否	是	女	129.00	76.00	132.00	79.00
72	否	否	是	女	139.00	80.00	138.00	80.00
74	是	否	是	女	132.00	74.00	136.00	80.00
83	否	否	是	女	140.00	80.00	132.00	78.00
74	否	否	是	女	130.00	78.00	132.00	78.00
70	是	否	是	女	136.00	77.00	138.00	79.00
69	否	否	是	女	114.00	78.00	115.00	80.00
73	否	否	是	女	163.00	100.00	165.00	100.00
70	是	否	是	女	130.00	80.00	140.00	90.00
80	否	否	是	女	120.00	89.00	125.00	91.00
70	是	否	是	女	130.00	70.00	135.00	86.00
73	是	否	是	女	140.00	85.00	139.00	87.00
69	是	否	是	女	140.00	85.00	152.00	90.00
70	是	是	是	女	150.00	90.00	150.00	88.00
76	否	否	是	女	118.00	80.00	120.00	80.00
73	否	是	是	女	110.00	70.00	116.00	70.00
68	否	否	是	女	120.00	72.00	118.00	72.00
95	否	否	是	女	172.00	80.00	174.00	82.00
71	是	是	是	女	120.00	80.00	121.00	86.00
90	是	是	是	女	108.00	60.00	108.00	62.00
73	是	否	是	女	135.00	72.00	138.00	77.00
84	是	否	是	女	127.00	75.00	130.00	70.00
74	否	否	是	女	110.00	62.00	110.00	60.00
71	否	否	是	女	135.00	70.00	144.00	72.00
66	否	否	是	女	138.00	80.00	133.00	75.00
76	是	否	是	女	142.00	66.00	140.00	68.00
74	否	否	是	女	146.00	88.00	148.00	90.00
67	是	否	是	女	126.00	70.00	139.00	79.00
82	否	否	是	女	130.00	78.00	139.00	80.00
71	否	否	是	女	120.00	78.00	122.00	76.00
81	是	是	是	女	132.00	82.00	123.00	76.00
73	否	否	是	女	120.00	72.00	122.00	70.00
70	是	否	是	女	142.00	76.00	144.00	75.00
74	否	否	是	女	114.00	70.00	120.00	80.00
68	是	是	是	女	126.00	60.00	120.00	70.00
67	是	是	是	女	129.00	70.00	139.00	74.00
69	是	否	是	女	139.00	82.00	132.00	80.00
68	是	否	是	女	150.00	70.00	152.00	72.00
75	是	否	是	女	140.00	70.00	150.00	70.00
67	否	否	是	女	100.00	70.00	102.00	68.00
67	是	否	是	女	140.00	73.00	139.00	79.00
69	否	否	是	女	130.00	80.00	139.00	78.00
67	否	否	是	女	139.00	84.00	139.00	89.00
72	是	否	是	女	138.00	80.00	140.00	74.00
75	是	否	是	女	138.00	80.00	136.00	78.00
67	是	否	是	女	138.00	78.00	137.00	72.00
70	否	否	是	女	108.00	67.00	106.00	69.00

70	否	是	是	女	130.00	80.00	133.00	80.00
77	是	否	是	女	150.00	72.00	155.00	78.00
82	是	否	是	女	135.00	80.00	138.00	82.00
85	否	否	是	女	130.00	80.00	135.00	80.00
68	是	否	是	女	127.00	76.00	126.00	84.00
74	是	否	是	女	110.00	70.00	108.00	68.00
71	否	否	是	女	110.00	72.00	108.00	70.00
77	是	否	是	女	141.00	81.00	142.00	80.00
67	否	否	是	女	157.00	94.00	145.00	90.00
80	是	否	是	女	141.00	90.00	140.00	90.00
68	是	是	是	女	130.00	76.00	139.00	74.00
67	是	否	是	女	120.00	78.00	125.00	82.00
67	否	否	是	女	106.00	66.00	110.00	70.00
68	否	否	是	女	138.00	89.00	139.00	85.00
66	否	是	是	女	118.00	70.00	120.00	74.00
69	否	是	是	女	130.00	89.00	128.00	78.00
76	是	否	是	女	130.00	80.00	142.00	82.00
67	否	否	是	女	110.00	70.00	105.00	65.00
75	是	否	是	女	145.00	80.00	140.00	70.00
70	否	否	是	女	130.00	76.00	128.00	74.00
66	否	否	是	女	139.00	84.00	137.00	82.00
74	是	否	是	女	120.00	80.00	115.00	75.00
66	否	否	是	女	130.00	80.00	135.00	82.00
69	是	否	是	女	148.00	84.00	148.00	86.00
71	是	否	是	女	146.00	70.00	148.00	75.00
76	否	否	是	女	132.00	92.00	133.00	97.00
75	是	否	是	女	139.00	89.00	132.00	80.00
87	是	否	是	女	148.00	62.00	150.00	70.00
73	否	否	是	女	164.00	85.00	166.00	84.00
89	否	否	是	女	120.00	66.00	122.00	70.00
76	是	是	是	女	152.00	76.00	156.00	80.00
70	是	否	是	女	146.00	88.00	142.00	81.00
88	是	否	是	女	172.00	84.00	174.00	89.00
74	否	否	是	女	110.00	75.00	120.00	80.00
70	否	否	是	女	131.00	71.00	121.00	72.00
70	否	否	是	女	110.00	60.00	110.00	62.00
75	否	否	是	女	126.00	76.00	128.00	78.00
75	否	否	是	女	114.00	66.00	118.00	70.00
78	否	否	是	女	132.00	84.00	136.00	88.00
96	否	否	是	女	122.00	75.00	125.00	78.00
73	否	否	是	女	130.00	80.00	138.00	88.00
73	否	否	是	女	102.00	66.00	104.00	64.00
67	是	否	是	女	128.00	78.00	130.00	81.00
71	是	是	是	女	146.00	73.00	135.00	71.00
71	否	否	是	女	155.00	80.00	152.00	78.00
73	是	否	是	女	150.00	80.00	148.00	80.00
78	是	否	是	女	118.00	60.00	120.00	65.00
79	否	是	是	女	127.00	65.00	120.00	63.00
67	是	是	是	女	100.00	70.00	106.00	78.00
79	否	否	是	女	119.00	68.00	120.00	68.00
74	是	是	是	女	164.00	80.00	166.00	80.00

87	是	否	是	女	130.00	70.00	138.00	80.00
70	是	否	是	女	145.00	93.00	148.00	98.00
85	是	否	是	女	156.00	88.00	154.00	80.00
74	是	否	是	女	132.00	60.00	135.00	70.00
79	否	否	是	女	108.00	60.00	110.00	60.00
72	是	否	是	女	130.00	66.00	132.00	68.00
84	是	否	是	女	128.00	72.00	130.00	74.00
70	是	是	是	女	130.00	70.00	139.00	75.00
70	是	否	是	女	125.00	70.00	120.00	65.00
77	是	否	是	女	138.00	76.00	142.00	78.00
71	是	否	是	女	130.00	60.00	135.00	60.00
76	是	是	是	女	128.00	72.00	130.00	70.00
66	是	是	是	女	128.00	80.00	130.00	83.00
78	是	否	是	女	138.00	83.00	140.00	85.00
79	是	否	是	女	139.00	74.00	151.00	71.00
70	是	否	是	女	120.00	80.00	130.00	74.00
68	否	否	是	女	140.00	80.00	140.00	80.00
81	是	是	是	女	132.00	70.00	136.00	76.00
74	否	否	是	女	127.00	84.00	126.00	90.00
67	是	否	是	女	126.00	72.00	139.00	80.00
68	否	否	是	女	115.00	72.00	120.00	75.00
88	否	是	是	女	130.00	80.00	128.00	81.00
73	是	否	是	女	120.00	80.00	124.00	88.00
87	是	否	是	女	130.00	72.00	132.00	76.00
74	否	否	是	女	116.00	70.00	128.00	73.00
66	否	否	是	女	125.00	66.00	130.00	68.00
71	是	是	是	女	122.00	70.00	118.00	66.00
68	是	否	是	女	160.00	100.00	164.00	104.00
78	否	是	是	女	145.00	82.00	150.00	80.00
72	否	否	是	女	130.00	80.00	136.00	82.00
67	是	是	是	女	120.00	65.00	120.00	70.00
80	是	否	是	女	150.00	70.00	152.00	69.00
75	否	否	是	女	138.00	72.00	139.00	82.00
70	是	否	是	女	160.00	90.00	170.00	94.00
72	否	否	是	女	132.00	82.00	135.00	82.00
78	是	是	是	女	158.00	87.00	156.00	83.00
75	是	否	是	女	136.00	70.00	139.00	71.00
89	是	否	是	女	129.00	88.00	130.00	80.00
68	否	否	是	女	130.00	74.00	128.00	76.00
66	否	否	是	女	132.00	80.00	136.00	84.00
66	是	是	是	女	152.00	100.00	155.00	102.00
73	否	否	是	女	132.00	80.00	142.00	82.00
76	是	否	是	女	96.00	60.00	98.00	62.00
70	是	是	是	女	135.00	82.00	138.00	88.00
75	否	否	是	女	125.00	78.00	130.00	82.00
71	是	否	是	女	145.00	95.00	148.00	90.00
66	是	否	否	女	134.00	76.00	138.00	80.00
72	是	是	是	女	118.00	72.00	128.00	84.00
72	否	否	是	女	130.00	80.00	138.00	87.00
69	否	否	是	女	136.00	90.00	140.00	90.00
70	是	否	是	女	157.00	83.00	160.00	86.00

80	否	否	是	女	144.00	90.00	146.00	84.00
71	是	是	是	女	139.00	68.00	140.00	70.00
78	是	是	是	女	110.00	60.00	112.00	62.00
75	是	是	是	女	115.00	60.00	120.00	63.00
78	是	否	是	女	140.00	80.00	152.00	90.00
81	否	否	是	女	146.00	83.00	136.00	70.00

身高（cm	体重（kg	腰围（cm	体质指数	锻炼频率	每次锻炼	坚持锻炼	锻炼方式	吸烟状况
149.00	58.80	92.00	26.49	每天	60.00	1.00	散步	从不吸烟
149.00	54.80	90.00	24.68	不锻炼				从不吸烟
155.00	56.00	85.00	23.31	每天	20.00	1.00	散步	从不吸烟
144.00	51.00	75.00	24.59	不锻炼				从不吸烟
161.00	62.00	84.00	23.92	每周一次	30.00	4.00	散步	从不吸烟
150.00	55.50	76.00	24.67	每天	60.00	2.00	散步	从不吸烟
156.00	68.00	105.00	27.94	每天	30.00	2.00	散步	从不吸烟
146.00	54.00	96.80	25.33	不锻炼				从不吸烟
149.00	58.00	86.00	26.12	不锻炼				从不吸烟
149.00	60.00	91.00	27.03	每天	30.00	5.00	散步	从不吸烟
153.00	55.00	86.00	23.50	不锻炼				从不吸烟
151.00	57.00	93.00	25.00	每天	30.00	5.00	散步	从不吸烟
152.00	53.00	86.00	22.94	每天	30.00	7.00	散步	从不吸烟
141.00	56.00	87.00	28.17	每天	60.00	10.00	体操，太	从不吸烟
155.00	43.00	76.00	17.90	不锻炼				从不吸烟
148.00	56.00	90.00	25.57	不锻炼				从不吸烟
160.00	67.40	99.00	26.33	每周一次	30.00	10.00	散步	从不吸烟
146.00	55.00	85.00	25.80	不锻炼				从不吸烟
154.00	68.00	93.00	28.67	每天	60.00	12.00	散步	从不吸烟
154.00	47.00	72.00	19.82	不锻炼				从不吸烟
143.00	38.00	73.00	18.58	每天	30.00	4.00	散步	从不吸烟
155.50	70.30	98.00	29.07	每天	60.00	2.00	散步	从不吸烟
158.00	70.50	93.00	28.24	每天	30.00	3.00	散步	从不吸烟
158.00	61.50	86.00	24.64	每天	50.00	2.00	散步	从不吸烟
143.00	42.80	74.00	20.93	不锻炼				从不吸烟
166.00	68.00	100.00	24.68	不锻炼				从不吸烟
156.00	53.20	83.00	21.86	每天	60.00	8.00	散步	从不吸烟
145.50	72.00	103.00	34.01	每天	30.00	5.00	散步	从不吸烟
152.00	58.00	82.00	25.10	每天	60.00	3.00	散步	从不吸烟
156.00	62.20	91.00	25.56	每天	30.00	3.00	散步	从不吸烟
148.00	62.00	101.00	28.31	每天	20.00	2.00	散步	从不吸烟
144.00	51.10	90.00	24.64	每天	30.00	10.00	散步	从不吸烟
147.00	62.50	90.00	28.92	每天	30.00	3.00	散步	从不吸烟
150.00	75.00	98.00	33.33	每天	60.00	2.00	散步	从不吸烟
158.00	68.00	97.00	27.24	每天	60.00	8.00	跑步	从不吸烟
155.00	40.00	63.00	16.65	每周一次	30.00	6.00	散步	从不吸烟
152.00	73.00	104.00	31.60	不锻炼				从不吸烟
152.00	47.20	80.00	20.43	不锻炼				从不吸烟
145.00	49.50	83.00	23.54	每天	30.00	3.00	散步	从不吸烟
158.00	52.00	82.00	20.83	每天	60.00	5.00	散步	从不吸烟
140.00	45.00	82.00	22.96	每天	60.00	17.00	散步、太	从不吸烟
155.00	59.00	90.00	24.56	每天	30.00	5.00	散步	从不吸烟
154.00	54.00	86.00	22.77	每周一次	30.00	2.00	散步	从不吸烟
153.00	55.00	78.00	23.50	每周一次	45.00	4.00	散步	从不吸烟
150.00	52.00	88.00	23.11	不锻炼				从不吸烟
155.00	49.00	72.00	20.40	每天	30.00	7.00	散步	从不吸烟
150.00	51.00	80.00	22.67	每天	60.00	10.00	跳舞	从不吸烟
147.00	59.00	87.00	27.30	每天	60.00	20.00	散步	从不吸烟
150.00	55.00	85.00	24.44	每天	30.00	4.00	散步	从不吸烟
150.00	51.00	72.00	22.67	每天	40.00	10.00	慢跑	从不吸烟

140.00	55.00	97.00	28.06	不锻炼				从不吸烟
147.50	53.80	87.00	24.73	不锻炼				从不吸烟
150.50	60.80	90.50	26.84	每天	60.00	6.00	散步	从不吸烟
156.00	56.50	81.00	23.22	不锻炼				从不吸烟
148.00	48.00	81.00	21.91	每天	30.00	6.00	散步	从不吸烟
153.00	63.50	94.00	27.13	每天	30.00	9.00	散步	从不吸烟
145.00	51.00	84.00	24.26	每天	30.00		散步	从不吸烟
150.00	65.70	92.00	29.20	每天	90.00	7.00	散步	从不吸烟
146.00	66.00	94.00	30.96	每天	30.00	7.00	散步	从不吸烟
142.00	46.00	75.00	22.81	每天	30.00	4.00	散步	从不吸烟
148.00	47.00	76.00	21.46	每天	60.00	4.00	散步	从不吸烟
151.00	61.50	84.00	26.97	每天	60.00	6.00	散步	从不吸烟
155.00	69.50	95.00	28.93	每天	30.00	1.00	散步	从不吸烟
156.00	62.50	84.00	25.68	不锻炼				从不吸烟
148.00	66.00	92.00	30.13	每天	30.00	1.00	散步	从不吸烟
162.00	54.50	81.00	20.77	每天	30.00	5.00	散步	从不吸烟
150.00	45.00	74.00	20.00	每天	60.00	2.00	散步	从不吸烟
153.00	62.00	94.00	26.49	每天	30.00	8.00	散步	从不吸烟
154.00	53.10	81.00	22.39	每天	30.00	6.00	散步	从不吸烟
153.00	62.00	94.00	26.49	不锻炼				从不吸烟
156.00	60.00	86.00	24.65	每天	60.00	4.00	散步	从不吸烟
150.00	69.80	96.00	31.02	不锻炼				从不吸烟
160.00	62.00	90.00	24.22	每天	30.00	2.00	散步	从不吸烟
151.00	54.00	91.00	23.68	每周一次!	60.00	5.00	散步	从不吸烟
156.00	62.90	94.00	25.85	每天	30.00	10.00	散步	从不吸烟
155.00	63.00	93.00	26.22	每天	30.00	1.00	散步	从不吸烟
150.00	49.00	89.00	21.78	每天	20.00	5.00	散步,做操	从不吸烟
147.00	50.00	79.00	23.14	不锻炼				从不吸烟
153.00	57.00	85.00	24.35	每天	30.00	1.00	散步	从不吸烟
152.00	67.00	100.00	29.00	每天	30.00	4.00	做操	从不吸烟
141.00	46.20	79.00	23.24	每天	60.00	2.00	散步	从不吸烟
151.50	64.40	94.00	28.06	每天	60.00	11.00	散步	从不吸烟
156.00	70.00	97.00	28.76	每天	30.00	3.00	散步	从不吸烟
142.00	45.00	88.00	22.32	不锻炼				从不吸烟
150.00	56.20	83.00	24.98	每天	30.00	6.00	散步	从不吸烟
149.00	42.00	70.00	18.92	不锻炼				从不吸烟
153.00	74.00	101.00	31.61	每天	60.00	8.00	散步	从不吸烟
150.00	45.00	69.00	20.00	每天	60.00	7.00	跳舞	从不吸烟
151.00	58.50	93.00	25.66	不锻炼				从不吸烟
158.00	60.50	83.00	24.23	每天	30.00	2.00	散步	从不吸烟
151.00	77.00	108.00	33.77	每周一次!	30.00	2.00	散步	从不吸烟
148.00	65.00	105.00	29.67	每天	30.00	10.00	散步	从不吸烟
154.50	52.20	72.00	21.87	每天	30.00	4.00	散步	从不吸烟
156.50	54.60	88.00	22.29	每天	30.00	7.00	散步	从不吸烟
158.00	68.00	95.00	27.24	不锻炼				从不吸烟
149.00	50.00	78.00	22.52	每天	60.00	7.00	散步	从不吸烟
155.50	53.40	88.00	22.08	每周一次!	60.00	3.00	散步	从不吸烟
152.50	47.90	86.00	20.60	每周一次!	30.00	1.00	散步	从不吸烟
155.00	62.30	95.00	25.93	每天	35.00	2.00	散步	从不吸烟
150.00	52.30	95.00	23.24	每天	30.00	5.00	散步	从不吸烟
152.00	62.00	90.00	26.84	不锻炼				从不吸烟

152.50	52.75	79.00	22.68	不锻炼				从不吸烟
146.00	41.00	74.00	19.23	每天	30.00	2.00	散步	从不吸烟
163.00	57.00	79.00	21.45	每周一次!	60.00	10.00	散步	从不吸烟
149.00	61.00	88.00	27.48	每天	60.00	9.00	散步	从不吸烟
148.00	52.00	89.00	23.74	每天	30.00	3.00	散步	从不吸烟
147.00	55.00	89.00	25.45	不锻炼				从不吸烟
152.00	62.50	88.00	27.05	每天	60.00	11.00	散步	从不吸烟
154.00	63.50	90.00	26.78	每天	60.00	1.00	散步	从不吸烟
156.00	63.00	85.00	25.89	每天	30.00	5.00	散步	从不吸烟
150.00	42.00	70.00	18.67	每天	60.00	5.00	散步	从不吸烟
152.00	47.70	78.00	20.65	每天	30.00	1.00	散步	从不吸烟
148.00	39.00	66.00	17.80	不锻炼				从不吸烟
149.00	64.00	94.00	28.83	每天	30.00	3.00	散步	从不吸烟
140.00	54.30	93.00	27.70	每天	30.00	4.00	散步	从不吸烟
153.00	60.00	89.00	25.63	每天	30.00	10.00	散步	从不吸烟
157.00	60.20	92.00	24.42	每天	30.00	8.00	散步	从不吸烟
151.00	48.30	86.00	21.18	每天	30.00	1.00	散步	从不吸烟
152.00	52.00	84.00	22.51	每周一次!	30.00	3.00	散步	从不吸烟
156.50	62.60	97.00	25.56	每天	60.00	10.00	散步	从不吸烟
161.00	60.00	80.00	23.15	每天	60.00	15.00	散步	从不吸烟
142.00	44.00	78.00	21.82	每天	30.00	1.00	散步	从不吸烟
146.00	49.00	77.00	22.99	不锻炼				从不吸烟
153.00	60.00	87.00	25.63	不锻炼				从不吸烟
144.00	58.30	91.00	28.12	每天	30.00	4.00	散步	从不吸烟
148.00	58.00	91.00	26.48	不锻炼				从不吸烟
157.00	59.00	83.00	23.94	不锻炼				从不吸烟
148.50	45.70	84.00	20.72	每天	30.00	6.00	散步	从不吸烟
143.50	47.10	82.00	22.87	每天	30.00	2.00	散步	从不吸烟
154.00	60.00	88.00	25.30	每天	30.00	4.00	散步	从不吸烟
150.00	58.00	93.00	25.78	每天	60.00	8.00	散步	从不吸烟
141.50	51.55	85.00	25.75	每天	30.00	11.00	散步	从不吸烟
155.00	60.60	95.00	25.22	不锻炼				从不吸烟
152.00	55.00	86.00	23.81	不锻炼				从不吸烟
148.00	65.00	99.00	29.67	每周一次!	20.00	1.00	散步	从不吸烟
155.00	67.30	90.00	28.01	每天	60.00	8.00	散步	从不吸烟
145.00	55.00	91.00	26.16	每天	45.00	10.00	散步	从不吸烟
154.00	69.00	98.00	29.09	每周一次!	30.00	5.00	散步	从不吸烟
152.00	42.00	60.00	18.18	不锻炼				从不吸烟
158.00	62.00	92.00	24.84	每天	30.00	10.00	散步	从不吸烟
153.00	63.00	91.00	26.91	不锻炼				从不吸烟
152.00	41.00	65.00	17.75	每天	30.00	7.00	散步	从不吸烟
154.00	57.00	87.00	24.03	每天	40.00	2.00	散步	从不吸烟
148.00	56.70	88.00	25.89	每天	30.00	1.00	散步	从不吸烟
157.00	52.00	89.00	21.10	每天	60.00	5.00	散步	从不吸烟
149.00	50.50	87.00	22.75	每天	30.00	7.00	散步、跑	从不吸烟
144.50	57.90	82.00	27.73	每天	40.00	16.00	散步	从不吸烟
148.00	52.50	84.00	23.97	每天	30.00	2.00	散步	从不吸烟
150.50	52.50	92.00	23.18	每天	30.00	1.00	散步	从不吸烟
160.00	53.00	77.00	20.70	每天	120.00	13.00	散步	从不吸烟
153.00	55.00	89.00	23.50	每天	60.00	60.00	跳舞	从不吸烟
154.00	58.00	89.00	24.46	不锻炼				从不吸烟

145.00	43.00	80.00	20.45	每天	60.00	6.00	做操	从不吸烟
149.00	58.80	89.00	26.49	每天	30.00	8.00	散步	从不吸烟
151.00	53.00	82.00	23.24	每天	30.00	6.00	散步	从不吸烟
150.00	47.00	81.00	20.89	每天	60.00	4.00	散步	从不吸烟
151.50	50.00	84.00	21.78	每天	30.00	6.00	散步	从不吸烟
155.00	67.00	91.00	27.89	每天	30.00	4.00	散步	从不吸烟
164.00	73.00	88.00	27.14	不锻炼				从不吸烟
142.00	48.00	86.00	23.80	每天	75.00	1.00	散步	从不吸烟
151.00	58.00	94.00	25.44	不锻炼				从不吸烟
140.00	52.00	88.00	26.53	每天	30.00	6.00	散步	从不吸烟
141.50	47.75	83.00	23.85	每天	60.00	5.00	太极、跳	从不吸烟
166.00	71.00	94.00	25.77	每天	60.00	5.00	散步	从不吸烟
150.00	51.00	78.00	22.67	不锻炼				从不吸烟
157.00	56.00	88.00	22.72	每天	30.00	3.00	散步	从不吸烟
158.00	45.50	66.00	18.23	每天	30.00	1.00	散步	从不吸烟
154.00	53.10	81.00	22.39	每天	60.00	8.00	散步	从不吸烟
144.50	48.90	83.00	23.42	每周一次	30.00	3.00	散步	从不吸烟
147.00	50.25	80.00	23.25	每天	30.00	7.00	散步	从不吸烟
137.00	42.50	81.00	22.64	每天	30.00	4.00	散步	从不吸烟
157.50	61.75	87.00	24.89	不锻炼				从不吸烟
153.50	61.20	96.00	25.97	每天	30.00	1.00	散步	从不吸烟
152.00	63.70	89.00	27.57	每天	60.00	3.00	散步	从不吸烟
154.00	51.20	80.00	21.59	不锻炼				从不吸烟
147.00	68.00	94.00	31.47	每天	60.00	2.00	散步	从不吸烟
146.00	62.00	92.00	29.09	不锻炼				从不吸烟
147.00	53.45	80.00	24.74	不锻炼				从不吸烟
143.00	55.00	92.00	26.90	每天	30.00	1.00	散步	从不吸烟
153.00	58.00	95.00	24.78	不锻炼				从不吸烟
147.50	60.25	94.00	27.69	不锻炼				从不吸烟
154.00	52.40	81.00	22.09	不锻炼				从不吸烟
152.00	54.70	83.00	23.68	每天	60.00	6.00	散步	从不吸烟
150.00	60.00	91.00	26.67	每天	30.00	2.00	散步	从不吸烟
155.00	54.00	83.00	22.48	每周一次	30.00	5.00	散步	从不吸烟
152.00	56.00	84.00	24.24	每天	60.00	5.00	散步	从不吸烟
146.00	55.00	86.00	25.80	每天	30.00	10.00	散步	从不吸烟
148.00	45.50	76.00	20.77	每天	60.00	9.00	散步	从不吸烟
147.50	44.00	75.00	20.22	不锻炼				从不吸烟
147.00	57.00	78.00	26.38	每天	30.00	5.00	散步	从不吸烟
155.00	56.20	91.00	23.39	每天	30.00	1.00	散步	从不吸烟
151.00	58.90	89.00	25.83	每天	60.00	12.00	散步	从不吸烟
149.00	56.00	91.00	25.22	每天	60.00	20.00	散步	从不吸烟
154.00	82.00	114.00	34.58	每天	60.00	11.00	散步	从不吸烟
155.00	54.00	87.00	22.48	每天	30.00	4.00	散步	从不吸烟
148.00	42.00	79.00	19.17	不锻炼				从不吸烟
140.00	63.00	104.00	32.14	每天	30.00	4.00	散步	从不吸烟
148.00	38.00	66.00	17.35	不锻炼				从不吸烟
145.00	44.00	77.00	20.93	不锻炼				吸烟
149.00	55.00	81.00	24.77	每周一次	30.00	5.00	散步	从不吸烟
150.00	55.00	87.00	24.44	每天	30.00	3.00	散步	从不吸烟
141.00	50.00	79.00	25.15	每天	30.00	12.00	散步	从不吸烟
159.00	71.00	90.00	28.08	每天	30.00	7.00	散步	从不吸烟

161.00	65.00	95.00	25.08	每天	30.00	6.00	散步	从不吸烟
156.00	66.00	96.00	27.12	不锻炼				从不吸烟
148.50	65.60	94.00	29.75	每天	30.00	2.00	散步	从不吸烟
143.00	48.00	86.00	23.47	每天	30.00	14.00	散步	从不吸烟
158.00	56.00	86.00	22.43	每周一次l	40.00	5.00	散步	从不吸烟
153.00	61.00	89.00	26.06	不锻炼				从不吸烟
149.00	48.40	80.00	21.80	每天	30.00	11.00	散步	从不吸烟
152.00	63.00	95.00	27.27	不锻炼				从不吸烟
152.00	51.00	78.00	22.07	每天	30.00	4.00	散步	从不吸烟
145.00	50.00	86.00	23.78	每天	60.00	5.00	散步	从不吸烟
141.00	46.00	81.00	23.14	每天	30.00	4.00	散步	从不吸烟
149.00	46.00	77.00	20.72	每天	30.00	4.00	散步	从不吸烟
152.00	58.50	88.00	25.32	每天	20.00	7.00	散步	从不吸烟
154.00	46.00	75.00	19.40	每天	60.00	20.00	散步	从不吸烟
152.00	72.00	104.00	31.16	每天	60.00	1.00	散步	从不吸烟
160.00	48.30	76.00	18.87	每天	30.00	2.00	散步	从不吸烟
148.00	59.00	91.00	26.94	每天	30.00	1.00	散步	从不吸烟
144.00	43.00	78.00	20.74	每天	20.00	3.00	散步	从不吸烟
153.50	69.80	92.00	29.62	每天	30.00	5.00	散步	从不吸烟
150.00	51.00	84.00	22.67	不锻炼				从不吸烟
148.00	64.00	100.00	29.22	每周一次l	60.00	6.00	散步	从不吸烟
145.00	52.00	92.00	24.73	不锻炼				吸烟
151.00	45.00	71.00	19.74	每天	30.00	2.00	散步	从不吸烟
150.00	58.00	93.00	25.78	每天	30.00	2.00	散步	从不吸烟
145.50	47.80	91.00	22.58	每周一次l	30.00	2.00	散步	从不吸烟
156.50	43.50	68.00	17.76	每天	30.00	2.00	散步	从不吸烟
155.00	63.00	90.00	26.22	每天	60.00	6.00	散步	从不吸烟
153.50	49.20	77.00	20.88	不锻炼				从不吸烟
153.00	62.00	90.00	26.49	不锻炼				从不吸烟
142.00	46.00	75.00	22.81	每天	30.00	10.00	散步	从不吸烟
159.50	59.60	88.00	23.43	每天	60.00	10.00	散步	从不吸烟
158.00	69.00	89.00	27.64	不锻炼				从不吸烟
151.00	44.70	62.00	19.60	每天	30.00	3.00	散步	从不吸烟
156.50	53.60	73.00	21.88	每天	30.00	5.00	散步	从不吸烟
156.00	61.90	86.00	25.44	每天	60.00	6.00	散步	从不吸烟
147.00	58.00	94.00	26.84	不锻炼				从不吸烟
155.00	51.00	83.00	21.23	每天	30.00	3.00	散步	从不吸烟
156.00	56.50	93.80	23.22	每天	30.00	2.00	散步	从不吸烟
140.00	51.00	90.00	26.02	不锻炼				从不吸烟
139.00	42.80	93.00	22.15	每天	20.00	1.00	散步	从不吸烟
145.00	38.10	72.00	18.12	每天	120.00	2.00	散步	从不吸烟
150.50	54.80	87.00	24.19	每天	30.00	2.00	散步	从不吸烟
150.00	52.50	84.00	23.33	不锻炼				从不吸烟
154.00	57.00	92.00	24.03	不锻炼				从不吸烟
148.00	45.00	67.00	20.54	每周一次l	30.00	1.00	散步	从不吸烟
143.00	42.00	73.00	20.54	不锻炼				从不吸烟
149.00	56.50	93.00	25.45	每周一次l	30.00	10.00	散步	从不吸烟
152.00	66.00	96.00	28.57	每周一次l	30.00	6.00	散步	从不吸烟
158.00	62.00	82.00	24.84	每天	180.00	15.00	散步	从不吸烟
153.50	67.50	91.00	28.65	每天	60.00	5.00	做操	从不吸烟
158.00	50.00	81.00	20.03	不锻炼				从不吸烟

146.00	48.60	79.00	22.80	每周一次!	15.00	1.00	散步	从不吸烟
150.00	53.15	86.00	23.62	不锻炼				从不吸烟
146.00	57.00	90.00	26.74	每天	30.00	8.00	散步	从不吸烟
156.00	51.00	80.00	20.96	每天	60.00	3.00	散步	从不吸烟
144.00	61.00	97.00	29.42	不锻炼				从不吸烟
151.00	46.20	80.00	20.26	每天	60.00	6.00	散步	从不吸烟
159.00	58.00	85.00	22.94	每天	30.00	10.00	散步	从不吸烟
153.00	57.70	87.00	24.65	每天	60.00	10.00	太极	从不吸烟
167.00	47.00	67.00	16.85	每天	30.00	10.00	散步	从不吸烟
153.00	64.00	93.00	27.34	每天	60.00	8.00	散步、打	从不吸烟
151.00	58.00	90.00	25.44	每天	30.00	2.00	散步	从不吸烟
154.00	50.00	76.00	21.08	每天	30.00	2.00	散步	从不吸烟
152.00	51.00	88.00	22.07	不锻炼				从不吸烟
138.00	53.00	89.00	27.83	不锻炼				从不吸烟
148.00	87.00	117.00	39.72	每天	30.00	2.00	散步（使	从不吸烟
150.00	52.00	80.00	23.11	每天	30.00	11.00	散步	从不吸烟
154.50	57.55	82.00	24.11	每天	30.00	5.00	散步	从不吸烟
143.00	54.65	81.00	26.73	每天	40.00	7.00	散步	从不吸烟
156.00	75.00	108.00	30.82	不锻炼				从不吸烟
141.50	38.70	62.00	19.33	每天	60.00	6.00	散步	从不吸烟
153.00	60.00	81.00	25.63	每天	60.00	3.00	散步	从不吸烟
150.00	54.00	88.00	24.00	每天	60.00	3.00	散步	从不吸烟
158.00	63.00	97.00	25.24	不锻炼				从不吸烟
160.00	63.00	89.00	24.61	每天	60.00	20.00	散步	从不吸烟
146.00	37.00	67.00	17.36	每天	30.00	5.00	散步	从不吸烟
152.00	53.00	82.00	22.94	不锻炼				从不吸烟
150.00	49.00	78.00	21.78	每天	30.00	22.00	散步	从不吸烟
148.00	54.00	87.00	24.65	每天	30.00	2.00	散步	从不吸烟
147.00	73.00	104.00	33.78	不锻炼				从不吸烟
153.00	52.00	74.00	22.21	每天	30.00	3.00	散步	从不吸烟
166.00	78.00	102.00	28.31	不锻炼				从不吸烟
154.00	49.00	82.00	20.66	不锻炼				从不吸烟
151.00	52.00	78.00	22.81	每天	60.00	1.00	跳舞	从不吸烟
146.00	46.00	87.00	21.58	每天	20.00	5.00	散步	吸烟
151.00	47.00	82.00	20.61	每天	60.00	4.00	散步	从不吸烟
156.00	60.00	85.00	24.65	每天	30.00	1.00	散步	从不吸烟
154.00	55.00	79.00	23.19	每天	30.00	7.00	散步	从不吸烟
151.00	56.00	88.00	24.56	每天	60.00	20.00	散步	从不吸烟
148.00	65.80	90.00	30.04	每天	30.00	3.00	散步	从不吸烟
152.00	59.00	84.00	25.54	每天	30.00	21.00	散步	从不吸烟
151.00	71.00	102.00	31.14	每天	60.00	12.00	打太极	从不吸烟
149.00	64.00	92.00	28.83	每周一次!	30.00	12.00	散步	从不吸烟
156.00	47.00	72.00	19.31	每天	60.00	11.00	散步	从不吸烟
148.00	50.00	88.00	22.83	不锻炼				从不吸烟
158.00	60.00	86.00	24.03	每天	30.00	7.00	广场舞	从不吸烟
125.00	39.00	78.50	24.96	每天	30.00	1.00	散步	从不吸烟
150.00	48.00	83.00	21.33	不锻炼				从不吸烟
156.00	47.00	70.00	19.31	每天	30.00	5.00	散步	从不吸烟
145.00	51.00	98.00	24.26	每天	30.00	12.00	散步	从不吸烟
154.00	52.00	74.00	21.93	每天	60.00	17.00	跳舞	从不吸烟
153.00	55.50	83.00	23.71	每天	30.00	1.00	散步	从不吸烟

138.00	50.50	96.00	26.52	每天	30.00	7.00	散步	从不吸烟
148.00	51.00	89.00	23.28	每天	60.00	3.00	散步	从不吸烟
151.00	58.00	90.00	25.44	每天	30.00	6.00	散步	从不吸烟
148.00	54.00	88.00	24.65	每天	30.00	2.00	散步	从不吸烟
145.00	46.50	78.00	22.12	每天	60.00	14.00	散步	从不吸烟
150.00	49.00	87.00	21.78	不锻炼				从不吸烟
153.00	46.00	76.00	19.65	不锻炼				从不吸烟
142.50	54.80	88.00	26.99	不锻炼				从不吸烟
158.00	50.00	73.50	20.03	不锻炼				从不吸烟
159.00	78.00	107.00	30.85	不锻炼				从不吸烟
160.50	46.30	68.00	17.97	不锻炼				从不吸烟
145.00	61.00	101.00	29.01	每天	60.00	1.00	散步	从不吸烟
150.00	46.00	72.00	20.44	每天	60.00	22.00	散步	从不吸烟
151.00	55.00	84.00	24.12	每天	30.00	1.00	散步	从不吸烟
148.00	70.00	100.00	31.96	每天	30.00	23.00	散步	从不吸烟
155.00	54.50	78.00	22.68	每天	60.00	11.00	散步	从不吸烟
147.00	58.00	100.00	26.84	每天	60.00	6.00	散步	从不吸烟
154.00	57.00	87.00	24.03	每周一次[illegible]	20.00	4.00	慢走	从不吸烟
157.00	51.00	90.00	20.69	每天	30.00	20.00	散步	从不吸烟
150.00	41.00	70.00	18.22	不锻炼				从不吸烟
151.00	50.00	78.00	21.93	每天	60.00	6.00	散步	从不吸烟
140.00	50.00	88.00	25.51	每天	30.00	6.00	散步	从不吸烟
138.00	38.00	70.00	19.95	不锻炼				从不吸烟
155.00	65.00	94.00	27.06	每天	60.00	6.00	散步	从不吸烟
145.00	44.00	82.00	20.93	不锻炼				从不吸烟
145.00	53.00	85.00	25.21	每天	30.00	7.00	散步	从不吸烟
159.00	59.00	88.00	23.34	每天	30.00	2.00	散步	从不吸烟
160.00	52.00	80.00	20.31	不锻炼				从不吸烟
155.00	47.50	76.00	19.77	每天	60.00	2.00	散步	从不吸烟
148.00	60.00	92.00	27.39	每天	60.00	2.00	散步	从不吸烟
149.50	54.90	83.00	24.56	每天	30.00	4.00	散步	从不吸烟
150.00	35.00	78.00	15.56	不锻炼				从不吸烟
153.00	49.50	58.00	21.15	每天	40.00	2.00	散步	从不吸烟
149.00	64.00	91.00	28.83	每天	60.00	17.00	散步	从不吸烟
154.00	60.00	80.00	25.30	每天	60.00	22.00	散步	从不吸烟
152.00	57.00	85.00	24.67	每天	60.00	3.00	散步	从不吸烟
155.00	57.00	81.00	23.73	每天	60.00	5.00	散步	从不吸烟
145.00	49.80	84.00	23.69	每天	30.00	4.00	散步	从不吸烟
148.00	60.10	89.00	27.44	每周一次[illegible]	30.00	2.00	散步	从不吸烟
145.00	60.00	103.00	28.54	每天	60.00	2.00	散步	从不吸烟
152.00	44.00	70.00	19.04	每天	60.00	6.00	散步	从不吸烟
144.00	34.00	60.00	16.40	不锻炼				从不吸烟
152.00	76.70	99.00	33.20	每天	30.00	3.00	散步	从不吸烟
138.00	40.00	73.00	21.00	每天	30.00	20.00	散步	吸烟
152.00	64.00	97.00	27.70	不锻炼				从不吸烟
143.00	61.00	93.00	29.83	每天	30.00	2.00	助行器辅[illegible]	从不吸烟
146.00	54.00	84.00	25.33	每天	60.00	10.00	散步	从不吸烟
142.00	52.00	89.00	25.79	每天	60.00	1.00	散步	从不吸烟
148.00	55.00	90.00	25.11	每天	30.00	6.00	散步	从不吸烟
155.00	56.00	85.00	23.31	不锻炼				从不吸烟
156.00	54.00	84.00	22.19	每周一次[illegible]	60.00	16.00	跑步、散[illegible]	从不吸烟

146.00	54.00	86.00	25.33	每天	60.00	11.00	散步	从不吸烟
147.00	63.00	97.00	29.15	每周一次	30.00	5.00	散步	从不吸烟
137.00	60.00	90.00	31.97	每天	30.00	6.00	散步	从不吸烟
152.00	61.20	95.00	26.49	每天	60.00	1.00	散步	从不吸烟
148.00	58.00	91.00	26.48	每天	60.00	2.00	散步	从不吸烟
160.00	83.00	94.00	32.42	每周一次	60.00	1.00	散步	从不吸烟
145.00	55.00	94.00	26.16	不锻炼				从不吸烟
156.00	65.00	86.00	26.71	每天	30.00	12.00	散步	从不吸烟
158.00	65.00	95.00	26.04	不锻炼				从不吸烟
150.50	45.70	89.50	20.18	每天	60.00	10.00	散步	从不吸烟
156.00	56.00	89.00	23.01	每天	30.00	1.00	散步	从不吸烟
154.00	62.00	94.00	26.14	每天	30.00	6.00	散步	从不吸烟
152.00	65.50	112.00	28.35	不锻炼				从不吸烟
157.00	69.00	94.00	27.99	每天	30.00	1.00	散步	从不吸烟
136.50	47.90	89.00	25.71	每天	30.00	7.00	散步	吸烟
154.00	40.00	63.00	16.87	每天	30.00	3.00	散步	从不吸烟
152.00	52.00	85.00	22.51	每天	60.00	10.00	打球	从不吸烟
155.00	54.00	76.00	22.48	每周一次	60.00	12.00	跳舞	从不吸烟
156.00	49.00	75.00	20.13	每天	30.00	1.00	散步	从不吸烟
154.00	64.00	95.00	26.99	每天	60.00	1.00	散步	从不吸烟
160.00	59.00	94.00	23.05	每天	60.00	6.00	散步	从不吸烟
152.00	56.50	88.00	24.45	每天	60.00	10.00	散步	从不吸烟
165.50	71.00	95.00	25.92	每天	30.00	10.00	散步	从不吸烟
153.00	54.00	89.00	23.07	不锻炼				从不吸烟
155.00	55.50	89.00	23.10	每天	30.00	8.00	散步	从不吸烟
154.00	52.00	73.00	21.93	每天	60.00	5.00	散步	从不吸烟
160.00	56.00	84.00	21.87	每天	60.00	9.00	散步	从不吸烟
148.00	54.10	97.00	24.70	每天	30.00	11.00	散步	从不吸烟
156.00	57.00	81.00	23.42	每天	30.00	16.00	打乒乓球	从不吸烟
152.50	48.70	84.00	20.94	每天	60.00	6.00	散步	从不吸烟
155.00	56.50	96.00	23.52	不锻炼				从不吸烟
144.00	52.00	92.00	25.08	每天	30.00	5.00	散步	从不吸烟
151.00	73.00	105.00	32.02	每天	30.00	10.00	散步	从不吸烟
148.00	58.00	100.00	26.48	不锻炼				从不吸烟
150.00	52.30	95.00	23.24	不锻炼				从不吸烟
158.00	59.00	88.00	23.63	不锻炼				从不吸烟
157.00	57.00	84.00	23.12	每天	60.00	7.00	跳舞	从不吸烟
148.00	46.00	84.00	21.00	每天	60.00	4.00	散步	吸烟
144.50	39.70	83.00	19.01	不锻炼				从不吸烟
145.00	48.00	78.00	22.83	每周一次	20.00	6.00	散步	从不吸烟
148.00	56.50	98.00	25.79	不锻炼				从不吸烟
143.00	52.00	85.00	25.43	每天	30.00	5.00	散步	从不吸烟
145.50	44.50	70.00	21.02	不锻炼				从不吸烟
149.00	45.00	74.00	20.27	每天	60.00	10.00	散步	从不吸烟
158.00	48.20	73.00	19.31	每天	60.00	5.00	散步	从不吸烟
157.00	63.00	91.00	25.56	每天	60.00	18.00	散步	从不吸烟
147.50	38.00	81.00	17.47	每天	30.00	3.00	散步	从不吸烟
148.50	37.30	77.00	16.91	不锻炼				从不吸烟
154.00	75.50	103.00	31.84	每天	60.00	5.00	散步	从不吸烟
145.00	48.00	82.00	22.83	不锻炼				从不吸烟
160.00	61.00	88.00	23.83	不锻炼				从不吸烟

156.50	44.70	77.00	18.25	每天	30.00	6.00	散步	从不吸烟
152.00	52.00	86.00	22.51	每周一次!	30.00	2.00	散步	从不吸烟
152.00	53.00	84.00	22.94	每周一次!	20.00	1.00	散步	吸烟
155.00	55.00	80.00	22.89	不锻炼				从不吸烟
146.00	43.00	85.00	20.17	不锻炼				从不吸烟
152.00	58.00	90.00	25.10	每天	30.00	10.00	散步	从不吸烟
150.00	58.00	97.00	25.78	不锻炼				从不吸烟
146.00	43.00	78.00	20.17	每天	30.00	1.00	散步	从不吸烟
151.00	62.00	91.00	27.19	每天	60.00	5.00	散步	从不吸烟
148.00	62.00	96.00	28.31	不锻炼				从不吸烟
144.00	35.00	60.00	16.88	不锻炼				从不吸烟
150.00	51.50	82.00	22.89	每天	30.00	10.00	散步、跳舞	从不吸烟
154.00	65.00	98.00	27.41	每天	60.00	1.00	跳舞	从不吸烟
150.00	46.50	75.00	20.67	每周一次!	30.00	4.00	散步	从不吸烟
151.00	47.00	83.00	20.61	不锻炼				从不吸烟
158.00	43.85	62.00	17.57	每天	30.00	2.00	散步	从不吸烟
153.00	52.50	79.00	22.43	每天	30.00	1.00	散步	从不吸烟
150.00	71.00	104.00	31.56	每周一次!	30.00	5.00	散步	从不吸烟
159.00	78.00	102.00	30.85	每天	60.00	7.00	散步	从不吸烟
136.00	57.40	90.00	31.03	每天	30.00	6.00	散步	从不吸烟
144.00	57.00	96.00	27.49	每天	30.00	10.00	散步	从不吸烟
153.00	49.00	80.00	20.93	每天	30.00	4.00	散步	从不吸烟
140.00	47.50	87.00	24.23	不锻炼				从不吸烟
154.50	49.00	81.00	20.53	每周一次!	30.00	3.00	散步	从不吸烟
160.00	47.00	80.00	18.36	不锻炼				从不吸烟
145.00	57.00	90.00	27.11	每天	60.00	6.00	散步	从不吸烟
142.00	37.20	70.00	18.45	每天	60.00	1.00	骑自行车	从不吸烟
154.00	62.00	85.00	26.14	每天	15.00	5.00	散步	从不吸烟
155.00	61.00	88.00	25.39	每天	60.00	5.00	散步	从不吸烟
158.00	65.50	93.00	26.24	不锻炼				从不吸烟
158.00	63.00	91.00	25.24	每天	60.00	3.00	散步	从不吸烟
154.00	53.00	84.00	22.35	每天	30.00	5.00	散步	从不吸烟
158.00	62.00	90.00	24.84	每天	60.00	4.00	散步	从不吸烟
153.00	40.30	69.00	17.22	每天	90.00	12.00	散步	从不吸烟
148.00	50.00	84.00	22.83	每周一次!	25.00	1.00	散步	从不吸烟
151.00	52.20	73.00	22.89	不锻炼				从不吸烟
151.00	58.00	89.00	25.44	每天	60.00	3.00	散步	从不吸烟
153.00	66.50	86.00	28.41	每天	60.00	5.00	散步	从不吸烟
149.00	55.50	85.00	25.00	每天	30.00	3.00	散步	从不吸烟
143.00	41.00	72.00	20.05	每天	30.00	9.00	散步	从不吸烟
151.00	54.00	83.00	23.68	每天	45.00	4.00	散步	从不吸烟
150.00	60.50	91.00	26.89	每天	30.00	3.00	散步	从不吸烟
151.00	47.00	84.00	20.61	不锻炼				从不吸烟
143.00	58.90	93.00	28.80	每周一次!	30.00	2.00	散步	从不吸烟
152.00	46.30	76.00	20.04	每天	30.00	8.00	散步	从不吸烟
149.00	67.00	94.00	30.18	每天	30.00	4.00	散步	从不吸烟
158.00	55.20	80.00	22.11	每周一次!	60.00	1.00	散步	从不吸烟
138.50	49.30	80.00	25.70	每天	60.00	4.00	散步	从不吸烟
147.00	44.00	70.00	20.36	每天	60.00	2.00	散步	从不吸烟
152.40	56.40	88.00	24.28	不锻炼				从不吸烟
147.00	45.00	83.00	20.82	每天	30.00	2.00	散步	从不吸烟

159.00	54.00	80.00	21.36	不锻炼				从不吸烟
147.50	56.50	88.00	25.97	不锻炼				从不吸烟
160.00	67.00	90.00	26.17	每天	30.00	1.00	散步	从不吸烟
144.00	50.50	82.50	24.35	每天	30.00	3.00	散步	从不吸烟
154.00	47.50	80.00	20.03	每天	30.00	12.00	散步	从不吸烟
156.00	61.00	91.00	25.07	每天	30.00	10.00	散步	从不吸烟
158.00	65.00	96.00	26.04	每天	60.00	2.00	散步	从不吸烟
156.00	64.00	85.00	26.30	每天	30.00	5.00	散步	从不吸烟
147.50	54.00	88.00	24.82	每天	60.00	3.00	散步	从不吸烟
149.00	55.00	80.00	24.77	不锻炼				从不吸烟
155.00	59.00	82.00	24.56	每天	30.00	9.00	散步	从不吸烟
152.00	49.50	76.00	21.42	每天	30.00	1.00	散步	从不吸烟
152.00	51.80	86.00	22.42	不锻炼				从不吸烟
158.50	54.00	78.00	21.49	不锻炼				从不吸烟
156.00	44.00	78.00	18.08	不锻炼				从不吸烟
156.00	63.00	78.00	25.89	每天	60.00	1.00	打太极	从不吸烟
150.00	59.00	90.00	26.22	每周一次!	30.00	1.00	散步	从不吸烟
150.00	58.40	88.00	25.96	每天	30.00	7.00	散步	从不吸烟
153.00	63.00	93.00	26.91	每天	30.00	1.00	散步	从不吸烟
151.00	63.90	95.00	28.03	每天	45.00	3.00	散步	从不吸烟
153.00	47.00	79.00	20.08	不锻炼				从不吸烟
143.00	46.00	71.00	22.49	每天	60.00	4.00	散步	从不吸烟
157.00	49.50	75.00	20.08	每天	30.00	4.00	散步	从不吸烟
154.50	56.00	81.00	23.46	每天	30.00	10.00	散步	从不吸烟
143.00	69.00	96.00	33.74	每天	30.00	1.00	散步	从不吸烟
148.00	54.00	89.00	24.65	每天	60.00	8.00	散步	从不吸烟
146.00	54.70	95.00	25.66	不锻炼				从不吸烟
154.00	59.00	89.00	24.88	每天	60.00	12.00	散步	从不吸烟
142.00	46.00	82.00	22.81	每天	60.00	6.00	散步	从不吸烟
135.00	41.00	86.00	22.50	每天	20.00	11.00	散步	从不吸烟
149.00	49.90	75.50	22.48	每天	30.00	1.00	做体操	吸烟
153.00	49.50	78.00	21.15	不锻炼				从不吸烟
146.00	43.00	77.00	20.17	每天	30.00	12.00	散步	从不吸烟
164.00	65.00	96.00	24.17	不锻炼				从不吸烟
148.00	65.00	100.00	29.67	每天	60.00	5.00	散步	从不吸烟
156.00	56.00	83.00	23.01	每天	30.00	8.00	散步	从不吸烟
153.00	54.00	84.00	23.07	每天	60.00	12.00	散步	从不吸烟
156.00	70.00	97.00	28.76	每天	20.00	12.00	做操	从不吸烟
152.00	73.00	95.00	31.60	每天	30.00	3.00	散步	从不吸烟
146.00	60.00	89.00	28.15	每天	60.00	9.00	散步	从不吸烟
140.00	40.00	74.00	20.41	不锻炼				从不吸烟
141.00	61.50	85.00	30.93	不锻炼				从不吸烟
154.00	70.00	94.00	29.52	每天	60.00	8.00	散步	从不吸烟
147.00	46.00	74.00	21.29	每天	60.00	1.00	散步	从不吸烟
146.00	51.00	82.00	23.93	不锻炼				从不吸烟
151.00	78.90	102.00	34.60	不锻炼				从不吸烟
146.00	53.00	90.00	24.86	不锻炼				从不吸烟
146.00	52.00	86.00	24.39	每天	30.00	2.00	散步	从不吸烟
145.00	51.00	80.00	24.26	每天	30.00	11.00	散步	从不吸烟
148.00	52.00	88.00	23.74	每天	30.00	12.00	散步	从不吸烟
154.00	47.00	77.00	19.82	每天	20.00	12.00	散步	从不吸烟

154.00	59.00	90.00	24.88	每天	60.00	5.00	散步	从不吸烟
154.00	64.50	90.00	27.20	每天	60.00	2.00	散步	从不吸烟
145.00	49.00	90.00	23.31	不锻炼				从不吸烟
157.00	58.00	86.00	23.53	每天	30.00	3.00	散步	从不吸烟
155.00	79.00	107.00	32.88	每天	30.00	2.00	散步	从不吸烟
155.00	63.70	90.00	26.51	不锻炼				从不吸烟
153.00	58.00	87.00	24.78	每天	30.00	1.00	散步	从不吸烟
156.00	60.00	86.00	24.65	不锻炼				从不吸烟
153.00	65.00	88.00	27.77	每天	60.00	3.00	散步	从不吸烟
156.50	55.50	73.00	22.66	每天	30.00	3.00	散步	从不吸烟
154.00	68.00	85.00	28.67	每天	30.00	1.00	散步	从不吸烟
155.00	60.00	90.00	24.97	每天	60.00	12.00	跳舞	从不吸烟
160.00	57.60	90.00	22.50	每天	60.00	2.00	散步	从不吸烟
155.00	60.00	85.00	24.97	不锻炼				从不吸烟
150.00	65.00	94.00	28.89	不锻炼				从不吸烟
152.00	61.00	94.00	26.40	不锻炼				从不吸烟
158.00	63.00	91.00	25.24	每天	30.00	6.00	散步	从不吸烟
159.00	69.00	92.00	27.29	每天	60.00	17.00	做操	从不吸烟
153.00	65.00	100.00	27.77	不锻炼				从不吸烟
151.00	56.00	84.00	24.56	每天	30.00	10.00	散步	从不吸烟
154.00	48.50	77.00	20.45	不锻炼				从不吸烟
155.00	58.00	96.20	24.14	每天	10.00	2.00	散步	从不吸烟
164.00	63.00	90.00	23.42	每天	60.00	1.00	散步	从不吸烟
156.50	38.90	58.00	15.88	每天	30.00	10.00	散步	从不吸烟
153.00	65.00	94.00	27.77	每周一次!	30.00	1.00	散步	从不吸烟
132.00	31.65	70.00	18.16	每天	30.00	6.00	散步	从不吸烟
146.00	47.00	79.00	22.05	每天	60.00	11.00	散步	从不吸烟
146.00	58.20	98.00	27.30	每天	60.00	5.00	散步	从不吸烟
150.00	40.00	76.00	17.78	每天	30.00	1.00	散步	从不吸烟
135.00	43.00	90.00	23.59	每天	30.00	1.00	散步	从不吸烟
149.00	47.00	77.00	21.17	每天	30.00	5.00	散步	从不吸烟
162.00	52.00	80.00	19.81	每天	60.00	3.00	散步	从不吸烟
158.00	49.80	83.00	19.95	每天	30.00	6.00	散步	从不吸烟
154.00	49.40	79.00	20.83	每天	30.00	4.00	散步	从不吸烟
146.00	56.00	92.00	26.27	每天	60.00	2.00	散步	从不吸烟
152.00	64.00	100.00	27.70	每天	30.00	5.00	散步	从不吸烟
156.00	43.00	73.00	17.67	每天	60.00	2.00	散步	从不吸烟
158.00	84.00	117.00	33.65	每天	30.00	2.00	散步	从不吸烟
146.00	42.00	76.00	19.70	不锻炼				从不吸烟
153.50	34.00	60.00	14.43	每天	30.00	1.00	散步	从不吸烟
145.00	56.00	86.00	26.63	不锻炼				从不吸烟
156.00	50.00	76.00	20.55	每天	60.00	6.00	散步	从不吸烟
144.00	49.00	89.00	23.63	每天	30.00	10.00	散步	从不吸烟
146.00	56.70	86.00	26.60	每天	30.00	6.00	散步	从不吸烟
152.00	57.00	89.00	24.67	不锻炼				从不吸烟
152.20	57.50	90.00	24.82	每天	30.00	4.00	散步	从不吸烟
158.00	50.00	73.00	20.03	每天	60.00	2.00	散步	从不吸烟
145.00	48.50	76.00	23.07	每天	30.00	2.00	散步	从不吸烟
143.00	44.50	74.00	21.76	每天	60.00	5.00	散步	从不吸烟
146.50	57.30	90.00	26.70	每天	30.00	5.00	散步	从不吸烟
156.00	86.00	112.00	35.34	每天	30.00	1.00	散步	从不吸烟

150.00	43.10	71.00	19.16	不锻炼				从不吸烟
150.00	57.30	88.00	25.47	每天	30.00	5.00	散步	从不吸烟
156.00	68.15	89.00	28.00	每天	60.00	2.00	散步	从不吸烟
151.00	48.00	82.00	21.05	不锻炼				从不吸烟
148.00	46.00	78.00	21.00	每天	60.00	3.00	散步	从不吸烟
146.00	51.00	85.00	23.93	不锻炼				从不吸烟
150.00	56.00	81.00	24.89	每周一次!	30.00	1.00	散步	从不吸烟
162.00	61.00	90.00	23.24	每天	60.00	5.00	散步	从不吸烟
157.50	56.00	89.00	22.57	每天	60.00	3.00	散步	从不吸烟
157.00	69.00	102.00	27.99	每周一次!	30.00	20.00	散步	从不吸烟
144.00	39.00	73.00	18.81	每天	30.00	16.00	散步	从不吸烟
135.00	43.00	80.00	23.59	每天	60.00	12.00	散步	从不吸烟
148.00	47.00	80.00	21.46	每天	60.00	7.00	散步	从不吸烟
147.00	59.00	95.00	27.30	每天	50.00	1.00	散步	从不吸烟
148.00	67.00	100.00	30.59	每天	30.00	11.00	散步	从不吸烟
156.50	57.70	94.00	23.56	每天	40.00	5.00	散步	从不吸烟
159.00	62.00	94.00	24.52	不锻炼				从不吸烟
146.00	45.30	72.00	21.25	不锻炼				从不吸烟
148.00	62.00	93.00	28.31	每周一次!	60.00	5.00	散步	从不吸烟
156.00	52.50	75.00	21.57	每天	45.00	2.00	散步	从不吸烟
161.00	47.70	77.00	18.40	每天	30.00	1.00	散步	从不吸烟
164.00	50.00	87.00	18.59	每天	60.00	7.00	散步	从不吸烟
143.00	49.00	78.00	23.96	每天	30.00	1.00	散步	从不吸烟
147.00	49.90	78.00	23.09	每天	60.00	5.00	散步	从不吸烟
141.00	42.50	73.00	21.38	每天	30.00	1.00	散步	从不吸烟
148.00	68.00	104.00	31.04	不锻炼				从不吸烟
157.00	52.00	81.00	21.10	不锻炼				从不吸烟
145.00	60.00	92.00	28.54	不锻炼				从不吸烟
149.00	54.50	77.00	24.55	每天	30.00	1.00	散步	从不吸烟
150.00	54.00	90.00	24.00	不锻炼				从不吸烟
158.50	64.80	94.00	25.79	不锻炼				从不吸烟
150.50	40.10	69.00	17.70	每天	30.00	2.00	散步	从不吸烟
147.00	54.00	82.00	24.99	每周一次!	20.00	22.00	散步	从不吸烟
147.00	55.20	88.00	25.54	每天	30.00	1.00	散步	从不吸烟
160.00	54.50	78.00	21.29	不锻炼				从不吸烟
154.50	44.65	63.00	18.71	每天	60.00	7.00	散步	从不吸烟
148.00	52.00	78.00	23.74	每天	60.00	10.00	散步	从不吸烟
157.00	46.00	69.00	18.66	不锻炼				从不吸烟
162.00	50.10	83.00	19.09	每天	60.00	5.00	散步	从不吸烟
157.00	56.00	80.00	22.72	每天	30.00	10.00	散步	从不吸烟
155.00	58.00	86.00	24.14	每天	30.00	5.00	散步	从不吸烟
150.00	60.00	84.00	26.67	不锻炼				从不吸烟
157.00	74.00	105.00	30.02	每天	60.00	12.00	散步	从不吸烟
154.00	54.10	81.00	22.81	每天	60.00	3.00	健身操	从不吸烟
154.00	55.00	92.00	23.19	每天	30.00	5.00	散步	从不吸烟
143.00	52.20	85.00	25.53	每天	30.00	5.00	散步	从不吸烟
160.00	57.00	81.00	22.27	每周一次!	30.00	2.00	散步	从不吸烟
150.00	58.00	89.00	25.78	不锻炼				从不吸烟
153.00	65.00	89.00	27.77	每天	60.00	3.00	散步	从不吸烟
150.00	57.00	89.00	25.33	每天	60.00	11.00	散步	从不吸烟
152.00	54.00	88.00	23.37	每天	60.00	6.00	散步	从不吸烟

153.00	55.00	80.00	23.50	每天	30.00	12.00	散步	从不吸烟
163.00	64.00	95.00	24.09	每天	60.00	4.00	打太极	从不吸烟
140.00	40.00	78.00	20.41	每天	60.00	21.00	散步	从不吸烟
161.00	63.00	89.00	24.30	每天	30.00	1.00	散步	从不吸烟
148.00	41.00	84.00	18.72	不锻炼				从不吸烟
156.00	52.00	72.00	21.37	每天	60.00	11.00	散步	从不吸烟
146.00	42.00	80.00	19.70	每天	60.00	2.00	散步	从不吸烟
152.00	65.00	94.00	28.13	每天	30.00	5.00	散步	从不吸烟
161.00	61.00	84.00	23.53	不锻炼				从不吸烟
142.00	39.50	77.00	19.59	不锻炼				从不吸烟
148.00	51.00	86.00	23.28	每天	60.00	20.00	散步	从不吸烟
154.00	50.00	77.00	21.08	每天	60.00	5.00	散步	从不吸烟
148.00	38.00	65.00	17.35	不锻炼				从不吸烟
153.00	52.00	78.00	22.21	不锻炼				从不吸烟
150.00	57.00	87.00	25.33	每天	60.00	22.00	散步	从不吸烟
153.00	44.00	76.00	18.80	不锻炼				从不吸烟
142.00	46.50	79.00	23.06	每天	60.00	3.00	散步	从不吸烟
157.00	57.00	82.00	23.12	每天	30.00	3.00	散步	从不吸烟
154.50	60.70	95.00	25.43	每天	30.00	1.00	散步	从不吸烟
149.00	49.00	76.00	22.07	每天	60.00	8.00	散步	吸烟
147.00	37.00	65.00	17.12	每天	60.00	1.00	散步	从不吸烟
153.00	38.00	63.00	16.23	每天	30.00	5.00	散步	从不吸烟
151.00	47.00	82.00	20.61	每天	60.00	1.00	散步	从不吸烟
154.00	54.00	88.00	22.77	每天	30.00	5.00	散步	从不吸烟
150.00	43.00	76.00	19.11	不锻炼				从不吸烟
158.00	67.00	97.00	26.84	每周一次!	30.00	1.00	散步	从不吸烟
159.00	60.00	98.00	23.73	每周一次!	30.00	6.00	散步	从不吸烟
146.00	51.00	77.00	23.93	每天	60.00	23.00	散步	从不吸烟
142.00	52.00	84.00	25.79	每天	60.00	10.00	散步	从不吸烟
144.50	38.45	63.00	18.41	不锻炼				从不吸烟
131.00	56.00	92.00	32.63	每天	30.00	7.00	散在	从不吸烟
145.00	56.00	84.00	26.63	不锻炼				从不吸烟
138.00	43.00	81.00	22.58	不锻炼				从不吸烟
151.50	55.50	81.00	24.18	不锻炼				从不吸烟
146.50	42.50	78.00	19.80	每天	30.00	3.00	散步	从不吸烟
158.00	62.00	93.00	24.84	每天	60.00	2.00	散步	从不吸烟
158.00	51.00	83.00	20.43	不锻炼				从不吸烟
154.00	57.00	88.00	24.03	每天	60.00	2.00	散步	从不吸烟
153.00	49.00	77.00	20.93	每天	60.00	1.00	散步	从不吸烟
150.00	45.00	78.00	20.00	不锻炼				从不吸烟
150.00	68.00	107.00	30.22	每天	60.00	1.00	散步	从不吸烟
146.00	57.00	91.00	26.74	不锻炼				从不吸烟
158.00	57.20	91.00	22.91	每天	30.00	3.00	散步	从不吸烟
152.00	55.10	83.00	23.85	每天	60.00	1.00	跳舞	从不吸烟
156.00	58.00	90.00	23.83	每天	60.00	7.00	散步	从不吸烟
155.00	54.00	78.00	22.48	每天	90.00	10.00	散步	从不吸烟
168.00	66.00	93.00	23.38	每天	30.00	4.00	散步	从不吸烟
156.00	69.00	97.00	28.35	每天	30.00	3.00	散步	从不吸烟
161.00	58.00	80.00	22.38	每天	40.00	17.00	散步	从不吸烟
145.00	54.00	83.00	25.68	每天	30.00	5.00	散步	从不吸烟
144.00	60.00	97.00	28.94	每天	60.00	9.00	散步	从不吸烟

152.50	60.00	82.00	25.80	不锻炼				从不吸烟
145.00	53.60	82.00	25.49	每天	30.00	1.00	散步	从不吸烟
147.00	62.50	92.00	28.92	每天	30.00	10.00	散步	从不吸烟
158.00	60.00	92.00	24.03	每天	30.00	4.00	散步	从不吸烟
135.00	56.00	95.00	30.73	不锻炼				从不吸烟
145.00	34.00	70.00	16.17	不锻炼				从不吸烟
144.00	64.00	93.00	30.86	不锻炼				从不吸烟
153.00	63.00	94.00	26.91	不锻炼				从不吸烟
157.00	71.00	97.00	28.80	每天	60.00	5.00	散步	从不吸烟
140.00	52.10	86.00	26.58	不锻炼				从不吸烟
141.00	47.00	86.00	23.64	每天	30.00	4.00	散步	从不吸烟
148.00	60.00	97.00	27.39	不锻炼				吸烟
139.00	46.00	84.00	23.81	每天	60.00	2.00	跳舞	从不吸烟
150.00	55.00	91.00	24.44	每天	30.00	3.00	做操	从不吸烟
143.00	61.00	89.00	29.83	不锻炼				从不吸烟
154.00	63.00	98.00	26.56	不锻炼				从不吸烟
161.00	52.00	76.00	20.06	每周一次!	30.00	10.00	散步	从不吸烟
148.00	48.00	80.00	21.91	每天	30.00	1.00	散步	从不吸烟
146.00	48.50	82.00	22.75	每天	60.00	12.00	跳舞	从不吸烟
142.00	45.00	86.00	22.32	每天	60.00	4.00	散步	从不吸烟
152.00	56.00	91.00	24.24	每天	30.00	3.00	散步	从不吸烟
138.50	56.70	88.00	29.56	不锻炼				从不吸烟
152.00	60.00	87.00	25.97	每天	60.00	1.00	散步	从不吸烟
158.00	63.50	92.00	25.44	每天	30.00	1.00	散步	从不吸烟
155.00	62.00	97.00	25.81	不锻炼				从不吸烟
142.50	37.70	70.00	18.57	每天	30.00	6.00	散步	从不吸烟
162.00	74.00	101.00	28.20	每天	60.00	3.00	散步	从不吸烟
149.00	31.00	58.00	13.96	不锻炼				从不吸烟
155.00	73.50	108.00	30.59	每天	30.00	3.00	散步	从不吸烟
145.00	41.00	80.00	19.50	每天	30.00	2.00	散步	从不吸烟
156.50	68.70	101.00	28.05	每天	30.00	3.00	散步	从不吸烟
144.00	48.00	82.00	23.15	不锻炼				从不吸烟
148.00	48.00	80.00	21.91	每天	60.00	2.00	散步	从不吸烟
159.00	66.00	92.00	26.11	每天	60.00	6.00	散步	从不吸烟
155.00	49.00	76.00	20.40	每天	60.00	12.00	散步	从不吸烟
164.00	75.00	95.00	27.89	每天	60.00	8.00	散步	从不吸烟
155.00	66.00	94.00	27.47	每天	30.00	3.00	散步	从不吸烟
155.00	65.00	92.00	27.06	每天	60.00	1.00	散步	从不吸烟
151.00	50.00	77.00	21.93	不锻炼				从不吸烟
156.00	56.00	91.00	23.01	每天	60.00	10.00	散步	从不吸烟
147.00	57.00	92.00	26.38	每天	30.00	7.00	散步	从不吸烟
148.00	64.00	105.00	29.22	不锻炼				从不吸烟
146.00	48.00	78.00	22.52	不锻炼				从不吸烟
160.00	51.00	74.00	19.92	不锻炼				从不吸烟
160.00	65.00	89.00	25.39	每天	40.00	3.00	散步	从不吸烟
152.00	54.00	90.00	23.37	每天	30.00	3.00	散步	从不吸烟
152.00	50.00	70.00	21.64	不锻炼				从不吸烟
151.50	64.20	87.00	27.97	每天	30.00	1.00	散步	从不吸烟
150.00	69.00	100.00	30.67	每天	60.00	1.00	散步	从不吸烟
153.50	62.55	87.00	26.55	每周一次!	30.00	5.00	散步	从不吸烟
143.00	36.50	70.00	17.85	每天	60.00	10.00	散步	从不吸烟

156.00	58.10	91.00	23.87	不锻炼				从不吸烟
160.00	53.00	84.00	20.70	每天	30.00	1.00	散步	从不吸烟
153.00	88.00	114.00	37.59	每天	60.00	11.00	散步	从不吸烟
151.00	42.00	65.00	18.42	每天	60.00	2.00	散步	从不吸烟
155.00	62.00	90.00	25.81	每周一次！	30.00	2.00	散步	从不吸烟
163.00	65.00	98.00	24.46	每周一次！	30.00	1.00	散步	从不吸烟
153.00	56.50	85.00	24.14	每周一次！	30.00	2.00	散步	从不吸烟
152.00	44.00	73.00	19.04	每天	30.00	10.00	散步	从不吸烟
149.00	60.00	95.00	27.03	每天	60.00	4.00	散步	从不吸烟
152.50	61.40	88.00	26.40	每天	60.00	1.00	散步	从不吸烟
148.00	82.00	113.00	37.44	每周一次！	30.00	3.00	散步	从不吸烟
152.00	50.00	76.00	21.64	不锻炼				从不吸烟
148.00	47.00	72.00	21.46	每天	30.00	3.00	散步	从不吸烟
146.00	55.00	68.00	25.80	每天	30.00	1.00	散步	从不吸烟
149.00	51.80	78.00	23.33	每天	60.00	10.00	散步	从不吸烟
152.00	64.00	89.00	27.70	每天	60.00	2.00	散步	从不吸烟
156.00	40.00	65.00	16.44	每天	60.00	8.00	散步	从不吸烟
147.00	58.00	90.00	26.84	每天	30.00	4.00	散步	从不吸烟
148.00	54.50	95.20	24.88	每天	120.00	2.00	跳舞	从不吸烟
157.50	68.40	97.00	27.57	每天	30.00	4.00	散步	从不吸烟
153.00	47.00	71.00	20.08	每天	30.00	6.00	散步	从不吸烟
147.00	59.00	93.00	27.30	不锻炼				从不吸烟
155.00	50.00	73.00	20.81	不锻炼				从不吸烟
152.00	76.00	110.00	32.89	不锻炼				从不吸烟
143.00	41.00	75.50	20.05	每周一次！	30.00	2.00	散步	从不吸烟
152.00	55.00	84.00	23.81	每周一次！	30.00	4.00	散步	从不吸烟
154.00	60.00	87.00	25.30	不锻炼				从不吸烟
153.00	43.00	70.00	18.37	每天	60.00	1.00	散步	从不吸烟
147.00	36.00	65.00	16.66	不锻炼				从不吸烟
155.00	61.00	83.00	25.39	每天	60.00	6.00	跳舞	从不吸烟
150.00	52.50	91.00	23.33	不锻炼				从不吸烟
145.00	56.65	95.00	26.94	每天	30.00	4.00	散步	从不吸烟
152.00	51.00	76.00	22.07	不锻炼				从不吸烟
150.00	58.00	88.00	25.78	每天	60.00	4.00	散步	从不吸烟
143.00	44.00	82.00	21.52	每周一次！	20.00	2.00	散步	从不吸烟
142.00	33.00	70.00	16.37	不锻炼				从不吸烟
142.00	38.00	74.00	18.85	每周一次！	30.00	1.00	散步	从不吸烟
157.00	62.00	82.00	25.15	每天	60.00	7.00	散步、跳	从不吸烟
145.00	51.50	91.00	24.49	每天	60.00	12.00	健身操	从不吸烟
151.00	54.00	86.00	23.68	每天	60.00	4.00	散步	从不吸烟
148.00	66.80	106.00	30.50	每天	60.00	8.00	散步	从不吸烟
148.00	49.00	82.00	22.37	每天	60.00	3.00	散步	从不吸烟
146.00	52.50	87.00	24.63	每天	30.00	4.00	散步	从不吸烟
150.00	64.00	98.00	28.44	每天	45.00	6.00	散步	从不吸烟
151.00	72.00	104.00	31.58	不锻炼				从不吸烟
157.00	59.40	87.00	24.10	每天	30.00	1.00	散步	从不吸烟
154.00	55.40	87.00	23.36	不锻炼				从不吸烟
141.50	36.50	58.00	18.23	不锻炼				从不吸烟
150.00	57.00	93.00	25.33	每天	60.00	2.00	散步	从不吸烟
149.00	48.50	82.60	21.85	每周一次！	20.00	4.00	散步	从不吸烟
150.00	58.00	90.00	25.78	每天	30.00	1.00	健身操	从不吸烟

151.00	60.00	84.00	26.31	每周一次!	30.00	1.00	散步	从不吸烟
156.00	50.00	81.00	20.55	不锻炼				从不吸烟
156.00	59.00	87.00	24.24	每天	60.00	10.00	散步	从不吸烟
150.00	48.00	82.00	21.33	每天	60.00	10.00	散步	从不吸烟
156.00	52.00	80.00	21.37	每天	60.00	6.00	散步	从不吸烟
161.00	67.45	93.00	26.02	每天	30.00	7.00	散步	从不吸烟
150.00	43.00	70.00	19.11	不锻炼				从不吸烟
142.00	55.20	99.00	27.38	不锻炼				从不吸烟
149.00	57.00	85.00	25.67	每天	60.00	6.00	散步	从不吸烟
157.00	59.00	88.00	23.94	每天	30.00	2.00	散步	从不吸烟
152.00	51.00	80.00	22.07	每天	60.00	3.00	散步	从不吸烟
148.00	65.00	90.00	29.67	每天	30.00	24.00	散步	从不吸烟
136.00	37.00	78.00	20.00	不锻炼				从不吸烟
148.00	44.50	70.00	20.32	每天	60.00	3.00	散步	从不吸烟
134.00	46.00	82.00	25.62	每天	30.00	2.00	散步	从不吸烟
148.00	52.50	89.00	23.97	每天	60.00	2.00	跳舞	从不吸烟
159.00	57.00	83.00	22.55	每周一次!	60.00	10.00	跳舞	从不吸烟
146.00	46.00	75.00	21.58	每天	45.00	12.00	散步	从不吸烟
155.00	38.00	65.00	15.82	每天	60.00	2.00	散步	从不吸烟
154.00	68.00	93.00	28.67	每天	30.00	5.00	散步	从不吸烟
148.00	42.00	77.00	19.17	每天	60.00	2.00	跳舞	从不吸烟
151.00	57.50	88.00	25.22	每天	30.00	2.00	散步	从不吸烟
151.00	53.00	84.00	23.24	每天	60.00	14.00	散步	从不吸烟
136.50	50.00	98.00	26.84	每天	30.00	10.00	散步	从不吸烟
161.00	72.00	101.00	27.78	每天	60.00	3.00	散步	从不吸烟
145.00	48.50	89.00	23.07	每天	30.00	3.00	散步	从不吸烟
155.00	56.50	82.00	23.52	每天	60.00	7.00	散步	从不吸烟
153.00	50.00	76.00	21.36	每天	60.00	1.00	散步	从不吸烟
155.50	59.00	85.00	24.40	每天	60.00	3.00	散步	从不吸烟
153.00	62.30	92.00	26.61	每天	30.00	5.00	散步	从不吸烟
153.00	70.00	102.00	29.90	每天	60.00	20.00	散步	从不吸烟
161.00	56.00	84.00	21.60	每天	60.00	1.00	散步	从不吸烟
146.00	52.00	84.00	24.39	每天	60.00	7.00	散步	从不吸烟
145.00	43.00	78.00	20.45	不锻炼				从不吸烟
144.00	58.00	88.00	27.97	每天	60.00	3.00	散步	从不吸烟
143.00	58.50	87.50	28.61	每天	60.00	6.00	散步	从不吸烟
145.00	53.00	85.00	25.21	每周一次!	20.00	4.00	散步	从不吸烟
146.00	57.00	90.00	26.74	每天	30.00	1.00	散步	从不吸烟
144.00	39.00	76.00	18.81	不锻炼				从不吸烟
146.00	47.00	79.00	22.05	每天	60.00	9.00	散步	从不吸烟
151.00	45.00	76.00	19.74	每天	60.00	4.00	散步	从不吸烟
149.00	52.00	82.00	23.42	不锻炼				从不吸烟
149.00	47.00	74.00	21.17	每天	60.00	4.00	散步	从不吸烟
150.00	70.00	110.00	31.11	不锻炼				从不吸烟
152.00	69.00	110.00	29.86	每天	60.00	20.00	散步	从不吸烟
143.00	55.00	98.00	26.90	每周一次!	30.00	3.00	散步	从不吸烟
151.00	58.00	90.00	25.44	每天	60.00	2.00	散步	从不吸烟
150.00	62.00	90.00	27.56	每天	30.00	11.00	散步	从不吸烟
151.00	74.00	101.00	32.45	不锻炼				从不吸烟
152.00	65.00	95.00	28.13	不锻炼				从不吸烟
149.00	69.00	94.00	31.08	每天	30.00	3.00	散步	从不吸烟

148.00	65.00	92.00	29.67	每天	40.00	1.00	散步	从不吸烟
158.00	64.00	94.00	25.64	每天	60.00	5.00	打太极	从不吸烟
154.00	60.50	90.00	25.51	每天	60.00	3.00	散步	从不吸烟
158.00	56.00	86.00	22.43	每天	60.00	7.00	散步	从不吸烟
153.00	54.00	70.00	23.07	每天	30.00	5.00	散步	从不吸烟
140.00	43.00	72.00	21.94	不锻炼				从不吸烟
158.00	55.00	80.00	22.03	不锻炼				从不吸烟
158.50	65.00	92.00	25.87	不锻炼				从不吸烟
163.00	61.00	84.00	22.96	每天	30.00	6.00	散步	从不吸烟
149.00	60.00	89.00	27.03	每天	60.00	8.00	散步	从不吸烟
148.00	54.00	84.00	24.65	每周一次	40.00	5.00	散步	从不吸烟
155.00	60.00	95.00	24.97	每天	30.00	3.00	打太极、	从不吸烟
147.00	59.00	91.00	27.30	每天	60.00	13.00	跳舞	从不吸烟
152.00	58.00	90.00	25.10	不锻炼				从不吸烟
152.00	62.00	98.00	26.84	每天	30.00	1.00	做操	从不吸烟
146.00	62.00	89.00	29.09	不锻炼				从不吸烟
148.00	52.00	84.00	23.74	不锻炼				从不吸烟
148.00	42.80	71.00	19.54	每天	60.00	7.00	散步	从不吸烟
153.00	76.00	100.00	32.47	每天	60.00	1.00	散步	从不吸烟
147.00	50.50	87.00	23.37	每天	30.00	11.00	散步	从不吸烟
153.00	64.50	92.00	27.55	不锻炼				从不吸烟
154.00	68.00	94.00	28.67	每天	30.00	3.00	散步	从不吸烟
155.00	60.00	95.00	24.97	每天	30.00	1.00	散步	从不吸烟
151.50	64.00	94.00	27.88	不锻炼				从不吸烟
149.00	56.00	84.00	25.22	每天	30.00	3.00	散步	吸烟
150.00	53.00	83.00	23.56	不锻炼				从不吸烟
151.00	40.00	69.00	17.54	不锻炼				从不吸烟
146.00	71.00	106.00	33.31	不锻炼				从不吸烟
146.00	44.00	78.00	20.64	每天	30.00	1.00	散步	从不吸烟
150.00	54.00	89.00	24.00	每天	60.00	4.00	散步	从不吸烟
150.00	59.00	91.00	26.22	每天	30.00	1.00	做操	从不吸烟
160.00	55.00	80.00	21.48	不锻炼				从不吸烟
148.00	52.00	84.00	23.74	每天	30.00	4.00	散步	从不吸烟
142.00	40.00	69.00	19.84	不锻炼				从不吸烟
146.00	60.00	85.00	28.15	每天	30.00	1.00	跑步	从不吸烟
150.00	57.00	84.00	25.33	不锻炼				从不吸烟
148.00	54.50	81.00	24.88	每天	30.00	3.00	散步	从不吸烟
157.00	61.00	90.00	24.75	每天	60.00	3.00	散步	从不吸烟
139.00	41.00	77.00	21.22	每天	30.00	4.00	散步	从不吸烟
147.00	60.00	98.00	27.77	不锻炼				从不吸烟
147.00	49.00	74.00	22.68	每天	60.00	3.00	散步	从不吸烟
158.00	64.00	95.00	25.64	每周一次	10.00	4.00	散步	从不吸烟
152.00	44.00	90.00	19.04	不锻炼				从不吸烟
150.00	53.00	87.00	23.56	不锻炼				从不吸烟
143.00	37.00	65.00	18.09	不锻炼				从不吸烟
140.00	40.00	78.00	20.41	每周一次	30.00	3.00	散步	从不吸烟
155.00	74.00	107.00	30.80	不锻炼				从不吸烟
147.00	75.00	107.00	34.71	每周一次	30.00	3.00	散步	从不吸烟
143.00	60.00	94.00	29.34	不锻炼				从不吸烟
146.00	53.00	91.00	24.86	每天	30.00	4.00	散步	从不吸烟
154.00	64.00	102.00	26.99	每天	90.00	14.00	散步	从不吸烟

160.00	53.00	74.00	20.70	不锻炼				从不吸烟
142.00	61.50	99.00	30.50	不锻炼				从不吸烟
151.50	49.00	70.00	21.35	不锻炼				从不吸烟
152.00	68.00	94.00	29.43	每天	30.00	10.00	散步	从不吸烟
144.00	70.00	92.00	33.76	不锻炼				从不吸烟
145.50	58.15	88.00	27.47	不锻炼				从不吸烟
141.00	54.00	87.00	27.16	每天	60.00	6.00	散步	从不吸烟
147.00	60.00	90.00	27.77	每天	30.00	10.00	散步	从不吸烟
148.00	37.20	67.50	16.98	不锻炼				从不吸烟
146.00	50.00	87.00	23.46	不锻炼				从不吸烟
152.00	56.00	84.00	24.24	每天	60.00	11.00	散步	从不吸烟
158.00	61.00	97.00	24.44	不锻炼				从不吸烟
154.00	54.00	82.00	22.77	每天	60.00	7.00	散步	从不吸烟
146.00	56.00	93.00	26.27	不锻炼				从不吸烟
146.00	48.00	78.00	22.52	每天	30.00	2.00	体操	从不吸烟
158.50	57.60	81.00	22.93	每天	30.00	5.00	散步	从不吸烟
143.00	53.90	98.00	26.36	每天	30.00	1.00	散步	从不吸烟
153.00	59.00	92.00	25.20	不锻炼				从不吸烟
149.00	71.00	102.00	31.98	每天	30.00	2.00	散步	从不吸烟
145.00	55.00	84.00	26.16	每天	30.00	5.00	散步	从不吸烟
147.00	52.50	87.00	24.30	每天	60.00	3.00	跳舞	从不吸烟
150.00	58.00	85.00	25.78	每天	60.00	9.00	散步	从不吸烟
148.00	62.00	89.00	28.31	每天	30.00	3.00	散步	从不吸烟
148.00	45.00	76.00	20.54	每天	30.00	2.00	散步	从不吸烟
146.00	50.00	87.00	23.46	每天	60.00	2.00	散步	从不吸烟
136.00	51.00	88.00	27.57	不锻炼				从不吸烟
152.00	50.00	80.00	21.64	不锻炼				从不吸烟
145.00	55.00	93.00	26.16	每天	30.00	1.00	散步	从不吸烟
141.00	63.00	101.00	31.69	不锻炼				从不吸烟
144.00	37.00	81.00	17.84	每周一次!	20.00	2.00	散步	从不吸烟
148.00	59.50	89.00	27.16	不锻炼				从不吸烟
148.00	40.00	67.00	18.26	每天	30.00	3.00	散步	从不吸烟
156.00	75.00	104.00	30.82	不锻炼				从不吸烟
149.00	49.00	82.00	22.07	不锻炼				从不吸烟
155.00	57.00	82.00	23.73	每周一次!	15.00	6.00	散步	从不吸烟
150.00	58.00	101.00	25.78	每天	30.00	3.00	散步	从不吸烟
158.00	55.50	85.00	22.23	每天	60.00	3.00	散步	从不吸烟
145.00	67.00	95.00	31.87	每天	30.00	3.00	散步	从不吸烟
148.00	61.00	94.00	27.85	每天	60.00	1.00	散步	从不吸烟
144.00	68.00	101.00	32.79	不锻炼				从不吸烟
158.00	69.00	94.00	27.64	不锻炼				从不吸烟
143.00	49.00	88.00	23.96	每天	30.00	7.00	散步	从不吸烟
151.00	52.00	88.00	22.81	每天	60.00	17.00	散步	从不吸烟
146.50	39.50	72.00	18.40	不锻炼				从不吸烟
154.00	65.50	86.00	27.62	每周一次!	30.00	3.00	散步	从不吸烟
157.00	80.00	105.00	32.46	每天	30.00	2.00	散步	从不吸烟
150.00	36.00	63.00	16.00	不锻炼				从不吸烟
146.00	60.00	89.00	28.15	每天	60.00	10.00	散步	从不吸烟
153.00	58.00	85.00	24.78	每天	30.00	1.00	散步	从不吸烟
146.00	50.00	77.00	23.46	不锻炼				从不吸烟
154.00	65.00	90.00	27.41	每天	40.00	2.00	散步	从不吸烟

150.00	54.00	84.00	24.00	每天	60.00	1.00	散步	从不吸烟
154.00	45.00	78.00	18.97	每周一次!	30.00	3.00	散步	从不吸烟
139.00	49.00	86.00	25.36	每天	60.00	5.00	散步	从不吸烟
143.00	55.00	83.00	26.90	每天	60.00	15.00	散步	从不吸烟
148.00	61.00	96.00	27.85	不锻炼				从不吸烟
164.00	70.00	90.00	26.03	每天	30.00	9.00	散步	从不吸烟
156.00	73.00	93.00	30.00	不锻炼				从不吸烟
146.00	56.00	98.00	26.27	不锻炼				从不吸烟
147.00	59.00	85.00	27.30	每天	30.00	1.00	散步	从不吸烟
146.50	52.90	87.00	24.65	每天	60.00	3.00	散步	从不吸烟
152.00	43.00	63.00	18.61	不锻炼				从不吸烟
162.00	67.00	87.00	25.53	每天	30.00	3.00	散步	从不吸烟
142.00	64.00	91.00	31.74	每天	30.00	7.00	散步	从不吸烟
140.00	41.50	72.00	21.17	不锻炼				从不吸烟
150.00	41.20	75.00	18.31	不锻炼				从不吸烟
142.00	49.00	78.00	24.30	不锻炼				从不吸烟
153.00	60.00	87.00	25.63	每天	60.00	5.00	跳舞	从不吸烟
149.00	51.00	83.00	22.97	不锻炼				从不吸烟
148.00	60.00	82.00	27.39	不锻炼				从不吸烟
154.00	62.00	92.00	26.14	每天	30.00	4.00	散步	从不吸烟
146.00	79.50	105.00	37.30	不锻炼				从不吸烟
157.00	62.00	88.00	25.15	不锻炼				从不吸烟
150.00	69.50	100.00	30.89	每天	30.00	5.00	散步	从不吸烟
158.00	52.00	81.00	20.83	不锻炼				从不吸烟
157.00	60.00	89.00	24.34	每天	30.00	10.00	散步	从不吸烟
158.50	57.70	85.00	22.97	每天	60.00	5.00	散步	从不吸烟
156.00	76.20	96.50	31.31	不锻炼				从不吸烟
156.00	73.00	93.00	30.00	每周一次!	30.00	2.00	散步	从不吸烟
160.00	59.00	87.00	23.05	不锻炼				从不吸烟
162.00	74.00	92.00	28.20	每天	60.00	1.00	散步	从不吸烟
144.00	50.00	90.00	24.11	每天	30.00	3.00	散步	从不吸烟
141.00	51.00	82.00	25.65	每天	40.00	4.00	散步	从不吸烟
157.00	78.00	102.00	31.64	每天	30.00	2.00	散步	从不吸烟
146.00	67.00	92.00	31.43	每天	30.00	3.00	散步	从不吸烟
151.00	52.00	84.00	22.81	每天	60.00	10.00	散步	从不吸烟
150.00	85.00	115.00	37.78	每天	30.00	10.00	散步	从不吸烟
147.00	50.00	91.00	23.14	每周一次!	60.00	3.00	散步	从不吸烟
147.00	61.00	87.00	28.23	每天	30.00	5.00	散步	从不吸烟
154.00	85.00	110.00	35.84	每天	60.00	11.00	散步	从不吸烟
148.00	55.10	96.00	25.16	每天	30.00	3.00	散步	从不吸烟
135.00	38.00	72.00	20.85	不锻炼				从不吸烟
164.00	63.00	90.00	23.42	每天	60.00	10.00	散步	从不吸烟
162.00	63.50	87.00	24.20	每天	30.00	5.00	散步	从不吸烟
149.00	73.00	94.00	32.88	不锻炼				从不吸烟
144.00	53.00	78.00	25.56	每天	30.00	10.00	散步	从不吸烟
148.00	62.40	97.00	28.49	每天	30.00	2.00	散步	从不吸烟
153.00	57.00	80.00	24.35	每天	30.00	10.00	散步	从不吸烟
150.00	58.00	88.00	25.78	每周一次!	30.00	3.00	散步	从不吸烟
153.00	66.00	98.00	28.19	每周一次!	60.00	1.00	散步	从不吸烟
152.00	63.00	92.00	27.27	不锻炼				从不吸烟
152.00	60.00	85.00	25.97	不锻炼				从不吸烟

153.00	82.00	102.00	35.03	每天	30.00	10.00	散步	从不吸烟
148.50	61.50	92.00	27.89	每天	60.00	2.00	散步	从不吸烟
152.00	53.00	90.00	22.94	不锻炼				从不吸烟
149.00	52.80	86.00	23.78	每天	30.00	2.00	散步	从不吸烟
156.50	61.00	88.00	24.91	不锻炼				从不吸烟
149.00	57.00	85.00	25.67	每天	30.00	3.00	散步	从不吸烟
156.00	57.00	89.00	23.42	每天	30.00	3.00	散步	从不吸烟
140.00	50.00	86.00	25.51	不锻炼				从不吸烟
148.00	58.00	91.00	26.48	每天	30.00	3.00	散步	从不吸烟
158.00	63.00	89.00	25.24	每天	30.00	2.00	散步	从不吸烟
145.00	40.00	71.00	19.02	每天	60.00	2.00	散步	从不吸烟
152.00	65.00	85.00	28.13	不锻炼				从不吸烟
158.00	73.00	106.00	29.24	每天	60.00	3.00	跳舞	从不吸烟
150.00	50.50	79.00	22.44	每天	30.00	11.00	散步	从不吸烟
144.00	52.00	90.00	25.08	每周一次!	10.00	2.00	原地活动=	从不吸烟
141.00	47.30	90.00	23.79	不锻炼				从不吸烟
158.00	60.00	96.00	24.03	不锻炼				从不吸烟
145.50	47.80	87.00	22.58	不锻炼				从不吸烟
126.00	42.00	89.00	26.46	不锻炼				从不吸烟
159.00	65.00	96.00	25.71	每周一次!	30.00	4.00	散步	从不吸烟
149.00	68.00	91.00	30.63	每天	30.00	5.00	散步	从不吸烟
150.50	40.35	58.00	17.81	不锻炼				从不吸烟
156.00	64.70	84.00	26.59	每天	30.00	1.00	散步	从不吸烟
157.00	52.00	76.00	21.10	每周一次!	30.00	2.00	散步	从不吸烟
150.00	54.00	85.00	24.00	每天	60.00	2.00	散步	从不吸烟
152.00	50.00	72.00	21.64	每天	60.00	15.00	散步	从不吸烟
152.00	57.00	98.00	24.67	每天	30.00	3.00	散步	从不吸烟
159.00	61.00	79.00	24.13	每天	30.00	10.00	散步	从不吸烟
150.00	48.00	81.00	21.33	每天	30.00	2.00	散步	从不吸烟
146.00	40.00	75.00	18.77	每天	30.00	2.00	散步	从不吸烟
154.00	58.00	77.00	24.46	不锻炼				从不吸烟
150.00	58.70	86.00	26.09	每天	60.00	3.00	散步	从不吸烟
145.00	49.00	88.00	23.31	每天	30.00	10.00	散步	从不吸烟
152.00	50.00	86.00	21.64	每天	30.00	17.00	散步	从不吸烟
134.00	32.90	66.00	18.32	每天	30.00	2.00	散步	从不吸烟
151.00	69.50	99.00	30.48	每天	30.00	3.00	散步	从不吸烟
147.00	42.00	84.00	19.44	每天	60.00	10.00	打太极	从不吸烟
148.00	47.00	85.00	21.46	每天	30.00	2.00	散步	从不吸烟
158.00	66.00	92.00	26.44	不锻炼				从不吸烟
160.00	78.00	98.00	30.47	每天	30.00	3.00	散步	从不吸烟
144.00	53.00	81.00	25.56	每天	30.00	1.00	散步	从不吸烟
165.50	63.70	88.00	23.26	每天	30.00	4.00	散步	从不吸烟
154.00	59.00	82.00	24.88	不锻炼				从不吸烟
148.00	52.00	88.00	23.74	每天	30.00	8.00	散步	从不吸烟
143.00	62.00	96.00	30.32	不锻炼				从不吸烟
152.00	59.00	90.00	25.54	每周一次!	30.00	1.00	散步	从不吸烟
149.00	51.00	75.00	22.97	不锻炼				从不吸烟
152.00	58.00	82.00	25.10	每天	30.00	2.00	散步	从不吸烟
136.00	45.00	71.00	24.33	不锻炼				从不吸烟
154.00	52.00	84.00	21.93	不锻炼				从不吸烟
145.00	40.00	60.00	19.02	不锻炼				从不吸烟

149.00	62.00	87.00	27.93	每周一次Ⅰ	30.00	1.00	散步	从不吸烟
156.00	54.00	94.00	22.19	每周一次Ⅰ	10.00	5.00	散步	从不吸烟
155.00	51.00	81.00	21.23	不锻炼				从不吸烟
150.00	66.00	88.00	29.33	不锻炼				从不吸烟
145.00	41.30	66.00	19.64	每天	45.00	3.00	散步	从不吸烟
154.50	43.00	68.00	18.01	不锻炼				从不吸烟
155.00	61.00	84.00	25.39	每周一次Ⅰ	30.00	1.00	散步	从不吸烟
152.00	65.20	97.00	28.22	每天	60.00	12.00	散步	从不吸烟
165.00	73.00	90.00	26.81	每天	60.00	10.00	散步	从不吸烟
145.00	59.50	90.00	28.30	每天	40.00	1.00	散步	从不吸烟
158.00	72.00	92.00	28.84	不锻炼				从不吸烟
156.60	43.80	73.00	17.86	每天	30.00	6.00	散步	从不吸烟
158.00	80.00	110.00	32.05	不锻炼				从不吸烟
150.00	52.00	86.00	23.11	不锻炼				从不吸烟
154.00	54.00	90.00	22.77	每天	60.00	2.00	散步	从不吸烟
146.00	58.00	92.00	27.21	每天	30.00	9.00	散步	从不吸烟
148.00	45.60	79.00	20.82	每天	120.00	8.00	散步	从不吸烟
152.00	60.00	83.00	25.97	每天	30.00	6.00	散步	从不吸烟
151.50	58.00	81.00	25.27	每天	30.00	1.00	散步	从不吸烟
163.00	70.70	96.00	26.61	每天	30.00	3.00	散步	从不吸烟
152.50	55.00	86.00	23.65	不锻炼				从不吸烟
156.00	55.00	84.00	22.60	每天	30.00	3.00	散步	从不吸烟
154.00	54.00	87.00	22.77	不锻炼				从不吸烟
147.00	51.00	90.00	23.60	每周一次Ⅰ	30.00	4.00	散步	从不吸烟
159.50	47.20	75.00	18.55	每天	60.00	2.00	散步	从不吸烟
160.50	59.30	81.00	23.02	每天	60.00	5.00	散步	从不吸烟
150.00	65.50	94.00	29.11	每天	60.00	10.00	散步	从不吸烟
156.00	63.00	85.00	25.89	每天	30.00	3.00	散步	从不吸烟
155.00	59.50	85.00	24.77	每天	30.00		散步	从不吸烟
145.00	47.00	77.00	22.35	每天	40.00	2.00	散步	从不吸烟
145.00	40.00	76.00	19.02	不锻炼				从不吸烟
153.00	54.85	80.00	23.43	不锻炼				从不吸烟
149.00	49.00	80.00	22.07	每天	30.00	1.00	散步	从不吸烟
156.00	86.00	106.00	35.34	不锻炼				从不吸烟
152.00	60.00	85.00	25.97	每周一次Ⅰ	30.00	2.00	散步	从不吸烟
151.00	51.00	77.00	22.37	不锻炼				从不吸烟
143.50	58.00	87.00	28.17	每天	60.00	10.00	散步	从不吸烟
151.00	60.00	80.00	26.31	不锻炼				从不吸烟
156.50	53.00	93.00	21.64	不锻炼				从不吸烟
150.00	47.00	87.00	20.89	不锻炼				从不吸烟
155.00	63.00	95.00	26.22	每天	60.00	3.00	散步	从不吸烟
156.00	54.50	80.00	22.39	每周一次Ⅰ	30.00	2.00	散步	从不吸烟
148.00	52.50	91.00	23.97	不锻炼				从不吸烟
147.00	43.00	76.00	19.90	偶尔	30.00	8.00	散步	从不吸烟
159.00	58.70	85.00	23.22	每天	30.00	3.00	散步	从不吸烟
145.00	41.00	78.00	19.50	不锻炼				从不吸烟
142.00	50.00	88.00	24.80	每天	60.00	10.00	散步	从不吸烟
154.00	55.00	84.00	23.19	每天	60.00	2.00	散步	从不吸烟
145.00	50.00	79.00	23.78	不锻炼				从不吸烟
155.00	57.00	88.00	23.73	每天	30.00	3.00	八段锦	从不吸烟
153.00	69.00	98.00	29.48	每天	60.00	20.00	散步	从不吸烟

154.50	80.50	102.00	33.72	每天	30.00	1.00	散步	从不吸烟
145.00	61.10	95.00	29.06	不锻炼				从不吸烟
145.00	59.00	98.00	28.06	每天	30.00	6.00	散步	从不吸烟
145.00	52.00	88.00	24.73	不锻炼				从不吸烟
155.00	60.50	90.00	25.18	每天	30.00	4.00	散步	从不吸烟
143.00	44.00	85.00	21.52	每天	60.00	7.00	散步	从不吸烟
133.00	35.00	73.00	19.79	不锻炼				从不吸烟
155.00	74.00	87.00	30.80	每天	30.00	10.00	散步	从不吸烟
150.00	66.00	95.00	29.33	每天	30.00	2.00	散步	从不吸烟
157.00	46.50	74.00	18.86	每天	30.00	1.00	散步	从不吸烟
145.00	52.50	89.00	24.97	每周一次!	30.00	1.00	散步	从不吸烟
152.00	55.00	85.00	23.81	每天	30.00	9.00	散步	从不吸烟
153.00	40.50	65.00	17.30	每天	30.00	1.00	散步	从不吸烟
157.00	51.00	75.00	20.69	不锻炼				从不吸烟
153.00	57.00	86.00	24.35	每天	30.00	3.00	散步	从不吸烟
152.50	77.00	101.00	33.11	每周一次!	30.00	1.00	散步	从不吸烟
150.00	59.00	91.00	26.22	每天	30.00	2.00	散步	从不吸烟
145.00	59.00	89.00	28.06	每天	30.00	10.00	散步	从不吸烟
150.50	50.00	75.00	22.07	每周一次!	40.00	3.00	散步	从不吸烟
143.00	58.80	90.00	28.75	每天	30.00	10.00	散步	从不吸烟
155.00	46.10	74.00	19.19	不锻炼				从不吸烟
151.00	71.70	94.00	31.45	每天	30.00	1.00	散步	从不吸烟
157.00	80.00	110.00	32.46	不锻炼				从不吸烟
156.00	64.00	88.00	26.30	不锻炼				从不吸烟
155.00	51.10	72.00	21.27	每天	60.00	4.00	散步	从不吸烟
155.00	62.80	92.00	26.14	不锻炼				从不吸烟
140.00	40.00	74.00	20.41	每天	30.00	10.00	散步	从不吸烟
144.00	58.00	97.00	27.97	每天	30.00	10.00	散步	从不吸烟
142.00	44.00	70.00	21.82	每天	30.00	1.00	器械锻炼	从不吸烟
148.00	44.10	78.00	20.13	每天	30.00	2.00	散步	从不吸烟
153.50	62.00	90.00	26.31	每天	60.00	10.00	散步	从不吸烟
153.00	51.50	72.00	22.00	每天	30.00	5.00	散步	从不吸烟
140.00	44.00	84.00	22.45	每天	60.00	7.00	散步	从不吸烟
143.00	51.50	83.00	25.18	每天	30.00	6.00	散步	从不吸烟
156.00	57.50	86.00	23.63	不锻炼				从不吸烟
137.00	44.55	75.00	23.74	每天	60.00	6.00	做操，散	从不吸烟
152.00	49.00	86.00	21.21	不锻炼				从不吸烟
160.00	66.60	94.00	26.02	不锻炼				从不吸烟
151.00	50.50	74.00	22.15	不锻炼				从不吸烟
149.00	70.00	93.00	31.53	每天	60.00	8.00	散步	从不吸烟
156.00	43.50	69.00	17.87	每天	60.00	3.00	散步	从不吸烟
153.50	62.30	92.00	26.44	不锻炼				从不吸烟
155.00	61.00	89.00	25.39	每天	30.00	1.00	散步	从不吸烟
155.00	47.50	76.00	19.77	每天	30.00	2.00	散步	从不吸烟
154.00	61.00	98.00	25.72	每天	60.00	6.00	散步	从不吸烟
152.00	56.00	86.00	24.24	每天	30.00	7.00	散步	从不吸烟
139.50	40.50	78.00	20.81	不锻炼				从不吸烟
151.00	48.00	72.00	21.05	每天	40.00	2.00	散步	从不吸烟
155.00	48.00	75.00	19.98	每天	60.00	3.00	跳舞	从不吸烟
144.00	62.70	103.00	30.24	每天	60.00	7.00	散步	从不吸烟
152.00	52.00	81.00	22.51	不锻炼				从不吸烟

154.00	77.50	95.00	32.68	每天	60.00	7.00	散步	从不吸烟
135.50	48.00	83.00	26.14	每天	30.00	1.00	散步	从不吸烟
146.00	51.50	83.00	24.16	不锻炼				从不吸烟
157.00	60.00	91.00	24.34	每周一次!	30.00	3.00	散步	从不吸烟
150.00	45.00	74.00	20.00	每天	60.00	1.00	散步	从不吸烟
151.00	59.00	89.00	25.88	每天	40.00	1.00	散步	从不吸烟
160.00	59.00	78.00	23.05	每天	15.00	1.00	散步	吸烟
153.00	54.00	70.00	23.07	每天	60.00	5.00	散步	从不吸烟
157.00	64.00	86.00	25.96	不锻炼				从不吸烟
148.00	58.00	87.00	26.48	每天	30.00	2.00	散步	从不吸烟
152.50	55.20	77.00	23.74	每天	60.00	10.00	散步	从不吸烟
149.00	60.00	88.00	27.03	每天	30.00	3.00	散步	从不吸烟
148.00	55.50	87.00	25.34	每天	30.00	3.00	散步	从不吸烟
147.00	49.40	70.00	22.86	每天	30.00	1.00	散步	从不吸烟
163.00	61.40	85.00	23.11	每天	30.00	2.00	散步	从不吸烟
150.00	58.60	88.00	26.04	不锻炼				从不吸烟
152.00	56.00	90.00	24.24	每天	60.00	5.00	散步	从不吸烟
148.00	52.70	86.00	24.06	每周一次!	30.00	2.00	散步	从不吸烟
154.00	54.00	84.00	22.77	每天	30.00	7.00	散步	从不吸烟
140.00	50.00	78.00	25.51	每天	30.00	1.00	散步	从不吸烟
152.00	71.00	105.00	30.73	每天	30.00	3.00	散步	从不吸烟
152.00	40.80	61.00	17.66	每天	30.00	4.00	散步	从不吸烟
165.00	82.00	104.00	30.12	每天	60.00	10.00	散步	从不吸烟
145.00	47.00	78.00	22.35	每天	60.00	4.00	散步	从不吸烟
150.00	45.90	77.00	20.40	每天	30.00	2.00	散步	从不吸烟
152.00	64.00	92.00	27.70	每天	30.00	2.00	散步	从不吸烟
154.00	58.00	90.00	24.46	每周一次!	40.00	1.00	散步	从不吸烟
148.00	68.00	85.00	31.04	每天	120.00	1.00	散步	从不吸烟
149.00	63.00	91.00	28.38	每周一次!	30.00	3.00	散步	从不吸烟
148.00	60.00	86.00	27.39	每周一次!	30.00	1.00	散步	从不吸烟
156.00	56.00	84.00	23.01	每天	30.00	10.00	散步	从不吸烟
146.00	47.00	74.00	22.05	每天	30.00	8.00	散步	从不吸烟
151.00	42.00	67.00	18.42	每天	10.00	10.00	散步	过去吸，[illegible]
148.00	58.00	82.00	26.48	每天	30.00	10.00	散步	从不吸烟
160.00	80.00	100.00	31.25	每天	30.00	3.00	散步	从不吸烟
157.00	53.00	73.00	21.50	每天	30.00	3.00	散步	从不吸烟
148.00	49.50	88.00	22.60	每天	30.00	2.00	散步	从不吸烟
145.00	60.00	94.00	28.54	每周一次!	30.00	1.00	散步	从不吸烟
144.00	40.00	63.00	19.29	每天	30.00	10.00	散步	从不吸烟
150.00	59.00	91.00	26.22	每周一次!	30.00	1.00	散步	从不吸烟
148.00	41.00	76.00	18.72	每天	30.00	1.00	散步	从不吸烟
151.00	50.00	74.00	21.93	每天	30.00	2.00	散步	从不吸烟
133.00	48.00	89.00	27.14	不锻炼				从不吸烟
145.00	41.00	91.00	19.50	每周一次!	30.00	2.00	散步	从不吸烟
148.00	67.00	99.00	30.59	每天	30.00	6.00	散步	从不吸烟
157.00	84.00	106.00	34.08	不锻炼				从不吸烟
145.00	41.00	66.00	19.50	每周一次!	30.00	1.00	散步	从不吸烟
150.00	50.00	76.00	22.22	不锻炼				从不吸烟
142.00	54.00	84.00	26.78	每天	30.00	4.00	散步	从不吸烟
157.00	72.00	103.00	29.21	每天	30.00	12.00	散步	从不吸烟
148.00	51.00	75.00	23.28	每天	30.00	5.00	散步	从不吸烟

141.50	48.50	78.00	24.22	每周一次!	20.00	2.00	散步	从不吸烟
163.00	61.00	86.00	22.96	每天	60.00	21.00	散步	从不吸烟
151.00	64.00	85.00	28.07	每天	60.00	12.00	散步	从不吸烟
148.00	40.70	67.00	18.58	每周一次!	30.00	5.00	散步	从不吸烟
152.00	58.00	90.00	25.10	每天	30.00	5.00	散步	从不吸烟
151.00	59.10	94.00	25.92	每天	30.00	6.00	散步	从不吸烟
154.00	60.50	97.00	25.51	不锻炼				从不吸烟
150.00	60.00	90.00	26.67	每周一次!	30.00	1.00	散步	从不吸烟
151.00	63.00	101.00	27.63	每周一次!	30.00	1.00	散步	从不吸烟
156.00	74.00	96.00	30.41	每天	60.00	1.00	散步	从不吸烟
148.00	56.00	85.00	25.57	每天	30.00	2.00	散步	从不吸烟
144.50	71.50	89.00	34.24	每周一次!	60.00	1.00	散步	从不吸烟
151.00	56.60	87.00	24.82	每天	30.00	2.00	散步	从不吸烟
144.50	47.30	80.00	22.65	不锻炼				从不吸烟
157.00	60.00	88.00	24.34	每天	40.00	3.00	散步	从不吸烟
148.00	70.00	94.00	31.96	每天	30.00	11.00	散步	从不吸烟
157.50	57.30	83.00	23.10	每天	30.00	5.00	散步	从不吸烟
152.00	62.50	94.00	27.05	不锻炼				从不吸烟
152.00	51.00	86.00	22.07	每天	60.00	7.00	散步	从不吸烟
145.00	65.00	100.00	30.92	每天	30.00	10.00	散步	从不吸烟
152.50	47.40	78.00	20.38	每天	60.00	6.00	散步	从不吸烟
148.00	53.00	94.00	24.20	每天	10.00	1.00	散步	从不吸烟
145.00	48.00	80.00	22.83	每天	30.00	4.00	散步	从不吸烟
155.00	51.00	88.00	21.23	每天	20.00	1.00	散步	从不吸烟
149.00	55.00	82.00	24.77	不锻炼				从不吸烟
153.00	56.00	86.00	23.92	每天	40.00	2.00	散步	从不吸烟
149.00	52.00	79.00	23.42	每周一次!	30.00	5.00	散步	从不吸烟
152.00	50.00	84.00	21.64	每天	60.00	2.00	散步	从不吸烟
149.00	59.50	89.00	26.80	不锻炼				吸烟
154.00	67.00	102.00	28.25	不锻炼				从不吸烟
155.00	80.00	99.00	33.30	每天	30.00	3.00	散步	从不吸烟
138.00	59.00	97.00	30.98	每天	30.00	4.00	散步	从不吸烟
157.00	77.00	98.00	31.24	每天	30.00	1.00	散步	从不吸烟
155.00	60.00	90.00	24.97	每天	60.00	3.00	散步	从不吸烟
143.00	54.00	83.00	26.41	不锻炼				从不吸烟
160.00	62.00	86.00	24.22	不锻炼				从不吸烟
155.00	54.50	86.00	22.68	每天	30.00	1.00	散步	从不吸烟
140.00	40.00	75.00	20.41	每天	60.00	2.00	散步	从不吸烟
140.00	40.00	75.00	20.41	每天	60.00	2.00	散步	从不吸烟
142.00	37.80	82.00	18.75	不锻炼				从不吸烟
146.00	57.90	91.00	27.16	不锻炼				从不吸烟
145.00	36.00	69.00	17.12	不锻炼				从不吸烟
150.00	28.00	48.00	12.44	不锻炼				从不吸烟
147.00	56.00	87.00	25.92	每天	60.00	7.00	散步	从不吸烟
136.00	43.00	86.00	23.25	不锻炼				从不吸烟
159.00	66.00	91.00	26.11	每天	40.00	2.00	散步	从不吸烟
141.00	45.00	85.00	22.63	每天	60.00	11.00	散步	从不吸烟
155.00	57.00	83.00	23.73	每天	60.00	10.00	散步	从不吸烟
157.00	51.10	84.00	20.73	每天	30.00	12.00	骑自行车	从不吸烟
150.00	59.50	90.00	26.44	每天	30.00	5.00	散步	从不吸烟
159.00	55.00	84.00	21.76	每天	30.00	1.00	散步	从不吸烟

149.00	59.00	86.00	26.58	不锻炼				从不吸烟
142.50	53.00	87.00	26.10	每天	30.00	6.00	散步	从不吸烟
142.00	70.00	102.00	34.72	每天	30.00	5.00	散步	从不吸烟
151.00	44.00	69.00	19.30	每天	30.00	1.00	散步	从不吸烟
149.50	55.00	80.00	24.61	每天	30.00	1.00	散步	从不吸烟
152.00	49.00	84.00	21.21	每天	30.00	1.00	散步	从不吸烟
151.00	61.00	84.00	26.75	每天	60.00	10.00	散步	从不吸烟
163.00	41.00	67.00	15.43	每天	30.00	10.00	散步	从不吸烟
141.00	47.50	68.00	23.89	每天	60.00	3.00	散步	从不吸烟
143.00	73.10	114.00	35.75	不锻炼				从不吸烟
145.00	56.50	85.00	26.87	每天	60.00	10.00	散步	从不吸烟
143.00	42.00	72.00	20.54	不锻炼				从不吸烟
157.00	63.00	89.00	25.56	每天	90.00	11.00	散步	过去吸，[illegible]
150.00	52.00	76.00	23.11	不锻炼				从不吸烟
144.00	56.00	86.00	27.01	每天	30.00	1.00	散步	从不吸烟
152.00	59.50	84.00	25.75	每天	60.00	5.00	散步	从不吸烟
148.00	49.50	71.00	22.60	每天	30.00	5.00	散步	从不吸烟
150.00	41.00	74.00	18.22	每天	60.00	7.00	散步	从不吸烟
157.00	54.00	89.00	21.91	每天	30.00	2.00	散步	从不吸烟
159.00	58.00	83.00	22.94	每天	60.00	9.00	打球	从不吸烟
166.50	59.90	81.00	21.61	每天	30.00	3.00	散步	从不吸烟
158.00	55.50	76.00	22.23	每天	60.00	6.00	散步	从不吸烟
147.00	55.00	89.00	25.45	不锻炼				从不吸烟
152.00	81.00	109.00	35.06	每天	30.00	2.00	散步	从不吸烟
155.00	38.00	70.00	15.82	不锻炼				从不吸烟
154.00	49.00	79.00	20.66	不锻炼				从不吸烟
158.00	58.00	86.00	23.23	每天	30.00	3.00	散步	从不吸烟
148.00	44.00	72.00	20.09	不锻炼				从不吸烟
154.00	48.50	84.00	20.45	每天	30.00	8.00	散步	从不吸烟
158.00	58.00	80.00	23.23	不锻炼				从不吸烟
144.00	38.00	62.00	18.33	不锻炼				从不吸烟
135.00	56.50	85.00	31.00	每天	60.00	2.00	保健操	从不吸烟
153.00	60.00	95.00	25.63	每天	30.00	7.00	散步	从不吸烟
154.00	59.00	85.00	24.88	每周一次[illegible]	30.00	3.00	散步	从不吸烟
142.00	49.50	83.00	24.55	每天	60.00	7.00	散步	从不吸烟
154.00	46.00	78.00	19.40	不锻炼				从不吸烟
149.00	54.00	87.00	24.32	每天	30.00	5.00	散步	从不吸烟
161.00	62.00	86.00	23.92	每天	60.00	1.00	散步	从不吸烟
154.00	63.00	85.00	26.56	每周一次[illegible]	30.00	1.00	散步	从不吸烟
159.00	51.00	75.00	20.17	每天	30.00	9.00	散步	从不吸烟
145.00	62.30	100.00	29.63	每天	30.00	11.00	散步	从不吸烟
165.00	69.00	93.00	25.34	每天	60.00	5.00	散步	从不吸烟
150.00	58.50	90.00	26.00	不锻炼				从不吸烟
153.00	55.00	86.00	23.50	每天	30.00	10.00	散步	从不吸烟
148.00	57.00	85.00	26.02	每天	30.00	3.00	散步	从不吸烟
141.00	38.00	87.00	19.11	每天	30.00	1.00	散步	从不吸烟
160.00	63.00	90.00	24.61	每周一次[illegible]	30.00	1.00	散步	从不吸烟
150.50	64.60	88.00	28.52	每天	30.00	10.00	散步	从不吸烟
160.00	48.40	73.00	18.91	每天	60.00	5.00	散步	从不吸烟
150.00	47.00	72.00	20.89	每天	30.00	6.00	散步	从不吸烟
154.00	67.00	100.00	28.25	每天	30.00	6.00	散步	从不吸烟

152.00	74.00	99.00	32.03	每天	60.00	2.00	散步	从不吸烟
130.00	50.00	95.00	29.59	不锻炼				从不吸烟
153.00	50.50	74.00	21.57	每天	30.00	2.00	散步	从不吸烟
148.00	64.00	95.00	29.22	每天	30.00	6.00	散步	从不吸烟
151.00	53.00	83.00	23.24	不锻炼				从不吸烟
152.00	58.00	82.00	25.10	每天	30.00	7.00	散步	从不吸烟
153.00	55.00	80.50	23.50	每天	60.00	10.00	散步	从不吸烟
136.00	55.00	103.00	29.74	每天	30.00	2.00	散步	从不吸烟
148.00	62.00	101.00	28.31	每天	120.00	1.00	散步	从不吸烟
146.00	51.00	87.00	23.93	不锻炼				从不吸烟
152.00	59.00	89.00	25.54	每天	30.00	14.00	散步	从不吸烟
153.00	49.00	75.00	20.93	每天	30.00	1.00	散步	从不吸烟
149.00	39.50	65.00	17.79	每天	30.00	2.00	散步	从不吸烟
155.00	58.00	88.00	24.14	每天	60.00	1.00	跳舞	从不吸烟
147.50	38.00	61.00	17.47	每天	30.00	3.00	散步	从不吸烟
145.00	64.00	97.00	30.44	不锻炼				从不吸烟
160.00	71.50	104.00	27.93	每天	60.00	2.00	散步	从不吸烟
154.00	48.00	72.00	20.24	每天	60.00	6.00	散步	从不吸烟
150.00	57.00	90.00	25.33	不锻炼				从不吸烟
155.00	63.00	87.00	26.22	每天	30.00	3.00	散步	从不吸烟
150.00	62.50	98.00	27.78	每天	20.00	1.00	散步	从不吸烟
160.00	76.00	103.00	29.69	每天	30.00	2.00	散步	从不吸烟
148.00	55.00	78.00	25.11	每天	30.00	2.00	散步	从不吸烟
150.00	60.00	99.00	26.67	每天	60.00	1.00	散步	从不吸烟
154.00	62.00	85.00	26.14	每周一次!	30.00	1.00	散步	从不吸烟
157.00	63.50	89.00	25.76	不锻炼				从不吸烟
150.00	54.00	86.00	24.00	每天	45.00	1.00	散步	从不吸烟
158.00	56.50	93.00	22.63	每天	30.00	1.00	散步	从不吸烟
146.00	50.00	77.00	23.46	不锻炼				从不吸烟
156.00	57.00	88.00	23.42	每天	60.00	1.00	散步	从不吸烟
145.50	49.00	80.00	23.15	不锻炼				从不吸烟
147.00	54.00	84.00	24.99	每天	30.00	4.00	散步	从不吸烟
151.00	55.00	88.00	24.12	每天	30.00	5.00	散步	从不吸烟
155.00	60.00	80.00	24.97	不锻炼				从不吸烟
152.00	56.00	84.00	24.24	不锻炼				从不吸烟
143.00	50.00	85.00	24.45	不锻炼				从不吸烟
153.00	38.00	64.00	16.23	不锻炼				从不吸烟
152.00	76.00	112.00	32.89	每天	60.00	7.00	散步	从不吸烟
152.00	55.20	80.00	23.89	每天	30.00	4.00	散步	从不吸烟
148.00	59.00	95.00	26.94	不锻炼				从不吸烟
149.00	52.50	90.00	23.65	不锻炼				从不吸烟
153.00	56.00	84.00	23.92	每周一次!	30.00	2.00	散步	从不吸烟
153.00	55.50	85.00	23.71	每天	60.00	7.00	散步	从不吸烟
156.00	70.00	98.00	28.76	每天	60.00	1.00	散步	从不吸烟
157.00	65.00	92.00	26.37	每周一次!	30.00	1.00	散步	从不吸烟
153.00	64.00	100.00	27.34	不锻炼				从不吸烟
153.50	70.00	104.00	29.71	不锻炼				从不吸烟
147.00	56.50	99.00	26.15	每天	60.00	4.00	散步	从不吸烟
147.00	51.00	86.00	23.60	不锻炼				从不吸烟
145.00	57.00	93.00	27.11	不锻炼				从不吸烟
146.00	51.00	75.00	23.93	每天	30.00	10.00	散步	从不吸烟

157.00	51.00	75.00	20.69	每天	40.00	2.00	散步	从不吸烟
139.00	51.00	87.00	26.40	每天	30.00	4.00	散步	从不吸烟
150.00	42.00	73.00	18.67	不锻炼				从不吸烟
153.00	63.00	97.00	26.91	每天	30.00	3.00	散步	从不吸烟
148.00	56.20	98.00	25.66	每天	30.00	1.00	散步	从不吸烟
153.00	53.00	76.00	22.64	每天	30.00	5.00	散步	从不吸烟
144.50	71.00	102.00	34.00	不锻炼				从不吸烟
141.00	36.40	80.00	18.31	每天	30.00	11.00	散步	从不吸烟
140.50	41.30	73.00	20.92	每天	60.00	3.00	散步	从不吸烟
157.00	56.60	78.00	22.96	每天	60.00	4.00	散步	从不吸烟
141.00	31.50	67.00	15.84	每天	30.00	3.00	散步	从不吸烟
144.00	40.00	76.00	19.29	不锻炼				从不吸烟
155.00	62.00	91.00	25.81	不锻炼				从不吸烟
152.00	59.00	92.00	25.54	不锻炼				从不吸烟
155.00	62.50	93.00	26.01	每天	30.00	2.00	散步	从不吸烟
154.00	70.50	91.00	29.73	每天	60.00	4.00	散步	从不吸烟
153.00	49.00	82.00	20.93	每天	30.00	1.00	散步	从不吸烟
150.00	50.00	82.00	22.22	每天	30.00	1.00	散步	从不吸烟
155.00	55.00	84.00	22.89	每天	60.00	5.00	散步	从不吸烟
155.00	51.90	81.00	21.60	每天	60.00	12.00	打太极	从不吸烟
154.00	61.20	93.00	25.81	每天	45.00	15.00	散步	从不吸烟
149.00	57.50	79.00	25.90	每天	60.00	5.00	散步	从不吸烟
150.00	53.50	80.00	23.78	每天	60.00	1.00	散步	从不吸烟
144.50	43.00	69.00	20.59	不锻炼				从不吸烟
152.00	51.00	75.00	22.07	每周一次!	30.00	1.00	散步	从不吸烟
150.00	50.00	84.00	22.22	不锻炼				从不吸烟
151.00	47.00	84.00	20.61	每天	30.00	3.00	散步	从不吸烟
141.00	41.20	69.00	20.72	不锻炼				从不吸烟
151.50	53.80	85.00	23.44	每天	60.00	20.00	散步	从不吸烟
147.00	45.00	73.00	20.82	每天	30.00	5.00	散步	从不吸烟
156.00	51.50	75.00	21.16	每天	30.00	10.00	散步	从不吸烟
155.00	48.00	81.00	19.98	不锻炼				从不吸烟
135.00	39.00	82.00	21.40	不锻炼				从不吸烟
160.00	64.40	85.00	25.16	每天	90.00	21.00	散步	从不吸烟
157.00	56.00	84.00	22.72	不锻炼				从不吸烟
157.00	61.00	80.00	24.75	每周一次!	30.00	1.00	散步	从不吸烟
150.00	53.80	84.00	23.91	每天	60.00	4.00	散步	从不吸烟
151.00	41.00	71.00	17.98	不锻炼				从不吸烟
152.00	61.00	90.00	26.40	不锻炼				从不吸烟
148.00	42.00	79.00	19.17	不锻炼				从不吸烟
143.50	38.80	67.00	18.84	每天	30.00	4.00	散步	从不吸烟
147.00	60.00	99.00	27.77	不锻炼				从不吸烟
151.00	52.60	81.00	23.07	每天	60.00	4.00	散步	从不吸烟
152.00	52.00	80.00	22.51	每周一次!	30.00	1.00	散步	从不吸烟
147.50	50.50	76.00	23.21	每天	60.00	1.00	散步	从不吸烟
153.50	63.30	85.00	26.87	不锻炼				从不吸烟
137.00	51.00	94.00	27.17	每周一次!	30.00	1.00	散步	从不吸烟
147.00	65.00	102.00	30.08	每天	30.00	1.00	做操	从不吸烟
149.00	54.50	86.00	24.55	每天	120.00	7.00	散步	从不吸烟
146.00	63.00	95.00	29.56	不锻炼				从不吸烟
156.00	67.00	98.00	27.53	每天	60.00	16.00	跳舞	从不吸烟

153.00	59.00	94.00	25.20	每天	40.00	1.00	散步	从不吸烟
150.50	41.80	83.00	18.45	不锻炼				从不吸烟
149.00	36.00	63.00	16.22	不锻炼				从不吸烟
148.00	43.80	70.00	20.00	不锻炼				从不吸烟
148.50	60.50	94.00	27.43	不锻炼				从不吸烟
149.00	58.00	90.00	26.12	不锻炼				从不吸烟
140.00	47.00	75.00	23.98	不锻炼				从不吸烟
140.00	49.00	81.00	25.00	每周一次	30.00	1.00	散步	从不吸烟
147.00	50.00	78.00	23.14	每天	30.00			吸烟
149.50	61.20	104.00	27.38	不锻炼				从不吸烟
146.00	51.00	86.00	23.93	每天	30.00	5.00	散步，拍	从不吸烟
140.00	51.00	96.00	26.02	每周一次	30.00	1.00	散步	从不吸烟
153.00	64.00	86.00	27.34	每周一次	30.00	7.00	散步	从不吸烟
149.00	64.70	97.00	29.14	每天	30.00	1.00	散步	从不吸烟
152.00	62.00	78.00	26.84	不锻炼				从不吸烟
153.00	75.30	98.00	32.17	每天	15.00	2.00	散步	从不吸烟
145.00	59.50	97.00	28.30	不锻炼				从不吸烟
135.00	43.00	80.00	23.59	每天	30.00	6.00	做操	从不吸烟
143.00	52.00	77.00	25.43	每天	30.00	3.00	散步	从不吸烟
155.50	69.05	85.00	28.56	每天	60.00	7.00	散步	从不吸烟
146.00	45.00	73.00	21.11	每天	30.00	2.00	散步	从不吸烟
147.50	57.90	87.00	26.61	不锻炼				从不吸烟
146.50	52.00	87.00	24.23	每天	30.00	4.00	散步	从不吸烟
154.00	72.40	101.00	30.53	每天	60.00	30.00	散步	从不吸烟
157.00	62.50	83.00	25.36	每天	30.00	3.00	散步	从不吸烟
148.00	51.00	79.00	23.28	每天	30.00	12.00	散步	从不吸烟
160.00	61.50	94.00	24.02	每天	30.00	6.00	散步	从不吸烟
149.00	61.00	89.00	27.48	不锻炼				从不吸烟
154.00	55.00	83.00	23.19	每天	60.00	2.00	散步	从不吸烟
162.00	65.00	94.00	24.77	每周一次	30.00	1.00	散步	从不吸烟
145.00	65.00	94.00	30.92	每天	60.00	3.00	散步	从不吸烟
134.00	45.00	100.00	25.06	不锻炼				从不吸烟
148.00	54.80	85.00	25.02	每周一次	10.00	6.00	散步	从不吸烟
155.00	65.50	98.00	27.26	每天	15.00	1.00	散步	从不吸烟
154.00	65.00	88.00	27.41	每天	20.00	10.00	散步	从不吸烟
145.00	54.00	84.00	25.68	不锻炼				从不吸烟
149.00	59.00	94.00	26.58	不锻炼				从不吸烟
157.00	49.50	76.00	20.08	每天	30.00	7.00	散步	从不吸烟
153.00	57.00	94.00	24.35	每天	30.00	1.00	散步	从不吸烟
151.00	45.00	79.00	19.74	每天	60.00	12.00	散步	从不吸烟
157.00	67.00	85.00	27.18	每天	30.00	6.00	散步	从不吸烟
152.00	45.90	78.00	19.87	不锻炼				从不吸烟
150.00	38.00	75.00	16.89	不锻炼				从不吸烟
141.00	59.00	88.00	29.68	不锻炼				从不吸烟
145.00	58.00	88.00	27.59	每天	30.00	2.00	散步	从不吸烟
152.00	60.00	88.00	25.97	不锻炼				从不吸烟
150.00	58.00	88.00	25.78	每天	30.00	3.00	散步	从不吸烟
157.00	68.00	90.00	27.59	每天	30.00	3.00	散步	从不吸烟
147.50	50.70	78.00	23.30	每天	60.00	11.00	散步	从不吸烟
157.00	71.00	91.00	28.80	每天	30.00	3.00	散步	从不吸烟
150.00	59.00	84.00	26.22	每天	60.00	3.00	散步	从不吸烟

156.00	52.00	80.00	21.37	不锻炼				从不吸烟
154.00	63.00	93.00	26.56	每天	60.00	6.00	散步	从不吸烟
157.50	51.00	72.00	20.56	每天	60.00	3.00	散步	从不吸烟
158.00	71.00	92.00	28.44	每天	60.00	6.00	散步	从不吸烟
144.00	63.00	96.00	30.38	每天	30.00	6.00	散步	从不吸烟
159.50	68.90	90.00	27.08	每天	30.00	2.00	散步	从不吸烟
146.00	50.00	81.00	23.46	每天	30.00	1.00	散步	从不吸烟
151.50	63.20	83.00	27.54	每周一次!	30.00	2.00	散步	从不吸烟
148.50	44.20	79.00	20.04	不锻炼				从不吸烟
141.50	59.40	93.00	29.67	每天	30.00	5.00	散步	从不吸烟
153.00	60.00	88.00	25.63	每天	30.00	10.00	散步	从不吸烟
154.00	52.00	78.00	21.93	每周一次!	30.00	1.00	散步	从不吸烟
154.50	69.00	95.00	28.91	每天	30.00	4.00	散步	从不吸烟
147.00	72.00	115.00	33.32	每周一次!	30.00	6.00	散步	从不吸烟
150.00	63.00	99.00	28.00	每天	30.00	3.00	散步	从不吸烟
153.00	56.70	85.00	24.22	每天	60.00	3.00	散步	从不吸烟
139.00	37.00	77.00	19.15	不锻炼				从不吸烟
158.00	62.00	88.00	24.84	每周一次!	30.00	7.00	散步	从不吸烟
153.00	54.00	84.00	23.07	每天	30.00	6.00	散步	从不吸烟
130.00	60.00	94.00	35.50	不锻炼				从不吸烟
157.00	47.00	80.00	19.07	不锻炼				从不吸烟
140.00	55.00	92.00	28.06	每天	30.00	15.00	散步	从不吸烟
150.00	52.10	86.00	23.16	每天	30.00	1.00	散步	从不吸烟
134.00	42.00	82.00	23.39	每天	20.00	6.00	做操	从不吸烟
156.00	54.00	77.00	22.19	不锻炼				从不吸烟
148.00	50.10	85.00	22.87	每天	30.00	1.00	散步	从不吸烟
143.50	42.00	74.00	20.40	不锻炼				从不吸烟
146.00	49.00	77.00	22.99	每天	30.00	20.00	做操	从不吸烟
156.50	82.30	113.00	33.60	不锻炼				从不吸烟
156.50	55.00	84.00	22.46	每天	60.00	1.00	散步	从不吸烟
148.00	55.00	88.00	25.11	不锻炼				从不吸烟
152.50	54.60	89.00	23.48	每天	30.00	2.00	散步	从不吸烟
164.00	55.00	77.00	20.45	不锻炼				从不吸烟
149.00	53.00	81.50	23.87	每天	60.00	2.00	跳舞	从不吸烟
149.00	56.50	87.00	25.45	每天	30.00	5.00	散步	从不吸烟
142.50	41.00	82.00	20.19	每天	60.00	3.00	散步	从不吸烟
158.00	60.00	83.00	24.03	每天	30.00	4.00	散步	从不吸烟
135.00	47.80	90.00	26.23	不锻炼				从不吸烟
155.00	72.00	94.00	29.97	每天	30.00	3.00	散步	从不吸烟
153.00	59.20	89.00	25.29	不锻炼				从不吸烟
153.00	58.00	88.00	24.78	不锻炼				从不吸烟
153.00	63.00	92.00	26.91	每周一次!	60.00	1.00	散步	从不吸烟
153.00	70.00	93.00	29.90	每天	30.00	12.00	散步	从不吸烟
151.00	63.85	95.00	28.00	每天	30.00	4.00	散步	从不吸烟
163.00	54.00	85.00	20.32	每天	60.00	16.00	散步	从不吸烟
157.00	63.00	97.00	25.56	偶尔	30.00	2.00	散步	从不吸烟
150.00	52.80	91.00	23.47	不锻炼				从不吸烟
140.00	46.00	90.00	23.47	不锻炼				从不吸烟
150.00	33.00	70.00	14.67	不锻炼				从不吸烟
152.00	59.00	94.00	25.54	每天	30.00	1.00	散步	从不吸烟
145.00	46.50	72.00	22.12	每天	120.00	5.00	散步	从不吸烟

155.00	52.00	83.00	21.64	每天	30.00	2.00	散步	从不吸烟
155.00	57.10	91.00	23.77	每天	60.00	20.00	散步	从不吸烟
151.00	66.00	95.00	28.95	每天	30.00	5.00	散步	从不吸烟
146.00	40.00	79.00	18.77	不锻炼				从不吸烟
148.00	51.90	87.00	23.69	不锻炼				从不吸烟
134.00	55.00	100.00	30.63	每天	30.00	3.00	散步	从不吸烟
157.00	68.00	93.00	27.59	不锻炼				从不吸烟
144.00	36.00	75.00	17.36	不锻炼				从不吸烟
159.00	75.00	99.00	29.67	每天	60.00	11.00	散步	从不吸烟
151.00	51.00	84.00	22.37	每天	60.00	9.00	散步	从不吸烟
143.00	40.00	69.00	19.56	每天	30.00	8.00	散步	从不吸烟
150.00	48.00	76.00	21.33	每天	30.00	6.00	散步	从不吸烟
152.00	52.00	92.00	22.51	每周一次l	30.00	2.00	做体操	从不吸烟
144.00	52.00	82.00	25.08	不锻炼				从不吸烟
143.00	41.00	84.00	20.05	不锻炼				从不吸烟
161.00	70.50	92.00	27.20	每天	30.00	4.00	散步	从不吸烟
145.00	51.70	85.00	24.59	每天	30.00	5.00	散步	从不吸烟
146.00	43.70	83.00	20.50	每天	30.00	1.00	散步	从不吸烟
148.00	68.00	100.00	31.04	每天	60.00	2.00	跳舞	从不吸烟
152.00	60.00	95.00	25.97	不锻炼				从不吸烟
151.00	62.00	91.00	27.19	每周一次l	30.00	1.00	散步	从不吸烟
158.00	63.00	86.00	25.24	每周一次l	20.00	6.00	散步	从不吸烟
144.50	50.70	86.00	24.28	不锻炼				从不吸烟
153.00	57.00	87.00	24.35	每天	60.00	3.00	散步	从不吸烟
153.00	65.00	88.00	27.77	每天	60.00	4.00	骑自行车	从不吸烟
147.00	38.70	72.00	17.91	不锻炼				从不吸烟
152.00	49.30	77.00	21.34	不锻炼				从不吸烟
146.00	63.00	94.00	29.56	不锻炼				从不吸烟
140.00	58.50	97.00	29.85	每天	30.00	1.00	散步	从不吸烟
138.00	53.00	89.00	27.83	不锻炼				从不吸烟
160.00	79.00	94.00	30.86	不锻炼				从不吸烟
170.50	49.50	71.00	17.03	每天	60.00	1.00	散步	从不吸烟
154.00	57.00	75.00	24.03	每天	60.00	11.00	散步	从不吸烟
155.00	65.00	84.00	27.06	每周一次l	30.00	3.00	散步	从不吸烟
155.00	53.00	70.00	22.06	每天	60.00	2.00	跳舞	从不吸烟
152.00	83.00	107.00	35.92	不锻炼				从不吸烟
148.00	44.00	80.00	20.09	每天	30.00		散步	从不吸烟
150.00	42.00	79.00	18.67	每天	60.00	7.00	散步	从不吸烟
141.00	53.00	75.00	26.66	每天	30.00	10.00	散步	从不吸烟
145.00	57.00	90.00	27.11	每天	30.00	1.00	散步	从不吸烟
138.00	38.00	71.00	19.95	不锻炼				从不吸烟
137.50	58.00	98.00	30.68	不锻炼				从不吸烟
152.50	63.00	91.00	27.09	不锻炼				从不吸烟
150.00	53.20	93.00	23.64	每天	60.00	4.00	散步	从不吸烟
151.00	55.00	93.00	24.12	每天	60.00	2.00	散步	从不吸烟
154.00	62.00	93.00	26.14	不锻炼				从不吸烟
155.00	61.50	80.00	25.60	每天	30.00	6.00	散步	从不吸烟
154.50	55.00	82.00	23.04	每天	30.00	5.00	散步	从不吸烟
156.00	65.00	97.00	26.71	每天	30.00	1.00	散步	从不吸烟
149.00	50.00	79.00	22.52	不锻炼				从不吸烟
148.00	54.00	86.00	24.65	每天	30.00	1.00	骑自行车	从不吸烟

143.00	50.80	85.00	24.84	不锻炼				从不吸烟
153.00	55.00	70.00	23.50	每天	40.00	16.00	散步，骑	从不吸烟
151.00	63.00	83.00	27.63	每天	30.00	3.00	散步	从不吸烟
156.00	47.00	74.00	19.31	每周一次	40.00	1.00	散步	从不吸烟
151.00	52.00	87.00	22.81	每天	30.00	4.00	散步	从不吸烟
154.00	79.00	105.00	33.31	不锻炼				从不吸烟
153.00	65.40	94.00	27.94	每天	30.00	2.00	散步	从不吸烟
152.00	40.00	67.00	17.31	每天	30.00	1.00	散步	从不吸烟
151.00	56.00	84.00	24.56	每天	30.00	10.00	散步	从不吸烟
173.00	84.70	107.00	28.30	不锻炼				从不吸烟
161.50	62.50	85.00	23.96	不锻炼				从不吸烟
148.00	40.00	70.00	18.26	每周一次	30.00	3.00	散步	从不吸烟
158.00	64.50	91.00	25.84	不锻炼				从不吸烟
143.00	53.00	90.00	25.92	每天	30.00	2.00	散步	从不吸烟
148.00	61.00	95.00	27.85	每天	60.00	4.00	散步	从不吸烟
140.00	42.00	72.00	21.43	每天	60.00	3.00	体操	从不吸烟
160.00	50.00	75.00	19.53	每天	60.00	4.00	散步	从不吸烟
155.00	61.00	85.00	25.39	每天	30.00	5.00	散步	从不吸烟
155.50	54.60	80.00	22.58	每天	30.00	2.00	散步	从不吸烟
152.50	66.00	91.00	28.38	不锻炼				从不吸烟
153.00	59.00	86.00	25.20	每天	30.00	6.00	做操	从不吸烟
146.00	43.00	68.00	20.17	每天	60.00	4.00	散步	从不吸烟
157.00	60.00	91.00	24.34	每天	20.00	2.00	散步	从不吸烟
154.00	58.00	79.00	24.46	不锻炼				从不吸烟
149.00	61.00	94.00	27.48	不锻炼				从不吸烟
150.00	52.20	80.00	23.20	每天	20.00	4.00	体操	从不吸烟
148.00	51.00	65.00	23.28	每天	30.00	1.00	散步	从不吸烟
152.00	50.00	72.00	21.64	不锻炼				从不吸烟
152.50	50.00	78.00	21.50	每天	30.00	10.00	散步	从不吸烟
150.50	70.00	94.00	30.90	每天	30.00	4.00	散步	从不吸烟
151.00	45.00	71.00	19.74	每天	60.00	10.00	散步	从不吸烟
157.00	57.00	88.00	23.12	每天	30.00	2.00	散步	从不吸烟
154.00	42.60	74.00	17.96	每天	60.00	30.00	散步	从不吸烟
160.00	67.70	98.00	26.45	不锻炼				从不吸烟
150.00	51.00	76.00	22.67	每天	60.00	21.00	散步	从不吸烟
159.00	69.50	93.00	27.49	不锻炼				从不吸烟
150.00	50.50	86.00	22.44	不锻炼				从不吸烟
150.00	38.00	65.00	16.89	每天	30.00	11.00	散步	从不吸烟
146.00	62.00	96.00	29.09	每周一次	30.00	2.00	散步	从不吸烟
140.00	45.00	84.00	22.96	每天	30.00	4.00	散步	从不吸烟
151.00	66.60	94.00	29.21	每天	30.00	4.00	散步	从不吸烟
163.50	66.00	94.00	24.69	每天	30.00	3.00	散步	从不吸烟
154.50	68.50	91.00	28.70	每天	30.00	4.00	散步	从不吸烟
155.00	62.30	82.00	25.93	不锻炼				从不吸烟
149.50	57.00	84.00	25.50	每天	60.00	11.00	散步	从不吸烟
157.00	63.00	77.00	25.56	每天	30.00	2.00	散步	从不吸烟
147.00	62.00	95.00	28.69	每天	30.00	4.00	散步	从不吸烟
151.00	80.00	108.00	35.09	每周一次	40.00	1.00	散步	从不吸烟
149.00	52.00	85.00	23.42	每天	40.00	11.00	散步	从不吸烟
146.00	55.00	87.00	25.80	每天	30.00	4.00	散步	从不吸烟
151.00	83.50	108.00	36.62	不锻炼				从不吸烟

158.50	60.00	86.00	23.88	每天	60.00	7.00	散步	从不吸烟
150.50	41.50	66.00	18.32	不锻炼				从不吸烟
150.00	44.00	70.00	19.56	不锻炼				从不吸烟
149.00	49.00	82.00	22.07	每周一次!	30.00	1.00	散步	从不吸烟
153.00	52.00	88.00	22.21	每天	60.00	3.00	散步	从不吸烟
148.00	61.00	89.00	27.85	每天	30.00	2.00	散步	从不吸烟
153.00	60.00	86.00	25.63	每天	60.00	7.00	散步	从不吸烟
160.00	71.00	100.00	27.73	不锻炼				从不吸烟
144.00	48.00	90.00	23.15	每天	30.00	1.00	散步	从不吸烟
152.00	61.50	99.00	26.62	每天	45.00	1.00	散步	从不吸烟
152.00	55.00	79.00	23.81	每天	30.00	8.00	散步	从不吸烟
154.00	46.80	74.00	19.73	每天	60.00	4.00	散步	从不吸烟
158.00	43.00	75.00	17.22	每天	30.00	14.00	散步	从不吸烟
149.00	50.00	79.00	22.52	不锻炼				从不吸烟
151.50	49.00	79.00	21.35	每天	30.00	6.00	散步	从不吸烟
148.00	36.00	68.00	16.44	每天	30.00	3.00	散步	从不吸烟
136.00	42.00	84.00	22.71	每天	30.00	3.00	散步	从不吸烟
152.00	57.00	97.00	24.67	每天	30.00	2.00	散步	从不吸烟
154.00	64.00	88.00	26.99	每天	30.00	4.00	散步	从不吸烟
151.00	58.00	85.00	25.44	不锻炼				从不吸烟
149.00	45.00	70.00	20.27	每天	60.00	10.00	散步	从不吸烟
157.00	73.90	91.00	29.98	每天	60.00	12.00	散步	从不吸烟
153.50	49.50	77.00	21.01	不锻炼				从不吸烟
148.00	42.00	77.00	19.17	每周一次!	20.00	1.00	散步	从不吸烟
153.00	60.00	88.00	25.63	每天	60.00	7.00	散步	从不吸烟
140.00	52.00	84.00	26.53	每天	60.00	5.00	散步	从不吸烟
154.00	61.50	86.00	25.93	每天	60.00	2.00	散步	从不吸烟
142.00	41.50	83.00	20.58	不锻炼				从不吸烟
134.50	55.15	87.00	30.49	不锻炼				从不吸烟
158.00	52.50	90.00	21.03	每天	30.00	3.00	散步	从不吸烟
154.00	59.00	88.00	24.88	不锻炼				从不吸烟
151.50	71.00	95.00	30.93	每周一次!	30.00	1.00	散步	从不吸烟
155.00	50.00	84.00	20.81	每天	30.00	4.00	散步	从不吸烟
135.00	44.00	79.00	24.14	每天	30.00	8.00	散步	从不吸烟
156.00	43.00	68.00	17.67	每天	30.00	3.00	散步	从不吸烟
141.00	44.00	88.00	22.13	不锻炼				从不吸烟
148.00	53.00	84.00	24.20	每周一次!	60.00	6.00	散步	从不吸烟
144.00	58.50	98.00	28.21	每周一次!	30.00	2.00	散步	从不吸烟
155.00	52.50	84.00	21.85	每天	60.00	3.00	散步、跳	从不吸烟
152.00	63.00	106.00	27.27	不锻炼				从不吸烟
148.00	57.60	90.00	26.30	不锻炼				从不吸烟
147.00	52.00	89.00	24.06	不锻炼				从不吸烟
149.00	63.00	95.00	28.38	不锻炼				从不吸烟
132.00	39.00	86.00	22.38	不锻炼				从不吸烟
148.00	50.50	91.00	23.06	不锻炼				从不吸烟
151.50	51.00	80.00	22.22	每天	60.00	6.00	散步	从不吸烟
154.00	60.00	90.00	25.30	每天	60.00	9.00	散步	从不吸烟
150.00	61.00	99.00	27.11	每天	60.00	1.00	散步	从不吸烟
145.00	70.00	109.00	33.29	每天	30.00	7.00	散步	从不吸烟
145.00	43.50	74.00	20.69	每天	30.00	10.00	散步	从不吸烟
142.00	61.00	99.00	30.25	每天	30.00	7.00	散步	从不吸烟

147.00	57.00	86.00	26.38	不锻炼				从不吸烟
156.00	58.00	90.00	23.83	不锻炼				从不吸烟
160.00	57.00	80.00	22.27	每天	60.00	7.00	散步	从不吸烟
154.00	57.00	86.00	24.03	每天	60.00	1.00	散步	从不吸烟
153.00	64.50	93.00	27.55	每天	30.00	2.00	散步	从不吸烟
155.00	56.00	90.00	23.31	每天	30.00	1.00	散步	从不吸烟
137.00	41.00	80.00	21.84	不锻炼				从不吸烟
157.00	42.00	69.00	17.04	每天	60.00	4.00	散步	从不吸烟
157.00	63.00	88.00	25.56	每天	30.00	3.00	散步	从不吸烟
145.00	47.70	86.00	22.69	每天	30.00	13.00	散步	从不吸烟
152.00	53.00	84.00	22.94	不锻炼				从不吸烟
151.00	59.00	88.00	25.88	每天	40.00	4.00	散步	从不吸烟
152.00	48.00	75.00	20.78	不锻炼				从不吸烟
153.00	50.20	70.00	21.44	每天	60.00	5.00	散步	从不吸烟
150.00	70.00	97.00	31.11	每天	60.00	6.00	散步	从不吸烟
154.00	62.50	90.00	26.35	不锻炼				从不吸烟
148.00	47.00	77.00	21.46	不锻炼				从不吸烟
158.00	37.90	60.00	15.18	不锻炼				从不吸烟
163.00	82.00	102.00	30.86	每天	60.00	2.00	散步	从不吸烟
150.00	60.00	90.00	26.67	每天	30.00	3.00	散步	从不吸烟
156.50	60.00	92.00	24.50	每天	30.00	1.00	散步	从不吸烟
138.00	50.20	86.00	26.36	每天	30.00	10.00	散步	从不吸烟
140.00	50.00	90.00	25.51	不锻炼				从不吸烟
149.00	65.00	99.00	29.28	每天	30.00	3.00	散步	从不吸烟
149.00	61.90	90.00	27.88	每天	15.00	1.00	散步	从不吸烟
136.00	39.00	80.00	21.09	每天	30.00	6.00	散步	从不吸烟
140.00	51.00	80.00	26.02	每天	60.00	7.00	散步	从不吸烟
147.00	54.00	78.00	24.99	每天	30.00	1.00	散步	从不吸烟
151.00	36.30	67.00	15.92	每天	60.00	6.00	散步	从不吸烟
155.50	74.40	96.00	30.77	每天	60.00	10.00	散步	从不吸烟
154.00	50.00	82.00	21.08	每天	60.00	10.00	散步	从不吸烟
153.00	64.00	87.00	27.34	每天	30.00	11.00	散步	从不吸烟
138.00	47.50	86.00	24.94	每天	20.00	3.00	散步	从不吸烟
156.00	59.50	85.00	24.45	每天	30.00	9.00	散步	从不吸烟
147.00	68.50	91.00	31.70	每天	30.00	1.00	散步	从不吸烟
148.00	59.50	93.00	27.16	不锻炼				从不吸烟
160.00	46.00	78.00	17.97	每天	60.00	10.00	打太极	从不吸烟
161.00	72.00	91.00	27.78	每天	30.00	4.00	散步	从不吸烟
147.00	51.00	80.00	23.60	不锻炼				从不吸烟
160.00	50.00	80.00	19.53	每天	30.00	4.00	散步	从不吸烟
164.00	68.00	86.00	25.28	每天	30.00	3.00	散步	从不吸烟
148.00	55.00	86.00	25.11	每天	30.00	4.00	散步	从不吸烟
162.50	54.50	74.00	20.64	不锻炼				从不吸烟
158.00	67.80	96.00	27.16	每天	60.00	1.00	散步	从不吸烟
148.00	49.60	83.00	22.64	不锻炼				从不吸烟
150.00	53.50	78.00	23.78	每天	15.00	1.00	跳舞	从不吸烟
147.00	50.00	87.00	23.14	每周一次!	30.00	2.00	散步	从不吸烟
150.00	58.00	81.00	25.78	每天	60.00	10.00	跳舞	从不吸烟
144.00	48.00	76.00	23.15	每天	30.00	3.00	散步	从不吸烟
161.00	60.00	85.00	23.15	每天	30.00	3.00	散步	从不吸烟
149.00	42.00	69.00	18.92	每天	30.00	1.00	散步	从不吸烟

138.00	56.00	95.00	29.41	不锻炼				从不吸烟
147.00	54.00	88.00	24.99	不锻炼				从不吸烟
150.00	57.00	90.00	25.33	不锻炼				从不吸烟
157.00	65.00	81.00	26.37	每天	60.00	2.00	散步	从不吸烟
157.00	60.00	88.00	24.34	每周一次!	30.00	1.00	散步	从不吸烟
160.00	63.00	92.00	24.61	每天	30.00	3.00	散步	从不吸烟
145.00	47.00	86.00	22.35	每周一次!	30.00		散步	从不吸烟
146.50	56.00	83.00	26.09	每天	30.00	2.00	散步	从不吸烟
150.00	65.00	103.00	28.89	每天	30.00	10.00	散步	从不吸烟
150.00	58.00	98.00	25.78	每天	60.00	1.00	散步	从不吸烟
157.00	58.00	89.00	23.53	不锻炼				从不吸烟
143.00	54.00	92.00	26.41	不锻炼				从不吸烟
144.00	57.50	102.00	27.73	不锻炼				从不吸烟
155.00	59.00	86.00	24.56	每天	30.00	3.00	散步	从不吸烟
140.00	47.80	77.00	24.39	每天	60.00	6.00	散步	从不吸烟
149.00	60.00	92.00	27.03	不锻炼				从不吸烟
144.00	50.00	82.00	24.11	每天	60.00	8.00	散步	从不吸烟
146.00	52.00	82.00	24.39	不锻炼				从不吸烟
152.00	54.00	84.00	23.37	不锻炼				从不吸烟
139.00	67.00	108.00	34.68	每天	30.00	2.00	散步	从不吸烟
143.00	52.50	90.00	25.67	每天	30.00	2.00	散步	从不吸烟
154.00	71.00	99.00	29.94	每天	60.00	3.00	散步	从不吸烟
148.00	48.00	88.00	21.91	不锻炼				从不吸烟
150.00	38.00	70.00	16.89	不锻炼				从不吸烟
148.50	58.00	90.00	26.30	不锻炼				从不吸烟
154.00	47.00	71.00	19.82	不锻炼				从不吸烟
157.00	59.00	86.00	23.94	不锻炼				从不吸烟
152.00	50.00	80.00	21.64	不锻炼				从不吸烟
155.00	65.00	87.00	27.06	每天	30.00	3.00	散步	从不吸烟
149.00	55.00	89.00	24.77	每天	60.00	41.00	散步	从不吸烟
145.00	52.00	76.00	24.73	每周一次!	20.00	1.00	散步	从不吸烟
163.00	65.00	93.00	24.46	每天	60.00	2.00	散步	从不吸烟
150.00	42.00	72.00	18.67	每周一次!	30.00	1.00	散步	从不吸烟
151.00	40.00	63.00	17.54	不锻炼				从不吸烟
155.00	47.00	80.00	19.56	每天	15.00	10.00	散步	从不吸烟
143.00	49.00	87.00	23.96	每天	30.00	7.00	散步	从不吸烟
147.00	63.60	96.00	29.43	每天	30.00	4.00	散步	从不吸烟
165.00	63.10	87.00	23.18	不锻炼				从不吸烟
150.00	33.50	60.00	14.89	不锻炼				从不吸烟
153.00	71.00	91.00	30.33	不锻炼				从不吸烟
156.00	54.00	76.00	22.19	每天	60.00	3.00	跳舞	从不吸烟
157.00	67.00	93.00	27.18	每天	30.00	3.00	散步	从不吸烟
151.00	50.00	74.00	21.93	不锻炼				从不吸烟
163.00	72.70	102.00	27.36	每天	60.00	8.00	散步	从不吸烟
147.00	59.00	86.00	27.30	每天	30.00	5.00	散步	从不吸烟
138.00	47.00	72.00	24.68	每天	30.00	8.00	散步	从不吸烟
148.00	54.00	86.00	24.65	每周一次!	30.00	1.00	散步	从不吸烟
145.00	52.00	85.00	24.73	每天	30.00	1.00	散步	从不吸烟
155.00	55.00	83.00	22.89	每天	30.00	3.00	散步	从不吸烟
161.00	66.50	94.00	25.65	每天	30.00	10.00	散步	从不吸烟
147.00	48.90	77.00	22.63	每天	30.00	7.00	散步	从不吸烟

146.00	62.00	92.00	29.09	每天	60.00	8.00	散步	从不吸烟
141.50	53.50	87.00	26.72	不锻炼				从不吸烟
150.50	67.20	101.00	29.67	不锻炼				从不吸烟
158.00	60.00	80.00	24.03	每天	60.00	12.00	散步	从不吸烟
154.00	58.50	91.00	24.67	每天	30.00	1.00	散步	从不吸烟
155.50	73.00	105.00	30.19	不锻炼				从不吸烟
145.00	37.00	70.00	17.60	每周一次l	10.00	1.00	散步	从不吸烟
143.50	36.00	73.00	17.48	每天	30.00	3.00	散步	从不吸烟
145.00	68.00	96.00	32.34	不锻炼				从不吸烟
145.50	63.35	98.00	29.92	每天	60.00	5.00	散步	从不吸烟
142.00	53.70	96.00	26.63	每天	30.00	3.00	散步	从不吸烟
151.00	56.00	93.00	24.56	不锻炼				从不吸烟
147.00	58.00	92.00	26.84	每周一次l	30.00	2.00	散步	从不吸烟
152.00	50.00	85.00	21.64	每天	30.00	2.00	散步	从不吸烟
152.00	61.00	86.00	26.40	每天	60.00	4.00	散步	从不吸烟
154.00	67.00	93.00	28.25	每天	60.00	12.00	散步、跳	从不吸烟
155.00	48.30	80.00	20.10	每天	30.00	2.00	散步	从不吸烟
148.00	51.00	83.00	23.28	每天	30.00	3.00	散步	从不吸烟
149.00	57.50	86.00	25.90	每天	30.00	8.00	散步	从不吸烟
149.00	45.00	68.00	20.27	每天	60.00	3.00	打羽毛球	从不吸烟
155.50	59.00	88.00	24.40	每天	30.00	2.00	散步	从不吸烟
143.00	60.00	96.00	29.34	偶尔	30.00	3.00	散步	从不吸烟
155.00	58.00	92.00	24.14	每天	30.00	3.00	散步	从不吸烟
148.00	68.00	102.00	31.04	每天	30.00	5.00	散步	从不吸烟
161.50	62.00	79.00	23.77	每天	30.00	10.00	散步	从不吸烟
152.50	51.40	78.00	22.10	每天	60.00	3.00	散步	从不吸烟
146.00	48.00	84.00	22.52	不锻炼				从不吸烟
159.00	75.00	102.00	29.67	每天	30.00	5.00	散步	从不吸烟
163.00	66.00	86.00	24.84	每天	30.00	3.00	散步	从不吸烟
151.00	56.00	83.00	24.56	每天	30.00	10.00	散步	从不吸烟
159.00	61.20	85.00	24.21	不锻炼				从不吸烟
145.00	50.50	80.00	24.02	每天	30.00	10.00	散步	从不吸烟
173.00	80.00	93.00	26.73	每天	30.00	2.00	散步	从不吸烟
155.00	71.30	96.00	29.68	每天	30.00	2.00	散步	从不吸烟
151.00	54.00	83.00	23.68	每天	60.00	6.00	散步	从不吸烟
152.00	51.00	84.00	22.07	每天	30.00	4.00	散步	从不吸烟
154.00	55.00	80.00	23.19	每天	60.00	7.00	散步	从不吸烟
145.00	60.90	88.00	28.97	每天	30.00	1.00	散步	从不吸烟
152.00	71.40	109.00	30.90	每天	30.00	1.00	散步	从不吸烟
151.00	78.00	104.00	34.21	每天	60.00	7.00	跳舞	从不吸烟
144.00	55.00	88.00	26.52	每天	60.00	2.00	散步	从不吸烟
154.00	46.00	81.00	19.40	每天	60.00	2.00	散步	从不吸烟
149.00	65.00	86.00	29.28	每天	60.00	4.00	散步	从不吸烟
163.00	68.00	91.00	25.59	每天	30.00	1.00	散步	从不吸烟
141.00	38.00	78.00	19.11	每天	30.00	10.00	散步	从不吸烟
152.00	51.00	80.00	22.07	不锻炼				从不吸烟
144.00	72.50	100.00	34.96	每天	60.00	3.00	散步	从不吸烟
150.00	50.00	75.00	22.22	每天	30.00	13.00	散步	从不吸烟
155.00	68.00	86.00	28.30	每天	30.00	2.00	散步	从不吸烟
152.00	51.50	82.00	22.29	每天	30.00	7.00	做操	从不吸烟
147.00	59.00	80.00	27.30	不锻炼				从不吸烟

150.00	53.00	85.00	23.56	每天	30.00	1.00	散步	从不吸烟
159.00	72.00	94.00	28.48	每天	60.00	2.00	跳舞	从不吸烟
147.00	65.00	92.00	30.08	不锻炼				从不吸烟
152.50	72.00	90.00	30.96	每天	60.00	8.00	跳舞	从不吸烟
160.00	64.00	87.00	25.00	不锻炼				从不吸烟
159.00	55.00	90.00	21.76	每天	30.00	6.00	散步	从不吸烟
163.00	71.20	96.00	26.80	每天	30.00	8.00	散步	从不吸烟
155.00	59.00	86.00	24.56	不锻炼				从不吸烟
166.00	73.00	95.00	26.49	每周一次l	30.00	1.00	散步	从不吸烟
163.00	58.00	79.00	21.83	每天	60.00	17.00	散步	从不吸烟
157.00	70.00	105.00	28.40	每周一次l	30.00	5.00	散步	从不吸烟
161.00	52.00	81.00	20.06	每天	60.00	1.00	散步	从不吸烟
150.00	46.00	90.00	20.44	每天	45.00	1.00	散步	从不吸烟
154.50	37.30	63.00	15.63	不锻炼				从不吸烟
154.00	56.00	84.00	23.61	每天	60.00	16.00	散步	从不吸烟
155.50	68.90	90.00	28.49	不锻炼				从不吸烟
151.00	59.50	85.00	26.10	不锻炼				从不吸烟
140.00	47.00	75.00	23.98	不锻炼				从不吸烟
153.00	64.00	90.00	27.34	每天	30.00	10.00	散步	从不吸烟
146.00	51.50	83.00	24.16	每周一次l	30.00	12.00	散步	从不吸烟
155.00	55.00	85.00	22.89	每周一次l	30.00	3.00	做操	从不吸烟
150.00	47.50	72.00	21.11	每天	60.00	6.00	散步	从不吸烟
161.00	66.00	92.00	25.46	每天	30.00	10.00	散步	从不吸烟
154.00	51.00	69.00	21.50	每天	60.00	2.00	散步	从不吸烟
153.00	56.00	85.00	23.92	不锻炼				从不吸烟
150.50	49.00	79.00	21.63	每周一次l	30.00	5.00	散步	从不吸烟
156.00	60.00	87.00	24.65	每天	30.00	3.00	散步	从不吸烟
161.00	55.00	95.00	21.22	每天	30.00	4.00	散步	从不吸烟
155.00	62.60	87.00	26.06	每天	60.00	12.00	跳舞	从不吸烟
165.00	69.00	82.00	25.34	每周一次l	30.00	1.00	散步	从不吸烟
147.50	55.00	80.00	25.28	每天	60.00	4.00	散步	从不吸烟
156.00	54.00	78.00	22.19	每天	30.00	10.00	散步	从不吸烟
148.00	61.00	97.00	27.85	每天	60.00	1.00	散步	从不吸烟
157.00	65.50	96.00	26.57	每天	30.00	15.00	散步	从不吸烟
148.50	62.60	96.00	28.39	每周一次l	30.00	5.00	散步	从不吸烟
151.50	53.60	83.00	23.35	每天	30.00	6.00	散步	从不吸烟
150.00	40.50	66.00	18.00	每天	30.00	2.00	散步	从不吸烟
146.00	44.00	84.00	20.64	每天	30.00	1.00	散步	从不吸烟
145.00	60.60	88.00	28.82	每天	30.00	10.00	散步	从不吸烟
147.00	52.00	86.00	24.06	不锻炼				从不吸烟
151.00	55.00	87.00	24.12	每天	30.00	4.00	散步	从不吸烟
151.50	69.00	94.00	30.06	每天	30.00	4.00	散步	从不吸烟
155.50	63.00	96.00	26.05	每天	60.00	10.00	散步	从不吸烟
149.50	44.30	88.00	19.82	每天	30.00	6.00	散步	从不吸烟
161.50	70.00	94.00	26.84	每天	30.00	5.00	散步	从不吸烟
149.00	49.00	79.00	22.07	不锻炼				从不吸烟
146.00	56.00	94.00	26.27	每天	30.00	5.00	散步	从不吸烟
152.00	47.30	85.00	20.47	每天	60.00	12.00	打太极	从不吸烟
151.00	63.00	90.00	27.63	每天	60.00	16.00	散步	从不吸烟
152.00	57.40	86.00	24.84	每天	10.00		散步	从不吸烟
162.00	64.00	91.00	24.39	每天	60.00	6.00	散步	从不吸烟

148.00	46.00	82.00	21.00	每天	60.00	10.00	散步	从不吸烟
148.00	64.10	87.00	29.26	每天	60.00	11.00	散步	从不吸烟
150.00	49.70	87.00	22.09	不锻炼				从不吸烟
152.00	67.00	93.00	29.00	每天	60.00	11.00	散步	从不吸烟
153.50	61.00	82.00	25.89	每天	30.00	6.00	散步	从不吸烟
152.00	42.00	79.00	18.18	每天	60.00	2.00	散步	从不吸烟
154.00	66.00	96.00	27.83	每天	60.00	5.00	散步、跳	从不吸烟
143.00	58.00	101.00	28.36	不锻炼				从不吸烟
159.00	70.00	90.00	27.69	每周一次	30.00	1.00	散步	从不吸烟
155.00	62.90	90.00	26.18	不锻炼				从不吸烟
144.00	40.90	69.00	19.72	每天	30.00	11.00	骑自行车	从不吸烟
154.50	52.00	74.00	21.78	每天	60.00	4.00	散步	从不吸烟
149.50	45.60	73.00	20.40	每天	30.00	10.00	打太极	从不吸烟
151.00	61.00	90.00	26.75	每天	30.00	3.00	散步	从不吸烟
163.00	76.00	95.00	28.60	每天	60.00	10.00	跳舞	从不吸烟
150.00	54.00	64.00	24.00	不锻炼				从不吸烟
144.00	42.00	63.00	20.25	每天	30.00	2.00	散步	从不吸烟
154.00	70.00	92.00	29.52	不锻炼				从不吸烟
156.00	48.00	73.00	19.72	每天	30.00	1.00	散步	从不吸烟
146.00	56.00	97.00	26.27	每周一次	30.00	1.00	散步	从不吸烟
143.00	59.00	91.00	28.85	不锻炼				从不吸烟
147.00	60.00	91.00	27.77	每天	30.00	2.00	散步	从不吸烟
156.00	53.00	75.00	21.78	每天	30.00	8.00	散步	从不吸烟
145.00	61.00	100.00	29.01	每天	30.00	2.00	散步	从不吸烟
151.50	51.00	78.00	22.22	每天	60.00	15.00	散步	从不吸烟
155.50	66.30	90.00	27.42	每天	30.00	8.00	散步	从不吸烟
151.00	68.00	97.00	29.82	每周一次	30.00	1.00	散步	从不吸烟
143.00	69.70	104.00	34.08	每天	30.00	1.00	散步	从不吸烟
143.00	35.00	72.00	17.12	每天	60.00	20.00	散步	从不吸烟
148.00	42.00	81.00	19.17	每天	60.00	1.00	散步	从不吸烟
145.00	53.00	84.00	25.21	每周一次	30.00	1.00	散步	从不吸烟
145.00	39.50	70.00	18.79	每天	60.00	7.00	散步	从不吸烟
150.00	66.00	96.00	29.33	每天	30.00	4.00	散步	从不吸烟
144.00	47.50	79.00	22.91	每天	60.00	12.00	散步	从不吸烟
154.00	57.50	73.00	24.25	每天	20.00	2.00	散步	从不吸烟
156.00	53.00	81.00	21.78	每周一次	30.00	2.00	散步	从不吸烟
155.00	61.00	87.00	25.39	每天	60.00	5.00	散步	从不吸烟
156.00	59.00	85.00	24.24	每天	30.00	1.00	散步	从不吸烟
146.00	54.00	84.00	25.33	每天	30.00	10.00	散步	从不吸烟
149.00	61.00	84.00	27.48	每天	45.00	2.00	散步	从不吸烟
150.50	62.00	89.00	27.37	不锻炼				从不吸烟
146.00	51.00	82.00	23.93	每天	60.00	12.00	散步	从不吸烟
150.00	53.00	79.00	23.56	不锻炼				从不吸烟
146.00	50.00	84.00	23.46	每天	30.00	6.00	散步	从不吸烟
158.00	71.50	96.00	28.64	每天	60.00	8.00	散步	从不吸烟
157.00	57.00	92.00	23.12	每天	30.00	7.00	散步	从不吸烟
160.00	83.70	111.00	32.70	每天	60.00	10.00	散步	从不吸烟
148.00	52.00	86.00	23.74	每天	30.00	3.00	散步	从不吸烟
153.50	46.00	75.00	19.52	每天	30.00	3.00	散步	从不吸烟
150.00	54.00	85.00	24.00	每天	30.00	4.00	散步	从不吸烟
158.00	48.00	77.00	19.23	不锻炼				从不吸烟

146.00	57.00	89.00	26.74	不锻炼					从不吸烟
151.00	55.00	85.00	24.12	每天	30.00	3.00	散步		从不吸烟
153.00	60.00	87.00	25.63	不锻炼					从不吸烟
152.00	67.00	96.00	29.00	每天	60.00	11.00	散步		从不吸烟
154.00	60.00	87.00	25.30	每周一次l	30.00	1.00	散步		从不吸烟
157.00	71.00	102.00	28.80	每天	30.00	1.00	散步		从不吸烟
160.00	60.20	86.00	23.52	每天	60.00	4.00	散步		从不吸烟
139.00	50.00	86.00	25.88	每天	60.00	6.00	散步		从不吸烟
154.00	62.00	94.00	26.14	每天	30.00	5.00	散步		从不吸烟
143.00	55.90	92.00	27.34	每天	30.00	10.00	散步		从不吸烟
153.50	64.10	99.00	27.20	每天	30.00	3.00	散步		从不吸烟
151.00	50.00	80.00	21.93	每天	30.00	2.00	散步		从不吸烟
152.00	50.00	78.00	21.64	每周一次l	30.00	1.00	散步		从不吸烟
151.00	59.80	91.00	26.23	每天	60.00	12.00	散步、打		从不吸烟
150.00	61.00	84.00	27.11	每天	60.00	1.00	散步		从不吸烟
151.00	56.40	87.00	24.74	不锻炼					从不吸烟
149.50	66.00	99.00	29.53	每天	30.00	13.00	散步		从不吸烟
152.00	43.00	76.00	18.61	每天	30.00	1.00	散步		从不吸烟
161.00	62.75	84.00	24.21	每周一次l	20.00	5.00	散步		从不吸烟
149.00	63.50	100.00	28.60	每天	30.00	4.00	散步		从不吸烟
153.00	63.00	86.00	26.91	每天	30.00	1.00	散步		从不吸烟
154.50	50.00	85.00	20.95	不锻炼					从不吸烟
152.00	52.50	86.00	22.72	每天	30.00	6.00	散步		从不吸烟
141.00	42.00	70.00	21.13	不锻炼					从不吸烟
155.00	69.00	92.00	28.72	不锻炼					从不吸烟
151.50	52.00	79.00	22.66	每天	30.00	2.00	散步		从不吸烟
148.00	64.80	100.00	29.58	不锻炼					从不吸烟
149.00	37.50	67.00	16.89	每天	20.00	1.00	散步		吸烟
149.00	65.00	95.00	29.28	不锻炼					从不吸烟
150.00	40.00	65.00	17.78	不锻炼					从不吸烟
150.00	57.00	87.00	25.33	不锻炼					从不吸烟
134.00	42.00	88.00	23.39	偶尔	20.00	2.00	散步		从不吸烟
151.00	51.00	81.00	22.37	每天	90.00	3.00	散步		从不吸烟
163.00	68.00	96.00	25.59	不锻炼					从不吸烟
158.00	69.00	97.00	27.64	不锻炼					从不吸烟
147.50	57.00	91.00	26.20	每天	30.00	5.00	散步		从不吸烟
143.00	59.00	90.00	28.85	每天	60.00	7.00	散步		从不吸烟
145.00	50.00	81.00	23.78	不锻炼					从不吸烟
147.00	63.00	88.00	29.15	每天	60.00	10.00	散步		从不吸烟
152.00	59.00	89.00	25.54	不锻炼					从不吸烟
155.00	64.00	95.00	26.64	每天	30.00	1.00	散步		从不吸烟
150.50	53.50	79.00	23.62	每天	60.00	1.00	散步		从不吸烟
153.50	95.00	116.00	40.32	不锻炼					从不吸烟
150.00	62.00	86.00	27.56	每天	30.00	3.00	散步		从不吸烟
154.00	52.00	86.00	21.93	不锻炼					从不吸烟
151.00	51.00	81.00	22.37	每天	30.00	5.00	散步		从不吸烟
151.00	56.30	88.00	24.69	每天	30.00	5.00	散步		从不吸烟
145.00	64.80	98.00	30.82	不锻炼					从不吸烟
144.00	42.00	70.00	20.25	每天	30.00	15.00	散步		从不吸烟
152.00	59.00	95.00	25.54	每天	30.00	1.00	散步		从不吸烟
144.00	43.80	80.00	21.12	每天	30.00	2.00	散步		从不吸烟

153.00	62.00	94.00	26.49	不锻炼				从不吸烟
152.00	65.00	94.00	28.13	每天	45.00	3.00	散步	从不吸烟
158.00	64.00	90.00	25.64	每天	30.00		散步	从不吸烟
153.00	62.00	90.00	26.49	每天	60.00	7.00	散步	从不吸烟
164.50	76.60	97.00	28.31	每天	60.00	8.00	散步，跳	从不吸烟
160.00	68.00	90.00	26.56	每天	30.00	3.00	散步	从不吸烟
160.00	73.00	97.00	28.52	不锻炼				从不吸烟
147.00	60.00	96.00	27.77	每天	30.00	3.00	散步	从不吸烟
138.00	31.40	74.00	16.49	每天	15.00	7.00	散步	从不吸烟
148.00	35.00	56.00	15.98	不锻炼				从不吸烟
152.00	50.00	73.00	21.64	每天	60.00	5.00	散步	从不吸烟
146.00	48.00	86.00	22.52	不锻炼				从不吸烟
145.00	41.50	69.00	19.74	每天	30.00	2.00	散步	从不吸烟
160.00	80.00	104.00	31.25	每天	80.00	7.00	跳舞	从不吸烟
148.00	49.00	80.00	22.37	每天	60.00	7.00	打太极	从不吸烟
152.00	60.00	86.00	25.97	每周一次以	30.00	1.00	散步	从不吸烟
143.00	61.00	95.00	29.83	每周一次以	30.00	1.00	散步	从不吸烟
140.00	40.00	81.00	20.41	不锻炼				从不吸烟
151.00	57.00	87.00	25.00	每天	60.00	1.00	散步	从不吸烟
156.00	65.00	92.00	26.71	不锻炼				从不吸烟
160.00	84.00	100.00	32.81	每周一次以	30.00	3.00	散步	从不吸烟
152.00	55.00	88.00	23.81	每天	30.00	3.00	散步	从不吸烟
146.00	61.00	94.00	28.62	不锻炼				从不吸烟
154.00	66.00	94.00	27.83	每天	60.00	11.00	散步	从不吸烟
145.00	46.00	75.00	21.88	不锻炼				从不吸烟
158.00	52.00	85.00	20.83	每天	60.00	11.00	散步	从不吸烟
158.00	55.00	82.00	22.03	每天	60.00	2.00	散步	从不吸烟
159.00	70.00	94.00	27.69	每天	30.00	1.00	散步	从不吸烟
155.00	61.80	84.00	25.72	不锻炼				从不吸烟
154.00	59.00	75.00	24.88	每周一次以	30.00	1.00	散步	从不吸烟
149.00	62.00	93.00	27.93	每周一次以	30.00	1.00	散步	从不吸烟
145.00	67.60	93.00	32.15	每天	60.00	5.00	散步	从不吸烟
148.00	65.00	96.00	29.67	每周一次以	30.00	1.00	散步	从不吸烟
152.00	40.00	62.00	17.31	每天	30.00	2.00	散步	从不吸烟
155.00	64.50	87.00	26.85	每天	60.00	5.00	散步	从不吸烟
143.00	39.00	69.00	19.07	每天	60.00	3.00	散步	从不吸烟
153.50	72.60	100.00	30.81	每天	30.00	4.00	打球	从不吸烟
152.00	61.00	99.00	26.40	不锻炼				从不吸烟
147.00	50.00	87.00	23.14	不锻炼				从不吸烟
150.00	41.50	70.00	18.44	每天	30.00	1.00	散步	从不吸烟
140.00	55.40	86.00	28.27	不锻炼				从不吸烟
153.00	45.00	62.00	19.22	每天	60.00	1.00	散步	从不吸烟
145.00	59.00	85.00	28.06	每天	60.00	12.00	散步	从不吸烟
155.00	54.00	82.00	22.48	每天	30.00	5.00	散步	从不吸烟
152.50	57.40	82.00	24.68	每天	30.00	2.00	散步	从不吸烟
151.00	55.00	90.00	24.12	每天	60.00	3.00	散步	从不吸烟
153.00	59.00	86.00	25.20	不锻炼				从不吸烟
154.00	61.00	91.00	25.72	每天	30.00	1.00	散步	从不吸烟
156.00	73.00	97.00	30.00	不锻炼				从不吸烟
155.00	62.00	80.00	25.81	不锻炼				从不吸烟
152.00	55.00	87.00	23.81	每天	60.00	2.00	散步	从不吸烟

146.50	45.50	84.00	21.20	每天	30.00	2.00	散步	从不吸烟
143.00	46.00	77.00	22.49	每天	60.00	11.00	散步	从不吸烟
152.00	42.00	70.00	18.18	每天	30.00	3.00	散步	从不吸烟
161.00	61.40	89.00	23.69	每天	60.00	10.00	散步	从不吸烟
140.00	49.40	84.00	25.20	每天	30.00	1.00	散步	从不吸烟
148.00	55.00	87.00	25.11	每天	30.00	6.00	散步	从不吸烟
152.00	60.00	93.00	25.97	每天	30.00	1.00	散步	从不吸烟
136.00	36.00	80.00	19.46	每天	60.00	2.00	散步	从不吸烟
151.00	45.50	81.00	19.96	每天	30.00	2.00	散步、踩	从不吸烟
168.00	72.00	98.00	25.51	不锻炼				从不吸烟
158.00	61.40	89.00	24.60	每天	60.00	12.00	打太极	从不吸烟
153.00	60.00	85.00	25.63	不锻炼				从不吸烟
151.00	39.50	73.00	17.32	不锻炼				从不吸烟
156.00	68.00	99.00	27.94	不锻炼				从不吸烟
146.00	61.00	91.00	28.62	每天	60.00	16.00	散步	从不吸烟
149.00	51.00	90.50	22.97	每天	20.00	1.00	散步	从不吸烟
156.00	37.70	55.00	15.49	每天	30.00	2.00	散步	从不吸烟
152.00	69.00	99.00	29.86	每天	20.00	3.00	散步	从不吸烟
160.00	52.50	89.00	20.51	每天	30.00	12.00	散步	从不吸烟
154.00	68.00	96.00	28.67	每天	60.00	3.00	散步	从不吸烟
149.00	58.00	84.00	26.12	不锻炼				从不吸烟
158.00	56.00	85.00	22.43	每天	60.00	4.00	散步	从不吸烟
146.00	57.00	88.00	26.74	不锻炼				从不吸烟
137.00	40.00	75.00	21.31	不锻炼				从不吸烟
145.00	55.60	97.00	26.44	不锻炼				从不吸烟
152.00	41.50	69.00	17.96	每天	30.00	2.00	散步	从不吸烟
149.00	62.00	92.00	27.93	每天	30.00	3.00	散步	从不吸烟
157.00	56.00	81.00	22.72	每天	30.00	4.00	散步	从不吸烟
156.00	69.00	92.00	28.35	每周一次	20.00	2.00	散步	从不吸烟
149.00	55.00	86.00	24.77	偶尔	30.00	1.00	散步	从不吸烟
151.50	56.60	78.00	24.66	每天	30.00	1.00	散步	从不吸烟
148.00	50.00	85.00	22.83	每天	30.00	3.00	散步	从不吸烟
156.50	60.00	87.00	24.50	每天	60.00	8.00	散步	从不吸烟
167.00	60.00	88.00	21.51	每天	30.00	10.00	散步	从不吸烟
152.00	55.00	83.00	23.81	每天	30.00	1.00	散步	从不吸烟
149.00	63.50	88.00	28.60	每天	30.00	2.00	散步	从不吸烟
145.00	53.00	76.00	25.21	每天	30.00	10.00	散步	从不吸烟
151.00	68.00	93.00	29.82	每天	30.00	6.00	散步	从不吸烟
158.00	84.00	97.00	33.65	每天	60.00	7.00	散步	从不吸烟
150.00	65.30	95.00	29.02	每天	30.00	2.00	散步	从不吸烟
153.00	68.60	96.00	29.30	每天	30.00	1.00	散步	从不吸烟
145.00	36.00	71.00	17.12	不锻炼				从不吸烟
154.00	54.00	84.00	22.77	每天	30.00	1.00	散步	从不吸烟
151.00	64.00	93.00	28.07	每天	60.00	5.00	散步	从不吸烟
148.50	44.00	67.00	19.95	不锻炼				从不吸烟
152.00	50.00	82.00	21.64	每天	30.00	7.00	散步	从不吸烟
153.00	53.00	73.00	22.64	每天	30.00	3.00	散步	从不吸烟
153.00	63.00	89.00	26.91	每天	60.00	4.00	散步	从不吸烟
146.00	58.30	96.00	27.35	每天	60.00	8.00	散步	从不吸烟
146.00	53.00	83.00	24.86	每天	30.00	7.00	散步	从不吸烟
151.00	63.00	90.00	27.63	每天	30.00	16.00	散步	从不吸烟

140.00	45.00	80.00	22.96	偶尔	10.00	1.00	散步	从不吸烟
144.00	56.00	90.00	27.01	每天	30.00	5.00	散步	从不吸烟
146.00	53.00	94.00	24.86	不锻炼				从不吸烟
147.00	38.00	72.00	17.59	不锻炼				从不吸烟
150.00	55.80	86.00	24.80	每天	30.00	3.00	散步	从不吸烟
155.00	59.50	93.00	24.77	每天	30.00	2.00	散步	从不吸烟
161.00	55.00	80.00	21.22	每周一次	30.00	1.00	散步	从不吸烟
153.00	52.00	88.00	22.21	每天	60.00	6.00	散步	从不吸烟
145.50	44.60	87.50	21.07	每天	30.00	6.00	散步	从不吸烟
150.00	49.00	80.00	21.78	每天	30.00	1.00	散步	从不吸烟
150.00	43.00	69.00	19.11	每天	60.00	1.00	散步	从不吸烟
148.00	55.00	87.00	25.11	每天	60.00	3.00	散步	从不吸烟
148.00	63.80	91.00	29.13	每天	60.00	1.00	体操	从不吸烟
152.00	45.60	74.00	19.74	不锻炼				从不吸烟
151.00	38.00	62.00	16.67	不锻炼				从不吸烟
152.00	67.00	88.00	29.00	每天	30.00	18.00	散步	从不吸烟
156.00	61.00	87.00	25.07	不锻炼				从不吸烟
151.00	56.00	86.00	24.56	每天	30.00	6.00	散步	从不吸烟
152.00	53.30	81.00	23.07	不锻炼				从不吸烟
149.00	61.00	92.00	27.48	每天	30.00	7.00	散步	从不吸烟
148.00	36.60	64.00	16.71	每天	30.00	4.00	散步	从不吸烟
155.00	59.20	85.00	24.64	每天	60.00	7.00	散步	从不吸烟
159.00	53.00	84.00	20.96	每天	60.00	4.00	散步、骑	从不吸烟
155.00	60.00	85.00	24.97	每天	30.00	10.00	散步	从不吸烟
157.00	42.00	65.00	17.04	每周一次	30.00	2.00	散步	从不吸烟
153.00	51.00	78.00	21.79	每周一次	30.00	1.00	散步	从不吸烟
145.00	45.00	78.00	21.40	每天	30.00	6.00	散步	从不吸烟
155.00	54.00	87.00	22.48	每天	15.00	1.00	散步	从不吸烟
151.00	59.00	86.00	25.88	不锻炼				从不吸烟
145.00	45.00	75.00	21.40	每天	30.00	2.00	散步	从不吸烟
148.00	60.00	87.00	27.39	每周一次	30.00	1.00	散步	从不吸烟
133.00	29.00	73.00	16.39	每天	60.00	7.00	散步	从不吸烟
153.00	64.00	94.00	27.34	每天	30.00	3.00	散步	从不吸烟
146.50	55.00	83.00	25.63	每天	60.00	3.00	散步	从不吸烟
159.50	70.00	93.00	27.52	每天	30.00	3.00	散步	从不吸烟
144.00	52.00	82.00	25.08	每周一次	15.00	1.00	被动运动	从不吸烟
146.00	58.00	92.00	27.21	每周一次	30.00	2.00	散步	从不吸烟
159.00	61.00	83.00	24.13	每天	60.00	1.00	散步	从不吸烟
148.00	45.00	75.00	20.54	每天	30.00	6.00	散步	从不吸烟
150.00	54.00	88.00	24.00	每天	60.00	6.00	散步	从不吸烟
138.00	43.00	84.00	22.58	不锻炼				从不吸烟
150.00	49.00	77.00	21.78	每天	50.00	10.00	散步	从不吸烟
148.00	54.70	93.00	24.97	不锻炼				从不吸烟
150.00	63.00	96.00	28.00	每天	30.00	6.00	散步	从不吸烟
150.00	46.00	83.00	20.44	每天	30.00	2.00	散步	从不吸烟
152.00	62.10	86.00	26.88	每天	60.00	2.00	体操	从不吸烟
146.00	51.80	88.00	24.30	每天	60.00	6.00	散步	从不吸烟
150.00	61.00	90.00	27.11	不锻炼				从不吸烟
152.00	66.50	92.00	28.78	每天	60.00	4.00	散步	从不吸烟
155.50	57.00	86.00	23.57	每天	30.00	4.00	散步	从不吸烟
162.50	58.00	80.00	21.96	每天	60.00	10.00	散步	从不吸烟

155.50	57.30	88.00	23.70	每天	60.00	5.00	散步	从不吸烟
155.00	60.00	80.00	24.97	每天	30.00	6.00	散步	从不吸烟
162.00	58.00	83.00	22.10	每天	40.00	4.00	散步	从不吸烟
149.00	34.05	78.00	15.34	每天	10.00	2.00	散步	从不吸烟
152.00	64.00	91.00	27.70	每天	60.00	2.00	散步	从不吸烟
157.00	52.50	77.00	21.30	每天	30.00	2.00	散步	从不吸烟
144.00	41.00	68.00	19.77	每天	30.00	3.00	散步	从不吸烟
139.00	56.00	88.00	28.98	每天	30.00	1.00	散步	从不吸烟
144.00	60.00	90.00	28.94	每天	90.00		散步	从不吸烟
154.00	67.00	92.00	28.25	每天	60.00	2.00	散步、游i	从不吸烟
150.00	57.00	86.00	25.33	每天	30.00	3.00	散步	从不吸烟
148.00	60.70	99.00	27.71	不锻炼				从不吸烟
143.00	46.00	78.00	22.49	不锻炼				从不吸烟
147.00	39.10	68.00	18.09	每天	60.00	2.00	散步	从不吸烟
151.00	49.00	80.00	21.49	不锻炼				从不吸烟
148.00	48.10	89.00	21.96	每天	15.00	2.00	散步	从不吸烟
152.00	59.00	92.00	25.54	每天	60.00	8.00	散步	从不吸烟
152.50	60.00	89.00	25.80	不锻炼				从不吸烟
160.00	60.80	85.00	23.75	不锻炼				从不吸烟
156.00	63.00	86.00	25.89	每天	20.00	1.00	散步	从不吸烟
146.00	54.50	89.00	25.57	不锻炼				从不吸烟
156.00	66.00	98.00	27.12	每天	30.00	2.00	散步	从不吸烟
146.50	49.40	90.50	23.02	每天	30.00	10.00	散步	从不吸烟
151.00	55.00	84.00	24.12	每天	30.00	4.00	散步	从不吸烟
153.00	52.00	78.00	22.21	每天	30.00	4.00	散步	从不吸烟
155.50	61.00	85.00	25.23	每天	60.00	4.00	散步	从不吸烟
155.00	60.45	99.00	25.16	不锻炼				从不吸烟
145.00	65.00	98.00	30.92	每天	120.00	8.00	散步	从不吸烟
153.00	60.00	85.00	25.63	每天	60.00	2.00	散步	从不吸烟
146.50	39.50	73.00	18.40	每天	30.00	4.00	散步	从不吸烟
145.00	45.00	73.00	21.40	每天	20.00	1.00	散步	从不吸烟
148.00	52.00	85.00	23.74	不锻炼				从不吸烟
152.00	64.00	86.00	27.70	不锻炼				从不吸烟
152.00	56.50	88.00	24.45	不锻炼				从不吸烟
152.00	54.00	82.00	23.37	每天	30.00	2.00	散步	从不吸烟
150.00	42.00	74.00	18.67	不锻炼				从不吸烟
148.00	42.00	71.00	19.17	每天	60.00	1.00	散步	从不吸烟
153.00	45.50	70.00	19.44	每天	30.00	1.00	散步	从不吸烟
145.00	63.50	89.00	30.20	不锻炼				从不吸烟
155.00	43.00	70.00	17.90	每天	30.00	4.00	散步	从不吸烟
157.50	65.50	88.00	26.40	每天	60.00	15.00	散步	从不吸烟
158.00	62.00	90.00	24.84	每天	30.00	5.00	散步	从不吸烟
145.00	40.00	61.00	19.02	每天	30.00	1.00	散步	从不吸烟
150.00	58.00	87.00	25.78	每天	30.00	4.00	散步	从不吸烟
152.00	64.00	97.00	27.70	不锻炼				从不吸烟
153.00	60.00	95.00	25.63	不锻炼				从不吸烟
145.00	42.00	83.00	19.98	每天	30.00	2.00	散步	从不吸烟
158.00	51.60	81.00	20.67	每天	30.00	4.00	散步	从不吸烟
154.50	60.80	90.00	25.47	每天	30.00	4.00	散步	从不吸烟
139.00	42.00	80.00	21.74	不锻炼				从不吸烟
154.00	48.00	76.00	20.24	每周一次!	30.00	1.00	散步	从不吸烟

157.00	69.00	101.00	27.99	每天	60.00	1.00	散步	从不吸烟
146.00	49.00	84.00	22.99	每周一次	30.00	1.00	散步	从不吸烟
160.00	65.00	94.00	25.39	每天	30.00	2.00	散步	从不吸烟
153.00	64.00	101.00	27.34	不锻炼				从不吸烟
144.00	58.00	91.00	27.97	每天	30.00	11.00	散步	从不吸烟
150.00	42.00	69.00	18.67	每天	30.00	3.00	散步	从不吸烟
162.00	72.00	92.00	27.43	每天	30.00	7.00	散步	从不吸烟
157.00	62.00	98.00	25.15	不锻炼				从不吸烟
148.00	50.00	72.00	22.83	不锻炼				从不吸烟
158.50	59.40	83.00	23.64	每天	30.00	4.00	散步	从不吸烟
145.00	44.00	90.00	20.93	不锻炼				从不吸烟
162.50	84.00	105.00	31.81	每天	30.00	5.00	散步	从不吸烟
152.00	54.20	81.00	23.46	不锻炼				从不吸烟
145.50	36.50	64.00	17.24	不锻炼				从不吸烟
138.00	44.00	86.00	23.10	不锻炼				从不吸烟
148.00	54.70	88.00	24.97	每天	60.00	10.00	散步	从不吸烟
153.00	60.00	94.00	25.63	每天	30.00	4.00	散步	从不吸烟
153.00	61.00	94.00	26.06	每周一次	60.00	1.00	散步	从不吸烟
156.50	61.00	85.00	24.91	每天	60.00	4.00	散步	从不吸烟
149.00	52.00	86.00	23.42	每天	30.00	2.00	散步	从不吸烟
146.00	45.00	70.00	21.11	不锻炼				从不吸烟
142.50	63.35	101.00	31.20	不锻炼				从不吸烟
143.00	54.00	83.00	26.41	不锻炼				从不吸烟
147.00	55.00	90.00	25.45	每天	30.00	5.00	散步	从不吸烟
150.00	60.60	94.00	26.93	不锻炼				从不吸烟
158.00	42.00	68.00	16.82	不锻炼				从不吸烟
164.00	66.00	86.00	24.54	每天	30.00		散步	从不吸烟
148.00	41.50	66.00	18.95	每天	90.00	20.00	跳舞	从不吸烟
150.00	56.00	88.00	24.89	每天	30.00	5.00	跳舞	从不吸烟
154.50	51.10	79.00	21.41	每天	120.00	10.00	散步	从不吸烟
152.00	63.00	81.00	27.27	每周一次	30.00	1.00	散步	从不吸烟
143.00	50.50	91.00	24.70	不锻炼				从不吸烟
152.00	54.00	86.00	23.37	不锻炼				从不吸烟
154.00	44.00	66.00	18.55	每天	60.00	1.00	散步	从不吸烟
156.00	63.00	92.00	25.89	每天	30.00	11.00	散步	从不吸烟
154.00	46.00	80.00	19.40	不锻炼				从不吸烟
144.00	36.00	68.00	17.36	每天	30.00	4.00	散步	从不吸烟
150.00	54.00	87.00	24.00	每天	30.00	5.00	散步	从不吸烟
156.00	68.00	96.00	27.94	每天	30.00	2.00	散步	从不吸烟
150.00	49.00	79.00	21.78	每周一次	40.00	3.00	散步	从不吸烟
139.00	40.00	81.00	20.70	不锻炼				从不吸烟
150.00	49.00	86.00	21.78	每天	30.00	4.00	散步	从不吸烟
143.00	58.50	88.00	28.61	不锻炼				从不吸烟
153.00	56.00	82.00	23.92	每天	60.00	2.00	散步	从不吸烟
158.00	76.80	100.00	30.76	每天	60.00	1.00	散步	从不吸烟
150.00	46.10	75.00	20.49	每天	60.00	5.00	散步	从不吸烟
158.00	46.00	72.00	18.43	每天	60.00	1.00	散步	从不吸烟
151.00	53.00	83.00	23.24	每天	30.00	5.00	散步	从不吸烟
151.00	65.50	92.00	28.73	每天	60.00	4.00	散步	从不吸烟
143.00	58.00	81.00	28.36	每天	30.00	1.00	散步	从不吸烟
152.00	55.70	84.00	24.11	每天	60.00	1.00	散步	从不吸烟

147.00	70.00	94.00	32.39	每天	30.00	7.00	散步	从不吸烟
159.00	79.00	92.00	31.25	每天	40.00	1.00	散步	从不吸烟
151.00	58.80	87.00	25.79	每天	60.00	2.00	散步	从不吸烟
152.00	47.00	73.00	20.34	每天	30.00	1.00	跑步	从不吸烟
154.00	61.50	88.00	25.93	每天	120.00	2.00	散步	从不吸烟
148.00	39.00	64.00	17.80	每天	15.00	8.00	体操	从不吸烟
144.00	40.00	71.00	19.29	每周一次!	30.00	1.00	散步	从不吸烟
156.00	61.00	94.00	25.07	每天	30.00	6.00	散步	从不吸烟
144.00	57.00	92.00	27.49	每天	30.00	10.00	散步	从不吸烟
146.50	63.00	101.00	29.35	不锻炼				从不吸烟
151.00	60.00	93.00	26.31	每周一次!	30.00	2.00	散步	从不吸烟
152.00	57.90	89.00	25.06	每天	60.00	18.00	散步	从不吸烟
150.00	62.50	86.00	27.78	每天	40.00	1.00	散步	从不吸烟
138.00	45.00	76.00	23.63	不锻炼				从不吸烟
156.00	71.00	104.00	29.17	每天	30.00	6.00	散步	从不吸烟
159.50	69.50	91.00	27.32	每天	60.00	7.00	散步	从不吸烟
156.00	63.50	86.00	26.09	每天	30.00	6.00	跳舞	从不吸烟
155.00	51.00	84.00	21.23	每天	30.00	4.00	太极	从不吸烟
155.00	51.00	68.00	21.23	每天	40.00	1.00	散步	从不吸烟
150.00	48.30	82.00	21.47	每天	60.00	4.00	散步	从不吸烟
143.00	33.50	61.00	16.38	每天	60.00	11.00	散步	从不吸烟
145.00	50.00	85.00	23.78	每天	60.00	12.00	散步	从不吸烟
142.00	39.00	86.00	19.34	每天	20.00	7.00	散步	从不吸烟
145.00	59.00	100.00	28.06	每天	20.00	6.00	散步	从不吸烟
143.00	55.00	90.00	26.90	每天	30.00	8.00	散步	从不吸烟
149.00	51.00	72.00	22.97	每天	60.00	4.00	散步	从不吸烟
141.00	49.00	84.00	24.65	每天	30.00	2.00	散步	从不吸烟
146.00	62.50	86.00	29.32	每天	60.00	7.00	跳舞	从不吸烟
148.00	54.00	86.00	24.65	每天	60.00	3.00	散步	从不吸烟
151.00	37.50	60.00	16.45	不锻炼				从不吸烟
149.00	62.50	97.00	28.15	每天	60.00	3.00	散步	从不吸烟
151.00	52.00	84.00	22.81	每周一次!	30.00	1.00	散步	从不吸烟
157.00	59.00	90.00	23.94	每天	120.00	16.00	散步	从不吸烟
145.00	50.00	93.00	23.78	每周一次!	30.00	5.00	散步	从不吸烟
157.00	45.00	62.00	18.26	每天	30.00	10.00	散步	吸烟
136.00	35.50	69.00	19.19	不锻炼				从不吸烟
153.00	53.50	84.00	22.85	不锻炼				从不吸烟
156.00	62.00	95.00	25.48	每天	60.00	5.00	打乒乓球、	从不吸烟
152.00	56.00	87.00	24.24	每天	30.00	2.00	散步	从不吸烟
153.00	71.00	95.00	30.33	每周一次!	30.00	3.00	散步	从不吸烟
151.50	68.00	96.00	29.63	每天	30.00	13.00	散步	从不吸烟
133.00	39.00	78.00	22.05	每天	30.00	3.00	散步	从不吸烟
156.00	65.00	86.00	26.71	不锻炼				从不吸烟
154.00	71.70	101.00	30.23	不锻炼				从不吸烟
154.00	57.00	81.00	24.03	不锻炼				从不吸烟
150.00	55.00	86.00	24.44	不锻炼				从不吸烟
155.00	54.00	81.00	22.48	每天	60.00	4.00	散步	从不吸烟
155.00	51.50	79.00	21.44	每天	30.00	2.00	散步	从不吸烟
153.00	68.00	92.00	29.05	每天	30.00	4.00	散步	从不吸烟
161.00	54.00	84.00	20.83	每周一次!	30.00	10.00	散步	从不吸烟
155.00	64.00	93.00	26.64	不锻炼				从不吸烟

154.00	44.00	71.00	18.55	每天	30.00	1.00	散步	从不吸烟
154.00	54.00	86.00	22.77	每天	30.00	6.00	散步	从不吸烟
152.00	60.00	88.00	25.97	每天	30.00	1.00	散步	从不吸烟
154.00	74.00	95.00	31.20	每周一次以	30.00	1.00	散步	从不吸烟
159.50	50.00	79.00	19.65	每天	60.00	6.00	散步	从不吸烟
147.00	60.50	88.00	28.00	每天	60.00	5.00	散步	从不吸烟
143.00	49.00	87.00	23.96	不锻炼				从不吸烟
152.00	50.00	72.00	21.64	不锻炼				从不吸烟
160.00	62.00	94.00	24.22	每天	60.00	2.00	散步	从不吸烟
155.00	67.00	101.00	27.89	每天	60.00	1.00	散步	从不吸烟
148.00	52.00	80.00	23.74	不锻炼				从不吸烟
154.00	60.00	83.00	25.30	每天	30.00	12.00	散步	从不吸烟
148.00	60.00	98.00	27.39	不锻炼				从不吸烟
149.00	65.00	102.00	29.28	不锻炼				从不吸烟
152.00	58.80	89.00	25.45	不锻炼				从不吸烟
149.00	62.50	90.00	28.15	不锻炼				从不吸烟
161.00	61.50	98.00	23.73	每天	60.00	3.00	散步	从不吸烟
150.00	43.00	67.00	19.11	每天	60.00	5.00	散步	从不吸烟
149.00	58.00	86.00	26.12	每天	30.00	12.00	散步	从不吸烟
144.00	65.00	108.00	31.35	每周一次以	20.00	2.00	散步	从不吸烟
162.00	79.50	104.00	30.29	每天	60.00	4.00	散步	从不吸烟
157.00	66.00	90.00	26.78	每周一次以	30.00	2.00	散步	从不吸烟
154.00	52.00	82.00	21.93	每天	30.00	9.00	散步	从不吸烟
157.50	54.80	89.00	22.09	每周一次以	30.00	1.00	散步	从不吸烟
154.00	42.00	77.00	17.71	每天	30.00	2.00	散步	从不吸烟
152.00	54.00	89.00	23.37	不锻炼				从不吸烟
155.00	58.30	79.00	24.27	每天	60.00	6.00	散步	从不吸烟
148.00	50.00	85.00	22.83	不锻炼				从不吸烟
147.00	54.00	88.00	24.99	不锻炼				从不吸烟
151.00	58.50	89.00	25.66	每天	30.00	2.00	散步	从不吸烟
155.00	59.00	98.00	24.56	每天	15.00	1.00	散步	从不吸烟
141.00	43.00	84.00	21.63	每天	40.00	12.00	散步	从不吸烟
159.00	67.00	98.00	26.50	每天	30.00	1.00	散步,打太	从不吸烟
146.00	39.00	72.00	18.30	不锻炼				从不吸烟
150.00	60.50	91.00	26.89	每天	30.00	1.00	散步	从不吸烟
150.00	69.00	99.00	30.67	不锻炼				从不吸烟
151.00	45.00	70.00	19.74	不锻炼				从不吸烟
158.00	45.00	65.00	18.03	每天	60.00	1.00	散步	从不吸烟
138.00	39.00	81.00	20.48	每天	60.00	1.00	散步	从不吸烟
143.00	49.00	82.00	23.96	每天	30.00	5.00	做操	从不吸烟
146.00	53.00	90.00	24.86	不锻炼				从不吸烟
157.00	60.00	89.00	24.34	不锻炼				从不吸烟
150.00	62.00	87.00	27.56	每天	30.00	2.00	散步	从不吸烟
146.00	78.00	106.00	36.59	每天	40.00	4.00	散步	从不吸烟
157.00	77.00	99.00	31.24	每天	30.00	5.00	散步	从不吸烟
151.00	48.00	72.00	21.05	每天	30.00	1.00	散步	从不吸烟
145.00	45.00	72.00	21.40	每天	30.00	2.00	保健操	从不吸烟
152.00	51.00	76.00	22.07	每天	30.00	4.00	散步	从不吸烟
154.00	61.00	91.00	25.72	每天	30.00	1.00	散步	从不吸烟
151.00	56.00	89.00	24.56	每周一次以	30.00	1.00	散步	吸烟
160.00	63.00	76.00	24.61	每天	30.00	1.00	散步	从不吸烟

146.50	43.30	70.00	20.17	每周一次!	30.00	3.00	散步	从不吸烟
148.00	55.00	88.00	25.11	不锻炼				从不吸烟
146.00	41.00	72.00	19.23	每天	30.00	7.00	散步	从不吸烟
150.50	55.00	86.00	24.28	不锻炼				从不吸烟
152.00	63.00	90.00	27.27	不锻炼				从不吸烟
154.00	63.00	94.00	26.56	每天	40.00	10.00	散步	从不吸烟
158.00	46.00	75.00	18.43	每天	60.00	12.00	散步	从不吸烟
152.00	65.00	90.00	28.13	每天	30.00	2.00	散步	从不吸烟
147.00	54.00	89.00	24.99	每天	20.00	6.00	散步	从不吸烟
155.00	62.00	89.00	25.81	每天	30.00	10.00	散步	从不吸烟
139.50	41.10	78.50	21.12	不锻炼				从不吸烟
149.00	44.00	83.00	19.82	每天	30.00	1.00	散步	从不吸烟
142.00	46.00	84.00	22.81	每天	60.00	4.00	散步	从不吸烟
144.00	50.00	88.00	24.11	每天	60.00	2.00	散步	从不吸烟
146.00	40.00	77.00	18.77	不锻炼				从不吸烟
148.00	54.80	90.00	25.02	每天	20.00	1.00	散步	从不吸烟
153.30	40.00	76.00	17.02	每周一次!	60.00	4.00	散步	从不吸烟
150.00	56.50	85.00	25.11	每周一次!	30.00	5.00	散步	从不吸烟
151.00	50.00	82.00	21.93	每天	60.00	7.00	散步	从不吸烟
152.00	46.00	84.00	19.91	不锻炼				从不吸烟
148.00	50.50	86.00	23.06	不锻炼				从不吸烟
143.00	51.00	84.00	24.94	每天	30.00	7.00	散步	从不吸烟
148.00	51.00	83.00	23.28	每天	60.00	1.00	散步	从不吸烟
147.00	51.00	75.00	23.60	每周一次!	30.00	1.00	散步	从不吸烟
147.00	40.50	70.00	18.74	每天	60.00	6.00	散步	从不吸烟
145.00	58.00	88.00	27.59	每天	30.00	6.00	散步	从不吸烟
148.00	48.00	84.00	21.91	每周一次!	10.00	1.00	被动运动	从不吸烟
153.00	60.50	88.00	25.84	每天	30.00	1.00	散步	从不吸烟
156.00	51.00	78.00	20.96	每天	30.00	2.00	散步	从不吸烟
168.00	73.00	95.00	25.86	每天	30.00	16.00	散步	从不吸烟
144.00	53.00	85.00	25.56	不锻炼				从不吸烟
148.00	58.00	84.00	26.48	每天	60.00	2.00	散步	从不吸烟
143.00	44.00	74.00	21.52	不锻炼				从不吸烟
158.00	55.50	77.00	22.23	每天	30.00	2.00	散步	从不吸烟
140.00	40.00	76.00	20.41	不锻炼				从不吸烟
158.00	79.80	99.00	31.97	每天	30.00	4.00	散步	从不吸烟
144.00	47.00	73.00	22.67	每天	30.00	1.00	散步	从不吸烟
150.00	50.00	89.00	22.22	不锻炼				从不吸烟
151.00	61.00	93.00	26.75	不锻炼				从不吸烟
153.00	64.50	87.00	27.55	不锻炼				从不吸烟
155.00	57.00	96.00	23.73	每天	30.00	1.00	散步	从不吸烟
150.00	51.00	78.00	22.67	每周一次!	30.00	5.00	散步	从不吸烟
157.00	73.00	98.00	29.62	每天	30.00	1.00	散步	从不吸烟
150.00	55.00	92.00	24.44	不锻炼				从不吸烟
160.00	67.20	99.00	26.25	每天	20.00	5.00	散步	从不吸烟
150.00	63.50	89.00	28.22	每天	30.00	5.00	散步	从不吸烟
150.00	69.60	96.00	30.93	每天	30.00	2.00	散步	从不吸烟
146.00	55.50	91.00	26.04	每天	60.00	4.00	散步	从不吸烟
150.00	55.50	87.00	24.67	每天	60.00	5.00	跳舞	从不吸烟
151.00	54.30	93.00	23.81	每天	45.00	10.00	散步	从不吸烟
153.00	82.90	111.00	35.41	不锻炼				从不吸烟

153.00	56.60	78.00	24.18	不锻炼				从不吸烟
153.00	66.00	91.00	28.19	每天	60.00	2.00	散步	从不吸烟
137.00	35.00	74.00	18.65	每天	60.00	8.00	散步	从不吸烟
156.50	65.00	88.00	26.54	每周一次	60.00	13.00	散步	从不吸烟
155.00	51.00	80.00	21.23	不锻炼				从不吸烟
155.00	44.00	79.00	18.31	每天	30.00	27.00	散步	从不吸烟
145.00	51.00	90.00	24.26	每天	30.00	11.00	散步	从不吸烟
151.00	66.20	92.00	29.03	每天	30.00	1.00	散步	从不吸烟
149.00	49.10	78.00	22.12	每天	60.00	13.00	散步	从不吸烟
154.00	62.80	94.00	26.48	每天	60.00	7.00	散步	从不吸烟
141.50	52.40	90.00	26.17	每天	30.00	2.00	散步	从不吸烟
162.00	65.00	97.00	24.77	每天	60.00	5.00	打拳，健	从不吸烟
155.00	66.00	90.00	27.47	每天	30.00	2.00	散步	从不吸烟
163.00	75.00	105.00	28.23	每周一次	60.00	3.00	散步	从不吸烟
152.00	57.00	83.00	24.67	不锻炼				从不吸烟
150.00	52.50	83.00	23.33	每天	60.00	15.00	打太极	从不吸烟
152.00	60.00	91.00	25.97	每天	30.00	1.00	做操	从不吸烟
146.00	50.00	83.00	23.46	每周一次	30.00	1.00	散步	从不吸烟
154.00	54.00	82.00	22.77	不锻炼				从不吸烟
150.00	35.50	66.00	15.78	每天	60.00	2.00	散步	从不吸烟
163.00	56.50	87.00	21.27	每天	40.00	2.00	散步	从不吸烟
143.00	58.00	89.00	28.36	每天	60.00	12.00	散步，跳	从不吸烟
154.50	62.00	87.00	25.97	不锻炼				从不吸烟
156.00	65.00	91.00	26.71	不锻炼				从不吸烟
157.00	62.00	91.00	25.15	每天	50.00	11.00	散步	从不吸烟
155.50	80.50	98.00	33.29	每天	60.00	1.00	散步	从不吸烟
146.50	53.10	87.00	24.74	每天	60.00	5.00	散步	从不吸烟
159.00	43.00	77.00	17.01	每周一次	20.00	5.00	散步	从不吸烟
152.50	58.50	90.00	25.15	每天	30.00	5.00	散步	从不吸烟
151.00	48.00	88.00	21.05	每天	60.00	4.00	散步	从不吸烟
147.00	57.50	90.00	26.61	每天	60.00	3.00	散步	从不吸烟
143.00	56.50	88.00	27.63	每天	30.00	2.00	散步	从不吸烟
147.00	54.00	84.00	24.99	不锻炼				从不吸烟
150.00	63.00	93.00	28.00	每天	25.00	2.00	散步	从不吸烟
158.50	48.50	74.00	19.31	不锻炼				从不吸烟
144.00	52.00	82.00	25.08	每天	60.00	2.00	散步	从不吸烟
158.50	63.40	89.00	25.24	每天	30.00	13.00	打太极	从不吸烟
151.00	50.00	79.00	21.93	每周一次	30.00	1.00	散步	从不吸烟
153.00	62.00	90.00	26.49	不锻炼				从不吸烟
152.00	61.00	83.00	26.40	每天	30.00	9.00	散步	从不吸烟
145.00	54.00	90.00	25.68	每天	60.00	8.00	散步	从不吸烟
143.00	50.00	88.00	24.45	不锻炼				从不吸烟
165.00	75.00	95.00	27.55	每周一次	30.00	2.00	散步	从不吸烟
149.00	58.50	84.00	26.35	每天	60.00	5.00	散步	从不吸烟
157.00	59.00	70.00	23.94	每天	35.00	1.00	晨操	从不吸烟
152.00	79.00	103.00	34.19	每周一次	30.00	5.00	散步	从不吸烟
149.00	54.00	85.00	24.32	每天	20.00	8.00	散步	从不吸烟
158.00	54.00	88.00	21.63	不锻炼				从不吸烟
150.50	49.00	76.00	21.63	每天	30.00	10.00	散步	从不吸烟
153.00	45.40	82.00	19.39	每天	30.00	5.00	散步	从不吸烟
158.00	59.00	82.00	23.63	不锻炼				从不吸烟

150.00	62.00	91.00	27.56	每天	30.00	6.00	散步	从不吸烟
160.00	75.00	89.00	29.30	每天	60.00	15.00	散步、打	从不吸烟
147.50	65.40	88.00	30.06	不锻炼				从不吸烟
147.00	58.00	95.00	26.84	每天	30.00	12.00	散步	从不吸烟
150.00	46.00	78.00	20.44	每天	30.00	6.00	散步	从不吸烟
158.00	74.00	94.00	29.64	每天	60.00	2.00	散步	从不吸烟
144.00	46.00	71.00	22.18	每天	60.00	14.00	散步	从不吸烟
150.00	58.40	94.00	25.96	每天	60.00	6.00	散步	从不吸烟
149.00	58.00	94.00	26.12	每天	60.00	11.00	散步	从不吸烟
151.00	50.00	75.00	21.93	每天	30.00	1.00	散步	从不吸烟
154.00	52.00	85.00	21.93	每天	60.00	13.00	散步	从不吸烟
147.50	53.30	92.00	24.50	每天	30.00	3.00	散步	从不吸烟
155.00	55.70	84.00	23.18	每天	40.00	6.00	散步	从不吸烟
152.00	48.00	84.00	20.78	不锻炼				从不吸烟
157.00	60.40	83.00	24.50	每天	30.00	10.00	散步	从不吸烟
146.00	54.00	77.00	25.33	每天	30.00	4.00	散步	从不吸烟
154.00	80.00	93.00	33.73	每天	60.00	3.00	散步	从不吸烟
149.00	53.00	85.00	23.87	每周一次	15.00	2.00	散步	从不吸烟
151.00	51.00	79.00	22.37	每天	15.00	5.00	做操	从不吸烟
160.00	48.00	75.00	18.75	不锻炼				从不吸烟
145.00	66.00	90.00	31.39	每天	30.00	4.00	散步	从不吸烟
161.00	67.65	83.00	26.10	每天	30.00	1.00	散步	从不吸烟
155.00	57.00	85.00	23.73	每天	30.00	12.00	散步	从不吸烟
150.50	66.00	96.00	29.14	每天	30.00	1.00	散步	从不吸烟
154.50	53.50	76.00	22.41	不锻炼				从不吸烟
153.00	49.00	83.00	20.93	每天	30.00	10.00	散步	从不吸烟
142.00	57.00	90.00	28.27	不锻炼				从不吸烟
154.00	62.00	86.00	26.14	每天	30.00	2.00	散步	从不吸烟
154.00	54.00	78.00	22.77	每周一次	30.00	1.00	散步	从不吸烟
156.00	54.40	81.00	22.35	每天	60.00	4.00	散步	从不吸烟
146.50	60.40	99.00	28.14	每天	30.00	20.00	做操	从不吸烟
148.00	52.80	90.00	24.11	每天	30.00	10.00	散步	从不吸烟
152.00	67.00	105.00	29.00	每天	30.00	5.00	散步	从不吸烟
143.00	50.00	87.00	24.45	不锻炼				从不吸烟
146.00	50.00	78.00	23.46	每天	30.00	4.00	散步	从不吸烟
154.00	67.30	99.00	28.38	不锻炼				从不吸烟
150.00	62.00	92.00	27.56	不锻炼				从不吸烟
157.00	63.00	86.00	25.56	每天	60.00	10.00	散步	从不吸烟
144.00	36.20	60.00	17.46	每天	40.00	12.00	散步	从不吸烟
153.00	50.00	75.00	21.36	每天	30.00	5.00	散步	从不吸烟
155.00	51.00	79.00	21.23	每天	60.00	8.00	散步	从不吸烟
143.00	55.00	85.00	26.90	每天	60.00	2.00	做操	从不吸烟
153.50	55.50	79.00	23.55	每天	60.00	20.00	散步	从不吸烟
157.00	61.40	99.00	24.91	不锻炼				从不吸烟
142.00	56.00	86.00	27.77	每天	30.00	4.00	散步	从不吸烟
144.00	52.00	79.00	25.08	每天	30.00	1.00	散步	从不吸烟
149.50	54.20	95.00	24.25	每天	30.00	8.00	散步	从不吸烟
144.00	46.50	76.00	22.42	每天	30.00	10.00	散步	从不吸烟
151.00	67.00	87.00	29.38	每天	30.00	5.00	散步	从不吸烟
159.00	67.00	87.00	26.50	每天	60.00	5.00	散步	从不吸烟
151.00	59.50	85.00	26.10	不锻炼				从不吸烟

145.50	44.50	76.00	21.02	不锻炼				从不吸烟
152.00	46.70	80.00	20.21	不锻炼				从不吸烟
160.00	73.00	91.00	28.52	每周一次!	30.00	3.00	散步	从不吸烟
154.00	57.00	86.00	24.03	不锻炼				从不吸烟
153.00	65.00	89.00	27.77	不锻炼				从不吸烟
153.00	69.00	91.00	29.48	每天	30.00	3.00	散步	从不吸烟
158.50	65.90	90.00	26.23	每天	30.00	5.00	散步	从不吸烟
149.00	40.20	68.00	18.11	每天	60.00	3.00	散步	从不吸烟
158.00	54.00	85.00	21.63	每天	30.00	3.00	散步	从不吸烟
146.00	51.40	83.00	24.11	每天	30.00	1.00	散步	从不吸烟
157.00	43.50	61.00	17.65	每天	60.00	3.00	散步	从不吸烟
156.00	54.50	78.00	22.39	每天	30.00	6.00	散步	从不吸烟
142.00	50.00	82.00	24.80	不锻炼				从不吸烟
147.00	59.00	86.00	27.30	每天	60.00	1.00	保健操	从不吸烟
155.00	66.30	88.00	27.60	每天	50.00	10.00	散步	从不吸烟
138.00	46.00	84.00	24.15	每天	30.00	3.00	散步	从不吸烟
146.00	45.00	84.00	21.11	每天	60.00	3.00	散步	从不吸烟
149.00	54.00	82.00	24.32	不锻炼				从不吸烟
150.00	50.00	77.00	22.22	每天	40.00	2.00	散步	从不吸烟
150.50	52.00	91.00	22.96	每天	30.00	2.00	散步	从不吸烟
155.00	59.00	98.00	24.56	不锻炼				从不吸烟
147.00	59.00	79.00	27.30	每天	30.00	1.00	散步	从不吸烟
147.00	57.00	86.00	26.38	每天	60.00	6.00	散步	从不吸烟
157.00	48.00	76.00	19.47	不锻炼				从不吸烟
152.00	54.00	82.00	23.37	不锻炼				从不吸烟
146.00	61.00	95.00	28.62	每天	30.00	10.00	散步	从不吸烟
144.00	56.00	78.00	27.01	每天	30.00	1.00	散步	从不吸烟
147.00	55.00	88.00	25.45	每天	60.00	5.00	散步	从不吸烟
147.00	58.00	88.00	26.84	每天	60.00	3.00	做操	从不吸烟
154.50	60.40	83.00	25.30	每天	30.00	5.00	散步	从不吸烟
152.00	58.50	87.00	25.32	每天	30.00	15.00	散步	从不吸烟
141.00	38.80	73.00	19.52	每天	60.00	2.00	散步	从不吸烟
163.00	65.00	91.00	24.46	每天	20.00	6.00	散步	从不吸烟
150.00	52.50	87.00	23.33	不锻炼				从不吸烟
151.00	59.00	86.00	25.88	不锻炼				从不吸烟
157.00	66.00	87.00	26.78	不锻炼				从不吸烟
147.00	74.50	112.00	34.48	不锻炼				从不吸烟
154.00	65.00	94.00	27.41	每天	60.00	2.00	散步	从不吸烟
137.00	44.50	75.00	23.71	每天	30.00	2.00	散步	从不吸烟
140.00	44.00	76.00	22.45	每天	120.00	3.00	散步	从不吸烟
155.00	52.50	86.00	21.85	每周一次!	20.00	1.00	散步	从不吸烟
149.00	63.00	90.00	28.38	每周一次!	30.00	3.00	散步	从不吸烟
154.00	62.00	92.00	26.14	每天	60.00	2.00	散步	从不吸烟
150.00	67.90	93.00	30.18	每天	60.00	12.00	散步	从不吸烟
148.50	65.00	96.00	29.48	不锻炼				从不吸烟
153.00	61.00	88.00	26.06	不锻炼				从不吸烟
140.00	60.00	94.00	30.61	每天	30.00	1.00	散步	从不吸烟
162.00	51.00	69.00	19.43	每天	30.00	2.00	做操	从不吸烟
147.00	62.50	95.00	28.92	每天	60.00	2.00	散步	从不吸烟
156.00	68.00	92.00	27.94	每天	30.00	1.00	散步	从不吸烟
150.00	59.80	86.00	26.58	不锻炼				从不吸烟

142.00	41.00	78.00	20.33	不锻炼				从不吸烟
147.00	51.00	83.00	23.60	每天	30.00	2.00	散步	从不吸烟
139.00	58.00	97.00	30.02	每天	30.00	11.00	散步	从不吸烟
158.00	59.00	83.00	23.63	不锻炼				从不吸烟
152.00	40.00	66.00	17.31	不锻炼				从不吸烟
143.00	59.50	95.00	29.10	每天	30.00	4.00	散步	从不吸烟
148.00	58.00	87.00	26.48	每天	60.00	15.00	散步	从不吸烟
154.00	57.50	89.00	24.25	每天	30.00	1.00	散步	从不吸烟
168.00	61.00	81.00	21.61	不锻炼				从不吸烟
139.00	50.00	94.00	25.88	不锻炼				从不吸烟
147.00	43.50	68.00	20.13	每周一次!	30.00	3.00	散步	从不吸烟
149.00	59.00	84.00	26.58	不锻炼				从不吸烟
144.00	54.00	98.00	26.04	每天	30.00	7.00	散步	从不吸烟
162.00	55.20	86.00	21.03	每天	30.00	10.00	散步	从不吸烟
152.00	61.00	90.00	26.40	每天	60.00	11.00	散步	从不吸烟
156.00	65.00	105.00	26.71	每天	30.00	10.00	散步	从不吸烟
142.00	50.50	80.00	25.04	每天	45.00	21.00	散步	从不吸烟
151.50	46.70	75.00	20.35	每天	30.00	3.00	散步	从不吸烟
151.00	65.00	92.00	28.51	每天	30.00	6.00	散步	从不吸烟
143.00	50.00	88.00	24.45	每天	40.00	5.00	散步	从不吸烟
153.00	64.50	91.00	27.55	每天	60.00	2.00	散步	从不吸烟
158.00	52.00	78.00	20.83	不锻炼				从不吸烟
142.00	35.00	58.00	17.36	不锻炼				从不吸烟
144.00	61.00	102.00	29.42	不锻炼				从不吸烟
160.00	56.60	84.00	22.11	每天	60.00	10.00	散步	从不吸烟
153.00	51.00	78.00	21.79	每天	30.00	4.00	散步	从不吸烟
148.00	51.00	87.00	23.28	不锻炼				从不吸烟
152.00	57.00	86.00	24.67	不锻炼				从不吸烟
161.00	48.00	70.00	18.52	不锻炼				从不吸烟
142.00	48.00	86.00	23.80	每天	48.00	10.00	散步	从不吸烟
156.50	57.20	98.00	23.35	每天	60.00	2.00	散步	从不吸烟
147.00	64.00	85.00	29.62	每天	30.00	10.00	散步	从不吸烟
148.00	56.00	88.00	25.57	不锻炼				从不吸烟
154.00	90.00	108.00	37.95	每天	60.00	10.00	散步	从不吸烟
145.00	45.10	80.00	21.45	不锻炼				从不吸烟
160.00	68.00	83.00	26.56	每天	30.00	7.00	散步	从不吸烟
140.50	47.50	89.00	24.06	不锻炼				从不吸烟
157.00	55.00	86.00	22.31	每天	60.00	3.00	广播操	从不吸烟
150.00	60.00	83.00	26.67	不锻炼				从不吸烟
153.00	52.00	84.00	22.21	每天	30.00	10.00	散步	从不吸烟
155.00	73.00	92.00	30.39	每天	60.00	3.00	散步	从不吸烟
155.00	64.00	90.00	26.64	每天	30.00	3.00	散步	从不吸烟
142.50	50.10	85.00	24.67	不锻炼				从不吸烟
154.50	59.00	85.00	24.72	每天	30.00	4.00	散步	从不吸烟
154.00	63.00	92.00	26.56	每周一次!	30.00	1.00	散步	从不吸烟
155.00	68.00	90.00	28.30	每天	30.00	3.00	散步	从不吸烟
162.00	58.00	84.00	22.10	每天	60.00	10.00	散步	从不吸烟
148.00	41.00	71.00	18.72	不锻炼				从不吸烟
149.00	59.00	87.00	26.58	不锻炼				从不吸烟
155.00	56.10	93.00	23.35	不锻炼				从不吸烟
145.00	56.00	90.00	26.63	每天	30.00	1.00	散步	从不吸烟

149.00	61.00	78.00	27.48	不锻炼				从不吸烟
151.00	64.00	90.00	28.07	每天	30.00	3.00	散步	从不吸烟
164.00	59.30	94.00	22.05	每天	60.00	10.00	散步	从不吸烟
161.00	54.00	82.00	20.83	每天	30.00	10.00	散步	从不吸烟
156.00	50.00	89.00	20.55	每天	40.00	3.00	散步	从不吸烟
155.00	58.00	87.00	24.14	每天	30.00	3.00	散步	从不吸烟
169.00	70.00	91.00	24.51	每周一次!	30.00	2.00	散步	从不吸烟
153.00	49.00	78.00	20.93	不锻炼				从不吸烟
149.00	50.85	80.00	22.90	每天	60.00	10.00	散步	从不吸烟
157.50	49.00	81.00	19.75	每天	60.00	7.00	散步	从不吸烟
156.00	63.50	87.00	26.09	不锻炼				从不吸烟
153.00	47.00	80.00	20.08	不锻炼				从不吸烟
150.00	54.00	76.00	24.00	不锻炼				从不吸烟
152.00	65.00	95.00	28.13	每周一次!	30.00	1.00	散步	从不吸烟
156.00	70.00	87.00	28.76	每天	30.00	10.00	散步	从不吸烟
153.50	57.00	78.00	24.19	每天	60.00	6.00	散步	从不吸烟
163.00	84.00	91.00	31.62	每天	60.00	15.00	散步	从不吸烟
151.00	50.50	78.00	22.15	每天	60.00	2.00	散步	从不吸烟
142.00	47.00	82.00	23.31	不锻炼				从不吸烟
152.00	47.00	78.00	20.34	每天	60.00	3.00	散步	从不吸烟
148.00	44.00	76.00	20.09	每天	60.00	6.00	散步	从不吸烟
150.00	48.00	89.00	21.33	每天	20.00	2.00	散步	从不吸烟
153.00	63.00	86.00	26.91	每天	20.00	5.00	散步	从不吸烟
156.00	73.10	99.00	30.04	每天	60.00	3.00	散步	从不吸烟
153.00	61.70	93.00	26.36	每天	60.00	6.00	散步	从不吸烟
149.00	50.80	78.00	22.88	每天	30.00	10.00	散步	从不吸烟
153.00	60.00	93.00	25.63	每天	20.00	4.00	散步	从不吸烟
149.00	54.00	89.00	24.32	不锻炼				从不吸烟
148.00	52.00	94.00	23.74	不锻炼				从不吸烟
139.00	55.00	86.00	28.47	每周一次!	30.00	1.00	散步	从不吸烟
149.00	65.00	98.00	29.28	不锻炼				从不吸烟
149.00	68.00	93.00	30.63	每天	60.00	6.00	散步	从不吸烟
153.50	54.90	86.00	23.30	每天	60.00	10.00	散步	从不吸烟
150.00	58.00	87.00	25.78	不锻炼				从不吸烟
160.00	69.50	95.00	27.15	每天	30.00	1.00	散步	从不吸烟
154.00	60.00	93.00	25.30	不锻炼				从不吸烟
148.00	59.00	89.00	26.94	每天	60.00	2.00	散步	从不吸烟
151.00	61.00	89.00	26.75	每天	60.00	6.00	散步	从不吸烟
143.00	59.00	91.00	28.85	每周一次!	30.00	1.00	散步	从不吸烟
150.00	47.00	84.00	20.89	每天	30.00	1.00	散步	从不吸烟
142.00	53.00	82.00	26.28	每天	60.00	1.00	散步	从不吸烟
146.00	58.00	81.00	27.21	每天	30.00	1.00	散步	从不吸烟
163.00	73.00	98.00	27.48	每天	60.00	2.00	散步	从不吸烟
167.00	76.00	98.00	27.25	每天	30.00	2.00	散步	从不吸烟
147.00	47.50	75.00	21.98	每天	30.00	2.00	散步	从不吸烟
154.00	52.00	85.00	21.93	不锻炼				从不吸烟
144.00	46.00	91.00	22.18	每天	30.00	2.00	散步	从不吸烟
146.00	57.00	88.00	26.74	每天	15.00	3.00	散步	从不吸烟
151.00	51.00	81.00	22.37	不锻炼				从不吸烟
151.00	59.00	80.00	25.88	不锻炼				从不吸烟
156.50	76.00	102.00	31.03	每天	60.00	4.00	散步	从不吸烟

168.50	73.30	102.00	25.82	不锻炼				从不吸烟
147.00	56.50	94.00	26.15	每天	60.00	4.00	散步	从不吸烟
152.00	67.00	90.00	29.00	每周一次!	30.00	1.00	散步	从不吸烟
151.00	73.00	96.00	32.02	每天	30.00	10.00	散步	从不吸烟
146.00	44.00	69.00	20.64	每天	30.00	12.00	散步	从不吸烟
151.00	45.40	73.00	19.91	每天	30.00	2.00	散步	从不吸烟
161.00	71.00	96.00	27.39	每天	60.00	4.00	散步	从不吸烟
149.00	48.10	78.00	21.67	不锻炼				从不吸烟
158.00	60.00	87.00	24.03	每天	30.00	1.00	散步	从不吸烟
151.00	47.50	77.00	20.83	每天	60.00	12.00	散步	从不吸烟
146.00	47.50	92.00	22.28	每天	30.00	5.00	散步	从不吸烟
147.00	54.00	86.00	24.99	每天	30.00	10.00	散步	从不吸烟
151.00	53.00	87.00	23.24	不锻炼				从不吸烟
142.00	52.30	90.00	25.94	不锻炼				从不吸烟
154.50	60.00	87.00	25.14	每天	30.00	10.00	散步	从不吸烟
141.00	50.50	88.00	25.40	每天	30.00	1.00	散步	从不吸烟
152.00	51.80	81.00	22.42	每天	30.00	15.00	散步	从不吸烟
153.00	67.10	94.00	28.66	不锻炼				从不吸烟
150.00	61.00	95.00	27.11	不锻炼				从不吸烟
161.00	53.00	83.00	20.45	每天	60.00	4.00	散步	从不吸烟
152.00	51.00	75.00	22.07	每天	30.00	1.00	散步	从不吸烟
145.00	53.00	87.00	25.21	不锻炼				从不吸烟
157.50	58.00	86.00	23.38	每天	30.00	10.00	散步	从不吸烟
154.00	53.50	87.00	22.56	不锻炼				从不吸烟
148.00	42.00	80.00	19.17	不锻炼				从不吸烟
155.00	76.00	107.00	31.63	每天	30.00	2.00	散步	从不吸烟
150.50	60.60	99.00	26.75	每天	60.00	5.00	散步	从不吸烟
143.00	52.00	91.00	25.43	每天	60.00	7.00	散步	从不吸烟
148.00	50.00	79.00	22.83	每周一次!	30.00	2.00	散步	从不吸烟
147.00	52.00	80.00	24.06	每天	30.00	10.00	散步	从不吸烟
152.00	52.00	75.00	22.51	每天	30.00	10.00	散步	从不吸烟
161.00	58.00	83.00	22.38	每天	40.00	6.00	散步	从不吸烟
153.00	51.00	84.00	21.79	每天	20.00	20.00	散步，打;	从不吸烟
149.00	59.00	82.00	26.58	每天	60.00	16.00	散步	从不吸烟
155.00	75.00	101.00	31.22	每天	30.00	3.00	散步	从不吸烟
141.00	50.00	88.00	25.15	每天	30.00	2.00	散步	从不吸烟
148.50	55.00	75.00	24.94	每天	60.00	4.00	散步	从不吸烟
155.00	61.50	89.00	25.60	不锻炼				从不吸烟
144.00	42.00	67.00	20.25	每天	60.00	4.00	散步	从不吸烟
150.00	55.50	88.00	24.67	每天	30.00	4.00	散步	从不吸烟
153.00	55.00	87.00	23.50	每天	30.00	12.00	散步	从不吸烟
147.00	60.00	90.00	27.77	每天	30.00	5.00	散步	从不吸烟
149.50	41.00	62.50	18.34	不锻炼				从不吸烟
136.00	30.00	66.00	16.22	每周一次!	10.00	1.00	散步	从不吸烟
160.00	73.00	96.00	28.52	每天	60.00	10.00	散步	从不吸烟
157.00	76.00	102.00	30.83	不锻炼				从不吸烟
157.00	64.10	97.00	26.01	每天	30.00	1.00	散步	从不吸烟
157.00	63.00	89.00	25.56	每天	30.00	5.00	散步	从不吸烟
147.00	69.00	102.00	31.93	不锻炼				从不吸烟
143.00	47.00	86.00	22.98	每天	30.00	11.00	散步	从不吸烟
155.00	52.50	78.00	21.85	每天	60.00	12.00	散步	从不吸烟

154.00	57.00	86.00	24.03	每天	60.00	3.00	散步	从不吸烟
147.50	58.00	93.00	26.66	每天	30.00	2.00	散步	从不吸烟
147.00	51.50	89.00	23.83	每天	60.00	1.00	散步	从不吸烟
141.00	42.90	83.00	21.58	每天	60.00	20.00	散步	从不吸烟
137.00	47.00	92.00	25.04	每天	30.00	4.00	散步	从不吸烟
147.00	66.00	93.00	30.54	每天	30.00	12.00	散步	从不吸烟
152.00	63.00	91.00	27.27	每天	30.00	3.00	散步	从不吸烟
145.00	53.00	82.00	25.21	每天	30.00	10.00	散步	从不吸烟
146.00	63.00	94.00	29.56	每天	60.00	6.00	散步	从不吸烟
153.00	55.50	83.00	23.71	不锻炼				从不吸烟
148.00	47.00	71.00	21.46	不锻炼				从不吸烟
148.00	54.00	94.00	24.65	每天	60.00	1.00	散步	从不吸烟
150.00	53.00	92.00	23.56	每天	30.00	3.00	散步	从不吸烟
165.00	77.00	98.00	28.28	每天	30.00	3.00	散步	从不吸烟
156.00	50.00	74.00	20.55	不锻炼				从不吸烟
152.00	68.00	103.00	29.43	每天	30.00	11.00	散步	从不吸烟
143.00	54.00	88.00	26.41	每天	60.00	1.00	散步	从不吸烟
157.10	71.20	96.00	28.85	不锻炼				从不吸烟
151.00	59.00	98.00	25.88	每天	60.00	1.00	做操	从不吸烟
149.00	64.00	91.00	28.83	每天	60.00	10.00	散步	从不吸烟
147.00	41.00	62.00	18.97	每天	120.00	2.00	散步	从不吸烟
163.00	65.00	85.00	24.46	每天	30.00	2.00	散步	从不吸烟
153.00	58.00	71.00	24.78	每天	60.00	1.00	散 步	从不吸烟
147.00	46.50	78.00	21.52	每天	60.00	10.00	散步	从不吸烟
156.00	59.00	85.00	24.24	每天	60.00	11.00	跳舞、打	从不吸烟
146.00	50.00	86.00	23.46	每天	60.00	4.00	散步	从不吸烟
150.00	55.50	82.00	24.67	不锻炼				从不吸烟
139.00	43.00	69.00	22.26	不锻炼				从不吸烟
154.00	52.00	82.00	21.93	每天	30.00	2.00	散步	从不吸烟
152.00	63.80	90.00	27.61	每天	30.00	6.00	散步	从不吸烟
153.00	53.40	80.00	22.81	不锻炼				从不吸烟
156.00	61.00	87.00	25.07	每天	30.00	2.00	散步	从不吸烟
158.00	62.00	90.00	24.84	每周一次	20.00	1.00	散步	从不吸烟
141.50	42.90	87.00	21.43	每天	30.00	5.00	散步	从不吸烟
153.00	71.50	98.00	30.54	每天	30.00	1.00	散步	从不吸烟
142.00	40.00	68.00	19.84	每天	30.00	5.00	散步	从不吸烟
147.00	52.00	72.00	24.06	每天	30.00	3.00	散步	从不吸烟
164.00	64.00	85.00	23.80	每天	30.00	2.00	散步	从不吸烟
151.00	65.00	92.00	28.51	不锻炼				从不吸烟
149.00	41.50	66.00	18.69	每天	30.00	7.00	散步	从不吸烟
155.00	51.00	90.00	21.23	偶尔	30.00	1.00	散步	从不吸烟
152.00	66.00	91.00	28.57	每天	30.00	5.00	散步	从不吸烟
146.00	49.90	86.00	23.41	每天	60.00	7.00	散步	从不吸烟
149.00	51.80	78.00	23.33	每天	60.00	4.00	散步	从不吸烟
144.00	56.00	78.00	27.01	每周一次	30.00	3.00	散步	从不吸烟
146.00	52.00	87.00	24.39	不锻炼				从不吸烟
145.00	45.00	87.00	21.40	不锻炼				从不吸烟
153.00	59.00	94.00	25.20	每周一次	30.00	1.00	散步	从不吸烟
148.00	44.50	74.00	20.32	每天	60.00	12.00	散步	从不吸烟
150.00	49.00	95.00	21.78	不锻炼				从不吸烟
158.00	52.00	80.00	20.83	每天	30.00	1.00	散步	从不吸烟

154.50	50.00	80.00	20.95	每天	60.00	9.00	散步	从不吸烟
144.00	62.00	92.00	29.90	每天	30.00	3.00	散步	从不吸烟
143.50	48.30	81.00	23.46	每天	30.00	5.00	散步	从不吸烟
154.00	56.20	88.00	23.70	每天	30.00	10.00	散步	从不吸烟
147.00	53.00	88.00	24.53	每天	30.00	11.00	散步	从不吸烟
151.00	48.10	82.00	21.10	不锻炼				从不吸烟
153.00	65.00	86.00	27.77	每天	60.00	11.00	散步	从不吸烟
156.00	58.00	87.00	23.83	每天	60.00	3.00	散步	从不吸烟
162.00	64.50	89.00	24.58	每周一次!	30.00	3.00	散步	从不吸烟
152.00	55.00	87.00	23.81	每天	30.00	1.00	散步	从不吸烟
147.00	45.00	77.00	20.82	每天	30.00	2.00	散步	从不吸烟
159.00	63.00	90.00	24.92	不锻炼				从不吸烟
152.50	75.00	100.00	32.25	每天	30.00	3.00	散步	从不吸烟
145.00	58.00	88.00	27.59	每天	20.00	3.00	散步	从不吸烟
154.00	59.00	83.00	24.88	不锻炼				从不吸烟
143.00	46.00	80.00	22.49	每天	30.00	3.00	散步	从不吸烟
141.00	39.00	58.00	19.62	每天	30.00	5.00	散步	从不吸烟
156.00	61.00	89.00	25.07	每天	60.00	2.00	跳舞	从不吸烟
143.00	38.00	66.00	18.58	每天	30.00	1.00	散步	从不吸烟
145.00	60.00	90.00	28.54	不锻炼				从不吸烟
160.00	68.00	93.00	26.56	每天	60.00	12.00	散步	从不吸烟
147.00	46.00	76.00	21.29	每天	30.00	10.00	散步	从不吸烟
144.50	40.50	66.00	19.40	不锻炼				从不吸烟
147.00	47.50	81.00	21.98	每天	30.00	5.00	散步	从不吸烟
141.00	52.00	83.00	26.16	不锻炼				从不吸烟
153.00	61.00	86.00	26.06	每天	60.00	3.00	散步	从不吸烟
144.00	56.00	91.00	27.01	每天	60.00	17.00	散步	从不吸烟
154.00	70.00	109.00	29.52	每天	30.00	6.00	散步	从不吸烟
158.00	58.00	88.00	23.23	每天	30.00	5.00	散步	从不吸烟
160.50	62.40	88.00	24.22	每天	30.00	2.00	散步	从不吸烟
138.00	45.00	71.00	23.63	每周一次!	30.00	1.00	散步	从不吸烟
154.00	49.00	81.00	20.66	每天	30.00	2.00	散步	从不吸烟
146.00	44.00	86.00	20.64	每天	30.00	4.00	散步	从不吸烟
154.00	47.00	70.00	19.82	每天	30.00	3.00	散步	从不吸烟
156.00	63.00	83.00	25.89	每天	30.00	10.00	散步	从不吸烟
154.00	50.00	76.00	21.08	不锻炼				从不吸烟
167.00	78.00	92.00	27.97	每天	30.00	4.00	散步	从不吸烟
152.00	62.00	92.00	26.84	不锻炼				从不吸烟
140.00	58.50	87.00	29.85	每天	60.00	6.00	散步	从不吸烟
165.00	57.00	76.00	20.94	每周一次!	30.00	1.00	散步	从不吸烟
149.00	55.40	91.00	24.95	不锻炼				从不吸烟
148.00	55.00	80.00	25.11	每天	30.00	3.00	散步	从不吸烟
154.00	64.00	92.00	26.99	每天	30.00	4.00	散步	从不吸烟
152.50	51.00	76.00	21.93	不锻炼				从不吸烟
143.00	52.50	93.00	25.67	每天	30.00	2.00	散步	从不吸烟
162.00	72.00	90.00	27.43	每天	30.00	10.00	散步	从不吸烟
152.00	60.50	97.00	26.19	每天	60.00	3.00	散步	从不吸烟
154.00	64.00	87.00	26.99	每天	30.00	1.00	散步	从不吸烟
158.00	47.00	73.00	18.83	每天	60.00	5.00	散步	从不吸烟
147.50	50.50	85.00	23.21	每天	30.00	2.00	散步	从不吸烟
159.00	63.00	85.00	24.92	每天	30.00	10.00	散步	从不吸烟

146.00	48.00	80.00	22.52	每周一次L	20.00	1.00	散步	从不吸烟
156.00	66.00	96.00	27.12	每天	60.00	2.00	跳舞	从不吸烟
155.00	60.00	92.00	24.97	每天	30.00	11.00	散步	从不吸烟
155.00	62.00	99.00	25.81	每天	30.00	2.00	散步	从不吸烟
155.00	65.50	92.00	27.26	不锻炼				从不吸烟
150.00	41.00	79.00	18.22	不锻炼				从不吸烟
149.00	56.00	76.00	25.22	每天	30.00	10.00	散步	从不吸烟
156.00	51.80	75.00	21.29	每周一次L	30.00	2.00	散步	从不吸烟
157.00	61.30	81.00	24.87	不锻炼				从不吸烟
152.00	42.00	70.00	18.18	每周一次L	30.00	3.00	散步	从不吸烟
158.00	61.80	90.00	24.76	每天	60.00	7.00	散步	从不吸烟
153.00	60.00	89.00	25.63	每天	30.00	3.00	散步	从不吸烟
152.00	68.00	95.00	29.43	每周一次L	20.00	2.00	散步	从不吸烟
156.00	65.00	82.00	26.71	每周一次L	30.00	1.00	散步	从不吸烟
152.00	50.00	68.00	21.64	每天	30.00	1.00	散步	从不吸烟
156.00	63.00	90.00	25.89	每天	60.00	11.00	散步	从不吸烟
151.00	47.00	88.00	20.61	不锻炼				从不吸烟
146.00	58.00	96.00	27.21	不锻炼				从不吸烟
155.00	80.00	106.00	33.30	每天	30.00	2.00	散步	从不吸烟
148.00	54.00	75.00	24.65	每天	30.00	8.00	做操	从不吸烟
145.00	54.00	87.00	25.68	不锻炼				从不吸烟
147.00	68.50	106.00	31.70	每天	60.00	6.00	散步	从不吸烟
153.00	62.00	89.00	26.49	每天	120.00	10.00	打太极	从不吸烟
143.00	49.00	86.00	23.96	每周一次L	30.00	1.00	散步	从不吸烟
156.00	52.40	78.00	21.53	每天	30.00	11.00	散步	从不吸烟
156.00	62.00	96.00	25.48	每天	30.00	6.00	散步	从不吸烟
156.00	56.00	79.00	23.01	不锻炼				从不吸烟
147.00	52.00	85.00	24.06	不锻炼				从不吸烟
151.00	59.00	92.00	25.88	不锻炼				从不吸烟
157.00	70.00	95.00	28.40	每天	20.00	7.00	散步	从不吸烟
155.00	54.40	90.00	22.64	每天	30.00	5.00	散步	从不吸烟
153.00	53.00	90.00	22.64	不锻炼				从不吸烟
153.00	54.00	86.00	23.07	每天	30.00	20.00	散步	从不吸烟
148.50	64.90	100.00	29.43	每天	30.00	11.00	散步	从不吸烟
152.00	58.00	91.00	25.10	每天	60.00	6.00	散步	从不吸烟
155.00	65.90	92.00	27.43	每天	30.00	10.00	散步	从不吸烟
147.00	52.00	86.00	24.06	每天	30.00	1.00	散步	从不吸烟
134.00	41.00	76.00	22.83	不锻炼				从不吸烟
152.00	47.00	70.00	20.34	不锻炼				从不吸烟
155.00	64.80	86.00	26.97	每天	40.00	3.00	散步	从不吸烟
153.00	57.00	88.00	24.35	每周一次L	20.00	3.00	散步	从不吸烟
148.00	48.50	76.00	22.14	每天	30.00	7.00	散步	从不吸烟
150.00	67.00	96.00	29.78	不锻炼				从不吸烟
158.00	56.00	87.00	22.43	不锻炼				从不吸烟
154.00	50.50	79.00	21.29	不锻炼				从不吸烟
145.00	52.00	91.00	24.73	每周一次L	60.00	3.00	散步	从不吸烟
152.00	61.00	94.00	26.40	每天	30.00	2.00	散步	从不吸烟
147.00	50.00	83.00	23.14	每天	30.00	10.00	散步	从不吸烟
161.00	71.00	102.00	27.39	每天	30.00	1.00	早操	从不吸烟
145.00	37.00	67.00	17.60	不锻炼				从不吸烟
137.00	39.00	77.00	20.78	每天	30.00	12.00	散步	从不吸烟

149.00	66.00	92.00	29.73	不锻炼				从不吸烟
153.00	61.00	91.00	26.06	每天	30.00	1.00	散步、做抖	从不吸烟
150.00	51.00	73.00	22.67	每周一次以	30.00	2.00	散步	从不吸烟
146.00	57.50	87.00	26.98	每天	20.00	1.00	散步	从不吸烟
155.00	57.30	84.00	23.85	每天	60.00	8.00	散步	从不吸烟
148.00	52.00	90.00	23.74	每天	60.00	5.00	散步	从不吸烟
153.00	48.00	73.00	20.50	每天	60.00	5.00	散步	从不吸烟
157.00	71.20	104.00	28.89	每天	20.00	1.00	散步	从不吸烟
153.00	58.00	78.00	24.78	每天	30.00	1.00	散步	从不吸烟
149.50	38.00	68.50	17.00	不锻炼				从不吸烟
150.00	62.00	90.00	27.56	每天	40.00	3.00	散步	从不吸烟
149.00	53.00	76.00	23.87	不锻炼				从不吸烟
154.00	63.00	92.00	26.56	不锻炼				从不吸烟
148.00	53.60	83.00	24.47	不锻炼				从不吸烟
153.00	49.30	79.00	21.06	每天	60.00	11.00	散步	从不吸烟
145.00	64.00	112.00	30.44	每周一次以	30.00	1.00	散步	从不吸烟
150.50	50.00	85.00	22.07	不锻炼				从不吸烟
150.00	52.50	86.00	23.33	每天	30.00	9.00	散步	从不吸烟
150.00	60.00	93.00	26.67	每天	60.00	3.00	散步	从不吸烟
154.00	44.00	77.00	18.55	每天	30.00	2.00	散步	从不吸烟
150.00	54.00	82.00	24.00	每天	30.00	2.00	散步	从不吸烟
155.00	41.00	69.00	17.07	每天	30.00	2.00	散步	从不吸烟
154.00	62.00	87.00	26.14	每天	60.00	15.00	散步	从不吸烟
136.00	60.20	95.00	32.55	每天	60.00	3.00	散步	从不吸烟
146.50	57.50	82.00	26.79	不锻炼				从不吸烟
150.00	53.00	71.00	23.56	每天	60.00	13.00	散步	从不吸烟
153.00	58.00	86.00	24.78	每天	60.00	4.00	散步	从不吸烟
148.00	40.00	78.00	18.26	每天	60.00	5.00	散步	从不吸烟
151.00	41.00	65.00	17.98	不锻炼				从不吸烟
162.00	70.00	98.00	26.67	每天	30.00	1.00	散步	从不吸烟
147.00	50.00	82.00	23.14	不锻炼				从不吸烟
146.00	56.00	87.00	26.27	每天	30.00	2.00	散步	从不吸烟
168.00	61.90	90.00	21.93	不锻炼				从不吸烟
146.00	55.00	86.00	25.80	每天	30.00	1.00	散步	从不吸烟
145.00	52.00	90.00	24.73	每天	30.00	10.00	散步	从不吸烟
152.50	51.70	82.00	22.23	每天	60.00	10.00	散步	从不吸烟
149.00	42.00	87.00	18.92	每天	20.00	4.00	散步	从不吸烟
154.50	64.50	88.00	27.02	每天	20.00	2.00	散步	从不吸烟
159.00	56.00	73.00	22.15	每天	30.00	2.00	散步	从不吸烟
153.00	68.00	88.00	29.05	每天	60.00	3.00	散步	从不吸烟
150.00	60.00	88.00	26.67	每天	30.00	4.00	散步	从不吸烟
153.00	47.00	76.00	20.08	不锻炼				从不吸烟
156.00	72.00	96.00	29.59	不锻炼				从不吸烟
151.00	53.00	83.00	23.24	每天	60.00	1.00	散步	从不吸烟
154.00	55.00	84.00	23.19	每天	60.00	4.00	散步	从不吸烟
139.00	38.00	70.00	19.67	每天	30.00	5.00	散步	从不吸烟
151.00	59.00	87.00	25.88	每天	30.00	10.00	散步	从不吸烟
154.00	50.00	80.00	21.08	每天	30.00	4.00	散步	从不吸烟
151.00	54.30	92.00	23.81	每天	60.00	5.00	散步	从不吸烟
147.00	48.40	76.00	22.40	每天	30.00	3.00	散步	从不吸烟
137.00	34.80	60.00	18.54	每天	30.00	10.00	散步	从不吸烟

158.00	63.00	83.00	25.24	每天	30.00	2.00	散步	从不吸烟
162.00	64.00	93.00	24.39	每天	30.00	1.00	散步	从不吸烟
152.00	61.00	87.00	26.40	每天	30.00	7.00	散步	从不吸烟
148.00	42.00	70.00	19.17	每天	15.00	1.00	散步	从不吸烟
155.00	44.00	76.00	18.31	每周一次l	30.00	1.00	散步	从不吸烟
148.00	51.00	83.00	23.28	每天	60.00	6.00	散步	从不吸烟
152.00	67.00	94.00	29.00	不锻炼				从不吸烟
144.00	42.40	86.00	20.45	每天	60.00	7.00	散步	从不吸烟
160.00	82.00	102.00	32.03	每天	30.00	3.00	散步	从不吸烟
159.50	68.00	97.00	26.73	每天	30.00	1.00	散步	从不吸烟
159.50	68.00	90.00	26.73	不锻炼				从不吸烟
149.00	46.00	73.00	20.72	每天	120.00	6.00	散步、跳纟	从不吸烟
155.00	56.30	85.00	23.43	不锻炼				从不吸烟
151.00	44.00	73.00	19.30	每天	20.00	14.00	散步	从不吸烟
153.00	60.00	89.00	25.63	每天	30.00	4.00	散步	从不吸烟
154.00	65.00	89.00	27.41	每天	30.00	4.00	散步	从不吸烟
153.00	86.50	104.00	36.95	不锻炼				从不吸烟
158.00	61.00	87.00	24.44	每天	30.00	9.00	散步	从不吸烟
138.00	54.00	92.00	28.36	不锻炼				从不吸烟
145.00	57.00	88.00	27.11	每天	60.00	7.00	散步	从不吸烟
149.00	59.00	94.00	26.58	每天	60.00	3.00	散步	从不吸烟
137.00	30.00	76.00	15.98	每天	30.00	4.00	散步	从不吸烟
164.00	58.80	82.00	21.86	每天	30.00	10.00	散步	从不吸烟
153.00	52.00	97.00	22.21	每天	60.00	18.00	散步	吸烟
145.50	50.00	83.00	23.62	不锻炼				从不吸烟
153.00	54.00	80.00	23.07	不锻炼				从不吸烟
141.00	45.60	80.00	22.94	不锻炼				从不吸烟
143.00	52.00	95.00	25.43	每天	60.00	5.00	散步	从不吸烟
148.00	42.00	70.00	19.17	每天	30.00	1.00	散步	从不吸烟
144.50	50.70	76.00	24.28	每周一次l	30.00	1.00	散步	从不吸烟
159.00	52.80	83.00	20.89	每天	30.00	10.00	散步	从不吸烟
150.50	46.30	73.00	20.44	每天	40.00	10.00	散步	从不吸烟
156.50	54.45	87.00	22.23	每天	30.00	4.00	散步	从不吸烟
139.00	53.00	82.00	27.43	不锻炼				从不吸烟
158.00	65.50	89.00	26.24	不锻炼				从不吸烟
138.50	50.00	84.00	26.07	每天	60.00	4.00	散步	从不吸烟
157.00	64.00	84.00	25.96	不锻炼				从不吸烟
154.00	57.30	85.00	24.16	每天	30.00	8.00	散步	从不吸烟
156.00	60.00	87.00	24.65	不锻炼				从不吸烟
147.00	55.00	90.00	25.45	每天	30.00	6.00	散步	从不吸烟
144.00	53.00	83.00	25.56	每天	30.00	2.00	散步	从不吸烟
145.00	47.00	77.00	22.35	每天	60.00	6.00	散步	从不吸烟
158.00	66.00	91.00	26.44	每天	60.00	9.00	散步	从不吸烟
147.00	48.50	75.50	22.44	每天	30.00	2.00	散步	从不吸烟
151.00	59.00	85.00	25.88	不锻炼				从不吸烟
148.50	50.50	72.50	22.90	每天	30.00	1.00	散步	从不吸烟
165.00	73.70	96.00	27.07	每天	60.00		散步、跳纟	从不吸烟
160.00	59.50	91.00	23.24	不锻炼				从不吸烟
151.00	59.00	90.00	25.88	每天	60.00	5.00	散步	从不吸烟
159.50	63.00	90.00	24.76	每天	30.00	4.00	散步	从不吸烟
147.50	51.50	87.00	23.67	不锻炼				从不吸烟

150.00	50.00	85.00	22.22	每周一次	30.00	1.00	散步	从不吸烟
151.50	50.10	79.00	21.83	每天	30.00	5.00	散步	从不吸烟
150.00	39.80	80.00	17.69	不锻炼				从不吸烟
131.00	36.00	80.00	20.98	每天	30.00	17.00	散步	吸烟
143.00	58.50	92.00	28.61	每天	30.00	2.00	散步	从不吸烟
143.00	66.00	99.00	32.28	不锻炼				从不吸烟
156.00	49.00	81.00	20.13	不锻炼				从不吸烟
157.00	52.00	83.00	21.10	每天	60.00	5.00	散步	从不吸烟
167.00	67.00	86.00	24.02	不锻炼				从不吸烟
141.00	40.50	64.50	20.37	每天	30.00	1.00	散步	从不吸烟
141.00	55.50	90.00	27.92	每天	30.00	3.00	散步	从不吸烟
151.00	59.50	90.00	26.10	每天	40.00	1.00	散步	从不吸烟
148.00	46.00	72.00	21.00	每周一次	30.00	1.00	散步	从不吸烟
146.00	60.00	92.00	28.15	每天	60.00	2.00	散步	从不吸烟
152.00	63.00	102.00	27.27	每天	30.00	1.00	散步	从不吸烟
157.00	80.00	105.00	32.46	每天	20.00	2.00	散步	从不吸烟
140.00	51.00	85.00	26.02	每天	30.00	3.00	散步	从不吸烟
147.00	53.00	80.00	24.53	每天	60.00	11.00	跳舞	从不吸烟
148.00	62.80	100.00	28.67	每天	60.00	1.00	散步	从不吸烟
150.50	67.00	102.00	29.58	每天	60.00		骑车	从不吸烟
148.00	50.00	79.00	22.83	每天	30.00	10.00	跳舞	从不吸烟
153.50	45.00	73.00	19.10	不锻炼				从不吸烟
155.00	52.00	74.00	21.64	每天	20.00	2.00	散步	从不吸烟
158.00	74.00	97.00	29.64	每天	60.00	11.00	散步	从不吸烟
157.00	75.00	100.00	30.43	每天	60.00	4.00	散步	从不吸烟
147.00	55.00	88.00	25.45	每天	30.00	3.00	散步	从不吸烟
142.00	49.50	85.00	24.55	每天	30.00	8.00	散步	从不吸烟
143.50	59.20	93.00	28.75	每天	30.00	8.00	散步	从不吸烟
141.00	51.00	83.00	25.65	不锻炼				从不吸烟
143.00	48.00	86.00	23.47	每天	60.00	11.00	散步	从不吸烟
142.00	42.00	76.00	20.83	每天	60.00	7.00	散步	从不吸烟
147.00	60.30	87.00	27.91	不锻炼				从不吸烟
132.00	36.00	78.00	20.66	每天	30.00	8.00	散步	从不吸烟
155.00	57.00	91.00	23.73	每天	60.00	4.00	散步	从不吸烟
153.50	58.20	90.00	24.70	每天	30.00	3.00	散步	从不吸烟
153.00	52.00	75.00	22.21	每天	30.00	7.00	散步	从不吸烟
148.00	44.00	71.00	20.09	每天	30.00	2.00	做操	从不吸烟
153.00	58.00	85.00	24.78	每周一次	30.00	1.00	散步	从不吸烟
144.00	50.00	82.00	24.11	每周一次	30.00	1.00	散步	从不吸烟
143.00	45.00	76.00	22.01	不锻炼				从不吸烟
150.00	48.00	80.00	21.33	不锻炼				从不吸烟
150.00	59.00	88.00	26.22	每天	60.00	11.00	散步	从不吸烟
155.00	56.00	87.00	23.31	每天	30.00	7.00	散步	从不吸烟
151.00	52.30	83.00	22.94	每周一次	30.00	11.00	散步	从不吸烟
152.00	59.00	81.00	25.54	每天	60.00	8.00	散步	从不吸烟
160.00	56.00	79.00	21.87	每天	30.00	2.00	散步	从不吸烟
144.00	55.00	91.00	26.52	每天	60.00	3.00	散步	从不吸烟
148.00	45.00	82.00	20.54	不锻炼				从不吸烟
152.00	93.00	117.00	40.25	每天	30.00	6.00	散步	从不吸烟
160.00	67.00	97.00	26.17	每天	30.00	11.00	散步	从不吸烟
155.00	64.00	93.00	26.64	不锻炼				从不吸烟

147.00	42.00	73.00	19.44	每天	30.00	15.00	散步	吸烟
149.00	47.00	83.00	21.17	每天	60.00	12.00	散步	从不吸烟
153.50	60.00	91.00	25.46	不锻炼				从不吸烟
150.00	49.00	80.00	21.78	每天	30.00	1.00	散步	从不吸烟
140.00	45.00	93.00	22.96	每天	30.00	2.00	散步	从不吸烟
154.00	55.90	86.00	23.57	每周一次!	15.00	1.00	散步	从不吸烟
150.00	53.50	90.00	23.78	每天	30.00	4.00	散步	从不吸烟
154.00	63.00	94.00	26.56	每天	40.00	3.00	散步	从不吸烟
155.00	61.70	81.00	25.68	每天	60.00	10.00	散步	从不吸烟
144.00	58.00	87.00	27.97	每天	40.00	6.00	散步	从不吸烟
146.00	53.00	90.00	24.86	每天	60.00	4.00	散步	从不吸烟
154.00	52.00	86.00	21.93	每天	30.00	1.00	散步	从不吸烟
156.00	63.50	90.00	26.09	每天	60.00	3.00	散步	从不吸烟
154.00	53.00	79.00	22.35	每天	30.00	7.00	散步	从不吸烟
147.00	56.80	91.00	26.29	每周一次!	10.00	11.00	散步	从不吸烟
146.00	65.00	94.00	30.49	每天	30.00	2.00	散步	从不吸烟
154.00	55.50	78.00	23.40	不锻炼				从不吸烟
142.00	41.00	77.00	20.33	每周一次!	30.00	7.00	做操	从不吸烟
157.50	71.20	103.00	28.70	每天	50.00	1.00	散步	从不吸烟
157.00	55.00	83.00	22.31	每天	60.00	11.00	散步	从不吸烟
147.00	57.00	85.00	26.38	不锻炼				从不吸烟
141.00	39.50	70.00	19.87	不锻炼				从不吸烟
146.00	54.00	89.00	25.33	不锻炼				从不吸烟
148.00	42.00	74.00	19.17	每天	30.00	5.00	散步	从不吸烟
150.00	57.00	92.00	25.33	不锻炼				从不吸烟
156.00	59.00	94.00	24.24	每天	60.00	4.00	散步	从不吸烟
142.00	58.00	94.00	28.76	不锻炼				从不吸烟
164.00	60.00	92.00	22.31	不锻炼				从不吸烟
160.50	61.80	89.00	23.99	不锻炼				从不吸烟
154.00	55.00	82.00	23.19	每天	30.00	3.00	散步	从不吸烟
156.00	53.50	85.00	21.98	每天	30.00	2.00	散步	从不吸烟
156.00	66.50	94.00	27.33	不锻炼				从不吸烟
142.50	46.00	73.00	22.65	不锻炼				从不吸烟
146.50	55.50	85.00	25.86	每天	60.00	2.00	散步	从不吸烟
153.00	54.20	78.00	23.15	每天	60.00	5.00	跳舞	从不吸烟
150.00	62.50	90.00	27.78	不锻炼				从不吸烟
148.50	48.50	72.00	21.99	每天	30.00	10.00	散步	从不吸烟
154.00	74.00	90.00	31.20	不锻炼				从不吸烟
146.00	61.00	88.00	28.62	每天	30.00	4.00	散步	从不吸烟
145.00	53.30	84.00	25.35	不锻炼				从不吸烟
163.00	66.00	92.00	24.84	不锻炼				从不吸烟
158.50	58.70	84.00	23.37	不锻炼				从不吸烟
138.00	50.00	83.00	26.25	每天	60.00	1.00	散步	从不吸烟
144.00	55.00	92.00	26.52	每天	30.00	4.00	散步	从不吸烟
144.50	64.00	94.00	30.65	每天	60.00	8.00	散步	吸烟
150.00	54.00	76.00	24.00	每天	30.00	1.00	散步	从不吸烟
160.00	56.00	78.00	21.87	不锻炼				从不吸烟
155.00	53.50	90.00	22.27	每天	60.00	1.00	散步	从不吸烟
153.00	66.00	100.00	28.19	每天	40.00	7.00	散步	从不吸烟
154.00	62.50	85.00	26.35	每天	90.00	5.00	散步	从不吸烟
151.00	51.00	84.00	22.37	每天	60.00	5.00	散步	从不吸烟

145.00	51.00	92.00	24.26	不锻炼				从不吸烟
149.00	70.50	97.00	31.76	不锻炼				从不吸烟
142.00	65.00	96.00	32.24	每天	60.00	7.00	瑜伽	从不吸烟
146.00	60.00	90.00	28.15	每天	60.00	10.00	散步	从不吸烟
158.00	59.00	93.00	23.63	每天	30.00	1.00	散步	从不吸烟
135.00	48.40	87.00	26.56	不锻炼				从不吸烟

日吸烟量	开始吸烟年	戒烟年龄	饮酒频率	日饮酒量	是否戒酒	戒酒年龄	开始饮酒年	饮酒种类
			从不					
			从不					
			从不					
			从不					
			从不					
			从不					
			从不					
			从不					
			从不					
			从不					
			从不					
			从不					
			从不					
			从不					
			从不					
			从不					
			从不					
			从不					
			从不					
			从不					
			从不					
			从不					
			从不					
			从不					
			从不					
			从不					
			从不					
			从不					
			从不					
			从不					
			从不					
			从不					
			从不					
			从不					
			从不					
			从不					
			从不					
			从不					
			从不					
			从不					
			从不					
			从不					
			从不					
			从不					
			从不					
			从不					
			从不					
			从不					
			从不					
			从不					

从不
从不
从不
从不
从不
从不
从不
从不
从不
从不
从不
从不
从不
从不
从不
从不
从不
从不
从不
从不
从不
从不
从不
从不
从不
从不
从不
从不
从不
从不
从不
从不
从不
从不
从不
从不
从不
从不
从不
从不
从不
从不
从不
从不
从不
从不
从不
从不
从不
从不
从不

从不
从不
从不
从不
从不
从不
从不
从不
从不
从不
从不
从不
从不
从不
从不
从不
从不
从不
从不
从不
从不
从不
从不
从不
从不
从不
从不
从不
从不
从不
从不
从不
从不
从不
从不
从不
从不
从不
从不
从不
从不
从不
从不
从不
从不
从不
从不
从不
从不
从不

		从不
		从不
		从不
		从不
		从不
		从不
		从不
		从不
		从不
		从不
		从不
		从不
		从不
		从不
		从不
		从不
		从不
		从不
		从不
		从不
		从不
		从不
		从不
		从不
		从不
		从不
		从不
		从不
		从不
		从不
		从不
		从不
		从不
		从不
		从不
		从不
		从不
		从不
		从不
		从不
		从不
		从不
		从不
		从不
		从不
		从不
3.00	16.00	从不
		从不
		从不
		从不
		从不

		从不
		从不
		从不
		从不
		从不
		从不
		从不
		从不
		从不
		从不
		从不
		从不
		从不
		从不
		从不
		从不
		从不
		从不
		从不
		从不
		从不
10.00	25.00	从不
		从不
		从不
		从不
		从不
		从不
		从不
		从不
		从不
		从不
		从不
		从不
		从不
		从不
		从不
		从不
		从不
		从不
		从不
		从不
		从不
		从不
		从不
		从不
		从不
		从不
		从不
		从不
		从不
		从不

		从不
		从不
		从不
		从不
		从不
		从不
		从不
		从不
		从不
		从不
		从不
		从不
		从不
		从不
		从不
		从不
		从不
		从不
		从不
		从不
		从不
		从不
		从不
		从不
		从不
		从不
		从不
		从不
		从不
		从不
		从不
		从不
		从不
20.00	17.00	从不
		从不
		从不
		从不
		从不
		从不
		从不
		从不
		从不
		从不
		从不
		从不
		从不
		从不
		从不
		从不
		从不
		从不

		从不
		从不
		从不
		从不
		从不
		从不
		从不
		从不
		从不
		从不
		从不
		从不
		从不
		从不
		从不
		从不
		从不
		从不
		从不
		从不
		从不
		从不
		从不
		从不
		从不
		从不
		从不
		从不
		从不
		从不
		从不
		从不
		从不
		从不
		从不
		从不
		从不
		从不
		从不
		从不
		从不
		从不
		从不
2.00	25.00	从不
		从不
		从不
		从不
		从不
		从不
		从不
		从不

		从不
		从不
		从不
		从不
		从不
		从不
		从不
		从不
		从不
		从不
		从不
		从不
		从不
		从不
6.00	20.00	从不
		从不
		从不
		从不
		从不
		从不
		从不
		从不
		从不
		从不
		从不
		从不
		从不
		从不
		从不
		从不
		从不
		从不
		从不
		从不
		从不
		从不
		从不
3.00	62.00	从不
		从不
		从不
		从不
		从不
		从不
		从不
		从不
		从不
		从不
		从不
		从不
		从不
		从不

		从不
		从不
20.00	20.00	从不
		从不
		从不
		从不
		从不
		从不
		从不
		从不
		从不
		从不
		从不
		从不
		从不
		从不
		从不
		从不
		从不
		从不
		从不
		从不
		从不
		从不
		从不
		从不
		从不
		从不
		从不
		从不
		从不
		从不
		从不
		从不
		从不
		从不
		从不
		从不
		从不
		从不
		从不
		从不
		从不
		从不
		从不
		从不
		从不
		从不
		从不
		从不
		从不

		从不				
		从不				
		从不				
		从不				
		从不				
		从不				
		从不				
		从不				
		从不				
		从不				
		从不				
		从不				
		从不				
		从不				
		从不				
		从不				
		从不				
		从不				
		从不				
		从不				
		从不				
		从不				
		从不				
		从不				
		从不				
		从不				
		从不				
		从不				
		从不				
		从不				
20.00	36.00	每天	0.50	未戒酒	72.00	白酒
		从不				
		从不				
		从不				
		从不				
		从不				
		从不				
		从不				
		从不				
		从不				
		从不				
		从不				
		从不				
		从不				
		从不				
		从不				
		从不				
		从不				
		从不				
		从不				
		从不				

从不
从不
从不
从不
从不
从不
从不
从不
从不
从不
从不
从不
从不
从不
从不
从不
从不
从不
从不
从不
从不
从不
从不
从不
从不
从不
从不
从不
从不
从不
从不
从不
从不
从不
从不
从不
从不
从不
从不
从不
从不
从不
从不
从不
从不
从不
从不
从不
从不
从不
从不

从不
从不
从不
从不
从不
从不
从不
从不
从不
从不
从不
从不
从不
从不
从不
从不
从不
从不
从不
从不
从不
从不
从不
从不
从不
从不
从不
从不
从不
从不
从不
从不
从不
从不
从不
从不
从不
从不
从不
从不
从不
从不
从不
从不
从不
从不
从不
从不
从不
从不
从不

		从不
		从不
		从不
		从不
		从不
		从不
		从不
		从不
		从不
		从不
		从不
		从不
		从不
		从不
		从不
		从不
		从不
		从不
		从不
2.00	30.00	从不
		从不
		从不
		从不
		从不
		从不
		从不
		从不
		从不
		从不
		从不
		从不
		从不
		从不
		从不
		从不
		从不
		从不
		从不
		从不
		从不
		从不
		从不
		从不
		从不
		从不
		从不
		从不
		从不
		从不
		从不
		从不

		从不				
		从不				
		从不				
		从不				
		从不				
		从不				
		从不				
		从不				
		从不				
		从不				
		从不				
3.00	25.00	从不				
		从不				
		从不				
		从不				
		从不				
		从不				
		从不				
		从不				
		从不				
		从不				
		从不				
		从不				
		从不				
		偶尔	2.00	未戒酒	52.00	黄酒
		从不				
		从不				
		从不				
		从不				
		从不				
		从不				
		从不				
		从不				
		从不				
		从不				
		从不				
		从不				
		从不				
		从不				
		从不				
		从不				
		从不				
		从不				
		从不				
		从不				
		从不				
		从不				
		从不				
		从不				
		从不				
		从不				

从不
从不
从不
从不
从不
从不
从不
从不
从不
从不
从不
从不
从不
从不
从不
从不
从不
从不
从不
从不
从不
从不
从不
从不
从不
从不
从不
从不
从不
从不
从不
从不
从不
从不
从不
从不
从不
从不
从不
从不
从不
从不
从不
从不
从不
从不
从不
从不
从不
从不
从不

从不
从不
从不
从不
从不
从不
从不
从不
从不
从不
从不
从不
从不
从不
从不
从不
从不
从不
从不
从不
从不
从不
从不
从不
从不
从不
从不
从不
从不
从不
从不
从不
从不
从不
从不
从不
从不
从不
从不
从不
从不
从不
从不
从不
从不
从不
从不
从不
从不
从不
从不

		从不
		从不
		从不
		从不
		从不
		从不
		从不
		从不
		从不
		从不
		从不
		从不
		从不
		从不
		从不
		从不
		从不
		从不
		从不
		从不
		从不
		从不
		从不
		从不
20.00	20.00	从不
		从不
		从不
		从不
		从不
		从不
		从不
		从不
		从不
		从不
		从不
		从不
		从不
		从不
		从不
		从不
		从不
		从不
		从不
		从不
		从不
		从不
		从不
		从不
		从不
		从不

从不
从不
从不
从不
从不
从不
从不
从不
从不
从不
从不
从不
从不
从不
从不
从不
从不
从不
从不
从不
从不
从不
从不
从不
从不
从不
从不
从不
从不
从不
从不
从不
从不
从不
从不
从不
从不
从不
从不
从不
从不
从不
从不
从不
从不
从不
从不
从不
从不
从不
从不

从不
从不
从不
从不
从不
从不
从不
从不
从不
从不
从不
从不
从不
从不
从不
从不
从不
从不
从不
从不
从不
从不
从不
从不
从不
从不
从不
从不
从不
从不
从不
从不
从不
从不
从不
从不
从不
从不
从不
从不
从不
从不
从不
从不
从不
从不
从不
从不
从不
从不
从不

从不
从不
从不
从不
从不
从不
从不
从不
从不
从不
从不
从不
从不
从不
从不
从不
从不
从不
从不
从不
从不
从不
从不
从不
从不
从不
从不
从不
从不
从不
从不
从不
从不
从不
从不
从不
从不
从不
从不
从不
从不
从不
从不
从不
从不
从不
从不
从不
从不
从不
从不

从不					
从不					
从不					
从不					
从不					
从不					
从不					
从不					
从不					
从不					
从不					
从不					
从不					
从不					
从不					
从不					
从不					
从不					
从不					
从不					
经常	2.00	已戒酒	80.00	65.00	白酒
从不					
从不					
从不					
从不					
从不					
从不					
从不					
从不					
从不					
从不					
从不					
从不					
从不					
从不					
从不					
从不					
从不					
从不					
从不					
从不					
从不					
从不					
从不					
从不					
从不					
从不					
从不					
从不					
从不					
从不					

从不
从不
从不
从不
从不
从不
从不
从不
从不
从不
从不
从不
从不
从不
从不
从不
从不
从不
从不
从不
从不
从不
从不
从不
从不
从不
从不
从不
从不
从不
从不
从不
从不
从不
从不
从不
从不
从不
从不
从不
从不
从不
从不
从不
从不
从不
从不
从不
从不
从不
从不

			从不
			从不
			从不
			从不
			从不
			从不
30.00	18.00		从不
			从不
			从不
			从不
			从不
			从不
			从不
			从不
			从不
			从不
			从不
			从不
			从不
			从不
			从不
			从不
			从不
			从不
			从不
			从不
			从不
			从不
			从不
			从不
			从不
			从不
5.00	12.00	85.00	从不
			从不
			从不
			从不
			从不
			从不
			从不
			从不
			从不
			从不
			从不
			从不
			从不
			从不
			从不
			从不
			从不
			从不
			从不
			从不
			从不
			从不
			从不

		从不
		从不
		从不
		从不
		从不
		从不
		从不
		从不
		从不
		从不
		从不
		从不
		从不
		从不
		从不
		从不
		从不
		从不
		从不
		从不
		从不
		从不
		从不
		从不
		从不
		从不
		从不
		从不
20.00	18.00	从不
		从不
		从不
		从不
		从不
		从不
		从不
		从不
		从不
		从不
		从不
		从不
		从不
		从不
		从不
		从不
		从不
		从不
		从不
		从不
		从不
		从不
		从不

			从不
			从不
			从不
			从不
			从不
			从不
			从不
			从不
			从不
			从不
			从不
			从不
10.00	30.00	50.00	从不
			从不
			从不
			从不
			从不
			从不
			从不
			从不
			从不
			从不
			从不
			从不
			从不
			从不
			从不
			从不
			从不
			从不
			从不
			从不
			从不
			从不
			从不
			从不
			从不
			从不
			从不
			从不
			从不
			从不
			从不
			从不
			从不
			从不
			从不
			从不
			从不
			从不
			从不

从不				
从不				
从不				
从不				
从不				
从不				
从不				
从不				
从不				
从不				
从不				
从不				
从不				
从不				
从不				
从不				
从不				
从不				
从不				
从不				
从不				
从不				
从不				
从不				
从不				
从不				
从不				
从不				
从不				
从不				
从不				
从不				
从不				
从不				
从不				
从不				
从不				
从不				
从不				
从不				
经常	1.00	未戒酒	25.00	白酒
从不				
从不				
从不				
从不				
从不				
从不				
从不				
从不				
从不				
从不				

从不
从不
从不
从不
从不
从不
从不
从不
从不
从不
从不
从不
从不
从不
从不
从不
从不
从不
从不
从不
从不
从不
从不
从不
从不
从不
从不
从不
从不
从不
从不
从不
从不
从不
从不
从不
从不
从不
从不
从不
从不
从不
从不
从不
从不
从不
从不
从不
从不
从不
从不
从不
从不
从不
从不
从不
从不

从不
从不
从不
从不
从不
从不
从不
从不
从不
从不
从不
从不
从不
从不
从不
从不
从不
从不
从不
从不
从不
从不
从不
从不
从不
从不
从不
从不
从不
从不
从不
从不
从不
从不
从不
从不
从不
从不
从不
从不
从不
从不
从不
从不
从不
从不
从不
从不
从不
从不
从不

从不
从不
从不
从不
从不
从不
从不
从不
从不
从不
从不
从不
从不
从不
从不
从不
从不
从不
从不
从不
从不
从不
从不
从不
从不
从不
从不
从不
从不
从不
从不
从不
从不
从不
从不
从不
从不
从不
从不
从不
从不
从不
从不
从不
从不
从不
从不
从不
从不
从不
从不

从不
从不
从不
从不
从不
从不
从不
从不
从不
从不
从不
从不
从不
从不
从不
从不
从不
从不
从不
从不
从不
从不
从不
从不
从不
从不
从不
从不
从不
从不
从不
从不
从不
从不
从不
从不
从不
从不
从不
从不
从不
从不
从不
从不
从不
从不
从不
从不
从不
从不
从不

从不
从不
从不
从不
从不
从不
从不
从不
从不
从不
从不
从不
从不
从不
从不
从不
从不
从不
从不
从不
从不
从不
从不
从不
从不
从不
从不
从不
从不
从不
从不
从不
从不
从不
从不
从不
从不
从不
从不
从不
从不
从不
从不
从不
从不
从不
从不
从不
从不
从不
从不

从不
从不
从不
从不
从不
从不
从不
从不
从不
从不
从不
从不
从不
从不
从不
从不
从不
从不
从不
从不
从不
从不
从不
从不
从不
从不
从不
从不
从不
从不
从不
从不
从不
从不
从不
从不
从不
从不
从不
从不
从不
从不
从不
从不
从不
从不
从不
从不
从不
从不
从不
从不
从不
从不
从不
从不
从不
从不
从不
从不
从不
从不
从不

从不
从不
从不
从不
从不
从不
从不
从不
从不
从不
从不
从不
从不
从不
从不
从不
从不
从不
从不
从不
从不
从不
从不
从不
从不
从不
从不
从不
从不
从不
从不
从不
从不
从不
从不
从不
从不
从不
从不
从不
从不
从不
从不
从不
从不
从不
从不
从不
从不
从不
从不

从不
从不
从不
从不
从不
从不
从不
从不
从不
从不
从不
从不
从不
从不
从不
从不
从不
从不
从不
从不
从不
从不
从不
从不
从不
从不
从不
从不
从不
从不
从不
从不
从不
从不
从不
从不
从不
从不
从不
从不
从不
从不
从不
从不
从不
从不
从不
从不
从不
从不
从不

从不
从不
从不
从不
从不
从不
从不
从不
从不
从不
从不
从不
从不
从不
从不
从不
从不
从不
从不
从不
从不
从不
从不
从不
从不
从不
从不
从不
从不
从不
从不
从不
从不
从不
从不
从不
从不
从不
从不
从不
从不
从不
从不
从不
从不
从不
从不
从不
从不
从不
从不
从不

从不				
从不				
从不				
从不				
从不				
从不				
从不				
从不				
从不				
从不				
从不				
从不				
从不				
从不				
从不				
从不				
从不				
从不				
从不				
从不				
从不				
从不				
从不				
从不				
从不				
从不				
从不				
从不				
从不				
从不				
从不				
从不				
每天	2.00	未戒酒	40.00	白酒
从不				
从不				
从不				
从不				
从不				
从不				
从不				
从不				
从不				
从不				
从不				
从不				
从不				
从不				
从不				
从不				
从不				
从不				

从不				
从不				
每天	2.00	未戒酒	18.00	白酒
从不				
从不				
从不				
从不				
从不				
从不				
从不				
从不				
从不				
从不				
从不				
从不				
从不				
从不				
从不				
从不				
从不				
从不				
从不				
从不				
从不				
从不				
从不				
从不				
从不				
从不				
从不				
从不				
从不				
从不				
从不				
从不				
从不				
从不				
从不				
从不				
从不				
从不				
从不				
从不				
从不				
从不				
从不				
从不				
从不				
从不				
从不				
从不				

		从不
		从不
		从不
		从不
		从不
		从不
		从不
		从不
		从不
		从不
		从不
		从不
		从不
		从不
		从不
		从不
		从不
		从不
		从不
		从不
		从不
		从不
		从不
		从不
		从不
		从不
		从不
8.00	16.00	从不
		从不
		从不
		从不
		从不
		从不
		从不
		从不
		从不
		从不
		从不
		从不
		从不
		从不
		从不
		从不
		从不
		从不
		从不
		从不
		从不
		从不
		从不
		从不

从不
从不
从不
从不
从不
从不
从不
从不
从不
从不
从不
从不
从不
从不
从不
从不
从不
从不
从不
从不
从不
从不
从不
从不
从不
从不
从不
从不
从不
从不
从不
从不
从不
从不
从不
从不
从不
从不
从不
从不
从不
从不
从不
从不
从不
从不
从不
从不
从不
从不
从不

从不
从不
从不
从不
从不
从不
从不
从不
从不
从不
从不
从不
从不
从不
从不
从不
从不
从不
从不
从不
从不
从不
从不
从不
从不
从不
从不
从不
从不
从不
从不
从不
从不
从不
从不
从不
从不
从不
从不
从不
从不
从不
从不
从不
从不
从不
从不
从不
从不
从不
从不

从不
从不
从不
从不
从不
从不
从不
从不
从不
从不
从不
从不
从不
从不
从不
从不
从不
从不
从不
从不
从不
从不
从不
从不
从不
从不
从不
从不
从不
从不
从不
从不
从不
从不
从不
从不
从不
从不
从不
从不
从不
从不
从不
从不
从不
从不
从不
从不
从不
从不
从不

从不
从不
从不
从不
从不
从不
从不
从不
从不
从不
从不
从不
从不
从不
从不
从不
从不
从不
从不
从不
从不
从不
从不
从不
从不
从不
从不
从不
从不
从不
从不
从不
从不
从不
从不
从不
从不
从不
从不
从不
从不
从不
从不
从不
从不
从不
从不
从不
从不
从不
从不

从不
从不
从不
从不
从不
从不
从不
从不
从不
从不
从不
从不
从不
从不
从不
从不
从不
从不
从不
从不
从不
从不
从不
从不
从不
从不
从不
从不
从不
从不
从不
从不
从不
从不
从不
从不
从不
从不
从不
从不
从不
从不
从不
从不
从不
从不
从不
从不
从不
从不
从不

		从不
		从不
		从不
		从不
		从不
		从不
		从不
		从不
		从不
		从不
		从不
		从不
		从不
		从不
		从不
		从不
		从不
		从不
		从不
		从不
		从不
		从不
		从不
		从不
		从不
		从不
		从不
		从不
		从不
		从不
		从不
		从不
		从不
		从不
5.00	18.00	从不
		从不
		从不
		从不
		从不
		从不
		从不
		从不
		从不
		从不
		从不
		从不
		从不
		从不
		从不
		从不
		从不

		从不
		从不
		从不
		从不
		从不
		从不
		从不
		从不
		从不
		从不
		从不
		从不
		从不
		从不
		从不
		从不
		从不
		从不
		从不
		从不
		从不
		从不
		从不
		从不
		从不
		从不
		从不
		从不
		从不
		从不
		从不
		从不
		从不
		从不
		从不
		从不
		从不
		从不
		从不
		从不
		从不
		从不
		从不
		从不
		从不
		从不
		从不
		从不
		从不
6.00	19.00	从不
		从不

从不
从不
从不
从不
从不
从不
从不
从不
从不
从不
从不
从不
从不
从不
从不
从不
从不
从不
从不
从不
从不
从不
从不
从不
从不
从不
从不
从不
从不
从不
从不
从不
从不
从不
从不
从不
从不
从不
从不
从不
从不
从不
从不
从不
从不
从不
从不
从不
从不
从不
从不

从不
从不
从不
从不
从不
从不
从不
从不
从不
从不
从不
从不
从不
从不
从不
从不
从不
从不
从不
从不
从不
从不
从不
从不
从不
从不
从不
从不
从不
从不
从不
从不
从不
从不
从不
从不
从不
从不
从不
从不
从不
从不
从不
从不
从不
从不
从不
从不
从不
从不
从不

从不
从不
从不
从不
从不
从不
从不
从不
从不
从不
从不
从不
从不
从不
从不
从不
从不
从不
从不
从不
从不
从不
从不
从不
从不
从不
从不
从不
从不
从不
从不
从不
从不
从不
从不
从不
从不
从不
从不
从不
从不
从不
从不
从不
从不
从不
从不
从不
从不
从不
从不

从不				
从不				
从不				
从不				
从不				
从不				
从不				
从不				
从不				
从不				
从不				
从不				
从不				
从不				
从不				
从不				
从不				
从不				
从不				
从不				
从不				
从不				
从不				
从不				
从不				
从不				
从不				
经常	0.50	未戒酒	50.00	白酒,其他
从不				
从不				
从不				
从不				
从不				
从不				
从不				
从不				
从不				
从不				
从不				
从不				
从不				
从不				
从不				
从不				
从不				
从不				
从不				
从不				
从不				
从不				
从不				

从不
从不
从不
从不
从不
从不
从不
从不
从不
从不
从不
从不
从不
从不
从不
从不
从不
从不
从不
从不
从不
从不
从不
从不
从不
从不
从不
从不
从不
从不
从不
从不
从不
从不
从不
从不
从不
从不
从不
从不
从不
从不
从不
从不
从不
从不
从不
从不
从不
从不
从不

从不
从不
从不
从不
从不
从不
从不
从不
从不
从不
从不
从不
从不
从不
从不
从不
从不
从不
从不
从不
从不
从不
从不
从不
从不
从不
从不
从不
从不
从不
从不
从不
从不
从不
从不
从不
从不
从不
从不
从不
从不
从不
从不
从不
从不
从不
从不
从不
从不
从不
从不

从不
从不
从不
从不
从不
从不
从不
从不
从不
从不
从不
从不
从不
从不
从不
从不
从不
从不
从不
从不
从不
从不
从不
从不
从不
从不
从不
从不
从不
从不
从不
从不
从不
从不
从不
从不
从不
从不
从不
从不
从不
从不
从不
从不
从不
从不
从不
从不
从不
从不
从不

从不
从不
从不
从不
从不
从不
从不
从不
从不
从不
从不
从不
从不
从不
从不
从不
从不
从不
从不
从不
从不
从不
从不
从不
从不
从不
从不
从不
从不
从不
从不
从不
从不
从不
从不
从不
从不
从不
从不
从不
从不
从不
从不
从不
从不
从不
从不
从不
从不
从不
从不

从不
从不
从不
从不
从不
从不
从不
从不
从不
从不
从不
从不
从不
从不
从不
从不
从不
从不
从不
从不
从不
从不
从不
从不
从不
从不
从不
从不
从不
从不
从不
从不
从不
从不
从不
从不
从不
从不
从不
从不
从不
从不
从不
从不
从不
从不
从不
从不
从不
从不
从不

从不
从不
从不
从不
从不
从不
从不
从不
从不
从不
从不
从不
从不
从不
从不
从不
从不
从不
从不
从不
从不
从不
从不
从不
从不
从不
从不
从不
从不
从不
从不
从不
从不
从不
从不
从不
从不
从不
从不
从不
从不
从不
从不
从不
从不
从不
从不
从不
从不
从不
从不

从不
从不
从不
从不
从不
从不
从不
从不
从不
从不
从不
从不
从不
从不
从不
从不
从不
从不
从不
从不
从不
从不
从不
从不
从不
从不
从不
从不
从不
从不
从不
从不
从不
从不
从不
从不
从不
从不
从不
从不
从不
从不
从不
从不
从不
从不
从不
从不
从不
从不
从不
从不

		从不
		从不
		从不
		从不
		从不
		从不
		从不
		从不
		从不
		从不
		从不
		从不
		从不
		从不
		从不
		从不
		从不
		从不
		从不
		从不
		从不
		从不
		从不
4.00	18.00	从不
		从不
		从不
		从不
		从不
		从不
		从不
		从不
		从不
		从不
		从不
		从不
		从不
		从不
		从不
		从不
		从不
		从不
		从不
		从不
		从不
		从不
		从不
		从不
		从不
		从不
		从不
		从不

		从不
		从不
		从不
20.00	7.00	从不
		从不
		从不
		从不
		从不
		从不
		从不
		从不
		从不
		从不
		从不
		从不
		从不
		从不
		从不
		从不
		从不
		从不
		从不
		从不
		从不
		从不
		从不
		从不
		从不
		从不
		从不
		从不
		从不
		从不
		从不
		从不
		从不
		从不
		从不
		从不
		从不
		从不
		从不
		从不
		从不
		从不
		从不
		从不
		从不
		从不
		从不
		从不

5.00	20.00	从不
		从不
		从不
		从不
		从不
		从不
		从不
		从不
		从不
		从不
		从不
		从不
		从不
		从不
		从不
		从不
		从不
		从不
		从不
		从不
		从不
		从不
		从不
		从不
		从不
		从不
		从不
		从不
		从不
		从不
		从不
		从不
		从不
		从不
		从不
		从不
		从不
		从不
		从不
		从不
		从不
		从不
		从不
		从不
5.00	24.00	从不
		从不
		从不
		从不
		从不
		从不
		从不

从不
从不
从不
从不
从不
从不

饮酒种类	空腹血糖	总胆固醇	甘油三脂	血清低密	血清高密	B超检查异	B超检查异常描述
	5.01	6.69	2.09	3.66	1.74	异常	脂肪肝声像
	6.06	6.63	1.82	3.80	1.53	异常	轻度脂肪肝声像
	4.85	6.59	1.50	3.67	1.59	异常	胆囊内强回声（考虑
	4.68	5.03	0.81	2.73	1.40	未见异常	
	5.02	6.42	1.98	3.70	1.64	未见异常	
	5.05	5.46	0.81	2.50	2.06	未见异常	
	6.10	4.08	1.99	1.94	1.46	异常	肝囊肿；肝内强回声
	6.13	5.71	1.36	3.18	1.59	异常	1、脂肪肝声像；2、
	6.23	5.93	2.76	3.33	1.37	异常	腹部B超，脂肪肝声
	6.52	7.30	1.20	4.50	1.97	异常	轻度脂肪肝声像。
	5.65	5.46	1.13	2.65	1.98	未见异常	
	4.88	7.06	1.29	4.54	1.76	异常	轻度脂肪肝声像
	4.57	4.41	1.82	2.17	1.26	未见异常	
	5.25	3.49	1.25	1.65	1.37	异常	轻度脂肪肝声像
	6.90	6.06	1.57	3.53	1.66	未见异常	
	5.53	4.10	2.15	2.05	1.34	异常	胆囊结石
	4.08	5.60	2.59	2.86	1.47	异常	1、脂肪肝声像2、胆
	4.83	6.32	2.10	3.44	1.55	未见异常	
	5.65	7.30	2.22	4.23	1.74	异常	脂肪肝声像
	4.88	6.21	2.46	3.43	1.89	未见异常	
	4.41	5.39	1.19	2.51	1.93	未见异常	
	4.65	6.84	1.55	4.06	1.90	异常	脂肪肝，肝内强回声
	6.26	5.62	1.90	3.41	1.39	异常	脂肪肝声像。
	6.95	6.81	3.31	3.71	1.77	异常	脂肪肝声像；
	4.73	3.92	0.69	1.77	1.51	未见异常	
	4.82	4.21	0.86	1.67	1.85	未见异常	
	5.69	3.63	2.74	1.70	1.26	异常	脂肪肝声像
	5.97	7.38	6.34	2.71	1.67	异常	1、脂肪肝声像2、肝
	5.44	9.22	1.72	6.10	2.23	异常	轻度脂肪肝声像
	15.48	7.33	3.87	3.87	1.57	异常	脂肪肝声像
	10.87	4.81	1.39	2.51	1.64	异常	脂肪肝声像。
	18.06	8.18	2.15	5.16	2.04	异常	脂肪肝声像；
	4.61	4.26	1.21	2.01	1.71	未见异常	
	5.36	5.21	1.33	2.89	1.46	异常	脂肪肝声像
	7.77	6.11	2.74	3.35	1.65	异常	轻度脂肪肝声像
	5.17	6.58	2.01	2.99	2.37	未见异常	
	6.10	6.36	2.56	3.49	1.60	异常	轻度脂肪肝声像
	5.67	5.28	1.31	2.31	1.95	未见异常	
	5.02	4.60	1.59	2.53	1.28	异常	脂肪肝声像
	5.01	7.07	1.49	4.04	1.75		
	4.62	7.45	1.61	4.43	1.98	异常	轻度脂肪肝声像
	5.19	4.45	3.51	1.91	1.37	异常	轻度脂肪肝声像
	4.78	4.87	0.66	2.35	1.84		
	5.29	5.79	1.32	2.99	1.81	未见异常	
	5.46	7.66	1.17	5.00	1.86		
	5.20	5.34	0.65	3.01	1.56	未见异常	
	5.49	6.21	1.28	3.70	1.75		
	4.92	7.68	2.96	4.30	1.84	异常	脂肪肝声像
	5.02	6.01	1.63	3.38	1.45		
	5.13	7.20	0.89	4.49	1.70	异常	肝实质回声稍粗、肝

5.25	4.80	2.77	2.35	1.51	未见异常	
4.80	5.58	2.62	3.24	1.49	异常	胆囊结石
4.99	6.51	1.29	3.71	1.68	异常	胆囊内强回声(考虑
5.04	4.20	1.88	1.89	1.62	异常	胆囊内强回声（考虑
5.77	6.42	1.34	2.79	2.42	异常	胆囊结石
8.37	4.75	0.98	2.19	1.84	未见异常	
5.35	5.20	1.40	2.82	1.76	异常	未除胆囊结石
6.91	4.98	2.78	2.43	1.36	异常	轻度脂肪肝声像
7.37	6.64	1.56	3.83	1.82	异常	脂肪酐声像、胆囊内
3.98	7.47	1.13	4.24	2.17	未见异常	
5.72	5.98	1.69	3.68	1.55	异常	肝囊肿
5.34	6.29	0.73	3.40	2.03	异常	1、肝左叶实质回声
4.69	4.18	3.17	2.08	1.07	异常	脂肪肝声像，肝囊
16.88	3.53	2.65	1.62	1.26	异常	脂肪肝声像
5.86	4.61	1.98	2.52	1.43	异常	胆囊结石
4.97	5.39	1.14	3.19	1.37		
4.45	5.00	1.32	2.30	1.95	未见异常	
6.83	11.80	17.83	3.25	1.17	异常	脂肪肝
5.08	5.68	2.42	2.63	1.89	未见异常	
4.67	5.23	0.84	2.85	1.88	异常	1、脂肪肝声像；2、
6.35	5.09	1.70	2.75	1.37	异常	腹部B超：胆囊壁欠
13.11	3.49	1.37	1.58	1.31	异常	腹部B超，脂肪肝声
4.99	5.59	3.85	2.69	1.32	异常	轻度脂肪肝声像
5.10	4.33	1.25	2.34	1.33	未见异常	
5.98	5.92	2.27	3.12	1.52	异常	腹部B超；1.脂肪肝
7.34	4.39	2.25	2.23	1.61	异常	1.肝脏弥漫性改变.
7.66	4.63	2.54	2.25	1.41	异常	1.胆囊缩小 2.胆总
4.83	6.70	3.40	3.44	1.49	异常	肝内多发囊性回声（
5.06	7.14	1.86	4.45	1.60	异常	1.脂肪肝声像 2.肝
6.74	5.83	2.17	3.13	1.67		
5.09	3.59	1.25	1.59	1.49	未见异常	
5.15	3.96	1.99	1.69	1.55	异常	脂肪肝声像
11.94	4.66	5.30	1.72	1.22	异常	脂肪肝声像
5.03	5.51	1.86	2.96	1.64	异常	胆囊内强回声,肝内
5.00	5.17	2.20	2.73	1.47		
6.68	5.15	2.17	2.68	1.52	异常	轻度脂肪肝声像
7.43	5.27	2.01	2.93	1.48	异常	腹部B超：脂肪肝声
4.81	5.28	0.97	2.58	1.80	未见异常	
5.94	5.30	2.63	2.85	1.46	异常	轻度脂肪肝声像
6.26	6.25	3.45	3.30	1.55	异常	脂肪肝声像
4.45	8.21	1.86	4.79	2.28	异常	轻度脂肪肝声像，肝
6.33	6.37	1.36	3.78	1.54	异常	脂肪肝声像
5.18	6.47	4.70	2.55	1.66	异常	轻度脂肪肝声像
6.91	5.81	2.66	3.16	1.59	异常	轻度脂肪肝声像
5.47	5.38	7.74	2.50	1.16	异常	轻度脂肪肝声像。
4.72	6.35	2.03	4.09	1.41	异常	1、脂肪肝声像；2、
4.65	4.40	1.80	2.32	1.44	未见异常	
5.79	7.31	1.84	4.26	1.61	未见异常	
5.27	7.73	1.84	4.60	2.22	异常	轻度脂肪肝声像
5.12	7.27	1.58	4.42	1.79	未见异常	
4.60	6.21	1.63	3.53	1.64	异常	1、脂肪肝声像；2、

5.18	5.30	1.29	2.91	1.73	异常	肝囊肿
4.89	6.86	2.05	4.25	1.43	未见异常	
4.79	5.89	1.25	2.93	2.21	异常	胆囊结石
6.21	6.88	1.38	3.61	1.98	异常	1脂肪肝声像　2胆囊
4.93	5.99	1.25	3.72	1.48	异常	胆囊切除术后
5.90	6.56	2.47	3.23	1.83	异常	肝左叶增大，肝右叶
6.35	6.14	1.51	3.35	1.96	异常	轻度脂肪肝声像
5.78	7.03	1.79	4.15	1.58	异常	轻度脂肪肝声像
4.76	5.05	1.45	2.77	1.52		
4.39	4.72	1.52	2.44	1.50	未见异常	
4.77	7.27	1.42	4.36	1.86	异常	轻度脂肪肝
4.65	6.42	0.56	3.24	2.38	未见异常	
5.11	5.62	1.42	3.21	1.45	异常	轻度脂肪肝声像
4.87	6.96	1.84	3.07	2.32	未见异常	
4.77	5.01	1.24	2.87	1.50	异常	轻度脂肪肝声像
5.33	7.56	1.49	3.85	1.82	未见异常	
4.76	4.14	0.94	2.01	1.45	未见异常	
4.82	5.29	3.15	2.68	1.51	异常	轻度脂肪肝声像
5.58	6.59	2.04	3.57	1.76	异常	腹部B超，轻度脂肪[illegible]
6.09	4.48	2.51	1.97	1.49	未见异常	
4.78	4.70	0.88	2.57	1.66		
5.65	2.58	1.47	0.88	1.07	未见异常	
5.98	5.83	1.88	3.30	1.56	异常	脂肪肝声像
5.59	5.50	2.92	2.90	1.51	未见异常	
4.40	4.99	1.90	2.65	1.47	异常	脂肪肝声像
4.65	5.00	0.91	3.00	1.18	异常	脂肪肝
4.73	4.32	0.76	1.77	1.79	异常	腹部B超1.肝内囊性[illegible]
4.93	4.76	1.89	2.26	1.68	异常	肝实质回声稍粗
5.12	5.46	1.92	3.00	1.57	异常	脂肪肝声像
6.68	5.17	2.86	2.57	1.59	异常	1、脂肪肝声像　2、
6.98	3.36	1.59	1.61	1.24	异常	轻度脂肪肝声像
6.84	4.32	2.06	2.27	1.11	异常	腹部B超；脂肪肝声[illegible]
7.76	4.25	7.34	1.05	0.94	异常	轻度脂肪肝声像
4.83	7.82	2.55	4.83	1.69	未见异常	
5.30	6.00	1.44	3.48	1.72	未见异常	
5.25	5.11	2.71	2.64	1.52	异常	轻度脂肪肝声像
5.35	5.14	3.27	2.23	1.65	异常	脂肪肝声像
4.60	5.35	1.31	2.68	1.68	未见异常	
4.95	5.53	1.45	2.99	1.76	未见异常	
4.82	7.50	1.50	4.65	1.67	异常	胆囊结石
5.39	4.57	0.87	1.99	1.90	未见异常	
8.40	5.97	1.18	3.31	1.73	异常	1、脂肪肝声像2、肝
9.38	5.57	1.31	3.10	1.54	异常	脂肪肝声像
6.42	5.41	1.11	2.94	1.75	异常	1、轻度脂肪肝声像；
6.09	4.94	2.10	2.71	1.29	异常	胆囊切除术后
4.96	5.96	2.11	2.66	2.19	异常	轻度脂肪肝声像，肝
5.05	6.24	2.03	3.77	1.32	异常	胆囊结石
4.77	7.38	4.28	3.38	2.01	异常	1.轻度脂肪肝声像
4.97	4.38	1.84	2.20	1.48	异常	轻度脂肪肝声像
16.75	4.95	4.73	2.10	1.24	异常	1、脂肪肝声像；2、
6.85	3.57	2.26	1.62	1.43		

5.22	5.10	1.00	2.69	1.73	未见异常	
6.49	4.00	3.74	1.68	1.18	异常	餐后胆囊，胆囊内异
6.19	5.04	2.11	2.74	1.34	未见异常	
5.58	4.88	0.99	2.58	1.59	未见异常	
4.70	5.08	1.06	2.69	1.63	未见异常	
5.79	5.27	1.53	3.18	1.42	异常	脂肪肝声像。肝内多
5.19	6.39	2.60	3.78	1.56	异常	脂肪肝声像
4.61	7.49	1.99	3.90	2.13	未见异常	
6.47	5.39	1.25	3.07	1.50	异常	轻度脂肪肝声像
4.53	8.16	4.00	3.62	2.18	未见异常	
4.74	5.54	1.14	2.83	1.91	未见异常	
9.33	7.03	3.42	4.03	1.43	异常	脂肪肝声像。
4.79	4.86	1.02	2.25	1.98	未见异常	
5.47	3.53	0.86	1.61	1.34	未见异常	
4.39	4.70	1.12	2.50	1.36	异常	1.肝实质回声稍粗
4.96	6.56	2.02	3.63	1.86	未见异常	
7.53	4.91	1.47	2.21	2.01	异常	胆囊内强回声，结石
6.11	5.19	2.04	2.48	1.76	异常	轻度脂肪肝声像
8.68	5.61	3.15	3.24	1.17	异常	胆囊结石
5.48	6.12	2.53	3.33	1.55	未见异常	
6.41	6.21	3.23	3.28	1.49	异常	1肝右叶稍高回声结
4.60	5.94	1.39	3.59	1.57	未见异常	
4.56	5.61	0.93	2.76	2.03	异常	腹部B超；肝内囊性
5.28	4.73	2.65	2.39	1.32	异常	脂肪肝声像
4.88	5.93	3.02	3.28	1.33	异常	轻度脂肪肝声像
4.67	4.95	1.65	2.44	1.54	未见异常	
7.31	5.72	1.84	3.36	1.50	异常	1、轻度脂肪肝声像；
6.61	4.00	2.66	1.79	1.41	未见异常	
5.44	4.07	1.79	2.13	1.36	异常	1、 轻度脂肪肝声像
4.57	5.98	0.73	2.74	1.98	未见异常	
8.65	4.20	1.55	1.51	1.93	异常	肝胆胰彩超：1、脂
5.15	5.42	1.15	3.17	1.59	未见异常	
5.17	6.85	6.59	2.28	1.51	未见异常	
4.89	6.55	1.83	4.01	1.49	异常	胆囊多发性结石
4.58	5.56	1.83	2.78	1.68	异常	肝囊肿
6.41	6.33	2.39	3.41	1.60		
5.17	4.44	0.61	2.38	1.48	未见异常	
5.59	5.46	3.90	2.44	1.48	未见异常	
6.12	5.20	2.00	2.71	1.48	异常	脂肪肝声像
6.44	6.19	5.48	2.16	1.72	异常	脂肪肝声像；
5.19	4.00	0.78	1.98	1.55	异常	脂肪肝声像
5.71	6.22	4.32	3.12	1.39	异常	脂肪肝声像
6.87	7.06	1.87	3.96	1.92	异常	脂肪肝声像
5.16	4.27	3.20	2.05	1.12	未见异常	
8.03	5.86	1.07	3.29	1.70	异常	轻度脂肪肝声像、胆
5.00	4.88	1.50	2.32	1.71	未见异常	
6.94	6.75	1.77	3.63	2.06	未见异常	
4.11	3.34	0.55	1.64	1.22		
4.84	5.64	1.04	3.19	1.74	未见异常	
4.80	4.63	3.80	1.62	1.68	异常	肝内强回声斑，考虑
5.52	5.54	1.45	3.07	1.49	异常	脂肪肝声像

8.09	8.05	1.82	5.42	1.64	异常	1、脂肪肝声像；2、
5.90	4.66	1.73	2.44	1.44	未见异常	
4.99	4.48	1.51	2.26	1.49	异常	脂肪肝声像
5.37	6.38	1.39	3.61	1.62	未见异常	
4.97	6.41	2.05	3.71	1.53	异常	胆囊壁稍厚
3.92	5.37	1.46	2.87	1.72	未见异常	
5.88	5.30	2.29	2.59	1.94		
5.34	3.82	1.08	1.74	1.50	异常	1、肝内囊 性回声（
5.13	5.53	1.48	3.07	1.75		
14.85	3.17	10.90	1.43	1.30	未见异常	
4.75	5.15	1.65	2.78	1.36	未见异常	
4.83	4.84	0.82	2.11	2.01	异常	1、肝实质回声稍粗；
6.28	6.25	1.85	3.49	1.51		
4.89	4.56	1.34	2.03	1.75	异常	肝小囊肿
4.87	6.42	1.50	4.04	1.37	异常	1、脂肪肝声像；2、
5.33	4.23	0.98	2.30	1.37	未见异常	
4.45	5.22	0.97	2.62	1.69	异常	肝囊肿
5.36	5.92	2.36	3.25	1.58	异常	轻度脂肪肝声像
6.18	6.14	3.12	3.26	1.60	异常	1.脂肪肝声像
4.82	4.34	2.26	2.25	1.21		
10.06	2.96	3.40	1.31	1.02		
4.73	3.53	1.79	1.42	1.34	未见异常	
5.69	4.60	0.78	1.61	2.10	未见异常	
5.39	6.69	1.71	4.13	1.56	未见异常	
5.26	4.23	1.26	1.62	1.85	未见异常	
5.23	5.00	0.92	2.78	1.63	异常	肝右叶稍强回声结节
4.82	8.57	2.57	4.86	1.98	异常	轻度脂肪肝声像
4.80	5.15	3.01	2.40	1.46	异常	轻度脂肪肝声像
5.07	8.80	5.15	4.13	1.87	异常	腹部B超；脂肪肝声
4.35	7.11	15.10	4.12	1.96		
4.31	6.21	1.00	3.05	1.94	异常	腹部B超；轻度脂肪
5.32	7.18	3.99	3.47	1.80		
5.39	5.95	1.89	3.19	1.75		
5.32	6.85	0.95	3.48	2.38	未见异常	
6.39	7.63	1.53	4.53	2.30		
4.32	5.10	2.55	2.24	1.67	异常	腹部B超：肝囊肿
4.48	5.84	2.92	2.95	1.45	异常	脂肪肝声像
5.45	5.90	0.93	3.44	1.67	异常	轻度脂肪肝声像
5.43	4.60	0.79	2.40	1.65		
5.27	4.46	1.71	2.25	1.38	未见异常	
4.75	7.30	1.36	4.30	1.81		
5.41	6.99	4.68	3.21	1.36	异常	脂肪肝声像
5.81	5.87	3.82	2.83	1.37	异常	脂肪肝声像
5.92	4.71	2.52	2.40	1.40	异常	脂肪肝声像
4.54	5.57	1.22	3.23	1.69	未见异常	
5.41	4.26	0.70	2.26	1.40	未见异常	
5.52	6.56	1.35	4.07	1.66	异常	脂肪肝声像
5.32	5.93	2.75	3.18	1.55	异常	1.轻度脂肪肝声像2.
4.94	6.38	1.96	3.92	1.42	异常	脂肪肝声像
5.17	5.26	1.93	2.84	1.57	异常	脂肪肝声像
4.59	3.18	1.55	5.74	2.13	未见异常	

5.46	5.04	1.67	2.70	1.56	异常	轻度脂肪肝声像
4.58	4.05	3.35	1.64	1.51	异常	脂肪肝声像
7.56	5.50	4.17	2.78	1.19		
5.21	6.93	1.56	4.03	1.73	未见异常	
5.24	6.83	3.87	3.10	1.82	未见异常	
6.04	5.57	2.40	2.97	1.61	未见异常	
17.42	3.14	4.09	1.06	1.18	异常	脂肪肝声像
5.89	5.04	1.13	3.01	1.42		
5.97	5.43	1.06	2.74	1.82	未见异常	
5.70	7.13	2.56	3.96	1.59	异常	脂肪肝声像
5.88	6.07	3.15	3.09	1.79	异常	脂肪肝声像。
4.77	5.49	1.72	2.29	2.17	未见异常	
5.60	7.33	1.99	3.88	2.10	异常	肝囊肿
4.92	6.32	1.31	3.61	1.59	未见异常	
14.46	4.86	1.96	2.79	1.29	异常	1、脂肪肝声像；2、
5.36	3.66	1.33	1.62	1.50	未见异常	
5.20	4.19	1.22	2.37	1.22	异常	肝内囊性回声（考虑
8.56	5.44	5.27	2.03	1.43	异常	1、脂肪肝声像2、肝
7.13	6.49	1.46	3.96	1.74	异常	脂肪肝声像
5.19	4.88	1.16	1.99	2.10	未见异常	
5.45	5.02	2.71	2.44	1.69	异常	轻度脂肪肝声像
4.77	8.31	2.64	4.34	1.93	异常	轻度脂肪肝声像；肝
5.91	6.68	9.10	1.47	1.12	异常	脂肪肝声像
6.39	6.57	3.56	3.32	1.67	未见异常	
4.52	4.78	1.26	2.24	1.68	未见异常	
5.07	8.32	0.86	4.46	2.21	未见异常	
5.03	4.70	1.96	2.35	1.38	异常	1、轻度脂肪肝声像；
7.79	8.38	3.68	4.37	1.75	异常	轻度脂肪肝声像
5.37	5.08	1.88	2.99	1.20	异常	胆囊内强回声（考虑
5.42	6.90	0.89	3.64	2.10	未见异常	
5.00	6.31	2.78	3.05	1.75	异常	脂肪肝声像
4.61	6.24	1.75	3.46	1.39	异常	脂肪肝声像、肝内小
4.28	5.56	3.79	2.64	1.59	异常	1.轻度脂肪肝声像2.
5.42	5.61	1.61	2.23	2.17	异常	肝实质回声增粗，请
4.80	4.62	2.63	2.38	1.40		
4.27	6.74	1.13	3.57	1.96	未见异常	
4.96	6.66	2.03	2.23	2.23	未见异常	
5.39	5.98	1.68	3.63	1.49		
5.27	8.97	2.88	5.35	1.82		
5.53	6.37	1.87	3.97	1.46	异常	脂肪肝声像
7.77	7.98	1.58	4.86	1.70	异常	脂肪肝声像；胆囊内
5.81	3.79	1.69	1.59	1.44	未见异常	
6.50	4.44	1.46	2.09	1.61	未见异常	
5.27	5.39	1.42	2.68	1.86		
6.00	5.36	0.94	2.85	1.76	未见异常	
4.91	4.17	0.80	1.67	1.86	未见异常	
4.78	5.82	3.93	2.54	1.65		
5.16	5.55	0.90	3.36	1.60	异常	肝囊肿
7.49	5.57	1.38	3.20	1.63	异常	肝多发囊肿、胆囊稍
4.89	5.96	0.73	2.72	2.22	异常	1、胆囊内强回声（
5.25	4.57	1.78	2.38	4.53		

4.59	6.65	1.10	2.67	2.51	异常	肝内囊性回声（考虑
5.68	6.36	5.09	2.45	1.67	异常	脂肪肝声像、肝囊肿
8.93	5.06	4.92	2.02	1.25		
16.63	8.86	5.93	3.45	1.75	异常	脂肪肝声像
5.21	5.60	1.10	2.41	2.10	未见异常	
5.42	6.33	2.14	3.44	1.70	异常	轻度脂肪肝声像，胆
5.92	6.12	0.98	3.45	1.82	异常	肝实质回声稍粗
5.74	4.96	1.52	2.13	2.00	异常	胆囊内强回声（考虑
5.41	5.32	0.61	2.78	1.85	异常	1.肝内外胆管强回声
8.08	5.02	1.86	2.88	1.39	异常	轻度脂肪肝声像
5.09	5.47	1.40	2.55	2.09	未见异常	
5.22	5.51	1.27	3.17	1.70	未见异常	
6.10	6.15	1.79	3.00	2.20	未见异常	
5.09	6.77	2.25	4.37	1.46	异常	肝内强回声斑，考虑
5.24	5.23	2.53	2.45	1.83	异常	脂肪肝声像
6.70	5.92	1.34	3.00	1.91	异常	轻度脂肪肝声像
4.83	8.84	23.04	1.56	0.88	异常	胆囊内似见强回声（
10.18	5.95	1.72	3.29	1.78		
7.63	5.77	1.27	2.30	2.37	异常	胆囊内强回声（考虑
5.70	4.25	1.46	2.23	1.33	异常	肝内稍强回声结节（
4.85	5.05	1.06	2.92	1.62		
9.09	6.20	2.01	3.68	1.52		
5.54	5.31	0.93	1.89	2.54	未见异常	
4.98	5.55	1.24	3.21	1.64	异常	轻度脂肪肝声像、肝
5.62	3.75	1.19	1.67	1.62	未见异常	
5.43	3.06	0.74	1.09	1.59	异常	脂肪肝声像
6.50	6.49	2.28	3.61	1.74	异常	1、脂肪肝声像；2、
4.59	4.78	1.46	2.69	1.29	异常	胆囊壁胆固醇结晶沉
8.32	3.79	0.90	1.67	1.53	未见异常	
4.90	8.74	4.75	4.26	1.99		
4.93	6.94	1.39	3.87	1.83	异常	胆囊结石
4.88	4.12	1.75	2.12	1.31	异常	所示范围内肝实质回
4.66	5.34	0.90	2.42	1.92	异常	肝实质回声稍粗
6.22	9.00	9.83	2.36	1.36	异常	1、脂肪肝声像；2、
4.76	6.79	2.14	4.09	1.72	异常	1、脂肪肝声像；2、
5.85	5.67	1.61	3.07	1.62	异常	脂肪肝声像
6.52	4.01	2.68	1.72	1.57	异常	脂肪肝声像；胆囊内
6.61	7.09	1.66	4.44	1.71	异常	肝囊肿
5.23	6.89	1.60	3.89	1.70	异常	肝内囊性回声（考虑
5.46	4.33	0.88	2.60	1.00	异常	脂肪肝声像
4.77	5.17	1.65	2.28	1.92	异常	胆囊内异常回声，请
5.99	6.39	0.84	3.94	1.81		
5.59	6.93	3.40	3.06	1.44	异常	脂肪肝声像
4.85	3.92	1.61	2.04	1.32	未见异常	
5.81	5.01	1.40	2.74	1.38		
6.03	5.97	1.18	3.26	1.77	异常	脂肪肝声像
5.40	5.21	7.73	1.20	1.02	未见异常	
15.80	5.59	4.39	2.85	1.12	未见异常	
4.39	3.95	0.79	1.95	1.50		
5.04	6.08	3.52	3.08	1.58	异常	脂肪肝声像
5.02	5.13	1.59	2.82	1.39	异常	轻度脂肪肝声像

4.74	5.81	2.44	3.20	1.38	异常	轻度脂肪肝声像
5.74	5.70	7.85	1.44	1.20	异常	脂肪肝声像。
5.76	6.28	1.90	3.87	1.46	异常	腹部B超；脂肪肝声[illegible]
5.14	5.57	1.84	2.92	1.50	异常	脂肪肝声像，胆囊内
6.35	5.20	3.74	2.67	1.34	异常	脂肪肝声像，胆囊结
6.39	3.74	1.28	1.86	1.43	异常	轻度脂肪肝声像
7.98	6.24	1.98	3.86	1.37	异常	1、脂肪肝声像；2、
6.39	7.63	2.60	4.50	1.77	异常	脂肪肝声像
4.94	4.97	1.71	2.18	1.87	未见异常	
4.76	4.89	0.43	2.52	1.57	未见异常	
4.64	4.85	1.80	2.44	1.37	异常	轻度脂肪肝声像
4.64	5.83	0.79	3.08	1.90	异常	肝囊肿
5.93	5.18	1.10	2.93	1.44	异常	轻度脂肪肝声像
5.70	4.89	1.74	2.54	1.57	异常	1、脂肪肝声像、2、
5.34	4.50	0.99	2.43	1.46	异常	1、脂肪肝声像2、肝
6.19	7.55	1.03	3.73	2.54	异常	胆囊息肉样病变
5.06	5.12	5.99	1.60	1.22	异常	轻度脂肪肝声像
5.40	4.60	2.10	2.63	1.18	异常	1、脂肪肝声像 2、[illegible]
4.83	6.41	1.14	3.66	1.60	未见异常	
4.57	6.13	1.57	3.67	1.60	异常	肝囊肿
5.11	6.94	2.24	4.07	1.56	异常	轻度脂肪肝声像、肝
4.51	5.30	1.27	2.99	1.55	未见异常	
4.95	5.89	3.83	2.53	1.63	异常	1、肝囊肿2、 肝内[illegible]
16.70	4.89	2.00	2.59	1.53	异常	脂肪肝声像
5.27	5.96	3.13	3.00	1.26	未见异常	
5.15	6.55	1.27	4.00	1.43	异常	脂肪肝声像、胆囊结
4.95	6.80	1.72	4.60	1.74	未见异常	
5.07	6.58	1.53	3.69	1.64	异常	轻度脂肪肝
6.82	7.67	1.79	4.51	1.74	异常	脂肪肝声像
5.48	8.38	1.58	4.74	2.11	未见异常	
11.80	6.23	1.78	3.84	1.48	异常	1.脂肪肝声像2.胆囊
6.70	8.12	1.59	4.68	1.86	未见异常	
5.65	5.70	2.08	3.27	1.42	异常	腹部B超；1.脂肪肝[illegible]
5.38	5.50	3.15	2.81	1.61	异常	脂肪肝声像
5.55	5.18	1.02	2.67	1.65	异常	腹部B超；胆囊内强[illegible]
9.27	7.28	2.13	4.27	1.84	异常	脂肪肝声像
5.12	6.01	0.98	3.00	1.92	未见异常	
10.36	5.97	2.69	3.23	1.37	未见异常	
4.94	5.45	1.55	2.47	1.84	未见异常	
4.82	5.55	3.91	2.71	1.59		
5.28	6.24	2.19	3.49	1.49	异常	胆囊内强回声（考虑
5.05	6.67	2.43	3.48	1.84	异常	轻度脂肪肝声像
5.08	6.69	0.78	3.72	2.01	未见异常	
5.02	7.75	1.53	4.75	2.08	未见异常	
5.42	3.89	0.68	1.88	1.24	未见异常	
10.18	4.08	2.11	2.08	1.34	异常	1、脂肪肝声像；2、
5.39	6.04	1.39	3.14	1.79	异常	肝内多发囊性回声（
4.32	5.91	0.83	2.82	2.05	未见异常	
5.93	5.44	1.75	3.01	1.51	异常	1. 脂肪肝声像；2 .
6.48	6.40	1.22	3.98	1.57	未见异常	
5.53	6.22	1.22	3.56	1.59	异常	轻度脂肪肝声像

4.87	5.83	5.50	2.07	1.33	异常	腹部B超；胆囊切除
4.76	4.90	2.04	2.62	1.50	未见异常	
4.69	5.80	1.30	3.45	1.61	异常	轻度脂肪肝声像
5.28	4.18	0.90	1.99	1.59	异常	肝囊肿
4.92	6.22	1.92	3.78	1.64	异常	脂肪肝声像
6.23	6.68	1.69	3.98	1.95	异常	脂肪肝声像，肝囊肿
5.46	6.24	1.35	2.95	2.04	异常	脂肪肝声像
4.99	5.69	3.59	2.74	1.26	未见异常	
5.23	4.99	0.69	2.85	1.76	异常	胆囊结石
5.22	5.91	1.28	3.57	1.47	未见异常	
6.97	5.86	1.58	3.27	1.91		
4.48	3.40	1.71	1.23	1.57	异常	轻度脂肪肝声像。
5.30	5.84	0.96	3.36	1.39	异常	1.轻度脂肪肝声像
4.93	5.85	1.42	2.98	2.03	异常	肝实质回声稍粗
4.47	5.51	1.81	2.73	1.60		
5.06	5.11	1.49	2.10	2.05	异常	1.胆囊切除术后声像
5.32	6.14	0.77	2.84	2.33	未见异常	
6.60	5.57	0.80	3.43	1.46	异常	脂肪肝声像，肝内强
6.28	5.96	3.51	3.15	1.48	异常	轻度脂肪肝声像
8.36	6.35	2.89	3.47	1.67	异常	轻度脂肪肝声像
7.03	4.40	1.46	2.40	1.39		
4.92	5.55	1.60	3.03	1.67	未见异常	
4.59	3.42	0.82	1.38	1.43		
4.95	6.31	1.46	3.44	1.90	异常	肝实质回声稍粗
5.90	5.51	1.60	2.43	1.95	未见异常	
8.99	4.59	1.74	2.16	1.64	异常	胆囊切除术后。
5.22	5.94	0.92	3.51	1.66	异常	肝囊肿；脾内点状强
7.46	7.25	2.75	4.45	1.54	异常	轻度脂肪肝声像
5.28	4.85	1.98	2.59	1.63	异常	脂肪肝声像
5.78	7.37	2.64	4.28	1.72	异常	轻度脂肪肝声像
5.17	6.26	3.76	3.10	1.66	异常	轻度脂肪肝声像。
5.38	4.48	1.27	2.26	1.47	异常	脂肪肝声像、肝内囊
4.85	7.11	2.19	4.14	1.61	异常	轻度脂肪肝声像
5.22	5.48	1.01	2.93	1.90	异常	1、肝内稍强回声小
6.11	5.16	0.94	3.00	1.58	异常	脂肪肝声像
4.46	5.45	1.31	3.13	1.55		
16.69	7.21	6.40	2.54	1.67		
7.07	6.39	2.25	3.08	1.84	异常	轻度脂肪肝声像。
7.08	6.20	3.46	3.28	1.48	异常	轻度脂肪肝声像
6.50	5.37	2.00	2.97	1.28	未见异常	
5.12	5.38	1.03	2.99	1.54	未见异常	
4.65	4.34	1.48	2.10	1.48	未见异常	
6.67	5.33	2.18	2.86	1.52	异常	肝内囊性回声（考虑
4.91	6.17	2.74	3.46	1.47	异常	脂肪肝声像。肝内多
5.02	6.12	0.74	2.93	2.34	异常	肝囊肿
4.63	5.95	1.53	3.36	1.63	异常	轻度脂肪肝声像
5.60	6.53	2.67	3.74	1.27	异常	脂肪肝、肝囊肿？
5.03	5.71	0.95	3.34	1.57	未见异常	
6.01	5.00	0.99	2.51	1.76	异常	肝囊肿；
6.73	4.22	1.20	2.33	1.38	异常	轻度脂肪肝声像
4.63	7.22	2.49	3.78	1.91	未见异常	

6.74	4.43	1.81	1.47	1.97	异常	肝内囊性回声（考虑
5.47	4.76	1.20	2.58	1.61	未见异常	
5.62	5.87	3.07	3.17	1.61	未见异常	
4.75	5.79	1.02	3.40	1.61	异常	胆囊切除术后声像改
7.50	6.31	0.81	3.23	2.29	异常	1、肝实质回声稍粗；
5.22	3.09	3.09	4.28	1.57	异常	脂肪肝声像
5.58	5.38	1.56	3.25	1.55	异常	胆囊泥沙样结石？
5.12	6.80	2.08	4.03	1.60	异常	脂肪肝声像
4.44	5.83	1.27	3.42	1.55	异常	胆囊结石
5.21	5.79	2.11	3.15	1.51	异常	脂肪肝声像
4.91	5.30	0.86	3.26	1.33	异常	肝囊肿
5.89	4.66	2.00	2.56	1.29	未见异常	
5.94	6.49	2.96	3.40	1.61	未见异常	
5.10	5.12	0.96	2.87	1.40	异常	腹部B超：胆囊内强[illegible]
4.88	5.38	2.49	3.04	1.34	异常	1、肝内强回声斑：[illegible]
5.10	6.68	0.60	3.29	2.13	异常	胆多囊性回声（考虑
5.19	4.79	1.93	2.21	1.51	异常	轻度脂肪肝声像、肝
12.42	6.42	7.43	2.00	1.35	异常	脂肪肝声像
5.25	3.75	1.34	1.78	1.44	未见异常	
23.60	7.23	3.26	3.97	5.74		
5.56	7.92	11.55	1.65	1.37	异常	脂肪肝
4.70	5.23	0.87	3.11	1.54	异常	1、胆囊结石。
5.22	4.50	2.04	2.23	1.54	异常	肝实质回声稍粗
4.96	6.06	2.90	3.18	1.58	未见异常	
5.40	5.52	1.04	3.25	1.56	异常	胆囊强回声，结石？
4.93	4.28	0.79	2.01	1.45	未见异常	
4.96	4.67	0.58	1.96	2.02		
8.03	8.39	3.78	4.80	1.82	异常	胆囊内强回声（考虑
5.34	7.77	2.72	4.23	1.56	异常	肝内强回声斑：考虑
4.91	6.10	1.62	3.27	1.85		
5.37	6.36	0.68	3.55	1.93	未见异常	
4.21	6.71	0.79	3.41	2.36	未见异常	
5.23	4.85	1.73	2.58	1.53	未见异常	
9.09	5.37	1.23	3.07	1.59	异常	所示范围内脂肪肝声
5.04	5.02	1.56	3.02	1.31	异常	脂肪肝声像，肝囊肿
6.54	4.71	2.47	2.34	1.39		
6.44	6.08	2.04	2.97	1.89	异常	脂肪肝声像
6.12	5.84	1.45	3.75	1.40	异常	1、脂肪肝声像；2、
5.14	5.14	2.12	2.73	1.67	异常	脂肪肝声像。
4.59	8.10	1.58	4.60	2.07	异常	轻度脂肪肝声像；胆
5.26	4.88	4.13	2.03	1.36		
4.71	5.89	2.63	3.30	1.69	异常	轻度脂肪肝声像
5.42	4.41	1.67	2.25	1.60	异常	轻度脂肪肝声像
6.90	5.02	1.02	2.55	1.74		
7.32	4.04	2.08	1.97	1.27	未见异常	
5.71	7.32	2.13	4.65	1.61	异常	脂肪肝声像
4.58	6.12	0.98	3.66	1.54	未见异常	
7.08	5.56	7.06	1.57	1.07	异常	脂肪肝声像　肝内[illegible]
4.82	6.39	1.21	4.05	1.55		
5.84	7.98	2.86	4.48	1.87	异常	脂肪肝声像
6.97	3.11	1.91	1.14	1.35		

4.96	3.56	1.44	1.90	1.12	未见异常	
4.79	9.04	1.13	5.54	2.14	异常	脂肪肝声像
6.41	7.09	1.27	4.27	1.90		
5.34	4.99	1.91	2.62	1.51	异常	1.脂肪肝声像，2.胆
7.75	5.86	1.38	3.49	1.52	异常	1、脂肪肝声像2、肝
5.35	6.74	1.41	4.38	1.43	异常	轻度脂肪肝声像；肝
4.86	5.55	0.81	3.38	1.52	异常	轻度脂肪肝声像
5.26	5.46	1.11	3.50	1.29	异常	肝实质回声稍粗
9.90	4.84	1.56	2.64	1.53		
4.75	5.42	1.97	2.82	1.69	未见异常	
6.09	9.35	4.35	5.09	1.71	异常	脂肪肝声像
6.18	6.21	8.44	1.15	0.91		
5.81	6.29	1.08	3.47	1.83		
4.63	6.48	2.21	3.69	1.71	异常	肝实质回声稍粗
5.16	6.37	1.42	3.91	1.47	异常	肝内囊性回声（考虑
6.84	7.94	3.89	4.26	1.65	异常	脂肪肝声像
4.66	4.14	1.51	2.21	3.37	未见异常	
5.79	5.80	2.30	3.13	1.58	未见异常	
8.46	4.70	4.45	2.01	1.13	异常	脂肪肝声像
5.56	6.62	2.32	3.61	1.63	异常	腹部B超，胆囊结石
5.13	5.75	1.04	2.56	2.33	未见异常	
5.68	7.38	1.69	4.46	1.63	异常	肝实质回声稍粗
5.18	3.95	6.45	1.12	0.86	异常	轻度脂肪肝声像、胆
4.88	6.21	0.96	2.91	2.36	未见异常	
5.52	7.93	2.70	4.95	1.96	异常	脂肪肝声像
6.17	4.66	1.16	2.35	1.63	异常	肝内强回声斑：考虑
5.17	3.75	0.89	1.25	2.06	未见异常	
6.52	5.82	2.13	3.35	1.52	异常	脂肪肝声像、肝囊肿
4.23	6.70	2.94	2.67	2.13	未见异常	
6.88	4.95	2.92	2.85	1.01		
4.70	5.41	0.54	2.52	1.95	异常	肝内囊性回声（考虑
4.77	5.94	0.55	3.10	2.15	异常	肝多发囊肿
6.34	7.31	2.56	4.03	1.59	未见异常	
5.10	7.43	0.94	3.69	2.20	未见异常	
4.59	4.58	1.39	2.43	1.50	异常	轻度脂肪肝声像
6.76	4.49	2.05	1.64	1.94	异常	脂肪肝声像
5.08	4.85	1.17	2.48	1.59	异常	肝右叶稍强回声结节
7.39	5.91	3.43	2.97	1.64	异常	脂肪肝声像
7.69	7.13	4.19	3.58	1.61	异常	1、脂肪肝声像；2、
6.84	5.80	0.93	2.61	2.23	未见异常	
5.00	0.83	4.42	2.28	1.46	未见异常	
5.92	5.39	0.94	2.91	1.75	异常	胆囊内强回声（考虑
5.83	5.29	2.27	2.60	1.80	未见异常	
10.76	6.92	0.90	3.20	2.13	未见异常	
4.81	5.95	1.82	3.14	1.83	异常	肝囊肿
4.89	7.81	2.65	4.43	1.71	异常	腹部B超；胆囊内强[illegible]
4.49	4.92	1.05	2.64	1.66	未见异常	
5.80	4.70	2.55	2.37	1.32		
5.53	6.09	2.56	3.40	1.62	异常	1.肝内囊性回声（考
4.42	6.46	1.21	3.38	2.12	异常	腹部B超；1.脂肪肝[illegible]
7.50	4.75	1.64	2.50	1.42	异常	脂肪肝声像

4.71	5.87	0.85	3.31	1.68	未见异常	
6.61	5.27	1.70	3.02	1.27	异常	1.脂肪肝声像2.肝内
6.01	5.53	1.62	2.78	1.83	异常	脂肪肝声像
6.38	4.63	1.67	1.80	1.95	未见异常	
6.03	8.87	1.07	5.10	2.57	异常	轻度脂肪肝
4.91	5.30	1.14	2.91	1.80	未见异常	
4.46	5.05	0.88	2.67	1.74	未见异常	
5.58	5.95	1.34	3.26	1.72	未见异常	
7.92	6.05	1.86	3.63	1.32	异常	脂肪肝声像
8.10	6.05	2.00	3.47	1.70	异常	1.胆囊切除术后声像
4.42	4.68	1.38	1.93	1.98		
4.57	4.13	0.57	1.56	2.05	未见异常	
7.49	8.98	6.51	2.41	1.86	异常	1、脂肪肝声像；2、
6.01	6.48	1.28	3.01	2.27	未见异常	
6.92	6.53	2.42	3.49	1.63	异常	脂肪肝声像
5.22	6.03	2.65	2.93	4.16	异常	脂肪肝声像
5.51	6.43	1.40	3.66	1.78		
4.87	5.77	1.02	3.36	1.62	未见异常	
7.27	5.75	1.36	3.66	1.45	异常	脂肪肝声像
5.03	5.01	1.49	2.46	1.94	未见异常	
5.22	6.67	1.32	3.62	1.85	未见异常	
4.91	4.21	0.97	1.40	2.23	异常	1、肝实质回声稍粗；
5.63	5.07	1.35	2.84	1.36	未见异常	
4.53	6.41	3.18	3.34	1.73	未见异常	
4.81	4.98	1.28	2.28	2.00	未见异常	
4.79	4.09	1.42	1.42	1.97		
5.06	3.73	1.37	1.36	1.89	异常	肝内强回声斑（考虑
6.01	7.00	3.48	3.12	1.95		
4.83	5.54	1.53	2.98	1.45	异常	腹部B超：1.轻度脂肪
10.43	4.87	1.31	2.76	1.55	异常	1、轻度脂肪肝声像；
5.51	5.35	1.82	3.05	1.33	未见异常	
5.28	4.46	0.68	1.60	2.21	异常	腹部B超；1.胆囊切
5.39	8.21	2.48	5.04	1.70	异常	轻度脂肪肝声像
5.97	7.28	3.00	3.91	1.68		
5.26	5.64	0.47	3.20	1.96	异常	肝实质回声稍粗
4.67	5.91	0.96	2.96	2.06	未见异常	
4.79	7.28	1.76	4.34	1.91	未见异常	
4.98	5.23	0.75	2.58	2.02	异常	肝囊肿
3.94	5.71	2.53	2.88	1.70	未见异常	
5.74	7.43	2.93	4.01	1.73	异常	1.轻度脂肪肝声像 2
7.42	4.68	1.27	2.67	1.39		
4.99	5.64	1.56	3.12	1.68	异常	1.肝囊肿2.胆囊多发
6.49	6.05	2.04	3.58	1.49	异常	脂肪肝声像
5.15	7.79	7.24	2.11	1.47		
4.47	6.65	3.05	3.60	1.57	未见异常	
5.20	6.94	1.68	3.92	1.97	异常	轻度脂肪肝声像、肝
6.45	5.36	1.69	2.82	1.51		
4.53	5.33	1.25	2.52	2.09	未见异常	
6.46	7.64	2.57	4.54	1.91	异常	1、脂肪肝声像；2、
5.84	8.77	2.17	5.45	1.54	异常	胆囊内强回声（考虑
6.55	7.53	1.81	4.06	2.31	异常	脂肪肝声像

4.60	5.68	4.67	2.37	1.53	未见异常	
8.36	6.47	1.42	3.73	1.77	异常	1、脂肪肝声像，2、
6.47	6.23	3.42	3.32	1.37	未见异常	
8.09	4.43	1.30	2.71	1.15	异常	脂肪肝声像
6.31	3.43	0.97	1.48	1.13		
5.44	5.93	1.78	2.99	2.01	异常	胆囊切除术后、胆总
5.21	5.82	2.23	3.01	1.77	未见异常	
11.04	6.43	3.25	3.59	1.37	异常	1、脂肪肝声像
4.79	5.65	1.28	3.03	1.69	未见异常	
4.41	4.62	1.25	2.11	1.83	异常	1、肝实质回声稍粗2
5.73	2.96	1.07	1.27	1.19		
7.48	6.20	1.97	3.27	1.59	异常	轻度脂肪肝声像
5.05	4.88	1.33	2.22	1.92	异常	1.肝实质回声稍粗2.
4.64	6.12	1.65	3.05	1.94	未见异常	
6.72	2.30	0.66	1.05	0.93	未见异常	
4.76	6.68	0.80	3.70	2.00	异常	胆囊强回声团，考虑
8.59	5.27	2.09	2.87	1.53	未见异常	
4.42	3.83	0.79	1.82	1.58		
4.42	6.06	1.21	3.69	1.58	未见异常	
6.42	6.84	2.42	4.06	1.32		
4.54	6.06	0.86	2.80	2.28	未见异常	
6.74	5.70	1.78	2.57	2.06	未见异常	
4.84	4.97	1.16	2.45	1.74	未见异常	
20.76	4.18	2.37	2.24	1.18	未见异常	
4.90	6.08	2.55	3.26	1.63	异常	肝胆胰彩超：肝囊肿
6.07	7.17	1.61	3.98	1.83	未见异常	
5.52	5.30	2.30	2.87	1.23	异常	1、脂肪肝声像；2、
5.79	7.20	1.80	4.24	1.95	异常	1、脂肪肝声像；2、
6.93	6.82	1.22	3.66	2.21	未见异常	
5.86	5.95	0.98	2.83	2.09	未见异常	
4.26	4.16	3.04	1.96	1.28		
4.77	5.71	1.30	2.90	1.74	未见异常	
4.75	7.12	1.92	3.86	2.14	未见异常	
5.81	6.48	0.94	3.58	2.01	未见异常	
5.42	4.64	3.29	2.24	1.19	未见异常	
5.35	5.44	2.32	2.70	1.68	异常	轻度脂肪肝声像
4.70	4.65	1.47	2.53	1.40	未见异常	
5.01	6.13	2.56	3.10	1.71	异常	脂肪肝声像
5.65	3.46	3.24	1.07	1.42	异常	脂肪肝声像；
4.98	4.84	2.48	2.51	1.32		
7.92	6.07	4.41	2.73	1.51		
5.34	6.05	1.13	3.69	1.67	未见异常	
6.42	5.58	1.84	3.19	1.51	未见异常	
4.57	3.29	0.61	1.39	1.51	未见异常	
8.28	4.58	1.10	2.53	1.42	异常	肝胆胰彩超：轻度脂
4.76	4.20	0.97	2.38	1.35	异常	1、胆囊内低回声区，
4.49	5.11	1.37	2.45	1.90	未见异常	
7.79	7.82	6.25	3.45	1.59	异常	1脂肪肝声像2胆囊结
5.67	6.44	0.68	3.48	1.92	未见异常	
5.30	5.33	1.83	2.93	1.44	未见异常	
6.00	4.43	0.91	1.89	1.95	异常	脂肪肝声像。

4.91	5.05	0.90	2.44	1.68	异常	胆囊息肉样病变
4.96	6.64	1.35	3.97	1.86	未见异常	
7.82	7.37	3.98	3.95	1.78	异常	1、脂肪肝声像；2、
5.52	9.16	6.30	3.59	1.86	未见异常	
19.68	6.28	1.57	3.67	1.46	未见异常	
4.67	5.75	1.15	2.98	1.86		
4.37	7.40	1.71	3.96	2.03	异常	脂肪肝声像；
6.33	5.64	2.35	3.10	1.49	异常	脂肪肝声像
7.39	3.54	1.96	1.83	1.13	异常	脂肪肝声像
6.73	5.80	1.49	3.39	1.72	异常	1、肝囊肿
6.19	7.02	2.62	4.03	1.71	异常	腹部B超：肝囊肿
13.77	4.05	2.05	2.04	1.27	异常	轻度脂肪肝声像；胆
4.88	8.51	11.96	1.60	1.08	异常	脂肪肝声像
5.14	5.09	1.83	2.78	1.19	异常	1轻度脂肪肝声像 2
6.09	6.52	3.90	3.22	1.78		
4.97	5.49	1.29	3.32	1.62	异常	1.胆囊结石
4.95	5.56	0.92	2.38	2.11	异常	1肝实质回声增粗；2
4.89	4.35	1.22	2.10	1.62	未见异常	
5.21	5.21	4.91	2.03	1.33	异常	脂肪肝声像
5.41	6.78	3.19	3.46	1.75	未见异常	
5.14	6.05	1.54	3.24	1.91	异常	肝内稍高回声结节，
6.16	6.15	3.64	3.19	1.34	异常	1、肝内回声，不均[illegible]
6.45	6.13	1.15	4.08	1.34	异常	脂肪肝声像（肝右叶
5.10	5.53	1.70	2.91	1.56	异常	轻度脂肪肝声像。
4.70	5.54	2.55	2.38	1.79	异常	轻度脂肪肝声像
4.63	5.64	0.82	2.96	1.87	未见异常	
5.24	5.36	1.12	3.03	1.49	异常	肝内异常回声，不均[illegible]
5.19	5.55	1.14	2.91	1.81	异常	腹部B超；胆囊稍强[illegible]
15.53	5.95	1.26	2.76	2.07		
4.95	3.59	0.62	1.50	1.47	未见异常	
5.40	1.89	1.22	2.61	1.52	异常	脂肪肝声像
5.56	4.24	3.06	1.96	1.51	异常	轻度脂肪肝声像
5.33	5.03	4.56	2.08	1.28	异常	轻度脂肝肝声像;肝[illegible]
5.99	4.72	3.75	2.18	1.33	未见异常	
4.38	6.86	2.10	3.70	1.61	异常	胆囊腔内强回声团，
5.39	5.31	1.36	2.66	1.93	未见异常	
5.56	4.95	2.09	2.60	1.52	异常	脂肪肝声像
5.79	4.27	0.86	2.32	1.32		
4.27	5.69	0.79	3.49	1.69	未见异常	
5.09	6.31	0.80	3.80	1.65	未见异常	
5.25	4.97	0.61	2.84	1.52	异常	肝胆胰彩超：1、肝[illegible]
4.70	4.96	1.04	1.30	2.75	异常	胆囊结石
4.81	3.99	0.99	1.74	1.66		
5.25	4.24	1.32	1.51	2.00	未见异常	
12.51	6.62	3.40	3.49	1.66	异常	轻度脂肪肝声像。
4.90	6.99	1.84	3.73	1.89	异常	轻度脂肪肝声像、肝
5.26	6.76	2.43	3.53	1.62	未见异常	
5.56	6.44	1.83	4.03	1.57	异常	脂肪肝声像
8.20	4.74	2.34	1.38	2.23	异常	胆囊结石
6.08	5.18	0.81	2.69	1.68	异常	脂肪肝声像
5.01	4.84	0.96	2.29	1.90	未见异常	

5.22	6.98	1.22	4.15	1.51	异常	轻度脂肪肝声像
6.04	5.49	0.76	2.70	2.11		
7.03	4.26	1.10	2.31	1.32		
5.31	5.85	0.82	2.72	1.99	未见异常	
4.77	5.92	0.98	3.19	1.84	异常	轻度脂肪肝声像。
5.42	5.52	1.09	3.49	1.43		
3.98	6.52	4.41	3.03	1.29	未见异常	
4.99	6.13	1.65	3.14	2.08	异常	肝内小囊性回声（考
7.13	6.71	2.39	3.93	1.48	异常	1、脂肪肝声像；2、
5.79	4.99	0.97	2.79	1.55	异常	脂肪肝声像
12.73	4.82	1.74	2.38	1.47	异常	1脂肪肝声像2胆囊息
6.42	5.80	1.42	3.33	1.63		
5.12	4.39	1.90	2.03	1.68	未见异常	
4.76	8.63	2.21	4.64	2.45	异常	腹部B超：轻度脂肪[illegible]
5.58	7.72	2.53	4.47	2.16		
5.69	4.23	1.68	2.40	1.05	异常	脂肪肝声像
4.96	3.93	1.39	1.95	1.30	未见异常	
7.41	4.20	1.69	1.89	1.38	异常	1.脂肪肝声像2.胆囊
6.93	5.71	2.19	3.18	1.65	异常	轻度脂肪肝声像
5.70	5.63	1.24	3.12	1.55	异常	腹部B超；脂肪肝声[illegible]
4.68	4.98	2.03	2.67	1.39	未见异常	
7.05	5.02	3.38	2.55	1.40	异常	脂肪肝声像
4.30	7.38	1.22	4.05	2.08	异常	脂肪肝声像
6.39	5.84	6.42	1.78	1.42	异常	轻度脂肪肝声像
13.73	4.11	0.86	1.74	1.85	未见异常	
4.77	4.98	0.89	2.57	1.68	未见异常	
4.94	4.85	2.32	2.63	1.33		
5.97	5.22	0.81	2.83	1.71	未见异常	
4.71	3.62	1.33	1.36	1.53	未见异常	
5.25	6.79	1.37	3.68	2.24	未见异常	
6.96	5.86	4.56	3.19	1.55	异常	脂肪肝声像
4.57	5.34	4.01	2.54	1.39	异常	1.脂肪肝声像 2.肝[illegible]
5.18	6.71	1.75	4.00	1.82		
14.98	9.41	1.97	4.49	2.57	异常	脂肪肝声像
5.23	4.45	1.21	1.59	1.94	未见异常	
4.67	3.98	1.29	1.90	1.45	异常	肝囊肿
6.30	2.55	0.94	0.82	1.20		
5.37	5.82	1.52	3.39	1.59	异常	1、脂肪肝声像；2、
5.45	5.29	1.94	2.76	1.59	异常	轻度脂肪肝声像
4.98	6.74	2.24	4.15	1.39	未见异常	
5.98	5.31	2.62	2.87	4.15		
7.02	6.33	1.26	3.48	1.81	异常	肝实质回声稍粗
6.42	5.53	0.87	2.31	2.45	异常	轻度脂肪肝声像
4.59	4.68	1.37	2.62	1.37		
5.69	8.80	19.17	1.45	1.12	异常	脂肪肝声像
12.76	3.00	1.03	1.11	1.33	未见异常	
5.02	5.14	1.07	2.52	1.81	未见异常	
4.60	5.70	1.02	2.75	2.11	异常	肝实质回声稍粗
5.17	5.06	1.53	2.32	1.71	异常	1 肝囊肿，2 肝内强
15.55	3.97	1.39	1.97	1.33	未见异常	
4.69	5.27	1.33	2.63	1.79	未见异常	

5.67	4.52	1.33	2.66	1.28	未见异常	
6.53	4.40	0.85	2.66	1.31	未见异常	
5.60	6.88	2.71	4.11	1.70	未见异常	
5.06	6.21	4.26	2.81	1.72	异常	轻度脂肪肝声像
5.34	6.29	1.68	3.69	1.76	异常	脂肪肝声像
5.02	5.27	3.07	2.82	1.28	异常	胆囊内强回声(考虑纟
6.23	6.87	2.10	3.85	1.58	异常	1、胆囊多发结石;
4.84	6.29	1.44	3.89	1.63	未见异常	
4.28	4.77	2.08	2.69	1.41		
5.39	6.54	1.89	3.87	1.68	异常	脂肪肝声像。
5.40	6.88	6.80	2.33	1.20	异常	脂肪肝声像
5.53	7.12	2.24	4.29	1.84		
4.51	5.99	1.11	2.95	2.30	未见异常	
4.96	6.14	1.29	2.87	2.30	未见异常	
10.14	5.99	1.30	3.60	1.61	异常	轻度脂肪肝声像。
4.20	6.17	1.75	3.26	1.59	未见异常	
5.18	7.08	3.33	4.30	1.35	异常	脂肪肝声像
5.19	6.28	5.84	2.13	1.69	异常	肝内似见稍低回音区
5.51	6.02	1.51	2.95	2.10	异常	肝实质回声稍粗
6.08	7.38	1.51	4.08	1.93	未见异常	
4.96	4.38	0.75	2.10	1.83	异常	肝实质回声稍粗
5.14	5.91	1.86	3.36	1.37	异常	轻度脂肪肝
6.62	7.18	1.26	3.68	2.18	未见异常	
4.91	5.88	0.91	3.62	1.54	未见异常	
8.15	5.65	1.43	2.68	2.12	异常	脂肪肝声像
4.92	5.28	0.83	2.83	1.51	异常	肝囊肿
5.91	5.84	1.31	3.26	1.56	未见异常	
5.06	5.65	3.25	2.81	1.36	异常	轻度脂肪肝声像
5.54	5.80	1.63	3.31	1.76	未见异常	
5.66	5.17	3.05	2.56	1.56	异常	脂肪肝声像
5.44	6.85	2.27	3.76	1.75	异常	脂肪肝声像
4.84	6.02	2.25	3.24	1.57		
5.09	5.24	1.73	2.79	1.63	未见异常	
5.95	4.27	1.12	2.17	1.23		
4.75	6.95	1.05	4.03	1.77	未见异常	
4.61	6.27	2.07	3.49	1.52	异常	胆囊内稍高回声结节
4.38	5.25	1.51	2.93	1.51	异常	肝囊肿
6.76	7.77	3.15	4.61	1.48	未见异常	
4.95	6.04	0.91	3.12	2.11	未见异常	
6.21	4.61	2.76	2.03	1.65	未见异常	
4.75	5.28	0.71	2.14	2.23	异常	肝内囊性回声（考虑
5.07	4.84	1.22	2.44	1.67	未见异常	
5.22	6.20	1.43	3.01	1.95	未见异常	
6.02	7.96	1.17	4.80	1.69	异常	脂肪肝，胆囊内强回
5.80	4.73	0.79	2.61	1.57	异常	轻度脂肪肝声像；胆
4.49	5.67	1.47	3.01	1.45	异常	轻度脂肪肝、肝囊肿
4.65	6.30	1.42	3.56	1.39	未见异常	
4.82	7.04	9.33	1.41	1.06		
6.72	5.25	1.92	2.39	1.97	异常	脂肪肝声像
5.96	5.81	1.73	3.48	1.56	异常	胆囊结石
5.07	5.78	1.10	3.52	1.51	异常	1、轻度脂肪肝声像

5.09	5.87	1.21	2.99	1.72	未见异常	
6.18	6.75	1.79	3.83	1.79	异常	胆囊多发息肉样病变
9.43	6.12	1.62	3.59	1.82	异常	1、脂肪肝声像 2、月
5.72	3.16	1.51	1.48	1.18		
6.17	4.75	0.42	2.37	1.66	异常	肝实质回声增粗；肝
5.98	3.01	1.45	1.26	1.29		
4.58	3.64	0.83	1.50	1.39		
5.56	5.98	1.84	3.55	1.49	异常	轻度脂肪肝，胆囊息
4.98	3.97	4.44	1.36	1.25	异常	胆囊结石
9.09	6.14	1.44	3.15	1.73	异常	脂肪肝声像
4.80	7.70	1.32	4.63	1.97	未见异常	
4.24	6.74	1.79	3.74	1.65		
4.39	5.96	2.42	2.84	1.73	异常	脂肪肝声像
6.44	3.82	3.16	1.72	1.34	异常	肝高回声结节，性质
6.91	4.45	2.26	2.10	1.39	异常	脂肪肝声像
5.75	5.01	3.02	2.15	1.58	异常	脂肪肝声像
7.15	4.38	2.16	2.15	1.44	异常	肝囊内强回声（考虑
4.88	6.16	0.78	2.71	2.15	未见异常	
4.90	5.05	3.95	2.18	1.36	异常	脂肪肝声像
4.69	7.40	1.96	4.06	2.00	未见异常	
5.67	5.50	3.57	2.79	1.24	异常	轻度脂肪肝声像
10.18	6.29	1.32	2.98	2.07	异常	脂肪肝声像
4.96	6.91	2.46	3.52	1.67	未见异常	
9.79	5.11	2.08	2.72	1.49	异常	轻度脂肪肝声像
6.40	8.34	6.08	3.64	1.19	异常	肝左叶稍高回声结节
6.41	5.67	2.95	3.05	1.26	异常	脂肪肝声像
3.72	6.38	1.14	3.52	1.79	异常	肝内局部胆管稍扩张
8.75	6.10	1.20	3.53	1.38	异常	脂肪肝声像
4.60	4.61	2.95	2.20	1.28	未见异常	
9.22	6.08	1.49	3.46	1.39	异常	胆囊内强回声（考虑
5.15	7.18	1.51	3.87	2.01	异常	1、肝实质内稍高回声
5.78	6.97	2.72	3.61	1.61	异常	肝囊肿
4.74	4.80	2.82	2.33	1.17	未见异常	
6.49	5.88	1.12	2.55	2.08	未见异常	
4.56	6.60	2.20	3.48	1.56	异常	轻度脂肪肝声像
3.85	5.14	2.98	2.33	1.56	未见异常	
5.72	4.77	4.52	1.76	1.53	异常	脂肪肝声像
4.96	7.58	2.35	4.05	1.85	未见异常	
4.97	6.43	0.88	3.79	1.44	未见异常	
4.69	7.14	2.40	4.16	1.32	未见异常	
5.60	6.32	1.03	3.51	1.63	未见异常	
8.65	5.25	0.88	2.71	1.48	未见异常	
5.61	6.78	2.23	3.92	1.33	未见异常	
4.70	5.81	1.30	2.88	1.91	异常	胆囊内稍强回声区：
4.87	4.63	1.20	1.70	1.84	未见异常	
4.95	6.78	2.77	3.75	1.42	异常	肝囊肿，胆囊结石
5.76	6.60	2.70	3.48	1.60	异常	脂肪肝声像，胆囊内
4.92	7.23	3.60	3.99	1.51	异常	1、脂肪肝声像；
4.26	3.33	1.32	1.64	0.71	异常	胆囊切除术后
4.85	3.90	0.54	1.59	1.55	未见异常	
5.50	7.78	1.59	4.67	1.56	异常	1、肝囊肿；2、胆囊

4.53	6.20	3.40	2.93	1.55	异常	1肝多发囊肿。2胆囊
5.33	6.51	1.25	3.63	1.80	异常	1.脂肪肝升声像。2.
4.90	4.96	1.43	1.78	2.34	未见异常	
4.94	6.27	1.50	4.16	1.01	未见异常	
5.36	7.17	2.00	4.32	1.39	异常	脂肪肝声像；胆囊结
5.17	5.84	1.04	3.38	1.58	未见异常	
5.21	5.95	1.69	3.22	1.49	未见异常	
5.29	5.74	1.53	2.88	1.63	未见异常	
5.10	5.92	1.23	2.59	1.96	异常	胆囊息肉样病变
5.24	6.50	1.56	4.16	1.39	未见异常	
6.62	4.44	1.07	2.21	1.62	未见异常	
5.14	4.11	1.13	2.03	1.49	未见异常	
7.60	6.70	1.40	3.82	1.71	未见异常	
5.11	4.36	1.74	2.09	1.25	未见异常	
11.33	6.93	1.41	3.91	1.67		
4.71	5.65	1.88	3.07	1.53	未见异常	
6.89	6.62	2.35	3.64	1.42	异常	轻度脂肪肝声像
5.57	7.01	2.55	3.96	1.69	未见异常	
7.39	4.75	6.61	1.35	1.31	异常	轻度脂肪肝声像
5.44	4.87	0.96	2.13	1.82	未见异常	
4.75	5.94	1.70	3.17	1.62	未见异常	
4.25	5.85	1.68	3.37	1.40	异常	轻度脂肪肝声像
15.37	4.65	3.77	2.12	1.15	异常	胆囊腔内稍高回声点
4.61	6.56	0.74	3.54	1.81	异常	肝内强回声斑：考虑
5.26	4.27	2.19	1.98	1.34	未见异常	
6.12	5.88	0.92	2.80	1.86	异常	脾内囊性暗区，考虑
4.52	5.86	1.74	3.21	1.56	未见异常	
5.21	4.80	1.45	2.62	1.28	未见异常	
4.41	7.63	4.54	3.75	1.52	异常	脂肪肝声像
4.62	7.06	0.92	3.76	2.03	未见异常	
5.54	6.21	1.79	3.67	1.35	异常	脂肪肝
4.36	5.91	1.00	2.92	2.24	未见异常	
7.62	4.12	1.81	2.19	1.00	异常	轻度脂肪肝声像
4.77	3.45	0.89	1.53	1.35	未见异常	
4.54	7.33	1.76	3.62	1.98	未见异常	
5.02	5.27	1.71	3.04	1.54	异常	脾内强回声斑：考虑
7.33	7.30	1.76	4.30	1.93	异常	胆囊结石
5.44	6.09	2.00	3.38	1.68	未见异常	
5.59	6.57	4.40	2.93	1.62	异常	脂肪肝声像
4.81	4.88	0.77	2.25	1.55	异常	脂肪肝声像；
5.27	6.06	2.90	3.19	1.52	未见异常	
5.52	6.15	2.11	3.33	1.52	未见异常	
5.67	4.13	1.33	1.61	1.69	未见异常	
5.36	7.13	1.45	4.01	2.05	异常	肝囊肿
4.71	4.87	1.29	2.42	1.66	未见异常	
7.11	4.60	3.07	2.29	1.13	异常	脂肪肝声像
6.81	4.48	1.22	1.90	1.61	未见异常	
5.54	5.79	1.22	3.19	1.54	未见异常	
5.32	6.28	1.30	3.64	1.37	未见异常	
4.46	6.21	2.50	2.67	1.91	未见异常	
5.31	4.50	1.46	2.03	1.43	异常	脂肪肝声像

4.60	6.96	1.02	3.90	1.85	未见异常	
4.87	8.19	3.10	4.24	2.13	未见异常	
5.25	6.80	3.19	3.25	1.60	未见异常	
11.82	5.11	0.73	2.63	1.63	异常	轻度脂肪肝声像
4.86	5.26	1.10	2.80	1.57	未见异常	
7.18	6.09	2.00	3.32	1.40	异常	轻度脂肪肝声像
14.47	6.58	1.04	3.63	2.20	未见异常	
4.92	4.30	1.46	2.42	1.17	异常	胆囊内强回声（考虑
7.30	4.43	1.69	2.48	1.30	异常	轻度脂肪肝声像
4.69	4.85	0.92	2.70	1.48	未见异常	
5.17	4.91	0.78	1.97	2.00	异常	肝左叶稍高回声结节
6.22	6.43	2.59	3.64	1.63	异常	肝内强回声斑：考虑
5.27	3.98	1.31	2.15	1.08	异常	轻度脂肪肝声像
3.79	4.98	0.71	2.37	1.67	未见异常	
4.35	4.53	0.74	1.82	1.88	未见异常	
4.36	4.75	1.19	2.37	1.45	异常	肝内稍高回声，钙化
5.92	8.95	0.94	5.50	1.87	异常	轻度脂肪肝声像；
4.61	6.77	3.15	3.68	1.48	未见异常	
4.79	9.45	2.83	5.12	2.14	未见异常	
21.42	4.28	3.42	1.96	0.96	异常	脂肪肝声像
5.75	4.06	1.52	1.85	1.38	异常	脂肪肝声像
6.40	4.65	2.86	2.37	1.20	异常	1.脂肪肝声像2.肝内
5.00	7.72	3.88	4.02	1.37	未见异常	
5.43	9.45	8.56	1.91	1.70	异常	轻度脂肪肝声像；
7.39	5.71	1.55	3.19	1.70		
4.96	4.89	1.44	2.41	1.67	未见异常	
4.46	3.66	0.71	1.45	1.52	异常	轻度脂肪肝声像
5.55	6.40	1.97	4.17	1.34	异常	肝实质内异常回声，
6.56	3.20	1.71	1.72	1.00		
5.02	4.64	0.60	1.91	1.95	未见异常	
4.80	4.70	0.86	2.29	1.79	未见异常	
5.21	6.11	1.52	3.35	1.97	异常	轻度脂肪肝声像；
4.71	4.08	1.42	2.15	1.18	异常	脂肪肝声像
9.69	5.65	1.93	3.23	1.43	异常	脂肪肝声像。胆囊内
6.31	5.77	0.91	3.02	1.64	异常	胆结石
5.38	4.92	2.04	2.19	1.45	异常	轻度脂肪肝声像
4.64	6.44	2.72	3.89	1.39	异常	胆囊内强性回声（考
8.55	5.55	0.92	3.57	1.35	异常	轻度脂肪肝声像
5.51	5.86	2.87	3.24	1.36	异常	轻度脂肪肝声像。
5.39	5.66	3.12	3.04	1.21	未见异常	
4.16	5.25	0.66	2.68	1.38	未见异常	
5.75	3.89	3.65	1.58	1.05	异常	脾稍大
5.36	4.92	3.42	2.41	1.32	异常	脂肪肝声像；肝囊肿
4.99	4.82	3.32	2.29	1.21	异常	1.脂肪肝声像 2.肝[illegible]
5.51	6.38	1.27	3.66	1.67	异常	胆囊结石
4.58	5.65	1.71	3.18	1.39	未见异常	
5.13	6.17	1.75	3.37	1.72	异常	轻度脂肪肝声像；胆
5.18	6.21	2.37	3.74	1.29	异常	腹部B超：1、脂肪肝
5.91	5.17	2.65	2.57	1.59	异常	脂肪肝声像
6.16	5.37	1.44	3.04	1.51	异常	轻度脂肪肝声像
5.32	7.30	2.36	4.32	1.80	未见异常	

6.29	6.08	1.84	3.56	1.51	异常	脂肪肝声像
10.54	5.52	2.55	2.80	1.53	异常	脂肪肝声像；胆囊壁
5.81	5.09	2.95	2.43	1.40	异常	脂肪肝声像
5.63	6.80	0.81	3.98	1.68	未见异常	
5.87	6.58	1.95	3.91	1.53	异常	肝内强回声斑：考虑
5.49	3.36	0.66	1.68	1.16	异常	1轻度脂肪肝声像2 肝
5.59	7.51	3.22	4.03	1.85	异常	轻度脂肪肝声像。
6.82	4.61	4.17	1.85	1.50	异常	1、脂肪肝声像 2胆
4.95	4.05	2.81	1.81	1.25	异常	轻度脂肪肝声像
5.94	5.61	1.38	2.77	1.66	未见异常	
4.74	6.20	2.15	3.13	1.72	异常	肝囊肿
6.33	5.29	1.65	2.47	1.78	未见异常	
5.90	5.92	0.85	3.51	1.36	异常	脂肪肝声像
7.34	3.87	1.03	2.01	1.02	异常	轻度脂肪肝声像、胆
5.06	3.70	0.72	1.80	1.13	未见异常	
6.34	6.08	1.56	3.43	1.31	异常	轻度脂肪肝声像；胆
5.54	5.87	0.91	3.27	1.68	未见异常	
4.77	5.67	0.83	2.87	1.90	异常	胆囊腔内多发强回声
4.61	5.70	2.05	3.02	1.72	未见异常	
5.31	4.91	1.50	2.82	1.37	异常	轻度脂肪肝声像
6.51	5.42	5.39	1.81	1.26	异常	脂肪肝；肝内囊性回
4.94	5.98	1.31	3.00	1.96	异常	肝囊肿
7.21	7.92	6.89	2.46	1.53	异常	脂肪肝声像
5.02	5.02	1.69	2.44	1.51	未见异常	
5.69	5.09	2.12	2.55	1.75	未见异常	
4.70	5.84	1.12	3.32	1.44	异常	胆囊多发性结石
4.78	10.05	2.39	6.72	1.74	异常	脂肪肝声像。
6.89	5.18	1.35	2.77	1.50	异常	轻度脂肪肝，肝囊肿
7.49	5.06	1.04	2.49	1.52	异常	胆囊切除术后，胆总
5.04	5.96	4.16	2.54	1.51	未见异常	
5.11	9.56	2.21	5.44	2.04	异常	轻度脂肪肝
6.00	9.01	1.81	5.79	1.48	未见异常	
6.13	4.26	0.92	2.02	1.39	未见异常	
4.84	4.72	1.28	2.16	1.67	未见异常	
4.78	5.61	1.50	2.84	1.83	异常	肝囊肿
4.64	4.38	1.25	2.14	1.32	异常	1.轻度脂肪肝声像；
4.89	5.47	1.58	2.07	2.20	异常	肝内强回声斑，考虑
4.88	6.54	5.67	2.42	1.62	异常	轻度脂肪肝声像
4.61	4.62	1.33	2.77	1.11		
4.46	4.09	0.86	2.00	1.30	异常	脂肪肝声像，肝囊肿
7.89	5.89	3.45	2.58	1.65	异常	脂肪肝声像
12.43	4.36	1.52	2.03	1.52	异常	轻度脂肪肝声像
5.38	6.90	2.46	3.86	1.46	异常	肝囊肿
5.42	5.80	1.72	3.35	1.49	未见异常	
5.64	4.20	1.97	1.75	1.60	异常	脂肪肝声像
4.86	5.55	1.71	2.94	1.84	未见异常	
4.53	5.42	1.51	2.95	1.44	异常	肝左叶体积小、肝内
6.02	4.79	0.62	2.44	1.53	未见异常	
4.70	6.94	2.40	3.59	1.95	未见异常	
5.10	5.38	4.31	2.43	1.19	异常	脂肪肝声像,胆囊结石
4.39	7.63	1.98	4.18	1.59	异常	胆总管扩张、脾内强

6.72	2.77	0.78	1.11	1.15	未见异常	
15.03	4.74	1.32	2.26	1.63	异常	胆囊结石
4.57	5.35	1.23	2.69	1.63	异常	肝内囊性回声（考虑
4.59	6.09	1.09	3.15	1.81	未见异常	
5.53	7.23	1.01	3.97	1.80	异常	胆囊结石
7.30	6.42	0.83	3.52	1.67	未见异常	
5.60	6.51	0.98	3.23	1.84	未见异常	
6.18	5.68	2.16	2.73	1.57	异常	轻度脂肪肝声像
5.52	6.36	1.42	3.72	1.48	异常	脂肪肝声像
6.68	6.75	1.67	3.82	1.68	异常	腹部B超：.胆囊内强
5.04	5.07	1.42	2.79	1.09	异常	1.轻度脂肪肝 2.胆
5.46	6.66	2.37	3.63	1.49	异常	胆囊结石
8.01	3.47	1.61	1.48	1.46	异常	脂肪肝声像
6.19	4.45	1.33	1.90	1.64	异常	脂肪肝声像。
4.33	5.03	1.74	2.74	1.48	异常	肝囊肿声像
4.79	6.76	0.94	4.21	1.71	异常	胆囊结石
5.02	5.56	0.82	2.93	1.90	异常	肝囊肿
5.81	5.53	1.01	2.76	1.69	异常	1.轻度脂肪肝2.肝小
5.65	6.28	3.22	3.04	1.46	异常	轻度脂肪肝声像
5.25	10.27	18.13	2.31	1.25	异常	1、脂肪肝声像；2、
15.31	6.83	2.60	4.07	1.74	异常	腹部B超：肝内强回
5.36	6.68	1.37	3.69	1.84	未见异常	
16.62	5.23	1.83	2.53	1.44	异常	胆囊内强回声（考虑
4.69	3.92	4.94	1.27	1.07	未见异常	
5.14	4.51	0.91	2.08	1.92	未见异常	
4.44	5.20	2.55	2.63	1.56	异常	胆囊腔内稍强回声，
6.31	5.26	4.19	2.76	1.31	异常	轻度脂肪肝声像
5.46	6.90	2.05	4.04	1.57	未见异常	
6.38	8.32	7.17	2.63	1.73	异常	1、脂肪肝声像；2、
5.57	6.75	2.24	3.57	1.81	未见异常	
4.97	5.76	1.18	3.16	1.41	未见异常	
6.40	4.82	0.82	2.67	1.62	未见异常	
5.38	6.21	1.57	3.49	1.35	异常	轻度脂肪肝声像，肝
5.10	5.44	1.08	2.33	2.27	未见异常	
5.46	5.52	1.37	3.01	1.56	异常	胆囊息肉样病变
5.12	6.30	1.81	3.39	1.49	未见异常	
5.48	7.78	1.08	3.99	2.07	异常	肝囊肿
5.61	5.28	1.50	2.52	1.66	未见异常	
5.13	5.65	1.27	2.94	1.70	异常	脂肪肝声像
4.58	4.78	1.82	2.78	1.03	未见异常	
6.45	5.49	5.54	2.24	1.16	异常	脂肪肝
5.36	6.64	1.76	3.15	2.04	异常	胆囊内强回声
6.38	8.22	11.37	1.36	1.20	异常	肝胆脾胰B超：脂肪
4.38	5.50	2.76	2.80	1.34	异常	肝实质回声增粗
5.05	4.24	1.02	1.77	1.74	异常	轻度脂肪肝
5.59	4.60	2.10	2.58	1.06	异常	胆囊息肉病变
4.71	6.05	1.35	3.63	1.37	未见异常	
6.63	6.87	1.32	3.71	1.98	未见异常	
4.76	5.61	1.26	2.89	1.47	异常	胆囊窝强回声团（胆
9.74	7.59	2.00	4.18	1.79	异常	脂肪肝声像
5.36	5.98	2.62	3.36	1.63	异常	脂肪肝声像

5.48	5.97	1.39	3.36	1.69	异常	脂肪肝声像
7.00	5.40	1.30	3.07	1.61	异常	肝小囊肿
4.37	5.30	1.90	2.56	1.56	异常	轻度脂肪肝声像
5.58	5.72	2.48	2.54	1.76	异常	胆囊结石
5.60	4.54	1.78	2.37	1.28	异常	胆囊息肉样病变
4.82	5.98	1.48	2.98	1.67	未见异常	
6.11	4.63	1.32	1.81	1.75	未见异常	
4.78	5.47	1.76	2.97	1.49	异常	1.脂肪肝声像
5.91	6.79	3.62	3.42	1.70	异常	脂肪肝声像；胆囊内
5.33	5.81	2.37	3.18	1.15	未见异常	
5.44	6.59	1.73	3.77	1.43	未见异常	
4.81	4.54	1.10	1.52	2.17	未见异常	
4.97	5.19	1.47	2.45	1.85	异常	胆囊结石
5.15	4.40	7.80	1.85	1.61	未见异常	
4.86	6.48	1.64	4.06	1.51	异常	腹部B超：1、胆囊内
5.96	5.84	1.87	3.13	1.78	异常	1.脂肪肝声像 2.胆[illegible]
5.59	5.87	1.86	3.27	1.48	异常	轻度脂肪肝声像。
5.00	7.15	1.05	4.10	1.94	未见异常	
4.93	7.37	1.18	3.08	2.51	未见异常	
6.69	6.98	1.97	4.20	1.43	未见异常	
4.86	5.11	0.95	2.87	1.51	异常	肝内强回声斑（考虑
5.61	5.86	1.67	3.48	1.35	异常	轻度脂肪肝声像
8.96	5.43	2.41	2.90	1.30	异常	轻度脂肪肝声像
6.63	5.17	2.93	2.16	1.41	异常	1.实质回声稍增粗 2
5.02	6.42	1.55	3.44	1.64	未见异常	
6.14	3.75	1.09	1.97	1.27	异常	轻度脂肪肝声像
6.25	7.03	1.85	4.15	1.41	异常	胆内强回声团，考虑
7.66	6.89	2.46	3.91	1.56	异常	脂肪肝声像
4.58	5.75	1.18	3.33	1.48	未见异常	
5.45	4.77	1.66	2.22	1.42	未见异常	
4.53	6.34	3.91	2.77	1.54	异常	脂肪肝声像；胆囊结
5.22	5.04	3.56	2.25	1.52	异常	肝实质回声稍增粗
6.41	6.55	1.38	3.73	1.61	异常	胆囊息肉样变
6.75	6.15	2.46	3.17	1.60	未见异常	
5.56	5.74	2.13	3.02	1.68	异常	脂肪肝声像
4.88	5.39	3.88	2.52	1.38	异常	轻度脂肪肝声像
9.20	6.40	10.77	0.88	0.98		
4.51	5.77	1.67	3.11	1.51	未见异常	
5.24	7.99	4.09	3.68	1.84	未见异常	
5.76	4.84	3.43	2.46	1.14		
5.19	6.22	1.17	3.39	1.91	未见异常	
8.65	5.67	4.30	2.51	1.45	异常	1、脂肪肝声像2、胆
6.97	6.27	1.69	3.63	1.37	异常	肝内强回声斑，考虑
4.56	5.39	0.81	2.90	1.81	未见异常	
6.13	7.33	1.55	4.08	1.63	未见异常	
5.09	4.77	2.20	2.56	1.36	未见异常	
4.68	4.57	2.99	2.02	1.43	未见异常	
6.61	6.07	1.10	2.78	2.12	未见异常	
4.78	4.92	0.94	2.64	1.51	未见异常	
4.43	4.29	1.37	1.86	1.77	异常	胆囊区强回声团
4.96	5.81	1.31	3.14	1.51	异常	肝囊肿

6.82	5.55	2.84	2.97	1.27	异常	1.脂肪肝声像2.胆囊
7.55	5.40	1.26	2.81	1.65	未见异常	
5.39	7.30	1.60	4.33	1.88	异常	肝囊肿
4.77	4.13	2.12	1.97	1.19	异常	轻度脂肪肝声像；肝
8.51	6.10	0.84	3.19	1.63	未见异常	
5.05	4.72	5.53	1.62	1.07	异常	胆囊强回声团，可能
5.62	6.07	0.67	3.17	1.93	未见异常	
6.17	4.75	0.42	2.37	1.66	异常	1肝内强回声（考虑钅
6.07	7.00	2.94	3.92	1.45		
6.21	8.76	1.94	5.21	1.84	未见异常	
8.50	5.84	2.29	3.21	1.46	异常	1轻度脂肪肝声像2肝
4.62	5.49	2.66	3.19	1.34	未见异常	
5.80	7.36	2.31	4.26	1.48	未见异常	
5.23	6.70	1.37	3.42	1.90	异常	1.肝内强回声灶：考
4.70	5.59	1.05	2.92	1.66	未见异常	
14.70	4.86	1.35	2.38	1.80	异常	轻度脂肪肝声像
6.27	7.21	2.84	3.61	1.83	异常	脂肪肝声像
5.02	7.30	2.35	4.17	1.53	未见异常	
5.05	5.81	10.00	0.99	1.13	异常	轻度脂肪肝声像，肝
5.59	7.64	2.93	4.16	1.64	未见异常	
4.76	6.85	2.46	4.01	1.41	异常	脂肪肝声像，胆囊结
5.52	4.95	0.91	1.99	2.07	未见异常	
5.59	6.05	5.73	2.20	1.39	异常	轻度脂肪肝声像
4.49	5.52	1.28	3.15	1.58	未见异常	
4.19	6.31	1.20	3.28	1.91	未见异常	
4.89	5.77	1.93	3.01	1.80	未见异常	
4.94	9.17	2.07	5.80	1.66	异常	轻度脂肪肝
6.99	4.69	2.28	2.39	1.29	异常	脂肪肝声像
9.29	5.58	1.40	3.34	1.27	异常	脂肪肝声像，肝囊肿
5.40	6.81	0.74	3.46	2.37	异常	肝多发囊性回声(考［
4.60	4.83	0.97	2.39	1.73	异常	胆囊壁欠光滑，稍毛
5.74	5.01	0.62	2.36	1.84	异常	脂肪肝声像
4.61	6.03	1.11	3.90	1.94	未见异常	
4.84	8.05	1.57	4.61	2.03	异常	胆囊结石
5.82	5.08	1.01	2.64	1.70	异常	脂肪肝声像。
5.09	6.90	1.20	4.00	1.80	异常	肝实质回声稍增粗
5.77	7.23	1.82	4.07	1.78	未见异常	
6.74	6.26	4.31	3.01	1.58	异常	轻度脂肪肝
4.62	5.82	0.60	2.00	2.87	未见异常	
7.30	7.67	8.90	1.53	1.57	异常	轻度脂肪肝
5.03	5.42	1.23	2.97	1.50	未见异常	
5.32	6.35	3.50	2.89	1.69	未见异常	
4.21	3.87	1.38	1.77	1.36	异常	胆囊内强回声,考虑纟
4.01	6.96	0.92	3.91	2.19	异常	胆囊结石
5.16	4.83	4.06	1.84	1.56	未见异常	
8.31	4.17	1.61	2.05	1.28	异常	轻度脂肪肝
5.10	5.89	0.86	13.23	1.64	未见异常	
6.43	6.21	2.42	3.01	1.84	未见异常	
13.05	5.42	1.65	3.09	1.31	未见异常	
5.29	5.91	2.24	3.12	1.41	未见异常	
5.32	5.06	5.11	1.95	1.40	异常	轻度脂肪肝声像

5.68	8.54	0.93	5.24	2.07	未见异常	
8.37	5.61	0.88	2.88	1.65	未见异常	
5.29	4.71	0.99	2.05	1.85	未见异常	
4.83	10.64	1.25	5.99	2.70	异常	肝实质回声增粗
4.66	4.83	1.68	2.36	1.64	异常	肝内强回声斑，考虑
5.31	6.69	3.29	3.49	1.52	异常	腹部B超：1、脂肪肝
4.97	5.67	5.01	2.26	1.37	异常	轻度脂肪肝声像
5.17	5.71	3.09	2.90	1.21	异常	1 轻度脂肪肝；2 胆
6.36	6.33	6.51	2.05	1.32	异常	轻度脂肪肝
4.73	6.02	3.50	3.00	1.68	异常	轻度脂肪肝声像。胆
7.36	4.88	1.50	2.26	1.60	异常	脾内囊性回声，考虑
7.36	6.17	3.19	3.36	1.61	异常	脂肪肝声像
5.89	5.31	1.99	2.72	1.63	异常	脂肪肝声像,胆囊结
4.52	5.98	1.67	3.02	1.65	异常	胆囊结石
6.67	6.25	1.27	3.89	1.53	异常	腹部B超：1、脂肪肝
5.65	4.86	0.94	2.62	1.65	异常	脂肪肝声像
5.65	7.83	14.77	1.07	1.47	未见异常	
6.83	5.06	2.70	2.31	1.55	异常	轻度脂肪肝声像
8.34	6.55	2.12	3.46	1.64	未见异常	
11.07	4.66	5.42	1.50	1.21	异常	脂肪肝声像，胆囊多
4.34	5.45	1.26	2.34	1.80	异常	胆囊壁毛糙
5.44	5.41	3.60	2.45	1.68	未见异常	
8.26	4.27	4.53	1.63	1.08	异常	轻度脂肪肝声像，肝
5.57	4.66	1.08	2.44	1.32	未见异常	
5.05	5.99	1.89	3.03	1.64	未见异常	
10.75	7.56	4.41	3.97	1.38	异常	脂肪肝声像
4.91	5.60	1.95	2.62	1.80	未见异常	
4.67	6.51	1.01	3.52	2.07	异常	肝囊肿
4.45	4.06	2.93	1.71	1.46	异常	1.脂肪肝声像2.胆囊
5.93	3.91	5.02	1.36	1.12	异常	轻度脂肪肝声像
6.25	6.60	2.08	3.67	1.40	异常	胆囊息肉样病变
4.50	4.50	1.52	1.94	1.70	未见异常	
4.99	5.42	0.89	3.03	1.61	异常	腹部B超：1、轻度脂
5.63	7.67	1.29	4.41	1.68	未见异常	
4.82	5.87	1.41	3.17	1.45	未见异常	
4.84	7.09	0.76	3.85	2.20	未见异常	
4.88	3.83	1.75	1.72	1.34	未见异常	
4.84	5.00	1.41	2.54	1.60	未见异常	
4.33	6.20	0.77	2.60	2.33	未见异常	
5.45	7.48	2.34	2.83	2.22	未见异常	
4.65	5.94	1.01	2.88	2.01	异常	1、肝囊肿，2、胆囊
5.67	5.54	0.62	2.31	2.22	未见异常	
5.39	4.67	0.59	2.27	1.41		
4.75	3.97	1.28	1.38	1.72	未见异常	
7.22	5.95	8.38	1.28	1.01	异常	脂肪肝声像；胆囊结
6.06	5.26	3.66	2.26	1.41	异常	轻度脂肪肝声像
4.44	4.38	1.22	2.34	1.15	未见异常	
4.84	7.74	1.54	5.18	1.50	异常	胆囊内强回声（考虑
5.66	5.60	1.90	3.69	1.18	异常	肝左叶低回声，性质
6.45	5.97	1.41	3.61	1.44	异常	脂肪肝
5.17	4.55	0.95	1.91	1.77	异常	胆囊结石。

4.73	6.33	1.90	3.80	1.20	异常	脂肪肝声像
6.30	4.87	2.25	1.81	1.91	未见异常	
6.63	7.23	2.66	4.12	1.44	异常	轻度脂肪肝声像
5.82	5.96	2.52	3.10	1.50	异常	轻度脂肪肝
4.43	8.15	3.13	4.44	1.92	未见异常	
4.68	5.71	1.99	2.74	1.75	异常	胆囊壁稍强回声区
5.32	6.49	1.70	3.75	1.85	未见异常	
5.42	3.71	1.38	1.21	1.74	未见异常	
4.50	6.01	1.53	3.08	1.89	未见异常	
7.88	3.36	1.34	1.49	1.24	异常	脂肪肝声像
5.07	6.21	1.26	2.92	2.17	异常	轻度脂肪肝声像；肝
6.33	5.57	1.29	3.16	1.55		
9.96	4.13	2.20	2.21	1.21		
5.19	4.77	2.07	2.41	1.42	未见异常	
14.82	7.54	2.08	4.28	1.87	异常	轻度脂肪肝声像
5.27	7.12	3.60	3.94	1.40	未见异常	
5.19	6.20	0.80	3.93	1.42	异常	未排除胆囊结石
4.68	5.61	0.89	2.33	2.19	未见异常	
4.38	5.19	1.98	2.18	1.78	未见异常	
5.02	6.37	3.59	3.10	1.71	未见异常	
4.99	5.97	1.52	3.10	1.90	异常	肝小囊肿
5.06	4.77	0.70	2.63	1.44	未见异常	
7.37	5.04	2.20	2.83	1.13	异常	脂肪肝声像
9.25	4.71	1.82	2.51	1.38	异常	腹部B超异常：1.所
15.40	5.29	1.78	2.84	1.47	未见异常	
5.26	4.12	3.39	1.76	1.27	异常	轻度脂肪肝
4.88	7.25	1.60	3.73	2.04	未见异常	
5.41	4.98	1.41	2.63	1.54	未见异常	
5.10	6.05	3.30	3.13	1.31	异常	肝内囊性回声（考虑
6.52	4.35	1.29	2.66	1.08		
4.33	4.44	1.20	2.10	1.42	异常	肝右叶稍高回声结节
4.16	4.85	1.51	2.47	1.29	未见异常	
5.23	4.91	1.60	2.74	1.24	未见异常	
5.12	6.43	0.98	3.12	2.15	未见异常	
4.86	7.96	0.87	5.09	1.48	异常	胆囊腔内强回声团（
5.40	4.09	0.72	1.98	1.32	未见异常	
5.25	5.49	2.14	3.30	1.33	异常	肝囊肿
5.41	6.86	1.76	3.81	1.72	异常	轻度脂肪肝声像
4.87	4.88	2.80	2.39	1.27	异常	脂肪肝声像
5.05	7.55	2.94	3.93	2.26	未见异常	
6.35	4.66	3.47	2.22	1.24	异常	1.轻度脂肪肝声像2.
5.44	6.87	0.70	3.87	1.87	未见异常	
4.97	3.58	1.58	1.38	1.45	未见异常	
6.42	6.16	1.05	3.94	1.16	异常	轻度脂肪肝，肝内强
4.93	5.88	1.19	3.07	1.92	未见异常	
4.86	5.44	2.59	2.94	1.32	未见异常	
5.27	5.26	1.57	2.95	1.20	异常	腹部B超：轻度脂肪
5.81	7.17	1.16	2.97	2.52	异常	胆囊结石
6.20	5.47	1.31	2.60	1.89	异常	胆囊壁稍强回声（未
4.62	6.15	0.90	2.84	2.11	异常	胆囊结石
7.98	6.38	1.68	3.87	1.54	异常	胆囊切除术后；轻度

5.96	5.64	2.63	2.92	1.50	异常	脂肪肝声像；胆囊内
3.98	6.40	1.05	3.10	2.00	异常	胆囊结石
4.70	7.07	2.29	3.82	1.85	异常	轻度脂肪肝声像，肝
6.46	5.52	2.08	3.02	1.40	异常	脂肪肝声像
7.68	7.19	1.50	3.95	2.01	异常	胆囊结石
6.12	6.63	1.96	3.66	1.65	异常	脂肪肝声像
5.01	6.34	1.27	3.03	1.97	异常	1、胆囊息肉样病变
6.01	5.64	2.36	2.96	1.44	异常	1、胆囊结石
5.34	7.62	2.46	4.57	1.37	异常	脂肪肝声像、胆囊内
4.60	4.98	1.50	1.98	1.98	未见异常	
5.31	5.31	1.93	2.87	1.34	未见异常	
5.98	6.91	2.10	3.72	1.62	未见异常	
5.23	7.65	1.72	3.90	2.04	未见异常	
4.55	6.39	2.77	3.05	2.04	未见异常	
4.92	3.64	0.89	1.61	1.43	未见异常	
5.64	6.92	1.92	4.02	1.77	异常	轻度脂肪肝声像
5.48	6.14	2.94	3.46	1.26	异常	胆囊结石
5.84	7.89	1.25	4.15	2.06	异常	1.肝囊肿2.肝内强回
5.20	8.17	3.20	4.76	1.55	异常	脂肪肝声像
7.20	10.46	13.72	1.94	1.41	异常	腹部B超：脂肪肝声
9.11	4.95	3.27	2.62	1.35	异常	腹部B超：1、脂肪肝
8.04	4.85	2.35	2.44	1.34		
4.85	6.44	1.23	3.63	1.72	未见异常	
4.35	5.15	0.98	2.61	1.61	异常	肝囊肿
5.21	6.18	1.50	3.51	1.62	异常	所示范围内轻度脂肪
5.56	7.84	0.98	4.11	2.20	未见异常	
5.15	5.93	3.40	3.07	1.49		
5.62	4.79	1.45	2.71	1.15	未见异常	
7.77	5.71	3.05	2.77	1.57	异常	1、轻度脂肪肝声像；
5.85	5.18	1.29	2.87	1.28	未见异常	
5.44	4.76	0.68	2.35	1.73	未见异常	
4.70	4.73	2.59	2.58	1.09	未见异常	
7.15	3.46	1.32	1.36	1.33	异常	轻度脂肪肝声像，胆
4.53	6.34	1.69	3.14	1.94	未见异常	
4.66	5.94	1.36	3.04	1.83	未见异常	
3.94	7.11	2.04	3.36	1.79		
4.94	5.00	0.83	2.28	1.91	未见异常	
5.90	3.75	1.06	1.04	1.92	未见异常	
6.06	4.65	6.64	1.11	1.21	异常	腹部B超：轻度脂肪
5.22	5.93	1.65	3.17	1.72	未见异常	
4.90	5.58	0.75	3.32	1.58	未见异常	
5.61	7.21	0.90	4.37	1.73	未见异常	
5.21	3.89	3.56	1.57	1.21	异常	脂肪肝声像
5.39	6.23	2.37	3.49	1.50	异常	轻度脂肪肝
4.52	6.31	3.28	3.10	1.56	异常	轻度脂肪肝
6.57	5.26	3.77	2.44	1.39	异常	1.轻度脂肪肝声像2.
6.02	5.18	1.56	2.97	1.38	未见异常	
5.17	7.98	3.08	4.28	1.67	未见异常	
4.96	4.60	3.23	2.12	1.53	异常	肝内囊性回声（考虑
5.22	6.57	1.30	3.68	1.52	未见异常	
5.34	4.78	2.16	2.47	1.52	异常	脂肪肝声像

4.31	5.83	3.26	2.95	1.58	异常	轻度脂肪肝声像
6.82	2.95	1.49	1.00	1.42	未见异常	
4.89	5.47	1.13	2.41	1.86	未见异常	
5.56	5.34	1.51	2.94	1.65	异常	轻度脂肪肝声像。
5.11	3.59	1.63	1.75	1.15	未见异常	
6.79	4.20	1.52	2.15	1.09	异常	胆囊结石并胆囊增大
6.37	6.15	0.93	4.03	1.30	异常	1、脂肪肝声像，2、
8.15	6.79	4.98	3.06	1.51	异常	腹部B超：脂肪肝声[illegible]
4.57	5.67	0.84	3.27	1.81	异常	胆囊结石
5.52	5.32	1.10	2.89	1.75	异常	胆囊结石
5.49	5.54	1.39	2.11	2.49	未见异常	
3.64	4.48	1.22	1.87	1.66	未见异常	
4.34	7.07	4.06	3.17	1.83	未见异常	
5.15	6.24	2.57	3.14	1.52	未见异常	
4.66	6.47	2.29	3.37	1.78	异常	轻度脂肪肝声像
4.79	6.23	3.34	3.53	1.36	异常	轻度脂肪肝声像。
5.11	4.89	1.71	2.42	1.49	未见异常	
7.26	5.48	1.87	2.95	1.41	未见异常	
5.73	5.38	1.46	2.93	1.38	异常	肝内强回声斑（考虑
5.09	5.49	2.35	2.50	1.56	未见异常	
4.80	6.56	3.30	3.09	1.60	异常	脂肪肝
4.84	6.36	1.16	3.71	1.59	未见异常	
5.09	5.96	1.01	3.23	1.61	异常	胆囊息肉样病变
5.72	5.67	1.83	2.58	1.75	未见异常	
4.84	5.15	0.90	2.22	1.85	未见异常	
4.96	4.93	1.23	2.77	1.43		
6.98	7.39	1.18	3.20	2.36	未见异常	
5.17	3.82	0.74	1.16	2.11	未见异常	
4.23	5.58	2.84	2.86	1.35	未见异常	
4.70	7.16	0.92	3.61	2.17	异常	未除胆囊息肉病变
6.27	6.34	1.39	3.22	2.00	异常	肝囊肿
4.30	5.33	1.19	2.87	1.46	异常	胆囊内息肉样病变
5.15	4.04	0.94	1.76	1.69	未见异常	
4.78	4.52	1.41	2.38	1.38	异常	胆囊结石
4.49	7.94	2.09	4.29	1.66	未见异常	
4.81	6.66	1.05	3.37	2.01	未见异常	
5.60	5.50	1.87	2.87	1.49	未见异常	
6.16	8.89	1.58	3.54	2.82	未见异常	
4.76	7.34	2.41	4.18	1.69	未见异常	
5.21	5.58	1.24	2.99	1.44	异常	胆囊内稍强回声（胆
5.98	5.69	1.06	2.88	2.13	未见异常	
6.01	4.88	1.07	2.81	1.42	异常	轻度脂肪肝声像
5.79	4.97	1.01	2.45	1.86	未见异常	
5.51	7.22	1.89	4.02	1.90	未见异常	
7.39	5.27	1.56	2.99	1.45	未见异常	
8.93	5.30	1.79	2.88	1.33	异常	脂肪肝声像
4.30	6.73	1.62	3.89	1.66	异常	胆囊结石
7.03	5.11	1.91	2.37	1.63	异常	1.脂肪肝声像 2.肝[illegible]
4.70	6.07	1.73	3.37	1.67	异常	肝内囊性回声（考虑
4.33	4.53	2.64	1.91	1.35	异常	1.胆囊结石 2.胆总[illegible]
5.35	5.54	2.00	2.58	1.72	异常	肝胆脾胰B超：肝左[illegible]

5.46	5.14	1.24	2.51	1.58	未见异常	
4.40	5.24	1.86	2.89	1.49	异常	肝囊肿
4.52	5.70	1.26	2.65	1.66	未见异常	
5.69	4.72	0.74	2.03	1.92	异常	胆囊息肉样病变
4.72	6.32	3.58	3.09	1.49	未见异常	
4.73	3.06	0.84	1.18	1.40	未见异常	
4.45	5.86	1.22	3.23	1.36	异常	胆囊结石
4.81	4.19	1.26	1.98	1.36	未见异常	
5.28	5.25	1.30	2.82	1.45	异常	肝囊肿
4.71	6.15	2.38	3.39	1.37	异常	脂肪肝
5.03	5.27	1.24	2.54	1.67	未见异常	
6.16	6.18	2.38	3.22	1.55	异常	脂肪肝声像
4.68	5.36	2.01	2.98	1.39	未见异常	
5.81	6.73	0.87	3.95	1.77	异常	轻度脂肪肝声像
4.82	5.44	2.29	2.90	1.34	异常	脂肪肝声像
4.42	4.99	1.73	2.74	1.32	异常	脂肪肝声像
5.27	3.87	2.16	1.68	1.19	异常	脂肪肝声像
4.71	6.83	1.31	3.66	1.57	异常	脂肪肝声像
8.39	3.94	1.11	1.78	1.45	异常	肝左叶强回声结节,
6.07	7.43	2.25	4.62	1.52	异常	1、脂肪肝声像；2、
5.17	4.81	2.49	2.60	1.39	异常	肝囊肿
4.85	4.85	0.98	2.35	1.71	异常	胆囊息肉样病变
5.65	5.40	1.58	2.86	1.68	未见异常	
4.35	5.46	2.14	2.36	2.10	异常	脂肪肝
5.26	6.77	1.27	3.19	2.20	未见异常	
5.26	6.73	3.79	3.29	1.58	异常	轻度脂肪肝声像
5.91	5.34	1.45	2.85	1.42	未见异常	
6.27	5.37	8.58	0.96	1.10	异常	脂肪肝声像
4.06	5.34	1.13	2.56	1.72	未见异常	
5.01	5.92	1.01	3.35	1.66	异常	胆囊结石
5.24	5.41	1.75	2.98	1.40	异常	轻度脂肪肝声像
4.38	4.59	2.38	2.32	1.10	异常	肝囊肿
25.45	4.43	0.94	1.95	1.51	异常	脂肪肝声像、胆囊内
4.98	5.84	0.94	3.28	1.52	未见异常	
4.88	6.20	2.75	3.37	1.40	异常	餐后胆囊，胆囊稍强
16.02	6.41	3.79	3.48	1.41	未见异常	
5.07	7.69	2.00	4.41	1.93	未见异常	
6.01	4.26	1.01	1.84	1.61	异常	胆囊多发结石
5.34	8.37	3.00	4.37	1.77	异常	1.脂肪肝声像 2.肝[illegible]
5.74	5.49	5.53	1.62	1.64	异常	肿小囊肿，胆囊腔内
5.31	6.14	2.23	3.09	1.60	异常	脂肪肝声像
3.80	6.34	1.16	3.31	1.61	未见异常	
6.04	4.67	0.73	2.22	1.65	异常	肝内强回声斑 胆管[illegible]
8.07	5.17	2.79	2.66	1.41	异常	脂肪肝声像
4.56	7.31	1.85	4.45	1.62	异常	轻度脂肪肝声像
4.88	7.40	1.13	3.95	2.16	未见异常	
4.66	6.02	2.25	3.55	1.38	异常	1、脂肪肝声像；
4.67	5.17	4.59	1.96	1.62	未见异常	
6.34	6.81	2.72	4.17	1.35	异常	肝内强性回声斑（考
4.94	6.00	1.28	3.58	1.57	异常	肝内低回声结节：性
4.84	5.80	1.03	3.01	1.70	未见异常	

4.65	5.56	1.46	2.95	1.68	异常	脂肪肝声像，肝囊肿
5.31	7.41	1.57	4.26	1.68	未见异常	
5.05	6.89	1.19	3.57	2.06	异常	1.轻度脂肪肝声像2.
4.94	7.32	2.04	4.14	1.76	异常	轻度脂肪肝声像。
5.20	6.76	1.19	3.99	1.56	未见异常	
9.12	5.63	4.03	2.78	1.43	异常	1、脂肪肝声像；2、
4.80	1.50	2.02	3.27	6.03	未见异常	
5.12	4.99	1.66	2.16	1.89	异常	脂肪肝声像
5.39	5.67	1.31	3.41	1.45	未见异常	
5.56	3.63	1.16	2.11	0.98	异常	1.肝内囊性回声 2.[illegible]
5.59	4.68	1.57	2.65	1.28	未见异常	
5.51	6.15	1.09	3.27	1.81	未见异常	
6.02	4.64	2.40	1.91	1.77	异常	肝实质回声增粗；肝
5.09	5.58	2.94	3.03	1.30	异常	1脂肪肝声像2胆囊结
5.90	5.74	3.70	3.01	1.30	异常	脂肪肝声像。肝囊肿
4.90	4.84	0.46	2.24	1.74	未见异常	
10.53	4.94	1.21	2.68	1.51		
4.80	5.45	4.11	2.40	1.44	异常	1轻度脂肪肝声像2肝
5.11	6.33	1.68	3.66	1.35	异常	轻度脂肪肝声像。
5.44	5.07	2.25	2.74	1.12	未见异常	
4.90	4.77	0.68	1.90	1.72	未见异常	
5.32	5.64	2.00	2.55	1.92	异常	胆囊息肉病变
6.44	6.39	2.16	3.51	1.48	未见异常	
6.55	6.13	1.40	2.99	1.76	异常	脂肪肝声像
4.72	4.64	1.68	2.61	1.00	异常	胆囊结石
4.78	5.01	2.47	2.61	1.28	未见异常	
4.55	5.94	1.30	2.58	1.94	未见异常	
4.99	5.85	1.41	2.66	2.09	未见异常	
5.63	5.79	2.27	3.16	1.50	异常	脂肪肝声像
5.89	5.39	0.95	2.92	1.61	异常	1、轻度脂肪肝声像；
5.75	7.40	1.39	4.46	1.79	未见异常	
5.60	6.02	3.99	2.89	1.32	未见异常	
5.36	4.38	0.92	2.26	1.52	异常	胆囊区强回声
4.22	6.74	3.30	3.80	1.52	未见异常	
5.14	5.61	1.45	2.72	1.86	未见异常	
4.80	5.71	2.50	2.84	1.62	异常	胆囊壁欠光滑、稍毛
5.30	6.78	3.10	3.21	1.81	未见异常	
5.39	5.65	1.11	3.34	1.48	异常	肝囊肿
5.19	7.14	1.24	4.53	1.65	异常	脂肪肝声像。
5.29	5.92	1.19	3.04	1.86	异常	1、脂肪肝声像2、肝
6.41	5.60	2.14	3.09	1.28	异常	轻度脂肪肝声像
6.60	6.00	2.27	3.12	1.69	未见异常	
6.77	4.14	2.05	2.10	1.52	未见异常	
5.05	5.63	2.09	3.25	1.52	未见异常	
7.18	4.08	0.73	1.73	1.50	未见异常	
8.88	8.17	4.91	4.06	2.02	未见异常	
6.44	8.22	1.69	4.88	1.62	异常	1.肝多发囊肿2.肝内
5.73	5.09	1.52	2.59	1.60	异常	轻度脂肪肝声像
4.37	5.31	0.76	2.65	1.84	未见异常	
5.89	5.08	1.81	3.00	1.33		
6.14	8.10	1.51	3.63	2.69	未见异常	

5.20	7.14	0.90	3.71	2.01	未见异常	
4.84	5.63	0.65	3.12	1.57	异常	肝右叶内稍高回声结
4.87	5.59	2.50	2.95	1.51	异常	脂肪肝声像
5.24	4.85	2.88	2.28	1.41	未见异常	
4.31	5.52	2.39	3.01	1.25	异常	轻度脂肪肝声像
5.32	4.79	1.17	2.48	1.43	未见异常	
6.97	4.72	2.75	2.36	1.17	异常	脂肪肝声像。胆囊内
4.35	6.80	1.34	3.49	1.70	异常	肝多发性囊肿声像
5.66	5.26	1.26	2.81	1.53	异常	脂肪肝声像
4.86	4.50	1.25	2.43	1.28	异常	肝多发囊肿
5.59	6.63	1.94	3.45	1.75	未见异常	
4.73	3.58	0.88	3.29	1.62	异常	肝左叶内稍高回声区
4.44	5.40	1.50	2.77	1.60	未见异常	
4.98	4.51	0.94	2.36	1.52	异常	肝多发囊肿　肝内强
7.05	5.93	1.35	3.12	1.66	未见异常	
5.88	4.54	0.96	2.28	1.60	异常	脾门处异常实质回声
5.44	7.38	3.43	4.23	1.51	异常	轻度脂肪肝
6.12	5.78	1.80	3.52	1.21	未见异常	
4.50	4.47	1.70	2.22	1.40		
5.00	5.28	5.60	1.80	1.29	未见异常	
11.22	5.67	1.32	3.22	1.37	异常	胆囊壁稍强光点（考
5.47	5.88	1.25	3.27	1.50	异常	轻度脂肪肝声像
5.53	4.14	1.15	1.84	1.35	未见异常	
4.59	4.79	2.02	2.27	1.45		
4.97	4.20	0.78	1.82	1.67	未见异常	
5.11	5.08	1.30	2.30	1.65	未见异常	
5.10	4.82	1.19	2.52	1.57	异常	胆囊结石
4.57	6.83	2.10	3.68	1.84	异常	轻度脂肪肝声像
4.78	3.37	2.33	1.22	1.33	未见异常	
5.27	6.37	2.77	3.18	1.77	未见异常	
4.94	5.86	2.27	2.88	1.70	异常	胆囊多发性结石
4.70	5.22	0.66	2.41	1.74	未见异常	
4.82	7.12	1.26	4.06	1.88	异常	腹部B超：脂肪肝声
5.00	4.32	1.80	2.31	1.21	异常	脂肪肝声像
4.96	5.08	1.72	2.56	1.53	异常	脂肪肝声像
5.17	4.73	0.90	3.17	1.01	异常	脂肪肝声像
4.75	6.02	1.18	2.87	2.05	未见异常	
5.72	5.09	3.25	2.31	1.71	未见异常	
5.10	4.27	0.84	1.45	2.14	未见异常	
5.06	5.47	1.06	2.94	1.42	未见异常	
5.43	5.78	7.71	2.49	2.07	未见异常	
5.62	5.56	2.53	2.88	1.51	异常	脂肪肝声像
12.54	6.26	2.17	3.19	1.89	异常	脂肪肝声像
4.28	4.45	1.36	1.99	1.52	异常	胆囊结石
9.14	5.35	2.05	2.57	1.49	未见异常	
4.63	5.49	0.64	1.90	2.26	未见异常	
4.17	3.85	1.31	1.56	1.66	未见异常	
5.52	5.45	1.10	3.11	1.54	异常	肝实质回声稍增粗
13.50	4.74	1.11	2.24	1.64	未见异常	
5.55	6.61	2.66	3.65	1.73	未见异常	
5.44	5.22	1.52	2.91	1.55	异常	轻度脂肪肝声像

4.22	4.65	1.20	2.60	1.35	异常	肝多发囊肿
5.07	7.76	1.00	4.78	1.63	异常	肝内囊性回声（考虑
6.15	10.73	5.83	4.39	1.99	异常	1.轻度脂肪肝声像2.
5.95	6.69	0.98	3.23	2.17	异常	肝内低回声区
5.46	7.24	1.50	4.05	1.71	未见异常	
6.81	6.49	1.01	4.06	1.38	异常	肝多发囊肿
11.07	5.05	2.61	2.48	1.46	异常	脂肪肝声像
4.35	6.70	1.55	3.07	2.23	未见异常	
4.23	6.38	2.62	3.25	1.74	未见异常	
4.64	4.50	0.93	1.89	1.86	未见异常	
5.12	8.26	1.14	4.69	2.09	异常	肝胆脾胰B超：轻度
4.82	6.80	1.16	3.32	2.19	未见异常	
6.32	5.15	4.95	1.63	1.66	异常	轻度脂肪肝声像，肝
5.47	4.89	3.69	2.06	1.52	未见异常	
4.20	5.10	0.98	2.78	1.24	未见异常	
5.05	6.35	1.61	3.50	1.60	未见异常	
5.08	5.56	0.82	2.87	1.58	异常	肝内强回声斑：考虑
5.62	4.96	2.01	2.34	1.62	未见异常	
4.99	4.92	0.93	2.85	1.30	异常	肝多发囊行回声
5.72	6.28	2.81	3.77	1.44	未见异常	
9.15	8.25	3.35	4.34	1.70	异常	脂肪肝声像
7.13	5.05	1.57	2.26	1.84	未见异常	
7.28	7.27	3.06	3.84	1.91	未见异常	
5.15	5.40	1.46	2.74	1.61	未见异常	
5.47	5.39	2.14	3.02	1.38		
5.09	6.52	1.45	3.77	1.42	未见异常	
4.47	4.91	1.01	2.27	1.79	未见异常	
7.43	5.33	1.36	3.45	1.06	未见异常	
5.34	6.56	1.84	3.19	1.63	未见异常	
5.51	5.15	2.60	2.58	1.76	异常	1、脂肪肝声像2、胆
4.86	4.23	0.93	1.56	1.96	异常	腹部B超：肝多发囊
7.14	7.11	2.49	4.01	1.46	异常	胆囊息肉样病变
4.61	5.25	1.99	2.06	2.01	异常	胆囊内强回声（结石
6.38	6.51	2.96	3.27	1.67	异常	脂肪肝；胆囊偏小似
5.09	4.25	1.61	2.39	1.19	未见异常	
5.04	4.90	2.29	2.45	1.24	未见异常	
8.43	6.67	2.49	3.64	1.54	异常	脂肪肝声像，肝左叶
4.84	4.44	1.24	1.79	2.07	未见异常	
5.36	4.79	1.99	2.40	1.46	异常	脂肪肝声像
5.26	6.85	3.53	3.71	1.67	异常	胆囊结石
5.34	8.02	5.39	3.41	1.95	异常	肝内强回声，考虑钙
7.10	4.49	2.42	2.33	1.38	异常	脂肪肝声像
4.70	6.62	3.69	3.47	1.75	异常	脂肪肝声像
5.29	4.86	0.59	2.20	1.81	异常	腹部B超：1、肝脏弥
4.32	6.32	3.56	3.34	1.34	异常	脂肪肝声像
5.89	3.79	0.76	1.51	1.60	未见异常	
5.77	4.25	1.76	1.60	1.74	异常	脂肪肝声像 ，胆囊
5.99	6.11	1.93	3.16	1.87	异常	脂肪肝
4.84	7.83	1.60	4.48	1.81	异常	胆囊切除术后
22.12	6.34	2.06	3.42	1.68	异常	胆囊结石
5.61	11.56	1.58	7.51	1.52	异常	1、轻度脂肪肝声像；

5.27	4.66	0.88	1.78	2.12	未见异常	
4.90	6.18	0.64	2.60	2.26	未见异常	
4.74	5.90	2.07	2.65	2.12	未见异常	
5.03	4.20	1.11	2.24	1.43	异常	肝囊肿
4.46	6.41	2.80	3.54	1.35	未见异常	
5.29	6.27	3.28	3.13	1.48	异常	轻度脂肪肝
6.73	4.93	0.97	2.51	1.55	未见异常	
5.28	5.68	2.14	2.93	1.62	异常	1、轻度脂肪肝，2、
4.59	4.78	2.02	2.51	1.32	异常	1、脂肪肝；2、胆囊
5.60	3.12	1.91	1.36	1.14	异常	轻度脂肪肝声像
5.87	6.47	1.93	3.64	1.57	未见异常	
5.42	4.97	1.23	2.87	1.33	未见异常	
5.49	5.89	3.06	2.47	2.03	异常	肝脏实质光点回声稍
4.46	6.07	2.16	2.82	1.85	未见异常	
7.25	6.46	2.15	3.78	1.46	异常	轻度脂肪肝声像
4.94	6.13	1.39	3.00	2.06	未见异常	
4.82	5.27	0.56	2.07	2.01	未见异常	
4.45	7.13	2.65	3.97	1.60	异常	轻度脂肪肝声像；肝
4.89	4.43	2.23	2.44	1.21	异常	轻度脂肪肝声像。
5.56	5.54	2.26	2.78	1.77	未见异常	
4.98	7.45	2.64	4.06	1.81	异常	胆囊结石
6.59	6.62	1.37	4.06	1.27	未见异常	
5.13	5.62	1.31	2.80	1.81	异常	轻度脂肪肝声像；肝
5.03	4.02	1.29	1.47	1.91		
9.05	5.51	0.58	2.79	1.67	异常	胆囊充满型结石可能
5.63	6.92	2.68	3.52	1.72	异常	1、轻度脂肪肝声像；
5.03	6.30	1.84	3.56	1.62	异常	轻度脂肪肝声像；胆
5.85	5.64	0.94	2.93	1.44	未见异常	
7.48	6.42	2.81	3.61	1.50	未见异常	
5.01	8.57	1.14	5.40	2.01	未见异常	
5.33	6.64	1.49	3.54	1.65	未见异常	
5.04	5.10	2.40	2.48	1.41	异常	轻度脂肪肝声像
4.48	5.54	2.40	2.27	1.95	异常	腹部B超：轻度脂肪
5.35	5.10	1.93	2.98	1.32	未见异常	
5.31	6.12	0.83	3.13	2.28	未见异常	
5.92	5.35	2.51	2.53	1.43	未见异常	
6.00	5.80	0.91	3.02	2.05	未见异常	
5.04	5.61	0.80	2.91	1.57	未见异常	
8.78	5.75	2.76	2.76	1.59	未见异常	
5.93	7.34	2.08	4.08	1.64	异常	1、轻度脂肪肝声像；
7.15	7.06	2.44	3.98	1.74	未见异常	
5.28	6.27	2.25	2.87	1.63	未见异常	
6.26	6.94	2.78	3.55	1.61	未见异常	
5.13	3.78	0.63	2.02	1.23	未见异常	
4.45	5.43	1.64	3.40	1.16	异常	肝内囊性回声
5.01	6.56	1.76	3.40	2.02	异常	轻度脂肪肝声像
5.18	5.84	1.60	3.00	1.67	未见异常	
6.00	5.90	3.18	3.31	1.15	异常	脂肪肝声像；胆囊内
6.40	5.20	2.03	2.63	1.50	异常	肝胆脾胰B超：脂肪
5.13	5.27	0.76	2.43	2.00	异常	胆囊息肉样病变
6.06	4.95	2.85	2.47	1.20	异常	轻度脂肪肝声像

5.19	5.43	2.80	2.82	1.24	未见异常	
5.40	4.27	2.17	2.30	1.11	异常	肝囊肿
5.83	4.50	0.82	2.58	1.44	未见异常	
5.51	9.20	7.58	2.98	1.44	异常	轻度脂肪肝声像
5.32	5.54	3.92	2.61	1.44	异常	脂肪肝声像
6.69	7.75	1.98	4.33	1.59	异常	多发性胆囊结石
7.02	5.48	0.89	2.25	2.17	未见异常	
4.94	6.99	1.17	3.17	2.62	未见异常	
5.79	6.28	1.70	3.52	1.63	未见异常	
5.11	5.63	0.96	2.75	1.72	未见异常	
5.61	6.51	3.64	3.18	1.58	未见异常	
4.93	7.34	1.40	4.26	1.75	异常	轻度脂肪肝声像
5.50	5.99	1.66	2.88	1.99	异常	肝内囊性回声（考虑
4.92	4.94	0.88	2.26	2.06	未见异常	
7.23	6.52	2.65	3.66	1.45	异常	脂肪肝声像
5.32	4.84	2.64	2.22	1.46	异常	脂肪肝声像
5.77	5.69	1.18	3.30	1.44	异常	肝囊肿
5.44	3.80	0.66	1.07	1.83	未见异常	
14.37	5.81	4.04	2.58	1.49	异常	脂肪肝声像
5.38	5.38	0.89	2.94	1.79	未见异常	
5.22	6.14	2.86	3.54	1.36	异常	脂肪肝声像
4.40	4.37	0.87	2.38	1.35	未见异常	
5.11	6.19	1.17	3.16	1.83	未见异常	
14.68	5.77	7.15	1.81	1.28	异常	1，脂肪肝声像2，肝
9.12	6.20	1.79	3.81	1.29	异常	脂肪肝声像，胆囊内
6.06	6.01	2.33	2.99	1.89	未见异常	
4.93	5.56	1.71	3.05	1.58	异常	胆囊壁欠光滑
5.17	7.83	1.11	4.30	1.99	未见异常	
4.84	4.84	0.99	2.39	1.50	未见异常	
6.00	4.62	0.75	2.42	1.22	异常	1、轻度脂肪肝声像2
5.59	5.02	1.11	2.85	1.48	未见异常	
4.64	5.61	2.61	2.80	1.39	未见异常	
4.80	3.97	0.59	1.95	1.52	未见异常	
4.94	5.00	2.10	2.72	1.51		
5.51	4.98	0.87	2.56	1.54	未见异常	
6.31	3.67	1.41	1.93	1.01	未见异常	
5.01	6.19	1.04	3.05	1.89		
7.04	6.55	1.99	3.31	1.68	未见异常	
5.69	6.87	10.84	1.07	1.06	异常	1、轻度脂肪肝声像;
5.89	5.77	2.49	2.83	1.35	未见异常	
4.94	6.36	1.45	3.48	1.99	未见异常	
5.67	5.98	1.35	3.79	1.25	异常	轻度脂肪肝声像。
4.43	9.46	1.08	5.42	2.36	未见异常	
4.58	4.80	2.06	2.45	1.22	未见异常	
9.14	3.21	1.22	1.45	1.23	异常	1、脂肪肝声像2、胆
4.46	6.39	1.98	3.59	1.71	异常	轻度脂肪肝声像
4.68	5.97	0.80	2.74	1.88	未见异常	
6.14	5.11	3.13	2.45	1.45	未见异常	
5.82	6.25	1.11	3.48	1.87	异常	胆囊内稍强回声团，
4.80	5.40	1.16	2.81	1.51	异常	胆囊息肉样病变
3.90	5.78	1.35	2.33	2.22	异常	腹部B超：肝内囊性[illegible]

4.24	6.03	1.10	3.60	1.53	未见异常	
5.03	4.66	1.25	1.88	2.02	异常	轻度脂肪肝
5.33	5.75	1.05	2.98	1.57	异常	轻度脂肪肝声像
5.01	4.84	0.76	2.06	1.85	未见异常	
4.76	6.52	3.37	3.20	1.56	未见异常	
4.90	5.31	2.06	3.01	1.46	异常	轻度脂肪肝声像
5.62	6.02	1.15	3.42	1.63	未见异常	
11.00	8.43	3.43	4.41	2.24	未见异常	
5.10	6.31	1.74	3.73	1.45	异常	胆囊多发结石
5.10	6.96	3.14	3.33	1.79	异常	轻度脂肪肝声像
4.97	4.76	1.15	2.01	1.69	未见异常	
10.48	6.67	2.14	3.72	1.55	未见异常	
4.55	1.91	1.43	2.53	1.91	异常	1、肝囊肿； 2、胆[illegible]
6.56	5.82	3.25	3.10	1.50	异常	1，轻度脂肪肝声像，
6.01	1.65	1.65	1.70	1.52	未见异常	
5.92	6.99	3.96	3.55	1.43	异常	脂肪肝声像
7.07	5.76	0.95	2.72	1.84	异常	胆囊结石
4.09	6.18	2.22	3.15	1.82	未见异常	
4.73	6.46	1.28	3.39	1.88	未见异常	
4.88	9.15	2.02	4.52	2.33	未见异常	
6.77	4.31	1.83	2.15	1.12	未见异常	
6.12	6.19	1.31	3.45	1.69	异常	1.轻度脂肪肝声像2.
5.63	5.67	1.95	3.15	1.53	异常	脂肪肝声像
4.34	4.94	0.96	2.38	1.62	未见异常	
7.18	7.60	2.40	4.45	1.87	异常	胆囊多发结石
4.65	4.43	1.05	1.84	1.68	未见异常	
5.17	6.33	1.20	3.49	1.50	未见异常	
4.35	4.62	0.65	2.31	1.54	未见异常	
7.92	5.22	4.56	2.35	1.26	异常	轻度脂肪肝声像
4.66	6.39	1.45	3.62	1.70	异常	轻度脂肪肝声像
7.47	6.47	2.32	3.58	1.45	未见异常	
4.99	5.72	1.02	2.87	1.95	异常	脂肪肝声像。肝内囊
4.77	6.54	0.92	3.48	1.76	未见异常	
5.80	6.55	1.11	2.97	2.01	未见异常	
4.14	5.48	1.20	2.94	1.66	异常	肝囊肿
4.93	4.09	1.79	1.83	1.57	异常	腹部B超：胆囊异常[illegible]
4.70	6.59	2.30	3.45	1.93	异常	肝内强回声斑，考虑
4.96	7.09	2.55	3.25	2.04	异常	轻度脂肪肝、肝多发
5.99	5.48	0.99	2.54	1.79	未见异常	
4.59	6.96	1.72	3.97	1.70	异常	脂肪肝声像；胆囊内
5.41	6.05	0.67	2.83	1.91	未见异常	
5.27	7.08	1.69	4.68	1.44	异常	腹部B超：1、脂肪肝
4.77	5.72	1.30	3.05	1.61	未见异常	
5.62	6.60	1.13	3.32	1.98	异常	肝胆胰彩超：1、轻[illegible]
4.71	4.99	2.00	2.43	1.51	异常	餐后胆囊（未除胆囊
7.29	3.79	3.56	1.44	1.26	异常	1.胆囊点状弱强回声
4.89	6.58	1.48	3.99	1.53	异常	轻度脂肪肝声像
4.42	5.44	1.59	2.48	1.87	未见异常	
19.14	5.55	2.65	2.86	1.41	异常	轻度脂肪肝声像
5.01	5.22	0.92	2.40	2.13	异常	1.肝多发囊性回声（
6.93	4.34	0.91	1.75	1.84	未见异常	

4.65	3.97	2.66	1.86	1.22	异常	脂肪肝声像
5.58	5.03	1.22	2.44	1.56	未见异常	
5.66	7.49	1.33	3.62	2.35	未见异常	
4.92	5.23	1.41	2.71	1.65	异常	肝内囊性回声（考虑
11.86	5.48	1.07	2.79	1.72	异常	脂肪肝声像
5.76	5.91	2.45	3.06	1.61	异常	轻度脂肪肝声像
4.88	5.42	0.68	1.57	2.76	未见异常	
9.05	3.81	0.84	1.63	1.40	异常	胆囊结石
7.36	4.71	5.06	1.88	1.07	异常	轻度脂肪肝声像
5.40	6.71	1.77	4.12	1.58	异常	脂肪肝声像
5.97	6.51	2.15	3.87	1.70	异常	脂肪肝声像
4.75	5.52	1.14	2.95	1.62	异常	肝多发囊性回声(考
6.60	6.19	1.56	3.54	1.73	异常	1肝内强回声（钙化
5.36	6.92	0.92	3.56	2.03	异常	轻度脂肪肝；肝实质
5.05	4.95	0.63	2.67	1.59		
4.88	6.20	0.99	3.38	1.69	异常	脂肪肝声像；胆囊内
6.31	4.22	1.18	2.12	1.33	异常	肝囊肿
7.65	7.93	1.42	4.76	1.77	未见异常	
9.18	7.02	0.98	3.94	1.99	未见异常	
4.85	5.97	1.96	3.50	1.56	未见异常	
13.55	7.13	1.44	3.89	1.94	异常	1、脂肪肝声像
5.29	7.41	1.64	4.99	1.43	异常	轻度脂肪肝声像。
4.75	5.39	1.26	2.72	1.53	异常	胆囊结石
5.77	5.87	1.67	3.51	1.50	异常	脂肪肝声像
4.97	6.98	2.93	3.71	2.00	异常	1、轻度脂肪肝声像，
4.31	5.01	1.05	2.48	1.38	异常	肝内强回声改变考虑
4.56	6.86	6.44	1.98	1.51	未见异常	
4.95	4.25	1.14	2.16	1.34	异常	轻度脂肪肝声像、肝
5.26	7.02	2.58	3.86	1.86	异常	肝囊肿
5.23	5.33	1.39	3.04	1.34	未见异常	
5.21	6.24	1.35	3.19	1.79	未见异常	
4.81	6.62	3.08	3.58	1.44	异常	1.脂肪肝声像2.肝囊
6.18	6.68	6.55	2.14	1.64	异常	脂肪肝声像
6.41	4.91	1.38	2.79	1.46	异常	脂肪肝声像；肝囊肿
7.35	5.58	0.83	3.27	1.71	未见异常	
4.86	6.09	1.47	3.75	1.50	异常	轻度脂肪肝声像、胆
6.06	6.37	1.69	3.40	1.87	异常	腹部B超：胆囊多发
7.49	7.06	3.89	3.79	1.38	异常	脂肪肝声像
8.49	6.68	2.63	3.76	1.55	异常	脂肪肝声像
5.23	6.20	2.78	3.33	1.67	异常	脂肪肝声像、胆囊结
5.63	5.96	3.22	2.98	1.40	异常	腹部B超:1、肝囊肿2
6.22	6.66	3.51	3.65	1.49	异常	胆囊结石
4.90	4.80	1.64	2.16	1.70	未见异常	
5.29	4.73	3.80	1.95	1.29	异常	1 轻度脂肪肝，2 胆
6.13	5.54	1.14	2.95	1.78	异常	肝脏实质光点回声增
5.46	6.37	2.17	3.58	1.50	未见异常	
4.97	4.34	1.02	1.98	1.59	异常	脂肪肝；胆囊结石；
6.05	5.08	1.35	2.50	1.58	未见异常	
8.14	7.37	1.89	4.23	1.73	异常	脂肪肝声像
4.59	4.76	0.99	2.31	1.60	异常	肝小囊肿 胆囊结石
5.29	2.66	0.61	1.07	1.02	未见异常	

4.18	6.25	3.73	3.10	1.30	未见异常	
5.01	6.21	1.19	3.64	1.42	异常	1、脂肪肝声像； 2、
4.77	6.05	1.62	3.21	1.66	异常	轻度脂肪肝声像
5.11	7.17	1.37	3.76	2.03	未见异常	
5.14	5.70	1.32	3.16	1.58	未见异常	
5.78	5.79	0.85	3.59	1.14	未见异常	
7.09	5.70	10.49	0.77	1.02	未见异常	
4.93	5.20	0.69	2.69	1.79	未见异常	
5.45	7.15	0.83	4.07	1.98	异常	肝囊肿
8.23	5.15	4.93	2.08	1.33	异常	轻度脂肪肝声像
5.39	5.49	3.05	3.03	1.23	异常	1、脂肪肝声像
6.41	6.00	7.95	1.35	1.26	异常	胆囊结石
5.48	6.09	2.58	3.39	1.55	异常	轻度脂肪肝声像；肝
4.65	5.04	1.03	2.13	1.77	未见异常	
7.40	5.49	5.74	2.17	1.36	异常	脂肪肝声像
5.42	6.00	3.00	3.31	1.55	异常	胆囊息肉样病变
5.48	6.07	2.78	3.09	1.63	异常	脂肪肝声像
5.10	6.14	1.40	2.91	1.95	异常	肝内多发稍强回声小
6.39	5.51	1.54	2.57	1.94	异常	脂肪肝声像
6.37	5.04	1.50	2.76	1.39	异常	轻度脂肪肝声像
7.09	5.06	1.56	2.80	1.28	未见异常	
12.17	7.08	2.20	4.24	1.70	未见异常	
5.05	7.00	2.77	3.91	1.74		
4.87	5.37	0.75	1.90	2.43	未见异常	
5.10	7.14	1.72	4.13	1.66	未见异常	
6.21	6.07	2.34	3.19	1.79	未见异常	
11.25	5.18	1.84	3.08	1.41	异常	腹部B超：1、轻度脂
5.35	2.52	0.70	0.66	1.43	异常	肝小囊肿,胆囊切除
4.30	7.79	2.83	4.08	1.71	异常	脂肪肝；肝右叶低回
4.92	7.49	1.70	4.21	7.89	异常	轻度脂肪肝声像
5.32	4.97	1.87	2.71	1.50	未见异常	
5.48	5.37	2.68	2.91	1.22	异常	肝内强回声斑，考虑
6.43	5.32	1.03	2.73	1.58	异常	轻度脂肪肝声像
4.96	6.64	1.76	3.48	1.60	异常	胆囊壁稍毛糙
6.12	4.14	1.45	2.20	0.96	异常	脂肪肝声像
5.67	3.21	1.01	1.45	1.29	未见异常	
5.49	5.45	0.90	2.56	1.76	异常	胆囊多发结石
5.27	6.74	1.36	3.46	2.13	未见异常	
7.47	4.95	1.12	2.48	1.45	异常	胆囊结石
5.11	4.99	0.96	2.67	1.68	未见异常	
7.42	5.95	0.96	3.49	1.75	异常	胆囊壁强回声：结石
4.84	7.00	1.91	3.68	2.15	异常	轻度脂肪肝声像
4.52	5.35	1.56	2.84	1.58	未见异常	
4.73	5.18	0.99	2.63	1.84	未见异常	
5.32	7.52	2.55	4.25	1.79	未见异常	
29.43	4.57	4.99	1.71	1.12	异常	轻度脂肪肝
6.11	4.98	1.36	2.73	1.22	异常	轻度脂肪肝声像
5.13	6.45	1.15	3.32	1.83	未见异常	
6.02	5.18	1.11	3.00	1.49	异常	轻度脂肪肝声像
5.18	5.38	1.58	2.78	1.67	异常	轻度脂肪肝声像；2、
4.76	6.49	1.68	3.83	1.64	异常	胆囊内强回声（考虑

6.10	7.27	1.04	4.55	1.96	异常	腹部B超：胆囊结石
5.47	5.27	1.25	2.97	1.53	异常	肝胆胰彩超：肝内囊
5.36	5.30	1.39	2.57	1.95	异常	肝囊肿
4.36	4.81	1.04	2.54	1.30	未见异常	
6.81	5.82	2.13	3.16	1.33	异常	轻度脂肪肝声像
5.13	5.97	2.72	3.31	1.49	异常	胆囊内强回声（考虑
4.33	6.18	1.61	3.10	1.79	异常	肝囊肿
4.33	5.19	2.96	2.66	1.15	异常	轻度脂肪肝、肝囊肿
4.89	4.23	2.78	1.90	1.40	异常	胆囊结石
15.84	3.99	1.37	1.80	1.59	异常	轻度脂肪肝声像
5.07	5.41	1.32	2.38	2.12	未见异常	
7.92	7.62	1.48	4.96	1.72	异常	轻度脂肪肝声像
4.47	6.27	1.02	3.15	1.86	异常	肝实质回声稍增粗
4.96	5.66	2.17	2.83	1.83	异常	肝内囊性回声（考虑
6.63	3.70	1.57	1.55	1.61	未见异常	
5.28	5.25	2.23	2.74	1.66	异常	胆囊结石
4.69	6.42	1.32	3.48	1.84	未见异常	
6.03	5.20	0.66	3.00	1.54	未见异常	
4.27	6.49	1.68	3.74	1.50	未见异常	
5.10	6.72	0.92	3.75	1.83	未见异常	
5.18	4.43	1.27	2.12	1.39	异常	所属范围内脂肪肝声
4.89	6.62	1.29	3.88	1.81	异常	脂肪肝声像。
4.04	7.64	3.03	4.18	1.57	未见异常	
7.33	4.28	1.75	2.38	1.37	异常	轻度脂肪肝声像；肝
6.22	6.30	1.78	3.10	1.84	异常	轻度脂肪肝声像
5.92	4.49	1.21	2.36	1.52	异常	肝胆胰彩超：轻度脂
5.69	6.16	1.94	3.45	1.58	异常	轻度脂肪肝、肝囊肿
4.73	5.40	2.60	3.33	1.05	异常	脂肪肝声像，胆脾胰
4.68	6.11	1.47	3.25	1.63	异常	肝小囊肿
8.64	6.89	1.35	3.65	1.95	未见异常	
4.29	6.61	1.67	3.47	1.96	未见异常	
6.25	5.50	1.81	2.65	1.74	未见异常	
7.20	3.98	2.36	2.08	1.17	异常	腹部B超：1、肝囊肿
4.91	6.10	1.11	3.61	1.57	异常	胆囊内强回声（考虑
4.26	4.27	0.84	1.68	1.79	异常	轻度脂肪肝声像
5.52	3.95	2.22	1.82	1.24	未见异常	
6.43	7.03	2.42	4.18	1.58	未见异常	
8.75	6.43	2.44	3.42	1.52	异常	轻度脂肪肝
16.75	6.85	2.44	3.33	2.12	异常	轻度脂肪声像
6.13	8.69	1.94	4.88	1.89	未见异常	
4.43	6.12	0.92	3.15	1.71	未见异常	
5.16	4.70	2.39	2.19	1.54	异常	轻度脂肪肝声像
8.30	4.90	2.71	2.30	1.33	异常	轻度脂肪肝
5.69	1.94	1.88	2.53	1.42	异常	肝囊肿
4.89	8.20	2.22	4.86	1.88	异常	胆囊内强回声（考虑
9.72	8.42	4.12	4.63	1.54	异常	轻度脂肪肝声像、胆
15.62	5.32	3.58	2.82	1.21	异常	1.脂肪肝声像2.胆囊
5.42	6.43	6.94	1.71	1.34	异常	腹部B超：胆囊多发
6.12	3.17	0.91	0.76	1.93	未见异常	
4.66	4.18	1.86	2.23	1.26	异常	1、轻度脂肪肝声像；
4.49	5.03	0.78	2.35	1.74	未见异常	

5.43	6.48	2.30	3.69	1.42	异常	轻度脂肪肝
5.38	4.53	3.89	2.15	1.40	未见异常	
4.86	6.67	1.85	3.31	1.97	异常	肝囊肿
5.65	5.36	1.21	2.63	1.97	异常	轻度脂肪肝声像
5.20	4.75	0.98	2.23	1.87	异常	胆囊内强回声(考虑纟
2.87	4.97	3.56	2.29	1.63	异常	轻度脂肪肝声像
4.80	5.73	2.08	2.95	1.76	异常	1、脂肪肝声像 2、
6.72	5.20	1.40	2.46	1.73		
4.98	4.49	0.91	2.39	1.43	异常	轻度脂肪肝声像
4.41	5.49	2.17	2.97	1.33	未见异常	
5.37	7.04	3.35	3.97	1.61	未见异常	
6.28	6.54	3.69	3.70	1.58	异常	轻度脂肪肝声像
4.61	4.80	1.06	2.11	1.73	未见异常	
5.15	5.69	0.88	2.91	1.52	异常	肝小囊肿
4.43	6.25	0.85	3.57	1.60	异常	胆囊内异常声像
5.10	4.44	1.26	2.24	1.54	异常	1、脂肪肝声像，2、
4.80	7.96	2.89	4.33	1.93	未见异常	
7.48	6.76	1.36	4.09	1.72		
5.42	4.54	0.89	2.07	1.76	异常	1、轻度脂肪肝声像；
4.91	5.28	1.07	3.07	1.35	异常	脂肪肝声像
5.75	6.85	0.91	3.06	2.46	未见异常	
5.42	3.33	2.33	1.33	1.28	未见异常	
5.12	5.87	2.66	3.07	1.54	异常	轻度脂肪肝
5.02	6.85	1.26	3.91	1.76	未见异常	
9.09	3.95	1.62	1.69	1.55	异常	腹部B超：轻度脂肪
5.05	4.65	1.10	2.14	1.57	异常	胆囊结石；脾大
5.10	5.23	2.47	2.71	1.30	异常	轻度脂肪肝声像
8.07	4.78	1.41	2.23	1.42	异常	肝囊肿
5.66	8.38	4.49	3.77	1.90	异常	1、轻度脂肪肝声像。
5.14	5.59	0.69	2.45	2.30	未见异常	
8.24	3.98	0.65	2.27	1.15	未见异常	
4.42	6.47	1.71	3.43	1.79	异常	肝囊肿
5.04	7.05	1.00	4.21	1.51	未见异常	
10.26	4.96	0.65	2.81	1.47	未见异常	
4.46	6.09	3.23	2.97	1.58	异常	轻度脂肪肝
4.34	5.36	1.95	2.98	1.43	异常	轻度脂肪肝声像
6.79	4.21	1.06	1.91	1.41	异常	1.胆囊腔内强回声团
4.77	5.60	1.14	2.72	1.82	未见异常	
4.58	10.60	5.39	4.31	1.86	异常	1、轻度脂肪肝声像2
5.25	5.87	1.64	2.76	1.92	未见异常	
5.00	6.62	1.57	3.16	1.94	异常	轻度脂肪肝声像
4.91	4.25	1.09	2.30	1.02	未见异常	
4.29	5.87	2.71	3.21	1.58	异常	脂肪肝声像
4.56	5.33	1.71	2.73	1.68	异常	轻度脂肪肝声像
4.58	6.81	1.43	3.66	1.84	未见异常	
5.44	4.89	0.84	2.42	1.89	未见异常	
5.81	5.80	1.72	3.19	1.70	异常	轻度脂肪肝声像
6.27	5.71	1.32	2.99	1.77	异常	脂肪肝声像；胆汁粘
7.53	6.78	3.25	2.84	2.02	未见异常	
5.53	5.13	2.60	2.90	1.32	未见异常	
4.85	4.22	0.88	2.33	1.32	未见异常	

6.15	4.89	2.06	2.60	1.18	异常	肝囊肿
5.96	7.36	2.71	3.57	1.92	未见异常	
7.87	5.65	2.33	3.11	1.29	异常	轻度脂肪肝
5.61	5.98	0.59	2.58	1.99	异常	肝内稍高回声结节，
5.09	5.45	3.78	2.60	1.39	异常	1.脂肪肝声像；2.肝
4.61	6.29	0.85	3.74	1.61	异常	胆囊内强回声(考虑
5.06	5.55	1.13	2.85	1.70	异常	1、轻度脂肪肝声像；
4.97	2.92	0.77	1.20	1.32	未见异常	
5.14	4.68	1.00	2.29	1.62	异常	肝囊肿
4.72	6.81	1.23	2.85	2.32	异常	胆囊结石；
5.75	5.54	1.29	1.97	2.56	异常	胆囊内稍强回声团，
5.98	6.73	0.66	3.90	1.79	未见异常	
5.25	5.86	1.35	2.54	2.25	未见异常	
4.83	4.52	0.72	2.17	1.62	未见异常	
5.64	7.10	1.66	4.21	1.64	异常	胆囊内强回声（考虑
5.13	6.65	4.39	3.21	1.57	异常	肝囊肿
8.83	3.89	0.86	1.74	1.47	异常	轻度脂肪肝声像
4.91	5.79	1.01	2.17	2.35	未见异常	
4.85	5.20	1.65	2.74	1.32	异常	1.脂肪肝声像2.肚囊
4.32	5.18	2.30	2.44	1.64	未见异常	
5.57	6.63	0.92	3.46	2.09	异常	轻度脂肪肝，肝囊息
5.02	5.13	0.86	2.82	1.57	异常	胆囊内强回声(未除
5.65	7.59	1.13	4.33	1.83	异常	胆囊息肉样病变
4.92	8.08	1.04	4.56	2.05	未见异常	
5.21	6.69	2.82	3.26	1.75	异常	轻度脂肪肝、肝囊肿
5.19	4.17	0.89	1.97	1.31	未见异常	
7.04	8.36	17.14	1.68	1.11	异常	1、脂肪肝声像2、肝
5.35	3.76	2.80	1.76	1.21	未见异常	
5.03	4.31	1.27	1.78	1.75	未见异常	
4.42	8.23	1.00	4.57	2.13	异常	胆囊结石
4.74	5.92	1.48	3.45	1.45	异常	轻度脂肪肝声像
6.31	7.94	3.27	4.12	1.81	异常	脂肪肝声像
4.63	6.41	2.07	3.76	1.34	未见异常	
4.76	6.89	0.52	3.41	2.34	异常	肝内强回声斑：考虑
5.45	6.20	1.79	3.50	1.55	异常	轻度脂肪肝声像；
6.90	5.49	0.92	3.00	1.64	未见异常	
5.43	7.02	1.10	3.21	2.69	未见异常	
5.27	5.92	0.82	2.85	1.82	异常	轻度脂肪肝声像
5.21	4.55	0.96	1.43	2.35	异常	胆囊息肉样变
5.20	4.05	0.79	2.00	1.54		
5.32	5.46	6.00	1.84	1.26	异常	脂肪肝声像；肝内囊
4.39	5.39	1.49	2.43	1.80	异常	胆囊结石
4.89	5.88	1.85	3.11	1.74	异常	轻度脂肪肝；肝内强
5.55	5.73	0.94	2.98	1.68	异常	胆囊结石
5.13	5.82	4.69	2.71	1.18	未见异常	
4.38	6.64	3.12	2.88	1.60	异常	轻度脂肪肝，肝囊息
4.78	5.36	1.34	2.71	1.47		
6.41	5.20	0.78	2.63	1.53	异常	胆囊息肉样病变
5.50	4.34	2.27	2.18	1.14	异常	所属范围内轻度脂肪
6.58	5.46	1.24	2.47	1.93	未见异常	
5.04	5.68	2.28	3.03	1.68	异常	轻度脂肪肝声像。

7.05	4.55	0.87	2.45	1.39	异常	轻度脂肪肝声像
5.18	6.25	1.23	2.92	2.21	异常	肝内囊性回声
5.23	6.79	1.24	3.69	1.78	异常	肝囊肿
4.12	5.84	1.33	3.15	1.49	异常	轻度脂肪肝
5.70	5.74	1.32	3.47	1.31	未见异常	
5.56	6.16	2.09	3.24	1.53	未见异常	
5.28	8.61	2.94	5.00	1.39	异常	轻度脂肪肝声像
4.61	3.26	0.77	1.26	1.50		
5.17	6.50	1.10	3.71	1.48	异常	肝实质回声略增粗，
4.26	4.94	1.49	2.72	1.46	异常	1.轻度脂肪肝声像2.
5.23	6.69	1.31	3.97	1.79	未见异常	
4.80	5.19	0.91	2.76	1.67	异常	轻度脂肪肝声像
4.30	5.32	1.11	2.94	1.40	异常	肝囊肿；胆囊息肉样
11.91	4.02	3.33	1.45	1.30	异常	1、轻度脂肪肝声像2
4.93	6.35	1.29	3.90	1.67	异常	轻度脂肪肝声像
6.54	5.28	2.15	2.47	1.69	未见异常	
4.65	5.23	0.89	2.24	1.87	未见异常	
3.18	5.89	5.28	2.40	1.28	异常	1、脂肪肝声像；2、
15.71	5.54	1.41	3.06	1.53	未见异常	
7.83	5.62	3.92	2.73	1.61	异常	脂肪肝声像，肝囊肿
4.99	4.28	1.31	2.32	1.32	异常	脂肪肝声像
4.72	5.71	1.87	3.06	1.37	异常	胆囊结石
7.58	5.43	2.54	2.81	1.36	异常	1.轻度脂肪肝声像 2
6.58	4.66	1.33	2.58	1.30	异常	轻度脂肪肝声像
6.44	5.33	4.23	2.63	1.35	异常	脂肪肝声像
5.18	4.56	1.34	1.93	1.92	未见异常	
6.06	6.73	2.40	3.85	1.42	异常	脂肪肝声像
4.96	3.22	1.52	1.37	1.22	异常	肝右叶稍高回声结节
9.40	9.70	19.82	2.07	1.02	异常	轻度脂肪肝声像、胆
3.94	6.00	2.07	3.21	1.82	未见异常	
5.62	5.67	1.41	3.05	1.48	异常	胆囊息肉样病变
5.63	8.15	1.20	3.67	2.65	未见异常	
5.85	7.87	1.64	4.94	1.56	异常	轻度脂肪肝声像
5.81	3.74	2.46	1.60	1.32	未见异常	
5.10	5.86	1.48	3.03	1.58	未见异常	
4.63	6.67	3.82	3.11	1.70	异常	肝囊肿
4.86	6.06	1.22	3.01	2.04	异常	轻度脂肪肝声像
4.31	5.75	2.50	3.06	1.50	异常	轻度脂肪肝声像
5.80	4.00	1.34	1.94	1.38	未见异常	
4.48	6.67	1.74	4.21	1.56	异常	1、脂肪肝声像；2、
5.80	5.30	1.79	3.00	1.15	异常	轻度脂肪肝声像
4.87	4.65	0.80	2.12	1.62	异常	胆总管稍扩张
5.28	5.12	2.03	2.91	1.23	未见异常	
4.87	4.59	1.50	2.30	1.41	未见异常	
5.35	6.76	1.14	4.23	1.67	异常	脂肪肝声像
5.60	6.55	1.50	3.35	1.97	异常	轻度脂肪肝
4.47	6.35	2.25	3.07	1.85	未见异常	
4.35	5.14	1.91	2.48	1.49	未见异常	
4.67	5.36	4.30	2.58	1.29	异常	脂肪肝声像
5.29	4.07	0.92	2.21	1.33	未见异常	
4.86	5.42	1.07	3.03	1.66	异常	轻度脂肪肝声像

4.58	5.63	1.46	3.05	1.50	未见异常	
4.94	6.14	4.77	2.85	1.23	未见异常	
4.36	5.91	2.20	2.80	1.69	未见异常	
4.71	5.57	0.97	2.27	2.11	异常	胆囊管扩张
5.13	5.56	1.37	2.92	1.50	未见异常	
4.95	3.87	1.89	1.63	1.47	异常	脂肪肝声像
5.01	4.36	1.26	1.96	1.63	未见异常	
4.96	7.02	1.63	3.79	1.69	异常	肝内囊性回声（考虑
5.15	5.26	2.56	2.57	1.33	未见异常	
5.28	3.56	1.23	1.66	1.12	未见异常	
4.69	6.93	1.24	3.25	2.32	未见异常	
4.80	5.43	1.81	2.91	1.06	未见异常	
5.35	8.06	0.84	4.87	2.23	未见异常	
6.25	4.63	1.06	2.29	1.51	异常	轻度脂肪肝声像
5.57	6.38	1.06	2.50	2.31	未见异常	
4.73	6.05	1.39	3.14	1.55	异常	轻度脂肪肝声像
5.44	5.13	1.97	2.73	1.47	异常	轻度脂肪肝声像
4.42	4.63	1.41	2.51	1.46	异常	1、肝囊肿。2、胆囊
6.05	7.58	1.41	3.89	2.20	异常	肝内小囊性回声（考
6.43	4.68	2.66	2.34	1.33	未见异常	
4.62	4.59	1.32	1.87	1.91	异常	1、肝囊肿；2、胆囊
10.35	4.52	0.71	2.26	1.67	异常	肝胆胰彩超：脂肪肝
7.00	1.19	1.43	2.13	1.19	异常	脾大
5.17	7.21	1.44	3.54	2.25	异常	1.轻度脂肪肝声像 2
5.88	5.71	0.95	2.89	2.01	未见异常	
5.45	6.58	1.41	3.43	1.77	异常	肝内强回声斑，考虑
4.97	2.63	0.94	1.07	1.09	异常	胆囊结石
5.19	6.99	1.91	3.74	1.65	异常	轻度脂肪肝
5.12	5.47	2.24	2.93	1.45	异常	轻度脂肪肝声像
6.76	4.28	1.31	2.21	1.58	未见异常	
5.21	4.46	1.99	2.31	1.20	未见异常	
5.38	6.19	1.91	3.46	1.39	未见异常	
4.62	5.19	3.25	2.42	1.32	异常	脂肪肝声像，胆囊内
5.52	4.41	1.39	2.11	1.57	未见异常	
4.90	6.31	1.52	3.48	1.56	未见异常	
4.55	6.31	1.17	2.92	1.90	异常	腹部B超：肝囊肿
5.02	6.68	2.14	3.55	1.66	异常	轻度脂肪肝
4.90	6.56	0.97	3.96	1.60	异常	腹部B超：轻度脂肪[illegible]
5.38	7.97	8.97	1.73	1.52	异常	胆囊内强回声
5.38	6.02	2.62	2.95	1.58	异常	肝胆脾胰B超：肝内[illegible]
11.94	5.62	1.22	3.12	1.63	异常	轻度脂肪肝声像
8.26	7.30	1.87	4.08	1.82	异常	肝囊肿
5.79	3.82	1.58	1.92	1.22	未见异常	
4.11	5.04	0.64	2.17	1.97	未见异常	
5.39	4.87	3.74	2.21	1.30	未见异常	
4.99	6.60	1.51	3.81	1.69	未见异常	
4.30	5.73	0.91	3.40	1.50	异常	肝内强回声斑（未除
5.36	49.00	2.00	2.90	1.68	异常	1.轻度脂肪肝2.胆囊
5.28	4.33	2.60	2.12	1.34	未见异常	
4.36	4.43	5.02	1.75	1.20	未见异常	
8.23	3.47	1.89	1.28	1.30	异常	轻度脂肪肝

5.54	4.08	2.45	2.02	1.16	异常	肝右叶低回声区
5.87	8.06	1.76	4.85	1.72	异常	轻度脂肪肝声像
11.15	6.24	1.34	3.52	1.66	异常	1.轻度脂肪肝 2.脾[illegible]
4.89	5.42	0.64	2.51	2.04	异常	考虑胆囊多发结石。
5.34	4.45	1.00	2.13	1.48	异常	脂肪肝，胆囊区强回
4.79	6.00	1.35	3.11	1.55	未见异常	
7.32	4.49	2.39	2.13	1.44	未见异常	
5.40	7.66	1.32	3.89	2.04	异常	肝内强回声斑：考虑
9.68	8.70	2.73	4.88	1.76	异常	脂肪肝声像
5.26	5.16	1.21	3.15	1.21	异常	轻度脂肪肝
6.45	6.15	2.96	3.16	1.84	异常	腹部B超：脂肪肝声[illegible]
6.26	6.08	2.69	3.13	1.47	异常	1.轻度脂肪肝声像2.
5.49	4.73	1.38	2.15	1.59	异常	肝囊肿
4.92	5.02	1.13	2.28	1.88	未见异常	
5.09	7.09	1.07	3.46	2.09	异常	腹部B超：肝多发囊[illegible]
5.11	7.18	4.89	3.45	1.41	异常	轻度脂肪肝声像
4.61	6.32	2.09	3.55	1.74	未见异常	
4.88	7.62	1.59	4.36	1.75	未见异常	
4.84	5.98	1.35	3.16	1.80	未见异常	
17.73	6.95	2.48	4.09	1.75	异常	脂肪肝声像。
4.63	5.79	1.02	3.25	1.71	异常	腹部B超：脂肪肝声[illegible]
6.79	8.09	2.93	4.71	1.80	异常	腹部B超：脂肪肝声[illegible]
4.60	6.24	1.77	3.49	1.42	异常	胆囊多发结石
6.93	6.24	0.89	3.74	1.45	异常	胆囊壁稍毛糙
6.24	5.28	0.86	3.00	1.69	异常	胆囊息肉样病变
5.71	7.15	1.64	4.04	1.82	未见异常	
6.40	6.17	1.52	3.40	1.79	未见异常	
7.44	6.30	1.58	3.52	1.68	异常	脂肪肝声像
5.89	5.09	3.70	2.42	1.27	异常	轻度脂肪肝声像
4.76	5.28	0.73	1.65	2.78	异常	胆囊结石
5.15	4.84	1.17	2.82	1.17	未见异常	
5.02	7.17	1.25	4.74	1.45	异常	1.脂肪肝声像 2.肝[illegible]
4.19	4.42	1.51	2.08	1.38	未见异常	
5.05	6.07	1.16	2.50	2.07	未见异常	
4.93	5.41	1.47	3.01	1.38	异常	脂肪肝声像
5.88	4.68	1.54	2.45	1.15	未见异常	
4.44	4.27	1.50	2.25	1.25	未见异常	
6.23	4.60	0.70	1.84	1.90	未见异常	
4.67	5.44	2.26	2.98	1.27	异常	脂肪肝声像
4.94	5.38	1.38	2.87	1.68	异常	1，肝囊肿，2肝内强
5.96	7.30	3.23	4.23	1.57	异常	脂肪肝声像
5.07	5.73	1.66	3.13	1.29	异常	轻度脂肪肝声像
5.52	5.34	1.17	2.47	1.86	未见异常	
6.07	7.08	2.13	3.93	1.75	未见异常	
4.45	6.24	5.43	2.45	1.38	异常	轻度脂肪肝声像
4.60	5.36	2.06	2.87	1.37	未见异常	
7.36	4.42	1.08	2.33	1.42	异常	胆囊结石
5.24	4.69	2.36	2.29	1.58	未见异常	
7.31	7.43	4.30	3.72	1.90	异常	轻度脂肪肝声像
4.25	4.36	0.71	2.04	1.78	异常	1、肝多发囊肿；2、
5.56	5.48	1.99	2.92	1.44	异常	轻度脂肪肝

5.36	5.91	3.18	3.02	1.48	异常	胆囊结石；轻度脂肪
4.95	5.23	2.15	2.55	1.45	异常	胆囊壁稍强光点
4.59	6.37	1.26	3.82	1.32	异常	轻度脂肪肝声像
6.16	6.85	1.85	3.95	1.58	异常	胆囊息肉样病变
5.21	5.64	1.25	2.97	1.49	未见异常	
5.17	4.40	0.77	1.88	1.78	未见异常	
4.74	5.99	2.31	3.00	1.73	异常	脂肪肝声像
5.76	6.84	2.90	3.27	1.82	未见异常	
5.29	6.23	0.92	2.77	2.26	异常	肝内强回声斑：考虑
4.75	5.12	2.51	2.79	1.47	异常	1、脂肪肝声像；2、
7.04	8.23	8.06	2.25	1.66	未见异常	
5.31	6.26	1.88	3.59	1.62	异常	1、脂肪肝声像；2、
6.37	7.08	2.22	3.69	1.65	异常	肝小囊肿
4.71	4.47	1.29	2.21	1.62	未见异常	
4.89	5.27	0.92	2.22	2.09	未见异常	
5.67	8.78	3.82	4.76	1.68	异常	轻度脂肪肝声像
7.49	5.91	1.12	3.55	1.36	未见异常	
4.85	3.81	2.08	1.72	1.26	未见异常	
4.91	5.09	1.48	2.73	1.62	异常	胆囊内强回声
5.36	6.71	0.78	3.32	2.27	未见异常	
5.54	6.91	1.24	3.44	2.04	未见异常	
5.19	6.45	2.47	3.68	1.59	未见异常	
4.67	4.48	1.37	2.00	1.59	未见异常	
6.48	5.28	4.81	2.07	1.39	异常	轻度脂肪肝
4.51	5.08	2.44	2.35	1.70	异常	轻度脂肪肝声像，胆
5.07	8.68	1.28	4.37	2.31	未见异常	
4.59	5.47	1.44	3.46	1.36	异常	脂肪肝声像
5.31	6.53	1.42	3.14	2.25	未见异常	
5.81	6.36	1.66	3.30	2.01	异常	胆囊强回声团，结石
4.25	4.61	0.80	1.61	2.24	未见异常	
4.52	4.18	1.98	1.92	1.39	未见异常	
4.23	5.35	0.95	3.13	1.53	未见异常	
6.60	3.63	1.53	1.82	1.27	未见异常	
5.51	4.95	1.08	2.22	1.87	未见异常	
4.60	5.68	2.81	2.86	1.61	未见异常	
6.60	5.46	1.46	3.01	1.49	未见异常	
4.90	7.70	2.46	4.23	2.12	异常	腹部B超：肝囊肿
5.03	5.37	0.90	2.83	1.78	未见异常	
4.92	4.51	1.58	2.04	1.39	未见异常	
4.79	1.06	3.96	1.74	1.54	未见异常	
6.47	5.75	1.69	3.36	1.31	异常	肝囊肿
5.29	7.26	3.09	3.92	1.65	异常	腹部B超：轻度脂肪
8.07	5.67	1.70	3.12	1.48	异常	脂肪肝声像
6.52	6.23	1.64	3.67	1.58	未见异常	
4.57	5.89	2.48	3.08	1.29	未见异常	
5.86	4.26	1.58	2.10	1.51	异常	脂肪肝声像
6.46	5.33	0.56	2.61	1.84	未见异常	
6.39	6.19	1.72	3.13	1.98	未见异常	
5.26	4.97	2.09	2.57	1.56	异常	1、脂肪肝声像 2、
7.43	5.43	1.68	2.77	1.73	异常	脂肪肝声像
6.22	4.62	2.54	2.21	1.31	异常	胆囊切除术后声像改

4.33	6.23	0.85	2.40	2.24	异常	胆囊结石
4.80	5.23	0.75	2.80	1.61	异常	腹部B超：1.脂肪肝声
4.62	4.19	0.47	1.55	1.97	未见异常	
6.14	6.98	1.92	3.75	1.99	未见异常	
6.21	5.79	0.74	2.99	1.99	异常	1、胆囊壁多发稍强回
4.74	6.05	1.18	3.35	1.85	未见异常	
5.05	5.75	1.42	3.32	1.44	未见异常	
5.17	5.89	1.44	3.15	1.67	异常	腹部B超：1、脂肪肝
5.48	5.42	1.39	2.98	1.54	未见异常	
4.93	6.52	2.21	3.84	1.36	异常	腹部B超：1.脂肪肝声
5.51	5.85	1.92	3.46	1.44	异常	轻度脂肪肝声像。
5.38	4.69	1.40	2.56	1.44	异常	轻度脂肪肝声像
4.63	7.39	1.44	3.95	2.03	未见异常	
4.51	6.36	2.82	2.96	1.72	未见异常	
9.70	6.06	0.83	2.97	1.92	异常	肝胆脾胰B超：轻度脂
4.96	5.67	2.06	2.97	1.44	异常	肝胆脾胰B超：轻度脂
5.59	6.94	5.22	2.73	1.45	异常	肝囊肿
4.26	7.42	1.97	4.44	1.62	未见异常	
4.12	6.01	0.70	3.14	1.98	异常	肝囊肿
4.21	6.79	2.21	3.92	1.59	未见异常	
5.08	5.98	0.77	2.45	2.21	异常	胆囊结石
5.21	5.85	1.75	3.16	1.36	异常	肝右叶低回声结节，
5.47	7.49	1.60	3.61	2.08	未见异常	
4.11	4.18	1.43	1.89	1.41	异常	胆囊稍增大，胆囊结
7.82	4.62	1.97	2.51	1.21	异常	脂肪肝声像
4.51	6.15	1.28	3.41	1.80	未见异常	
6.57	5.34	0.96	3.01	1.52	未见异常	
4.23	5.75	1.91	3.32	1.26	异常	1、轻度脂肪肝声像2
5.82	5.91	1.98	3.20	1.29	异常	1、脂肪肝声像，2、
5.26	5.82	1.61	3.10	1.46	未见异常	
7.85	7.05	1.32	4.04	2.13	异常	轻度脂肪肝
4.97	5.55	2.34	2.70	1.33	异常	肝囊肿
4.47	6.56	1.86	3.79	1.66	异常	脂肪肝声像 胆囊内强
3.98	4.63	2.71	2.36	1.06	未见异常	
4.57	6.06	1.67	3.13	1.85	异常	肝小囊肿；肝内强回
5.80	5.82	1.09	3.26	1.71	异常	1肝实质回声增粗；2
6.10	7.68	1.41	4.71	1.73	异常	轻度脂肪肝声像。
6.39	5.34	2.66	2.72	1.34	异常	脂肪肝声像；肝内囊
10.54	7.25	2.44	4.00	1.84	未见异常	
5.06	6.31	1.87	3.67	1.33	未见异常	
5.09	5.98	2.74	3.31	1.45	异常	轻度脂肪肝声像
4.46	7.70	2.76	5.09	1.52	未见异常	
4.71	6.22	4.50	2.81	1.38	异常	轻度脂肪肝声像
5.28	5.41	1.71	2.91	1.42	异常	脂肪肝声像
4.40	6.83	1.35	3.69	2.06	异常	1肝囊肿声像2脂肪肝
4.15	6.09	2.18	3.27	1.74	异常	1脂肪肝声像2肝左叶
5.06	5.63	2.08	3.06	1.45	异常	肝内强回声斑：考虑
5.72	5.06	2.95	2.64	1.26	未见异常	
5.79	5.13	0.93	2.15	1.97	异常	胆囊结石
6.84	4.59	1.07	2.84	1.03	未见异常	
5.77	5.98	2.23	2.81	1.55	未见异常	

5.54	5.07	1.24	2.91	1.31	未见异常	
6.10	6.35	1.28	3.77	1.55	异常	脂肪肝声像
5.92	5.84	0.78	3.03	1.69	异常	腹部B超：轻度脂肪丿
5.30	5.87	1.89	3.13	1.53	异常	轻度脂肪肝声像
19.43	7.04	0.94	3.52	1.94	未见异常	
5.53	4.28	1.69	2.09	1.42	未见异常	
4.92	5.41	2.53	2.72	1.42	异常	肝内强回声斑
4.34	4.93	0.95	1.59	2.35	异常	腹部B超：胆囊结石
6.99	6.69	1.44	3.16	1.96	异常	胆偏大声像
6.37	4.21	5.82	2.11	1.45	异常	1、脂肪肝声像；2、
4.46	5.05	0.63	2.66	1.51	未见异常	
5.16	7.52	1.59	3.89	2.05	未见异常	
5.09	3.89	1.21	1.66	1.37	未见异常	
4.51	7.20	2.79	3.72	1.66	异常	1、脂肪肝声像； 2、
6.91	4.75	2.84	2.19	1.45	异常	轻度脂肪肝声像
5.29	4.85	1.28	2.68	1.39	未见异常	
4.88	4.81	0.79	2.39	1.53	未见异常	
4.67	6.91	0.68	3.66	2.44	未见异常	
4.97	6.14	5.51	2.29	1.38	异常	1.脂肪肝声像 2.丿
7.36	4.95	1.88	2.88	1.45		
6.33	6.50	2.03	3.56	1.60	异常	轻度脂肪肝
8.78	5.09	2.85	2.60	1.46	异常	脂肪肝声像
5.04	6.81	2.09	3.77	1.71	异常	轻度脂肪肝声像
4.36	5.81	2.01	2.64	2.19	未见异常	
5.17	9.31	1.66	4.12	2.97	未见异常	
5.47	7.90	1.77	4.38	2.11	异常	轻度脂肪肝声像
5.82	7.05	2.31	3.75	1.70	未见异常	
7.28	5.28	0.64	2.62	1.79	异常	肝囊肿
5.79	5.74	1.29	2.96	1.67	未见异常	
7.06	5.59	2.36	2.76	1.45	异常	脂肪肝声像，肝内强
5.97	6.30	1.46	4.08	1.03	未见异常	
4.87	6.05	1.20	2.97	2.01	未见异常	
5.07	5.98	4.48	2.42	1.63	异常	脂肪肝声像
5.70	6.10	1.37	3.58	1.59	未见异常	
7.92	6.95	2.17	3.60	1.80	异常	轻度脂肪肝声像
5.41	4.76	2.09	2.47	1.24	异常	轻度脂肪肝声像；
5.09	5.12	3.28	2.53	1.44	异常	胆囊息肉样病变。
5.72	8.70	1.48	4.92	1.98	未见异常	
5.06	6.59	1.30	3.05	2.42	异常	胆囊息肉样病变
4.85	6.81	3.13	3.46	1.91	异常	肝内低回声结节
4.70	4.04	0.96	2.12	1.44	异常	肝胆胰彩超：肝囊肿
5.17	6.15	1.11	3.46	1.88	未见异常	
7.60	5.81	1.81	3.41	1.46	未见异常	
10.60	3.74	2.52	1.50	1.39	异常	轻度脂肪肝声像
6.55	6.24	2.24	3.03	1.71	异常	轻度脂肪肝声像
4.47	5.65	0.96	2.86	1.74	异常	肝囊肿
4.74	5.37	1.71	2.72	1.72	异常	胆囊结石
4.72	5.47	2.29	2.29	1.51	未见异常	
4.66	4.68	2.50	2.39	1.23	异常	轻度脂肪肝声像，肝
4.15	7.05	2.35	4.00	1.55	异常	轻度脂肪肝
5.53	5.26	1.39	2.78	1.50	未见异常	

5.37	4.26	0.82	2.05	1.59	未见异常	
4.74	7.18	2.01	3.87	1.69	异常	肝内强回声斑:考虑钅
4.74	6.08	1.71	3.31	1.67	未见异常	
5.04	3.96	1.20	1.76	1.44	未见异常	
5.27	5.59	6.80	2.72	1.54	异常	轻度脂肪肝
5.65	6.83	1.58	3.55	1.70		
9.01	7.86	1.62	4.43	1.91	未见异常	
7.77	5.74	4.35	2.43	1.37		
4.34	6.04	1.50	3.35	1.57	未见异常	
5.31	4.59	1.27	2.35	1.58	未见异常	
6.78	3.34	0.70	1.29	1.52	未见异常	
6.96	5.80	1.74	2.74	1.78	异常	肝囊肿
7.22	6.57	2.62	3.57	1.76	未见异常	
4.75	6.74	1.05	3.45	1.91	异常	1.肝内囊性回声2.肝
4.45	4.84	0.91	1.91	2.03	未见异常	
4.88	5.19	0.98	2.88	1.47	未见异常	
4.43	4.57	3.51	1.92	1.32	未见异常	
5.47	4.52	1.64	2.15	1.41	未见异常	
4.95	10.35	1.90	6.10	2.00	未见异常	
7.61	5.09	2.05	2.74	1.19	异常	1、脂肪肝声像；2、
3.87	4.78	1.22	2.37	1.22	未见异常	
6.36	6.33	2.27	3.30	1.46	未见异常	
5.63	5.42	1.04	2.54	1.74	未见异常	
5.09	6.43	4.26	2.83	1.50	未见异常	
5.04	7.91	0.99	3.70	2.45	异常	肝内强回声斑：考虑
6.91	4.55	3.63	2.05	1.21	异常	轻度脂肪肝声像，胆
4.86	4.66	0.81	1.90	1.71	异常	肝胆脾胰B超：肝内引
5.01	6.13	2.74	3.08	1.33	异常	肝囊肿
6.17	4.49	2.28	2.03	1.34	异常	胆囊息肉样病变
10.39	6.62	1.96	4.16	1.41	异常	轻度脂肪肝声像
4.54	6.04	2.47	2.99	1.79	未见异常	
5.35	6.43	1.74	2.69	2.41	未见异常	
4.72	4.95	2.07	2.57	1.27	未见异常	
5.68	5.60	1.77	2.11	2.46	异常	轻度脂肪肝
4.43	4.82	0.74	2.69	1.20	异常	胆囊未探及 胆总管_
3.85	7.27	2.87	4.04	1.55	异常	轻度脂肪肝
4.94	5.95	0.78	2.75	1.94	未见异常	
13.24	7.43	2.95	3.96	1.74	异常	轻度脂肪肝
4.97	5.01	1.04	2.49	1.65	未见异常	
4.99	3.83	2.19	1.92	1.03	未见异常	
5.23	5.62	1.31	2.87	1.77	未见异常	
5.81	5.25	1.32	2.59	1.87	未见异常	
5.00	5.32	1.78	2.81	1.51	异常	肝多发囊性回声（考
5.08	5.17	4.74	1.95	1.55	异常	脂肪肝声像
5.65	4.20	1.60	2.20	1.09	异常	脂肪肝声像
7.44	9.35	1.85	6.10	1.49	异常	肝内强回声：钙化灶
4.94	5.42	1.55	2.94	1.48	异常	轻度脂肪肝声像
4.68	5.30	1.23	2.93	1.39	未见异常	
4.66	8.14	3.73	4.22	1.42	异常	肝脾内脏转位
6.02	4.98	1.13	2.99	1.38	未见异常	
6.35	5.64	1.18	3.34	1.61	异常	肝脏实质回声增粗、

4.87	5.16	0.68	2.64	1.65	异常	轻度脂肪肝声像
5.55	6.41	1.72	3.39	1.86	异常	脂肪肝声像
5.22	6.72	2.11	3.55	1.90		
4.18	5.33	5.02	2.13	1.31	未见异常	
4.79	4.81	1.65	2.36	1.69	未见异常	
7.06	7.22	3.17	3.98	1.41	异常	脂肪肝声像
4.97	5.72	0.81	2.81	1.81	未见异常	
5.04	5.44	0.92	1.47	2.79	异常	脂肪肝声像
4.83	4.61	3.79	1.64	1.35	异常	胆囊多发性结石
5.56	5.89	1.17	3.62	1.58	异常	轻度脂肪肝声像
8.70	6.19	0.79	3.60	1.80	未见异常	
5.85	7.66	2.13	4.20	1.86	未见异常	
5.06	7.14	1.18	4.36	1.81	异常	肝囊肿
18.94	7.33	1.90	4.25	1.83	异常	脂肪肝声像
5.72	7.99	2.48	4.33	1.79	未见异常	
5.74	6.34	0.73	2.99	2.05	异常	肝囊肿
5.64	7.13	2.98	3.75	1.52	异常	左心房心律?
4.76	5.13	0.94	2.54	1.93	异常	肝实质回声稍粗
6.97	5.72	2.62	3.34	1.36		
4.11	5.44	0.64	2.41	2.00	异常	胆囊强回声团，考虑
5.31	7.34	1.27	4.44	1.65	未见异常	
4.67	4.63	1.95	2.19	1.09	异常	肝胆脾胰B超：1.轻
9.15	7.59	7.86	3.97	1.45	未见异常	
5.40	4.24	1.07	2.03	1.30	异常	1、轻度脂肪肝声像；
4.92	5.79	1.45	3.11	1.38	异常	脂肪肝声像
4.39	5.07	4.05	2.32	1.34	异常	脂肪肝声像
5.63	7.15	2.22	4.34	1.48	异常	轻度脂肪肝
4.36	6.21	1.02	3.24	1.84	未见异常	
4.91	6.68	2.72	3.57	1.83	异常	轻度脂肪肝声像
6.65	5.77	5.13	1.93	1.62	异常	轻度脂肪肝声像
9.46	4.66	1.43	2.23	1.57	未见异常	
5.26	5.67	1.09	2.60	2.15	异常	胆囊息肉样病变
5.16	4.14	1.47	2.01	1.35	未见异常	
6.84	7.91	1.13	4.37	1.81	未见异常	
5.26	3.03	0.87	1.41	1.21	异常	胆囊强回声团，考虑
6.03	6.51	1.77	3.26	1.53	未见异常	
4.03	6.42	2.08	3.28	1.59	未见异常	
5.22	5.35	1.35	2.44	1.79	未见异常	
5.40	6.43	1.97	3.22	1.61	未见异常	
5.24	6.02	5.21	2.43	1.48	异常	1脂肪肝声像。2胆囊
4.52	6.10	2.33	3.35	1.40	异常	轻度脂肪肝声像
6.53	6.06	4.00	2.82	1.53	异常	肝多发囊肿
5.54	5.57	0.91	2.83	1.81	异常	肝内强回声斑（考虑
5.94	3.91	1.01	1.30	1.86	未见异常	
5.22	5.91	0.99	3.23	1.63	未见异常	
5.03	5.94	0.91	3.32	1.78	异常	脂肪肝声像
5.05	5.70	0.99	2.97	1.54	异常	肝小囊肿
5.09	7.56	3.21	4.04	1.78	未见异常	
4.92	5.79	0.72	2.61	1.76	未见异常	
5.38	6.11	1.51	3.42	1.77	异常	肝右叶稍高回声结节
9.12	7.08	2.71	3.88	1.67	异常	1、脂肪肝声像； 2、

5.00	7.27	1.61	4.38	1.42	未见异常	
4.51	5.50	1.62	2.95	1.70		
6.21	7.23	8.67	1.68	1.21	异常	轻度脂肪肝声像
6.84	6.30	4.76	2.69	1.67	异常	胆总管稍扩张，结合
4.59	4.09	1.18	2.29	1.23	未见异常	
7.85	4.95	0.95	3.09	1.36	异常	1、脂肪肝声像2、胆
4.43	3.90	1.05	1.42	1.84		
5.28	5.56	3.18	2.92	1.43	未见异常	
6.45	10.13	3.53	5.52	1.55	异常	轻度脂肪肝声像
10.91	6.06	1.84	3.48	1.45	异常	腹部B超：1.轻度脂肪
3.95	7.44	2.19	4.11	1.97	未见异常	
6.64	1.31	5.36	2.56	1.75	异常	轻度脂肪肝声像
8.12	3.31	1.69	1.43	1.14	异常	1轻度脂肪肝声像 2
5.42	6.13	1.23	3.97	1.28	异常	胆囊息肉样病变
4.79	8.24	1.57	4.79	1.76	未见异常	
4.87	6.65	1.50	3.11	2.17	异常	肝囊肿；脂肪肝
4.66	5.43	1.13	2.95	1.51	未见异常	
4.78	7.61	2.01	4.13	1.81	异常	肝囊肿
5.03	5.42	1.67	3.07	1.39	异常	肝内强回声斑（未初
6.02	4.94	1.43	2.25	1.82	未见异常	
5.54	4.83	2.73	2.56	1.10	异常	1、脂肪肝声像； 2、
4.64	5.22	1.42	2.50	1.96	未见异常	
5.10	8.73	3.78	4.25	2.12	异常	肝内强回声斑（考虑
5.01	5.84	1.03	3.05	1.93	未见异常	
5.02	5.78	1.05	3.10	1.46	未见异常	
4.54	5.12	10.04	2.88	1.15	异常	胆囊结石
4.59	6.19	1.98	3.33	1.62	异常	脂肪肝声像
7.32	6.77	1.95	3.71	1.70	未见异常	
5.11	6.28	1.20	3.05	2.04	未见异常	
4.99	5.75	1.58	2.97	1.68	未见异常	
6.71	7.82	2.35	4.51	1.53	异常	脂肪肝声像
5.18	5.37	1.51	2.89	1.55	异常	轻度脂肪肝声像
4.91	5.90	1.92	3.14	1.47	异常	脂肪肝声像
5.34	5.41	1.55	2.17	2.01	未见异常	
5.24	6.99	1.65	3.62	2.13	未见异常	
5.78	6.39	1.45	3.96	1.46	异常	腹部B超：脂肪肝声
5.42	8.49	3.34	4.66	1.58	异常	脂肪肝声像
5.40	5.97	0.93	3.09	1.70	未见异常	
5.01	4.53	1.06	1.91	1.91	未见异常	
8.14	6.54	1.19	3.66	1.84	未见异常	
5.46	5.85	1.21	3.36	1.53	未见异常	
4.50	5.72	1.79	3.02	1.53	未见异常	
4.45	4.90	2.04	2.23	1.38	未见异常	
6.51	6.80	2.04	3.62	1.58	异常	脂肪肝声像
4.61	6.58	3.00	3.19	1.62	未见异常	
4.82	5.71	2.34	3.23	1.36	未见异常	
5.56	6.68	1.54	3.41	1.65	异常	1、肝实质回声增粗2
5.54	4.51	0.90	2.04	1.70	异常	轻度脂肪肝
6.80	5.30	2.12	2.80	1.48	异常	轻度脂肪肝，肝囊息
5.56	6.19	1.97	3.38	1.70		
5.31	5.76	3.13	3.00	1.36	未见异常	

	6.17	2.83	0.94	1.28	0.99	异常	门静脉局部扩张
	19.81	5.48	2.54	2.44	1.69	未见异常	
	7.52	6.58	3.37	3.40	1.43		
	4.51	6.30	1.87	3.71	1.24	未见异常	
	4.80	7.01	1.68	3.78	1.89	未见异常	
	7.32	5.47	2.16	1.94	1.13	异常	脂肪肝声像
	8.62	6.06	1.91	3.57	1.56	异常	脂肪肝声像
	10.55	3.93	1.02	1.70	1.67	未见异常	
	5.54	6.13	3.11	3.24	1.60	异常	腹部B超：轻度脂肪
	5.17	6.01	2.36	3.37	1.55	未见异常	
	5.35	6.68	1.44	2.36	2.99	异常	肝囊肿
	6.04	5.69	0.52	2.67	2.18	未见异常	
	4.95	5.14	0.76	2.43	1.79	异常	肝内强回声结节(血
	4.90	6.35	1.92	3.46	1.48	异常	脂肪肝声像
	5.31	6.08	7.00	1.47	1.46	未见异常	
	5.21	6.65	1.73	4.00	1.48	未见异常	
	4.44	6.22	1.45	3.69	1.38	异常	轻度脂肪肝声像。
	4.57	6.27	2.08	3.43	1.59	异常	轻度脂肪肝声像
	4.70	7.76	1.49	4.90	1.62	未见异常	
	4.67	4.85	1.14	2.49	1.61	未见异常	
	5.22	9.44	3.29	5.52	1.80	异常	轻度脂肪肝声像
	4.70	5.73	0.84	2.86	1.82	异常	肝内强回声斑：考虑
	5.47	6.90	1.27	3.67	1.72	未见异常	
	7.82	6.99	1.27	3.69	1.99	未见异常	
	4.57	5.50	3.01	2.41	1.71	异常	1.实质光点回声增粗
	6.59	6.82	1.36	3.97	1.68	异常	脂肪胺，肝囊肿
	4.98	7.65	1.58	4.22	1.87	异常	1.脂肪肝声像 2.肝
药酒	5.17	6.20	0.94	2.61	2.37	异常	肝实质回声稍粗，肝
	5.70	6.26	2.57	3.31	1.62	异常	轻度脂肪肝声像
	6.54	9.71	1.08	5.10	2.75	异常	肝内囊性回声（考虑
	4.84	4.64	1.73	2.24	1.62	异常	胆囊息肉样病变
	5.40	3.36	0.75	1.29	1.55	异常	胆囊多发结石。
	6.79	4.05	1.91	1.94	1.36	异常	轻度脂肪肝声像
	5.80	6.96	1.55	4.13	1.38	未见异常	
	4.68	4.60	0.69	2.04	1.63	异常	胆囊内强回声（考虑
	5.29	6.63	1.04	3.69	1.91	未见异常	
	6.04	8.47	2.84	4.72	1.61	异常	轻度脂肪肝声像
	5.21	5.80	0.80	3.17	1.75	未见异常	
	6.12	4.67	2.33	2.15	1.55	异常	胆囊结石
	5.34	6.17	0.72	3.19	1.82	未见异常	
	9.40	6.07	2.28	3.57	0.88	异常	脂肪肝声像
	4.46	5.25	1.18	2.31	1.84	异常	轻度脂肪肝
	8.04	5.11	0.91	3.04	1.21	未见异常	
	5.62	5.92	2.36	3.18	1.22	异常	胆囊切除术
	4.21	6.90	5.70	2.62	1.51	异常	脂肪肝声像
	5.32	5.49	0.82	2.13	2.15	未见异常	
	11.34	6.00	2.37	3.31	1.50	异常	脂肪肝声像
	4.41	6.05	1.18	2.24	2.02	未见异常	
	6.03	6.27	2.17	3.20	1.79	异常	脂肪肝声像
	6.99	6.86	2.45	3.82	1.81	异常	轻度脂肪肝；肝囊肿
	4.93	5.11	1.19	2.69	1.75	未见异常	

5.83	4.92	0.62	2.01	2.00	未见异常	
5.79	3.42	3.33	1.53	0.88	异常	脂肪肝声像
9.66	5.05	2.34	2.64	1.36	异常	脂肪肝声像
5.19	3.83	0.95	1.72	1.52	未见异常	
4.34	4.47	0.47	1.64	2.07		
4.37	6.96	2.32	4.08	1.57	未见异常	
11.32	7.77	8.28	1.61	1.47	异常	脂肪肝声像
4.67	8.16	4.29	4.21	1.48	异常	脂肪肝声像
4.45	4.86	2.72	2.43	1.38	未见异常	
5.84	5.69	0.76	2.75	1.94	异常	轻度脂肪肝
4.29	5.00	1.53	2.23	1.88	未见异常	
5.37	6.00	1.64	3.21	1.59	异常	胆囊内强回声（考虑
5.19	6.01	2.55	3.19	1.46	异常	胆囊结石
4.72	5.59	6.98	2.57	1.67	异常	肝内多发囊肿
4.45	5.35	1.38	2.68	1.43	异常	轻度脂肪肝
9.72	6.10	1.88	2.91	2.17	异常	1.脂肪肝声像2.胆囊
4.75	6.96	0.61	3.58	2.33	未见异常	
5.40	6.05	2.03	3.30	1.61	未见异常	
6.95	5.89	1.40	2.91	1.87	异常	脂肪肝声像
5.57	8.85	1.72	5.00	1.87	未见异常	
6.63	6.55	2.94	3.55	1.30	异常	肝胆脾胰B超：脂肪
4.75	6.55	0.83	3.30	2.02	未见异常	
5.15	5.02	1.48	2.68	1.54		
13.08	7.96	1.68	4.53	1.73	异常	轻度脂肪肝声像
5.61	5.41	0.92	2.75	1.55	未见异常	
4.81	4.56	0.97	2.14	1.85	未见异常	
4.02	6.15	2.43	3.51	1.41	未见异常	
4.85	5.71	2.81	2.76	1.53	异常	轻度脂肪肝声像
5.15	6.05	1.26	2.95	1.96	未见异常	
5.40	3.97	1.34	1.86	1.45	异常	肝内囊性回声（考虑
4.77	6.43	2.43	3.53	1.47	异常	1、肝囊肿 2、胆囊
4.50	7.39	2.16	4.24	1.93	异常	肝多发囊性回声
5.44	7.04	3.00	3.37	2.07	未见异常	
12.44	3.56	1.04	1.80	1.20	异常	1、轻度脂肪肝声像;
4.66	6.58	1.09	3.48	1.72	异常	肝囊肿
7.65	5.26	2.23	2.58	1.50	异常	轻度脂肪肝
4.30	5.55	2.81	3.11	1.45	异常	脂肪肝声像
4.61	6.22	0.67	3.61	1.57	未见异常	
5.14	6.18	1.63	3.13	1.69	异常	肝右叶稍高回声结节
4.93	5.49	2.19	2.92	1.66	未见异常	
4.76	4.76	1.47	2.38	1.54	异常	脂肪肝
5.16	5.56	1.23	2.67	1.88	未见异常	
4.75	5.44	1.10	2.82	1.68	异常	1、肝囊肿2、胆囊息
5.27	7.57	1.47	4.77	1.53	未见异常	
7.14	4.18	1.93	1.76	1.61	异常	胆囊结石
6.53	5.03	3.02	2.42	1.37	异常	轻度脂肪肝声像；肝
5.45	5.80	1.99	2.87	1.64	异常	轻度脂肪肝声像
5.28	4.43	1.23	2.19	1.46	未见异常	
5.85	5.42	1.62	3.06	1.24	异常	轻度脂肪肝声像
4.60	5.48	0.72	2.17	2.06	未见异常	
5.04	8.24	1.84	5.09	1.69	异常	脂肪肝声像

5.19	7.36	1.16	4.22	1.85	未见异常	
5.70	6.33	1.68	3.07	1.98	异常	肝内囊性回声（考虑
6.93	5.93	1.86	3.14	1.76	异常	脂肪肝，肝右叶片状
7.36	7.43	3.67	3.83	1.51	异常	腹部B超：轻度脂肪肝
6.47	4.62	1.48	1.96	1.62	未见异常	
5.96	6.32	1.85	3.55	1.75	异常	脂肪肝声像。
5.01	7.60	2.51	4.53	1.74	未见异常	
5.03	4.60	0.73	2.35	1.56	异常	肝囊肿
7.50	3.96	0.61	1.87	1.55	异常	胆囊息肉样病变
5.59	8.08	2.09	4.22	2.00	未见异常	
6.60	5.66	1.26	3.11	1.54	异常	轻度脂肪肝声像
4.60	4.68	2.27	2.26	1.48	未见异常	
6.17	6.10	0.77	2.69	2.10	异常	肝内强回声斑
4.50	5.20	0.97	2.55	1.74	异常	轻度脂肪肝
5.06	5.99	1.23	3.43	1.54	异常	脂肪肝声像
7.63	6.85	3.01	3.57	1.66	异常	脂肪肝声像
5.26	6.96	1.44	4.04	1.65	异常	轻度脂肪肝声像
6.06	4.68	1.10	1.93	1.75	异常	肝内外胆管结石并扩
4.87	6.63	1.48	3.74	1.59	未见异常	
9.26	6.78	2.24	3.92	1.61	未见异常	
6.87	7.11	1.91	4.21	1.32	未见异常	
5.99	5.14	1.74	2.87	1.25	未见异常	
7.56	4.17	2.88	2.29	1.08	异常	脂肪肝声像
4.34	6.32	2.31	3.50	1.37	异常	轻度脂肪肝;胆囊壁
5.75	6.16	7.33	1.76	1.38	异常	轻度脂肪肝声像
5.19	6.24	0.68	2.37	2.51	未见异常	
5.98	6.44	2.05	3.71	1.44	异常	肝内强回声斑：考虑
4.65	6.46	1.67	3.05	2.28	异常	胆囊内强回声（考虑
4.64	6.23	1.22	3.79	1.60		
4.86	7.37	1.75	4.21	1.81	未见异常	
4.51	5.76	1.73	3.07	1.64	异常	所示范围内脂肪肝声
5.70	7.01	1.17	4.04	1.91	异常	肝内囊性回声
4.75	7.37	1.71	4.20	1.48	异常	肝内强回声斑，考虑
5.56	7.06	1.47	4.21	1.48	异常	轻度脂肪肝
5.61	5.58	2.59	3.01	1.40	异常	轻度脂肪肝
4.37	6.08	1.43	3.66	1.69		
6.29	5.79	4.65	2.51	1.23	异常	轻度脂肪肝声像
5.46	5.85	3.24	3.00	1.60	未见异常	
7.54	6.09	3.31	2.94	1.75	异常	轻度脂肪肝声像
4.63	4.42	2.00	2.13	1.39	未见异常	
3.79	4.00	1.41	2.16	1.18	异常	腹部B超：1.脂肪肝
5.77	4.68	0.96	2.17	1.69	异常	脂肪肝声像，肝内强
6.49	4.12	1.37	2.06	1.53	异常	胆囊点状稍高回声，
4.59	4.31	1.60	2.49	1.16	未见异常	
4.37	5.09	1.12	2.86	1.21	未见异常	
5.97	5.48	0.78	2.55	1.92	未见异常	
5.26	4.69	1.24	2.52	1.48	异常	轻度脂肪肝声像
7.53	5.45	0.86	2.98	1.30	异常	轻度脂肪肝声像
6.09	6.33	1.60	3.31	1.87	未见异常	
4.65	4.59	0.88	2.22	1.60	未见异常	
4.91	4.71	2.80	2.29	1.42	异常	脂肪肝声像

5.32	6.06	1.14	3.34	1.36	未见异常	
5.13	4.87	1.10	2.59	1.67	异常	胆囊内强回声（考虑
5.03	5.46	1.37	3.15	1.39	异常	轻度脂肪肝
7.14	6.23	0.92	3.95	1.47	异常	轻度脂肪肝声像
5.69	5.04	0.84	2.29	1.91	未见异常	
5.78	6.54	0.91	3.29	2.04	未见异常	
5.58	8.13	3.06	4.71	1.89	异常	轻度脂肪肝声像
6.23	4.53	3.00	2.53	1.24	异常	轻度脂肪肝声像
5.79	5.42	1.37	2.99	1.62	异常	腹部B超：轻度脂肪肝
4.48	5.82	1.83	3.18	1.69	异常	1.胆囊多发息肉样病
4.65	5.61	2.17	3.34	1.43	未见异常	
5.02	4.98	2.66	2.33	1.59	异常	肝内强回声斑：考虑
4.98	5.29	1.03	3.19	1.05	异常	轻度脂肪肝
4.18	6.54	1.87	3.53	1.44	异常	胆囊内强回声（考虑
4.20	3.76	3.11	1.78	1.11	异常	轻度脂肪肝声像
4.33	7.35	2.61	3.98	1.81	未见异常	
4.84	3.91	1.06	1.37	1.85	未见异常	
4.02	5.46	0.97	3.13	1.48	异常	肝囊肿，胆囊结石
8.24	5.77	2.27	2.97	1.47	异常	轻度脂肪肝声像
5.26	5.78	1.03	2.63	1.95	未见异常	
6.09	5.67	2.85	3.05	1.60	异常	轻度脂肪肝声像
4.78	5.01	0.82	1.90	2.24	异常	肝多发囊肿
4.96	6.04	1.30	3.23	1.57	未见异常	
5.38	4.37	2.47	2.15	1.23	未见异常	
5.10	3.80	1.11	1.76	1.31	异常	胆囊息肉样病变
4.94	4.71	2.32	2.41	1.58	异常	脂肪肝声像
7.52	6.32	4.59	2.91	1.69	异常	轻度脂肪肝声像
4.90	7.55	2.22	4.00	1.76	未见异常	
4.84	3.19	1.51	1.34	1.25	未见异常	
5.19	4.97	1.75	2.55	1.47	未见异常	
4.36	5.66	1.44	2.97	1.71	异常	1.肝囊肿2.胆囊结石
4.80	6.39	1.72	3.33	2.01	未见异常	
4.46	5.65	1.75	3.01	1.62	异常	轻度脂肪肝
4.28	5.81	1.64	3.08	1.59	异常	轻度脂肪肝
3.30	4.34	0.66	2.21	1.29	异常	1、轻度脂肪肝声像2
4.58	3.69	2.07	1.72	1.21	异常	肝小囊肿
4.54	7.09	2.74	3.79	1.46	异常	囊内强回声（考虑结
6.66	5.44	2.16	2.93	1.69	异常	脂肪肝声像
4.70	4.89	1.32	1.95	1.96	异常	肝内强回声斑(考虑钅
7.36	6.56	2.06	3.18	2.08	异常	轻度脂肪肝声像
4.90	4.60	3.17	2.14	1.40	未见异常	
4.95	4.24	1.69	2.11	1.12	异常	轻度脂肪肝声像、肝
4.62	7.76	1.47	4.08	2.08	未见异常	
4.11	6.49	1.31	3.54	1.48	未见异常	
5.38	4.14	2.52	1.73	1.21	异常	轻度脂肪肝声像
5.57	5.40	1.61	3.24	1.34		
4.38	6.67	2.18	3.70	1.60	异常	脂肪肝声像
3.66	6.42	3.67	3.15	1.54	异常	脂肪肝声像
11.37	5.14	1.41	2.92	1.15	未见异常	
7.71	7.13	1.09	4.04	1.65	未见异常	
6.21	7.13	1.56	3.33	2.17	未见异常	

4.81	5.00	1.98	2.51	1.36	未见异常	
4.99	4.37	3.33	1.99	1.34	异常	轻度脂肪肝声像,肝囊
4.80	4.89	0.97	2.78	1.50	未见异常	
4.44	5.06	0.77	1.73	2.17	异常	肝内强回声斑
4.73	5.94	2.16	3.14	1.40	异常	所示范围内轻度脂肪
4.83	7.36	1.68	4.18	1.68	异常	轻度脂肪肝声像
5.71	5.98	1.66	3.76	1.49	异常	轻度脂肪肝声像。
4.25	4.42	0.79	2.19	1.58	未见异常	
4.81	7.05	1.99	3.93	1.61	异常	脂肪肝声像；肝左叶
4.85	6.23	1.73	3.78	1.41	未见异常	
4.74	6.31	0.73	3.16	2.10	未见异常	
5.06	5.91	1.50	2.63	2.18	异常	肝囊肿
5.36	6.19	1.42	3.87	1.47	异常	脂肪肝声像。
5.22	6.49	2.23	3.54	1.79	未见异常	
5.36	2.76	0.91	1.07	1.08	未见异常	
8.41	5.74	3.61	2.87	1.56	异常	轻度脂肪肝声像
4.49	6.97	0.92	3.97	1.83	异常	轻度脂肪肝声像
4.82	4.46	0.82	1.99	1.53	未见异常	
4.55	5.23	1.15	2.59	1.64	异常	肝内强回声斑：考虑
6.70	3.87	1.45	1.79	1.39	未见异常	
5.13	6.58	1.17	3.53	1.75	未见异常	
6.06	6.70	1.86	4.09	1.55	未见异常	
5.80	7.41	2.12	3.82	2.04	异常	脂肪肝声像
5.12	6.38	0.85	3.74	1.97	未见异常	
5.32	6.51	0.83	3.32	2.11	异常	胆囊内强回声（考虑
4.22	3.23	1.68	1.41	1.18	异常	肝内强回声斑
5.48	8.04	2.04	4.36	1.74	异常	肝囊肿
4.63	5.34	1.11	2.80	1.67	异常	胆囊结石
7.68	6.56	2.68	3.25	1.56	异常	肝囊肿
6.67	6.31	1.36	3.28	1.51	异常	1、脂肪肝声像2、胆
5.83	9.13	3.03	5.23	2.15	异常	轻度脂肪肝声像
5.43	5.92	1.26	3.44	1.67	异常	脂肪肝声像。
5.34	7.10	2.03	3.71	1.89	未见异常	
4.87	6.24	1.15	2.71	2.01	未见异常	
8.28	7.94	4.31	3.73	1.66	异常	胆囊结石
4.42	5.17	1.25	2.48	1.80	异常	肝实质回声稍增粗
5.59	6.92	2.13	3.78	1.65	异常	脂肪肝声像，肝囊肿
5.81	5.35	3.97	2.42	1.31		
4.87	5.29	3.14	2.74	1.39	异常	肝多发囊肿；轻度脂
5.92	4.79	2.71	2.48	1.19	未见异常	
6.59	4.04	2.20	1.84	1.28	异常	轻度脂肪肝
4.74	4.69	1.19	2.23	1.70	异常	脂肪肝声像
5.44	5.70	2.13	1.60	3.24	异常	胆囊稍高回声结节，
5.00	5.59	1.10	2.36	1.98	异常	肝小囊肿
5.13	5.24	1.03	2.77	1.69	异常	脂肪肝声像
4.93	6.76	2.22	3.53	1.87	异常	脂肪肝声像，胆囊结
4.40	5.13	1.33	2.45	1.77	异常	肝左叶无回声区
6.19	7.72	0.96	3.71	2.12	异常	轻度脂肪肝，胆囊增
5.45	4.53	0.80	2.09	1.69	未见异常	
5.22	4.72	2.33	2.28	1.32	异常	轻度脂肪肝声像
5.62	5.95	1.44	3.28	1.62	异常	轻度脂肪肝声像。

5.08	6.28	1.81	3.54	1.53	异常	胆囊充满型结石声像
6.93	5.74	1.54	2.92	1.64	异常	轻度脂肪肝声像
5.36	4.87	2.15	2.57	1.37	未见异常	
4.59	6.71	2.36	3.85	1.30	异常	轻度脂肪肝，肝内强
5.85	6.90	2.28	3.87	1.79	未见异常	
5.04	6.08	1.35	3.46	1.65	异常	肝囊肿
5.55	5.96	0.84	2.93	1.82	异常	肝囊肿
5.35	4.30	2.39	2.00	1.60	异常	胆囊息肉样病变
4.93	5.60	2.22	2.95	1.60	异常	脂肪肝声像
4.93	7.28	2.00	3.92	1.62	未见异常	
5.56	6.18	0.77	3.53	1.76	未见异常	
6.92	4.96	2.10	2.75	1.22	未见异常	
7.48	7.61	1.55	4.77	1.51	异常	脂肪肝声像。
16.69	6.50	4.51	3.35	1.45	异常	脂肪肝声像。
4.56	5.27	1.27	2.58	1.62	未见异常	
5.65	5.26	1.50	2.33	1.85	未见异常	
4.61	6.14	1.16	2.62	2.28	异常	肝实质性回声稍增粗
4.51	5.65	1.23	3.01	1.85	未见异常	
5.47	7.82	1.23	4.77	1.98		
5.77	7.28	3.29	3.77	1.61	异常	所示范围内脂肪肝声
7.61	4.20	1.04	1.86	1.46	未见异常	
4.41	5.02	0.54	1.91	2.36	未见异常	
4.49	5.80	0.50	2.45	2.33	异常	肝囊肿
4.88	7.57	1.53	4.36	1.82	未见异常	
6.53	4.36	2.31	2.14	1.23	异常	肝肾区囊性回声包块
10.00	6.75	1.31	3.30	1.88	异常	胆囊息肉样病变
6.83	5.69	1.62	3.01	1.64	异常	肝囊肿
7.10	4.42	1.23	2.30	1.33	异常	脂肪肝声像
4.91	6.61	1.32	2.92	2.36	异常	脂肪肝声像
5.20	4.35	1.27	2.27	1.52	异常	1、脂肪肝声像；2、
5.70	4.62	0.50	2.04	1.62	异常	胆囊切除后声像改变
9.27	7.84	1.10	4.30	2.12	未见异常	
5.95	4.36	2.01	2.18	1.35	异常	腹部B超：胆囊壁欠光
6.87	7.86	1.43	5.14	1.65	未见异常	
4.70	6.05	1.96	1.37	3.59	异常	脂肪肝声像
5.99	5.56	2.03	3.07	1.47	异常	脂肪肝声像
9.25	5.60	2.57	2.73	1.42	异常	脂肪肝声像
6.41	7.20	3.57	3.64	1.54	异常	轻度脂肪肝
5.05	5.88	0.75	2.78	1.91	异常	胆囊结石
5.09	3.91	1.28	1.55	1.57	未见异常	
4.80	8.99	1.99	4.61	2.09	异常	肝内强回声斑，考虑
5.66	5.45	0.86	2.77	1.80	未见异常	
4.74	5.86	1.32	2.92	1.82	未见异常	
4.86	4.27	0.75	1.73	1.63	未见异常	
4.79	7.51	8.75	1.63	1.24	未见异常	
5.53	5.60	5.24	2.23	1.20	异常	脂肪肝声像
5.27	8.43	1.85	4.88	1.94	未见异常	
6.10	5.14	3.50	2.58	1.15	异常	脂肪肝声像
5.97	7.32	1.40	3.92	2.02	未见异常	
5.53	4.27	2.68	2.09	1.28	异常	轻度脂肪肝声像
6.47	5.02	1.43	2.49	1.67	异常	肝内强回声斑，考虑

4.49	6.41	1.57	3.69	1.60	异常	胆囊结石
6.86	6.01	2.91	3.00	1.74	异常	脂肪肝声像
4.57	6.83	3.03	3.63	1.42	异常	不均质脂肪肝声像
8.92	4.76	1.17	2.31	1.49	异常	肝内强回声斑：考虑
4.68	9.76	2.24	5.16	2.44	未见异常	
4.71	4.95	1.19	2.54	1.48	未见异常	
5.10	5.84	1.05	2.92	1.84	异常	轻度脂肪肝
13.30	5.17	0.82	2.89	1.57	未见异常	
5.31	4.28	1.20	1.84	1.45	未见异常	
5.41	5.15	1.37	2.44	1.78	未见异常	
6.44	6.20	3.82	3.16	1.63	异常	脂肪肝声像
5.11	6.50	1.73	4.06	1.28	异常	脂肪肝声像
4.70	8.04	2.33	5.04	1.71	异常	脂肪肝声像
4.97	5.71	3.09	2.91	1.31	异常	轻度脂肪肝声像
4.17	5.17	1.97	2.52	1.51	未见异常	
7.21	6.76	1.92	3.94	1.55	未见异常	
4.97	6.62	1.43	3.54	1.79	异常	肝囊肿
4.69	5.52	1.55	2.79	1.47	异常	肝内多发性囊肿，肝
6.74	5.77	1.02	3.37	1.34	异常	1、脂肪肝 2、肝囊
4.99	7.26	1.35	3.96	2.14	未见异常	
6.03	6.46	2.00	3.29	1.82	未见异常	
5.96	7.48	3.75	3.87	1.37	异常	1、脂肪肝声像 2、
5.24	6.94	1.60	3.98	1.78	异常	轻度脂肪肝声像
4.43	5.98	2.94	2.60	1.91	未见异常	
4.44	5.65	0.90	2.94	1.73	异常	1、肝多发发性囊肿，
5.46	4.81	3.78	2.13	1.39	异常	轻度脂肪肝声像
4.64	5.45	0.70	2.45	2.16	未见异常	
4.67	7.93	3.00	4.57	1.75	异常	脂肪肝声像
5.73	5.35	3.02	2.58	1.49	未见异常	
6.74	6.68	3.55	3.82	1.30	异常	脂肪肝声像
4.26	8.14	0.92	3.95	2.15	异常	肝内囊性回声（考虑
4.85	7.18	1.41	4.19	1.79	异常	肝内囊性回声(考虑
4.08	6.90	1.23	3.99	1.47	异常	胆囊内强回声，考虑
7.74	6.30	2.75	3.42	1.38	异常	脂肪肝声像
5.36	5.90	1.48	3.21	1.48	异常	轻度脂肪肝声像、胆
5.55	4.60	1.72	2.35	1.29	异常	轻度脂肪肝声像
4.95	5.11	1.54	2.52	1.63	异常	轻度脂肪肝
7.21	3.99	0.74	1.86	1.38	异常	肝囊肿
4.52	4.69	1.34	2.20	1.44	异常	肝囊肿 胆囊结石
4.91	4.02	0.90	2.03	1.29	未见异常	
5.51	4.63	3.56	1.85	1.54	异常	腹部B超：1、脂肪肝
5.14	6.26	0.91	2.95	1.94	未见异常	
4.94	5.64	2.80	2.77	1.60	异常	轻度脂肪肝声像
4.71	7.27	1.38	3.55	2.02	未见异常	
4.63	5.91	1.92	2.50	2.36	未见异常	
5.82	6.15	3.73	2.97	1.49	异常	胆囊结石
5.00	7.45	3.38	3.93	1.38	异常	胆囊息肉样病变
4.65	6.55	3.41	3.19	1.72	异常	肝囊肿
7.46	6.00	4.97	2.57	1.29	异常	脂肪肝声像
4.98	4.15	1.85	2.02	1.32	未见异常	
4.87	3.28	1.90	1.35	1.15	异常	肝囊肿

5.47	6.09	0.84	3.15	1.82	未见异常	
5.09	5.88	3.15	2.73	1.47	异常	轻度脂肪肝、胆囊多
4.78	5.31	1.53	2.80	1.44	未见异常	
6.67	4.51	0.88	2.51	1.37		
6.99	6.41	1.71	3.79	1.80	未见异常	
7.58	3.84	2.05	1.62	1.57		
5.76	5.75	1.29	3.27	1.05	异常	轻度脂肪肝声像
5.67	5.13	3.57	2.07	1.56	异常	脂肪肝声像
4.93	6.14	2.35	3.41	1.37	未见异常	
5.46	6.41	0.81	3.21	1.83	未见异常	
4.33	4.58	1.34	2.50	1.34	异常	轻度脂肪肝声像
4.77	7.50	1.02	4.23	1.97	未见异常	
5.22	5.80	2.52	3.12	1.43	异常	轻度脂肪肝声像
4.98	6.01	1.28	3.59	1.65	异常	胆囊结石
10.19	7.22	1.74	4.10	1.57	未见异常	
4.99	4.07	0.64	1.60	1.81	未见异常	
5.16	4.89	0.78	1.52	2.28	未见异常	
4.99	6.83	3.20	3.48	4.53	未见异常	
5.87	7.73	1.49	4.59	1.76	异常	轻度脂肪肝声像
4.29	5.01	1.00	2.54	1.57	未见异常	
4.66	6.13	1.70	3.53	1.46	异常	轻度脂肪肝声像
4.32	4.68	1.95	1.98	1.91	未见异常	
6.69	3.71	1.59	1.27	1.65	异常	脂肪肝声像；肝囊肿
5.40	7.17	1.10	4.01	1.69	未见异常	
5.07	6.80	0.81	4.14	1.92	未见异常	
5.54	5.15	0.72	2.43	1.56	异常	肝实质回声稍增粗
5.40	6.43	1.82	2.37	2.74	异常	轻度脂肪肝声像
14.14	4.26	2.77	1.97	1.40	未见异常	
4.62	6.20	1.99	3.34	1.62	未见异常	
13.87	6.55	2.00	3.91	1.30	异常	1.脂肪肝声像 2.肝
5.53	7.06	1.82	3.60	1.60	未见异常	
4.98	7.68	0.98	4.55	1.49	未见异常	
5.67	6.41	0.84	3.55	1.85	未见异常	
6.49	6.83	3.27	3.63	1.46	异常	脂肪肝声像
9.93	5.54	6.07	1.72	1.23	异常	轻度脂肪肝声像，胆
9.03	6.44	2.96	3.38	1.45	异常	肝内稍强回声小结节
5.11	6.08	1.12	3.15	1.96	异常	胆囊腔内强回声团（
5.84	6.30	1.29	3.57	1.84	异常	轻度脂肪肝声像
6.41	6.42	2.69	1.41	3.50	异常	脂肪肝声像
4.51	6.30	1.37	3.66	1.67	未见异常	
5.48	7.75	2.25	4.53	2.00	异常	胆囊结石
5.37	5.94	2.62	3.15	1.70	异常	腹部B超：肝多发囊
5.83	6.26	1.63	3.42	1.51	未见异常	
6.97	9.04	2.83	4.91	1.78	未见异常	
4.91	8.09	1.15	4.44	2.06	未见异常	
4.98	4.30	0.68	1.61	1.71	未见异常	
9.33	5.51	1.93	3.03	1.63	异常	肝囊肿
5.91	5.35	2.10	2.70	1.72	异常	肝多发囊肿
4.44	4.88	1.15	2.64	1.56	异常	轻度脂肪肝声像
5.29	5.30	1.59	2.57	1.53	异常	胆囊内强回声（考虑
5.00	6.22	1.59	3.28	2.09	未见异常	

7.46	4.04	2.53	2.01	1.37	异常	脂肪肝声像
4.54	3.68	1.98	1.87	1.12	异常	脂肪肝声像
5.35	4.22	1.15	1.87	1.50	异常	脾大
5.12	5.82	1.37	2.89	1.68	未见异常	
5.11	5.52	0.99	2.72	1.95	未见异常	
5.23	5.44	1.00	3.10	1.62	未见异常	
5.29	4.90	1.81	2.64	1.28	异常	轻度脂肪肝
4.27	5.52	1.00	3.00	1.59	未见异常	
4.25	7.17	3.75	3.67	1.58	异常	脂肪肝声像
5.67	5.81	1.26	3.18	1.40	异常	轻度脂肪肝；肝囊肿
4.83	4.59	3.26	1.99	1.44	未见异常	
5.40	5.96	1.05	3.11	1.73	异常	肝内强回声，考虑钙
4.88	7.78	1.46	4.24	1.98	异常	脂肪肝声像，肝内囊
5.04	6.49	1.05	2.86	2.12	未见异常	
4.89	6.86	1.05	3.46	1.95	异常	轻度脂肪肝声像
5.90	5.10	1.67	2.01	1.89	异常	轻度脂肪肝声像
5.53	4.20	0.99	2.17	1.47	未见异常	
11.14	6.95	1.44	3.83	1.76	异常	1、轻度脂肪肝声像2
4.23	5.12	1.66	2.16	1.99	异常	轻度脂肪肝声像
7.13	4.84	1.60	2.61	1.37	未见异常	
4.77	4.52	1.63	2.35	1.28	异常	轻度脂肪肝，肝内强
4.44	6.42	1.12	2.84	2.23	异常	1.肝内强回声斑，考
6.24	4.61	2.24	2.22	1.39	未见异常	
9.23	6.07	4.92	2.66	1.62	异常	轻度脂肪肝声像
4.85	5.83	1.12	3.31	1.64	异常	轻度脂肪肝声像
5.28	6.52	2.11	3.62	1.94	未见异常	
6.68	7.54	2.96	3.85	1.68	未见异常	
4.22	5.36	1.50	2.77	1.53	异常	轻度脂肪肝
4.80	4.91	1.41	2.46	1.71	未见异常	
4.83	5.11	0.76	2.16	1.81	异常	1肝内强回声斑：考[illegible]
5.18	6.39	0.84	3.54	1.75	未见异常	
5.27	5.49	1.37	2.52	2.06	异常	1、肝多发囊肿；2、
5.61	5.85	3.11	3.16	1.52	异常	轻度脂肪肝声像　胆
5.09	6.09	1.75	3.33	1.69	未见异常	
4.70	6.46	1.06	3.80	1.65	异常	1、脂肪肝声像；　2、
5.28	5.22	1.28	3.21	1.23	异常	脂肪肝声像
5.87	4.99	0.67	2.82	1.71	未见异常	
4.88	6.41	0.94	3.56	1.70	异常	轻度脂肪肝声像
7.36	5.73	2.49	3.02	1.34	异常	脂肪肝声像
15.86	6.59	2.27	4.16	1.52	异常	脂肪肝声像
5.21	5.46	2.44	2.48	1.86	未见异常	
5.27	5.78	1.06	2.96	1.79	未见异常	
4.68	6.39	1.49	3.27	1.83	未见异常	
5.30	4.84	0.58	2.46	1.70	异常	肝内囊性回声
5.77	6.82	1.24	3.97	1.77	未见异常	
5.20	8.33	1.82	4.75	1.83	未见异常	
5.04	6.72	5.68	2.13	1.28	异常	肝多发囊肿
4.49	7.04	1.57	4.33	1.80	异常	脂肪肝声像
4.53	5.41	4.08	3.00	1.45	未见异常	
5.63	5.91	5.30	2.30	1.48	异常	脂肪肝声像
5.31	5.93	1.05	3.18	1.62	未见异常	

6.63	5.03	1.75	2.39	1.86	未见异常	
6.23	6.26	1.99	3.39	1.58	异常	肝小囊肿
5.28	5.44	0.74	2.80	1.93	未见异常	
4.50	6.81	1.27	3.47	1.93	未见异常	
5.53	7.56	3.48	3.63	1.84	异常	1、脂肪肝； 2、胆
5.40	6.30	4.05	3.01	1.57	异常	1.脂肪肝声像
4.56	5.25	1.31	2.80	1.60	异常	肝内强内声斑
5.66	9.24	1.59	5.94	2.10	异常	1、肝内强回声斑（
4.49	6.32	1.11	3.24	1.69	未见异常	
5.08	4.91	1.01	2.17	1.52	未见异常	
6.70	7.35	4.39	3.32	1.58	异常	脂肪肝声像
4.68	4.08	2.36	1.97	1.25	异常	脂肪肝声像
5.06	5.19	1.78	2.57	1.56	未见异常	
4.72	8.50	1.55	4.05	1.89	未见异常	
6.64	7.80	1.59	5.14	2.03		
11.77	5.66	1.94	3.09	1.65	异常	1.脂肪肝声像 2.胆
5.11	9.06	2.54	5.20	1.95	未见异常	
5.06	4.85	1.75	2.64	1.45	未见异常	
5.88	8.05	2.09	4.40	1.82	异常	脂肪肝声像
7.54	4.99	2.10	2.77	1.41	未见异常	
5.30	6.92	1.54	4.20	1.67	未见异常	
5.70	7.95	1.28	5.50	1.51	异常	肝囊肿
5.16	6.48	1.53	3.51	1.93	未见异常	
5.98	5.30	2.77	2.40	1.58	未见异常	
5.94	6.62	6.20	2.13	1.45		
5.07	7.09	2.70	3.17	2.21	异常	轻度脂肪肝声像。
5.71	5.15	1.42	3.03	1.38	异常	1、脂肪肝声像
7.48	6.99	1.65	3.73	1.68	异常	脂肪肝声像。
4.08	5.83	1.49	3.14	1.67	未见异常	
3.15	5.26	1.44	3.07	1.03	未见异常	
6.16	5.91	1.60	3.05	1.70	异常	肝囊息肉样病变
5.26	5.37	1.58	2.57	2.00	未见异常	
5.05	4.43	1.54	2.17	1.28	未见异常	
5.51	4.90	1.01	2.62	1.44	未见异常	
5.28	5.64	1.85	3.05	1.55	未见异常	
5.23	5.09	1.08	2.39	1.86	未见异常	
4.49	6.21	1.71	3.66	1.47	未见异常	
6.22	6.55	1.78	3.99	1.34	未见异常	
4.23	6.29	1.36	3.32	1.82	未见异常	
6.73	6.95	2.65	3.72	1.48	异常	肝囊肿
4.95	6.58	1.22	3.38	2.06	未见异常	
5.55	6.78	2.49	3.48	1.80	异常	轻度脂肪肝，胆囊结
4.58	5.93	1.01	3.06	1.69	异常	1.肝内强回声斑：考
5.47	4.52	1.03	2.17	1.70	未见异常	
4.66	5.57	1.67	2.66	1.95	未见异常	
5.87	7.02	2.04	3.98	1.68	未见异常	
7.68	6.18	5.16	2.50	1.30	未见异常	
5.01	4.76	1.01	2.42	1.52	未见异常	
11.11	5.17	2.16	2.69	1.46	异常	1、脂肪肝声像；2、
5.68	4.88	2.45	2.61	1.37	异常	1.脂肪肝声像 2.肝
7.53	4.26	1.35	2.13	1.53	未见异常	

4.92	5.88	1.81	3.11	1.80	未见异常	
4.72	4.80	1.57	2.33	1.54	未见异常	
4.47	4.90	1.32	2.12	1.73	异常	胆囊内强回声堆，考
5.59	5.41	1.56	3.10	1.56		
5.24	4.70	0.67	2.58	1.41	未见异常	
4.24	5.34	1.96	2.82	1.33	异常	轻度脂肪肝声像
10.36	5.19	3.04	2.45	1.57	异常	轻度脂肪肝声像
5.40	4.88	2.66	2.72	1.17	异常	脂肪肝声像
5.72	5.52	1.21	3.36	1.48	异常	胆囊点状稍高回声，
5.14	3.65	1.73	1.73	1.23	异常	脂肪肝声像
5.21	5.29	1.91	2.79	1.52	异常	轻度脂肪肝声像，胆
9.32	4.68	1.40	2.18	1.67	异常	胆囊结石
5.28	5.90	5.09	2.39	1.14	异常	胆囊结石
6.56	4.13	2.81	1.73	1.50	未见异常	
4.17	6.40	1.92	3.23	1.58	异常	轻度脂肪肝声像
5.46	4.00	1.68	1.81	1.60	未见异常	
5.66	6.66	1.35	3.83	1.54	异常	肝囊肿
6.51	5.52	5.12	2.05	1.39	未见异常	
4.59	6.47	3.30	3.74	1.37	异常	1.脂肪肝声像2.肝内
4.39	3.46	1.95	1.50	1.30	未见异常	
5.60	5.99	1.61	3.29	1.71	未见异常	
10.43	5.61	1.84	3.27	1.22	异常	1.轻度脂肪肝声像 2
4.69	8.76	2.00	5.34	1.37	未见异常	
5.80	6.52	1.43	3.87	1.41	异常	胆总管扩张、餐后胆
4.84	3.12	0.84	1.28	1.38	未见异常	
5.10	4.79	4.39	2.02	1.03	异常	轻度脂肪肝声像
5.56	4.81	2.10	2.44	1.33	异常	轻度脂肪肝声像、胆
7.84	5.93	1.17	3.52	1.50		
10.14	4.31	1.64	2.24	1.36	异常	1.脂肪肝声像2.胆囊
5.10	6.90	1.92	3.49	1.96	异常	腹部B超：肝囊肿
7.95	6.54	4.49	3.22	1.34	异常	脂肪肝声像
5.26	5.63	1.63	2.97	1.81	异常	脂肪肝声像
7.35	6.95	10.75	1.26	1.12	异常	轻度脂肪肝声像
5.42	7.03	3.94	3.61	1.33	异常	脂肪肝
5.26	6.99	1.09	3.42	2.01	异常	胆囊腔内强回声团块
6.82	5.50	7.61	1.26	1.44	异常	1、脂肪肝声像；2、
5.89	6.49	1.11	3.43	2.16	异常	胆囊结石？
7.41	7.97	2.79	4.68	1.27	异常	肝胆脾胰B超：轻度[illegible]
4.84	5.23	1.09	2.76	1.47	未见异常	
4.61	5.61	4.18	2.85	1.81	未见异常	
5.09	5.89	1.65	3.31	1.56	异常	脂肪肝声像
5.19	4.75	6.92	1.20	1.14	未见异常	
4.55	6.87	1.38	3.20	2.47	异常	肝多发囊性
22.14	5.83	1.31	3.09	1.61	异常	胆囊结石
4.86	5.85	1.68	3.00	1.58	异常	肝内强回声斑：考虑
4.58	4.52	1.76	2.31	1.40	异常	轻度脂肪肝声像
4.59	7.05	1.28	3.66	2.21	未见异常	
6.74	5.51	1.16	2.93	1.70	异常	脂肪肝声像
5.29	5.97	1.27	3.26	1.77	异常	轻度脂肪肝声像
5.85	4.54	2.49	2.10	1.40	未见异常	
5.89	6.03	7.50	1.88	1.09	异常	轻度脂肪肝声像

5.25	4.77	0.83	1.91	1.78	未见异常	
6.87	4.39	2.23	2.20	1.21	异常	脂肪肝声像
7.98	7.65	2.32	4.32	1.55	未见异常	
7.24	7.37	2.82	4.12	1.52	异常	轻度脂肪肝声像
5.42	5.95	1.71	2.95	1.66	未见异常	
4.67	5.13	1.70	3.00	1.24	未见异常	

WHtR	RFM	PI	CI	LAP	身体形态指数
0.62	43.61	17.78	1.34	71.06	0.0848
0.60	42.89	16.57	1.36	58.24	0.0870
0.66	45.83	17.35	1.46	52.77	0.0929
0.58	41.35	17.53	1.26	77.28	0.0800
0.61	43.25	18.14	1.32	39.60	0.0828
0.62	43.53	16.56	1.39	45.15	0.0885
0.62	43.59	19.98	1.27	36.25	0.0791
0.62	43.68	16.46	1.40	106.19	0.0884
0.60	42.88	18.62	1.28	77.70	0.0800
0.63	44.27	18.70	1.34	62.00	0.0831
0.59	42.02	17.87	1.28	66.50	0.0798
0.54	39.26	15.59	1.26	92.68	0.0808
0.53	38.41	14.01	1.30	68.50	0.0850
0.71	47.75	23.37	1.34	285.30	0.0813
0.54	38.93	16.52	1.22	41.28	0.0776
0.58	41.71	16.38	1.32	127.71	0.0840
0.68	46.69	19.13	1.43	59.77	0.0894
0.63	44.00	17.11	1.39	68.80	0.0886
0.65	45.39	22.22	1.27	53.20	0.0773
0.61	43.42	17.24	1.36	106.86	0.0852
0.68	46.77	20.79	1.38	117.76	0.0844
0.57	41.06	16.24	1.30	39.75	0.0839
0.59	41.85	16.40	1.33	38.64	0.0858
0.58	41.56	15.84	1.34	112.32	0.0856
0.59	42.21	18.57	1.26	85.84	0.0791
0.61	43.39	19.47	1.28	94.52	0.0792
0.61	43.37	18.66	1.30	117.29	0.0810
0.54	38.86	16.46	1.22	68.90	0.0773
0.61	43.45	17.31	1.35	641.88	0.0855
0.61	43.45	17.31	1.35	30.24	0.0855
0.64	44.75	20.68	1.29	52.06	0.0794
0.56	40.44	15.14	1.33	123.20	0.0850
0.60	42.81	16.57	1.36	81.72	0.0861
0.56	40.00	15.91	1.28	50.22	0.0818
0.62	43.77	18.52	1.32	71.64	0.0827
0.62	43.84	18.44	1.33	206.70	0.0827
0.47	33.43	12.70	1.21	26.04	0.0808
0.66	45.70	20.66	1.33	86.43	0.0817
0.62	43.53	16.99	1.37	92.05	0.0870
0.53	37.93	15.34	1.23	86.25	0.0789
0.72	48.04	22.36	1.39	93.00	0.0841
0.71	47.81	20.05	1.45	63.92	0.0901
0.47	33.08	14.15	1.14	65.80	0.0741
0.56	40.43	14.24	1.37	79.80	0.0888
0.60	42.74	17.24	1.33	286.38	0.0835
0.52	37.79	15.12	1.24	40.60	0.0801
0.61	43.37	16.73	1.37	68.08	0.0871
0.59	42.22	17.65	1.29	52.16	0.0814
0.59	42.14	18.44	1.26	41.40	0.0792
0.58	41.45	17.80	1.26	45.30	0.0792

0.58	41.78	17.39	1.29	57.28	0.0810
0.51	37.03	13.58	1.28	28.40	0.0841
0.63	44.30	19.35	1.32	51.12	0.0819
0.58	41.62	16.75	1.30	38.44	0.0828
0.55	39.81	14.81	1.32	81.90	0.0855
0.62	43.73	16.33	1.41	79.56	0.0894
0.57	40.83	16.75	1.27	54.52	0.0809
0.61	43.47	17.89	1.33	62.70	0.0842
0.53	38.17	15.25	1.24	22.75	0.0797
0.57	41.00	16.43	1.29	57.60	0.0823
0.62	43.74	17.19	1.37	100.10	0.0870
0.60	42.71	18.20	1.29	42.93	0.0819
0.61	43.37	16.27	1.39	76.22	0.0887
0.57	40.65	15.66	1.31	205.52	0.0843
0.63	44.13	18.04	1.36	89.43	0.0858
0.64	44.57	18.89	1.34	130.80	0.0835
0.56	40.60	15.61	1.31	34.22	0.0842
0.59	42.36	17.49	1.30	39.30	0.0827
0.57	40.72	13.44	1.42	34.41	0.0930
0.57	40.76	19.19	1.19	50.64	0.0745
0.61	43.28	15.40	1.43	145.52	0.0922
0.48	34.44	12.94	1.23	34.96	0.0807
0.58	41.62	15.36	1.36	146.63	0.0877
0.59	41.93	17.99	1.27	50.49	0.0795
0.54	38.73	16.55	1.21	78.00	0.0761
0.62	43.87	16.85	1.39	45.00	0.0884
0.57	40.68	15.52	1.32	123.12	0.0836
0.54	39.25	15.82	1.26	44.88	0.0810
0.64	44.72	21.41	1.27	95.40	0.0778
0.63	44.26	19.92	1.30	102.68	0.0805
0.64	44.91	18.81	1.36	62.56	0.0857
0.64	44.62	18.78	1.35	64.44	0.0846
0.55	39.37	15.58	1.27	38.75	0.0816
0.59	41.93	15.09	1.39	66.00	0.0894
0.59	42.07	17.11	1.31	169.88	0.0829
0.61	43.25	16.93	1.36	25.74	0.0867
0.74	48.98	22.45	1.43	241.92	0.0865
0.56	40.37	14.50	1.35	54.23	0.0877
0.74	49.08	22.96	1.42	49.22	0.0870
0.57	40.67	17.66	1.24	46.40	0.0773
0.59	42.11	15.58	1.37	67.34	0.0874
0.63	44.40	20.03	1.30	54.36	0.0803
0.68	46.77	20.50	1.39	69.00	0.0852
0.54	39.08	14.40	1.31	47.20	0.0861
0.60	42.63	19.30	1.25	106.08	0.0776
0.58	41.56	16.92	1.30	82.24	0.0819
0.50	36.13	13.60	1.25	57.19	0.0820
0.59	42.00	17.31	1.30	164.80	0.0819
0.55	39.75	14.69	1.32	30.00	0.0851
0.54	38.65	13.70	1.33	73.00	0.0870
0.60	42.74	14.88	1.43	33.29	0.0923

0.58	41.40	16.08	1.32	135.72	0.0848
0.56	40.29	15.56	1.30	99.32	0.0840
0.60	42.52	15.61	1.39	85.68	0.0890
0.62	43.96	17.08	1.39	47.25	0.0881
0.63	44.33	18.79	1.34	104.50	0.0833
0.52	37.46	15.72	1.20	47.04	0.0766
0.59	42.26	18.66	1.26	63.69	0.0784
0.54	39.04	15.62	1.26	35.07	0.0813
0.57	41.12	15.75	1.33	93.80	0.0853
0.53	38.59	14.43	1.29	110.43	0.0835
0.61	43.10	17.87	1.32	89.60	0.0828
0.60	42.44	16.85	1.33	100.80	0.0847
0.79	50.70	26.84	1.40	115.64	0.0826
0.57	40.69	18.69	1.20	121.21	0.0758
0.69	47.11	19.76	1.43	73.00	0.0880
0.53	38.22	16.75	1.19	62.33	0.0753
0.59	41.91	16.00	1.35	79.20	0.0864
0.61	43.42	15.97	1.41	354.90	0.0897
0.52	37.54	14.52	1.25	39.20	0.0817
0.59	41.98	16.66	1.32	106.72	0.0844
0.61	43.45	17.05	1.37	122.32	0.0852
0.53	38.44	13.42	1.33	42.00	0.0878
0.52	37.28	15.10	1.22	75.80	0.0789
0.55	39.81	16.80	1.24	48.62	0.0786
0.68	46.39	20.62	1.36	69.52	0.0839
0.60	42.74	15.73	1.39	157.79	0.0897
0.59	42.36	16.66	1.34	177.90	0.0854
0.58	41.52	14.52	1.40	62.06	0.0911
0.67	46.28	19.40	1.40	91.14	0.0863
0.68	46.40	21.59	1.33	106.26	0.0816
0.50	36.26	14.64	1.21	26.80	0.0782
0.61	43.02	17.45	1.33	44.64	0.0838
0.59	41.88	17.38	1.29	19.98	0.0821
0.55	39.86	14.68	1.33	68.40	0.0855
0.61	43.25	19.35	1.27	324.39	0.0793
0.52	37.50	16.43	1.18	47.08	0.0748
0.56	40.24	16.23	1.27	43.47	0.0814
0.52	37.73	15.31	1.23	61.64	0.0788
0.71	47.84	19.68	1.47	39.60	0.0916
0.65	45.29	21.84	1.28	139.40	0.0777
0.65	45.25	20.86	1.31	41.30	0.0809
0.55	39.53	15.04	1.30	95.04	0.0837
0.54	38.86	14.22	1.31	41.34	0.0852
0.59	42.05	17.35	1.30	68.32	0.0825
0.66	45.69	19.83	1.36	306.15	0.0845
0.66	45.56	23.33	1.25	60.80	0.0763
0.63	44.00	17.43	1.37	68.08	0.0867
0.61	43.47	17.89	1.33	123.42	0.0842
0.59	41.96	20.26	1.20	46.08	0.0731
0.65	45.15	18.04	1.40	71.28	0.0886
0.55	39.72	17.12	1.22	72.80	0.0771

0.57	40.94	14.75	1.36	55.80	0.0881
0.74	48.86	18.65	1.57	59.40	0.0977
0.60	42.60	17.83	1.30	62.64	0.0814
0.65	45.33	18.83	1.38	30.69	0.0874
0.56	40.24	14.81	1.33	161.73	0.0865
0.49	35.21	14.50	1.18	37.80	0.0766
0.59	41.96	14.40	1.42	80.64	0.0918
0.58	41.62	15.08	1.37	62.00	0.0888
0.47	33.81	14.24	1.15	19.05	0.0751
0.66	45.48	16.69	1.47	59.67	0.0940
0.52	37.48	15.01	1.23	41.17	0.0792
0.62	43.71	15.17	1.46	67.64	0.0939
0.70	47.24	21.20	1.39	97.76	0.0847
0.68	46.40	17.89	1.47	132.30	0.0925
0.56	40.09	14.96	1.32	63.90	0.0850
0.59	42.35	17.78	1.29	65.61	0.0822
0.58	41.49	16.28	1.32	69.63	0.0837
0.67	46.10	20.67	1.35	78.75	0.0826
0.55	39.64	14.89	1.31	36.60	0.0840
0.55	39.81	15.09	1.31	33.80	0.0844
0.58	41.65	13.82	1.44	51.84	0.0949
0.59	42.22	16.52	1.34	54.08	0.0852
0.65	45.07	17.19	1.43	52.65	0.0908
0.55	39.41	15.26	1.28	41.04	0.0830
0.64	44.57	17.80	1.38	38.40	0.0869
0.69	47.15	21.04	1.39	36.80	0.0850
0.64	44.82	19.40	1.34	154.44	0.0822
0.66	45.78	22.82	1.27	92.48	0.0782
0.55	39.76	16.98	1.23	74.25	0.0778
0.57	40.77	16.38	1.29	59.40	0.0818
0.59	42.02	16.61	1.33	92.40	0.0838
0.58	41.27	15.97	1.32	124.08	0.0841
0.55	39.33	14.51	1.31	33.02	0.0853
0.57	40.89	15.72	1.32	70.08	0.0841
0.57	40.76	15.42	1.33	24.44	0.0858
0.56	40.42	18.57	1.20	63.00	0.0747
0.57	40.94	16.78	1.28	93.42	0.0814
0.65	45.25	20.14	1.33	95.90	0.0828
0.63	44.30	20.25	1.29	55.08	0.0794
0.51	36.50	13.99	1.24	58.74	0.0808
0.58	41.36	15.93	1.33	36.00	0.0850
0.58	41.71	16.07	1.34	101.97	0.0851
0.54	39.29	16.86	1.22	56.16	0.0770
0.54	38.75	16.63	1.21	46.42	0.0771
0.60	42.67	17.48	1.32	61.76	0.0833
0.59	41.91	17.30	1.29	222.90	0.0820
0.52	37.27	13.12	1.31	242.55	0.0865
0.59	41.83	14.74	1.40	46.74	0.0897
0.68	46.40	20.05	1.38	65.52	0.0858
0.55	39.57	15.08	1.30	53.04	0.0838
0.62	43.84	18.44	1.33	56.55	0.0827

0.63	44.00	20.79	1.26	78.44	0.0771
0.61	43.19	19.28	1.27	48.98	0.0796
0.60	42.82	21.94	1.18	71.01	0.0726
0.61	43.23	19.17	1.28	60.12	0.0793
0.68	46.39	22.92	1.29	93.72	0.0782
0.59	42.05	16.71	1.32	197.68	0.0846
0.59	42.36	16.04	1.36	85.80	0.0876
0.58	41.78	17.66	1.28	36.16	0.0802
0.55	39.49	14.99	1.30	53.48	0.0836
0.69	47.03	21.21	1.38	67.62	0.0837
0.58	41.56	17.11	1.29	45.12	0.0813
0.57	40.83	16.19	1.30	23.49	0.0828
0.55	39.76	18.62	1.17	117.45	0.0731
0.62	43.66	17.37	1.36	140.04	0.0860
0.65	45.40	18.15	1.41	186.90	0.0882
0.55	39.56	14.28	1.33	206.40	0.0859
0.61	43.45	18.15	1.32	97.20	0.0829
0.67	46.20	18.70	1.42	85.20	0.0895
0.63	44.26	17.99	1.36	47.26	0.0862
0.66	45.60	18.22	1.41	86.10	0.0886
0.74	48.99	21.30	1.47	202.37	0.0893
0.52	37.58	13.50	1.30	75.42	0.0862
0.61	43.44	18.22	1.32	38.72	0.0832
0.72	48.14	22.65	1.38	88.56	0.0833
0.59	41.91	16.98	1.31	51.00	0.0830
0.57	40.94	17.95	1.24	50.22	0.0773
0.53	38.05	14.19	1.28	21.40	0.0842
0.57	40.61	14.33	1.37	57.66	0.0888
0.65	45.22	17.83	1.41	88.00	0.0883
0.54	39.00	14.50	1.30	143.22	0.0852
0.68	46.40	20.67	1.36	101.64	0.0840
0.60	42.70	15.05	1.42	95.40	0.0914
0.63	44.17	19.13	1.32	47.60	0.0823
0.52	37.30	16.48	1.17	29.07	0.0747
0.60	42.67	16.00	1.38	41.92	0.0883
0.56	40.15	17.00	1.24	59.52	0.0791
0.51	36.75	14.47	1.23	64.46	0.0796
0.67	46.10	19.12	1.40	95.88	0.0868
0.59	42.35	17.85	1.29	45.36	0.0820
0.58	41.62	18.15	1.25	79.67	0.0785
0.58	41.45	15.38	1.35	54.30	0.0873
0.58	41.68	14.78	1.39	52.54	0.0892
0.55	39.82	15.10	1.31	40.30	0.0836
0.62	43.66	18.51	1.32	117.00	0.0824
0.50	36.00	13.69	1.24	37.43	0.0813
0.62	43.55	14.93	1.46	92.00	0.0941
0.53	38.08	16.39	1.20	34.20	0.0767
0.59	42.02	15.72	1.36	81.20	0.0869
0.57	41.00	15.61	1.33	76.80	0.0852
0.50	36.26	13.68	1.25	61.56	0.0820
0.58	41.33	15.28	1.35	35.20	0.0870

0.62	43.84	18.18	1.34	243.75	0.0835
0.67	46.31	20.09	1.38	35.49	0.0858
0.63	44.04	19.68	1.29	135.32	0.0805
0.65	45.03	21.43	1.28	59.85	0.0788
0.61	43.45	17.59	1.34	84.60	0.0846
0.62	43.63	18.35	1.32	76.44	0.0824
0.66	45.48	18.51	1.40	79.95	0.0877
0.60	42.90	17.13	1.34	310.96	0.0861
0.61	43.03	16.30	1.38	60.39	0.0882
0.56	40.39	15.58	1.31	117.84	0.0845
0.64	44.52	21.34	1.26	109.20	0.0782
0.57	41.06	17.09	1.27	33.35	0.0805
0.58	41.65	16.10	1.33	57.80	0.0846
0.63	44.04	16.65	1.41	99.45	0.0892
0.62	43.92	17.41	1.37	48.16	0.0857
0.65	45.01	17.92	1.40	52.46	0.0875
0.57	40.88	16.08	1.30	73.44	0.0841
0.61	43.02	17.72	1.32	75.24	0.0829
0.56	40.04	15.87	1.28	105.40	0.0814
0.59	42.22	15.38	1.39	58.88	0.0893
0.57	41.17	18.46	1.23	53.07	0.0767
0.57	40.71	17.29	1.25	23.49	0.0789
0.58	41.71	15.30	1.37	40.26	0.0879
0.58	41.56	16.65	1.31	31.36	0.0828
0.64	44.63	18.14	1.37	88.43	0.0864
0.58	41.34	17.31	1.27	29.10	0.0804
0.76	49.81	25.29	1.39	95.70	0.0830
0.47	33.06	17.67	1.02	22.10	0.0645
0.59	41.84	18.22	1.26	52.08	0.0789
0.61	43.33	18.26	1.31	54.08	0.0828
0.64	44.91	16.81	1.44	81.47	0.0918
0.62	43.53	17.51	1.35	48.36	0.0847
0.63	44.39	18.57	1.35	118.30	0.0846
0.47	33.53	13.43	1.18	18.30	0.0775
0.72	48.36	21.64	1.43	333.84	0.0869
0.61	43.03	15.56	1.41	150.48	0.0910
0.66	45.47	18.58	1.39	148.37	0.0878
0.59	41.91	17.19	1.30	59.10	0.0823
0.52	37.71	16.02	1.20	36.48	0.0762
0.63	44.13	16.89	1.40	64.02	0.0896
0.60	42.44	16.87	1.33	25.23	0.0851
0.69	46.96	20.91	1.38	881.82	0.0847
0.55	39.41	14.22	1.33	102.24	0.0871
0.51	37.00	13.70	1.27	36.96	0.0832
0.56	40.32	15.25	1.32	56.70	0.0846
0.53	38.00	14.52	1.27	149.60	0.0825
0.61	43.32	19.12	1.28	31.20	0.0815
0.52	37.69	14.18	1.27	83.25	0.0825
0.58	41.68	16.70	1.31	55.80	0.0833
0.63	44.12	17.25	1.39	61.49	0.0868
0.50	35.74	13.96	1.22	58.50	0.0798

0.60	42.74	17.39	1.32	103.70	0.0834
0.67	46.00	19.54	1.38	99.88	0.0856
0.73	48.73	20.74	1.48	60.84	0.0908
0.72	48.36	19.65	1.50	41.08	0.0927
0.69	46.82	18.81	1.45	58.80	0.0913
0.67	46.10	21.49	1.32	82.56	0.0808
0.63	44.30	20.86	1.27	39.60	0.0779
0.58	41.78	16.57	1.32	51.84	0.0837
0.58	41.54	16.32	1.32	62.56	0.0835
0.60	42.52	18.14	1.29	44.64	0.0810
0.62	43.69	18.57	1.32	79.86	0.0828
0.64	44.98	17.65	1.41	90.40	0.0887
0.61	43.19	19.92	1.25	93.62	0.0779
0.65	45.40	21.22	1.30	165.90	0.0794
0.60	42.74	18.01	1.30	121.38	0.0815
0.61	43.23	18.62	1.30	47.52	0.0809
0.62	43.77	18.41	1.33	74.88	0.0831
0.55	39.86	15.70	1.28	73.75	0.0825
0.73	48.45	22.81	1.39	57.60	0.0847
0.58	41.65	19.28	1.22	59.40	0.0760
0.55	39.46	16.81	1.22	103.96	0.0781
0.69	47.03	19.87	1.42	132.30	0.0875
0.73	48.52	23.61	1.37	176.40	0.0829
0.70	47.31	21.48	1.38	51.25	0.0851
0.64	44.70	23.44	1.21	68.00	0.0734
0.69	46.82	18.43	1.46	94.00	0.0925
0.68	46.78	21.46	1.36	290.84	0.0829
0.57	40.71	17.19	1.25	45.36	0.0795
0.72	48.08	22.47	1.39	195.22	0.0849
0.60	42.74	18.35	1.29	55.49	0.0810
0.67	46.00	19.76	1.38	83.26	0.0847
0.64	44.51	18.82	1.34	158.40	0.0841
0.70	47.49	22.77	1.35	33.11	0.0822
0.67	46.10	20.67	1.35	144.29	0.0824
0.58	41.78	17.80	1.27	46.72	0.0798
0.58	41.54	18.81	1.23	18.25	0.0773
0.55	39.56	15.87	1.26	64.00	0.0800
0.58	41.41	18.57	1.23	45.63	0.0773
0.64	44.79	22.35	1.24	43.23	0.0762
0.57	40.83	16.75	1.27	27.26	0.0809
0.60	42.52	16.98	1.33	116.28	0.0842
0.72	48.19	25.55	1.31	71.44	0.0778
0.56	40.32	16.02	1.28	85.80	0.0818
0.51	36.99	13.18	1.30	196.88	0.0851
0.62	43.67	20.07	1.27	27.34	0.0778
0.60	42.45	19.23	1.25	68.95	0.0771
0.58	41.61	18.19	1.25	36.48	0.0794
0.65	45.22	20.16	1.33	62.48	0.0814
0.63	44.26	21.53	1.25	65.62	0.0765
0.77	49.91	25.19	1.40	116.28	0.0834
0.59	42.21	19.20	1.24	26.68	0.0774

0.71	48.00	23.27	1.36	149.24	0.0815
0.54	38.76	14.94	1.27	99.18	0.0817
0.63	44.30	22.07	1.23	119.52	0.0750
0.52	37.75	15.91	1.20	38.50	0.0770
0.59	41.91	17.19	1.30	71.10	0.0823
0.64	44.78	18.43	1.37	106.00	0.0855
0.61	42.96	17.94	1.31	48.96	0.0824
0.67	46.00	22.89	1.28	80.96	0.0770
0.62	43.72	18.78	1.31	86.70	0.0821
0.59	42.22	15.09	1.40	94.40	0.0904
0.57	40.94	17.23	1.26	17.82	0.0800
0.57	40.94	15.01	1.35	99.82	0.0871
0.61	43.44	18.22	1.32	116.76	0.0839
0.61	43.47	17.89	1.33	92.73	0.0842
0.67	46.19	18.51	1.43	40.80	0.0889
0.53	38.03	14.96	1.25	21.63	0.0811
0.64	44.67	16.87	1.43	49.92	0.0916
0.60	42.88	16.17	1.38	57.00	0.0874
0.61	43.25	20.56	1.24	177.87	0.0762
0.54	38.86	17.04	1.20	179.14	0.0755
0.64	44.98	16.23	1.47	95.60	0.0938
0.50	35.75	15.18	1.17	28.35	0.0750
0.50	36.00	15.88	1.15	41.99	0.0736
0.66	45.49	20.19	1.34	51.25	0.0826
0.57	41.18	14.50	1.38	153.09	0.0905
0.61	43.35	19.04	1.29	34.40	0.0794
0.56	40.44	17.75	1.22	79.35	0.0778
0.53	38.39	14.05	1.30	45.60	0.0839
0.67	46.21	21.20	1.34	74.86	0.0826
0.55	39.33	14.24	1.33	112.06	0.0864
0.64	44.66	18.57	1.36	84.24	0.0849
0.55	39.33	16.25	1.24	45.44	0.0782
0.58	41.65	18.25	1.25	48.28	0.0778
0.70	47.27	20.28	1.42	83.72	0.0867
0.57	41.12	15.41	1.34	37.24	0.0865
0.55	39.37	17.09	1.21	25.25	0.0768
0.53	38.59	16.68	1.20	74.06	0.0764
0.59	42.04	16.33	1.34	688.94	0.0844
0.63	44.09	19.41	1.31	150.84	0.0811
0.55	39.53	15.98	1.26	193.59	0.0803
0.54	38.75	14.81	1.28	34.54	0.0833
0.59	42.34	13.83	1.47	44.45	0.0957
0.61	43.37	16.92	1.37	204.98	0.0865
0.61	43.47	16.19	1.40	375.21	0.0900
0.53	38.59	14.60	1.28	27.54	0.0828
0.57	40.77	15.31	1.33	60.00	0.0856
0.64	44.78	19.27	1.34	104.80	0.0830
0.66	45.71	21.83	1.30	61.16	0.0786
0.68	46.41	19.35	1.41	76.00	0.0881
0.56	40.37	19.87	1.16	51.04	0.0711
0.63	44.42	19.56	1.31	133.94	0.0816

0.66	45.80	21.71	1.30	80.41	0.0793
0.61	43.03	17.48	1.33	61.38	0.0842
0.62	43.87	20.83	1.25	60.12	0.0768
0.70	47.45	20.67	1.41	125.32	0.0863
0.59	42.30	16.86	1.33	37.06	0.0839
0.67	46.31	19.42	1.40	95.94	0.0877
0.59	41.89	17.14	1.30	125.12	0.0821
0.55	39.72	15.15	1.30	59.64	0.0836
0.55	39.47	17.33	1.21	65.96	0.0776
0.60	42.63	17.23	1.32	146.20	0.0837
0.62	43.58	21.22	1.23	105.08	0.0749
0.58	41.49	15.50	1.35	69.96	0.0865
0.50	36.39	15.56	1.17	43.51	0.0755
0.59	41.91	17.36	1.29	40.50	0.0818
0.59	42.22	15.95	1.36	90.88	0.0872
0.55	39.33	14.79	1.30	260.00	0.0843
0.69	47.05	20.22	1.41	115.62	0.0868
0.63	44.27	18.25	1.35	263.58	0.0836
0.58	41.78	15.88	1.35	66.24	0.0861
0.57	41.18	20.98	1.15	61.56	0.0707
0.61	43.25	19.05	1.28	46.20	0.0801
0.51	36.54	15.10	1.20	9.92	0.0779
0.63	44.00	19.53	1.30	42.42	0.0797
0.65	45.15	19.68	1.34	155.16	0.0836
0.61	43.03	17.48	1.33	293.70	0.0842
0.68	46.38	21.71	1.33	77.28	0.0805
0.51	36.53	15.73	1.17	86.87	0.0756
0.62	43.87	17.17	1.38	118.44	0.0873
0.63	44.25	16.57	1.42	195.39	0.0902
0.60	42.67	17.78	1.31	98.88	0.0823
0.67	46.10	18.30	1.43	279.93	0.0899
0.62	43.50	19.49	1.28	133.00	0.0789
0.62	43.53	23.70	1.16	98.89	0.0702
0.58	41.29	16.44	1.30	57.71	0.0832
0.56	40.32	15.50	1.31	38.10	0.0836
0.64	44.51	21.59	1.25	33.84	0.0767
0.62	43.66	17.80	1.34	97.20	0.0846
0.69	47.00	21.32	1.37	227.64	0.0843
0.55	39.75	15.74	1.28	99.66	0.0826
0.56	40.42	15.64	1.30	123.48	0.0837
0.60	42.52	17.99	1.29	90.83	0.0814
0.66	45.80	18.34	1.42	220.88	0.0886
0.62	43.96	19.90	1.28	35.60	0.0789
0.63	44.37	17.09	1.40	234.64	0.0905
0.57	41.05	16.42	1.30	120.78	0.0820
0.60	42.67	17.63	1.31	45.12	0.0828
0.58	41.35	17.84	1.25	53.20	0.0791
0.72	48.16	24.45	1.33	117.04	0.0804
0.46	32.23	12.78	1.17	27.72	0.0780
0.80	50.91	25.00	1.46	75.04	0.0878
0.59	41.88	18.53	1.25	34.02	0.0787

0.60	42.51	18.75	1.27	58.24	0.0796
0.61	42.97	17.31	1.33	68.20	0.0848
0.51	37.01	13.42	1.28	71.19	0.0845
0.53	38.56	14.86	1.27	49.28	0.0816
0.55	39.76	17.25	1.22	75.60	0.0769
0.69	47.00	20.44	1.40	145.74	0.0867
0.56	40.42	15.36	1.32	29.40	0.0847
0.56	40.44	15.38	1.32	50.24	0.0841
0.65	45.20	18.34	1.39	70.56	0.0869
0.65	45.29	21.07	1.30	107.83	0.0796
0.48	34.65	14.10	1.18	36.64	0.0772
0.64	44.84	19.74	1.33	76.96	0.0823
0.54	38.93	16.52	1.22	47.04	0.0776
0.68	46.69	19.13	1.43	105.78	0.0894
0.67	46.10	20.99	1.34	74.88	0.0826
0.60	42.67	16.89	1.34	102.40	0.0852
0.56	40.37	16.92	1.25	397.88	0.0792
0.65	45.39	18.52	1.39	130.80	0.0872
0.55	39.76	16.98	1.23	40.50	0.0778
0.53	38.08	16.07	1.21	57.95	0.0778
0.58	41.68	15.97	1.34	39.60	0.0858
0.53	38.00	15.72	1.22	146.08	0.0782
0.56	40.00	15.50	1.29	96.12	0.0833
0.63	44.16	18.44	1.34	94.80	0.0836
0.59	41.87	16.80	1.31	111.52	0.0829
0.65	45.40	17.87	1.42	158.34	0.0891
0.51	37.07	16.39	1.16	36.72	0.0747
0.48	34.13	13.18	1.21	55.42	0.0794
0.63	44.45	17.59	1.39	58.89	0.0873
0.71	47.67	23.53	1.33	40.92	0.0809
0.57	40.75	12.99	1.44	109.56	0.0970
0.60	42.67	16.78	1.34	80.15	0.0851
0.59	42.15	19.30	1.23	110.22	0.0764
0.60	42.88	16.76	1.35	115.50	0.0858
0.67	46.30	18.89	1.42	43.87	0.0890
0.55	39.88	17.50	1.21	48.33	0.0765
0.69	47.18	20.46	1.41	84.04	0.0870
0.70	47.25	18.32	1.49	109.48	0.0936
0.69	46.83	18.59	1.46	90.44	0.0924
0.65	45.28	19.56	1.35	33.93	0.0839
0.51	37.03	17.65	1.12	45.80	0.0706
0.64	44.78	21.02	1.28	69.20	0.0783
0.67	46.10	19.52	1.39	84.24	0.0867
0.59	42.25	17.48	1.30	28.82	0.0837
0.55	39.41	18.36	1.17	60.75	0.0730
0.66	45.50	19.82	1.35	92.02	0.0833
0.53	38.53	15.73	1.23	79.59	0.0796
0.60	42.52	18.44	1.28	265.98	0.0801
0.65	45.15	21.32	1.29	63.00	0.0792
0.57	41.18	16.90	1.28	25.38	0.0817
0.61	43.45	15.91	1.41	108.00	0.0905

0.54	39.06	17.31	1.19	60.21	0.0750
0.62	43.95	21.05	1.25	83.70	0.0773
0.61	43.05	19.02	1.28	55.50	0.0800
0.59	41.91	17.19	1.30	67.50	0.0823
0.51	37.00	13.70	1.27	32.12	0.0832
0.46	32.25	13.05	1.16	16.66	0.0764
0.58	41.65	18.00	1.26	69.36	0.0786
0.56	40.56	16.98	1.26	128.96	0.0790
0.55	39.49	18.18	1.18	41.50	0.0739
0.78	50.43	22.67	1.51	167.58	0.0916
0.66	45.70	18.67	1.40	151.70	0.0877
0.56	40.09	15.72	1.29	123.30	0.0822
0.55	39.57	15.08	1.30	43.68	0.0838
0.61	43.32	17.46	1.34	33.60	0.0866
0.72	48.30	21.47	1.43	124.85	0.0867
0.54	38.74	14.35	1.30	24.70	0.0843
0.61	43.02	19.33	1.27	44.64	0.0783
0.58	41.62	16.53	1.31	36.89	0.0835
0.58	41.23	16.19	1.31	64.20	0.0837
0.64	44.89	16.76	1.44	48.64	0.0928
0.63	44.21	19.17	1.32	92.50	0.0820
0.59	41.98	16.01	1.35	69.31	0.0867
0.59	42.24	17.57	1.30	96.25	0.0813
0.62	43.88	18.66	1.32	51.66	0.0819
0.59	41.88	16.96	1.31	92.61	0.0835
0.54	39.26	15.97	1.25	35.00	0.0795
0.64	44.94	20.24	1.31	75.60	0.0814
0.49	34.93	15.61	1.13	21.42	0.0726
0.54	39.10	17.45	1.19	46.80	0.0749
0.45	31.71	14.23	1.10	20.64	0.0715
0.70	47.59	23.63	1.33	44.10	0.0797
0.71	47.94	22.31	1.38	101.20	0.0853
0.60	42.48	17.76	1.30	71.61	0.0817
0.58	41.58	16.66	1.31	42.56	0.0835
0.46	32.29	15.36	1.07	12.00	0.0690
0.55	39.61	18.30	1.18	145.75	0.0739
0.61	43.45	18.26	1.32	93.96	0.0825
0.53	38.00	14.84	1.25	30.78	0.0805
0.58	41.27	16.35	1.31	163.35	0.0828
0.56	40.42	16.47	1.27	93.80	0.0809
0.62	43.98	20.53	1.26	93.60	0.0778
0.61	43.35	16.53	1.38	118.40	0.0873
0.57	41.12	14.96	1.36	69.72	0.0883
0.66	45.58	19.92	1.35	75.62	0.0840
0.57	41.21	15.10	1.36	87.12	0.0867
0.59	42.04	18.57	1.25	121.77	0.0781
0.56	40.40	17.06	1.25	92.56	0.0793
0.65	45.05	19.52	1.34	65.12	0.0836
0.72	48.04	23.24	1.36	96.50	0.0820
0.72	48.04	24.25	1.33	79.00	0.0797
0.60	42.74	18.82	1.27	101.68	0.0796

0.63	44.00	17.33	1.38	89.88	0.0863
0.63	44.00	16.08	1.43	64.64	0.0923
0.65	45.29	17.51	1.43	78.31	0.0901
0.52	37.65	14.09	1.27	45.15	0.0834
0.64	44.66	16.23	1.45	103.35	0.0928
0.57	41.00	17.52	1.25	66.90	0.0788
0.50	36.13	13.69	1.24	24.89	0.0816
0.60	42.67	18.95	1.26	69.68	0.0798
0.56	40.19	16.84	1.25	51.52	0.0791
0.63	44.11	20.42	1.27	88.80	0.0783
0.54	39.10	13.43	1.36	62.40	0.0892
0.70	47.32	17.94	1.51	99.84	0.0949
0.53	38.13	14.67	1.26	38.72	0.0822
0.66	45.70	18.07	1.42	130.38	0.0896
0.75	49.39	22.96	1.44	103.53	0.0875
0.70	47.31	21.30	1.39	116.85	0.0856
0.56	40.19	15.61	1.30	212.24	0.0832
0.61	43.10	18.01	1.31	137.20	0.0824
0.58	41.68	17.14	1.29	42.00	0.0818
0.65	45.07	20.74	1.30	103.35	0.0801
0.58	41.78	17.11	1.30	84.48	0.0819
0.63	44.04	18.93	1.32	177.76	0.0812
0.59	41.98	15.65	1.36	97.24	0.0872
0.66	45.90	19.65	1.38	293.15	0.0854
0.60	42.89	18.71	1.28	57.28	0.0802
0.62	43.60	19.79	1.27	28.50	0.0784
0.54	39.25	16.06	1.25	238.48	0.0802
0.58	41.58	16.97	1.29	37.80	0.0824
0.56	40.34	15.30	1.32	30.50	0.0853
0.52	37.54	15.85	1.20	39.60	0.0770
0.60	42.59	17.00	1.33	37.50	0.0849
0.60	42.67	16.89	1.34	33.60	0.0852
0.58	41.22	15.38	1.35	70.04	0.0860
0.65	45.39	17.19	1.45	125.60	0.0917
0.55	39.95	15.84	1.28	91.00	0.0818
0.62	43.61	18.14	1.33	134.64	0.0837
0.64	44.89	19.44	1.34	53.71	0.0827
0.59	42.36	14.81	1.42	58.50	0.0924
0.56	40.37	17.45	1.23	132.24	0.0775
0.60	42.52	16.63	1.34	44.95	0.0858
0.57	40.95	15.01	1.35	35.70	0.0864
0.65	45.38	20.02	1.34	87.40	0.0831
0.53	38.07	14.05	1.29	73.95	0.0833
0.59	42.37	19.82	1.23	56.76	0.0756
0.59	42.24	17.31	1.31	59.15	0.0821
0.63	44.04	16.79	1.40	49.72	0.0880
0.58	41.58	16.66	1.31	41.44	0.0835
0.54	38.65	14.77	1.28	66.25	0.0827
0.63	44.26	19.92	1.30	90.44	0.0805
0.59	42.15	16.02	1.35	35.31	0.0865
0.68	46.38	19.41	1.41	115.15	0.0868

0.66	45.79	22.31	1.29	192.28	0.0785
0.67	46.31	20.57	1.36	70.80	0.0843
0.68	46.42	18.75	1.43	81.70	0.0903
0.56	40.24	14.24	1.36	24.84	0.0888
0.60	42.88	18.34	1.29	34.65	0.0808
0.57	40.66	15.69	1.31	43.20	0.0839
0.67	46.21	20.52	1.36	62.32	0.0844
0.69	46.98	20.98	1.38	73.48	0.0849
0.49	35.11	14.72	1.17	61.53	0.0752
0.64	44.82	18.66	1.36	50.16	0.0844
0.55	39.75	16.56	1.24	67.76	0.0798
0.54	38.80	15.45	1.25	229.25	0.0791
0.62	43.71	19.15	1.30	52.44	0.0804
0.55	39.81	14.52	1.33	38.22	0.0866
0.61	43.05	19.98	1.25	116.70	0.0775
0.72	48.11	20.33	1.46	134.13	0.0898
0.69	46.96	22.65	1.33	127.88	0.0803
0.56	40.18	15.70	1.29	125.40	0.0821
0.69	47.20	24.28	1.29	42.84	0.0779
0.55	39.95	18.26	1.19	52.92	0.0744
0.59	42.17	17.91	1.28	42.84	0.0799
0.63	44.04	20.46	1.27	55.08	0.0785
0.48	34.73	13.39	1.22	103.53	0.0792
0.67	46.10	18.09	1.44	143.35	0.0900
0.60	42.67	13.63	1.49	82.56	0.0983
0.55	39.33	15.33	1.28	149.24	0.0822
0.56	40.47	17.28	1.24	75.06	0.0786
0.59	42.00	17.87	1.28	49.28	0.0802
0.57	40.82	16.55	1.28	37.50	0.0822
0.56	40.14	15.80	1.29	53.36	0.0822
0.56	40.37	16.81	1.26	82.07	0.0795
0.50	35.76	15.36	1.16	40.80	0.0740
0.66	45.48	18.82	1.39	40.17	0.0868
0.65	45.06	19.12	1.36	55.10	0.0847
0.62	43.77	19.84	1.28	68.76	0.0790
0.53	38.28	14.81	1.26	104.79	0.0823
0.64	44.94	17.99	1.39	48.96	0.0880
0.60	42.44	18.30	1.28	35.52	0.0801
0.57	40.65	16.34	1.28	44.24	0.0819
0.53	38.56	16.87	1.19	51.12	0.0756
0.71	47.68	19.83	1.45	127.28	0.0908
0.58	41.56	16.89	1.30	43.84	0.0820
0.48	34.24	14.10	1.17	23.68	0.0763
0.64	44.57	20.18	1.30	41.91	0.0809
0.62	43.69	18.89	1.31	42.57	0.0818
0.69	47.00	20.01	1.41	73.50	0.0880
0.51	37.15	14.67	1.23	35.60	0.0802
0.58	41.44	17.63	1.26	38.72	0.0794
0.64	44.87	19.75	1.33	75.66	0.0821
0.73	48.50	23.84	1.37	119.60	0.0827
0.47	33.81	15.74	1.10	12.60	0.0702

0.54	39.29	15.54	1.27	65.88	0.0813
0.56	40.39	16.39	1.27	57.36	0.0817
0.53	38.03	15.70	1.22	56.91	0.0785
0.59	41.87	14.73	1.40	140.08	0.0905
0.69	47.17	20.43	1.41	189.74	0.0858
0.57	40.71	16.00	1.30	50.22	0.0834
0.61	43.19	18.32	1.31	71.30	0.0824
0.63	44.33	19.08	1.33	45.98	0.0825
0.65	45.22	18.35	1.39	156.64	0.0866
0.54	38.79	14.70	1.29	58.24	0.0828
0.61	43.23	16.98	1.36	32.76	0.0860
0.53	38.25	14.52	1.28	81.18	0.0831
0.58	41.29	16.38	1.31	36.54	0.0834
0.52	37.67	15.04	1.23	23.14	0.0791
0.67	46.20	19.20	1.41	44.94	0.0876
0.57	40.65	14.95	1.34	74.48	0.0870
0.59	42.30	18.53	1.27	55.08	0.0788
0.68	46.40	19.99	1.39	103.74	0.0859
0.64	44.63	19.65	1.32	166.13	0.0819
0.61	43.42	17.49	1.35	125.97	0.0844
0.62	43.58	17.76	1.34	64.35	0.0849
0.60	42.59	19.83	1.23	161.70	0.0766
0.61	43.37	17.19	1.36	58.09	0.0855
0.76	49.53	26.27	1.35	157.18	0.0796
0.57	41.12	18.37	1.23	47.88	0.0770
0.58	41.68	16.35	1.32	51.60	0.0845
0.68	46.41	21.26	1.34	52.80	0.0828
0.57	40.89	16.23	1.30	74.56	0.0823
0.59	42.08	17.21	1.30	147.42	0.0814
0.61	43.01	17.82	1.32	44.07	0.0822
0.66	45.89	20.86	1.33	31.82	0.0826
0.58	41.29	16.56	1.30	47.85	0.0828
0.63	44.00	20.51	1.27	38.64	0.0771
0.52	37.33	15.09	1.22	47.94	0.0796
0.52	37.46	13.94	1.28	411.36	0.0830
0.62	43.96	18.74	1.32	51.80	0.0828
0.64	44.82	22.17	1.25	114.45	0.0764
0.56	40.37	17.32	1.24	51.91	0.0779
0.65	45.29	17.37	1.43	33.62	0.0906
0.61	43.44	20.19	1.25	168.00	0.0783
0.59	41.88	19.35	1.22	49.95	0.0764
0.60	42.44	15.97	1.37	99.84	0.0877
0.62	43.84	19.23	1.30	88.53	0.0804
0.57	41.06	15.66	1.33	66.12	0.0853
0.57	41.12	14.47	1.38	22.62	0.0906
0.55	39.82	14.71	1.32	41.23	0.0850
0.61	43.31	17.09	1.36	102.90	0.0860
0.58	41.71	15.18	1.37	59.60	0.0872
0.56	40.00	16.75	1.25	24.57	0.0791
0.63	44.48	17.91	1.38	136.53	0.0861
0.62	43.91	19.60	1.29	42.57	0.0805

0.65	45.29	19.65	1.35	216.48	0.0834
0.62	43.92	18.62	1.33	148.96	0.0826
0.56	40.52	17.53	1.24	34.06	0.0782
0.60	42.82	18.32	1.29	76.20	0.0814
0.55	39.47	15.56	1.27	22.61	0.0834
0.67	46.10	18.24	1.44	164.97	0.0908
0.62	43.61	18.74	1.31	81.60	0.0819
0.59	42.09	18.18	1.27	673.88	0.0792
0.56	40.02	15.65	1.29	47.56	0.0824
0.52	37.84	17.38	1.15	21.96	0.0734
0.62	43.53	19.75	1.27	87.50	0.0787
0.63	44.42	19.35	1.32	64.38	0.0821
0.63	44.13	19.15	1.32	68.02	0.0817
0.45	31.67	13.44	1.13	10.26	0.0747
0.54	38.93	14.24	1.31	36.00	0.0856
0.66	45.58	18.73	1.39	163.40	0.0875
0.60	42.44	18.30	1.28	34.24	0.0801
0.60	42.67	15.98	1.38	66.15	0.0879
0.49	34.92	12.98	1.24	16.96	0.0822
0.58	41.45	19.08	1.22	41.70	0.0756
0.56	40.14	16.07	1.28	57.13	0.0813
0.55	39.53	15.90	1.26	19.17	0.0806
0.55	39.53	16.11	1.25	38.88	0.0799
0.56	40.37	14.50	1.35	55.39	0.0877
0.57	40.88	17.14	1.26	62.72	0.0800
0.61	43.45	17.87	1.33	117.00	0.0837
0.63	44.26	18.64	1.34	72.76	0.0842
0.52	37.69	15.18	1.23	24.25	0.0788
0.61	43.14	16.36	1.38	31.72	0.0895
0.60	42.67	18.07	1.29	64.00	0.0814
0.49	35.38	13.52	1.23	41.58	0.0800
0.52	37.25	16.11	1.18	38.72	0.0752
0.51	36.96	13.64	1.27	33.50	0.0828
0.60	42.59	18.22	1.29	33.00	0.0806
0.63	44.00	20.09	1.28	87.36	0.0796
0.60	42.52	18.34	1.28	41.14	0.0799
0.57	41.12	16.89	1.28	82.88	0.0814
0.67	46.10	18.72	1.42	110.29	0.0889
0.60	42.74	14.84	1.43	151.59	0.0933
0.55	39.72	16.59	1.24	69.44	0.0787
0.61	43.19	17.51	1.34	31.62	0.0849
0.63	44.16	17.38	1.38	117.20	0.0869
0.68	46.41	21.32	1.34	63.20	0.0826
0.56	40.00	16.75	1.25	99.90	0.0791
0.57	41.18	16.04	1.32	33.75	0.0846
0.54	38.93	15.38	1.26	35.28	0.0814
0.61	43.42	20.83	1.23	70.06	0.0762
0.56	40.20	16.76	1.25	96.90	0.0791
0.57	40.89	15.72	1.32	53.12	0.0841
0.64	44.66	18.22	1.37	211.77	0.0859
0.58	41.67	16.49	1.32	137.60	0.0836

0.49	35.47	13.14	1.25	35.82	0.0825
0.64	44.91	17.83	1.40	136.74	0.0874
0.59	41.96	15.87	1.35	45.36	0.0860
0.57	40.78	16.94	1.27	78.54	0.0795
0.52	37.81	14.92	1.24	62.75	0.0800
0.65	45.05	19.58	1.34	88.36	0.0820
0.59	42.36	16.87	1.33	114.60	0.0847
0.61	43.33	17.31	1.35	153.92	0.0858
0.63	44.09	17.96	1.36	87.84	0.0854
0.52	37.86	14.96	1.24	40.32	0.0795
0.57	41.12	14.52	1.38	86.52	0.0900
0.62	43.50	20.01	1.26	51.00	0.0787
0.50	36.00	13.66	1.24	26.86	0.0818
0.61	43.17	19.02	1.28	71.06	0.0798
0.57	40.69	19.83	1.17	38.64	0.0728
0.58	41.43	19.65	1.20	25.50	0.0735
0.60	42.81	16.07	1.38	51.84	0.0879
0.69	46.99	20.04	1.41	95.03	0.0877
0.62	43.53	17.43	1.35	67.20	0.0856
0.59	41.84	16.49	1.32	43.40	0.0843
0.67	46.00	18.70	1.41	38.18	0.0879
0.57	40.95	17.13	1.26	67.98	0.0794
0.63	44.22	18.81	1.33	63.04	0.0838
0.59	42.05	20.08	1.21	53.48	0.0749
0.58	41.58	16.66	1.31	55.44	0.0835
0.65	45.28	18.89	1.37	51.48	0.0859
0.57	41.11	15.25	1.35	59.52	0.0865
0.55	39.57	14.94	1.30	36.66	0.0843
0.61	43.16	16.33	1.38	98.42	0.0878
0.63	44.44	19.56	1.31	104.12	0.0815
0.55	39.72	17.12	1.22	126.00	0.0771
0.66	45.50	19.63	1.36	73.53	0.0839
0.53	37.98	15.61	1.22	31.05	0.0784
0.57	41.12	16.30	1.30	61.04	0.0834
0.56	40.19	14.79	1.33	35.84	0.0863
0.58	41.45	17.09	1.28	23.40	0.0814
0.62	43.58	20.26	1.26	69.93	0.0772
0.65	45.31	17.99	1.41	250.26	0.0882
0.68	46.78	19.65	1.42	122.76	0.0880
0.59	41.84	16.74	1.31	88.04	0.0834
0.58	41.35	17.53	1.26	154.28	0.0800
0.64	44.85	18.70	1.36	93.38	0.0841
0.57	41.11	17.05	1.27	91.20	0.0802
0.53	38.44	14.24	1.29	50.16	0.0843
0.59	41.84	15.38	1.37	54.87	0.0883
0.59	42.07	16.99	1.31	73.16	0.0833
0.62	43.55	16.67	1.39	179.20	0.0874
0.61	43.03	17.93	1.31	71.61	0.0828
0.66	45.70	20.44	1.34	85.69	0.0825
0.73	48.45	25.06	1.33	120.96	0.0796
0.63	44.28	19.90	1.30	91.84	0.0797

0.59	42.15	16.70	1.33	82.50	0.0842
0.59	42.07	16.27	1.34	72.85	0.0857
0.59	42.22	17.94	1.28	217.60	0.0806
0.55	39.81	13.10	1.40	53.30	0.0927
0.61	43.05	19.02	1.28	108.90	0.0800
0.57	40.63	15.40	1.32	72.52	0.0838
0.49	34.96	14.07	1.19	33.63	0.0775
0.63	44.08	20.23	1.28	117.67	0.0782
0.59	42.29	14.81	1.41	91.45	0.0919
0.61	43.39	16.30	1.39	161.16	0.0892
0.62	43.68	16.41	1.40	65.60	0.0886
0.64	44.75	20.62	1.29	58.90	0.0795
0.51	36.77	15.80	1.18	13.60	0.0754
0.59	42.37	18.43	1.27	56.76	0.0794
0.51	36.76	11.82	1.36	66.57	0.0913
0.61	43.17	19.23	1.27	31.28	0.0793
0.61	43.23	17.19	1.35	42.12	0.0853
0.64	44.95	17.32	1.42	89.30	0.0887
0.62	43.87	19.83	1.28	60.45	0.0800
0.58	41.71	17.12	1.29	35.31	0.0815
0.58	41.49	16.02	1.33	47.85	0.0846
0.63	44.27	21.41	1.25	162.00	0.0759
0.59	42.32	16.89	1.33	64.38	0.0847
0.59	42.11	16.49	1.33	87.04	0.0849
0.58	41.68	13.94	1.43	153.90	0.0939
0.55	39.37	17.37	1.20	130.25	0.0759
0.62	43.78	17.71	1.35	74.56	0.0859
0.68	46.49	22.50	1.31	40.95	0.0793
0.52	37.46	14.96	1.23	65.04	0.0792
0.60	42.48	20.38	1.21	260.10	0.0749
0.59	42.38	18.76	1.26	34.20	0.0781
0.63	44.30	17.53	1.38	127.08	0.0875
0.50	35.73	14.52	1.20	31.28	0.0779
0.62	43.93	16.61	1.40	182.24	0.0898
0.54	39.10	14.96	1.29	43.94	0.0830
0.53	38.08	17.35	1.16	28.50	0.0739
0.62	43.78	21.65	1.22	87.36	0.0751
0.63	44.44	19.91	1.30	63.36	0.0814
0.68	46.40	19.21	1.41	96.35	0.0884
0.61	43.11	16.29	1.38	48.32	0.0886
0.69	47.05	19.08	1.45	90.24	0.0902
0.64	44.89	18.43	1.37	59.45	0.0857
0.61	43.39	18.37	1.31	113.56	0.0823
0.63	44.28	15.87	1.45	83.64	0.0926
0.53	38.11	15.57	1.23	16.20	0.0797
0.58	41.29	19.46	1.20	61.48	0.0744
0.59	42.37	19.27	1.24	71.28	0.0771
0.57	40.78	16.55	1.28	61.12	0.0810
0.54	38.82	13.69	1.33	83.97	0.0871
0.59	41.81	18.57	1.25	53.76	0.0782
0.58	41.24	14.46	1.39	37.70	0.0910

0.55	39.66	16.32	1.25	49.92	0.0800
0.63	44.37	15.84	1.46	131.60	0.0932
0.54	39.08	18.75	1.15	31.60	0.0722
0.60	42.59	18.26	1.29	77.10	0.0810
0.56	40.18	15.01	1.32	63.03	0.0846
0.76	49.75	23.45	1.44	153.36	0.0872
0.55	39.95	14.10	1.36	63.84	0.0884
0.60	42.89	19.05	1.27	37.76	0.0792
0.65	45.06	19.85	1.33	216.60	0.0826
0.67	46.21	21.87	1.32	85.32	0.0812
0.65	45.05	19.68	1.34	80.29	0.0832
0.59	42.09	17.91	1.28	83.30	0.0800
0.56	40.58	16.06	1.29	83.25	0.0832
0.70	47.34	21.60	1.38	91.26	0.0852
0.59	41.98	17.89	1.27	240.12	0.0805
0.58	41.39	15.74	1.34	132.99	0.0856
0.68	46.43	18.62	1.44	27.36	0.0911
0.59	42.22	17.37	1.30	44.16	0.0823
0.67	46.29	17.12	1.49	88.36	0.0941
0.61	43.17	18.88	1.29	47.60	0.0802
0.59	42.37	18.01	1.29	97.02	0.0807
0.71	47.76	20.43	1.44	73.92	0.0892
0.57	40.65	16.23	1.29	78.68	0.0823
0.70	47.48	24.64	1.30	52.00	0.0771
0.52	37.45	16.60	1.17	55.75	0.0737
0.63	44.43	17.13	1.40	87.11	0.0901
0.59	42.30	19.60	1.23	49.98	0.0759
0.58	41.56	18.26	1.25	96.64	0.0778
0.52	37.43	13.64	1.29	51.74	0.0838
0.58	41.75	17.84	1.27	46.98	0.0800
0.62	43.78	18.37	1.33	58.88	0.0838
0.57	41.11	13.44	1.43	66.96	0.0933
0.51	36.73	12.94	1.30	88.08	0.0854
0.56	40.37	15.58	1.30	53.65	0.0837
0.56	40.14	16.73	1.25	36.54	0.0792
0.63	44.00	18.51	1.33	35.89	0.0833
0.56	40.14	18.44	1.19	35.67	0.0742
0.51	36.64	15.76	1.17	60.20	0.0753
0.56	40.18	19.40	1.16	47.52	0.0713
0.56	40.42	17.59	1.23	80.64	0.0774
0.63	44.48	19.26	1.33	94.71	0.0820
0.61	43.10	17.23	1.34	256.55	0.0849
0.66	45.59	19.65	1.36	69.20	0.0845
0.58	41.52	17.19	1.28	42.63	0.0814
0.59	42.32	16.97	1.32	95.83	0.0831
0.60	42.74	18.20	1.29	144.15	0.0814
0.64	44.57	20.18	1.30	109.23	0.0809
0.58	41.37	18.51	1.23	33.84	0.0778
0.55	39.95	18.64	1.18	22.08	0.0741
0.63	44.35	15.41	1.48	40.92	0.0961
0.60	42.82	18.32	1.29	25.80	0.0814

0.65	45.31	19.83	1.34	123.20	0.0826
0.59	42.22	19.08	1.24	43.84	0.0773
0.64	44.54	21.20	1.27	34.96	0.0775
0.60	42.46	17.01	1.33	116.28	0.0833
0.52	37.79	14.54	1.26	60.00	0.0822
0.55	39.68	15.21	1.30	39.73	0.0831
0.58	41.29	15.39	1.35	29.87	0.0869
0.56	40.48	16.27	1.28	90.19	0.0816
0.63	44.42	18.07	1.37	83.99	0.0860
0.49	35.47	14.52	1.19	48.45	0.0773
0.69	47.03	20.41	1.40	113.68	0.0859
0.66	45.60	17.78	1.43	188.19	0.0902
0.55	39.57	14.24	1.33	45.50	0.0870
0.55	39.66	17.84	1.20	39.36	0.0754
0.65	45.31	20.14	1.33	28.38	0.0818
0.57	41.17	16.52	1.30	66.96	0.0823
0.59	41.91	16.44	1.33	61.80	0.0848
0.61	43.33	18.89	1.29	54.08	0.0809
0.60	42.67	17.82	1.30	95.76	0.0813
0.62	43.63	16.56	1.39	85.02	0.0882
0.57	40.72	16.28	1.29	113.77	0.0819
0.63	44.28	18.07	1.36	116.55	0.0858
0.67	46.22	18.28	1.44	73.44	0.0918
0.63	44.39	20.78	1.27	58.80	0.0785
0.60	42.59	17.94	1.30	54.78	0.0815
0.64	44.94	20.24	1.31	71.64	0.0814
0.61	43.39	15.70	1.42	48.28	0.0914
0.68	46.49	19.36	1.41	162.45	0.0876
0.62	43.50	18.47	1.31	27.60	0.0830
0.46	32.90	16.19	1.06	27.56	0.0675
0.59	42.22	18.17	1.27	43.52	0.0799
0.52	37.75	14.91	1.24	66.66	0.0804
0.56	40.14	16.07	1.28	36.54	0.0813
0.49	35.17	16.37	1.11	29.82	0.0713
0.61	43.17	18.88	1.29	106.76	0.0802
0.58	41.56	13.70	1.44	70.40	0.0943
0.60	42.59	18.79	1.27	39.27	0.0790
0.54	39.08	18.75	1.15	20.60	0.0722
0.60	42.44	16.71	1.34	64.38	0.0856
0.61	43.45	16.47	1.39	34.56	0.0884
0.63	44.42	14.52	1.52	86.21	0.0995
0.51	36.50	13.18	1.28	31.68	0.0841
0.64	44.70	20.76	1.29	52.36	0.0796
0.57	41.00	15.39	1.34	70.80	0.0859
0.55	39.60	15.17	1.29	68.82	0.0827
0.66	45.50	21.15	1.31	65.10	0.0799
0.61	43.05	19.02	1.28	135.30	0.0800
0.62	43.78	19.68	1.28	105.28	0.0800
0.71	47.74	19.17	1.48	62.73	0.0920
0.56	40.09	14.70	1.33	39.60	0.0860
0.55	39.52	15.09	1.29	38.10	0.0830

0.53	38.41	16.59	1.20	49.00	0.0759
0.49	35.47	13.69	1.22	36.54	0.0803
0.55	39.70	16.75	1.24	87.38	0.0773
0.61	42.96	17.65	1.32	121.38	0.0832
0.56	40.00	16.94	1.24	167.68	0.0777
0.56	40.60	17.52	1.24	101.50	0.0779
0.58	41.29	15.74	1.33	72.36	0.0860
0.62	43.50	17.38	1.35	110.58	0.0851
0.59	42.30	16.11	1.36	103.02	0.0865
0.51	36.79	16.93	1.14	18.90	0.0724
0.57	40.89	15.67	1.32	122.24	0.0843
0.58	41.62	16.75	1.30	53.63	0.0828
0.63	44.00	19.36	1.30	86.21	0.0808
0.53	37.95	17.12	1.17	74.16	0.0735
0.68	46.75	21.48	1.35	48.96	0.0823
0.72	48.26	21.56	1.42	180.00	0.0873
0.58	41.62	17.31	1.28	49.60	0.0810
0.62	43.50	16.33	1.40	143.64	0.0888
0.58	41.41	16.37	1.31	81.00	0.0841
0.61	42.95	18.09	1.31	131.35	0.0815
0.67	46.30	19.82	1.39	115.50	0.0861
0.60	42.59	16.52	1.35	48.84	0.0861
0.59	42.30	17.70	1.29	58.48	0.0812
0.59	41.81	16.37	1.33	43.12	0.0851
0.58	41.23	15.91	1.32	106.80	0.0847
0.64	44.75	19.85	1.32	106.40	0.0816
0.63	44.43	17.01	1.41	218.68	0.0885
0.59	42.37	17.03	1.32	103.95	0.0837
0.48	34.08	13.40	1.20	19.35	0.0788
0.66	45.81	18.40	1.42	164.22	0.0882
0.60	42.67	18.37	1.28	42.88	0.0805
0.60	42.52	17.25	1.32	85.68	0.0833
0.62	43.74	17.78	1.35	52.15	0.0851
0.55	39.41	16.00	1.25	40.80	0.0805
0.56	40.60	16.98	1.26	46.11	0.0796
0.56	40.42	16.19	1.28	50.96	0.0818
0.60	42.94	16.46	1.37	80.00	0.0863
0.59	42.05	17.67	1.29	91.56	0.0815
0.62	43.78	17.06	1.38	194.24	0.0881
0.57	40.89	17.49	1.25	38.70	0.0786
0.46	32.44	13.93	1.13	40.35	0.0734
0.61	43.17	15.77	1.41	39.10	0.0905
0.53	37.93	15.97	1.21	63.25	0.0767
0.57	41.16	15.05	1.36	69.30	0.0869
0.62	43.66	19.08	1.30	65.16	0.0808
0.64	44.63	20.02	1.31	165.00	0.0800
0.61	43.11	16.76	1.36	49.14	0.0859
0.55	39.53	15.12	1.29	39.42	0.0834
0.58	41.62	16.75	1.30	32.55	0.0828
0.58	41.39	17.80	1.26	51.77	0.0789
0.55	39.68	15.47	1.28	41.76	0.0822

0.67	46.00	20.55	1.35	56.44	0.0842
0.63	44.30	17.84	1.37	58.68	0.0865
0.63	44.45	14.52	1.53	191.88	0.0992
0.57	40.94	16.23	1.30	28.00	0.0836
0.66	45.89	17.78	1.45	55.50	0.0919
0.56	40.02	14.21	1.35	90.19	0.0880
0.56	40.49	16.61	1.27	32.86	0.0802
0.61	43.02	18.82	1.28	33.28	0.0812
0.55	39.76	15.69	1.28	25.38	0.0820
0.56	40.14	15.80	1.29	72.21	0.0822
0.61	43.33	17.31	1.35	72.64	0.0858
0.57	40.84	14.53	1.37	51.81	0.0883
0.56	40.56	15.53	1.31	169.60	0.0839
0.64	44.91	20.01	1.32	118.32	0.0822
0.69	47.11	22.57	1.34	166.05	0.0817
0.64	44.67	19.80	1.32	140.48	0.0824
0.60	42.44	17.28	1.32	75.52	0.0832
0.67	46.10	20.67	1.35	91.18	0.0824
0.68	46.40	19.37	1.41	87.78	0.0878
0.60	42.59	17.31	1.32	81.00	0.0839
0.60	42.59	17.29	1.32	38.34	0.0844
0.65	45.14	20.03	1.33	57.75	0.0827
0.59	41.91	17.48	1.29	74.70	0.0814
0.77	50.02	26.48	1.37	127.44	0.0808
0.61	43.01	16.36	1.38	95.55	0.0870
0.56	40.19	15.31	1.31	54.88	0.0843
0.60	42.67	15.85	1.38	97.28	0.0889
0.61	43.23	17.25	1.35	95.76	0.0851
0.60	42.90	19.42	1.26	50.17	0.0787
0.62	43.56	17.03	1.37	61.12	0.0874
0.62	43.69	17.88	1.34	63.36	0.0849
0.65	45.42	18.22	1.41	148.50	0.0876
0.50	35.71	14.09	1.21	22.08	0.0804
0.60	42.81	15.54	1.40	158.04	0.0899
0.66	45.79	20.26	1.35	75.60	0.0840
0.55	39.93	14.95	1.32	50.84	0.0845
0.54	39.29	14.09	1.33	121.23	0.0867
0.60	42.81	17.52	1.32	58.68	0.0829
0.51	36.96	15.90	1.18	161.25	0.0764
0.58	41.53	17.65	1.27	106.38	0.0803
0.60	42.67	18.52	1.28	243.52	0.0801
0.58	41.78	20.26	1.19	89.28	0.0732
0.56	40.57	15.24	1.33	56.10	0.0846
0.51	36.53	16.00	1.16	31.68	0.0746
0.58	41.56	14.37	1.41	37.12	0.0913
0.65	45.40	18.43	1.40	53.34	0.0873
0.56	40.05	14.81	1.33	195.00	0.0861
0.65	45.28	21.31	1.29	86.97	0.0792
0.62	43.56	19.28	1.29	90.24	0.0805
0.55	39.53	15.04	1.30	40.50	0.0837
0.52	37.60	17.08	1.16	13.77	0.0739

0.52	37.67	14.86	1.24	51.48	0.0797
0.51	36.53	16.44	1.15	14.58	0.0732
0.67	46.29	17.91	1.46	93.53	0.0913
0.56	40.42	15.36	1.32	31.64	0.0847
0.57	40.65	15.09	1.34	50.96	0.0864
0.49	35.21	11.55	1.32	28.26	0.0892
0.61	43.11	17.27	1.34	68.80	0.0852
0.58	41.65	17.67	1.27	56.70	0.0806
0.47	33.22	12.87	1.20	34.44	0.0792
0.51	36.82	12.99	1.30	17.85	0.0870
0.52	37.35	14.64	1.24	11.04	0.0815
0.60	42.80	14.87	1.43	36.12	0.0916
0.61	43.33	19.68	1.27	38.72	0.0788
0.41	26.79	10.74	1.14	10.05	0.0776
0.53	38.00	13.44	1.32	28.82	0.0868
0.52	37.46	13.18	1.31	35.76	0.0862
0.56	40.19	14.79	1.33	18.48	0.0863
0.51	36.77	15.36	1.19	26.40	0.0769
0.59	41.91	15.41	1.37	35.10	0.0886
0.46	32.94	13.16	1.17	9.10	0.0775
0.53	38.50	15.11	1.26	28.16	0.0815
0.57	40.71	16.30	1.29	44.01	0.0824
0.48	34.33	15.11	1.13	12.46	0.0734
0.69	47.13	20.04	1.42	108.03	0.0888
0.59	42.09	16.77	1.32	75.98	0.0844
0.60	42.74	17.84	1.31	41.93	0.0823
0.52	37.48	14.88	1.23	43.24	0.0797
0.55	39.46	14.81	1.30	30.82	0.0850
0.61	43.45	17.73	1.34	35.28	0.0842
0.58	41.48	16.73	1.30	36.40	0.0832
0.64	44.94	21.21	1.28	56.16	0.0789
0.53	38.13	16.07	1.21	19.21	0.0783
0.51	37.05	14.50	1.24	30.42	0.0809
0.56	40.05	17.86	1.21	18.98	0.0760
0.62	43.83	20.36	1.26	67.32	0.0781
0.50	36.00	12.82	1.28	26.22	0.0842
0.49	35.46	13.33	1.24	21.12	0.0820
0.53	37.98	14.54	1.27	55.66	0.0822
0.55	39.72	15.80	1.27	47.60	0.0813
0.60	42.81	15.68	1.40	41.25	0.0898
0.60	42.67	16.92	1.34	78.75	0.0846
0.59	42.29	14.52	1.43	78.74	0.0932
0.54	38.78	15.74	1.24	71.40	0.0802
0.66	45.60	19.08	1.38	91.14	0.0859
0.56	40.30	16.48	1.27	26.25	0.0817
0.62	43.73	15.72	1.43	55.80	0.0932
0.55	39.86	16.65	1.24	55.00	0.0793
0.46	32.52	13.33	1.16	10.67	0.0765
0.57	40.66	14.20	1.38	54.00	0.0897
0.56	40.53	13.51	1.41	51.52	0.0927
0.63	44.42	15.50	1.48	58.46	0.0952

0.52	37.39	14.87	1.23	27.09	0.0798
0.51	36.54	13.17	1.28	32.80	0.0853
0.48	34.73	13.16	1.23	26.25	0.0802
0.60	42.74	16.04	1.38	38.75	0.0886
0.61	42.97	17.31	1.33	76.57	0.0848
0.54	39.29	16.59	1.23	39.15	0.0778
0.47	33.14	12.44	1.21	18.24	0.0812
0.45	31.15	12.03	1.18	4.48	0.0796
0.66	45.89	19.79	1.37	64.40	0.0859
0.59	41.87	15.56	1.36	50.66	0.0872
0.57	40.88	14.03	1.40	26.32	0.0914
0.50	35.75	14.38	1.20	55.22	0.0776
0.55	39.59	15.37	1.29	17.60	0.0838
0.53	38.08	15.74	1.22	27.93	0.0788
0.63	44.35	19.52	1.31	96.36	0.0820
0.57	40.64	13.96	1.39	19.76	0.0914
0.57	41.00	15.94	1.31	45.36	0.0850
0.67	46.10	20.05	1.37	104.55	0.0849
0.58	41.56	18.07	1.25	46.08	0.0784
0.39	25.33	11.96	1.05	2.62	0.0704
0.58	41.65	15.72	1.35	49.30	0.0860
0.59	42.37	17.59	1.30	49.50	0.0819
0.43	29.23	11.67	1.15	6.09	0.0775
0.58	41.75	15.27	1.37	60.90	0.0888
0.57	40.76	16.19	1.29	52.78	0.0831
0.58	41.39	15.88	1.33	70.06	0.0851
0.55	39.75	14.10	1.35	22.00	0.0888
0.60	42.52	17.78	1.30	115.94	0.0820
0.54	39.17	15.39	1.27	50.64	0.0819
0.54	38.96	13.93	1.33	22.77	0.0872
0.55	39.93	14.38	1.34	27.56	0.0875
0.61	42.98	16.76	1.36	55.72	0.0872
0.63	44.18	18.95	1.32	120.00	0.0836
0.59	41.90	16.85	1.31	28.50	0.0842
0.52	37.54	15.11	1.23	20.40	0.0795
0.56	40.32	14.47	1.35	25.80	0.0876
0.42	28.12	11.54	1.13	8.96	0.0758
0.53	37.98	14.54	1.27	46.46	0.0822
0.57	41.18	16.21	1.31	36.75	0.0844
0.59	42.17	16.53	1.33	72.45	0.0865
0.55	39.79	15.81	1.27	73.37	0.0813
0.63	44.02	16.92	1.39	122.74	0.0884
0.59	41.84	18.14	1.26	43.09	0.0791
0.52	37.50	14.02	1.27	20.46	0.0831
0.54	39.25	16.83	1.22	36.30	0.0777
0.62	43.79	16.19	1.42	98.42	0.0904
0.53	37.98	14.35	1.27	16.79	0.0829
0.61	43.03	17.78	1.32	37.95	0.0832
0.54	38.65	14.50	1.29	164.75	0.0837
0.55	39.81	15.95	1.27	47.58	0.0813
0.59	42.05	17.67	1.29	51.24	0.0815

0.51	37.05	14.04	1.26	43.02	0.0827
0.51	36.67	13.71	1.26	10.37	0.0832
0.53	38.31	17.94	1.15	78.00	0.0726
0.53	38.53	12.96	1.36	67.20	0.0907
0.45	31.15	11.72	1.19	12.00	0.0809
0.53	38.34	14.43	1.28	33.63	0.0842
0.54	39.21	16.63	1.22	12.65	0.0781
0.58	41.52	16.30	1.32	30.16	0.0843
0.56	40.30	17.84	1.22	79.80	0.0775
0.62	43.50	17.38	1.35	65.74	0.0851
0.60	42.74	16.41	1.36	38.92	0.0877
0.54	39.26	14.20	1.33	57.40	0.0860
0.58	41.62	17.03	1.29	45.26	0.0819
0.54	38.75	14.63	1.29	50.38	0.0840
0.63	44.00	17.94	1.35	39.96	0.0851
0.51	37.03	14.52	1.24	29.60	0.0804
0.59	42.28	16.40	1.34	305.20	0.0864
0.57	41.19	16.41	1.30	37.95	0.0840
0.52	37.30	13.91	1.27	15.58	0.0836
0.58	41.45	16.66	1.30	55.50	0.0828
0.49	34.93	12.59	1.26	22.78	0.0837
0.48	33.89	11.79	1.27	17.64	0.0848
0.61	43.47	18.20	1.32	32.01	0.0832
0.56	40.29	15.11	1.32	58.76	0.0856
0.68	46.40	19.74	1.40	142.80	0.0866
0.63	44.48	17.06	1.41	60.86	0.0900
0.47	33.46	13.07	1.19	10.14	0.0791
0.62	43.74	17.19	1.37	59.85	0.0870
0.63	44.02	15.52	1.46	41.58	0.0944
0.43	29.97	11.35	1.18	9.20	0.0799
0.53	38.13	16.07	1.21	256.70	0.0783
0.56	40.49	17.49	1.24	123.69	0.0775
0.41	27.29	12.98	1.05	7.56	0.0694
0.47	33.12	13.98	1.14	14.25	0.0746
0.55	39.72	16.30	1.25	42.84	0.0796
0.64	44.72	18.26	1.37	91.80	0.0865
0.64	44.89	18.59	1.37	25.28	0.0866
0.67	46.11	15.94	1.54	59.85	0.1000
0.50	35.72	12.50	1.29	19.04	0.0867
0.45	31.82	13.88	1.11	10.98	0.0734
0.51	36.82	14.36	1.24	10.50	0.0814
0.51	36.99	12.68	1.32	35.65	0.0874
0.62	43.56	18.32	1.32	133.44	0.0833
0.51	37.00	13.43	1.28	34.32	0.0843
0.67	46.31	20.43	1.37	150.93	0.0848
0.53	38.25	13.42	1.33	52.80	0.0876
0.57	40.83	16.11	1.30	32.77	0.0830
0.40	26.15	10.09	1.16	9.54	0.0789
0.49	35.47	13.69	1.22	30.96	0.0803
0.58	41.45	14.52	1.39	59.70	0.0907
0.64	44.99	20.17	1.32	40.61	0.0825

0.53	38.50	15.41	1.25	29.26	0.0805
0.53	38.32	15.60	1.23	29.28	0.0790
0.44	30.35	13.66	1.09	4.64	0.0724
0.56	40.04	15.38	1.30	110.36	0.0832
0.46	32.42	11.89	1.22	11.34	0.0827
0.54	38.93	15.09	1.27	20.64	0.0824
0.71	47.73	22.98	1.35	86.48	0.0821
0.48	34.65	14.52	1.16	14.24	0.0757
0.60	42.44	14.78	1.42	46.69	0.0929
0.54	39.17	13.65	1.35	63.12	0.0888
0.54	39.29	15.80	1.26	30.51	0.0803
0.51	37.01	15.06	1.21	42.63	0.0783
0.58	41.68	16.27	1.33	50.40	0.0848
0.61	43.11	20.30	1.24	92.16	0.0766
0.62	43.61	19.35	1.29	57.46	0.0802
0.46	32.67	12.38	1.20	20.44	0.0801
0.59	42.36	15.42	1.39	42.60	0.0899
0.54	39.26	15.21	1.28	26.32	0.0822
0.63	44.15	19.97	1.29	16.40	0.0822
0.55	39.86	14.22	1.35	98.25	0.0881
0.45	31.43	12.38	1.17	10.80	0.0779
0.68	46.41	16.73	1.52	55.20	0.0971
0.48	34.38	14.24	1.17	11.68	0.0761
0.54	39.13	15.50	1.26	44.50	0.0813
0.70	47.25	19.22	1.46	41.80	0.0919
0.60	42.44	16.85	1.33	157.44	0.0847
0.54	38.82	15.25	1.26	22.00	0.0822
0.50	35.74	12.84	1.27	17.64	0.0844
0.62	43.61	18.94	1.30	45.60	0.0819
0.47	33.01	12.68	1.20	9.46	0.0793
0.42	28.79	11.20	1.16	14.00	0.0782
0.70	47.29	20.01	1.43	54.61	0.0888
0.48	34.33	13.63	1.19	25.06	0.0786
0.56	40.05	15.97	1.28	58.50	0.0819
0.68	46.60	18.26	1.46	967.68	0.0920
0.56	40.60	15.61	1.31	49.88	0.0842
0.57	41.11	13.18	1.45	40.64	0.0953
0.47	33.14	12.15	1.23	17.52	0.0825
0.52	37.28	14.52	1.24	21.20	0.0810
0.63	44.18	18.22	1.35	60.30	0.0858
0.51	36.57	14.46	1.22	11.16	0.0810
0.57	40.63	14.43	1.37	28.56	0.0897
0.50	36.00	12.70	1.29	32.12	0.0850
0.49	35.21	12.76	1.26	16.20	0.0835
0.62	43.83	18.51	1.33	161.50	0.0832
0.56	39.98	16.43	1.26	34.75	0.0803
0.52	37.54	10.37	1.48	35.00	0.1022
0.38	23.24	13.82	0.94	0.00	0.0613
0.58	41.48	16.34	1.31	43.16	0.0846
0.60	42.74	18.54	1.28	49.60	0.0804
0.46	32.57	12.53	1.19	19.80	0.0796

0.42	28.00	11.39	1.13	1.68	0.0775
0.53	38.19	15.22	1.24	24.15	0.0816
0.64	44.66	18.22	1.37	54.60	0.0859
0.58	41.24	17.35	1.27	200.98	0.0806
0.63	44.09	18.16	1.35	136.09	0.0856
0.61	43.11	16.97	1.35	25.28	0.0863
0.60	42.74	16.48	1.36	63.27	0.0860
0.59	42.37	13.41	1.49	13.55	0.0984
0.61	43.23	16.98	1.36	28.44	0.0860
0.41	27.11	10.95	1.13	5.15	0.0772
0.48	34.40	12.91	1.23	19.38	0.0811
0.62	43.58	17.52	1.35	58.09	0.0851
0.58	41.45	16.09	1.32	38.10	0.0847
0.57	41.16	15.66	1.33	141.71	0.0843
0.57	41.17	14.90	1.36	97.03	0.0881
0.53	37.90	13.67	1.30	44.72	0.0849
0.55	39.69	13.73	1.36	41.08	0.0895
0.64	44.70	17.41	1.40	54.06	0.0895
0.63	44.42	15.50	1.48	37.74	0.0952
0.54	38.62	14.73	1.28	25.48	0.0826
0.57	40.76	14.19	1.38	69.94	0.0907
0.57	41.18	13.16	1.45	38.75	0.0969
0.54	38.82	15.74	1.24	78.20	0.0805
0.66	45.80	17.43	1.46	87.60	0.0923
0.48	34.43	14.45	1.16	9.36	0.0762
0.50	35.73	13.60	1.24	24.48	0.0815
0.46	32.71	12.22	1.21	10.20	0.0807
0.55	39.58	11.84	1.46	31.97	0.0991
0.52	37.43	11.39	1.41	15.77	0.0959
0.57	40.63	15.74	1.31	29.28	0.0846
0.49	35.35	11.66	1.32	104.50	0.0888
0.57	40.65	14.81	1.35	57.12	0.0875
0.52	37.25	14.77	1.23	19.80	0.0797
0.53	38.56	13.82	1.32	71.80	0.0871
0.60	42.81	18.01	1.30	22.77	0.0819
0.65	45.17	19.13	1.36	48.64	0.0850
0.42	28.00	11.72	1.12	3.16	0.0760
0.50	36.00	13.78	1.24	24.14	0.0813
0.55	39.61	13.65	1.36	45.25	0.0899
0.39	25.03	11.12	1.08	5.96	0.0730
0.52	37.27	14.66	1.24	16.17	0.0803
0.67	46.00	19.09	1.40	55.48	0.0878
0.52	37.75	13.68	1.30	35.20	0.0852
0.62	43.82	17.31	1.37	23.78	0.0878
0.52	37.85	13.29	1.32	33.58	0.0869
0.50	36.00	11.47	1.35	35.20	0.0909
0.62	43.78	18.70	1.32	55.68	0.0828
0.49	35.43	12.99	1.25	11.04	0.0841
0.45	31.65	11.25	1.23	11.11	0.0837
0.48	34.63	15.16	1.14	19.65	0.0737
0.59	42.07	16.85	1.32	198.40	0.0837

0.50	36.28	14.02	1.23	28.00	0.0816
0.55	39.61	15.68	1.27	25.75	0.0819
0.61	43.03	17.93	1.31	48.84	0.0828
0.56	40.05	13.65	1.38	56.68	0.0909
0.50	36.00	13.18	1.26	13.32	0.0836
0.58	41.38	18.56	1.23	20.90	0.0781
0.48	34.00	13.85	1.17	11.88	0.0774
0.56	40.58	14.17	1.38	62.25	0.0905
0.50	36.25	13.43	1.26	39.82	0.0824
0.60	42.48	17.61	1.30	36.00	0.0826
0.56	40.44	16.36	1.28	98.24	0.0807
0.57	41.09	16.91	1.28	24.99	0.0818
0.52	37.50	13.01	1.32	17.82	0.0874
0.61	43.08	16.48	1.37	59.28	0.0869
0.60	42.48	16.83	1.33	38.10	0.0852
0.53	38.20	15.84	1.22	20.64	0.0780
0.50	36.00	14.10	1.22	36.00	0.0799
0.57	40.65	14.75	1.35	82.88	0.0877
0.49	35.36	13.56	1.23	19.20	0.0802
0.50	36.00	11.59	1.35	49.80	0.0907
0.50	36.00	16.59	1.13	12.00	0.0714
0.61	43.10	17.59	1.33	46.90	0.0837
0.63	44.21	18.56	1.34	120.62	0.0838
0.50	35.72	15.73	1.15	11.31	0.0745
0.48	34.13	12.79	1.23	34.68	0.0810
0.52	37.85	15.18	1.23	66.70	0.0795
0.67	46.21	23.60	1.27	39.52	0.0769
0.60	42.74	16.66	1.35	24.49	0.0864
0.65	45.26	17.58	1.42	21.46	0.0904
0.58	41.39	16.15	1.32	117.18	0.0842
0.58	41.37	16.07	1.32	65.28	0.0856
0.64	44.60	16.66	1.43	45.36	0.0929
0.51	36.53	15.08	1.20	11.90	0.0777
0.51	36.77	13.82	1.26	15.80	0.0825
0.53	38.08	13.82	1.30	32.87	0.0860
0.53	38.41	14.75	1.27	61.75	0.0821
0.53	38.16	14.58	1.27	66.08	0.0837
0.50	36.27	14.48	1.21	16.32	0.0795
0.56	40.39	16.39	1.27	49.92	0.0817
0.62	43.56	17.03	1.37	31.36	0.0874
0.55	39.75	16.73	1.24	26.62	0.0793
0.50	36.00	12.87	1.28	36.29	0.0847
0.58	41.78	16.15	1.33	46.08	0.0851
0.62	43.78	16.07	1.42	40.64	0.0916
0.55	39.72	15.80	1.27	31.08	0.0813
0.58	41.23	18.15	1.24	46.80	0.0776
0.47	33.12	14.48	1.12	29.55	0.0729
0.58	41.56	16.11	1.33	270.08	0.0846
0.56	40.44	14.06	1.38	34.56	0.0893
0.55	39.53	16.11	1.25	59.67	0.0799
0.63	44.09	19.26	1.31	51.12	0.0815

0.58	41.27	15.97	1.32	49.83	0.0841
0.58	41.43	17.17	1.28	78.20	0.0805
0.56	40.05	16.27	1.27	60.32	0.0809
0.50	36.00	13.28	1.26	19.76	0.0830
0.62	43.78	15.58	1.44	64.56	0.0925
0.37	22.03	10.15	1.07	0.00	0.0734
0.53	38.29	13.76	1.31	13.92	0.0882
0.54	39.04	15.10	1.28	18.69	0.0831
0.51	36.53	11.85	1.35	52.92	0.0911
0.67	46.00	17.48	1.46	93.44	0.0942
0.52	37.30	14.21	1.26	10.26	0.0824
0.49	35.50	12.23	1.30	12.10	0.0859
0.53	37.93	12.63	1.36	64.00	0.0898
0.51	37.01	13.53	1.28	19.74	0.0841
0.47	33.26	11.33	1.28	17.55	0.0862
0.39	24.83	9.40	1.17	1.86	0.0817
0.59	42.28	18.37	1.27	123.76	0.0801
0.49	34.95	13.17	1.23	16.92	0.0811
0.62	43.64	16.41	1.40	70.37	0.0901
0.59	42.05	18.22	1.27	25.20	0.0799
0.59	41.84	16.23	1.33	56.42	0.0852
0.59	42.18	16.31	1.34	84.80	0.0857
0.46	32.71	12.68	1.19	15.75	0.0787
0.52	37.84	15.91	1.21	45.90	0.0779
0.52	37.35	15.22	1.22	40.96	0.0794
0.47	33.75	12.77	1.22	11.05	0.0810
0.54	39.17	13.94	1.33	40.08	0.0875
0.58	41.65	16.39	1.32	30.78	0.0847
0.54	38.96	16.59	1.22	20.24	0.0776
0.56	40.00	14.35	1.35	42.88	0.0868
0.51	36.55	13.06	1.29	20.70	0.0860
0.59	42.25	17.48	1.30	12.54	0.0837
0.65	45.05	18.57	1.38	47.36	0.0864
0.59	42.17	15.42	1.38	50.40	0.0883
0.49	35.44	14.56	1.19	14.28	0.0777
0.48	34.40	13.83	1.19	25.33	0.0775
0.48	34.18	11.43	1.30	25.08	0.0871
0.53	38.30	11.34	1.45	28.13	0.0968
0.55	39.33	16.76	1.22	27.00	0.0785
0.53	38.31	15.71	1.23	63.60	0.0793
0.52	37.37	15.16	1.22	19.20	0.0798
0.70	47.54	20.98	1.41	65.32	0.0866
0.52	37.23	13.44	1.29	31.51	0.0847
0.63	44.48	19.68	1.31	118.32	0.0818
0.59	42.28	16.27	1.35	65.52	0.0855
0.46	32.38	11.76	1.23	7.48	0.0828
0.60	42.59	17.38	1.32	90.00	0.0837
0.49	34.97	13.31	1.23	9.40	0.0803
0.41	26.95	12.11	1.08	4.80	0.0719
0.53	38.05	16.04	1.21	35.20	0.0776
0.44	30.49	11.89	1.17	8.25	0.0783

0.51	36.96	11.78	1.37	63.25	0.0913
0.55	39.95	15.58	1.29	35.56	0.0827
0.56	40.29	17.78	1.22	40.56	0.0768
0.53	37.98	14.81	1.25	166.52	0.0812
0.60	42.52	15.06	1.41	103.70	0.0912
0.51	36.49	13.92	1.25	38.87	0.0809
0.59	42.29	17.19	1.31	38.75	0.0833
0.59	42.29	16.89	1.32	67.27	0.0843
0.52	37.75	15.36	1.22	102.74	0.0788
0.56	40.10	14.58	1.34	68.40	0.0883
0.57	40.76	12.65	1.46	25.22	0.0979
0.46	32.67	13.70	1.14	24.92	0.0749
0.55	39.50	13.50	1.37	49.06	0.0908
0.52	37.67	14.62	1.25	33.28	0.0806
0.54	39.12	13.80	1.34	23.75	0.0889
0.58	41.58	15.73	1.34	29.96	0.0867
0.44	30.46	11.72	1.18	9.31	0.0797
0.51	36.77	14.52	1.23	33.00	0.0798
0.58	41.52	16.89	1.29	19.14	0.0824
0.50	35.74	12.29	1.30	14.40	0.0869
0.56	40.05	16.24	1.27	43.89	0.0818
0.52	37.71	14.73	1.25	18.96	0.0806
0.61	43.47	16.46	1.39	44.77	0.0884
0.51	36.79	14.81	1.22	43.56	0.0791
0.44	30.77	11.65	1.19	6.02	0.0807
0.41	27.43	10.61	1.16	8.90	0.0795
0.54	39.17	13.65	1.35	27.84	0.0888
0.57	41.00	14.79	1.36	71.10	0.0883
0.51	36.53	12.74	1.30	45.90	0.0868
0.61	43.42	16.99	1.37	62.79	0.0861
0.59	42.19	18.16	1.27	31.72	0.0808
0.44	30.13	12.74	1.12	4.90	0.0752
0.70	47.52	24.91	1.29	103.36	0.0787
0.58	41.48	18.37	1.24	33.80	0.0782
0.59	41.93	16.36	1.33	44.16	0.0863
0.53	38.59	15.96	1.23	21.62	0.0787
0.53	38.44	13.52	1.33	65.80	0.0881
0.53	37.93	12.93	1.34	36.75	0.0884
0.52	37.54	13.33	1.31	49.60	0.0864
0.71	47.96	20.15	1.46	216.09	0.0900
0.62	43.91	18.32	1.34	37.29	0.0842
0.58	41.27	14.50	1.39	60.72	0.0897
0.55	39.37	15.69	1.26	15.25	0.0813
0.50	36.26	14.50	1.21	19.40	0.0787
0.55	39.87	13.92	1.36	47.95	0.0878
0.50	35.75	13.90	1.22	14.96	0.0794
0.57	41.06	17.71	1.25	45.75	0.0792
0.54	38.80	16.92	1.20	21.60	0.0760
0.57	40.63	17.58	1.24	32.40	0.0786
0.58	41.65	15.21	1.37	214.20	0.0879
0.70	47.58	22.76	1.35	58.09	0.0833

0.48	34.57	11.15	1.33	13.80	0.0909
0.61	43.44	18.99	1.29	41.72	0.0816
0.61	43.21	16.77	1.37	73.36	0.0879
0.62	43.87	20.86	1.25	120.90	0.0774
0.64	44.57	17.25	1.41	51.60	0.0887
0.47	33.63	12.46	1.23	16.56	0.0811
0.54	39.00	14.81	1.29	26.84	0.0840
0.61	42.98	15.72	1.40	89.32	0.0910
0.60	42.59	15.95	1.38	50.82	0.0881
0.49	35.29	13.03	1.25	9.84	0.0836
0.39	24.62	9.37	1.17	0.00	0.0820
0.70	47.30	19.74	1.44	63.00	0.0887
0.55	39.75	13.45	1.38	13.64	0.0917
0.54	39.00	14.81	1.29	100.32	0.0840
0.58	41.43	16.42	1.31	127.50	0.0829
0.49	35.21	13.16	1.24	37.80	0.0818
0.58	41.47	17.00	1.29	50.32	0.0807
0.59	42.30	17.45	1.30	29.24	0.0820
0.51	36.78	14.52	1.23	15.01	0.0800
0.58	41.71	14.75	1.39	26.40	0.0901
0.63	44.04	17.94	1.36	20.74	0.0856
0.71	47.81	19.74	1.46	48.88	0.0910
0.53	38.56	15.42	1.25	19.80	0.0809
0.46	32.76	12.45	1.20	21.12	0.0796
0.46	32.57	14.24	1.12	29.16	0.0731
0.67	46.00	20.44	1.35	98.28	0.0833
0.49	35.14	12.48	1.27	11.52	0.0857
0.53	37.90	12.94	1.34	19.76	0.0881
0.75	49.16	24.57	1.38	61.60	0.0821
0.43	29.54	12.20	1.13	5.74	0.0758
0.60	42.73	15.01	1.42	43.60	0.0911
0.56	40.00	15.78	1.28	119.07	0.0823
0.48	34.36	12.53	1.24	24.75	0.0830
0.50	36.00	14.24	1.22	25.56	0.0794
0.49	34.89	14.50	1.17	26.60	0.0766
0.52	37.79	15.66	1.21	50.60	0.0783
0.42	28.00	10.54	1.18	9.73	0.0805
0.46	32.90	13.12	1.18	26.39	0.0777
0.53	38.12	14.02	1.29	15.05	0.0855
0.55	39.81	15.66	1.28	23.14	0.0823
0.56	40.60	16.43	1.28	67.28	0.0813
0.46	32.29	12.01	1.21	9.72	0.0813
0.44	30.77	11.33	1.21	9.31	0.0822
0.54	38.65	16.38	1.21	34.25	0.0772
0.50	36.00	14.52	1.20	31.50	0.0783
0.57	41.12	15.05	1.36	29.04	0.0886
0.49	35.43	11.53	1.33	15.48	0.0911
0.52	37.62	13.27	1.31	15.04	0.0877
0.57	40.88	15.68	1.32	62.72	0.0849
0.72	48.08	20.61	1.45	125.76	0.0893
0.55	39.90	15.12	1.31	30.24	0.0849

0.65	45.39	18.96	1.38	54.80	0.0859
0.55	39.91	15.35	1.30	29.87	0.0832
0.56	40.60	15.17	1.33	31.03	0.0858
0.41	27.21	12.88	1.05	0.00	0.0704
0.62	43.74	16.89	1.38	53.55	0.0880
0.55	39.92	14.66	1.33	34.19	0.0866
0.60	42.67	17.19	1.33	42.56	0.0842
0.56	40.05	17.43	1.22	34.58	0.0773
0.52	37.48	13.17	1.31	19.55	0.0864
0.56	40.14	15.54	1.30	78.59	0.0832
0.58	41.38	16.16	1.32	107.45	0.0835
0.47	33.14	12.74	1.20	25.20	0.0800
0.70	47.31	19.28	1.46	59.04	0.0915
0.57	40.94	17.23	1.26	56.16	0.0800
0.61	43.11	20.05	1.25	71.68	0.0772
0.57	41.13	14.71	1.37	22.20	0.0908
0.47	33.71	13.73	1.17	15.48	0.0773
0.60	42.74	16.19	1.37	54.25	0.0880
0.51	37.07	14.78	1.23	99.28	0.0801
0.42	28.31	10.20	1.20	10.57	0.0828
0.60	42.88	18.62	1.28	52.85	0.0800
0.52	37.56	12.96	1.33	14.25	0.0884
0.56	40.05	15.39	1.30	32.76	0.0839
0.72	48.14	19.66	1.49	36.40	0.0936
0.61	43.42	15.91	1.41	25.73	0.0912
0.53	38.20	15.17	1.25	31.44	0.0802
0.55	39.41	15.69	1.27	44.01	0.0810
0.52	37.67	13.42	1.31	58.50	0.0854
0.58	41.24	16.71	1.29	44.98	0.0827
0.54	38.82	14.10	1.31	22.40	0.0866
0.61	43.27	19.42	1.27	31.50	0.0796
0.61	43.31	20.01	1.26	61.07	0.0782
0.59	41.88	17.38	1.29	40.77	0.0821
0.62	43.56	18.32	1.32	100.80	0.0833
0.53	38.11	13.06	1.34	16.38	0.0895
0.54	39.04	15.10	1.28	57.96	0.0831
0.50	36.26	13.07	1.28	12.78	0.0847
0.55	39.66	15.72	1.27	29.28	0.0821
0.50	35.73	14.21	1.21	22.88	0.0792
0.60	42.44	16.85	1.33	45.44	0.0847
0.60	42.67	18.37	1.28	298.56	0.0805
0.63	44.00	18.51	1.33	64.01	0.0833
0.62	43.83	20.05	1.27	41.14	0.0789
0.59	42.38	16.23	1.36	64.44	0.0860
0.54	39.26	14.20	1.33	42.28	0.0860
0.46	32.29	15.08	1.08	5.04	0.0698
0.51	37.11	15.67	1.19	20.30	0.0776
0.51	36.50	13.94	1.24	18.26	0.0810
0.52	37.19	14.09	1.26	115.44	0.0814
0.57	40.76	16.66	1.28	34.32	0.0815
0.61	43.37	16.11	1.40	66.23	0.0893

0.59	42.22	16.52	1.34	101.12	0.0852
0.57	40.76	16.04	1.30	56.16	0.0836
0.48	34.31	13.20	1.21	10.14	0.0804
0.59	42.21	15.90	1.36	56.84	0.0878
0.61	43.37	16.11	1.40	91.02	0.0893
0.56	40.52	16.93	1.26	158.08	0.0800
0.46	32.23	11.62	1.23	12.54	0.0832
0.53	38.56	14.14	1.30	59.00	0.0858
0.59	42.29	16.00	1.36	46.19	0.0873
0.61	43.03	17.48	1.33	49.83	0.0842
0.50	36.00	13.43	1.25	59.84	0.0818
0.57	40.76	16.04	1.30	73.32	0.0836
0.49	34.84	13.97	1.19	12.32	0.0790
0.56	40.29	16.89	1.25	77.48	0.0795
0.57	41.11	15.76	1.32	75.20	0.0846
0.55	39.90	15.27	1.30	16.72	0.0852
0.67	46.00	18.89	1.41	96.00	0.0881
0.50	36.27	15.43	1.18	16.48	0.0762
0.60	42.74	16.23	1.37	32.56	0.0869
0.59	42.22	12.53	1.53	71.36	0.1024
0.58	41.52	15.70	1.34	37.70	0.0864
0.45	32.00	12.65	1.17	8.40	0.0789
0.56	40.10	14.58	1.34	55.40	0.0883
0.66	45.57	20.52	1.33	47.52	0.0826
0.62	43.91	17.03	1.39	17.82	0.0884
0.66	45.80	17.52	1.45	69.96	0.0913
0.46	32.76	12.94	1.18	54.40	0.0776
0.46	32.71	14.09	1.13	17.16	0.0739
0.62	43.66	19.36	1.29	54.00	0.0800
0.60	42.93	18.88	1.28	31.20	0.0801
0.62	43.59	19.26	1.29	49.01	0.0811
0.61	43.33	18.89	1.29	48.96	0.0809
0.46	32.15	11.48	1.24	11.69	0.0840
0.60	42.44	16.07	1.36	45.24	0.0879
0.55	39.81	15.95	1.27	27.82	0.0813
0.61	43.42	15.47	1.43	44.07	0.0916
0.53	38.44	14.79	1.27	33.60	0.0823
0.64	44.60	17.99	1.38	60.90	0.0871
0.53	38.56	15.42	1.25	28.20	0.0809
0.51	36.86	14.47	1.23	43.24	0.0797
0.60	42.74	16.47	1.36	86.70	0.0865
0.58	41.48	18.04	1.25	24.96	0.0792
0.59	42.21	16.53	1.34	49.30	0.0855
0.60	42.74	19.13	1.26	116.87	0.0788
0.51	37.05	13.88	1.26	13.32	0.0833
0.60	42.44	16.07	1.36	63.51	0.0879
0.65	45.09	20.27	1.32	27.60	0.0827
0.53	38.00	14.24	1.28	38.28	0.0836
0.64	44.82	18.04	1.39	50.75	0.0876
0.56	40.44	12.39	1.47	21.16	0.0989
0.45	31.82	12.34	1.18	9.00	0.0794

0.55	39.66	14.81	1.31	21.36	0.0854
0.53	38.20	15.31	1.24	42.24	0.0798
0.67	46.30	17.19	1.49	73.53	0.0945
0.54	38.82	14.07	1.32	47.52	0.0855
0.66	45.47	21.98	1.28	74.00	0.0785
0.59	42.38	17.49	1.30	104.40	0.0818
0.62	43.50	16.76	1.38	63.30	0.0885
0.58	41.68	15.10	1.38	39.90	0.0890
0.49	35.31	12.56	1.27	20.30	0.0853
0.56	40.19	17.93	1.21	36.12	0.0758
0.42	28.38	10.67	1.18	6.10	0.0810
0.61	43.19	19.28	1.27	37.82	0.0796
0.56	40.00	16.19	1.27	35.10	0.0808
0.53	38.08	16.07	1.21	47.50	0.0778
0.56	40.29	16.00	1.28	26.52	0.0824
0.51	36.51	12.32	1.32	62.00	0.0884
0.62	43.67	18.25	1.33	89.32	0.0845
0.65	45.17	18.82	1.37	41.80	0.0859
0.60	42.45	19.23	1.25	36.40	0.0771
0.67	46.20	17.99	1.45	58.40	0.0918
0.59	42.32	16.82	1.33	26.68	0.0849
0.41	27.75	12.24	1.09	3.90	0.0728
0.54	38.76	15.76	1.24	75.11	0.0788
0.51	37.11	15.12	1.21	9.94	0.0795
0.50	36.00	12.21	1.31	12.58	0.0882
0.55	39.59	17.11	1.22	23.80	0.0780
0.56	40.10	15.42	1.30	78.75	0.0841
0.55	39.90	18.51	1.18	67.92	0.0742
0.67	46.00	20.59	1.35	162.96	0.0829
0.57	40.72	15.50	1.32	48.05	0.0846
0.54	38.71	14.49	1.29	38.88	0.0836
0.54	39.22	14.40	1.31	49.59	0.0849
0.57	40.78	17.41	1.25	20.40	0.0780
0.63	44.00	16.74	1.40	27.52	0.0899
0.56	40.05	15.10	1.31	23.66	0.0850
0.62	43.69	15.74	1.43	89.76	0.0924
0.65	45.17	17.00	1.44	118.56	0.0919
0.53	38.50	15.44	1.25	9.24	0.0818
0.55	39.56	14.28	1.33	116.80	0.0859
0.54	39.08	17.75	1.18	25.40	0.0749
0.66	45.48	19.25	1.37	66.69	0.0855
0.56	40.24	17.09	1.24	63.72	0.0786
0.58	41.35	15.96	1.33	22.68	0.0852
0.56	40.43	15.91	1.29	58.50	0.0825
0.56	40.49	15.97	1.29	42.78	0.0823
0.49	35.15	13.12	1.24	27.95	0.0827
0.56	40.24	18.51	1.19	44.55	0.0745
0.63	44.00	17.41	1.37	23.04	0.0875
0.61	43.08	15.21	1.43	34.58	0.0917
0.60	42.55	15.52	1.39	24.07	0.0903
0.71	47.69	21.00	1.41	63.55	0.0893

0.39	24.10	11.84	1.03	0.00	0.0693
0.48	34.68	13.44	1.21	30.42	0.0794
0.57	40.71	16.00	1.30	57.24	0.0834
0.47	33.78	14.24	1.15	15.68	0.0752
0.54	38.96	14.22	1.31	23.92	0.0860
0.51	37.07	12.85	1.31	70.72	0.0879
0.57	41.12	17.39	1.26	50.68	0.0798
0.61	43.05	16.07	1.39	27.60	0.0896
0.57	40.65	14.24	1.38	35.84	0.0898
0.49	35.39	13.67	1.22	12.00	0.0820
0.57	41.00	13.22	1.44	41.08	0.0958
0.58	41.65	16.73	1.31	45.22	0.0825
0.53	38.44	16.15	1.22	59.04	0.0775
0.59	42.36	16.04	1.36	51.60	0.0876
0.59	42.22	16.80	1.33	54.72	0.0842
0.50	36.27	15.42	1.18	25.67	0.0760
0.54	38.93	16.52	1.22	14.88	0.0776
0.52	37.69	17.89	1.13	31.20	0.0725
0.41	27.67	13.12	1.05	3.96	0.0699
0.58	41.75	18.74	1.24	22.62	0.0774
0.60	42.81	14.22	1.47	47.52	0.0953
0.52	37.73	13.70	1.30	28.29	0.0849
0.59	41.91	19.56	1.22	32.70	0.0755
0.46	32.06	13.55	1.13	8.08	0.0753
0.44	30.56	11.66	1.18	8.30	0.0796
0.54	39.10	16.38	1.23	25.48	0.0781
0.62	43.78	19.52	1.29	53.44	0.0805
0.47	33.10	11.41	1.27	35.55	0.0854
0.58	41.78	14.79	1.39	55.68	0.0903
0.63	44.26	18.64	1.34	31.96	0.0842
0.53	38.53	14.07	1.31	17.22	0.0858
0.56	40.53	15.51	1.31	72.80	0.0845
0.54	38.86	14.49	1.30	35.62	0.0841
0.56	40.60	14.79	1.35	53.07	0.0873
0.61	43.33	16.06	1.40	158.08	0.0902
0.47	33.47	11.63	1.26	15.47	0.0847
0.50	36.37	14.34	1.22	58.65	0.0790
0.54	39.29	16.59	1.23	55.35	0.0778
0.53	38.34	15.42	1.24	42.56	0.0806
0.52	37.84	13.12	1.33	21.24	0.0886
0.52	37.75	15.31	1.23	18.04	0.0790
0.68	46.57	22.65	1.31	51.84	0.0788
0.56	40.24	17.09	1.24	36.99	0.0786
0.51	36.78	14.81	1.22	34.39	0.0789
0.61	43.01	19.63	1.26	31.32	0.0784
0.53	38.25	17.43	1.16	33.00	0.0736
0.58	41.52	13.93	1.43	52.78	0.0937
0.51	37.00	14.36	1.24	38.72	0.0806
0.52	37.32	13.54	1.29	49.68	0.0854
0.54	38.82	13.45	1.35	42.00	0.0894
0.62	43.73	17.46	1.36	40.50	0.0868

0.55	39.33	15.06	1.29	34.32	0.0832
0.54	39.29	16.40	1.23	26.46	0.0793
0.66	45.47	20.04	1.34	48.10	0.0835
0.61	43.05	17.06	1.35	74.40	0.0861
0.58	41.56	16.25	1.32	56.96	0.0841
0.59	42.35	15.05	1.41	39.96	0.0919
0.55	39.56	14.88	1.31	19.80	0.0865
0.47	33.57	12.02	1.25	37.92	0.0834
0.61	43.42	17.22	1.36	53.63	0.0865
0.56	40.24	15.66	1.30	29.70	0.0833
0.42	28.92	11.31	1.16	10.29	0.0786
0.48	34.13	13.18	1.21	132.60	0.0794
0.56	40.42	15.91	1.29	45.92	0.0828
0.61	43.42	19.35	1.28	32.55	0.0800
0.50	35.87	14.67	1.19	20.06	0.0777
0.63	44.22	20.11	1.29	63.04	0.0802
0.48	34.11	12.38	1.24	15.20	0.0829
0.56	40.55	16.86	1.26	87.90	0.0797
0.46	32.94	13.72	1.15	21.70	0.0753
0.53	38.16	14.58	1.27	29.60	0.0837
0.49	35.43	15.37	1.15	14.16	0.0752
0.53	38.05	13.60	1.31	33.20	0.0866
0.47	33.50	14.38	1.14	49.84	0.0741
0.60	42.67	16.03	1.37	35.88	0.0892
0.58	41.54	17.61	1.27	61.50	0.0808
0.57	40.65	13.95	1.39	301.56	0.0910
0.59	41.96	16.26	1.34	60.12	0.0846
0.49	35.19	14.67	1.17	65.44	0.0764
0.62	43.96	21.16	1.24	120.05	0.0763
0.44	30.78	11.46	1.20	12.87	0.0808
0.57	41.17	16.38	1.30	52.39	0.0828
0.49	35.21	12.76	1.26	14.58	0.0835
0.64	44.57	16.70	1.43	62.00	0.0906
0.57	40.65	15.95	1.30	61.60	0.0833
0.56	40.23	14.92	1.33	59.80	0.0873
0.48	34.06	13.94	1.17	15.40	0.0769
0.48	34.67	12.89	1.24	15.98	0.0818
0.72	48.04	21.00	1.43	61.65	0.0884
0.53	38.47	14.81	1.27	30.13	0.0824
0.61	43.35	19.29	1.28	31.50	0.0810
0.57	40.82	16.55	1.28	40.00	0.0822
0.49	35.46	13.33	1.24	13.44	0.0820
0.59	42.07	17.14	1.31	171.43	0.0828
0.49	34.97	14.40	1.18	13.40	0.0761
0.46	32.29	15.08	1.08	5.04	0.0698
0.55	39.49	16.54	1.24	82.32	0.0783
0.59	41.98	17.89	1.27	56.26	0.0805
0.59	42.14	18.14	1.27	79.80	0.0800
0.59	41.98	17.12	1.30	66.99	0.0829
0.48	34.00	15.55	1.11	16.44	0.0717
0.52	37.65	14.18	1.27	28.35	0.0821

0.58	41.58	16.26	1.32	65.80	0.0848
0.56	40.10	18.22	1.20	58.60	0.0761
0.40	26.16	11.62	1.08	2.73	0.0730
0.54	38.82	15.42	1.26	25.60	0.0816
0.51	37.04	13.60	1.28	22.80	0.0842
0.61	42.96	18.22	1.30	65.62	0.0815
0.58	41.58	18.51	1.24	20.72	0.0778
0.54	38.86	14.75	1.29	25.22	0.0831
0.44	30.93	12.20	1.17	9.99	0.0782
0.55	39.90	17.89	1.20	37.68	0.0759
0.46	32.99	13.70	1.15	18.00	0.0753
0.59	42.36	15.27	1.40	54.60	0.0905
0.44	30.29	13.40	1.10	3.00	0.0730
0.51	37.05	12.65	1.32	22.14	0.0886
0.49	35.19	14.52	1.18	56.00	0.0769
0.67	46.11	20.40	1.36	42.78	0.0855
0.63	44.13	13.45	1.57	30.36	0.1043
0.67	46.10	20.67	1.35	166.46	0.0832
0.46	32.06	13.45	1.14	6.88	0.0757
0.51	36.53	14.81	1.21	43.56	0.0785
0.59	42.19	18.86	1.25	42.90	0.0788
0.66	45.51	18.61	1.40	100.80	0.0867
0.55	39.72	17.12	1.22	18.60	0.0783
0.53	38.09	14.09	1.29	24.64	0.0834
0.56	40.47	18.59	1.20	26.73	0.0749
0.45	31.82	12.55	1.17	11.25	0.0785
0.59	42.22	16.52	1.34	53.76	0.0852
0.57	41.18	17.27	1.27	40.50	0.0805
0.55	39.88	15.68	1.28	36.74	0.0831
0.53	38.05	14.67	1.26	369.25	0.0815
0.57	40.65	14.52	1.36	59.36	0.0887
0.51	36.90	13.36	1.28	25.20	0.0847
0.64	44.51	16.35	1.44	129.60	0.0924
0.57	40.77	13.70	1.41	32.40	0.0922
0.55	39.66	16.63	1.24	45.36	0.0791
0.53	38.28	15.72	1.23	40.95	0.0791
0.55	39.81	14.24	1.34	26.26	0.0877
0.64	44.69	21.48	1.26	85.28	0.0768
0.70	47.55	22.45	1.36	59.28	0.0837
0.58	41.56	16.11	1.33	41.28	0.0846
0.58	41.54	18.47	1.24	35.25	0.0783
0.54	38.79	15.14	1.27	21.28	0.0812
0.55	39.95	14.64	1.33	49.00	0.0862
0.54	38.67	14.58	1.29	23.97	0.0849
0.54	38.67	14.58	1.29	13.09	0.0849
0.58	41.37	13.20	1.46	56.16	0.0975
0.62	43.91	18.60	1.33	33.33	0.0834
0.48	33.97	11.81	1.27	6.82	0.0863
0.32	13.50	8.30	1.02	-5.90	0.0730
0.59	42.21	17.63	1.29	37.12	0.0819
0.60	42.82	16.05	1.38	32.94	0.0895

0.54	38.65	15.31	1.26	38.50	0.0807
0.54	38.62	13.20	1.35	49.40	0.0888
0.53	38.14	13.68	1.31	24.70	0.0855
0.61	43.24	18.32	1.31	65.25	0.0828
0.54	38.63	16.46	1.21	68.86	0.0773
0.55	39.81	13.95	1.36	51.74	0.0889
0.56	40.05	17.72	1.21	44.20	0.0764
0.41	27.34	9.47	1.23	12.42	0.0847
0.48	34.53	16.94	1.07	15.30	0.0690
0.50	36.28	14.36	1.22	18.06	0.0803
0.57	40.72	16.28	1.29	68.20	0.0819
0.51	36.53	15.41	1.18	37.26	0.0765
0.55	39.81	16.94	1.23	93.60	0.0781
0.48	34.31	15.27	1.13	10.40	0.0730
0.49	35.46	12.15	1.30	14.24	0.0873
0.57	40.72	13.95	1.39	61.38	0.0907
0.52	37.69	14.43	1.26	89.75	0.0815
0.49	34.89	12.98	1.24	34.96	0.0809
0.48	34.42	14.07	1.18	12.60	0.0765
0.72	48.11	23.07	1.37	92.82	0.0825
0.45	31.71	10.20	1.30	21.36	0.0892
0.54	39.26	14.70	1.30	44.80	0.0840
0.49	34.89	13.57	1.21	19.74	0.0801
0.55	39.33	13.28	1.37	85.80	0.0905
0.51	36.50	14.70	1.21	28.38	0.0782
0.43	29.55	12.73	1.11	4.80	0.0743
0.63	44.24	22.96	1.21	40.77	0.0741
0.62	43.79	16.75	1.39	59.20	0.0884
0.55	39.76	16.15	1.26	26.46	0.0804
0.58	41.78	17.29	1.29	21.75	0.0825
0.51	36.51	12.59	1.31	14.40	0.0871
0.58	41.75	16.32	1.33	62.06	0.0849
0.47	33.60	12.69	1.21	49.98	0.0803
0.56	40.52	15.36	1.32	24.50	0.0839
0.60	42.67	17.33	1.32	50.56	0.0837
0.57	41.18	17.58	1.26	32.13	0.0796
0.62	43.59	13.56	1.54	75.11	0.1025
0.58	41.80	18.95	1.23	34.80	0.0768
0.46	32.16	11.82	1.22	19.65	0.0813
0.48	34.33	13.93	1.18	12.60	0.0775
0.73	48.63	22.76	1.41	38.85	0.0871
0.55	39.61	15.39	1.29	37.50	0.0829
0.53	37.99	15.36	1.23	28.58	0.0793
0.76	49.59	21.86	1.49	106.20	0.0920
0.60	42.44	16.39	1.35	43.50	0.0867
0.59	41.84	16.80	1.31	59.83	0.0832
0.49	35.20	13.68	1.22	35.70	0.0798
0.44	30.15	11.94	1.16	12.04	0.0781
0.57	40.77	15.58	1.32	83.10	0.0846
0.41	27.64	11.84	1.10	2.67	0.0746
0.65	45.23	17.46	1.43	135.24	0.0893

0.47	33.22	13.14	1.18	17.50	0.0781
0.64	44.93	18.55	1.37	105.75	0.0849
0.53	38.05	16.97	1.17	24.60	0.0748
0.66	45.70	17.78	1.44	40.18	0.0906
0.57	40.72	16.41	1.28	30.38	0.0814
0.57	41.12	16.00	1.31	95.20	0.0844
0.59	42.02	14.32	1.43	50.75	0.0925
0.56	40.55	15.01	1.34	38.70	0.0861
0.55	39.63	15.91	1.26	14.96	0.0817
0.57	41.00	17.00	1.27	67.34	0.0811
0.52	37.25	16.11	1.18	37.18	0.0752
0.55	39.81	15.95	1.27	35.36	0.0813
0.59	42.35	17.10	1.32	55.08	0.0844
0.42	28.19	10.61	1.18	4.98	0.0807
0.74	48.86	21.64	1.45	57.24	0.0885
0.64	44.84	18.20	1.38	61.05	0.0869
0.60	42.89	15.87	1.39	24.00	0.0895
0.55	39.57	15.64	1.27	23.40	0.0818
0.68	46.48	19.35	1.41	71.76	0.0875
0.67	46.30	17.79	1.47	126.28	0.0927
0.59	41.81	16.06	1.34	90.44	0.0862
0.64	44.82	18.70	1.36	45.50	0.0856
0.63	44.05	18.99	1.32	43.21	0.0832
0.49	34.90	12.44	1.27	16.95	0.0847
0.66	45.80	17.34	1.46	65.20	0.0926
0.50	35.74	14.80	1.18	27.36	0.0768
0.52	37.51	14.89	1.24	12.60	0.0811
0.50	35.74	14.63	1.19	22.00	0.0771
0.48	33.91	11.24	1.30	12.51	0.0895
0.53	38.11	13.40	1.32	21.96	0.0881
0.59	41.93	16.65	1.32	133.98	0.0837
0.61	42.96	16.80	1.35	87.38	0.0860
0.54	38.68	13.68	1.33	41.04	0.0873
0.55	39.41	14.81	1.30	44.88	0.0847
0.54	39.10	14.77	1.29	37.96	0.0837
0.52	37.73	13.94	1.28	54.05	0.0839
0.53	38.28	17.38	1.17	24.36	0.0739
0.53	38.50	15.85	1.23	22.22	0.0790
0.48	34.12	14.25	1.16	20.13	0.0764
0.49	35.47	14.52	1.19	15.30	0.0773
0.56	40.29	14.81	1.33	31.98	0.0868
0.56	40.05	13.65	1.38	30.68	0.0909
0.49	35.13	14.70	1.17	8.14	0.0770
0.56	40.35	15.47	1.31	76.68	0.0843
0.50	35.73	14.17	1.21	13.80	0.0796
0.48	34.40	13.57	1.20	23.63	0.0785
0.52	37.73	12.89	1.34	27.37	0.0884
0.61	43.07	15.85	1.40	22.56	0.0916
0.53	38.35	15.72	1.23	38.07	0.0783
0.54	38.62	14.47	1.29	54.34	0.0836
0.51	36.75	15.76	1.18	23.10	0.0752

0.56	40.29	15.94	1.29	48.62	0.0826
0.47	33.46	11.91	1.25	20.54	0.0842
0.59	42.22	17.37	1.30	77.12	0.0823
0.53	38.53	12.96	1.36	26.04	0.0907
0.47	33.16	13.13	1.18	9.54	0.0790
0.54	38.72	15.28	1.26	23.23	0.0813
0.53	38.00	14.81	1.25	41.58	0.0814
0.52	37.18	15.74	1.19	28.08	0.0769
0.69	46.85	19.83	1.41	58.32	0.0889
0.58	41.35	16.48	1.30	48.44	0.0834
0.65	45.26	20.24	1.33	97.68	0.0822
0.63	44.16	17.65	1.37	80.00	0.0861
0.61	43.45	16.47	1.39	44.64	0.0884
0.55	39.73	12.26	1.44	46.50	0.0969
0.42	28.70	10.88	1.18	6.30	0.0805
0.47	33.71	13.51	1.18	8.88	0.0781
0.63	44.40	18.47	1.35	128.88	0.0848
0.60	42.89	17.53	1.32	26.88	0.0838
0.54	38.67	17.13	1.19	20.74	0.0762
0.58	41.43	17.86	1.26	28.98	0.0801
0.53	38.31	15.74	1.23	26.00	0.0792
0.59	42.05	16.39	1.33	34.72	0.0857
0.56	40.42	17.87	1.22	56.28	0.0766
0.54	38.86	17.78	1.17	21.09	0.0745
0.50	36.00	14.46	1.21	37.35	0.0791
0.59	42.09	18.04	1.27	28.42	0.0804
0.59	42.32	16.54	1.34	45.82	0.0858
0.53	38.17	16.15	1.21	31.75	0.0767
0.59	41.96	15.01	1.39	52.20	0.0893
0.54	38.89	15.06	1.27	28.25	0.0822
0.58	41.53	15.29	1.36	36.36	0.0869
0.75	49.20	18.70	1.58	99.96	0.1009
0.63	44.37	17.59	1.38	37.60	0.0869
0.57	41.00	17.80	1.24	82.50	0.0780
0.58	41.48	17.71	1.26	98.54	0.0801
0.63	44.30	17.84	1.37	72.00	0.0865
0.48	34.68	12.79	1.24	18.18	0.0821
0.52	37.77	13.07	1.33	116.13	0.0880
0.51	37.03	13.07	1.30	23.20	0.0862
0.50	36.00	11.26	1.37	12.41	0.0930
0.58	41.45	17.09	1.28	33.90	0.0814
0.57	41.11	17.57	1.25	146.88	0.0787
0.53	38.18	15.80	1.22	54.40	0.0787
0.58	41.49	18.35	1.24	42.24	0.0773
0.56	40.29	17.48	1.23	26.78	0.0777
0.60	42.88	17.25	1.33	54.95	0.0842
0.67	46.00	21.10	1.33	45.22	0.0822
0.55	39.95	16.07	1.27	46.46	0.0818
0.53	38.41	13.50	1.33	27.51	0.0879
0.66	45.57	20.97	1.32	40.60	0.0816
0.58	41.23	16.75	1.29	47.10	0.0818

0.51	36.51	14.24	1.23	21.80	0.0802
0.61	43.47	18.71	1.30	88.80	0.0811
0.56	40.00	15.83	1.28	12.42	0.0821
0.55	39.90	13.78	1.37	22.99	0.0912
0.72	48.34	27.31	1.27	81.00	0.0763
0.51	36.75	12.15	1.34	14.96	0.0894
0.66	45.57	20.04	1.35	68.00	0.0842
0.57	41.12	15.44	1.34	60.48	0.0864
0.49	35.48	14.22	1.20	31.92	0.0781
0.57	41.18	15.45	1.34	66.69	0.0867
0.52	37.22	14.21	1.25	20.80	0.0827
0.53	38.08	15.74	1.22	26.79	0.0788
0.59	42.36	16.97	1.32	41.70	0.0844
0.58	41.73	15.40	1.36	123.69	0.0879
0.47	33.40	12.47	1.22	17.48	0.0804
0.55	39.44	16.02	1.25	77.55	0.0805
0.58	41.75	17.08	1.30	42.05	0.0824
0.58	41.24	14.17	1.40	60.00	0.0926
0.53	37.93	15.21	1.24	77.50	0.0793
0.67	46.00	19.43	1.39	35.52	0.0877
0.60	42.74	17.59	1.32	77.18	0.0828
0.61	43.10	19.54	1.26	71.75	0.0780
0.63	44.21	18.55	1.34	77.33	0.0838
0.52	37.65	12.47	1.35	19.71	0.0894
0.62	43.63	16.28	1.40	191.49	0.0892
0.61	43.03	15.64	1.41	55.77	0.0906
0.47	33.14	9.78	1.37	9.12	0.0954
0.62	43.66	16.80	1.38	65.16	0.0879
0.50	35.72	15.25	1.17	21.14	0.0759
0.54	38.65	13.96	1.31	22.50	0.0859
0.59	41.93	15.33	1.38	21.45	0.0884
0.54	39.04	12.85	1.38	60.48	0.0926
0.75	49.20	22.86	1.43	49.14	0.0882
0.52	37.60	12.06	1.38	22.78	0.0932
0.56	40.05	14.81	1.33	32.50	0.0861
0.48	34.55	13.68	1.20	21.34	0.0795
0.51	36.53	14.22	1.23	15.84	0.0807
0.61	42.96	14.81	1.44	51.00	0.0936
0.57	40.88	17.41	1.25	22.56	0.0798
0.59	41.95	14.02	1.44	35.10	0.0952
0.57	41.00	16.89	1.28	32.64	0.0802
0.57	40.82	14.04	1.39	45.00	0.0917
0.68	46.40	20.98	1.35	71.40	0.0832
0.63	44.00	17.09	1.39	207.20	0.0879
0.60	42.81	18.01	1.30	43.56	0.0819
0.60	42.40	16.80	1.33	32.20	0.0853
0.57	40.83	15.91	1.31	58.58	0.0837
0.58	41.23	18.15	1.24	23.40	0.0776
0.49	35.17	12.18	1.29	18.20	0.0868
0.51	36.52	14.04	1.24	22.61	0.0812
0.69	47.13	21.32	1.38	90.87	0.0852

0.64	44.99	20.17	1.32	85.87	0.0825
0.59	41.96	19.29	1.23	81.72	0.0755
0.42	27.97	9.99	1.21	8.58	0.0821
0.54	39.00	13.57	1.35	25.96	0.0890
0.53	38.03	12.44	1.37	68.25	0.0917
0.53	38.40	18.91	1.12	14.28	0.0708
0.62	43.78	18.70	1.32	33.92	0.0828
0.51	37.13	14.46	1.24	100.23	0.0822
0.62	43.74	15.76	1.43	47.60	0.0922
0.62	43.53	15.97	1.41	71.75	0.0907
0.60	42.88	16.98	1.34	22.40	0.0851
0.52	37.25	16.52	1.17	28.82	0.0740
0.53	38.32	14.91	1.26	26.40	0.0815
0.62	43.84	17.12	1.38	43.29	0.0869
0.53	38.28	15.12	1.25	55.86	0.0812
0.59	42.35	17.37	1.31	32.40	0.0835
0.47	33.84	12.38	1.24	15.68	0.0823
0.58	41.29	15.10	1.36	43.50	0.0880
0.68	46.67	21.63	1.34	47.47	0.0817
0.44	30.63	11.39	1.20	13.95	0.0812
0.56	40.05	16.27	1.27	68.12	0.0809
0.62	43.66	16.36	1.40	45.57	0.0876
0.47	33.71	12.34	1.24	13.92	0.0830
0.63	44.22	18.12	1.36	118.08	0.0859
0.64	44.84	18.82	1.36	36.26	0.0850
0.51	37.11	15.31	1.21	22.54	0.0789
0.47	33.33	12.21	1.23	13.94	0.0818
0.55	39.53	16.38	1.24	54.27	0.0790
0.51	37.13	14.52	1.24	20.46	0.0803
0.60	42.48	18.61	1.27	92.73	0.0792
0.47	33.06	13.82	1.15	15.70	0.0760
0.58	41.49	15.50	1.35	100.98	0.0865
0.51	37.01	15.88	1.18	30.66	0.0755
0.63	44.30	18.44	1.35	77.04	0.0846
0.53	38.50	15.47	1.24	31.90	0.0803
0.44	30.46	15.73	1.02	7.07	0.0655
0.47	33.78	14.24	1.15	19.04	0.0752
0.51	36.90	14.10	1.25	36.80	0.0817
0.47	33.46	13.07	1.19	12.09	0.0791
0.56	40.32	14.73	1.34	74.70	0.0865
0.48	34.38	11.66	1.29	31.84	0.0869
0.51	36.53	15.11	1.20	28.98	0.0775
0.58	41.81	17.29	1.29	80.15	0.0810
0.43	29.85	11.26	1.18	8.68	0.0806
0.60	42.67	16.40	1.36	91.78	0.0879
0.62	43.87	19.34	1.30	194.04	0.0807
0.53	38.20	16.73	1.19	14.16	0.0752
0.49	35.22	16.28	1.12	14.44	0.0708
0.57	40.94	15.72	1.32	43.20	0.0851
0.60	42.44	17.67	1.30	59.74	0.0825
0.54	39.14	15.07	1.28	24.64	0.0824

0.44	30.39	12.17	1.15	5.12	0.0774
0.47	33.14	13.04	1.19	24.84	0.0787
0.55	39.66	14.81	1.31	26.64	0.0854
0.58	41.23	14.52	1.38	84.00	0.0900
0.56	40.42	16.75	1.26	27.16	0.0800
0.52	37.52	15.66	1.20	40.53	0.0774
0.48	34.38	12.81	1.23	19.68	0.0817
0.47	33.87	10.90	1.32	52.02	0.0895
0.53	38.28	15.12	1.25	45.36	0.0812
0.46	32.47	11.10	1.26	13.90	0.0865
0.62	43.62	16.70	1.39	14.56	0.0898
0.56	40.47	16.85	1.26	61.02	0.0800
0.47	33.43	13.60	1.17	31.68	0.0771
0.58	41.49	19.10	1.22	45.21	0.0753
0.52	37.56	12.96	1.33	24.51	0.0884
0.58	41.23	16.75	1.29	17.40	0.0818
0.58	41.78	14.49	1.41	23.50	0.0927
0.65	45.08	22.67	1.25	81.49	0.0769
0.57	40.89	13.31	1.43	36.48	0.0940
0.57	41.00	16.15	1.30	44.70	0.0832
0.59	41.82	17.88	1.27	40.53	0.0814
0.44	30.12	11.33	1.19	8.30	0.0803
0.62	43.95	15.70	1.45	75.30	0.0940
0.57	40.76	16.35	1.29	23.66	0.0825
0.68	46.61	19.59	1.41	32.00	0.0881
0.54	39.10	14.10	1.32	71.76	0.0863
0.61	43.11	17.77	1.32	78.08	0.0837
0.61	42.97	16.37	1.37	69.75	0.0881
0.64	44.63	19.05	1.34	102.86	0.0836
0.65	45.30	16.96	1.45	17.64	0.0943
0.61	43.47	15.58	1.43	54.12	0.0923
0.58	41.78	16.43	1.32	51.20	0.0842
0.51	36.81	14.27	1.24	12.16	0.0815
0.59	41.81	17.94	1.27	78.40	0.0800
0.58	41.33	15.28	1.35	69.44	0.0870
0.50	36.00	13.92	1.23	18.04	0.0799
0.58	41.56	15.04	1.37	63.36	0.0886
0.58	41.75	15.94	1.34	19.58	0.0875
0.44	30.49	10.85	1.22	12.87	0.0832
0.56	40.32	16.28	1.27	51.00	0.0809
0.59	42.28	15.65	1.38	26.88	0.0891
0.55	39.81	15.09	1.31	94.64	0.0844
0.49	35.47	13.67	1.22	28.22	0.0805
0.46	32.29	14.02	1.12	10.56	0.0733
0.52	37.56	14.50	1.25	22.42	0.0820
0.38	23.33	9.61	1.12	1.32	0.0779
0.60	42.67	17.78	1.31	28.48	0.0823
0.62	43.91	19.10	1.31	24.36	0.0827
0.64	44.89	18.22	1.38	37.44	0.0878
0.59	42.00	15.50	1.37	51.26	0.0899
0.57	41.00	18.59	1.22	37.62	0.0770

0.53	38.31	17.00	1.18	22.20	0.0753
0.44	30.93	10.54	1.25	8.91	0.0862
0.53	38.44	13.69	1.32	26.64	0.0866
0.57	40.83	17.87	1.23	75.69	0.0775
0.62	43.91	18.07	1.34	16.52	0.0858
0.54	39.29	15.67	1.26	56.70	0.0808
0.62	43.69	21.56	1.22	28.71	0.0749
0.63	44.17	18.35	1.35	49.35	0.0846
0.49	34.97	11.23	1.33	20.80	0.0899
0.57	40.62	17.25	1.25	65.67	0.0782
0.50	36.00	12.21	1.31	54.78	0.0872
0.52	37.86	15.42	1.23	40.60	0.0780
0.46	32.08	12.70	1.17	17.28	0.0771
0.61	43.08	17.19	1.34	78.28	0.0845
0.59	42.21	15.74	1.37	23.20	0.0884
0.54	38.96	17.19	1.20	71.99	0.0758
0.53	38.11	16.08	1.21	19.98	0.0780
0.53	38.12	14.38	1.28	31.32	0.0825
0.46	32.81	12.70	1.19	14.85	0.0796
0.69	46.95	21.31	1.37	40.70	0.0849
0.52	37.23	16.80	1.15	17.48	0.0730
0.56	40.32	15.50	1.31	101.10	0.0836
0.59	42.28	15.42	1.39	32.20	0.0900
0.57	40.70	17.81	1.23	85.75	0.0780
0.69	46.87	19.26	1.44	78.30	0.0893
0.57	40.72	14.99	1.34	35.65	0.0865
0.64	44.91	18.47	1.37	72.76	0.0868
0.71	47.76	19.26	1.48	62.92	0.0928
0.55	39.64	17.42	1.21	31.35	0.0774
0.57	40.88	16.74	1.28	22.80	0.0819
0.56	40.39	16.71	1.26	53.28	0.0807
0.55	39.81	15.38	1.29	33.28	0.0834
0.78	50.26	24.95	1.43	101.00	0.0861
0.63	44.22	17.95	1.36	58.56	0.0865
0.47	33.14	11.26	1.28	11.52	0.0868
0.61	43.00	17.71	1.32	76.80	0.0835
0.46	32.62	12.87	1.18	13.65	0.0781
0.55	39.49	15.25	1.29	33.60	0.0826
0.53	38.00	14.24	1.28	14.30	0.0836
0.52	37.84	17.06	1.16	41.76	0.0744
0.48	34.33	12.44	1.25	12.88	0.0835
0.42	28.06	11.62	1.12	5.55	0.0759
0.52	37.25	12.62	1.33	26.40	0.0885
0.61	43.13	16.76	1.36	51.91	0.0875
0.40	26.00	9.93	1.16	1.98	0.0809
0.49	34.95	14.22	1.19	12.06	0.0771
0.49	35.19	14.52	1.18	20.80	0.0769
0.59	41.81	18.57	1.25	56.00	0.0782
0.52	37.67	17.88	1.13	49.84	0.0723
0.59	41.88	17.06	1.30	42.93	0.0832
0.58	41.74	15.93	1.34	33.12	0.0852

0.52	37.82	15.39	1.22	17.29	0.0794
0.61	43.47	18.88	1.30	35.38	0.0818
0.67	46.20	19.71	1.39	57.19	0.0859
0.51	36.50	15.21	1.19	31.02	0.0764
0.48	34.57	12.14	1.27	8.16	0.0859
0.51	36.68	12.18	1.34	12.60	0.0905
0.62	43.53	16.27	1.40	39.90	0.0896
0.63	44.04	18.26	1.34	53.04	0.0846
0.57	40.65	17.37	1.25	17.64	0.0787
0.52	37.25	12.97	1.31	25.96	0.0869
0.56	40.34	15.73	1.30	35.50	0.0837
0.58	41.35	17.38	1.27	27.44	0.0805
0.46	32.18	13.60	1.14	19.60	0.0749
0.59	42.30	15.58	1.38	42.84	0.0885
0.51	36.90	14.49	1.23	21.00	0.0802
0.58	41.24	15.42	1.34	167.44	0.0872
0.53	38.09	15.24	1.24	72.24	0.0791
0.55	39.61	16.27	1.25	34.75	0.0799
0.53	38.59	15.23	1.26	36.45	0.0805
0.55	39.61	15.68	1.27	20.75	0.0819
0.52	37.50	15.06	1.23	37.18	0.0793
0.61	43.27	18.42	1.31	96.60	0.0825
0.53	37.98	12.59	1.36	80.73	0.0904
0.58	41.35	19.65	1.19	45.92	0.0742
0.55	39.85	13.56	1.38	22.80	0.0919
0.53	38.00	14.52	1.27	47.74	0.0825
0.50	36.00	14.81	1.19	22.95	0.0775
0.54	38.93	14.66	1.29	23.76	0.0840
0.54	39.25	18.57	1.16	13.42	0.0728
0.57	40.71	15.70	1.31	100.71	0.0844
0.59	42.11	20.30	1.20	43.84	0.0739
0.54	39.22	15.63	1.26	38.28	0.0804
0.57	40.67	13.68	1.40	27.20	0.0916
0.59	42.04	16.44	1.33	398.62	0.0840
0.55	39.95	15.84	1.28	19.32	0.0818
0.57	41.05	15.96	1.31	30.71	0.0830
0.50	36.25	12.46	1.31	182.85	0.0865
0.41	26.95	10.11	1.18	5.15	0.0811
0.58	41.44	18.32	1.24	96.00	0.0774
0.54	38.67	17.13	1.19	23.80	0.0762
0.55	39.53	14.77	1.31	42.12	0.0847
0.48	34.33	14.07	1.17	30.80	0.0770
0.57	41.00	15.81	1.32	94.18	0.0838
0.45	31.36	13.96	1.10	8.25	0.0719
0.56	40.00	15.64	1.29	46.44	0.0828
0.52	37.90	14.37	1.27	49.14	0.0830
0.59	42.11	13.18	1.49	25.90	0.0977
0.54	39.13	17.14	1.20	41.14	0.0765
0.50	36.00	14.22	1.22	53.60	0.0791
0.61	43.29	16.93	1.36	66.88	0.0860
0.55	39.49	15.41	1.28	25.25	0.0825

0.44	30.55	12.00	1.17	7.20	0.0785
0.58	41.24	14.14	1.40	35.36	0.0924
0.61	43.05	19.88	1.25	33.60	0.0777
0.59	41.81	16.37	1.33	26.88	0.0851
0.58	41.29	15.97	1.32	27.84	0.0848
0.62	43.60	16.76	1.38	59.28	0.0876
0.59	42.02	13.26	1.48	29.70	0.0983
0.58	41.64	16.62	1.31	91.80	0.0825
0.56	40.24	13.47	1.40	31.05	0.0921
0.56	40.40	15.05	1.33	55.44	0.0850
0.55	39.90	14.19	1.35	24.96	0.0886
0.59	41.98	19.77	1.21	36.25	0.0753
0.58	41.52	14.73	1.39	40.31	0.0902
0.61	43.31	19.08	1.29	36.40	0.0799
0.52	37.52	11.96	1.38	57.12	0.0927
0.62	43.92	18.07	1.35	61.18	0.0842
0.57	40.67	17.41	1.24	88.96	0.0780
0.48	34.26	13.70	1.19	14.52	0.0788
0.49	35.04	13.65	1.21	15.30	0.0800
0.60	42.44	17.72	1.30	69.44	0.0819
0.58	41.68	17.55	1.28	58.09	0.0796
0.43	29.13	16.00	0.98	13.38	0.0628
0.44	30.29	14.07	1.07	6.60	0.0707
0.60	42.52	19.17	1.25	22.44	0.0776
0.47	33.26	12.64	1.21	25.20	0.0801
0.66	45.90	17.99	1.44	35.88	0.0908
0.48	34.40	13.96	1.18	51.51	0.0770
0.50	36.28	11.97	1.34	20.58	0.0906
0.55	39.46	12.96	1.39	31.05	0.0930
0.58	41.48	17.38	1.27	43.42	0.0811
0.48	34.57	12.96	1.23	21.72	0.0822
0.64	44.75	19.56	1.33	89.68	0.0824
0.55	39.54	15.91	1.26	23.31	0.0816
0.52	37.48	13.96	1.27	51.06	0.0832
0.56	40.37	16.38	1.27	70.18	0.0809
0.58	41.24	17.35	1.27	63.44	0.0806
0.56	40.52	18.44	1.20	50.44	0.0756
0.59	42.18	18.19	1.27	28.52	0.0799
0.58	41.24	16.07	1.32	48.88	0.0848
0.61	43.08	18.13	1.31	84.36	0.0816
0.58	41.58	16.04	1.33	194.32	0.0856
0.49	35.07	12.72	1.26	15.47	0.0835
0.49	34.96	12.17	1.28	14.82	0.0853
0.56	40.47	15.97	1.29	105.03	0.0829
0.57	40.83	16.75	1.27	53.65	0.0809
0.56	40.60	16.43	1.28	28.42	0.0813
0.62	43.67	18.62	1.32	39.20	0.0834
0.64	44.91	19.12	1.35	73.78	0.0848
0.64	44.99	17.72	1.41	137.35	0.0883
0.51	37.03	14.24	1.25	21.20	0.0815
0.60	42.81	17.37	1.33	29.04	0.0839

0.56	40.29	18.07	1.21	22.10	0.0760
0.66	45.80	19.75	1.37	118.49	0.0847
0.50	36.00	12.24	1.31	24.48	0.0878
0.56	40.42	17.59	1.23	25.48	0.0774
0.55	39.65	13.56	1.37	62.91	0.0900
0.50	35.71	14.98	1.18	15.12	0.0771
0.52	37.65	14.95	1.24	23.10	0.0802
0.45	31.52	11.34	1.23	12.69	0.0834
0.43	29.85	11.85	1.15	4.83	0.0779
0.58	41.52	16.89	1.29	18.85	0.0824
0.66	45.55	17.46	1.44	51.30	0.0929
0.54	38.72	14.81	1.28	23.00	0.0830
0.59	42.04	15.70	1.36	24.70	0.0866
0.63	44.22	20.18	1.29	33.92	0.0800
0.56	40.20	16.40	1.27	26.22	0.0813
0.59	41.84	16.80	1.31	50.84	0.0832
0.52	37.90	15.69	1.22	22.89	0.0782
0.56	40.19	14.24	1.36	40.04	0.0885
0.54	38.72	14.81	1.28	19.32	0.0830
0.49	34.86	14.07	1.19	39.00	0.0785
0.63	44.00	16.80	1.40	96.20	0.0888
0.56	40.00	14.67	1.33	19.36	0.0873
0.61	43.45	17.31	1.35	74.16	0.0855
0.62	43.66	18.51	1.32	97.56	0.0824
0.59	42.00	17.31	1.30	18.88	0.0819
0.56	40.44	16.60	1.27	27.20	0.0799
0.65	45.38	18.89	1.38	29.26	0.0863
0.54	38.70	11.95	1.42	16.00	0.0972
0.38	23.14	10.80	1.06	-2.46	0.0726
0.48	34.36	14.24	1.17	19.35	0.0762
0.59	42.05	15.42	1.38	18.48	0.0893
0.48	33.97	13.61	1.18	14.85	0.0785
0.65	45.23	19.53	1.35	33.12	0.0829
0.54	39.00	15.12	1.28	36.52	0.0828
0.57	40.65	17.09	1.26	122.92	0.0795
0.58	41.43	14.58	1.39	23.23	0.0917
0.59	42.09	17.12	1.31	78.20	0.0824
0.58	41.45	15.66	1.34	25.80	0.0862
0.64	44.94	19.60	1.33	40.68	0.0831
0.61	43.23	18.07	1.32	37.44	0.0825
0.54	38.82	13.18	1.36	24.03	0.0893
0.59	42.17	17.41	1.30	100.80	0.0814
0.54	39.10	16.60	1.22	33.02	0.0774
0.49	34.93	16.15	1.11	17.00	0.0709
0.65	45.17	20.05	1.33	78.66	0.0823
0.41	26.97	11.39	1.11	2.08	0.0752
0.48	34.55	13.34	1.21	10.12	0.0808
0.65	45.30	20.07	1.33	46.20	0.0821
0.59	42.21	15.74	1.37	27.84	0.0884
0.47	33.14	12.30	1.22	9.48	0.0819
0.41	26.65	12.56	1.05	5.96	0.0699

0.53	38.20	14.50	1.27	22.56	0.0827
0.54	38.80	16.18	1.23	112.56	0.0783
0.56	40.42	16.47	1.27	37.52	0.0809
0.59	42.15	16.70	1.33	25.74	0.0842
0.52	37.25	16.65	1.16	27.28	0.0736
0.54	38.86	15.73	1.25	23.37	0.0808
0.46	32.57	11.96	1.22	14.88	0.0821
0.60	42.67	18.00	1.30	34.32	0.0826
0.59	41.98	16.97	1.31	60.61	0.0834
0.59	42.00	14.31	1.43	16.94	0.0948
0.54	38.72	13.22	1.35	25.30	0.0896
0.56	40.49	15.57	1.31	40.61	0.0837
0.48	34.63	11.47	1.31	16.65	0.0887
0.61	43.07	15.42	1.42	69.88	0.0918
0.35	19.27	9.93	1.03	-2.67	0.0709
0.56	40.04	12.82	1.43	43.71	0.0939
0.54	38.82	14.20	1.31	50.49	0.0850
0.45	31.94	11.82	1.21	14.74	0.0816
0.52	37.23	14.47	1.24	34.96	0.0806
0.58	41.35	16.63	1.30	57.96	0.0829
0.51	37.15	16.28	1.17	28.20	0.0748
0.57	41.18	15.42	1.34	32.40	0.0868
0.53	38.05	12.88	1.35	73.80	0.0880
0.55	39.37	15.66	1.27	37.00	0.0813
0.59	42.14	19.20	1.24	114.60	0.0771
0.61	43.42	21.30	1.22	52.26	0.0740
0.49	35.15	11.81	1.31	10.40	0.0888
0.55	39.33	14.79	1.30	52.78	0.0843
0.62	43.53	18.59	1.31	52.50	0.0819
0.48	34.08	14.80	1.14	33.75	0.0737
0.58	41.62	17.59	1.27	59.21	0.0801
0.57	40.82	17.03	1.26	23.00	0.0806
0.57	41.00	16.40	1.29	32.12	0.0837
0.63	44.00	18.75	1.32	152.64	0.0833
0.64	44.94	17.03	1.43	79.20	0.0913
0.49	35.17	11.96	1.30	13.58	0.0878
0.57	41.12	16.53	1.29	38.36	0.0826
0.50	35.75	13.18	1.26	27.72	0.0823
0.58	41.23	14.52	1.38	48.90	0.0900
0.60	42.74	14.48	1.45	75.52	0.0951
0.53	38.50	14.52	1.28	27.06	0.0838
0.46	32.52	12.74	1.18	13.64	0.0788
0.59	41.98	16.97	1.31	52.49	0.0834
0.61	43.47	19.68	1.27	27.72	0.0790
0.41	27.29	11.04	1.13	4.24	0.0773
0.57	40.88	16.27	1.30	39.48	0.0828
0.53	38.47	15.18	1.25	32.43	0.0811
0.62	43.61	18.44	1.32	90.44	0.0828
0.43	29.75	11.29	1.18	7.92	0.0805
0.53	38.14	13.19	1.33	37.18	0.0876
0.41	27.69	10.85	1.15	6.65	0.0783

0.51	36.77	14.24	1.24	28.20	0.0808
0.54	38.82	14.76	1.28	18.80	0.0840
0.52	37.33	14.76	1.24	22.27	0.0808
0.59	41.98	18.51	1.25	57.71	0.0787
0.55	39.56	12.33	1.43	28.65	0.0981
0.57	40.70	17.49	1.24	34.75	0.0789
0.58	41.70	17.25	1.29	53.20	0.0808
0.57	40.88	17.41	1.25	28.08	0.0798
0.51	36.53	13.88	1.25	152.49	0.0822
0.59	41.91	16.00	1.35	78.60	0.0864
0.51	37.04	14.52	1.24	35.53	0.0806
0.63	44.17	16.87	1.40	55.30	0.0895
0.64	44.75	18.67	1.36	24.32	0.0850
0.55	39.86	13.63	1.38	93.50	0.0907
0.57	40.65	17.68	1.23	42.28	0.0777
0.60	42.82	16.64	1.36	27.30	0.0868
0.61	42.96	18.94	1.28	88.40	0.0795
0.55	39.84	15.16	1.30	140.56	0.0839
0.57	40.66	15.24	1.33	73.50	0.0855
0.52	37.79	10.29	1.50	12.80	0.1035
0.49	35.22	13.57	1.22	25.65	0.0800
0.47	33.65	13.73	1.17	23.90	0.0775
0.63	44.41	20.85	1.27	39.60	0.0791
0.55	39.33	15.73	1.26	27.60	0.0819
0.46	32.76	12.31	1.21	11.30	0.0814
0.53	38.25	14.23	1.29	23.54	0.0842
0.61	42.96	16.80	1.35	71.06	0.0860
0.58	41.73	16.92	1.30	49.29	0.0825
0.53	38.35	14.84	1.27	36.45	0.0813
0.62	43.62	15.71	1.43	57.53	0.0924
0.56	40.05	15.97	1.28	23.14	0.0819
0.51	36.77	14.52	1.23	17.20	0.0798
0.55	39.41	16.22	1.25	44.28	0.0792
0.64	44.69	16.23	1.45	62.32	0.0926
0.50	35.86	12.56	1.29	10.95	0.0865
0.50	36.27	14.76	1.20	17.55	0.0786
0.57	40.65	18.22	1.22	42.28	0.0762
0.58	41.45	16.09	1.32	34.80	0.0847
0.49	35.46	12.44	1.28	24.64	0.0859
0.48	34.31	12.96	1.22	19.50	0.0815
0.46	32.29	12.70	1.18	8.40	0.0783
0.45	31.71	11.55	1.22	16.56	0.0822
0.42	28.46	13.12	1.07	3.51	0.0711
0.58	41.52	17.19	1.28	61.77	0.0814
0.62	43.79	16.75	1.39	76.22	0.0884
0.57	41.06	13.78	1.41	27.00	0.0936
0.51	36.99	13.08	1.30	54.28	0.0856
0.58	41.25	15.64	1.34	15.62	0.0871
0.58	41.24	15.74	1.33	55.90	0.0860
0.66	45.70	17.87	1.43	79.55	0.0900
0.63	44.35	19.42	1.32	41.25	0.0823

0.46	32.52	12.44	1.20	8.47	0.0801
0.62	43.96	16.02	1.43	116.00	0.0911
0.49	34.89	15.42	1.14	12.88	0.0735
0.62	43.78	14.43	1.50	257.92	0.0984
0.53	38.47	15.43	1.24	51.06	0.0802
0.44	30.53	11.85	1.17	7.74	0.0795
0.62	43.91	16.74	1.40	25.76	0.0903
0.61	43.45	16.75	1.38	40.32	0.0874
0.61	43.45	17.03	1.37	74.88	0.0865
0.54	39.18	15.91	1.25	39.96	0.0797
0.58	41.35	15.72	1.34	21.84	0.0861
0.48	34.29	14.46	1.16	14.88	0.0758
0.71	47.78	21.89	1.39	106.21	0.0854
0.58	41.54	18.47	1.24	34.25	0.0783
0.43	29.53	10.65	1.21	12.80	0.0824
0.45	31.15	12.80	1.14	11.36	0.0763
0.59	41.91	16.59	1.32	49.80	0.0843
0.51	36.89	13.86	1.26	16.80	0.0824
0.53	38.47	17.94	1.15	45.54	0.0725
0.64	44.57	17.27	1.40	31.35	0.0897
0.57	40.65	15.38	1.32	42.84	0.0853
0.43	29.33	12.05	1.13	8.64	0.0759
0.59	42.09	16.59	1.33	95.54	0.0842
0.52	37.50	12.59	1.34	32.12	0.0893
0.47	33.65	12.06	1.25	24.60	0.0845
0.58	41.52	16.00	1.33	26.10	0.0854
0.62	43.50	17.91	1.33	60.04	0.0835
0.53	38.03	14.52	1.27	83.16	0.0827
0.58	41.68	14.89	1.39	38.87	0.0911
0.54	38.68	15.64	1.24	39.36	0.0799
0.63	44.40	19.47	1.32	104.16	0.0810
0.46	32.11	11.66	1.22	7.84	0.0821
0.55	39.61	15.39	1.29	43.00	0.0829
0.55	39.81	15.86	1.27	66.04	0.0816
0.64	44.72	22.04	1.25	30.60	0.0763
0.58	41.29	17.08	1.28	13.63	0.0811
0.48	34.36	13.38	1.20	28.80	0.0795
0.57	41.00	16.84	1.28	22.20	0.0809
0.43	29.75	12.03	1.14	7.08	0.0772
0.49	35.44	13.40	1.24	18.46	0.0823
0.64	44.70	19.09	1.34	47.26	0.0842
0.57	41.12	18.52	1.22	40.32	0.0766
0.55	39.68	17.12	1.22	50.76	0.0786
0.55	39.72	16.73	1.24	146.16	0.0783
0.54	39.10	13.70	1.34	51.22	0.0880
0.44	30.41	13.70	1.09	7.00	0.0712
0.55	39.41	14.31	1.33	53.04	0.0867
0.43	29.11	11.46	1.16	2.31	0.0791
0.59	41.88	16.40	1.33	47.25	0.0854
0.61	42.98	13.62	1.51	44.80	0.1002
0.69	47.00	19.35	1.44	60.06	0.0899

0.48	34.61	15.42	1.13	17.92	0.0730
0.60	42.43	17.48	1.31	24.96	0.0835
0.40	25.67	10.89	1.10	3.22	0.0755
0.56	40.05	15.10	1.31	60.84	0.0850
0.64	44.82	16.40	1.45	94.85	0.0934
0.39	25.35	11.63	1.06	6.68	0.0714
0.51	36.58	14.11	1.24	11.99	0.0825
0.57	41.06	15.95	1.31	70.76	0.0843
0.62	43.79	19.82	1.28	69.19	0.0790
0.59	41.90	16.58	1.32	55.20	0.0860
0.52	37.73	14.50	1.26	47.84	0.0817
0.51	36.76	13.83	1.26	61.95	0.0822
0.60	42.74	18.99	1.27	31.62	0.0787
0.52	37.67	12.94	1.33	27.82	0.0874
0.60	42.67	17.19	1.33	78.05	0.0837
0.46	32.62	12.05	1.22	16.12	0.0816
0.50	35.62	12.32	1.29	19.74	0.0859
0.60	42.59	19.05	1.26	50.70	0.0787
0.61	43.13	16.76	1.36	73.37	0.0875
0.47	33.78	14.24	1.15	13.30	0.0752
0.59	41.96	15.14	1.39	51.84	0.0888
0.54	39.00	16.04	1.24	13.86	0.0796
0.54	38.89	16.43	1.22	39.75	0.0776
0.66	45.80	18.51	1.41	48.40	0.0887
0.60	42.89	18.89	1.27	40.96	0.0797
0.61	43.14	14.74	1.45	31.60	0.0935
0.45	31.22	12.74	1.15	6.12	0.0765
0.75	49.33	21.77	1.47	94.00	0.0905
0.57	40.61	14.03	1.38	62.31	0.0901
0.50	36.00	11.50	1.35	31.54	0.0913
0.51	36.76	15.66	1.18	48.51	0.0757
0.57	41.18	15.42	1.34	17.28	0.0868
0.60	42.59	17.00	1.33	38.70	0.0849
0.63	44.37	15.84	1.46	58.40	0.0932
0.60	42.43	15.34	1.40	31.20	0.0911
0.49	35.44	12.53	1.28	19.18	0.0858
0.46	32.86	13.07	1.18	39.36	0.0780
0.41	27.38	11.41	1.12	10.36	0.0752
0.59	41.93	14.84	1.40	29.90	0.0921
0.57	41.12	16.76	1.29	75.12	0.0825
0.62	43.56	17.03	1.37	30.72	0.0874
0.57	40.72	15.50	1.32	34.41	0.0846
0.58	41.52	18.37	1.24	52.49	0.0779
0.48	34.06	13.94	1.17	13.44	0.0769
0.50	35.72	14.76	1.19	23.94	0.0776
0.50	36.00	14.52	1.20	41.22	0.0783
0.48	33.89	15.38	1.11	25.02	0.0710
0.48	34.14	13.77	1.18	9.84	0.0781
0.59	42.36	16.97	1.32	60.30	0.0844
0.49	35.44	13.17	1.25	23.94	0.0830
0.57	41.00	16.13	1.31	33.60	0.0836

0.61	43.23	17.25	1.35	56.88	0.0851
0.47	33.87	11.66	1.28	27.54	0.0855
0.59	42.22	18.51	1.26	139.20	0.0789
0.61	42.97	17.00	1.35	46.50	0.0859
0.57	41.17	16.65	1.29	39.37	0.0819
0.56	40.46	15.14	1.33	14.35	0.0870
0.56	40.10	13.30	1.40	43.50	0.0928
0.59	42.19	16.07	1.35	68.12	0.0876
0.61	43.27	16.74	1.37	31.50	0.0879
0.53	38.08	12.85	1.35	17.29	0.0902
0.61	43.11	16.90	1.36	31.36	0.0865
0.50	35.66	11.10	1.36	63.18	0.0928
0.57	40.71	16.74	1.27	44.28	0.0809
0.54	39.17	14.52	1.31	45.60	0.0852
0.58	41.58	15.58	1.35	34.16	0.0873
0.59	41.95	17.44	1.29	59.02	0.0823
0.56	40.34	15.73	1.30	26.00	0.0837
0.51	36.80	16.06	1.17	72.42	0.0752
0.48	34.00	12.75	1.22	11.88	0.0818
0.57	40.76	14.81	1.35	21.06	0.0882
0.58	41.23	16.89	1.28	82.20	0.0814
0.50	36.00	13.43	1.25	45.60	0.0821
0.59	42.12	17.75	1.29	66.69	0.0816
0.57	40.76	17.89	1.23	45.24	0.0777
0.52	37.35	15.05	1.22	33.12	0.0800
0.54	39.16	14.58	1.30	13.32	0.0860
0.51	36.55	15.74	1.17	11.70	0.0759
0.62	43.53	17.72	1.34	36.40	0.0846
0.57	40.83	18.01	1.23	63.51	0.0771
0.62	43.71	15.31	1.45	49.78	0.0934
0.52	37.54	15.11	1.23	26.40	0.0795
0.62	43.96	18.86	1.32	71.20	0.0817
0.59	42.29	18.81	1.25	57.35	0.0784
0.62	43.91	17.83	1.35	40.59	0.0857
0.58	41.52	16.44	1.31	108.17	0.0838
0.62	43.53	15.77	1.42	39.55	0.0914
0.73	48.43	23.15	1.38	62.54	0.0832
0.54	38.97	13.61	1.34	33.76	0.0899
0.56	40.43	16.96	1.25	150.60	0.0791
0.52	37.25	13.70	1.28	36.30	0.0838
0.62	43.78	16.73	1.39	25.92	0.0892
0.52	37.79	14.84	1.25	75.80	0.0811
0.64	44.56	18.50	1.36	25.28	0.0858
0.60	42.60	15.29	1.40	83.07	0.0897
0.58	41.56	17.72	1.27	37.76	0.0794
0.55	39.37	16.23	1.24	62.00	0.0794
0.55	39.86	15.56	1.29	18.25	0.0830
0.60	42.59	17.09	1.33	98.34	0.0842
0.57	40.82	16.07	1.30	23.50	0.0838
0.53	38.44	14.79	1.27	62.88	0.0823
0.44	30.55	10.52	1.24	5.12	0.0857

0.53	38.53	13.05	1.36	36.83	0.0888
0.56	40.48	16.81	1.26	227.94	0.0798
0.48	34.70	10.70	1.36	19.38	0.0923
0.61	43.33	18.10	1.32	45.76	0.0833
0.62	43.50	19.32	1.28	32.70	0.0805
0.57	41.00	17.00	1.27	38.22	0.0811
0.62	43.74	18.67	1.32	39.55	0.0824
0.47	33.16	12.18	1.23	13.92	0.0817
0.57	40.88	17.41	1.25	42.48	0.0798
0.56	40.38	15.92	1.29	64.48	0.0822
0.52	37.77	14.52	1.26	28.35	0.0821
0.59	42.00	17.31	1.30	63.04	0.0819
0.62	43.50	17.10	1.37	120.00	0.0874
0.58	41.26	16.70	1.29	33.67	0.0811
0.56	40.52	17.68	1.23	26.26	0.0777
0.45	31.14	15.25	1.05	11.88	0.0673
0.57	40.94	16.32	1.30	26.73	0.0830
0.56	40.09	13.69	1.38	96.30	0.0902
0.50	36.39	14.37	1.22	12.96	0.0798
0.54	38.68	12.68	1.38	36.24	0.0919
0.61	43.03	18.37	1.30	53.13	0.0814
0.56	40.04	18.31	1.19	50.22	0.0740
0.65	45.05	18.26	1.39	176.12	0.0874
0.52	37.54	13.63	1.29	23.60	0.0852
0.49	35.44	15.41	1.15	13.65	0.0749
0.63	44.09	17.30	1.38	114.48	0.0875
0.55	39.76	14.24	1.34	59.13	0.0874
0.55	39.81	13.67	1.37	31.98	0.0901
0.53	38.17	15.61	1.23	39.25	0.0785
0.60	42.88	21.90	1.18	39.55	0.0718
0.57	40.94	16.02	1.31	54.27	0.0840
0.52	37.77	14.81	1.25	35.07	0.0810
0.47	33.33	11.72	1.26	24.31	0.0840
0.52	37.20	16.21	1.17	35.50	0.0743
0.55	39.53	15.31	1.29	102.06	0.0827
0.64	44.65	19.36	1.33	39.14	0.0826
0.49	35.34	14.51	1.18	18.90	0.0769
0.54	39.13	13.68	1.35	251.00	0.0884
0.56	40.19	16.98	1.24	54.60	0.0787
0.51	36.51	14.79	1.21	24.00	0.0782
0.52	37.48	14.33	1.26	36.34	0.0817
0.61	43.13	17.10	1.35	44.95	0.0864
0.53	38.56	16.07	1.22	33.00	0.0788
0.55	39.49	16.28	1.25	26.04	0.0791
0.42	28.00	12.12	1.10	2.12	0.0743
0.49	35.20	13.96	1.20	20.23	0.0788
0.51	36.76	13.70	1.26	25.41	0.0828
0.59	42.35	18.81	1.26	48.33	0.0792
0.51	37.14	15.35	1.21	42.84	0.0776
0.61	42.98	19.56	1.26	84.00	0.0787
0.55	39.54	17.41	1.21	49.14	0.0768

0.64	44.53	16.22	1.45	56.98	0.0927
0.55	39.45	16.67	1.23	57.13	0.0776
0.56	40.47	17.28	1.24	84.51	0.0786
0.52	37.71	14.45	1.26	16.92	0.0827
0.53	38.00	13.30	1.32	55.88	0.0875
0.57	40.84	17.82	1.24	111.21	0.0771
0.56	40.19	15.61	1.30	52.36	0.0832
0.58	41.62	18.15	1.25	52.08	0.0785
0.46	32.18	12.15	1.20	10.20	0.0808
0.57	40.82	16.52	1.28	59.00	0.0823
0.39	24.52	11.24	1.06	4.32	0.0718
0.50	36.00	14.36	1.21	10.40	0.0786
0.58	41.37	17.46	1.27	18.24	0.0809
0.57	40.77	17.80	1.23	210.00	0.0774
0.61	43.14	17.50	1.33	44.98	0.0856
0.51	37.04	14.81	1.22	28.31	0.0795
0.60	42.92	15.25	1.42	37.62	0.0918
0.54	38.78	18.57	1.14	17.64	0.0719
0.59	41.81	17.94	1.27	35.56	0.0800
0.48	34.68	12.40	1.26	22.86	0.0838
0.54	38.93	15.38	1.26	72.24	0.0814
0.65	45.26	19.60	1.35	50.32	0.0840
0.60	42.59	17.31	1.32	28.20	0.0839
0.54	38.77	16.38	1.22	27.00	0.0775
0.57	41.06	16.66	1.29	50.17	0.0818
0.52	37.37	13.84	1.28	11.25	0.0848
0.58	41.52	15.56	1.35	44.95	0.0870
0.57	40.88	17.14	1.26	19.32	0.0800
0.55	39.91	17.05	1.23	30.16	0.0776
0.61	43.23	17.80	1.33	28.80	0.0833
0.55	39.47	17.31	1.21	39.61	0.0776
0.54	39.16	16.03	1.24	12.96	0.0807
0.60	42.52	16.98	1.33	30.94	0.0842
0.62	43.74	20.12	1.27	82.60	0.0783
0.58	41.23	17.03	1.28	24.60	0.0809
0.43	29.04	12.00	1.13	12.98	0.0750
0.57	41.12	17.72	1.25	33.32	0.0788
0.55	39.59	14.32	1.33	12.40	0.0879
0.53	37.93	14.96	1.25	23.75	0.0802
0.43	29.94	11.39	1.18	3.76	0.0800
0.66	45.89	20.35	1.35	85.84	0.0840
0.48	34.52	12.86	1.23	62.56	0.0805
0.46	32.76	13.69	1.15	15.30	0.0758
0.56	40.52	17.84	1.22	42.64	0.0773
0.68	46.61	18.08	1.47	102.00	0.0930
0.53	38.33	12.98	1.35	195.44	0.0887
0.56	40.50	17.64	1.23	13.42	0.0784
0.50	35.60	13.43	1.24	34.51	0.0817
0.62	43.50	17.10	1.37	51.60	0.0874
0.49	35.49	13.18	1.25	16.60	0.0820
0.41	27.03	12.22	1.07	0.00	0.0726

0.53	37.90	13.82	1.30	23.92	0.0843
0.51	36.77	14.24	1.24	19.40	0.0808
0.59	41.98	15.73	1.36	70.47	0.0877
0.43	30.00	11.50	1.18	15.12	0.0788
0.61	42.98	16.76	1.36	37.52	0.0872
0.63	44.06	14.92	1.49	97.20	0.0959
0.58	41.41	20.15	1.18	58.32	0.0732
0.59	42.36	17.27	1.31	90.00	0.0833
0.55	39.75	14.79	1.32	23.98	0.0861
0.55	39.49	14.21	1.33	18.76	0.0866
0.55	39.86	17.78	1.20	40.75	0.0759
0.55	39.57	14.52	1.32	56.94	0.0859
0.58	41.56	17.19	1.28	39.36	0.0810
0.60	42.47	17.31	1.32	29.70	0.0840
0.55	39.65	16.00	1.26	39.69	0.0806
0.60	42.52	17.25	1.32	65.62	0.0833
0.48	34.31	12.65	1.24	15.99	0.0828
0.60	42.67	15.06	1.42	25.20	0.0914
0.52	37.79	18.44	1.12	23.20	0.0702
0.60	42.44	18.59	1.27	53.76	0.0793
0.57	40.94	13.17	1.44	45.88	0.0950
0.54	38.86	14.50	1.30	82.83	0.0830
0.51	36.77	13.68	1.26	14.60	0.0830
0.54	38.75	15.37	1.26	13.42	0.0813
0.51	37.11	12.54	1.33	48.07	0.0883
0.52	37.75	13.12	1.32	49.94	0.0875
0.51	36.53	16.00	1.16	13.86	0.0746
0.52	37.28	14.67	1.24	22.00	0.0805
0.58	41.37	16.41	1.31	35.52	0.0843
0.51	37.03	13.38	1.29	44.80	0.0849
0.51	37.05	13.57	1.28	34.38	0.0845
0.59	42.29	14.22	1.44	53.94	0.0945
0.52	37.79	15.36	1.23	13.60	0.0793
0.61	43.10	16.75	1.36	71.75	0.0865
0.60	42.52	16.32	1.36	51.77	0.0869
0.64	44.51	16.04	1.45	43.92	0.0935
0.62	43.67	20.48	1.25	49.00	0.0782
0.62	43.96	20.56	1.26	40.95	0.0778
0.56	40.30	15.18	1.32	47.88	0.0851
0.60	42.88	16.43	1.37	50.05	0.0870
0.59	42.07	17.72	1.28	100.44	0.0810
0.56	40.29	13.93	1.38	52.00	0.0904
0.60	42.73	16.86	1.34	54.80	0.0843
0.59	41.92	16.32	1.33	64.00	0.0837
0.51	36.80	14.95	1.21	19.04	0.0788
0.55	39.76	14.24	1.34	21.06	0.0874
0.54	38.72	14.81	1.28	36.80	0.0830
0.53	38.25	17.14	1.17	19.36	0.0744
0.61	42.96	15.32	1.42	50.16	0.0899
0.64	44.72	17.79	1.39	39.60	0.0880
0.47	33.68	14.14	1.15	9.24	0.0759

0.48	34.63	13.19	1.22	13.65	0.0809
0.51	36.78	13.80	1.26	34.77	0.0828
0.63	44.26	15.26	1.48	73.78	0.0962
0.59	41.81	17.00	1.30	74.48	0.0830
0.63	44.44	18.27	1.36	59.84	0.0862
0.62	43.95	18.01	1.35	78.30	0.0858
0.53	38.47	14.75	1.27	24.38	0.0826
0.61	43.45	18.73	1.30	34.92	0.0811
0.52	37.20	12.70	1.33	25.75	0.0875
0.60	42.67	17.38	1.32	23.78	0.0840
0.55	39.37	14.85	1.30	36.40	0.0838
0.56	40.60	14.65	1.35	71.63	0.0878
0.54	39.00	12.96	1.38	24.42	0.0918
0.64	44.57	17.78	1.38	73.26	0.0880
0.53	38.53	15.42	1.25	31.71	0.0807
0.54	39.25	16.37	1.23	38.50	0.0792
0.49	35.47	14.81	1.18	24.48	0.0763
0.52	37.20	13.90	1.27	43.00	0.0824
0.62	43.95	17.84	1.36	62.10	0.0863
0.51	36.40	16.80	1.13	46.58	0.0721
0.47	33.01	14.07	1.14	11.88	0.0752
0.57	40.83	15.36	1.33	91.93	0.0857
0.42	28.16	12.27	1.09	6.62	0.0735
0.49	34.79	11.93	1.29	10.48	0.0883
0.65	45.22	19.64	1.34	70.84	0.0828
0.69	47.18	21.72	1.37	62.04	0.0836
0.60	42.74	16.07	1.38	30.52	0.0890
0.50	36.26	14.10	1.23	31.20	0.0802
0.56	40.19	15.61	1.30	55.44	0.0832
0.61	42.97	16.21	1.38	30.07	0.0886
0.59	42.02	15.30	1.38	19.25	0.0902
0.57	40.63	17.38	1.24	18.96	0.0792
0.54	39.13	15.50	1.26	43.25	0.0813
0.48	34.31	14.50	1.16	9.49	0.0756
0.64	44.51	16.66	1.43	54.00	0.0912
0.59	42.33	17.14	1.32	89.20	0.0822
0.47	33.84	13.17	1.20	14.56	0.0790
0.61	43.27	18.36	1.31	31.16	0.0814
0.65	45.18	17.14	1.44	46.00	0.0912
0.61	43.25	19.35	1.27	47.85	0.0793
0.42	28.58	12.91	1.08	4.68	0.0719
0.52	37.65	15.01	1.23	50.22	0.0790
0.53	38.31	14.64	1.27	17.00	0.0832
0.54	39.29	15.54	1.27	22.41	0.0813
0.59	42.05	16.07	1.35	47.04	0.0869
0.55	39.41	16.44	1.24	48.96	0.0790
0.50	35.71	16.01	1.14	12.21	0.0740
0.53	38.44	14.24	1.29	64.32	0.0843
0.57	40.89	15.72	1.32	64.96	0.0841
0.61	43.47	15.14	1.45	33.35	0.0948
0.64	44.78	19.96	1.32	172.40	0.0811

0.48	34.24	13.97	1.18	12.50	0.0779
0.52	37.41	14.51	1.25	107.19	0.0802
0.44	30.85	12.55	1.15	21.68	0.0768
0.59	42.05	16.03	1.35	59.64	0.0870
0.52	37.79	15.66	1.21	22.00	0.0783
0.60	42.67	14.76	1.43	38.57	0.0937
0.50	36.00	13.73	1.24	12.80	0.0817
0.52	37.38	13.56	1.29	39.82	0.0847
0.56	40.57	16.35	1.28	49.45	0.0825
0.60	42.59	16.68	1.34	68.40	0.0860
0.54	39.17	13.97	1.33	32.40	0.0874
0.56	40.42	18.15	1.21	23.52	0.0758
0.56	40.14	15.28	1.31	69.31	0.0841
0.57	41.06	15.66	1.33	58.00	0.0853
0.52	37.82	14.17	1.28	14.63	0.0839
0.57	40.67	15.67	1.31	67.20	0.0837
0.54	38.89	16.15	1.23	31.75	0.0785
0.56	40.25	15.73	1.29	33.00	0.0840
0.41	27.38	13.91	1.01	0.00	0.0671
0.57	40.94	16.07	1.31	38.13	0.0832
0.46	32.67	12.99	1.17	9.84	0.0787
0.58	41.59	16.60	1.31	36.40	0.0826
0.52	37.32	14.48	1.25	9.72	0.0816
0.46	32.21	13.42	1.14	4.00	0.0760
0.55	39.70	14.95	1.31	35.19	0.0851
0.59	42.02	18.55	1.25	57.75	0.0793
0.56	40.42	17.03	1.25	36.68	0.0791
0.63	44.35	18.75	1.34	53.46	0.0842
0.51	37.13	17.12	1.14	6.50	0.0734
0.53	37.98	13.42	1.32	25.30	0.0867
0.59	42.05	14.14	1.44	56.28	0.0946
0.45	32.00	12.87	1.16	17.16	0.0770
0.62	43.82	21.32	1.23	21.75	0.0764
0.46	32.58	12.69	1.19	23.04	0.0779
0.61	43.25	16.75	1.37	65.67	0.0873
0.54	39.00	16.97	1.20	18.92	0.0767
0.60	42.52	17.52	1.31	44.88	0.0824
0.50	35.87	14.38	1.21	13.50	0.0786
0.65	45.25	17.95	1.41	306.25	0.0894
0.64	44.66	17.23	1.41	72.15	0.0892
0.46	32.71	11.92	1.23	21.00	0.0821
0.53	38.59	15.67	1.24	38.61	0.0790
0.55	39.50	15.42	1.28	34.54	0.0830
0.64	44.69	16.65	1.44	47.97	0.0910
0.59	42.30	17.59	1.30	76.16	0.0816
0.53	38.03	12.15	1.39	24.99	0.0932
0.48	34.40	13.64	1.19	13.94	0.0782
0.52	37.23	15.84	1.19	27.60	0.0759
0.46	32.57	11.96	1.22	16.44	0.0821
0.45	31.29	14.24	1.09	19.70	0.0710
0.58	41.33	16.59	1.30	61.44	0.0823

0.58	41.68	13.65	1.45	42.90	0.0953
0.66	45.58	18.64	1.40	58.90	0.0878
0.51	36.53	16.66	1.14	22.95	0.0728
0.60	42.67	17.71	1.31	58.00	0.0830
0.60	42.74	16.76	1.35	82.32	0.0865
0.50	36.00	13.80	1.23	18.00	0.0807
0.51	36.51	14.75	1.21	14.70	0.0782
0.61	43.17	17.14	1.35	102.68	0.0856
0.58	41.56	14.61	1.39	29.44	0.0903
0.59	42.00	14.80	1.40	45.12	0.0909
0.56	40.42	15.08	1.33	34.44	0.0858
0.57	40.74	17.04	1.26	13.32	0.0816
0.46	32.57	13.38	1.15	16.08	0.0762
0.55	39.95	17.40	1.22	25.20	0.0768
0.51	37.05	14.96	1.22	16.38	0.0792
0.55	39.68	14.20	1.34	40.02	0.0870
0.51	37.01	13.83	1.27	40.32	0.0829
0.63	44.13	17.06	1.39	123.09	0.0890
0.62	43.66	17.37	1.36	121.68	0.0860
0.56	40.58	15.74	1.31	85.25	0.0843
0.46	32.72	12.14	1.22	16.65	0.0822
0.56	40.42	15.17	1.32	36.10	0.0870
0.62	43.61	19.95	1.27	28.56	0.0785
0.49	34.90	15.11	1.15	22.95	0.0744
0.60	42.44	18.48	1.27	25.52	0.0800
0.54	39.10	15.39	1.27	44.46	0.0814
0.61	43.11	16.04	1.39	65.60	0.0896
0.51	36.77	16.19	1.16	47.00	0.0742
0.46	32.35	11.37	1.25	8.51	0.0847
0.51	36.79	16.02	1.17	18.36	0.0751
0.56	40.34	16.53	1.27	32.00	0.0809
0.52	37.27	13.76	1.28	36.54	0.0837
0.77	50.11	20.99	1.55	34.56	0.0954
0.56	40.59	14.67	1.35	21.06	0.0881
0.57	41.12	15.56	1.33	89.60	0.0860
0.50	36.00	12.05	1.32	19.00	0.0885
0.45	31.07	11.01	1.23	21.45	0.0836
0.70	47.37	23.93	1.31	40.70	0.0799
0.56	40.27	18.29	1.20	19.44	0.0757
0.47	33.75	15.70	1.10	9.36	0.0705
0.53	38.05	12.34	1.38	55.40	0.0925
0.43	29.54	11.91	1.14	13.93	0.0771
0.56	40.15	15.74	1.29	43.68	0.0833
0.60	42.44	17.99	1.29	28.42	0.0815
0.54	38.67	13.05	1.36	26.88	0.0886
0.54	38.80	14.58	1.29	71.04	0.0840
0.58	41.75	12.70	1.50	32.48	0.1004
0.58	41.23	18.99	1.21	41.10	0.0753
0.59	41.91	17.78	1.28	67.50	0.0805
0.50	35.74	13.12	1.26	47.16	0.0832
0.62	43.50	18.97	1.30	61.94	0.0803

0.55	39.61	15.39	1.29	70.75	0.0829
0.55	39.33	15.06	1.29	29.90	0.0832
0.50	36.29	14.15	1.23	8.16	0.0815
0.58	41.29	17.14	1.28	55.97	0.0809
0.52	37.50	13.69	1.29	46.20	0.0845
0.52	37.32	15.24	1.22	28.62	0.0789
0.44	30.33	13.53	1.09	3.18	0.0732
0.57	41.06	17.37	1.26	33.35	0.0796
0.47	33.71	12.96	1.21	16.44	0.0803
0.49	35.21	11.82	1.31	17.82	0.0879
0.56	40.34	15.73	1.30	25.00	0.0837
0.60	42.51	14.20	1.45	28.00	0.0958
0.56	40.56	16.76	1.26	104.32	0.0797
0.49	35.18	13.91	1.21	15.75	0.0793
0.48	34.63	12.78	1.24	15.75	0.0826
0.68	46.58	24.15	1.27	45.54	0.0758
0.61	43.05	18.70	1.29	48.00	0.0810
0.55	39.95	11.67	1.49	20.16	0.1023
0.50	36.00	13.33	1.26	53.76	0.0819
0.52	37.75	15.08	1.24	46.42	0.0798
0.57	40.75	16.27	1.29	65.12	0.0834
0.47	33.71	12.96	1.21	16.92	0.0803
0.53	37.97	16.80	1.18	13.68	0.0754
0.52	37.69	13.14	1.32	21.00	0.0868
0.49	34.77	13.58	1.21	20.55	0.0796
0.59	42.10	19.73	1.22	42.00	0.0765
0.54	38.62	16.54	1.21	17.42	0.0765
0.58	41.30	17.75	1.26	61.00	0.0797
0.53	38.34	15.42	1.24	20.14	0.0806
0.58	41.27	16.73	1.29	49.17	0.0816
0.51	37.06	15.27	1.21	10.15	0.0783
0.56	40.47	17.14	1.25	33.48	0.0791
0.49	35.03	15.42	1.14	26.39	0.0738
0.58	41.63	16.41	1.32	215.84	0.0829
0.60	42.44	17.14	1.32	130.56	0.0837
0.59	42.09	16.05	1.35	30.45	0.0869
0.57	40.71	14.81	1.35	47.25	0.0878
0.52	37.65	14.41	1.26	41.79	0.0822
0.53	38.50	11.79	1.42	16.28	0.0962
0.61	43.25	16.01	1.40	27.94	0.0919
0.52	37.48	12.91	1.33	30.13	0.0876
0.53	38.17	13.44	1.32	39.75	0.0868
0.51	37.16	14.39	1.25	31.08	0.0799
0.46	32.28	14.45	1.10	6.57	0.0728
0.49	34.89	14.19	1.18	24.92	0.0778
0.63	44.26	19.28	1.32	52.70	0.0823
0.67	46.20	17.94	1.45	69.96	0.0913
0.61	43.06	18.59	1.29	68.58	0.0818
0.54	39.25	16.68	1.22	38.50	0.0782
0.68	46.49	19.65	1.40	92.40	0.0869
0.53	38.53	15.42	1.25	32.34	0.0807

0.48	33.95	12.44	1.24	19.20	0.0825
0.48	34.11	13.96	1.17	24.48	0.0765
0.61	43.42	18.76	1.30	108.03	0.0806
0.64	44.60	19.38	1.33	260.40	0.0819
0.59	42.02	18.19	1.27	37.25	0.0804
0.60	42.74	16.41	1.36	40.32	0.0877
0.54	38.63	14.67	1.28	28.80	0.0842
0.59	42.21	18.98	1.25	45.82	0.0780
0.59	42.15	15.65	1.37	30.80	0.0902
0.59	41.93	15.31	1.38	33.33	0.0885
0.59	41.89	16.09	1.34	59.20	0.0856
0.49	35.20	14.52	1.18	18.36	0.0767
0.48	34.31	13.57	1.19	22.23	0.0790
0.56	40.00	16.19	1.27	48.06	0.0808
0.57	40.88	16.74	1.28	32.64	0.0819
0.53	38.37	15.39	1.24	47.70	0.0809
0.53	38.50	14.22	1.30	26.84	0.0849
0.56	40.37	15.04	1.33	29.29	0.0856
0.55	39.61	15.19	1.29	25.75	0.0837
0.53	38.47	16.80	1.19	38.41	0.0758
0.49	35.49	13.67	1.23	42.84	0.0799
0.63	44.35	18.42	1.35	170.28	0.0853
0.55	39.90	13.88	1.36	24.24	0.0899
0.60	42.67	17.19	1.33	47.25	0.0837
0.50	35.73	13.22	1.25	27.15	0.0833
0.56	40.10	14.21	1.36	39.25	0.0889
0.59	42.26	16.59	1.34	43.56	0.0849
0.53	38.50	14.52	1.28	34.32	0.0838
0.66	45.89	16.40	1.50	23.45	0.0973
0.52	37.73	16.57	1.18	27.83	0.0747
0.56	40.19	14.24	1.36	39.20	0.0885
0.58	41.33	16.73	1.29	162.88	0.0819
0.51	37.01	14.51	1.24	59.01	0.0802
0.64	44.94	20.89	1.29	60.48	0.0797
0.51	36.51	15.20	1.19	27.00	0.0768
0.54	39.12	14.32	1.31	97.28	0.0867
0.53	38.17	14.21	1.29	48.75	0.0836
0.58	41.41	17.94	1.25	43.47	0.0791
0.61	43.19	17.35	1.34	62.00	0.0854
0.50	36.00	12.96	1.27	22.88	0.0849
0.61	43.39	16.89	1.37	28.56	0.0871
0.56	40.35	13.60	1.40	39.78	0.0906
0.53	38.44	15.06	1.26	46.08	0.0813
0.51	36.77	15.13	1.20	21.80	0.0776
0.48	34.75	14.81	1.16	15.54	0.0753
0.60	42.82	19.60	1.25	32.70	0.0778
0.58	41.48	17.48	1.27	108.68	0.0808
0.53	38.26	14.74	1.27	179.92	0.0816
0.60	42.75	19.03	1.27	34.50	0.0800
0.64	44.70	18.42	1.37	44.54	0.0862
0.65	45.26	21.21	1.30	60.48	0.0798

0.49	34.97	13.67	1.21	25.60	0.0789
0.55	39.76	17.11	1.22	67.23	0.0774
0.63	44.48	16.73	1.42	28.22	0.0912
0.68	46.42	22.70	1.30	88.16	0.0795
0.59	42.02	14.96	1.40	59.85	0.0898
0.64	44.97	19.67	1.33	49.30	0.0841
